# Physiology-inspired bifocal fronto-parietal tACS for working memory enhancement

**DOI:** 10.1016/j.heliyon.2024.e37427

**Published:** 2024-09-06

**Authors:** Monika Pupíková, Pablo Maceira-Elvira, Sylvain Harquel, Patrik Šimko, Traian Popa, Martin Gajdoš, Martin Lamoš, Umberto Nencha, Kristína Mitterová, Adam Šimo, Friedhelm C. Hummel, Irena Rektorová

**Affiliations:** a Applied Neuroscience Research Group, Central European Institute of Technology, Masaryk University, Brno, Czech Republic; b Neuro-X Institute (INX), École Polytechnique Fédérale de Lausanne (EPFL), Chemin des Mines 9, 1202, CH, Geneva, Switzerland; c Neuro-X Institute (INX), EPFL Valais, Clinique Romande de Réadaptation Sion, Switzerland; d International Clinical Research Center, Faculty of Medicine and St. Anne's University Hospital, Masaryk University, Brno, Czech Republic; e Brain and Mind Research, Central European Institute of Technology, Masaryk University, Brno, Czech Republic; f First Department of Neurology, Faculty of Medicine and St. Anne's University Hospital, Masaryk University, Brno, Czech Republic; g Clinical Neuroscience, University of Geneva Medical School, Geneva, Switzerland

**Keywords:** tACS, Multifocal, Orchestrated brain stimulation, Healthy aging, Cognition, Systems neuroscience, Electric field modelling, Neuroimaging, Working memory

## Abstract

Aging populations face significant cognitive challenges, particularly in working memory (WM). Transcranial alternating current stimulation (tACS) offer promising avenues for cognitive enhancement, especially when inspired by brain physiology. This study (NCT04986787) explores the effect of multifocal tACS on WM performance in healthy older adults, focusing on fronto-parietal network modulation. Individualized physiology-inspired tACS applied to the fronto-parietal network was investigated in two blinded cross-over experiments. The first experiment involved monofocal/bifocal theta-tACS to the fronto-parietal network, while in the second experiment cross-frequency theta-gamma interactions between these regions were explored. Participants have done online WM tasks under the stimulation conditions. Network connectivity was assessed via rs-fMRI and multichannel electroencephalography. Prefrontal monofocal theta tACS modestly improved WM accuracy over sham (d = 0.30). Fronto-parietal stimulation enhanced WM task processing speed, with the strongest effects for bifocal in-phase theta tACS (d = 0.41). Cross-frequency stimulations modestly boosted processing speed with or without impairing task accuracy depending on the stimulation protocol. This research adds to the understanding of physiology-inspired brain stimulation for cognitive enhancement in older subjects.

## Highlights

• Individualized frontal monofocal theta tACS enhanced high load working memory (WM) task accuracy in healthy older subjects.
• Fronto-parietal cross-frequency theta-gamma tACS enhanced WM processing speed at the expense of task accuracy in most cases.
• Fronto-parietal theta in-phase tACS enhanced WM processing speed without any detrimental impact on task accuracy.
• Magnitude and symmetry of induced electric fields in bi-focal theta tACS was related to tACS-induced speed enhancement.

## 1 Introduction

Global demographics indicate a rising proportion of older adults. Despite prolonged life expectancy, the duration of healthy cognitive years has remained unchanged [1]. Working memory (WM) represents a vital system, maintaining and manipulating transient information necessary for diverse tasks, which deteriorates with aging [2]. The age-compromised efficacy of WM can be attributed to various neurobiological factors, with notable changes in the functioning of fronto-parietal networks. Animal and human research has consistently emphasized integrative interactions between the prefrontal and parietal cortices for WM [[3], [4], [5], [6]].

The coordinated interplay within and between brain regions [7] orchestrated by brain oscillations and their intra- and cross-frequency interactions have been shown to be central to WM processes [8,9]. The fascinating multi-tiered organization of these oscillations has been described, revealing how they interconnect functional brain systems across diverse spatiotemporal scales, providing the brain with a sophisticated solution for handling cognitive tasks [10,11]. Theta oscillations (4–8 Hz) in particular coordinate neural assemblies, ensuring the temporal consistency of items retained in WM [12]. A specific form of cross-frequency coupling – theta (4–8 Hz) gamma (>30 Hz) phase-amplitude coupling (PAC) – where the amplitude of faster gamma rhythms aligns with the phase of the slower theta rhythms, was shown to temporally organize information in WM. Nested gamma cycles may code for individual items held in WM [12,13].

Based on the current understanding of key nodes of the fronto-parietal cognitive network, non-invasive brain stimulation (NIBS) interventions have been employed to modulate their activity and enhance our understanding of their roles in WM processes [[14], [15], [16], [17]]. Most studies targeted single nodes in the dorsolateral prefrontal cortex, inferior frontal gyrus, inferior parietal lobule, and superior temporal gyrus using either repetitive transcranial magnetic stimulation or transcranial direct current stimulation [[18], [19], [20], [21], [22], [23]]. These studies did not demonstrate any conclusive results in terms of which brain region should be targeted for enhancing distinct WM functions in specific age/disease groups. Based on the understanding of the functional role of oscillatory regional and interregional interactions, a different concept of interventional strategies based on transcranial alternating-current stimulation (tACS) has been developed and evaluated [3,24,25]. tACS has shown potential in selectively modulating large-scale cortical networks in a causal, frequency and phase specific manner with enduring behavioral effects [3,17,[24], [25], [26]]. Growing evidence from tACS studies implies the feasibility of modulating WM processing and capacity by targeting cortical regions with theta-band tACS [3,27]. Notably, stimulation of the left prefrontal cortex with a complex theta-gamma waveform during a WM task led to a noticeable modulation of WM performance in healthy young people [3]. This opens an interesting interventional window to enhance WM in healthy older people.

Our current study assesses how the principles of intra-areal and inter-areal synchronization and cross-frequency coupling impact WM performance in healthy older subjects following two tACS experiments. In the first experiment, monofocal theta tACS and synchronized theta tACS between two distant regions were tested in the same subjects. The latter stimulation protocol is motivated by the idea of increasing interregional synchronization and connectivity within a network [24,26,28,29], with the hypothesis that a reduced phase lag (in-phase stimulation) between sites would promote an optimal inter-areal coupling and thus, optimal communication and efficiency [30,31]. The second theoretical framework examined here relates to the concept of cross-frequency theta-gamma PAC during WM. In an effort to synchronize cross-regional neural activities, we employed cross-frequency theta-gamma entrainment of interregional oscillatory interactions within the fronto-parietal network via bifocal tACS.

We established spatially and temporally individualized protocols for both experiments. Our primary outcome was WM task performance (normalized accuracies and response times) as measured by an *n*-back task. As little is known about factors causing interindividual differences in stimulation responses, we explored EEG and electric field properties and their relations to stimulation outcome. Given the known association of PAC with WM performance [25], we examined whether the baseline PAC is associated with tACS responsiveness. Changes in the offline resting-state functional connectivity (rs-FC) within the stimulated network were also studied using fMRI as a potential readout of stimulation effects.

## 2 Methods

### 2.1 Study design and procedure

**Fig. 1:**
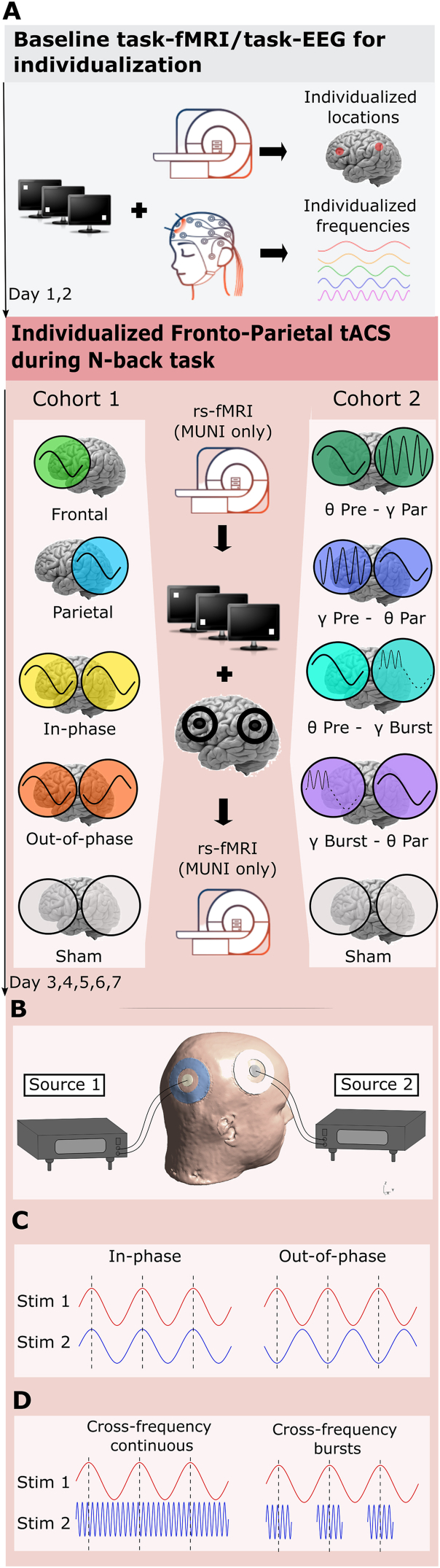
A. Design of the study. Prior to the stimulation sessions, participants underwent baseline multichannel scalp EEG during the task and task-fMRI measurements to personalize the stimulation parameters in terms of individual frequency and target location. Left: For the MUNI cohort, the stimulation protocols aimed to entrain oscillations within the theta range and synchronize fronto-parietal regions. Participants received monofocal stimulation in frontal or parietal areas and bifocal frontal and parietal stimulation in and out of phase. In the MUNI cohort only, resting-state fMRI (rs-fMRI) scans were acquired before and immediately after each stimulation session. The tACS and *n*-back tasks were conducted in the NIBS laboratory adjacent to the MRI scanner, with subjects easily moved between the two labs with a maximum of 5 min between the examinations. An alternating current was delivered using pairs of concentric rubber electrodes (Outer electrode: Ø 75 mm with a hole Ø 40 mm, inner electrode Ø 20 mm) Right: For the EPFL cohort, the stimulation protocols aimed to affect cross-frequency coupling between theta and gamma oscillations. Individualized gamma and theta cross-frequency tACS stimulation over the prefrontal and parietal cortex was applied. Continuous theta stimulation was applied while gamma stimulation was delivered either continuously or in bursts aligned to the theta peak. There was no before-and-after fMRI. **B.** Stimulation montage for bifocal stimulation. For monofocal stimulation, the electrode placement was the same, but only one current source was active. Image created with the use of SimNIBS software. Head is derived from a standard template provided. **C.** Stimulation protocols for inter-areal synchronization in experiment 1 (MUNI) **D.** Stimulation protocols for inter-areal cross-frequency stimulation in experiment 2 (EPFL).

**Fig. 2:**
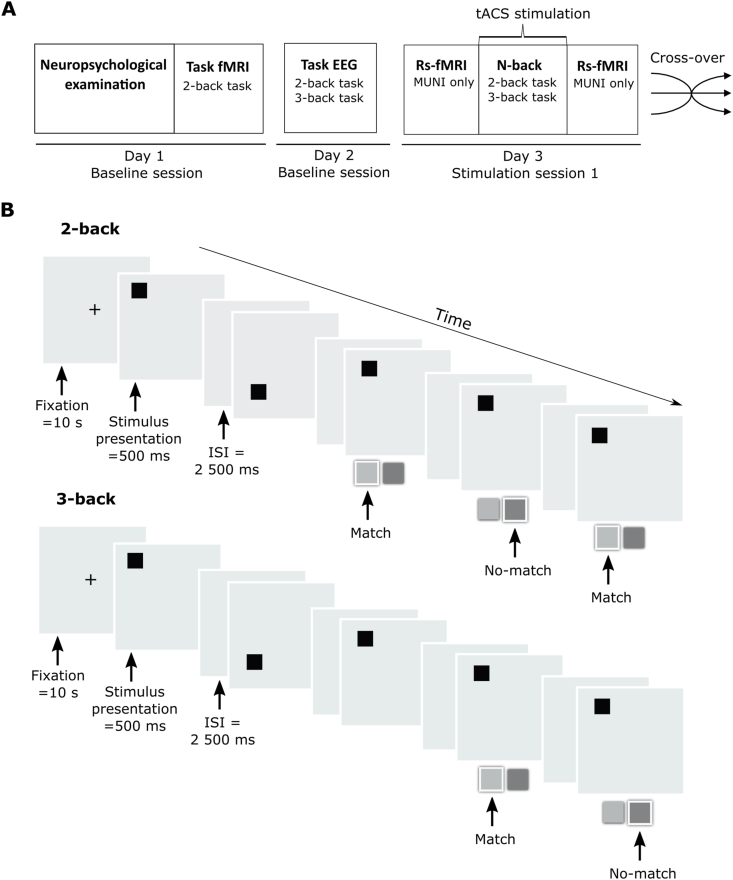
A. Schematic diagram of a study timeline. **B.** Main behavioral outcome – *n*-back task modified from Ref. [35]; task performed online during each stimulation condition. Participants completed two levels of difficulty during the stimulation. Participants indicate whether the current stimulus matches the one from *n* steps (2-back/3-back) earlier in the sequence. The task was performed also during fMRI to spatially individualize targets based on each participants activation.

This is a multicentric study consisting of two experiments on two cohorts investigating the effects of neuromodulation of the fronto-parietal network by means of mono- or bifocal theta-theta/theta-gamma tACS. An individualized approach in terms of spatial targets and stimulation frequency was adopted to maximize the personalization of the stimulation [24,25,[32], [33], [34]]. Each cohort underwent a randomized cross-over study with different tACS stimulation protocols, including sham stimulations, to determine the effects on WM-task performance (see Fig. 1). The primary outcome was the performance (normalized task accuracy and response times) during an *n*-back task conducted online during the stimulation (Fig. 2 and Supplementary materials for task description). The trial was registered (2021-04-19) in ClinicalTrial.gov under NCT04986787. See Supplementary materials for details.

### 2.2 Participants

In total, two cohorts of healthy participants were recruited, one in each research center (MUNI cohort: n = 20, EPFL cohort: n = 20), with intact cognition and no serious neuropsychiatric conditions (see Table 1 and Supplementary material). None of the participants had contraindications for either MRI or tACS. Each subject signed the informed consent form in accordance with the ethics codes and relevant regulations approved by the ethics committee of Masaryk University (EKV-2020-019) and from Swiss Ethics (SNCTP000003928 | BASEC2020-00127).

**Table 1:** Mean demographic, neuropsychological screening and EEG data., MoCA = Montreal Cognitive Assessment, SD = Standard deviation.

		Cohort	
		MUNI	EPFL
		n = 20	n = 20
Age	Mean	64.49	67.6
	SD	(6.98)	(4.70)
Men/Women Ratio		11/9	8/12
MoCA	Mean	27.21	26.35
	(SD)	(2.27)	(2.30)
Individual Theta (Hz)	Mean	4.51	4.64
	(Min-Max)	(4.17–4.98)	(4.32–5.28)
Individual Gamma (Hz)	Mean	–	34.04
	(Min-Max)	–	(30.06–37.04)

### 2.3 Transcranial alternating current stimulation

tACS was performed through two battery-driven stimulators attached to concentric electrodes placed over the task-fMRI individualized (see Supplementary materials) areas of the right middle frontal gyrus (rMFG) and right inferior parietal lobule (rIPL) for 20 min. Each concentric electrode pair was connected to a separate stimulator (current source, see Fig. 1B). A pair of concentric electrodes consists of smaller inner electrode, which is a target electrode (inner electrode Ø 20 mm) and a larger reference electrode around the target electrode (outer electrode: Ø 75 mm with a hole Ø 40 mm). The current flows between the inner and outer electrode. We used T1 MRI scan-based frameless stereotactic neuro-navigation to specify the exact location of the inner electrode center in each individual. The waveform of the stimulation was sinusoidal without DC offset. Individualized peak frequencies within the theta (4–7 Hz, both cohorts) and gamma (>30 Hz, EPFL cohort) range were calculated for each individual based on the pre-stimulation task EEG (see Table 1). The sham stimulation was applied with the same settings, but the stimulator was turned off after 30 s. For details see Fig. 1 and Supplementary materials. The impedance was kept below 20 kΩ.

#### 2.3.1 Inter-areal synchronization tACS – MUNI cohort

A continuous, sinusoidal stimulation within a theta range was applied at an intensity of 3 mA peak-to-peak. Protocols included monofocal theta stimulation over the frontal site only and over the parietal site only, and bifocal theta stimulation of both sites with a relative phase shift of 0° (in-phase) and 180°(out-of-phase). For monofocal stimulation protocols, electrodes were placed over both stimulation sites, despite only one of stimulators being active.

#### 2.3.2 Cross-frequency inter-areal tACS – EPFL cohort

This stimulation consisted of two components: continuous slow-wave theta frequency applied over the frontal or parietal cortices with concurrent fast gamma over the other pair of electrodes applied both continuously or in bursts. Gamma-burst stimulation was synchronized with the peaks within the continuous theta frequency and lasted 1000 ms. This results in four different stimulation protocols: slow theta over the frontal/parietal areas with continuous gamma (θ Pre - γ Par; γ Pre - θ Par resp., 2 mA peak-to-peak) or gamma applied in bursts (θ Pre - γ Burst, γ Burst - θ Par resp., 4 mA peak-to-peak) over the other area.

### 2.4 MRI data acquisition and pre-processing

The MRI data was acquired via 3.0 T Magnetom Siemens Prisma at both centers. For the structural MRI data, the T1 MPRAGE sequence was used. A gradient-echo, T2 echo-planar imaging sequence was used for the rs-fMRI and task-fMRI. The fMRI data were preprocessed and analyzed with SPM12 in MATLAB R2019a. For the data preprocessing pipeline and control for spatial abnormalities, see Supplementary materials.

### 2.5 Pre-stimulation task EEG data acquisition and baseline theta-gamma phase-amplitude coupling estimation

Participants underwent EEG recording concurrently with the pre-stimulation *n*-back task, in a shielded room using a 256-channel (MUNI) or 64 active channel (EPFL) system with task stimuli projected on a 150 cm screen. Task recording lasted approximately 20 min. The EEG sampling rate was 1 kHz with Cz (MUNI) or FCz (EPFL) as a reference electrode. EEG preprocessing involved epoching (between −7 and −10 to + 1 s relative to stimulus presentation for 2-back and 3-back tasks respectively), data filtering, artifact removal via ICA, and average re-referencing. PAC was computed in the −2.5 s–0 s time window using the Mean Vector Length method [36] on phase frequencies between 4 and 7 Hz (1 Hz step) and amplitude frequencies between 18 and 45 Hz (1 Hz step). Maximum electrode-level theta-gamma PAC was computed to assess WM load variations, contrasted between 2-back and 3-back tasks (contrast referred as Δ PAC scores) and analyzed for their relationship to performance. This approach aimed to better capture the PAC related to WM due to the variation in cognitive load across the tasks. The contrast was extracted from the frontal and parietal stimulation targets (frontal and parietal ROI). See Supplementary materials for more details.

### 2.6 Pre- and post-stimulation resting-state fMRI data analysis in MUNI cohort

After preprocessing, the resting-state functional connectivity (rs-FC) was assessed between the fronto-parietal control network (FPCN) major seeds using nine coordinates as described previously [37], see Supplementary material for details. We extracted fMRI signals from the region of interest (spheres, radius 6 mm) around seed positions and used means as representative signals. To analyze connectivity, we used the Pearson correlation between representative signals, converted to z values using the Fisher r-to-z transformation.

### 2.7 Montage current simulations

We simulated the electric fields (EF) induced at each stimulation site in all participants *a posteriori,* in order to estimate the influence of their magnitude on performance. Using SimNIBS [38], we constructed head models for each participant based on T1 and T2 MRI images. We extracted the magnitude of the EF induced within a 10 mm radius of each target. We calculated additional measures for the bifocal stimulation conditions, namely symmetry (i.e., relative magnitude of the field at one site with respect to the second site) and joint magnitude (i.e., the vectoral combination of the magnitude at both sites).

### 2.8 Statistical analysis

Data analyses were performed using R software (lme4 package). For the **behavioral data analysis**, our primary outcomes were normalized accuracy and normalized response times, which refer to the relative change in accuracy/response times as compared to the first block of each session. By normalizing the data to the first block of each session, we accounted for variability between sessions, as we did not measure pre-stimulation task performance on each stimulation sessions (see Fig. 2A). For response time, we considered the response times of the correct trials only. The behavioral data were checked for outliers (more than 3 SD from the group mean). We conducted a statistical analysis using linear mixed models (LMM) to investigate the interaction effect between the stimulation protocol and cognitive load on measures of normalized accuracy and response time, as well as accuracy and response time. The initial linear mixed model, referred to as the first-level model, included the stimulation protocol and cognitive load as fixed effects, along with their interaction. We then expanded the analysis to second-level models, which incorporated additional fixed factors such as training blocks, and random intercepts/factors of subject and days (for detailed model choice process, please refer to the reports on statistics). To ensure the appropriateness of our models, we assessed their fit by comparing the Akaike information criterion (AIC) and the Bayesian information criterion (BIC). These criteria take into account model overfitting by penalizing the number of parameters included in the model. In the MUNI cohort, the final LMM for normalized accuracy included also random intercept for subjects and days, and the LMM for normalized speed included random intercept for subjects. In the EPFL cohort, the final LMM for normalized accuracy included fixed effect for blocks with no additional random intercept/factors, and the LMM for normalized speed included a random intercept for days and subjects. For the **resting-state fMRI analysis**, LMM for each network pair were calculated. The differences of the z values of the connectivity changes (FC post–FC pre) were used as a dependent variable. For the final model, the variable stimulation protocol was used as an independent variable imputed as fixed effect, with no random intercepts or factors, as they did not improve the model fit. Post-hoc analyses were performed for the significant connectivity seed pairs. We further correlated the differences of the z values of the connectivity changes (FC post–FC pre) with within-session learning curves of accuracy and response time separately for each stimulation protocol. **For montage current simulations**, the LMM were computed to assess the interaction between stimulation protocol, cognitive load, and magnitude at each site (2 variables)/symmetry/joint magnitude which were imputed as fixed effects on measures of normalized accuracy and response time, as well as accuracy and response time (RT). For **pre-stimulation Δ PAC scores,** behavioral outcomes (normalized accuracy and RT) were taken as dependent variable with stimulation condition, Δ PAC Frontal and Δ PAC parietal were taken as fixed factors with no additional random intercepts/factors**.** Statistical significance (threshold p < 0.05) was assessed using the ANOVA function with Satterthwaite's approximations in the lmerTest package. Post-hoc pairwise comparisons were conducted by computing the estimated marginal means using the emmeans package. Effect sizes are reported as partial eta-squared (pη2) for F tests and Cohen's d for pairwise comparisons and were obtained using the effectsize package.

## 3 Results

### 3.1 Experiment 1. effect of monofocal and bifocal theta tACS on working memory (MUNI)

**Fig. 3:**
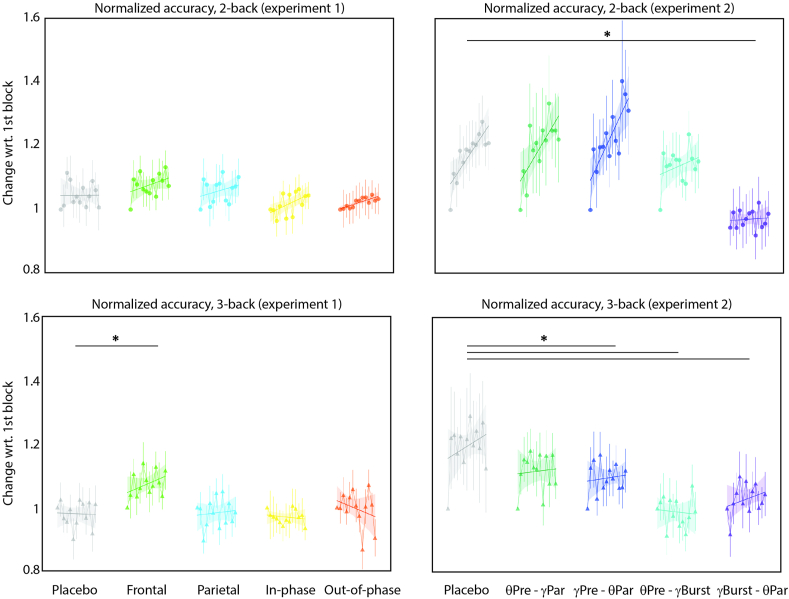
Normalized accuracy in the *n*-back tasks for the MUNI (experiment 1) and EPFL (experiment 2) cohorts. *sig. p < 0.05.

The participants in the MUNI cohort received either monofocal tACS to frontal/parietal areas or bifocal tACS to fronto-parietal regions (i.e., in-phase and out-of-phase). The accuracy was significantly different among the *n*-back task conditions (F_[1,2567]_ = 240.37, η^2^ = 0.09, p < 0.0001). When examined separately, we did not find a significant difference among the interventions in either the 2-back (F_[4,1243]_ = 1.16, η^2^ = 0.0037, p = 0.32) or the 3-back tasks (F_[4,1268]_ = 0.62, η^2^ = 0.0019, p = 0.64; Fig. S1 Left). When comparing the accuracy normalized to the first block, we found a significant interaction between the stimulation and the tasks (F_[4,2567]_ = 8.30, η^2^ = 0.01, p < 0.0001). Participants had a significantly larger accuracy increase in the 3-back task when receiving monofocal frontal stimulation compared to the sham condition (T_[1273]_ = 3.48, d = 0.30, p = 0.004; see Fig. 3 left).

**Fig. 4:**
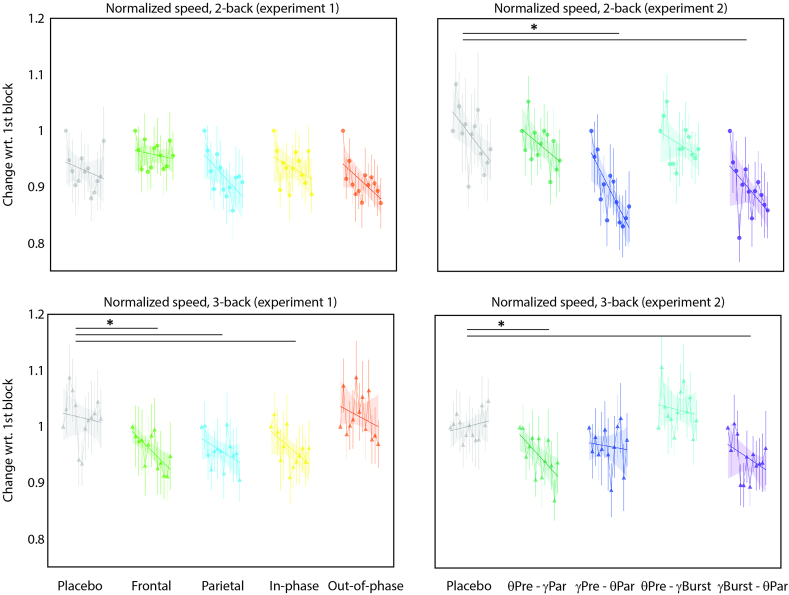
Normalized speed in the *n*-back tasks for the MUNI (experiment 1) and EPFL (experiment 2) cohorts. *sig. p < 0.05.

We found response times to differ significantly among tasks (F_[1,2571]_ = 89.83, η^2^ = 0.03, p < 0.0001). However, they did not vary significantly between the stimulation protocols in either the 2-back (F_[4,1276]_ = 1.60, η^2^ = 0.005, p = 0.16) or the 3-back (F_[4,1276]_ = 1.88, η^2^ = 0.005, p = 0.11) tasks (see Fig. S2 Left). In terms of the normalized speed, we found the change to be different among the tasks (F_[1,2567]_ = 48.55, η^2^ = 0.02, p < 0.0001). Post-hoc comparisons revealed that participants experienced significantly different changes in speed in the 3-back task when receiving monofocal frontal (d = 0.27), monofocal parietal (d = 0.286), and in-phase bifocal (d = 0.41) stimulations as compared to the sham stimulation, without compromising task accuracy (see Fig. 4 left).

### 3.2 Experiment 2. effects of cross-frequency tACS on working memory. (EPFL)

The participants in the EPFL cohort received cross-frequency tACS in individualized gamma and theta frequencies applied over the prefrontal and parietal cortices, with theta stimulation kept constant and gamma stimulation applied either continuously or in bursts. In regard to accuracy, a significant interaction between the tasks and the blocks of each difficulty was found (F_[1,2454]_ = 13.18, η^2^ = 0.0053, p = 0.0002), suggesting performance changing at different rates. No significant difference was found between the stimulation protocols in either the 2-back (F [4,39] = 0.72, η^2^ = 0.06, p = 0.57) or the 3-back (F_[4,83]_ = 0.32, η^2^ = 0.02, p = 0.86) tasks compared to sham (see Fig. S1 Right). For normalized accuracy, significant effects of the stimulation were observed in both the 2-back (F [4] = 14.29, η^2^ = 0.04, p < 0.0001) and the 3-back (F [4] = 12.29, η^2^ = 0.04, p < 0.0001) tasks. In the 2-back task, the participants experienced a significantly smaller improvement in accuracy when receiving parietal-theta-prefrontal-gamma-burst tACS (T_[1238]_ = 5.52, d = 0.49, p < 0.0001); in the 3-back tasks, participants experienced a significantly smaller improvement in accuracy in all interventions except for prefrontal-theta-parietal-gamma-continuous, when compared to sham stimulation (see Fig. 3 right). In other words, most of the active stimulation protocols led to normalized accuracy impairment compared to sham.

There was a significant difference in response time among the tasks (F_[1,2452]_ = 4.42, η^2^ = 0.001, p = 0.03). In the 3-back task, there was a significant difference among the stimulation types (F [4,40] = 4.29, η^2^ = 0.24, p = 0.004), with participants being significantly faster with the parietal-theta-prefrontal-gamma-burst stimulation compared to the sham (T [4] = 4.6, d = 0.74, p = 0.03; see Fig. S2 right). The normalized speed differed among the tasks (F [1] = 14.22, η^2^ = 0.005, p = 0.0001) with a significant effect of stimulation in both the 2-back (F [4] = 10.45, η^2^ = 0.03, p < 0.0001) and the 3-back (F [4] = 7.91, η^2^ = 0.02, p < 0.0001) tasks. In the 2-back, the change in speed was significantly larger in both the prefrontal-gamma-continuous and theta-parietal (T_[1225]_ = 4.83, d = 0.42, p < 0.0001) and the prefrontal-theta and parietal-gamma-burst (T_[1225]_ = 4.46, d = 0.40, p = 0.0001). In the 3-back task, participants experienced a significantly larger change in speed when receiving the prefrontal-theta and parietal-gamma-continuous (T_[1237]_ = 2.96, d = 0.24, p = 0.02) and the parietal-theta and prefrontal-gamma-burst (T_[1237]_ = 3.04, d = 0.27, p = 0.02) tACS stimulation (see Fig. 4 right).

### 3.3 Effect of monofocal and bifocal theta tACS upon rs-fMRI connectivity

**Fig. 5:**
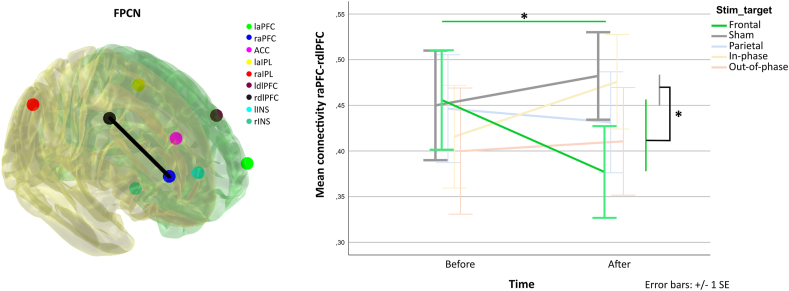
A Visualization of FPCN network seeds used for the rs-fMRI data analysis. raPFC-rdlPFC connection is highlighted by a line. **B** Connectivity between the raPFC-rdlPFC. Legend: l/raPFC = left/right anterior prefrontal cortex, ACC – anterior cingulate cortex, l/raIPL = left/right anterior intraparietal lobule, l/rdlPFC = left/right dorsolateral prefrontal cortex, l/rINS = left/right insula.

Within the fronto-parietal network only, the connectivity change between the right anterior prefrontal cortex (raPFC) and right dorsolateral prefrontal cortex (rdlPFC) (F [4] = 2.66, p = 0.04) was affected by the stimulation condition and was significantly different from the sham stimulation condition (t [41] = 2.33, p = 0.022). This difference resulted from a decrease in raPFC-rdlPFC connectivity following stimulation compared to pre-stimulation levels (t [38] = 2.068, p = 0.046, see Fig. 5). Connectivity changes were not correlated with any behavioral changes caused by the stimulation. For full results see Supplementary material and the Report on Statistics.

### 3.4 PAC scores and effect of stimulation

We found a relationship between the Δ PAC scores at the parietal target and the normalized accuracy under different stimulation conditions (F [3] = 2.78, η^2^ = 0.12, p = 0.04). Post-hoc comparisons revealed that the relationship of parietal Δ PAC scores and normalized accuracy differed between the frontal theta condition and bifocal theta stimulation conditions (frontal vs. in-phase p = 0.033; frontal vs. out-of-phase p = 0.046). Notably, the correlations between parietal Δ PAC scores and the relevant mean normalized accuracies were not significant. For all results, see Supplementary materials.

### 3.5 Influence of the magnitude of the induced electric fields (EFs) on WM enhancement: experiment 1

We found effects of individually induced EFs on normalized response times, but not on normalized accuracies in the bifocal theta-theta tACS conditions. We found a significant triple interaction between the stimulation and the magnitude of the EF induced in the prefrontal target and that of the parietal target (F [1] = 5.05, η^2^ = 0.07, p = 0.02). To explore the interaction, we introduced a feature representing the symmetry of EF at the prefrontal and parietal targets, defined by their ratio, and the vector magnitude of both fields to measure the total injected energy (see Table S2). These metrics were integrated into the linear mixed models, replacing the frontal/parietal magnitude variables. Our analysis confirmed significant differences in response time changes across stimulation conditions for symmetry of EFs (F [1] = 5.68, η^2^ = 0.1, p = 0.02), but not for their joint magnitude (F [3] = 1.17, p = 0.33).

**Fig. 6:**
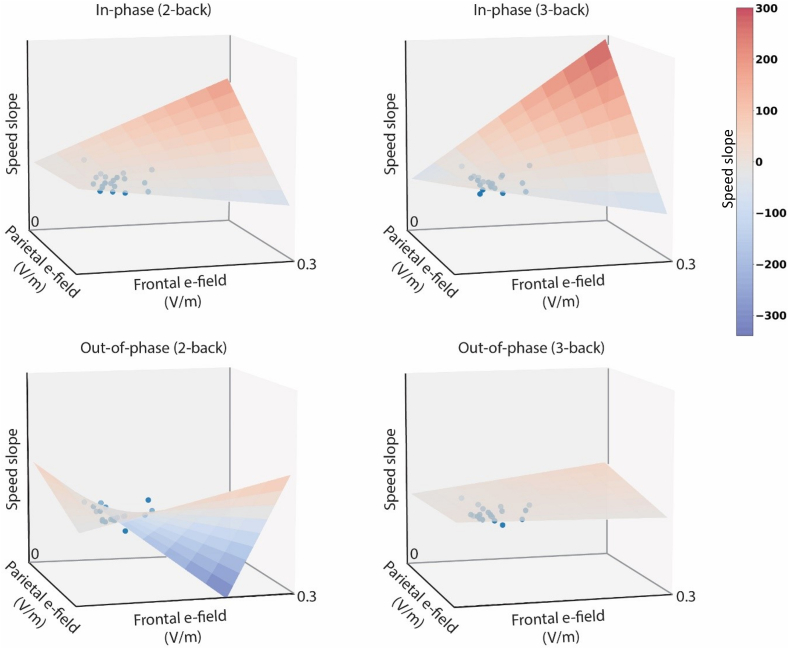
Visualization of triple interactions among the bifocal stimulation conditions and the magnitude of the EFs induced at both stimulation sites (i.e., prefrontal and parietal cortices) acting on the speed in the 2-back and the 3-back task. The dots represent individual datapoints; the planes reflect the statistical model adjusted to these points. In the in-phase condition, the model describes steeper improvements in speed predicted for individuals exposed to EFs of higher magnitude. In contrast, the model describes speed decreases in individuals receiving out-of-phase stimulation who were predicted to have speed decreases when exposed to EFs of higher magnitude. This detrimental effect is only apparent in the 2-back and not the 3-back task. Note that the vertical axis has the same scale in all four panels. Even though there is no numerical grading along the axes, all plots have the same scale.

The stimulation-related differences appear to be most pronounced when both EF magnitudes were similar at both stimulation sites, in both the in-phase and the out-of-phase conditions, with the opposite direction of changes (see Fig. 6). In the in-phase condition, higher EF magnitudes predicted more pronounced reaction time enhancement. Conversely, out-of-phase stimulation with higher EF magnitudes led to more pronounced speed decrements, especially in the 3-back task.

### 3.6 Influence of the magnitude of the induced EF on WM enhancement: experiment 2

**Fig. 7:**
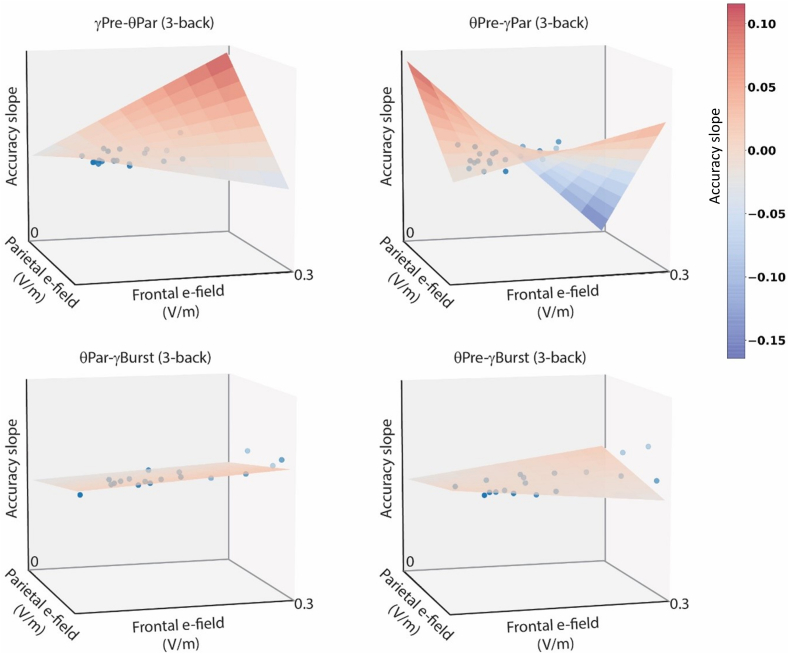
Visualization of triple interactions among the stimulation and the magnitude of the EFs induced at both stimulation sites (i.e., prefrontal and parietal cortices) acting on the accuracy in the 3-back task. The dots represent individual datapoints; the planes reflect the statistical model adjusted to these points. Specifically, when applying tACS within the gamma band to the prefrontal cortex and theta-tACS to the parietal cortex (stimulation condition 1), the rate of improvement in accuracy within a training session became steeper as a function of the magnitude of the induced EF, and this effect was more pronounced when the magnitude was similar at both stimulation sites. In contrast, when applying theta to the prefrontal cortex and gamma tACS to the parietal cortex (stimulation condition 2), the rate of improvement was steepest when the EFs were lateralized, and least pronounced when they were both symmetric and of high intensity. These interactions seem to be more attenuated in both conditions including gamma bursts (i.e., stimulation conditions 3 and 4). Note that even though there is no numerical grading along the axes, all the vertical axes in all plots have the same scale.

We found effects of the individually induced EF on normalized accuracies, but not on normalized response times in the cross-frequency tACS protocols. We observed a significant triple interaction between the stimulation and the magnitude of electrical fields at both stimulation sites (F [3] = 3.04, η^2^ = 0.7, p = 0.03), which was particularly significant in the 3-back task (F [3] = 4.08, η^2^ = 0.17, p = 0.01). The accuracy change was modulated differently across conditions (see Fig. 7). In continuous conditions, with gamma tACS on the prefrontal cortex and theta-tACS on the parietal cortex, accuracy improvement increased with higher EF magnitudes, particularly when fields at both sites were similar. In contrast, when theta tACS was applied to the prefrontal cortex and gamma tACS to the parietal cortex, the greatest improvement occurred with lateralized fields, decreasing with symmetric and intense fields. Additional modeling to examine these interactions revealed no significant effects for either symmetry or joint magnitude of the EFs.

## 4 Discussion

An effective exchange of information across neural structures necessitates the spatiotemporal coordination of oscillatory activity as outlined by oscillatory hierarchy [9,42]. Slower frequencies such as theta oscillations foster this inter-areal, cross-frequency neural communication. Specifically, increased fronto-parietal theta synchrony is associated with the maintenance and manipulation of information in WM [43,44]. Older populations were shown to be predisposed toward long-range theta desynchronization in comparison to younger adults [25,[39], [45], [46]]. We explored the online cognitive effects of individualized, monofocal, and multifocal physiology-based tACS in healthy older adults, focusing on enhancing intra-areal and inter-regional cognitive processing within the fronto-parietal network. To achieve these goals, we studied: (1) the entrainment of individualized theta-band neural oscillations at frontal and parietal sites separately as well as synchronously, and (2) inspired by the role of physiological theta-gamma coupling in WM [3,4], we examined the effects of cross-frequency theta and gamma tACS applied to the prefrontal and parietal cortices.

We found that the most pronounced effects were observed in the *n*-back task with higher cognitive loads. The monofocal prefrontal stimulation was the only stimulation condition that led to modest yet significant enhancements in WM task accuracy, surpassing other fronto-parietal stimulation protocols. On the other hand, fronto-parietal stimulation tended to modulate WM task processing speed with or without concomitant impairment of the task accuracy. Positive mild effects on response times without impacting the task accuracy were observed with the bifocal fronto-parietal theta in-phase tACS, followed by monofocal theta stimulation at parietal and frontal targets, and cross-frequency prefrontal theta and parietal gamma-continuous tACS.

### 4.1 Effects of intra-areal and inter-areal theta synchronization

Previous research has shown that tACS-induced phase synchronization of frontal and parietal regions improves WM performance [3,24,26,43], while desynchronization impairs it [47]. In our study, only the monofocal frontal stimulation provided modest accuracy modulation of the 3-back task as compared to sham stimulation. While no significant effect on accuracy was observed, the fronto-parietal in-phase condition significantly enhanced processing speed, particularly under higher WM loads (3-back), consistent with prior findings of faster reaction times using in-phase fronto-parietal stimulation [3,24,26,43,47,48]. Notably, this study extends previous works by examining monofocal conditions; both frontal and parietal monofocal stimulations led to slightly faster reaction times as compared to sham. There are some inconsistencies between prior monofocal stimulation studies and our findings. While the efficacy of frontal monofocal theta upon WM performance has been reported [49,50], a recent review identified the parietal node of the fronto-parietal network as superior for WM enhancement, both in reaction times and accuracies [51]. Discrepancies between our findings and the results of others may arise from using the *n*-back task, which strains the frontal lobe and its connections due to the higher emphasis of updating demands [52]. This contrasts with other studies that employed tasks focusing on pure WM capacity or simpler match-to-sample tasks (as listed in Nissim et al. [51]). Additionally, age might have been a major contributing factor. Unlike most prior studies involving young adults, we included older participants. Using resting state EEG, Perinelli et al. [53] reported decreased frontal intra-areal connectivity and lower modularity (less segregated network) in older versus younger adults; fronto-parietal and fronto-temporal inter-areal connectivity was rather increased [53,54]. Notably, connectivity differences between older and younger adults were mostly contributed by the theta (and alpha) band. Therefore, modulating frontal oscillation by monofocal theta tACS might bring significant (albeit modest) WM task improvement specifically in older subjects.

Interestingly, the behavioral effects of frontal monofocal tACS were accompanied by significant pre-post changes in resting state fMRI connectivity within the stimulated fronto-parietal network. Specifically, we observed a reduction in connectivity strength between the right anterior prefrontal cortex (aPFC) and the right dorsolateral prefrontal cortex, as opposed to unchanged connectivity in the sham condition. While Violante et al. [43] reported enhanced online connectivity within stimulated FPCN during theta in-phase tACS, Abellaneda-Perez et al. [55] showed, similarly to our study result, that prefrontal theta tACS led to post-versus pre-stimulation rs-connectivity decrease between discrete seeds of FPCN. While both the anterior prefrontal cortex and DLPFC are core components of the FPCN, they appear to have different roles in WM processing. The MFG is part of a subnetwork critical to WM performance, and aPFC seems to be part of a subnetwork more aligned with introspective processes [56], multitasking [40] or delayed response tasks [57]. The changes in offline rs-connectivity were not directly associated with the online behavioral changes in this study, pointing out that the offline rs-connectivity change is a coarse and indirect measure of the online tACS-induced network effects.

### 4.2 Effects of fronto-parietal theta-gamma cross-frequency tACS

Enhanced cross-frequency coupling, particularly gamma oscillations nested within theta cycles in prefrontal areas, have been linked to better WM performance [12]. This study expands upon this by exploring physiologically inspired multifocal stimulation and its effects on inter-areal interactions. Stimulation protocols involved the application of continuous slow-wave theta frequency over either the frontal or parietal cortices, coupled with concurrent fast gamma frequency stimulation over the other electrode pair, delivered either continuously or in bursts.

**Fig. 8:**
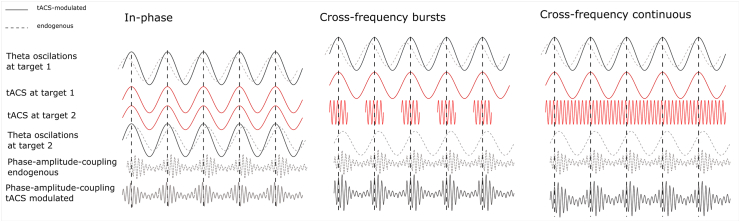
Visualization of the proposed misalignment between the externally induced fast gamma component and the inherent local theta rhythms in different stimulation conditions. Consequently, the theta-gamma coupling might be disproportionately skewed, which in turn would distort specific theta phase-locking present between prefrontal and distant posterior areas.

Unexpectedly, our stimulation conditions led to enhanced speed processing at the cost of task accuracy; this effect was more prominent in the WM task with a higher cognitive load. In fact, prefrontal-theta and parietal gamma-continuous tACS was the only configuration that led to a significant though modest increase in processing speed without a concomitant speed-accuracy trade-off. Our observation is in agreement with a recent meta-modeling study by Wischnewski et al. [58], which synthesizes findings from 28 placebo-controlled tACS studies. They showed dose-dependent improvement in working memory task performance after prefrontal theta and parietal gamma stimulation. Further, our finding that parietal-theta when paired with prefrontal-gamma stimulation negatively impacted WM performance aligns well with their reported detrimental effects on accuracy for prefrontal gamma modulation. Negative effects of prefrontal gamma stimulation were specific to accuracy; no detrimental effects were reported for response times. Speed-accuracy trade-off can manifest through the use of heuristics and mental shortcuts to ease the cognitive load associated with decision-making [59,60], potentially strengthened by our stimulation. Previous studies hypothesized engagement of the superior medial prefrontal cortices and the hyper-direct pathway connecting the prefrontal cortex to the subthalamic nucleus (STN) [[61], [62], [63]]; abnormal connectivity of the pathway has been implicated in impulse control disorders and might subserve the speed-accuracy trade-off [[64], [65], [66]]. Alternatively, the overall effectiveness of inter-areal cross-frequency coupling scenarios in the current study could have been compromised due to the misalignment between the externally induced fast gamma component and the inherent local theta rhythms leading to distorted theta-phase locking [67,68]; for visualization see Fig. 8), specifically in gamma-burst scenarios. Continuous gamma, delivered uniformly across theta phases, might be less affected allowing local theta to modulate gamma bursts through variations in local excitability [30]. Notably, inter-regional oscillatory mechanisms are dynamic, as recent studies show high-frequency gamma activity nested within various phases of the slow theta wave, depending on cognitive demand. This enables dynamic coupling or decoupling of the fronto-parietal control network to posterior areas, adjusted to cognitive effort [69].

Future studies should examine the integration of naturally occurring phase-lags in inter-areal theta activity and consider systems that superimpose both fast and slow rhythms in a closed-loop fashion. Specifically closed-loop systems that measure brain activity and apply targeted stimulation based on these measurements might be well suited in respect to optimal phase lag.

### 4.3 Individualization of stimulation paradigms for WM enhancement

In our study, we adopted an individualized approach, using each participant's activation maps and determining their peak frequencies, not to compare individualized versus standard methods but to maximize stimulation benefits based on current knowledge [32]. The substantial variation in targets within the large MFG region underscored the need for individualization. On the other hand, our study's mean frequency of 4.58 Hz aligns closely with the 4.5 Hz average reported in another tACS study [24], suggesting limited frequency variability. This narrow range of frequency variability raises questions about the necessity of frequency individualization, at least in older healthy subjects. A standardized frequency might suffice, thereby simplifying the tACS protocol without compromising efficacy.

The electrical field modelling data offer another possibility for individualization. Prior research indicates that individual anatomical differences lead to variations in electric field magnitude [70], affecting stimulation outcomes. In the present study, various stimulation condition outcomes depended on the magnitude and particularly on the symmetry of electrical fields induced at the two stimulated sites. This was most evident in the theta in-phase condition, where more balanced fields led to better behavioral effects, possibly more efficiently enhancing the fronto-parietal theta coherence crucial for demanding WM processes [44]. Thus, our results emphasize the need for pre-stimulation optimization by current modelling [71,41].

### 4.4 Limitations

This study has several limitations that require careful consideration. Notably, while our objective was to affect behavior by modulating the oscillatory activity within the fronto-parietal network in a physiology-inspired way by tACS, we only assessed behavioral changes without monitoring and adjusting the tACS to the individual “background” ongoing oscillatory brain activity by e.g. online EEG. Notably, EEG recording with concurrent tACS is currently limited due to tACS artifacts and problems with their elimination [72,73]. Another limitation is the fact that only one session of stimulation was studied. Further studies are needed to explore the effects of multiple tACS sessions. The fields induced beneath the electrodes were rather low in our study and could have led to small effects sizes [74]. This might stem from our focus on focality which is often achieved at the expense of field intensity [75,76]. Notably, we found that the symmetry of the induced electrical field magnitudes influenced the task performance during various stimulation conditions.

### 4.5 Conclusion

We used individualized, monofocal and multifocal, physiology-inspired tACS to modulate the fronto-parietal network with the aim of enhancing WM performance in healthy older individuals. The main effects were found for the 3-back task (with a higher cognitive load). Only prefrontal monofocal theta stimulation produced a modest yet significant improvement in WM task accuracy as compared to the sham. Fronto-parietal bifocal stimulation conditions enhanced WM task processing speed; however, this enhancement was at the expense of task accuracy in most cases, eventually leading to WM impairment. Fronto-parietal theta in-phase stimulation produced mild to moderate speed acceleration without any detrimental effect on task accuracy. The speed enhancement of this stimulation protocol was related to the magnitude and particularly to the symmetry of the induced electric fields at both stimulation targets, thus stressing the importance of current modelling for stimulation protocol optimization. Our research contributes to developing tACS-based intervention strategies to enhance WM in healthy older adults; further work is warranted in neurological conditions with WM deficits.

## Ethics statement

Each subject signed the informed consent form in accordance with the ethics codes and relevant regulations approved by the ethics committee of Masaryk University (EKV-2020-019) and from Swiss Ethics (SNCTP000003928 | BASEC2020-00127).

## Data availability statement

The datasets generated and/or analyzed during the current study are not publicly available due accordance with informed consents signed by study participants but are available from the corresponding author on a request.

## CRediT authorship contribution statement

**Monika Pupíková:** Writing – review & editing, Writing – original draft, Visualization, Methodology, Investigation, Formal analysis, Data curation, Conceptualization. **Pablo Maceira-Elvira:** Writing – review & editing, Visualization, Formal analysis, Data curation. **Sylvain Harquel:** Writing – review & editing, Visualization, Formal analysis, Data curation. **Patrik Šimko:** Writing – review & editing, Project administration, Investigation, Data curation, Conceptualization. **Traian Popa:** Writing – review & editing, Validation, Project administration, Methodology, Investigation, Data curation, Conceptualization. **Martin Gajdoš:** Validation, Software, Formal analysis, Data curation. **Martin Lamoš:** Software, Formal analysis, Data curation. **Umberto Nencha:** Investigation, Data curation. **Kristína Mitterová:** Investigation, Formal analysis. **Adam Šimo:** Investigation. **Friedhelm C. Hummel:** Writing – review & editing, Supervision, Methodology, Funding acquisition, Conceptualization. **Irena Rektorová:** Writing – review & editing, Supervision, Methodology, Funding acquisition, Conceptualization.

## Declaration of competing interest

The authors declare that they have no known competing financial interests or personal relationships that could have appeared to influence the work reported in this paper.
