# Supplementary material for: Physiology-inspired bifocal fronto-parietal tACS for working memory enhancement
Source: Heliyon. 2024 Sep 6;10(18):e37427. doi: 10.1016/j.heliyon.2024.e37427 (PMC11417162; doi:10.1016/j.heliyon.2024.e37427)
Supplement: Multimedia component 1 [file mmc1.zip › Supplementary materials_Reports on statistics_EPFL.nb.html]

iCOG MS stats for the EPFL data


Code 

- Show All Code
- Hide All Code
- Download Rmd

# iCOG MS stats for the EPFL data

renv::activate(“C:/Users/pablo.maceira/switchdrive/SD\_Cosmos/My\_documents/Projects/iCOG/Analysis/Stats/Clean/”)


```
library(Matrix)
```


```
Warning message:
In do_once((if (is_R_CMD_check()) stop else warning)("The function xfun::isFALSE() will be deprecated in the future. Please ",  :
  The function xfun::isFALSE() will be deprecated in the future. Please consider using base::isFALSE(x) or identical(x, FALSE) instead.
```


```
library(lme4)
library(carData)
library(car)
library(lmerTest)
library(emmeans)
library(effectsize)
library(ggplot2)
library(Rmisc)
#library(flexplot)
```


```
curr_dir <- getwd()
data_directory <- file.path(paste(curr_dir, '../../../Code/Notebooks/Results', sep='/'))

file_name <- file.path(paste(data_directory, "iCOG_all_data.txt", sep='/'))
df <- read.delim(file_name, header = TRUE, na.strings = "NN")
head(df, 5)
```


```
df$ID <- as.factor(df$ID)
df$School <- as.factor(df$School)
df$Day <- as.factor(df$Day)
df$Task <- as.factor(df$Task)
df$Block <- as.numeric(df$Block)
df$Stim <- as.factor(df$Stim)
df$Acc <- as.numeric(df$Acc)
df$Speed_corr <- as.numeric(df$Speed_corr)
df$D_prime <- as.numeric(df$D_prime)
df$Acc_norm <- as.numeric(df$Acc_norm)
df$Speed_norm <- as.numeric(df$Speed_norm)
df$D_prime_norm <- as.numeric(df$D_prime_norm)
```


# EPFL Data Results


```
df_epfl <- subset(df, School == 'EPFL')
df_epfl <- droplevels(df_epfl)
df_epfl$Stim <- factor(df_epfl$Stim, levels(df_epfl$Stim)[c(2, 1, 5, 3, 4)])
levels(df_epfl$Stim)
```


```
[1] "Placebo"   "GPre-TPar" "TPre-GPar" "Tpar-GBst" "Tpre-GBst"
```


## Accuracy Stats

### Simple model, averages

Here, we present different models to characterize each relevant
parameter related to the participants’ performance. In each case, the
models start off from a simple form (i.e., group averages per session),
and they grow in complexity as we add other fixed factors (e.g.,
training blocks) and random effects to account for the variability
across subjects. Each model is compared statistically to its (simpler)
predecessor to assess whether the eventual improvement in data
explainability, as quantified by two conventional criteria (i.e.,
Akaike’s and Bayes’ information criteria), justifies the use of a more
complex model.


```
m0 <- lm(formula = Acc ~ Stim*Task, data=df_epfl)
summary(m0)
```


```
Call:
lm(formula = Acc ~ Stim * Task, data = df_epfl)

Residuals:
     Min       1Q   Median       3Q      Max 
-0.81496 -0.09769  0.05077  0.15077  0.25731 

Coefficients:
                          Estimate Std. Error t value Pr(>|t|)    
(Intercept)              0.7976923  0.0122165  65.296  < 2e-16 ***
StimGPre-TPar            0.0080769  0.0172767   0.468  0.64018    
StimTPre-GPar            0.0200000  0.0172767   1.158  0.24713    
StimTpar-GBst            0.0172650  0.0177502   0.973  0.33081    
StimTpre-GBst            0.0155556  0.0177502   0.876  0.38092    
Task3back               -0.0450000  0.0172767  -2.605  0.00925 ** 
StimGPre-TPar:Task3back -0.0115385  0.0244330  -0.472  0.63679    
StimTPre-GPar:Task3back -0.0300000  0.0244330  -1.228  0.21962    
StimTpar-GBst:Task3back  0.0009829  0.0251025   0.039  0.96877    
StimTpre-GBst:Task3back -0.0212393  0.0251025  -0.846  0.39758    
---
Signif. codes:  0 ‘***’ 0.001 ‘**’ 0.01 ‘*’ 0.05 ‘.’ 0.1 ‘ ’ 1

Residual standard error: 0.197 on 2486 degrees of freedom
  (104 observations deleted due to missingness)
Multiple R-squared:  0.02271,   Adjusted R-squared:  0.01917 
F-statistic: 6.418 on 9 and 2486 DF,  p-value: 4.656e-09
```


```
anova(m0)
```


```
Analysis of Variance Table

Response: Acc
            Df Sum Sq Mean Sq F value   Pr(>F)    
Stim         4  0.091 0.02285  0.5890   0.6706    
Task         1  2.060 2.05965 53.0795 4.28e-13 ***
Stim:Task    4  0.090 0.02261  0.5827   0.6752    
Residuals 2486 96.465 0.03880                     
---
Signif. codes:  0 ‘***’ 0.001 ‘**’ 0.01 ‘*’ 0.05 ‘.’ 0.1 ‘ ’ 1
```


Linear model, no random effects


```
m1 <- lm(formula = Acc ~ Stim*Task*Block, data=df_epfl)
summary(m1)
```


```
Call:
lm(formula = Acc ~ Stim * Task * Block, data = df_epfl)

Residuals:
     Min       1Q   Median       3Q      Max 
-0.79444 -0.08010  0.05003  0.14959  0.26337 

Coefficients:
                                Estimate Std. Error t value Pr(>|t|)    
(Intercept)                    0.7478846  0.0258678  28.912   <2e-16 ***
StimGPre-TPar                  0.0011538  0.0365825   0.032   0.9748    
StimTPre-GPar                  0.0148077  0.0365825   0.405   0.6857    
StimTpar-GBst                  0.0431410  0.0375850   1.148   0.2512    
StimTpre-GBst                  0.0510470  0.0375850   1.358   0.1745    
Task3back                     -0.0026923  0.0365825  -0.074   0.9413    
Block                          0.0071154  0.0032590   2.183   0.0291 *  
StimGPre-TPar:Task3back       -0.0023077  0.0517355  -0.045   0.9644    
StimTPre-GPar:Task3back       -0.0213462  0.0517355  -0.413   0.6799    
StimTpar-GBst:Task3back       -0.0334188  0.0531532  -0.629   0.5296    
StimTpre-GBst:Task3back       -0.0347009  0.0531532  -0.653   0.5139    
StimGPre-TPar:Block            0.0009890  0.0046090   0.215   0.8301    
StimTPre-GPar:Block            0.0007418  0.0046090   0.161   0.8722    
StimTpar-GBst:Block           -0.0036966  0.0047353  -0.781   0.4351    
StimTpre-GBst:Block           -0.0050702  0.0047353  -1.071   0.2844    
Task3back:Block               -0.0060440  0.0046090  -1.311   0.1899    
StimGPre-TPar:Task3back:Block -0.0013187  0.0065181  -0.202   0.8397    
StimTPre-GPar:Task3back:Block -0.0012363  0.0065181  -0.190   0.8496    
StimTpar-GBst:Task3back:Block  0.0049145  0.0066967   0.734   0.4631    
StimTpre-GBst:Task3back:Block  0.0019231  0.0066967   0.287   0.7740    
---
Signif. codes:  0 ‘***’ 0.001 ‘**’ 0.01 ‘*’ 0.05 ‘.’ 0.1 ‘ ’ 1

Residual standard error: 0.1966 on 2476 degrees of freedom
  (104 observations deleted due to missingness)
Multiple R-squared:  0.03019,   Adjusted R-squared:  0.02275 
F-statistic: 4.057 on 19 and 2476 DF,  p-value: 8.422e-09
```


```
anova(m1)
```


```
Analysis of Variance Table

Response: Acc
                  Df Sum Sq Mean Sq F value    Pr(>F)    
Stim               4  0.091 0.02285  0.5912  0.669065    
Task               1  2.060 2.05965 53.2740 3.889e-13 ***
Block              1  0.355 0.35450  9.1694  0.002486 ** 
Stim:Task          4  0.090 0.02261  0.5849  0.673614    
Stim:Block         4  0.093 0.02319  0.5997  0.662878    
Task:Block         1  0.245 0.24486  6.3333  0.011912 *  
Stim:Task:Block    4  0.047 0.01166  0.3017  0.876953    
Residuals       2476 95.726 0.03866                      
---
Signif. codes:  0 ‘***’ 0.001 ‘**’ 0.01 ‘*’ 0.05 ‘.’ 0.1 ‘ ’ 1
```


Compare models


```
anova(m0, m1)
```


```
Analysis of Variance Table

Model 1: Acc ~ Stim * Task
Model 2: Acc ~ Stim * Task * Block
  Res.Df    RSS Df Sum of Sq      F  Pr(>F)  
1   2486 96.465                              
2   2476 95.726 10   0.73875 1.9108 0.03944 *
---
Signif. codes:  0 ‘***’ 0.001 ‘**’ 0.01 ‘*’ 0.05 ‘.’ 0.1 ‘ ’ 1
```


Linear model, random intercept and random slope Random Intercept per
subject


```
m2 <- lmer(formula = Acc ~ Stim*Task*Block + (1 | ID), data=df_epfl)
summary(m2)
```


```
Linear mixed model fit by REML. t-tests use Satterthwaite's method ['lmerModLmerTest']
Formula: Acc ~ Stim * Task * Block + (1 | ID)
   Data: df_epfl

REML criterion at convergence: -2692.2

Scaled residuals: 
    Min      1Q  Median      3Q     Max 
-5.6323 -0.5511  0.1311  0.6960  3.4043 

Random effects:
 Groups   Name        Variance Std.Dev.
 ID       (Intercept) 0.02077  0.1441  
 Residual             0.01798  0.1341  
Number of obs: 2496, groups:  ID, 20

Fixed effects:
                                Estimate Std. Error         df t value Pr(>|t|)    
(Intercept)                    7.479e-01  3.674e-02  3.165e+01  20.356  < 2e-16 ***
StimGPre-TPar                  1.154e-03  2.495e-02  2.457e+03   0.046  0.96311    
StimTPre-GPar                  1.481e-02  2.495e-02  2.457e+03   0.594  0.55286    
StimTpar-GBst                  4.595e-02  2.566e-02  2.457e+03   1.791  0.07340 .  
StimTpre-GBst                  5.386e-02  2.566e-02  2.457e+03   2.099  0.03589 *  
Task3back                     -2.692e-03  2.495e-02  2.457e+03  -0.108  0.91407    
Block                          7.115e-03  2.223e-03  2.457e+03   3.202  0.00138 ** 
StimGPre-TPar:Task3back       -2.308e-03  3.528e-02  2.457e+03  -0.065  0.94785    
StimTPre-GPar:Task3back       -2.135e-02  3.528e-02  2.457e+03  -0.605  0.54521    
StimTpar-GBst:Task3back       -3.342e-02  3.625e-02  2.457e+03  -0.922  0.35664    
StimTpre-GBst:Task3back       -3.470e-02  3.625e-02  2.457e+03  -0.957  0.33849    
StimGPre-TPar:Block            9.890e-04  3.143e-03  2.457e+03   0.315  0.75304    
StimTPre-GPar:Block            7.418e-04  3.143e-03  2.457e+03   0.236  0.81345    
StimTpar-GBst:Block           -3.697e-03  3.229e-03  2.457e+03  -1.145  0.25242    
StimTpre-GBst:Block           -5.070e-03  3.229e-03  2.457e+03  -1.570  0.11651    
Task3back:Block               -6.044e-03  3.143e-03  2.457e+03  -1.923  0.05460 .  
StimGPre-TPar:Task3back:Block -1.319e-03  4.445e-03  2.457e+03  -0.297  0.76674    
StimTPre-GPar:Task3back:Block -1.236e-03  4.445e-03  2.457e+03  -0.278  0.78094    
StimTpar-GBst:Task3back:Block  4.914e-03  4.567e-03  2.457e+03   1.076  0.28196    
StimTpre-GBst:Task3back:Block  1.923e-03  4.567e-03  2.457e+03   0.421  0.67371    
---
Signif. codes:  0 ‘***’ 0.001 ‘**’ 0.01 ‘*’ 0.05 ‘.’ 0.1 ‘ ’ 1
```


```
Correlation matrix not shown by default, as p = 20 > 12.
Use print(x, correlation=TRUE)  or
    vcov(x)        if you need it
```


```
anova(m2)
```


```
Type III Analysis of Variance Table with Satterthwaite's method
                 Sum Sq Mean Sq NumDF  DenDF F value    Pr(>F)    
Stim            0.13186 0.03296     4 2457.1  1.8335  0.119637    
Task            0.06126 0.06126     1 2457.0  3.4074  0.065024 .  
Block           0.33805 0.33805     1 2457.0 18.8025 1.508e-05 ***
Stim:Task       0.03022 0.00755     4 2457.0  0.4202  0.794197    
Stim:Block      0.09274 0.02319     4 2457.0  1.2896  0.271764    
Task:Block      0.23445 0.23445     1 2457.0 13.0404  0.000311 ***
Stim:Task:Block 0.04666 0.01166     4 2457.0  0.6487  0.627773    
---
Signif. codes:  0 ‘***’ 0.001 ‘**’ 0.01 ‘*’ 0.05 ‘.’ 0.1 ‘ ’ 1
```


#### Compare models

When comparing a linear model (lm) to a linear mixed-effect model
(lme), the regular ANOVA does not work, so I will only look at the
information criteria


```
AIC(m1, m2)
```


```
BIC(m1, m2)
```

### Note:

The model including random intercepts is much better than the regular
linear model. We will see whether including a random slope improves the
model.

#### Random intercept and random slope per subject


```
m3 <- lmer(formula = Acc ~ Stim*Task*Block + (1 + Block|ID), data=df_epfl)
```


```
Warning: Model failed to converge with max|grad| = 0.0129742 (tol = 0.002, component 1)
```


```
summary(m3)
```


```
Linear mixed model fit by REML. t-tests use Satterthwaite's method ['lmerModLmerTest']
Formula: Acc ~ Stim * Task * Block + (1 + Block | ID)
   Data: df_epfl

REML criterion at convergence: -2697.2

Scaled residuals: 
    Min      1Q  Median      3Q     Max 
-5.5488 -0.5567  0.1361  0.6976  3.2954 

Random effects:
 Groups   Name        Variance  Std.Dev. Corr 
 ID       (Intercept) 2.400e-02 0.154910      
          Block       5.413e-06 0.002327 -0.69
 Residual             1.790e-02 0.133809      
Number of obs: 2496, groups:  ID, 20

Fixed effects:
                                Estimate Std. Error         df t value Pr(>|t|)    
(Intercept)                    7.479e-01  3.886e-02  2.860e+01  19.248  < 2e-16 ***
StimGPre-TPar                  1.154e-03  2.490e-02  2.439e+03   0.046  0.96304    
StimTPre-GPar                  1.481e-02  2.490e-02  2.439e+03   0.595  0.55204    
StimTpar-GBst                  4.604e-02  2.562e-02  2.452e+03   1.797  0.07247 .  
StimTpre-GBst                  5.394e-02  2.562e-02  2.452e+03   2.106  0.03535 *  
Task3back                     -2.692e-03  2.490e-02  2.439e+03  -0.108  0.91389    
Block                          7.115e-03  2.278e-03  6.813e+02   3.123  0.00186 ** 
StimGPre-TPar:Task3back       -2.308e-03  3.521e-02  2.439e+03  -0.066  0.94774    
StimTPre-GPar:Task3back       -2.135e-02  3.521e-02  2.439e+03  -0.606  0.54437    
StimTpar-GBst:Task3back       -3.342e-02  3.617e-02  2.439e+03  -0.924  0.35564    
StimTpre-GBst:Task3back       -3.470e-02  3.617e-02  2.439e+03  -0.959  0.33749    
StimGPre-TPar:Block            9.890e-04  3.136e-03  2.439e+03   0.315  0.75255    
StimTPre-GPar:Block            7.418e-04  3.136e-03  2.439e+03   0.236  0.81307    
StimTpar-GBst:Block           -3.710e-03  3.225e-03  2.455e+03  -1.151  0.25005    
StimTpre-GBst:Block           -5.084e-03  3.225e-03  2.455e+03  -1.576  0.11505    
Task3back:Block               -6.044e-03  3.136e-03  2.439e+03  -1.927  0.05410 .  
StimGPre-TPar:Task3back:Block -1.319e-03  4.436e-03  2.439e+03  -0.297  0.76627    
StimTPre-GPar:Task3back:Block -1.236e-03  4.436e-03  2.439e+03  -0.279  0.78049    
StimTpar-GBst:Task3back:Block  4.914e-03  4.557e-03  2.439e+03   1.078  0.28096    
StimTpre-GBst:Task3back:Block  1.923e-03  4.557e-03  2.439e+03   0.422  0.67308    
---
Signif. codes:  0 ‘***’ 0.001 ‘**’ 0.01 ‘*’ 0.05 ‘.’ 0.1 ‘ ’ 1
```


```
Correlation matrix not shown by default, as p = 20 > 12.
Use print(x, correlation=TRUE)  or
    vcov(x)        if you need it
```


```
optimizer (nloptwrap) convergence code: 0 (OK)
Model failed to converge with max|grad| = 0.0129742 (tol = 0.002, component 1)
```


```
anova(m3)
```


```
Type III Analysis of Variance Table with Satterthwaite's method
                  Sum Sq  Mean Sq NumDF  DenDF F value    Pr(>F)    
Stim            0.131725 0.032931     4 2442.8  1.8392 0.1185602    
Task            0.061263 0.061263     1 2438.6  3.4216 0.0644704 .  
Block           0.219956 0.219956     1   19.3 12.2847 0.0023255 ** 
Stim:Task       0.030218 0.007554     4 2438.6  0.4219 0.7929311    
Stim:Block      0.092919 0.023230     4 2436.3  1.2974 0.2687205    
Task:Block      0.234455 0.234455     1 2438.6 13.0945 0.0003022 ***
Stim:Task:Block 0.046655 0.011664     4 2438.6  0.6514 0.6258660    
---
Signif. codes:  0 ‘***’ 0.001 ‘**’ 0.01 ‘*’ 0.05 ‘.’ 0.1 ‘ ’ 1
```

#### Compare models, even though this last one did not converge


```
anova(m2, m3)
```


```
refitting model(s) with ML (instead of REML)
```


```
Data: df_epfl
Models:
m2: Acc ~ Stim * Task * Block + (1 | ID)
m3: Acc ~ Stim * Task * Block + (1 + Block | ID)
   npar     AIC     BIC logLik deviance  Chisq Df Pr(>Chisq)  
m2   22 -2823.7 -2695.6 1433.8  -2867.7                       
m3   24 -2824.5 -2684.7 1436.2  -2872.5 4.7851  2     0.0914 .
---
Signif. codes:  0 ‘***’ 0.001 ‘**’ 0.01 ‘*’ 0.05 ‘.’ 0.1 ‘ ’ 1
```


The addition of a random slope results in a model that does not
converge. In addition, this model does not significantly improve the
previous one, so we will not include a random slope.

Model with random intercept per participant and per day, trying to
account for differences between session days


```
m4 <- lmer(formula = Acc ~ Stim*Task*Block + (1 + (1|ID) + (1|Day)), data=df_epfl)
summary(m4)
```


```
Linear mixed model fit by REML. t-tests use Satterthwaite's method ['lmerModLmerTest']
Formula: Acc ~ Stim * Task * Block + (1 + (1 | ID) + (1 | Day))
   Data: df_epfl

REML criterion at convergence: -2712.8

Scaled residuals: 
    Min      1Q  Median      3Q     Max 
-5.5850 -0.5841  0.1276  0.6956  3.5648 

Random effects:
 Groups   Name        Variance  Std.Dev.
 ID       (Intercept) 0.0207735 0.14413 
 Day      (Intercept) 0.0003244 0.01801 
 Residual             0.0177794 0.13334 
Number of obs: 2496, groups:  ID, 20; Day, 5

Fixed effects:
                                Estimate Std. Error         df t value Pr(>|t|)    
(Intercept)                    7.487e-01  3.814e-02  3.394e+01  19.630   <2e-16 ***
StimGPre-TPar                 -6.543e-04  2.481e-02  2.454e+03  -0.026   0.9790    
StimTPre-GPar                  1.404e-02  2.487e-02  2.456e+03   0.565   0.5724    
StimTpar-GBst                  4.509e-02  3.036e-02  2.821e+01   1.485   0.1485    
StimTpre-GBst                  5.300e-02  3.036e-02  2.821e+01   1.746   0.0917 .  
Task3back                     -2.692e-03  2.481e-02  2.454e+03  -0.109   0.9136    
Block                          7.115e-03  2.210e-03  2.454e+03   3.220   0.0013 ** 
StimGPre-TPar:Task3back       -2.308e-03  3.508e-02  2.454e+03  -0.066   0.9476    
StimTPre-GPar:Task3back       -2.135e-02  3.508e-02  2.454e+03  -0.608   0.5430    
StimTpar-GBst:Task3back       -3.342e-02  3.605e-02  2.454e+03  -0.927   0.3539    
StimTpre-GBst:Task3back       -3.470e-02  3.605e-02  2.454e+03  -0.963   0.3358    
StimGPre-TPar:Block            9.890e-04  3.125e-03  2.454e+03   0.316   0.7517    
StimTPre-GPar:Block            7.418e-04  3.125e-03  2.454e+03   0.237   0.8124    
StimTpar-GBst:Block           -3.697e-03  3.211e-03  2.454e+03  -1.151   0.2498    
StimTpre-GBst:Block           -5.070e-03  3.211e-03  2.454e+03  -1.579   0.1145    
Task3back:Block               -6.044e-03  3.125e-03  2.454e+03  -1.934   0.0533 .  
StimGPre-TPar:Task3back:Block -1.319e-03  4.420e-03  2.454e+03  -0.298   0.7655    
StimTPre-GPar:Task3back:Block -1.236e-03  4.420e-03  2.454e+03  -0.280   0.7797    
StimTpar-GBst:Task3back:Block  4.914e-03  4.541e-03  2.454e+03   1.082   0.2793    
StimTpre-GBst:Task3back:Block  1.923e-03  4.541e-03  2.454e+03   0.423   0.6720    
---
Signif. codes:  0 ‘***’ 0.001 ‘**’ 0.01 ‘*’ 0.05 ‘.’ 0.1 ‘ ’ 1
```


```
Correlation matrix not shown by default, as p = 20 > 12.
Use print(x, correlation=TRUE)  or
    vcov(x)        if you need it
```


```
anova(m4)
```


```
Type III Analysis of Variance Table with Satterthwaite's method
                 Sum Sq Mean Sq NumDF  DenDF F value    Pr(>F)    
Stim            0.04751 0.01188     4   40.2  0.6681 0.6178940    
Task            0.06126 0.06126     1 2454.0  3.4457 0.0635357 .  
Block           0.33805 0.33805     1 2454.0 19.0137 1.351e-05 ***
Stim:Task       0.03022 0.00755     4 2454.0  0.4249 0.7907701    
Stim:Block      0.09274 0.02319     4 2454.0  1.3041 0.2661499    
Task:Block      0.23445 0.23445     1 2454.0 13.1869 0.0002877 ***
Stim:Task:Block 0.04666 0.01166     4 2454.0  0.6560 0.6226167    
---
Signif. codes:  0 ‘***’ 0.001 ‘**’ 0.01 ‘*’ 0.05 ‘.’ 0.1 ‘ ’ 1
```

#### Compare models


```
anova(m2, m4)
```


```
refitting model(s) with ML (instead of REML)
```


```
Data: df_epfl
Models:
m2: Acc ~ Stim * Task * Block + (1 | ID)
m4: Acc ~ Stim * Task * Block + (1 + (1 | ID) + (1 | Day))
   npar     AIC     BIC logLik deviance  Chisq Df Pr(>Chisq)    
m2   22 -2823.7 -2695.6 1433.8  -2867.7                         
m4   23 -2840.2 -2706.3 1443.1  -2886.2 18.552  1  1.654e-05 ***
---
Signif. codes:  0 ‘***’ 0.001 ‘**’ 0.01 ‘*’ 0.05 ‘.’ 0.1 ‘ ’ 1
```

### Model choice

Adding a random intercept for the training day significantly improves
the model, so we will use this model.

### 


```
m_acc <- lmer(formula = Acc ~ Stim*Task*Block + (1 + (1|ID) + (1|Day)), data=df_epfl)
summary(m_acc)
```


```
Linear mixed model fit by REML. t-tests use Satterthwaite's method ['lmerModLmerTest']
Formula: Acc ~ Stim * Task * Block + (1 + (1 | ID) + (1 | Day))
   Data: df_epfl

REML criterion at convergence: -2712.8

Scaled residuals: 
    Min      1Q  Median      3Q     Max 
-5.5850 -0.5841  0.1276  0.6956  3.5648 

Random effects:
 Groups   Name        Variance  Std.Dev.
 ID       (Intercept) 0.0207735 0.14413 
 Day      (Intercept) 0.0003244 0.01801 
 Residual             0.0177794 0.13334 
Number of obs: 2496, groups:  ID, 20; Day, 5

Fixed effects:
                                Estimate Std. Error         df t value Pr(>|t|)    
(Intercept)                    7.487e-01  3.814e-02  3.394e+01  19.630   <2e-16 ***
StimGPre-TPar                 -6.543e-04  2.481e-02  2.454e+03  -0.026   0.9790    
StimTPre-GPar                  1.404e-02  2.487e-02  2.456e+03   0.565   0.5724    
StimTpar-GBst                  4.509e-02  3.036e-02  2.821e+01   1.485   0.1485    
StimTpre-GBst                  5.300e-02  3.036e-02  2.821e+01   1.746   0.0917 .  
Task3back                     -2.692e-03  2.481e-02  2.454e+03  -0.109   0.9136    
Block                          7.115e-03  2.210e-03  2.454e+03   3.220   0.0013 ** 
StimGPre-TPar:Task3back       -2.308e-03  3.508e-02  2.454e+03  -0.066   0.9476    
StimTPre-GPar:Task3back       -2.135e-02  3.508e-02  2.454e+03  -0.608   0.5430    
StimTpar-GBst:Task3back       -3.342e-02  3.605e-02  2.454e+03  -0.927   0.3539    
StimTpre-GBst:Task3back       -3.470e-02  3.605e-02  2.454e+03  -0.963   0.3358    
StimGPre-TPar:Block            9.890e-04  3.125e-03  2.454e+03   0.316   0.7517    
StimTPre-GPar:Block            7.418e-04  3.125e-03  2.454e+03   0.237   0.8124    
StimTpar-GBst:Block           -3.697e-03  3.211e-03  2.454e+03  -1.151   0.2498    
StimTpre-GBst:Block           -5.070e-03  3.211e-03  2.454e+03  -1.579   0.1145    
Task3back:Block               -6.044e-03  3.125e-03  2.454e+03  -1.934   0.0533 .  
StimGPre-TPar:Task3back:Block -1.319e-03  4.420e-03  2.454e+03  -0.298   0.7655    
StimTPre-GPar:Task3back:Block -1.236e-03  4.420e-03  2.454e+03  -0.280   0.7797    
StimTpar-GBst:Task3back:Block  4.914e-03  4.541e-03  2.454e+03   1.082   0.2793    
StimTpre-GBst:Task3back:Block  1.923e-03  4.541e-03  2.454e+03   0.423   0.6720    
---
Signif. codes:  0 ‘***’ 0.001 ‘**’ 0.01 ‘*’ 0.05 ‘.’ 0.1 ‘ ’ 1
```


```
Correlation matrix not shown by default, as p = 20 > 12.
Use print(x, correlation=TRUE)  or
    vcov(x)        if you need it
```


```
anova(m_acc)
```


```
Type III Analysis of Variance Table with Satterthwaite's method
                 Sum Sq Mean Sq NumDF  DenDF F value    Pr(>F)    
Stim            0.04751 0.01188     4   40.2  0.6681 0.6178940    
Task            0.06126 0.06126     1 2454.0  3.4457 0.0635357 .  
Block           0.33805 0.33805     1 2454.0 19.0137 1.351e-05 ***
Stim:Task       0.03022 0.00755     4 2454.0  0.4249 0.7907701    
Stim:Block      0.09274 0.02319     4 2454.0  1.3041 0.2661499    
Task:Block      0.23445 0.23445     1 2454.0 13.1869 0.0002877 ***
Stim:Task:Block 0.04666 0.01166     4 2454.0  0.6560 0.6226167    
---
Signif. codes:  0 ‘***’ 0.001 ‘**’ 0.01 ‘*’ 0.05 ‘.’ 0.1 ‘ ’ 1
```


```
eta_squared(m_acc)
```


```
# Effect Size for ANOVA (Type III)

Parameter       | Eta2 (partial) |       95% CI
-----------------------------------------------
Stim            |           0.06 | [0.00, 1.00]
Task            |       1.40e-03 | [0.00, 1.00]
Block           |       7.69e-03 | [0.00, 1.00]
Stim:Task       |       6.92e-04 | [0.00, 1.00]
Stim:Block      |       2.12e-03 | [0.00, 1.00]
Task:Block      |       5.34e-03 | [0.00, 1.00]
Stim:Task:Block |       1.07e-03 | [0.00, 1.00]

- One-sided CIs: upper bound fixed at [1.00].
```


There is a significant interaction of task and blocks suggesting
different rates in performance change on each task. Based on this, and
on the expectation for different performance in the 2-back compared to
the 3-back task resulting from the different difficulty levels in both
tasks, we will compare the accuracy among stimulation conditions for
each task separately.

### Accuracy in the 2-back task


```
data_subset <- subset(df_epfl, Task == '2back')
data_subset <- droplevels(data_subset)
levels(data_subset$Task)
```


```
[1] "2back"
```


```
m_acc_2back <- lmer(formula = Acc ~ Stim*Block + (1 + (1|ID) + (1|Day)), data=data_subset)
summary(m_acc_2back)
```


```
Linear mixed model fit by REML. t-tests use Satterthwaite's method ['lmerModLmerTest']
Formula: Acc ~ Stim * Block + (1 + (1 | ID) + (1 | Day))
   Data: data_subset

REML criterion at convergence: -1442

Scaled residuals: 
    Min      1Q  Median      3Q     Max 
-5.4036 -0.5353  0.1395  0.6105  2.7975 

Random effects:
 Groups   Name        Variance  Std.Dev.
 ID       (Intercept) 0.0247484 0.15732 
 Day      (Intercept) 0.0005201 0.02281 
 Residual             0.0160308 0.12661 
Number of obs: 1248, groups:  ID, 20; Day, 5

Fixed effects:
                      Estimate Std. Error         df t value Pr(>|t|)    
(Intercept)          7.497e-01  4.110e-02  3.006e+01  18.243  < 2e-16 ***
StimGPre-TPar       -1.068e-03  2.356e-02  1.216e+03  -0.045  0.96385    
StimTPre-GPar        1.155e-02  2.367e-02  1.218e+03   0.488  0.62585    
StimTpar-GBst        4.054e-02  3.197e-02  1.320e+01   1.268  0.22667    
StimTpre-GBst        4.845e-02  3.197e-02  1.320e+01   1.515  0.15324    
Block                7.115e-03  2.099e-03  1.216e+03   3.391  0.00072 ***
StimGPre-TPar:Block  9.890e-04  2.968e-03  1.216e+03   0.333  0.73901    
StimTPre-GPar:Block  7.418e-04  2.968e-03  1.216e+03   0.250  0.80268    
StimTpar-GBst:Block -3.697e-03  3.049e-03  1.216e+03  -1.212  0.22563    
StimTpre-GBst:Block -5.070e-03  3.049e-03  1.216e+03  -1.663  0.09661 .  
---
Signif. codes:  0 ‘***’ 0.001 ‘**’ 0.01 ‘*’ 0.05 ‘.’ 0.1 ‘ ’ 1

Correlation of Fixed Effects:
                   (Intr) StGP-TP StTP-GP StimTpar-GBst StimTpre-GBst Block  SGP-TP: STP-GP: StimTpar-GBst:Blck
StimGPr-TPr        -0.287                                                                                      
StimTPr-GPr        -0.287  0.498                                                                               
StimTpar-GBst      -0.344  0.369   0.369                                                                       
StimTpre-GBst      -0.344  0.369   0.369   0.698                                                               
Block              -0.357  0.623   0.621   0.459         0.459                                                 
StmGPr-TP:B         0.253 -0.882  -0.439  -0.325        -0.325        -0.707                                   
StmTPr-GP:B         0.253 -0.441  -0.878  -0.325        -0.325        -0.707  0.500                            
StimTpar-GBst:Blck  0.246 -0.429  -0.427  -0.668        -0.316        -0.688  0.487   0.487                    
StimTpre-GBst:Blck  0.246 -0.429  -0.427  -0.316        -0.668        -0.688  0.487   0.487   0.474
```


```
anova(m_acc_2back)
```


```
Type III Analysis of Variance Table with Satterthwaite's method
            Sum Sq Mean Sq NumDF  DenDF F value    Pr(>F)    
Stim       0.04671 0.01168     4   43.3  0.7284    0.5775    
Block      0.56778 0.56778     1 1216.0 35.4181 3.475e-09 ***
Stim:Block 0.10578 0.02644     4 1216.0  1.6496    0.1595    
---
Signif. codes:  0 ‘***’ 0.001 ‘**’ 0.01 ‘*’ 0.05 ‘.’ 0.1 ‘ ’ 1
```


```
eta_squared(m_acc_2back)
```


```
# Effect Size for ANOVA (Type III)

Parameter  | Eta2 (partial) |       95% CI
------------------------------------------
Stim       |           0.06 | [0.00, 1.00]
Block      |           0.03 | [0.01, 1.00]
Stim:Block |       5.40e-03 | [0.00, 1.00]

- One-sided CIs: upper bound fixed at [1.00].
```


There is no significant effect of stimulation, nor a significant
difference in the rate of performance change across conditions. There
was only a significant effect of block.

### Accuracy in the 3-back task


```
data_subset <- subset(df_epfl, Task == '3back')
data_subset <- droplevels(data_subset)
levels(data_subset$Task)
```


```
[1] "3back"
```


```
m_acc_3back <- lmer(formula = Acc ~ Stim*Block + (1 + (1|ID) + (1|Day)), data=data_subset)
summary(m_acc_3back)
```


```
Linear mixed model fit by REML. t-tests use Satterthwaite's method ['lmerModLmerTest']
Formula: Acc ~ Stim * Block + (1 + (1 | ID) + (1 | Day))
   Data: data_subset

REML criterion at convergence: -1338.6

Scaled residuals: 
    Min      1Q  Median      3Q     Max 
-3.7192 -0.6302  0.1156  0.7268  3.0706 

Random effects:
 Groups   Name        Variance  Std.Dev.
 ID       (Intercept) 0.0189834 0.13778 
 Day      (Intercept) 0.0001598 0.01264 
 Residual             0.0175639 0.13253 
Number of obs: 1248, groups:  ID, 20; Day, 5

Fixed effects:
                      Estimate Std. Error         df t value Pr(>|t|)    
(Intercept)          7.451e-01  3.615e-02  3.361e+01  20.610   <2e-16 ***
StimGPre-TPar       -2.216e-03  2.466e-02  1.216e+03  -0.090    0.928    
StimTPre-GPar       -5.188e-03  2.475e-02  1.219e+03  -0.210    0.834    
StimTpar-GBst        1.614e-02  2.789e-02  4.839e+01   0.579    0.566    
StimTpre-GBst        2.276e-02  2.789e-02  4.839e+01   0.816    0.418    
Block                1.071e-03  2.197e-03  1.216e+03   0.488    0.626    
StimGPre-TPar:Block -3.297e-04  3.106e-03  1.216e+03  -0.106    0.916    
StimTPre-GPar:Block -4.945e-04  3.106e-03  1.216e+03  -0.159    0.874    
StimTpar-GBst:Block  1.218e-03  3.192e-03  1.216e+03   0.382    0.703    
StimTpre-GBst:Block -3.147e-03  3.192e-03  1.216e+03  -0.986    0.324    
---
Signif. codes:  0 ‘***’ 0.001 ‘**’ 0.01 ‘*’ 0.05 ‘.’ 0.1 ‘ ’ 1

Correlation of Fixed Effects:
                   (Intr) StGP-TP StTP-GP StimTpar-GBst StimTpre-GBst Block  SGP-TP: STP-GP: StimTpar-GBst:Blck
StimGPr-TPr        -0.341                                                                                      
StimTPr-GPr        -0.342  0.498                                                                               
StimTpar-GBst      -0.355  0.442   0.443                                                                       
StimTpre-GBst      -0.355  0.442   0.443   0.566                                                               
Block              -0.425  0.623   0.621   0.551         0.551                                                 
StmGPr-TP:B         0.301 -0.882  -0.439  -0.390        -0.390        -0.707                                   
StmTPr-GP:B         0.301 -0.441  -0.879  -0.390        -0.390        -0.707  0.500                            
StimTpar-GBst:Blck  0.293 -0.429  -0.428  -0.801        -0.379        -0.688  0.487   0.487                    
StimTpre-GBst:Blck  0.293 -0.429  -0.428  -0.379        -0.801        -0.688  0.487   0.487   0.474
```


```
anova(m_acc_3back)
```


```
Type III Analysis of Variance Table with Satterthwaite's method
             Sum Sq   Mean Sq NumDF   DenDF F value Pr(>F)
Stim       0.022705 0.0056762     4   83.72  0.3232 0.8617
Block      0.004726 0.0047256     1 1216.07  0.2691 0.6041
Stim:Block 0.033621 0.0084052     4 1216.07  0.4785 0.7515
```


```
eta_squared(m_acc_3back)
```


```
# Effect Size for ANOVA (Type III)

Parameter  | Eta2 (partial) |       95% CI
------------------------------------------
Stim       |           0.02 | [0.00, 1.00]
Block      |       2.21e-04 | [0.00, 1.00]
Stim:Block |       1.57e-03 | [0.00, 1.00]

- One-sided CIs: upper bound fixed at [1.00].
```

### Note:

There is no evidence for significant differences in the 3-back
task.

## Normalized accuracy

Human behavior tends to be highly variable across subjects, which
sometimes makes a direct comparison of performance challenging. For this
reason, we will now assess the change in accuracy experienced by each
participant, which we intend to use as a correction for native
differences present at the start of each session. This correction was
done by dividing the accuracy score of each block by that of the first
training block of each session. Please note this correction was done
within each session (i.e., using the first training block of each
session), as opposed to using the first block of training ever performed
by each participant.

### Group averages


```
m0 <- lm(formula = Acc_norm ~ Stim*Task, data=df_epfl)
summary(m0)
```


```
Call:
lm(formula = Acc_norm ~ Stim * Task, data = df_epfl)

Residuals:
    Min      1Q  Median      3Q     Max 
-1.1164 -0.1946 -0.0710  0.1336  3.2793 

Coefficients:
                        Estimate Std. Error t value Pr(>|t|)    
(Intercept)              1.17234    0.02373  49.394  < 2e-16 ***
StimGPre-TPar            0.04834    0.03357   1.440 0.149940    
StimTPre-GPar            0.01960    0.03357   0.584 0.559350    
StimTpar-GBst           -0.20137    0.03449  -5.839 5.93e-09 ***
StimTpre-GBst           -0.03486    0.03449  -1.011 0.312132    
Task3back                0.02228    0.03357   0.664 0.506894    
StimGPre-TPar:Task3back -0.14791    0.04747  -3.116 0.001854 ** 
StimTPre-GPar:Task3back -0.09781    0.04747  -2.060 0.039461 *  
StimTpar-GBst:Task3back  0.03562    0.04877   0.730 0.465268    
StimTpre-GBst:Task3back -0.17049    0.04877  -3.496 0.000481 ***
---
Signif. codes:  0 ‘***’ 0.001 ‘**’ 0.01 ‘*’ 0.05 ‘.’ 0.1 ‘ ’ 1

Residual standard error: 0.3827 on 2486 degrees of freedom
  (104 observations deleted due to missingness)
Multiple R-squared:  0.04575,   Adjusted R-squared:  0.0423 
F-statistic: 13.24 on 9 and 2486 DF,  p-value: < 2.2e-16
```


```
anova(m0)
```


```
Analysis of Variance Table

Response: Acc_norm
            Df Sum Sq Mean Sq F value    Pr(>F)    
Stim         4  11.64 2.90928 19.8632 4.107e-16 ***
Task         1   1.83 1.83313 12.5158 0.0004109 ***
Stim:Task    4   3.99 0.99679  6.8056 1.910e-05 ***
Residuals 2486 364.11 0.14647                      
---
Signif. codes:  0 ‘***’ 0.001 ‘**’ 0.01 ‘*’ 0.05 ‘.’ 0.1 ‘ ’ 1
```

### Note:

We will now test a model including a time component (i.e., the
training blocks).


```
m1 <- lm(formula = Acc_norm ~ Stim*Task*Block, data=df_epfl)
summary(m1)
```


```
Call:
lm(formula = Acc_norm ~ Stim * Task * Block, data = df_epfl)

Residuals:
    Min      1Q  Median      3Q     Max 
-1.1164 -0.2010 -0.0776  0.1302  3.1938 

Coefficients:
                               Estimate Std. Error t value Pr(>|t|)    
(Intercept)                    1.066203   0.050185  21.245   <2e-16 ***
StimGPre-TPar                  0.004735   0.070973   0.067   0.9468    
StimTPre-GPar                  0.007409   0.070973   0.104   0.9169    
StimTpar-GBst                 -0.100076   0.072917  -1.372   0.1700    
StimTpre-GBst                  0.040397   0.072917   0.554   0.5796    
Task3back                      0.084729   0.070973   1.194   0.2327    
Block                          0.015162   0.006323   2.398   0.0166 *  
StimGPre-TPar:Task3back       -0.071635   0.100371  -0.714   0.4755    
StimTPre-GPar:Task3back       -0.050858   0.100371  -0.507   0.6124    
StimTpar-GBst:Task3back       -0.048116   0.103121  -0.467   0.6408    
StimTpre-GBst:Task3back       -0.193985   0.103121  -1.881   0.0601 .  
StimGPre-TPar:Block            0.006229   0.008942   0.697   0.4861    
StimTPre-GPar:Block            0.001741   0.008942   0.195   0.8456    
StimTpar-GBst:Block           -0.014470   0.009187  -1.575   0.1154    
StimTpre-GBst:Block           -0.010751   0.009187  -1.170   0.2420    
Task3back:Block               -0.008921   0.008942  -0.998   0.3185    
StimGPre-TPar:Task3back:Block -0.010897   0.012645  -0.862   0.3889    
StimTPre-GPar:Task3back:Block -0.006707   0.012645  -0.530   0.5959    
StimTpar-GBst:Task3back:Block  0.011962   0.012992   0.921   0.3573    
StimTpre-GBst:Task3back:Block  0.003356   0.012992   0.258   0.7962    
---
Signif. codes:  0 ‘***’ 0.001 ‘**’ 0.01 ‘*’ 0.05 ‘.’ 0.1 ‘ ’ 1

Residual standard error: 0.3815 on 2476 degrees of freedom
  (104 observations deleted due to missingness)
Multiple R-squared:  0.05575,   Adjusted R-squared:  0.0485 
F-statistic: 7.694 on 19 and 2476 DF,  p-value: < 2.2e-16
```


```
anova(m1)
```


```
Analysis of Variance Table

Response: Acc_norm
                  Df Sum Sq Mean Sq F value    Pr(>F)    
Stim               4  11.64 2.90928 19.9927 3.223e-16 ***
Task               1   1.83 1.83313 12.5974 0.0003935 ***
Block              1   1.83 1.82938 12.5716 0.0003989 ***
Stim:Task          4   3.99 0.99679  6.8500 1.761e-05 ***
Stim:Block         4   0.62 0.15378  1.0568 0.3763909    
Task:Block         1   0.82 0.82480  5.6680 0.0173519 *  
Stim:Task:Block    4   0.55 0.13632  0.9368 0.4414845    
Residuals       2476 360.30 0.14552                      
---
Signif. codes:  0 ‘***’ 0.001 ‘**’ 0.01 ‘*’ 0.05 ‘.’ 0.1 ‘ ’ 1
```


#### Compare the models


```
anova(m0, m1)
```


```
Analysis of Variance Table

Model 1: Acc_norm ~ Stim * Task
Model 2: Acc_norm ~ Stim * Task * Block
  Res.Df    RSS Df Sum of Sq      F   Pr(>F)   
1   2486 364.11                                
2   2476 360.30 10    3.8146 2.6214 0.003584 **
---
Signif. codes:  0 ‘***’ 0.001 ‘**’ 0.01 ‘*’ 0.05 ‘.’ 0.1 ‘ ’ 1
```


The addition of the blocks significantly improves the model. We will
test the inclusion of random effects next.

Include random effects Random intercept per subject


```
m2 <- lmer(formula = Acc_norm ~ Stim*Task*Block + (1 | ID), data=df_epfl)
summary(m2)
```


```
Linear mixed model fit by REML. t-tests use Satterthwaite's method ['lmerModLmerTest']
Formula: Acc_norm ~ Stim * Task * Block + (1 | ID)
   Data: df_epfl

REML criterion at convergence: 2292.4

Scaled residuals: 
    Min      1Q  Median      3Q     Max 
-3.1514 -0.5068 -0.0967  0.3660  8.2455 

Random effects:
 Groups   Name        Variance Std.Dev.
 ID       (Intercept) 0.008043 0.08968 
 Residual             0.137596 0.37094 
Number of obs: 2496, groups:  ID, 20

Fixed effects:
                                Estimate Std. Error         df t value Pr(>|t|)    
(Intercept)                    1.066e+00  5.276e-02  5.898e+02  20.209   <2e-16 ***
StimGPre-TPar                  4.735e-03  6.901e-02  2.457e+03   0.069   0.9453    
StimTPre-GPar                  7.409e-03  6.901e-02  2.457e+03   0.107   0.9145    
StimTpar-GBst                 -9.714e-02  7.096e-02  2.459e+03  -1.369   0.1712    
StimTpre-GBst                  4.333e-02  7.096e-02  2.459e+03   0.611   0.5415    
Task3back                      8.473e-02  6.901e-02  2.457e+03   1.228   0.2197    
Block                          1.516e-02  6.148e-03  2.457e+03   2.466   0.0137 *  
StimGPre-TPar:Task3back       -7.164e-02  9.760e-02  2.457e+03  -0.734   0.4630    
StimTPre-GPar:Task3back       -5.086e-02  9.760e-02  2.457e+03  -0.521   0.6024    
StimTpar-GBst:Task3back       -4.812e-02  1.003e-01  2.457e+03  -0.480   0.6314    
StimTpre-GBst:Task3back       -1.940e-01  1.003e-01  2.457e+03  -1.935   0.0532 .  
StimGPre-TPar:Block            6.229e-03  8.695e-03  2.457e+03   0.716   0.4738    
StimTPre-GPar:Block            1.741e-03  8.695e-03  2.457e+03   0.200   0.8413    
StimTpar-GBst:Block           -1.447e-02  8.933e-03  2.457e+03  -1.620   0.1054    
StimTpre-GBst:Block           -1.075e-02  8.933e-03  2.457e+03  -1.204   0.2289    
Task3back:Block               -8.921e-03  8.695e-03  2.457e+03  -1.026   0.3050    
StimGPre-TPar:Task3back:Block -1.090e-02  1.230e-02  2.457e+03  -0.886   0.3756    
StimTPre-GPar:Task3back:Block -6.707e-03  1.230e-02  2.457e+03  -0.545   0.5855    
StimTpar-GBst:Task3back:Block  1.196e-02  1.263e-02  2.457e+03   0.947   0.3438    
StimTpre-GBst:Task3back:Block  3.356e-03  1.263e-02  2.457e+03   0.266   0.7905    
---
Signif. codes:  0 ‘***’ 0.001 ‘**’ 0.01 ‘*’ 0.05 ‘.’ 0.1 ‘ ’ 1
```


```
Correlation matrix not shown by default, as p = 20 > 12.
Use print(x, correlation=TRUE)  or
    vcov(x)        if you need it
```


```
anova(m2)
```


```
Type III Analysis of Variance Table with Satterthwaite's method
                 Sum Sq Mean Sq NumDF  DenDF F value    Pr(>F)    
Stim            0.94501 0.23625     4 2459.0  1.7170 0.1434074    
Task            0.01929 0.01929     1 2457.2  0.1402 0.7081168    
Block           1.71886 1.71886     1 2457.2 12.4921 0.0004162 ***
Stim:Task       0.56423 0.14106     4 2457.2  1.0252 0.3927858    
Stim:Block      0.61511 0.15378     4 2457.2  1.1176 0.3463408    
Task:Block      0.76634 0.76634     1 2457.2  5.5695 0.0183538 *  
Stim:Task:Block 0.54528 0.13632     4 2457.2  0.9907 0.4112604    
---
Signif. codes:  0 ‘***’ 0.001 ‘**’ 0.01 ‘*’ 0.05 ‘.’ 0.1 ‘ ’ 1
```


```
AIC(m1, m2)
```


```
BIC(m1, m2)
```


The addition of a random intercept for subjects worsens the model. We
will test the addition of a random slope next.

### Test a model with a random slope


```
m3 <- lmer(formula = Acc_norm ~ Stim*Task*Block + (1 + Block|ID), data=df_epfl)
```


```
boundary (singular) fit: see help('isSingular')
```


```
summary(m3)
```


```
Linear mixed model fit by REML. t-tests use Satterthwaite's method ['lmerModLmerTest']
Formula: Acc_norm ~ Stim * Task * Block + (1 + Block | ID)
   Data: df_epfl

REML criterion at convergence: 2279.7

Scaled residuals: 
    Min      1Q  Median      3Q     Max 
-3.1592 -0.4927 -0.1039  0.3586  8.1344 

Random effects:
 Groups   Name        Variance  Std.Dev. Corr
 ID       (Intercept) 1.555e-03 0.039428     
          Block       5.228e-05 0.007231 1.00
 Residual             1.368e-01 0.369868     
Number of obs: 2496, groups:  ID, 20

Fixed effects:
                                Estimate Std. Error         df t value Pr(>|t|)    
(Intercept)                    1.066e+00  4.945e-02  1.302e+03  21.561   <2e-16 ***
StimGPre-TPar                  4.735e-03  6.882e-02  2.457e+03   0.069   0.9451    
StimTPre-GPar                  7.409e-03  6.882e-02  2.457e+03   0.108   0.9143    
StimTpar-GBst                 -9.894e-02  7.071e-02  2.457e+03  -1.399   0.1619    
StimTpre-GBst                  4.153e-02  7.071e-02  2.457e+03   0.587   0.5570    
Task3back                      8.473e-02  6.882e-02  2.457e+03   1.231   0.2183    
Block                          1.516e-02  6.340e-03  8.080e+02   2.391   0.0170 *  
StimGPre-TPar:Task3back       -7.164e-02  9.732e-02  2.457e+03  -0.736   0.4618    
StimTPre-GPar:Task3back       -5.086e-02  9.732e-02  2.457e+03  -0.523   0.6013    
StimTpar-GBst:Task3back       -4.812e-02  9.999e-02  2.457e+03  -0.481   0.6304    
StimTpre-GBst:Task3back       -1.940e-01  9.999e-02  2.457e+03  -1.940   0.0525 .  
StimGPre-TPar:Block            6.229e-03  8.670e-03  2.457e+03   0.719   0.4725    
StimTPre-GPar:Block            1.741e-03  8.670e-03  2.457e+03   0.201   0.8408    
StimTpar-GBst:Block           -1.426e-02  8.910e-03  2.457e+03  -1.601   0.1096    
StimTpre-GBst:Block           -1.054e-02  8.910e-03  2.457e+03  -1.183   0.2368    
Task3back:Block               -8.921e-03  8.670e-03  2.457e+03  -1.029   0.3036    
StimGPre-TPar:Task3back:Block -1.090e-02  1.226e-02  2.457e+03  -0.889   0.3742    
StimTPre-GPar:Task3back:Block -6.707e-03  1.226e-02  2.457e+03  -0.547   0.5844    
StimTpar-GBst:Task3back:Block  1.196e-02  1.260e-02  2.457e+03   0.950   0.3424    
StimTpre-GBst:Task3back:Block  3.356e-03  1.260e-02  2.457e+03   0.266   0.7899    
---
Signif. codes:  0 ‘***’ 0.001 ‘**’ 0.01 ‘*’ 0.05 ‘.’ 0.1 ‘ ’ 1
```


```
Correlation matrix not shown by default, as p = 20 > 12.
Use print(x, correlation=TRUE)  or
    vcov(x)        if you need it
```


```
optimizer (nloptwrap) convergence code: 0 (OK)
boundary (singular) fit: see help('isSingular')
```


```
anova(m3)
```


```
Type III Analysis of Variance Table with Satterthwaite's method
                 Sum Sq Mean Sq NumDF   DenDF F value   Pr(>F)   
Stim            0.98272 0.24568     4 2456.90  1.7959 0.126878   
Task            0.01929 0.01929     1 2457.22  0.1410 0.707310   
Block           1.05512 1.05512     1   29.65  7.7128 0.009411 **
Stim:Task       0.56423 0.14106     4 2457.22  1.0311 0.389659   
Stim:Block      0.58485 0.14621     4 2456.60  1.0688 0.370296   
Task:Block      0.76634 0.76634     1 2457.22  5.6018 0.018019 * 
Stim:Task:Block 0.54528 0.13632     4 2457.22  0.9965 0.408131   
---
Signif. codes:  0 ‘***’ 0.001 ‘**’ 0.01 ‘*’ 0.05 ‘.’ 0.1 ‘ ’ 1
```


```
AIC(m1, m3)
```


```
BIC(m1, m3)
```


The addition of a random slope also worsens m1. Next, we will test
the a model including a random intercept for days as well. Add random
intercept per day


```
m4 <- lmer(formula = Acc_norm ~ Stim*Task*Block + (1 + (1|ID) + (1|Day)), data=df_epfl)
summary(m4)
```


```
Linear mixed model fit by REML. t-tests use Satterthwaite's method ['lmerModLmerTest']
Formula: Acc_norm ~ Stim * Task * Block + (1 + (1 | ID) + (1 | Day))
   Data: df_epfl

REML criterion at convergence: 2279

Scaled residuals: 
    Min      1Q  Median      3Q     Max 
-3.2692 -0.5196 -0.1108  0.3641  8.2124 

Random effects:
 Groups   Name        Variance Std.Dev.
 ID       (Intercept) 0.008051 0.08972 
 Day      (Intercept) 0.001814 0.04259 
 Residual             0.136515 0.36948 
Number of obs: 2496, groups:  ID, 20; Day, 5

Fixed effects:
                                Estimate Std. Error         df t value Pr(>|t|)    
(Intercept)                    1.059e+00  5.808e-02  6.481e+01  18.236   <2e-16 ***
StimGPre-TPar                  5.037e-03  6.875e-02  2.454e+03   0.073   0.9416    
StimTPre-GPar                  2.851e-02  6.891e-02  2.457e+03   0.414   0.6791    
StimTpar-GBst                 -9.000e-02  8.069e-02  4.136e+01  -1.115   0.2711    
StimTpre-GBst                  5.047e-02  8.069e-02  4.136e+01   0.626   0.5351    
Task3back                      8.473e-02  6.874e-02  2.454e+03   1.233   0.2179    
Block                          1.516e-02  6.124e-03  2.454e+03   2.476   0.0134 *  
StimGPre-TPar:Task3back       -7.164e-02  9.722e-02  2.454e+03  -0.737   0.4613    
StimTPre-GPar:Task3back       -5.086e-02  9.722e-02  2.454e+03  -0.523   0.6009    
StimTpar-GBst:Task3back       -4.812e-02  9.988e-02  2.454e+03  -0.482   0.6300    
StimTpre-GBst:Task3back       -1.940e-01  9.988e-02  2.454e+03  -1.942   0.0522 .  
StimGPre-TPar:Block            6.229e-03  8.661e-03  2.454e+03   0.719   0.4720    
StimTPre-GPar:Block            1.741e-03  8.661e-03  2.454e+03   0.201   0.8407    
StimTpar-GBst:Block           -1.447e-02  8.898e-03  2.454e+03  -1.626   0.1040    
StimTpre-GBst:Block           -1.075e-02  8.898e-03  2.454e+03  -1.208   0.2271    
Task3back:Block               -8.921e-03  8.661e-03  2.454e+03  -1.030   0.3031    
StimGPre-TPar:Task3back:Block -1.090e-02  1.225e-02  2.454e+03  -0.890   0.3737    
StimTPre-GPar:Task3back:Block -6.707e-03  1.225e-02  2.454e+03  -0.548   0.5840    
StimTpar-GBst:Task3back:Block  1.196e-02  1.258e-02  2.454e+03   0.951   0.3419    
StimTpre-GBst:Task3back:Block  3.356e-03  1.258e-02  2.454e+03   0.267   0.7897    
---
Signif. codes:  0 ‘***’ 0.001 ‘**’ 0.01 ‘*’ 0.05 ‘.’ 0.1 ‘ ’ 1
```


```
Correlation matrix not shown by default, as p = 20 > 12.
Use print(x, correlation=TRUE)  or
    vcov(x)        if you need it
```


```
anova(m4)
```


```
Type III Analysis of Variance Table with Satterthwaite's method
                 Sum Sq Mean Sq NumDF   DenDF F value    Pr(>F)    
Stim            0.58609 0.14652     4   49.27  1.0733 0.3798118    
Task            0.01929 0.01929     1 2454.23  0.1413 0.7070171    
Block           1.71886 1.71886     1 2454.23 12.5910 0.0003949 ***
Stim:Task       0.56423 0.14106     4 2454.23  1.0333 0.3885251    
Stim:Block      0.61511 0.15378     4 2454.23  1.1264 0.3421349    
Task:Block      0.76634 0.76634     1 2454.23  5.6136 0.0178988 *  
Stim:Task:Block 0.54528 0.13632     4 2454.23  0.9986 0.4069963    
---
Signif. codes:  0 ‘***’ 0.001 ‘**’ 0.01 ‘*’ 0.05 ‘.’ 0.1 ‘ ’ 1
```


```
AIC(m1, m4)
```


```
BIC(m1, m4)
```

### Model choice

In the case of the normalized accuracy, adding random effects does
not improve the model.


```
m_acc_norm <- lm(formula = Acc_norm ~ Stim*Task*Block, data=df_epfl)
summary(m_acc_norm)
```


```
Call:
lm(formula = Acc_norm ~ Stim * Task * Block, data = df_epfl)

Residuals:
    Min      1Q  Median      3Q     Max 
-1.1164 -0.2010 -0.0776  0.1302  3.1938 

Coefficients:
                               Estimate Std. Error t value Pr(>|t|)    
(Intercept)                    1.066203   0.050185  21.245   <2e-16 ***
StimGPre-TPar                  0.004735   0.070973   0.067   0.9468    
StimTPre-GPar                  0.007409   0.070973   0.104   0.9169    
StimTpar-GBst                 -0.100076   0.072917  -1.372   0.1700    
StimTpre-GBst                  0.040397   0.072917   0.554   0.5796    
Task3back                      0.084729   0.070973   1.194   0.2327    
Block                          0.015162   0.006323   2.398   0.0166 *  
StimGPre-TPar:Task3back       -0.071635   0.100371  -0.714   0.4755    
StimTPre-GPar:Task3back       -0.050858   0.100371  -0.507   0.6124    
StimTpar-GBst:Task3back       -0.048116   0.103121  -0.467   0.6408    
StimTpre-GBst:Task3back       -0.193985   0.103121  -1.881   0.0601 .  
StimGPre-TPar:Block            0.006229   0.008942   0.697   0.4861    
StimTPre-GPar:Block            0.001741   0.008942   0.195   0.8456    
StimTpar-GBst:Block           -0.014470   0.009187  -1.575   0.1154    
StimTpre-GBst:Block           -0.010751   0.009187  -1.170   0.2420    
Task3back:Block               -0.008921   0.008942  -0.998   0.3185    
StimGPre-TPar:Task3back:Block -0.010897   0.012645  -0.862   0.3889    
StimTPre-GPar:Task3back:Block -0.006707   0.012645  -0.530   0.5959    
StimTpar-GBst:Task3back:Block  0.011962   0.012992   0.921   0.3573    
StimTpre-GBst:Task3back:Block  0.003356   0.012992   0.258   0.7962    
---
Signif. codes:  0 ‘***’ 0.001 ‘**’ 0.01 ‘*’ 0.05 ‘.’ 0.1 ‘ ’ 1

Residual standard error: 0.3815 on 2476 degrees of freedom
  (104 observations deleted due to missingness)
Multiple R-squared:  0.05575,   Adjusted R-squared:  0.0485 
F-statistic: 7.694 on 19 and 2476 DF,  p-value: < 2.2e-16
```


```
anova(m_acc_norm)
```


```
Analysis of Variance Table

Response: Acc_norm
                  Df Sum Sq Mean Sq F value    Pr(>F)    
Stim               4  11.64 2.90928 19.9927 3.223e-16 ***
Task               1   1.83 1.83313 12.5974 0.0003935 ***
Block              1   1.83 1.82938 12.5716 0.0003989 ***
Stim:Task          4   3.99 0.99679  6.8500 1.761e-05 ***
Stim:Block         4   0.62 0.15378  1.0568 0.3763909    
Task:Block         1   0.82 0.82480  5.6680 0.0173519 *  
Stim:Task:Block    4   0.55 0.13632  0.9368 0.4414845    
Residuals       2476 360.30 0.14552                      
---
Signif. codes:  0 ‘***’ 0.001 ‘**’ 0.01 ‘*’ 0.05 ‘.’ 0.1 ‘ ’ 1
```


```
eta_squared(m_acc_norm)
```


```
# Effect Size for ANOVA (Type I)

Parameter       | Eta2 (partial) |       95% CI
-----------------------------------------------
Stim            |           0.03 | [0.02, 1.00]
Task            |       5.06e-03 | [0.00, 1.00]
Block           |       5.05e-03 | [0.00, 1.00]
Stim:Task       |           0.01 | [0.00, 1.00]
Stim:Block      |       1.70e-03 | [0.00, 1.00]
Task:Block      |       2.28e-03 | [0.00, 1.00]
Stim:Task:Block |       1.51e-03 | [0.00, 1.00]

- One-sided CIs: upper bound fixed at [1.00].
```

### Note:

There is an effect of stimulation, which seems to differ among the
tasks, which are also different. Following the same rationale as we
applied to the uncorrected accuracy, we will test the tasks separately
next.

### Normalized accuracy in the 2-back task


```
data_subset <- subset(df_epfl, Task == '2back')
data_subset <- droplevels(data_subset)
levels(data_subset$Task)
```


```
[1] "2back"
```


```
m_acc_norm_2back <- lm(formula = Acc_norm ~ Stim*Block, data=data_subset)
summary(m_acc_norm_2back)
```


```
Call:
lm(formula = Acc_norm ~ Stim * Block, data = data_subset)

Residuals:
    Min      1Q  Median      3Q     Max 
-0.9751 -0.2176 -0.0814  0.1248  3.1938 

Coefficients:
                     Estimate Std. Error t value Pr(>|t|)    
(Intercept)          1.066203   0.053182  20.048   <2e-16 ***
StimGPre-TPar        0.004735   0.075211   0.063   0.9498    
StimTPre-GPar        0.007409   0.075211   0.099   0.9215    
StimTpar-GBst       -0.100076   0.077272  -1.295   0.1955    
StimTpre-GBst        0.040397   0.077272   0.523   0.6012    
Block                0.015162   0.006700   2.263   0.0238 *  
StimGPre-TPar:Block  0.006229   0.009476   0.657   0.5110    
StimTPre-GPar:Block  0.001741   0.009476   0.184   0.8542    
StimTpar-GBst:Block -0.014470   0.009735  -1.486   0.1374    
StimTpre-GBst:Block -0.010751   0.009735  -1.104   0.2696    
---
Signif. codes:  0 ‘***’ 0.001 ‘**’ 0.01 ‘*’ 0.05 ‘.’ 0.1 ‘ ’ 1

Residual standard error: 0.4042 on 1238 degrees of freedom
  (52 observations deleted due to missingness)
Multiple R-squared:  0.06018,   Adjusted R-squared:  0.05335 
F-statistic: 8.808 on 9 and 1238 DF,  p-value: 6.202e-13
```


```
anova(m_acc_norm_2back)
```


```
Analysis of Variance Table

Response: Acc_norm
             Df  Sum Sq Mean Sq F value    Pr(>F)    
Stim          4   9.347 2.33663  14.299 2.046e-11 ***
Block         1   2.555 2.55544  15.638 8.105e-05 ***
Stim:Block    4   1.052 0.26310   1.610    0.1693    
Residuals  1238 202.306 0.16341                      
---
Signif. codes:  0 ‘***’ 0.001 ‘**’ 0.01 ‘*’ 0.05 ‘.’ 0.1 ‘ ’ 1
```


```
eta_squared(m_acc_norm_2back)
```


```
# Effect Size for ANOVA (Type I)

Parameter  | Eta2 (partial) |       95% CI
------------------------------------------
Stim       |           0.04 | [0.03, 1.00]
Block      |           0.01 | [0.00, 1.00]
Stim:Block |       5.18e-03 | [0.00, 1.00]

- One-sided CIs: upper bound fixed at [1.00].
```

### Note:

There is an effect of stimulation in the 2-back task. We will now do
post-hoc comparisons to see where these differences come from.


```
my_model <- m_acc_norm_2back
level_1 <- "Stim"

my_model.compare <- emmeans(my_model, level_1, by="Block", at = list(Block = c(7, 13)))
my_model.compare.pairs <- pairs(my_model.compare, adjust='tukey')
test(my_model.compare.pairs, side='two-sided')
```


```
Block =  7:
 contrast                  estimate     SE   df t.ratio p.value
 Placebo - (GPre-TPar)      -0.0483 0.0355 1238  -1.363  0.6513
 Placebo - (TPre-GPar)      -0.0196 0.0355 1238  -0.553  0.9816
 Placebo - (Tpar-GBst)       0.2014 0.0364 1238   5.528  <.0001
 Placebo - (Tpre-GBst)       0.0349 0.0364 1238   0.957  0.8742
 (GPre-TPar) - (TPre-GPar)   0.0287 0.0355 1238   0.811  0.9274
 (GPre-TPar) - (Tpar-GBst)   0.2497 0.0364 1238   6.855  <.0001
 (GPre-TPar) - (Tpre-GBst)   0.0832 0.0364 1238   2.284  0.1506
 (TPre-GPar) - (Tpar-GBst)   0.2210 0.0364 1238   6.066  <.0001
 (TPre-GPar) - (Tpre-GBst)   0.0545 0.0364 1238   1.495  0.5658
 (Tpar-GBst) - (Tpre-GBst)  -0.1665 0.0374 1238  -4.455  0.0001

Block = 13:
 contrast                  estimate     SE   df t.ratio p.value
 Placebo - (GPre-TPar)      -0.0857 0.0670 1238  -1.279  0.7041
 Placebo - (TPre-GPar)      -0.0300 0.0670 1238  -0.448  0.9916
 Placebo - (Tpar-GBst)       0.2882 0.0688 1238   4.186  0.0003
 Placebo - (Tpre-GBst)       0.0994 0.0688 1238   1.444  0.5995
 (GPre-TPar) - (TPre-GPar)   0.0557 0.0670 1238   0.831  0.9211
 (GPre-TPar) - (Tpar-GBst)   0.3739 0.0688 1238   5.432  <.0001
 (GPre-TPar) - (Tpre-GBst)   0.1851 0.0688 1238   2.689  0.0562
 (TPre-GPar) - (Tpar-GBst)   0.3182 0.0688 1238   4.623  <.0001
 (TPre-GPar) - (Tpre-GBst)   0.1294 0.0688 1238   1.880  0.3286
 (Tpar-GBst) - (Tpre-GBst)  -0.1888 0.0706 1238  -2.673  0.0585

P value adjustment: tukey method for comparing a family of 5 estimates
```


```
confint(my_model.compare, calc = c(n = ~.wgt.))
```


```
Block =  7:
 Stim      emmean     SE   df   n lower.CL upper.CL
 Placebo    1.172 0.0251 1238 260    1.123     1.22
 GPre-TPar  1.221 0.0251 1238 260    1.171     1.27
 TPre-GPar  1.192 0.0251 1238 260    1.143     1.24
 Tpar-GBst  0.971 0.0264 1238 234    0.919     1.02
 Tpre-GBst  1.137 0.0264 1238 234    1.086     1.19

Block = 13:
 Stim      emmean     SE   df   n lower.CL upper.CL
 Placebo    1.263 0.0474 1238 260    1.170     1.36
 GPre-TPar  1.349 0.0474 1238 260    1.256     1.44
 TPre-GPar  1.293 0.0474 1238 260    1.200     1.39
 Tpar-GBst  0.975 0.0499 1238 234    0.877     1.07
 Tpre-GBst  1.164 0.0499 1238 234    1.066     1.26

Confidence level used: 0.95
```


```
eff_size(my_model.compare, sigma = sigma(my_model), edf = 23)
```


```
Block =  7:
 contrast                  effect.size     SE   df lower.CL upper.CL
 Placebo - (GPre-TPar)         -0.1196 0.0895 1238  -0.2951   0.0559
 Placebo - (TPre-GPar)         -0.0485 0.0880 1238  -0.2211   0.1242
 Placebo - (Tpar-GBst)          0.4981 0.1162 1238   0.2701   0.7262
 Placebo - (Tpre-GBst)          0.0862 0.0910 1238  -0.0923   0.2648
 (GPre-TPar) - (TPre-GPar)      0.0711 0.0883 1238  -0.1022   0.2444
 (GPre-TPar) - (Tpar-GBst)      0.6177 0.1281 1238   0.3664   0.8691
 (GPre-TPar) - (Tpre-GBst)      0.2058 0.0951 1238   0.0193   0.3924
 (TPre-GPar) - (Tpar-GBst)      0.5466 0.1209 1238   0.3094   0.7838
 (TPre-GPar) - (Tpre-GBst)      0.1347 0.0923 1238  -0.0463   0.3158
 (Tpar-GBst) - (Tpre-GBst)     -0.4119 0.1106 1238  -0.6289  -0.1949

Block = 13:
 contrast                  effect.size     SE   df lower.CL upper.CL
 Placebo - (GPre-TPar)         -0.2120 0.1687 1238  -0.5430   0.1189
 Placebo - (TPre-GPar)         -0.0743 0.1661 1238  -0.4002   0.2516
 Placebo - (Tpar-GBst)          0.7129 0.2001 1238   0.3203   1.1055
 Placebo - (Tpre-GBst)          0.2458 0.1741 1238  -0.0958   0.5874
 (GPre-TPar) - (TPre-GPar)      0.1377 0.1670 1238  -0.1899   0.4653
 (GPre-TPar) - (Tpar-GBst)      0.9249 0.2182 1238   0.4969   1.3530
 (GPre-TPar) - (Tpre-GBst)      0.4579 0.1832 1238   0.0985   0.8173
 (TPre-GPar) - (Tpar-GBst)      0.7872 0.2061 1238   0.3829   1.1915
 (TPre-GPar) - (Tpre-GBst)      0.3202 0.1767 1238  -0.0265   0.6668
 (Tpar-GBst) - (Tpre-GBst)     -0.4671 0.1878 1238  -0.8355  -0.0986

sigma used for effect sizes: 0.4042 
Confidence level used: 0.95
```

### Note:

The post-hoc tests revealed that the theta-parietal-gamma-burst
variant was significantly worse than the placebo, as well as
significantly worse than the gamma-prefrontal-theta-parietal and the
theta-prefrontal-gamma-parietal. Note that in this model we run post-hoc
tests at block 7 (i.e., mid-point of the fitted line, which is the
default in emmeans) and at block 13, which was the last block of each
session. The reason was to compare the performance of the participants
at the end of each session, as there seems to be a steep improvement in
performance over the course of training, on average. When looking at the
endpoint of the lines fitted to the normalized accuracy and, especially,
when comparing the gamma-prefrontal-theta-parietal condition to placebo
in the 2-back task, it seems like participants reach a higher point in
performance towards the end of training. This difference was not
statistically significant, however. When plotting individual points
(please see the plot for the normalized accuracy in the Supplementary
Materials), we can see a single lone data point above 4, which may be
driving the steeper slope observed.

### Normalized accuracy in the 3-back task


```
data_subset <- subset(df_epfl, Task == '3back')
data_subset <- droplevels(data_subset)
levels(data_subset$Task)
```


```
[1] "3back"
```


```
m_acc_norm_3back <- lm(formula = Acc_norm ~ Stim*Block, data=data_subset)
summary(m_acc_norm_3back)
```


```
Call:
lm(formula = Acc_norm ~ Stim * Block, data = data_subset)

Residuals:
    Min      1Q  Median      3Q     Max 
-1.1164 -0.1884 -0.0494  0.1323  2.8366 

Coefficients:
                     Estimate Std. Error t value Pr(>|t|)    
(Intercept)          1.150932   0.046998  24.489   <2e-16 ***
StimGPre-TPar       -0.066899   0.066465  -1.007   0.3144    
StimTPre-GPar       -0.043449   0.066465  -0.654   0.5134    
StimTpar-GBst       -0.148192   0.068286  -2.170   0.0302 *  
StimTpre-GBst       -0.153588   0.068286  -2.249   0.0247 *  
Block                0.006241   0.005921   1.054   0.2921    
StimGPre-TPar:Block -0.004668   0.008374  -0.557   0.5774    
StimTPre-GPar:Block -0.004966   0.008374  -0.593   0.5533    
StimTpar-GBst:Block -0.002508   0.008603  -0.292   0.7707    
StimTpre-GBst:Block -0.007395   0.008603  -0.860   0.3902    
---
Signif. codes:  0 ‘***’ 0.001 ‘**’ 0.01 ‘*’ 0.05 ‘.’ 0.1 ‘ ’ 1

Residual standard error: 0.3572 on 1238 degrees of freedom
  (52 observations deleted due to missingness)
Multiple R-squared:  0.03942,   Adjusted R-squared:  0.03244 
F-statistic: 5.646 on 9 and 1238 DF,  p-value: 1.107e-07
```


```
anova(m_acc_norm_3back)
```


```
Analysis of Variance Table

Response: Acc_norm
             Df  Sum Sq Mean Sq F value    Pr(>F)    
Stim          4   6.278 1.56944 12.2978 8.259e-10 ***
Block         1   0.099 0.09873  0.7736    0.3793    
Stim:Block    4   0.108 0.02700  0.2116    0.9321    
Residuals  1238 157.993 0.12762                      
---
Signif. codes:  0 ‘***’ 0.001 ‘**’ 0.01 ‘*’ 0.05 ‘.’ 0.1 ‘ ’ 1
```


```
eta_squared(m_acc_norm_3back)
```


```
# Effect Size for ANOVA (Type I)

Parameter  | Eta2 (partial) |       95% CI
------------------------------------------
Stim       |           0.04 | [0.02, 1.00]
Block      |       6.25e-04 | [0.00, 1.00]
Stim:Block |       6.83e-04 | [0.00, 1.00]

- One-sided CIs: upper bound fixed at [1.00].
```


```
my_model <- m_acc_norm_3back
level_1 <- "Stim"

my_model.compare <- emmeans(my_model, level_1, by="Block", at = list(Block = c(7, 13)))
my_model.compare.pairs <- pairs(my_model.compare, adjust='tukey')
test(my_model.compare.pairs, side='two-sided')
```


```
Block =  7:
 contrast                  estimate     SE   df t.ratio p.value
 Placebo - (GPre-TPar)       0.0996 0.0313 1238   3.178  0.0132
 Placebo - (TPre-GPar)       0.0782 0.0313 1238   2.496  0.0921
 Placebo - (Tpar-GBst)       0.1657 0.0322 1238   5.149  <.0001
 Placebo - (Tpre-GBst)       0.2054 0.0322 1238   6.379  <.0001
 (GPre-TPar) - (TPre-GPar)  -0.0214 0.0313 1238  -0.682  0.9604
 (GPre-TPar) - (Tpar-GBst)   0.0662 0.0322 1238   2.056  0.2403
 (GPre-TPar) - (Tpre-GBst)   0.1058 0.0322 1238   3.286  0.0092
 (TPre-GPar) - (Tpar-GBst)   0.0875 0.0322 1238   2.719  0.0517
 (TPre-GPar) - (Tpre-GBst)   0.1271 0.0322 1238   3.950  0.0008
 (Tpar-GBst) - (Tpre-GBst)   0.0396 0.0330 1238   1.199  0.7518

Block = 13:
 contrast                  estimate     SE   df t.ratio p.value
 Placebo - (GPre-TPar)       0.1276 0.0592 1238   2.155  0.1979
 Placebo - (TPre-GPar)       0.1080 0.0592 1238   1.824  0.3601
 Placebo - (Tpar-GBst)       0.1808 0.0608 1238   2.972  0.0251
 Placebo - (Tpre-GBst)       0.2497 0.0608 1238   4.105  0.0004
 (GPre-TPar) - (TPre-GPar)  -0.0196 0.0592 1238  -0.331  0.9974
 (GPre-TPar) - (Tpar-GBst)   0.0532 0.0608 1238   0.875  0.9062
 (GPre-TPar) - (Tpre-GBst)   0.1222 0.0608 1238   2.008  0.2627
 (TPre-GPar) - (Tpar-GBst)   0.0728 0.0608 1238   1.197  0.7534
 (TPre-GPar) - (Tpre-GBst)   0.1417 0.0608 1238   2.330  0.1362
 (Tpar-GBst) - (Tpre-GBst)   0.0689 0.0624 1238   1.104  0.8043

P value adjustment: tukey method for comparing a family of 5 estimates
```


```
confint(my_model.compare, calc = c(n = ~.wgt.))
```


```
Block =  7:
 Stim      emmean     SE   df   n lower.CL upper.CL
 Placebo    1.195 0.0222 1238 260    1.151     1.24
 GPre-TPar  1.095 0.0222 1238 260    1.052     1.14
 TPre-GPar  1.116 0.0222 1238 260    1.073     1.16
 Tpar-GBst  1.029 0.0234 1238 234    0.983     1.07
 Tpre-GBst  0.989 0.0234 1238 234    0.943     1.04

Block = 13:
 Stim      emmean     SE   df   n lower.CL upper.CL
 Placebo    1.232 0.0419 1238 260    1.150     1.31
 GPre-TPar  1.104 0.0419 1238 260    1.022     1.19
 TPre-GPar  1.124 0.0419 1238 260    1.042     1.21
 Tpar-GBst  1.051 0.0441 1238 234    0.965     1.14
 Tpre-GBst  0.982 0.0441 1238 234    0.896     1.07

Confidence level used: 0.95
```


```
eff_size(my_model.compare, sigma = sigma(my_model), edf = 23)
```


```
Block =  7:
 contrast                  effect.size     SE   df  lower.CL upper.CL
 Placebo - (GPre-TPar)          0.2787 0.0969 1238  0.088707    0.469
 Placebo - (TPre-GPar)          0.2189 0.0935 1238  0.035572    0.402
 Placebo - (Tpar-GBst)          0.4640 0.1131 1238  0.242014    0.686
 Placebo - (Tpre-GBst)          0.5748 0.1237 1238  0.332146    0.818
 (GPre-TPar) - (TPre-GPar)     -0.0598 0.0881 1238 -0.232740    0.113
 (GPre-TPar) - (Tpar-GBst)      0.1852 0.0942 1238  0.000517    0.370
 (GPre-TPar) - (Tpre-GBst)      0.2961 0.1001 1238  0.099673    0.493
 (TPre-GPar) - (Tpar-GBst)      0.2450 0.0971 1238  0.054582    0.436
 (TPre-GPar) - (Tpre-GBst)      0.3559 0.1043 1238  0.151342    0.560
 (Tpar-GBst) - (Tpre-GBst)      0.1109 0.0939 1238 -0.073317    0.295

Block = 13:
 contrast                  effect.size     SE   df  lower.CL upper.CL
 Placebo - (GPre-TPar)          0.3571 0.1739 1238  0.015930    0.698
 Placebo - (TPre-GPar)          0.3023 0.1716 1238 -0.034408    0.639
 Placebo - (Tpar-GBst)          0.5061 0.1859 1238  0.141339    0.871
 Placebo - (Tpre-GBst)          0.6991 0.1991 1238  0.308535    1.090
 (GPre-TPar) - (TPre-GPar)     -0.0548 0.1659 1238 -0.380363    0.271
 (GPre-TPar) - (Tpar-GBst)      0.1490 0.1717 1238 -0.187885    0.486
 (GPre-TPar) - (Tpre-GBst)      0.3419 0.1776 1238 -0.006493    0.690
 (TPre-GPar) - (Tpar-GBst)      0.2038 0.1729 1238 -0.135479    0.543
 (TPre-GPar) - (Tpre-GBst)      0.3967 0.1801 1238  0.043479    0.750
 (Tpar-GBst) - (Tpre-GBst)      0.1930 0.1770 1238 -0.154325    0.540

sigma used for effect sizes: 0.3572 
Confidence level used: 0.95
```

## Speed

Now, we present the same type of statistical analysis on the speed of
the participants. Please note for this comparison, we consider the speed
of correct trials only.


```
m0 <- lm(formula = Speed_corr ~ Stim*Task, data=df_epfl)
summary(m0)
```


```
Call:
lm(formula = Speed_corr ~ Stim * Task, data = df_epfl)

Residuals:
    Min      1Q  Median      3Q     Max 
-773.31 -253.47  -37.54  210.54 1420.25 

Coefficients:
                        Estimate Std. Error t value Pr(>|t|)    
(Intercept)             1122.249     20.503  54.737  < 2e-16 ***
StimGPre-TPar            -47.910     28.995  -1.652   0.0986 .  
StimTPre-GPar            -28.311     28.995  -0.976   0.3290    
StimTpar-GBst           -136.705     29.823  -4.584 4.79e-06 ***
StimTpre-GBst           -125.701     29.790  -4.220 2.54e-05 ***
Task3back                 58.645     28.995   2.023   0.0432 *  
StimGPre-TPar:Task3back   25.866     41.005   0.631   0.5282    
StimTPre-GPar:Task3back   29.731     41.025   0.725   0.4687    
StimTpar-GBst:Task3back   -8.744     42.153  -0.207   0.8357    
StimTpre-GBst:Task3back    7.817     42.129   0.186   0.8528    
---
Signif. codes:  0 ‘***’ 0.001 ‘**’ 0.01 ‘*’ 0.05 ‘.’ 0.1 ‘ ’ 1

Residual standard error: 330.6 on 2484 degrees of freedom
  (106 observations deleted due to missingness)
Multiple R-squared:  0.04019,   Adjusted R-squared:  0.03671 
F-statistic: 11.56 on 9 and 2484 DF,  p-value: < 2.2e-16
```


```
anova(m0)
```


```
Analysis of Variance Table

Response: Speed_corr
            Df    Sum Sq Mean Sq F value    Pr(>F)    
Stim         4   8170058 2042514 18.6882 3.790e-15 ***
Task         1   3059949 3059949 27.9974 1.321e-07 ***
Stim:Task    4    136575   34144  0.3124    0.8698    
Residuals 2484 271486623  109294                      
---
Signif. codes:  0 ‘***’ 0.001 ‘**’ 0.01 ‘*’ 0.05 ‘.’ 0.1 ‘ ’ 1
```


### Linear model, no random effects


```
m1 <- lm(formula = Speed_corr ~ Stim*Task*Block, data=df_epfl)
summary(m1)
```


```
Call:
lm(formula = Speed_corr ~ Stim * Task * Block, data = df_epfl)

Residuals:
    Min      1Q  Median      3Q     Max 
-763.81 -254.37  -32.58  212.20 1439.37 

Coefficients:
                               Estimate Std. Error t value Pr(>|t|)    
(Intercept)                   1189.1783    43.4176  27.389   <2e-16 ***
StimGPre-TPar                  -10.9560    61.4017  -0.178   0.8584    
StimTPre-GPar                  -58.9770    61.4017  -0.961   0.3369    
StimTpar-GBst                 -141.3562    63.3399  -2.232   0.0257 *  
StimTpre-GBst                 -157.0046    63.0843  -2.489   0.0129 *  
Task3back                      -12.0677    61.4017  -0.197   0.8442    
Block                           -9.5613     5.4701  -1.748   0.0806 .  
StimGPre-TPar:Task3back         16.8502    86.8351   0.194   0.8462    
StimTPre-GPar:Task3back        130.7438    86.8445   1.505   0.1323    
StimTpar-GBst:Task3back         33.0831    89.3956   0.370   0.7114    
StimTpre-GBst:Task3back         54.2862    89.2146   0.608   0.5429    
StimGPre-TPar:Block             -5.2792     7.7359  -0.682   0.4950    
StimTPre-GPar:Block              4.3808     7.7359   0.566   0.5712    
StimTpar-GBst:Block              0.6971     7.9712   0.087   0.9303    
StimTpre-GBst:Block              4.4719     7.9479   0.563   0.5737    
Task3back:Block                 10.1019     7.7359   1.306   0.1917    
StimGPre-TPar:Task3back:Block    1.2879    10.9402   0.118   0.9063    
StimTPre-GPar:Task3back:Block  -14.4303    10.9402  -1.319   0.1873    
StimTpar-GBst:Task3back:Block   -6.0079    11.2565  -0.534   0.5936    
StimTpre-GBst:Task3back:Block   -6.6385    11.2400  -0.591   0.5548    
---
Signif. codes:  0 ‘***’ 0.001 ‘**’ 0.01 ‘*’ 0.05 ‘.’ 0.1 ‘ ’ 1

Residual standard error: 330 on 2474 degrees of freedom
  (106 observations deleted due to missingness)
Multiple R-squared:  0.04736,   Adjusted R-squared:  0.04004 
F-statistic: 6.473 on 19 and 2474 DF,  p-value: < 2.2e-16
```


```
anova(m1)
```


```
Analysis of Variance Table

Response: Speed_corr
                  Df    Sum Sq Mean Sq F value    Pr(>F)    
Stim               4   8170058 2042514 18.7531 3.359e-15 ***
Task               1   3059949 3059949 28.0946 1.257e-07 ***
Block              1   1376061 1376061 12.6341 0.0003859 ***
Stim:Task          4    137338   34334  0.3152 0.8679333    
Stim:Block         4    148586   37146  0.3411 0.8503584    
Task:Block         1    217615  217615  1.9980 0.1576323    
Stim:Task:Block    4    285263   71316  0.6548 0.6234995    
Residuals       2474 269458334  108916                      
---
Signif. codes:  0 ‘***’ 0.001 ‘**’ 0.01 ‘*’ 0.05 ‘.’ 0.1 ‘ ’ 1
```


Compare models


```
anova(m0, m1)
```


```
Analysis of Variance Table

Model 1: Speed_corr ~ Stim * Task
Model 2: Speed_corr ~ Stim * Task * Block
  Res.Df       RSS Df Sum of Sq      F  Pr(>F)  
1   2484 271486623                              
2   2474 269458334 10   2028289 1.8622 0.04589 *
---
Signif. codes:  0 ‘***’ 0.001 ‘**’ 0.01 ‘*’ 0.05 ‘.’ 0.1 ‘ ’ 1
```


Linear model, random intercept and random slope Random Intercept per
subject


```
m2 <- lmer(formula = Speed_corr ~ Stim*Task*Block + (1 | ID), data=df_epfl)
summary(m2)
```


```
Linear mixed model fit by REML. t-tests use Satterthwaite's method ['lmerModLmerTest']
Formula: Speed_corr ~ Stim * Task * Block + (1 | ID)
   Data: df_epfl

REML criterion at convergence: 33330.5

Scaled residuals: 
    Min      1Q  Median      3Q     Max 
-3.7880 -0.6059 -0.0384  0.5205  6.0788 

Random effects:
 Groups   Name        Variance Std.Dev.
 ID       (Intercept) 76611    276.8   
 Residual             37673    194.1   
Number of obs: 2494, groups:  ID, 20

Fixed effects:
                               Estimate Std. Error        df t value Pr(>|t|)    
(Intercept)                   1189.1783    66.9520   25.7957  17.762 5.52e-16 ***
StimGPre-TPar                  -10.9560    36.1121 2454.9939  -0.303 0.761619    
StimTPre-GPar                  -58.9770    36.1121 2454.9939  -1.633 0.102562    
StimTpar-GBst                 -131.2201    37.2888 2455.0542  -3.519 0.000441 ***
StimTpre-GBst                 -146.4733    37.1375 2455.0536  -3.944 8.24e-05 ***
Task3back                      -12.0677    36.1121 2454.9939  -0.334 0.738277    
Block                           -9.5613     3.2171 2454.9939  -2.972 0.002987 ** 
StimGPre-TPar:Task3back         16.8502    51.0702 2454.9939   0.330 0.741471    
StimTPre-GPar:Task3back        128.9855    51.0757 2454.9939   2.525 0.011620 *  
StimTpar-GBst:Task3back         33.4784    52.5768 2454.9945   0.637 0.524345    
StimTpre-GBst:Task3back         54.2862    52.4696 2454.9939   1.035 0.300947    
StimGPre-TPar:Block             -5.2792     4.5497 2454.9939  -1.160 0.246026    
StimTPre-GPar:Block              4.3808     4.5497 2454.9939   0.963 0.335701    
StimTpar-GBst:Block              0.7394     4.6882 2454.9947   0.158 0.874686    
StimTpre-GBst:Block              4.4719     4.6744 2454.9939   0.957 0.338816    
Task3back:Block                 10.1019     4.5497 2454.9939   2.220 0.026487 *  
StimGPre-TPar:Task3back:Block    1.2879     6.4342 2454.9939   0.200 0.841369    
StimTPre-GPar:Task3back:Block  -14.4303     6.4342 2454.9939  -2.243 0.025002 *  
StimTpar-GBst:Task3back:Block   -6.0502     6.6203 2454.9943  -0.914 0.360866    
StimTpre-GBst:Task3back:Block   -6.6385     6.6105 2454.9939  -1.004 0.315366    
---
Signif. codes:  0 ‘***’ 0.001 ‘**’ 0.01 ‘*’ 0.05 ‘.’ 0.1 ‘ ’ 1
```


```
Correlation matrix not shown by default, as p = 20 > 12.
Use print(x, correlation=TRUE)  or
    vcov(x)        if you need it
```


```
anova(m2)
```


```
Type III Analysis of Variance Table with Satterthwaite's method
                 Sum Sq Mean Sq NumDF  DenDF F value    Pr(>F)    
Stim            1791584  447896     4 2455.1 11.8889 1.440e-09 ***
Task             165778  165778     1 2455.0  4.4004   0.03603 *  
Block           1351437 1351437     1 2455.0 35.8724 2.416e-09 ***
Stim:Task        290266   72567     4 2455.0  1.9262   0.10338    
Stim:Block       148519   37130     4 2455.0  0.9856   0.41409    
Task:Block       211994  211994     1 2455.0  5.6271   0.01776 *  
Stim:Task:Block  285390   71347     4 2455.0  1.8938   0.10880    
---
Signif. codes:  0 ‘***’ 0.001 ‘**’ 0.01 ‘*’ 0.05 ‘.’ 0.1 ‘ ’ 1
```


#### Compare models

When comparing a linear model (lm) to a linear mixed-effect model
(lme), the regular ANOVA does not work, so I will only look at the
information criteria


```
AIC(m1, m2)
```


```
BIC(m1, m2)
```


The model including random intercepts is better than the regular
linear model in this case.

Random intercept and random slope per subject


```
m3 <- lmer(formula = Speed_corr ~ Stim*Task*Block + (1 + Block|ID), data=df_epfl)
```


```
Warning: Model failed to converge with max|grad| = 0.286966 (tol = 0.002, component 1)
```


```
summary(m3)
```


```
Linear mixed model fit by REML. t-tests use Satterthwaite's method ['lmerModLmerTest']
Formula: Speed_corr ~ Stim * Task * Block + (1 + Block | ID)
   Data: df_epfl

REML criterion at convergence: 33328.7

Scaled residuals: 
    Min      1Q  Median      3Q     Max 
-3.8033 -0.5995 -0.0470  0.5234  6.0798 

Random effects:
 Groups   Name        Variance Std.Dev. Corr 
 ID       (Intercept) 72311.62 268.908       
          Block          10.69   3.269  -0.09
 Residual             37549.00 193.776       
Number of obs: 2494, groups:  ID, 20

Fixed effects:
                               Estimate Std. Error        df t value Pr(>|t|)    
(Intercept)                   1189.1783    65.3105   28.0777  18.208  < 2e-16 ***
StimGPre-TPar                  -10.9560    36.0524 2435.3660  -0.304 0.761236    
StimTPre-GPar                  -58.9770    36.0524 2435.3660  -1.636 0.101996    
StimTpar-GBst                 -130.7900    37.2580 2449.3728  -3.510 0.000456 ***
StimTpre-GBst                 -145.9093    37.1061 2449.1800  -3.932 8.65e-05 ***
Task3back                      -12.0677    36.0524 2435.3660  -0.335 0.737860    
Block                           -9.5613     3.2939  683.6914  -2.903 0.003819 ** 
StimGPre-TPar:Task3back         16.8502    50.9857 2435.3660   0.330 0.741059    
StimTPre-GPar:Task3back        128.9859    50.9913 2435.3660   2.530 0.011483 *  
StimTpar-GBst:Task3back         33.6122    52.4906 2435.6255   0.640 0.522007    
StimTpre-GBst:Task3back         54.2862    52.3829 2435.3660   1.036 0.300149    
StimGPre-TPar:Block             -5.2792     4.5422 2435.3660  -1.162 0.245246    
StimTPre-GPar:Block              4.3808     4.5422 2435.3660   0.964 0.334902    
StimTpar-GBst:Block              0.6726     4.6853 2451.6442   0.144 0.885856    
StimTpre-GBst:Block              4.3908     4.6715 2451.5525   0.940 0.347354    
Task3back:Block                 10.1019     4.5422 2435.3660   2.224 0.026239 *  
StimGPre-TPar:Task3back:Block    1.2879     6.4236 2435.3660   0.200 0.841110    
StimTPre-GPar:Task3back:Block  -14.4303     6.4236 2435.3660  -2.246 0.024764 *  
StimTpar-GBst:Task3back:Block   -6.0646     6.6094 2435.5541  -0.918 0.358940    
StimTpre-GBst:Task3back:Block   -6.6385     6.5996 2435.3660  -1.006 0.314566    
---
Signif. codes:  0 ‘***’ 0.001 ‘**’ 0.01 ‘*’ 0.05 ‘.’ 0.1 ‘ ’ 1
```


```
Correlation matrix not shown by default, as p = 20 > 12.
Use print(x, correlation=TRUE)  or
    vcov(x)        if you need it
```


```
optimizer (nloptwrap) convergence code: 0 (OK)
Model failed to converge with max|grad| = 0.286966 (tol = 0.002, component 1)
```


```
anova(m3)
```


```
Type III Analysis of Variance Table with Satterthwaite's method
                 Sum Sq Mean Sq NumDF   DenDF F value    Pr(>F)    
Stim            1762132  440533     4 2440.14 11.7322 1.936e-09 ***
Task             166034  166034     1 2435.47  4.4218   0.03559 *  
Block            908641  908641     1   18.75 24.1988 9.873e-05 ***
Stim:Task        290176   72544     4 2435.47  1.9320   0.10244    
Stim:Block       145688   36422     4 2434.95  0.9700   0.42270    
Task:Block       211746  211746     1 2435.44  5.6392   0.01764 *  
Stim:Task:Block  285433   71358     4 2435.44  1.9004   0.10768    
---
Signif. codes:  0 ‘***’ 0.001 ‘**’ 0.01 ‘*’ 0.05 ‘.’ 0.1 ‘ ’ 1
```

#### Compare models, even though this last one did not converge


```
anova(m2, m3)
```


```
refitting model(s) with ML (instead of REML)
```


```
Data: df_epfl
Models:
m2: Speed_corr ~ Stim * Task * Block + (1 | ID)
m3: Speed_corr ~ Stim * Task * Block + (1 + Block | ID)
   npar   AIC   BIC logLik deviance  Chisq Df Pr(>Chisq)
m2   22 33491 33619 -16723    33447                     
m3   24 33493 33633 -16723    33445 1.4608  2     0.4817
```

### Note:

Adding a random slope does not improve the amount of variance
explained by the model, besides the fact that the model including a
random slope did not converge.

### Model with random intercept per participant and per day, trying to account for differences between session days


```
m4 <- lmer(formula = Speed_corr ~ Stim*Task*Block + (1 + (1|ID) + (1|Day)), data=df_epfl)
summary(m4)
```


```
Linear mixed model fit by REML. t-tests use Satterthwaite's method ['lmerModLmerTest']
Formula: Speed_corr ~ Stim * Task * Block + (1 + (1 | ID) + (1 | Day))
   Data: df_epfl

REML criterion at convergence: 33311.3

Scaled residuals: 
    Min      1Q  Median      3Q     Max 
-3.7865 -0.5855 -0.0370  0.5166  6.2352 

Random effects:
 Groups   Name        Variance Std.Dev.
 ID       (Intercept) 76622.2  276.81  
 Day      (Intercept)   645.8   25.41  
 Residual             37276.8  193.07  
Number of obs: 2494, groups:  ID, 20; Day, 5

Fixed effects:
                              Estimate Std. Error       df t value Pr(>|t|)    
(Intercept)                   1187.257     68.501   27.581  17.332 2.33e-16 ***
StimGPre-TPar                   -8.477     35.926 2452.145  -0.236  0.81349    
StimTPre-GPar                  -55.691     36.012 2454.201  -1.546  0.12212    
StimTpar-GBst                 -129.031     43.758   30.373  -2.949  0.00609 ** 
StimTpre-GBst                 -144.549     43.631   30.023  -3.313  0.00241 ** 
Task3back                      -12.068     35.921 2452.010  -0.336  0.73694    
Block                           -9.561      3.200 2452.010  -2.988  0.00284 ** 
StimGPre-TPar:Task3back         16.850     50.801 2452.010   0.332  0.74015    
StimTPre-GPar:Task3back        128.905     50.806 2452.010   2.537  0.01124 *  
StimTpar-GBst:Task3back         33.213     52.299 2452.013   0.635  0.52545    
StimTpre-GBst:Task3back         54.286     52.193 2452.010   1.040  0.29839    
StimGPre-TPar:Block             -5.279      4.526 2452.010  -1.166  0.24353    
StimTPre-GPar:Block              4.381      4.526 2452.010   0.968  0.33314    
StimTpar-GBst:Block              0.711      4.663 2452.014   0.152  0.87883    
StimTpre-GBst:Block              4.472      4.650 2452.010   0.962  0.33626    
Task3back:Block                 10.102      4.526 2452.010   2.232  0.02570 *  
StimGPre-TPar:Task3back:Block    1.288      6.400 2452.010   0.201  0.84054    
StimTPre-GPar:Task3back:Block  -14.430      6.400 2452.010  -2.255  0.02424 *  
StimTpar-GBst:Task3back:Block   -6.022      6.585 2452.012  -0.914  0.36059    
StimTpre-GBst:Task3back:Block   -6.638      6.576 2452.010  -1.010  0.31281    
---
Signif. codes:  0 ‘***’ 0.001 ‘**’ 0.01 ‘*’ 0.05 ‘.’ 0.1 ‘ ’ 1
```


```
Correlation matrix not shown by default, as p = 20 > 12.
Use print(x, correlation=TRUE)  or
    vcov(x)        if you need it
```


```
anova(m4)
```


```
Type III Analysis of Variance Table with Satterthwaite's method
                 Sum Sq Mean Sq NumDF   DenDF F value    Pr(>F)    
Stim             631983  157996     4   41.86  4.2385  0.005705 ** 
Task             165117  165117     1 2452.01  4.4295  0.035425 *  
Block           1352669 1352669     1 2452.01 36.2872 1.959e-09 ***
Stim:Task        290068   72517     4 2452.01  1.9454  0.100284    
Stim:Block       148616   37154     4 2452.01  0.9967  0.408011    
Task:Block       212482  212482     1 2452.01  5.7001  0.017039 *  
Stim:Task:Block  285304   71326     4 2452.01  1.9134  0.105488    
---
Signif. codes:  0 ‘***’ 0.001 ‘**’ 0.01 ‘*’ 0.05 ‘.’ 0.1 ‘ ’ 1
```


#### Compare models


```
anova(m2, m4)
```


```
refitting model(s) with ML (instead of REML)
```


```
Data: df_epfl
Models:
m2: Speed_corr ~ Stim * Task * Block + (1 | ID)
m4: Speed_corr ~ Stim * Task * Block + (1 + (1 | ID) + (1 | Day))
   npar   AIC   BIC logLik deviance Chisq Df Pr(>Chisq)    
m2   22 33491 33619 -16723    33447                        
m4   23 33475 33609 -16715    33429 17.28  1  3.225e-05 ***
---
Signif. codes:  0 ‘***’ 0.001 ‘**’ 0.01 ‘*’ 0.05 ‘.’ 0.1 ‘ ’ 1
```

### Model choice

The model including random intercepts for each subject on each day
significantly improves the one with only random intercepts for
individuals, so this is the model we will use for the speed.


```
m_speed <- lmer(formula = Speed_corr ~ Stim*Task*Block + (1 + (1|ID) + (1|Day)), data=df_epfl)
summary(m_speed)
```


```
Linear mixed model fit by REML. t-tests use Satterthwaite's method ['lmerModLmerTest']
Formula: Speed_corr ~ Stim * Task * Block + (1 + (1 | ID) + (1 | Day))
   Data: df_epfl

REML criterion at convergence: 33311.3

Scaled residuals: 
    Min      1Q  Median      3Q     Max 
-3.7865 -0.5855 -0.0370  0.5166  6.2352 

Random effects:
 Groups   Name        Variance Std.Dev.
 ID       (Intercept) 76622.2  276.81  
 Day      (Intercept)   645.8   25.41  
 Residual             37276.8  193.07  
Number of obs: 2494, groups:  ID, 20; Day, 5

Fixed effects:
                              Estimate Std. Error       df t value Pr(>|t|)    
(Intercept)                   1187.257     68.501   27.581  17.332 2.33e-16 ***
StimGPre-TPar                   -8.477     35.926 2452.145  -0.236  0.81349    
StimTPre-GPar                  -55.691     36.012 2454.201  -1.546  0.12212    
StimTpar-GBst                 -129.031     43.758   30.373  -2.949  0.00609 ** 
StimTpre-GBst                 -144.549     43.631   30.023  -3.313  0.00241 ** 
Task3back                      -12.068     35.921 2452.010  -0.336  0.73694    
Block                           -9.561      3.200 2452.010  -2.988  0.00284 ** 
StimGPre-TPar:Task3back         16.850     50.801 2452.010   0.332  0.74015    
StimTPre-GPar:Task3back        128.905     50.806 2452.010   2.537  0.01124 *  
StimTpar-GBst:Task3back         33.213     52.299 2452.013   0.635  0.52545    
StimTpre-GBst:Task3back         54.286     52.193 2452.010   1.040  0.29839    
StimGPre-TPar:Block             -5.279      4.526 2452.010  -1.166  0.24353    
StimTPre-GPar:Block              4.381      4.526 2452.010   0.968  0.33314    
StimTpar-GBst:Block              0.711      4.663 2452.014   0.152  0.87883    
StimTpre-GBst:Block              4.472      4.650 2452.010   0.962  0.33626    
Task3back:Block                 10.102      4.526 2452.010   2.232  0.02570 *  
StimGPre-TPar:Task3back:Block    1.288      6.400 2452.010   0.201  0.84054    
StimTPre-GPar:Task3back:Block  -14.430      6.400 2452.010  -2.255  0.02424 *  
StimTpar-GBst:Task3back:Block   -6.022      6.585 2452.012  -0.914  0.36059    
StimTpre-GBst:Task3back:Block   -6.638      6.576 2452.010  -1.010  0.31281    
---
Signif. codes:  0 ‘***’ 0.001 ‘**’ 0.01 ‘*’ 0.05 ‘.’ 0.1 ‘ ’ 1
```


```
Correlation matrix not shown by default, as p = 20 > 12.
Use print(x, correlation=TRUE)  or
    vcov(x)        if you need it
```


```
anova(m_speed)
```


```
Type III Analysis of Variance Table with Satterthwaite's method
                 Sum Sq Mean Sq NumDF   DenDF F value    Pr(>F)    
Stim             631983  157996     4   41.86  4.2385  0.005705 ** 
Task             165117  165117     1 2452.01  4.4295  0.035425 *  
Block           1352669 1352669     1 2452.01 36.2872 1.959e-09 ***
Stim:Task        290068   72517     4 2452.01  1.9454  0.100284    
Stim:Block       148616   37154     4 2452.01  0.9967  0.408011    
Task:Block       212482  212482     1 2452.01  5.7001  0.017039 *  
Stim:Task:Block  285304   71326     4 2452.01  1.9134  0.105488    
---
Signif. codes:  0 ‘***’ 0.001 ‘**’ 0.01 ‘*’ 0.05 ‘.’ 0.1 ‘ ’ 1
```


```
eta_squared(m_speed)
```


```
# Effect Size for ANOVA (Type III)

Parameter       | Eta2 (partial) |       95% CI
-----------------------------------------------
Stim            |           0.29 | [0.06, 1.00]
Task            |       1.80e-03 | [0.00, 1.00]
Block           |           0.01 | [0.01, 1.00]
Stim:Task       |       3.16e-03 | [0.00, 1.00]
Stim:Block      |       1.62e-03 | [0.00, 1.00]
Task:Block      |       2.32e-03 | [0.00, 1.00]
Stim:Task:Block |       3.11e-03 | [0.00, 1.00]

- One-sided CIs: upper bound fixed at [1.00].
```


There is a significant effect of stimulation, as well as a
significant difference between the tasks and the slopes for each task.
We will make models for each task separately.

### Speed in the 2-back task


```
data_subset <- subset(df_epfl, Task == '2back')
data_subset <- droplevels(data_subset)
levels(data_subset$Task)
```


```
[1] "2back"
```


```
m_speed_2back <- lmer(formula = Speed_corr ~ Stim*Block + (1 + (1|ID) + (1|Day)), data=data_subset)
summary(m_speed_2back)
```


```
Linear mixed model fit by REML. t-tests use Satterthwaite's method ['lmerModLmerTest']
Formula: Speed_corr ~ Stim * Block + (1 + (1 | ID) + (1 | Day))
   Data: data_subset

REML criterion at convergence: 16668.4

Scaled residuals: 
    Min      1Q  Median      3Q     Max 
-3.7238 -0.5789 -0.0526  0.4748  5.7732 

Random effects:
 Groups   Name        Variance Std.Dev.
 ID       (Intercept) 74262    272.51  
 Day      (Intercept)  1149     33.89  
 Residual             36392    190.77  
Number of obs: 1247, groups:  ID, 20; Day, 5

Fixed effects:
                     Estimate Std. Error        df t value Pr(>|t|)    
(Intercept)         1184.3479    68.7560   27.9327  17.225  < 2e-16 ***
StimGPre-TPar         -8.4632    35.5013 1215.1483  -0.238  0.81162    
StimTPre-GPar        -46.9785    35.6687 1217.3029  -1.317  0.18806    
StimTpar-GBst       -125.0476    48.0057   13.6213  -2.605  0.02115 *  
StimTpre-GBst       -141.1685    47.8912   13.4926  -2.948  0.01095 *  
Block                 -9.5613     3.1619 1215.0023  -3.024  0.00255 ** 
StimGPre-TPar:Block   -5.2792     4.4716 1215.0023  -1.181  0.23800    
StimTPre-GPar:Block    4.3808     4.4716 1215.0023   0.980  0.32743    
StimTpar-GBst:Block    0.6465     4.6079 1215.0077   0.140  0.88845    
StimTpre-GBst:Block    4.4719     4.5942 1215.0023   0.973  0.33055    
---
Signif. codes:  0 ‘***’ 0.001 ‘**’ 0.01 ‘*’ 0.05 ‘.’ 0.1 ‘ ’ 1

Correlation of Fixed Effects:
                   (Intr) StGP-TP StTP-GP StimTpar-GBst StimTpre-GBst Block  SGP-TP: STP-GP: StimTpar-GBst:Blck
StimGPr-TPr        -0.258                                                                                      
StimTPr-GPr        -0.259  0.498                                                                               
StimTpar-GBst      -0.307  0.370   0.370                                                                       
StimTpre-GBst      -0.308  0.371   0.371   0.693                                                               
Block              -0.322  0.623   0.621   0.461         0.462                                                 
StmGPr-TP:B         0.228 -0.882  -0.439  -0.326        -0.327        -0.707                                   
StmTPr-GP:B         0.228 -0.441  -0.878  -0.326        -0.327        -0.707  0.500                            
StimTpar-GBst:Blck  0.221 -0.428  -0.426  -0.673        -0.317        -0.686  0.485   0.485                    
StimTpre-GBst:Blck  0.222 -0.429  -0.427  -0.317        -0.672        -0.688  0.487   0.487   0.472
```


```
anova(m_speed_2back)
```


```
Type III Analysis of Variance Table with Satterthwaite's method
            Sum Sq Mean Sq NumDF  DenDF F value   Pr(>F)    
Stim        392157   98039     4   44.1  2.6940  0.04297 *  
Block      1320998 1320998     1 1215.0 36.2993 2.24e-09 ***
Stim:Block  227758   56939     4 1215.0  1.5646  0.18141    
---
Signif. codes:  0 ‘***’ 0.001 ‘**’ 0.01 ‘*’ 0.05 ‘.’ 0.1 ‘ ’ 1
```


```
eta_squared(m_speed_2back)
```


```
# Effect Size for ANOVA (Type III)

Parameter  | Eta2 (partial) |       95% CI
------------------------------------------
Stim       |           0.20 | [0.00, 1.00]
Block      |           0.03 | [0.02, 1.00]
Stim:Block |       5.12e-03 | [0.00, 1.00]

- One-sided CIs: upper bound fixed at [1.00].
```


There is a significant effect of stimulation, so we will now do
post-hoc tests. Unlike what we did for the normalized accuracy, here we
will only do the default comparison halfway through the regression
lines, as the data is not corrected for differences in initial levels.
We will add a comparison of the last training block, if applicable, when
testing the normalized data.


```
my_model <- m_speed_2back
level_1 <- "Stim"

my_model.compare <- emmeans(my_model, level_1, by="Block")
my_model.compare.pairs <- pairs(my_model.compare, adjust='tukey')
test(my_model.compare.pairs, side='two-sided')
```


```
Block = 7:
 contrast                  estimate   SE      df t.ratio p.value
 Placebo - (GPre-TPar)         45.4 16.8 1215.63   2.713  0.0527
 Placebo - (TPre-GPar)         16.3 17.2 1213.24   0.949  0.8775
 Placebo - (Tpar-GBst)        120.5 35.5    4.07   3.395  0.1127
 Placebo - (Tpre-GBst)        109.8 35.5    4.07   3.095  0.1466
 (GPre-TPar) - (TPre-GPar)    -29.2 17.2 1213.24  -1.698  0.4354
 (GPre-TPar) - (Tpar-GBst)     75.1 35.5    4.07   2.115  0.3589
 (GPre-TPar) - (Tpre-GBst)     64.4 35.5    4.07   1.815  0.4697
 (TPre-GPar) - (Tpar-GBst)    104.2 35.6    4.10   2.930  0.1690
 (TPre-GPar) - (Tpre-GBst)     93.6 35.6    4.09   2.631  0.2224
 (Tpar-GBst) - (Tpre-GBst)    -10.7 17.7 1215.00  -0.605  0.9744

Degrees-of-freedom method: kenward-roger 
P value adjustment: tukey method for comparing a family of 5 estimates
```


```
confint(my_model.compare, calc = c(n = ~.wgt.))
```


```
Block = 7:
 Stim      emmean   SE   df   n lower.CL upper.CL
 Placebo     1117 65.1 22.4 260      983     1252
 GPre-TPar   1072 65.1 22.4 260      937     1207
 TPre-GPar   1101 65.1 22.5 260      966     1236
 Tpar-GBst    997 66.7 22.6 233      859     1135
 Tpre-GBst   1008 66.7 22.5 234      869     1146

Degrees-of-freedom method: kenward-roger 
Confidence level used: 0.95
```


```
eff_size(my_model.compare, sigma = sigma(my_model), edf = 23)
```


```
Block = 7:
 contrast                  effect.size     SE   df lower.CL upper.CL
 Placebo - (GPre-TPar)          0.2382 0.0946 22.4   0.0423   0.4341
 Placebo - (TPre-GPar)          0.0854 0.0909 22.4  -0.1028   0.2736
 Placebo - (Tpar-GBst)          0.6318 0.2081 22.4   0.2007   1.0628
 Placebo - (Tpre-GBst)          0.5758 0.2045 22.4   0.1522   0.9994
 (GPre-TPar) - (TPre-GPar)     -0.1528 0.0928 22.4  -0.3450   0.0393
 (GPre-TPar) - (Tpar-GBst)      0.3936 0.1949 22.4  -0.0102   0.7973
 (GPre-TPar) - (Tpre-GBst)      0.3376 0.1926 22.4  -0.0614   0.7365
 (TPre-GPar) - (Tpar-GBst)      0.5464 0.2031 22.5   0.1257   0.9670
 (TPre-GPar) - (Tpre-GBst)      0.4904 0.1999 22.5   0.0763   0.9045
 (Tpar-GBst) - (Tpre-GBst)     -0.0560 0.0929 22.5  -0.2484   0.1365

sigma used for effect sizes: 190.8 
Degrees-of-freedom method: inherited from kenward-roger when re-gridding 
Confidence level used: 0.95
```


There is a trend towards significance in the comparison between
gamma-prefrontal-theta-parietal and the placebo, in which people would
be faster when receiving stimulation.

Speed in the 3-back task


```
data_subset <- subset(df_epfl, Task == '3back')
data_subset <- droplevels(data_subset)
levels(data_subset$Task)
```


```
[1] "3back"
```


```
m_speed_3back <- lmer(formula = Speed_corr ~ Stim*Block + (1 + (1|ID) + (1|Day)), data=data_subset)
summary(m_speed_3back)
```


```
Linear mixed model fit by REML. t-tests use Satterthwaite's method ['lmerModLmerTest']
Formula: Speed_corr ~ Stim * Block + (1 + (1 | ID) + (1 | Day))
   Data: data_subset

REML criterion at convergence: 16573.4

Scaled residuals: 
    Min      1Q  Median      3Q     Max 
-3.6874 -0.5774 -0.0514  0.5403  4.7401 

Random effects:
 Groups   Name        Variance Std.Dev.
 ID       (Intercept) 83349.2  288.7   
 Day      (Intercept)   718.4   26.8   
 Residual             33628.8  183.4   
Number of obs: 1247, groups:  ID, 20; Day, 5

Fixed effects:
                     Estimate Std. Error        df t value Pr(>|t|)    
(Intercept)         1178.0881    70.6421   26.1003  16.677 1.95e-15 ***
StimGPre-TPar          8.2032    34.1266 1215.2066   0.240   0.8101    
StimTPre-GPar         64.4296    34.2889 1217.7811   1.879   0.0605 .  
StimTpar-GBst        -99.2896    42.8192   19.8285  -2.319   0.0312 *  
StimTpre-GBst        -93.7350    42.8192   19.8285  -2.189   0.0407 *  
Block                  0.5406     3.0395 1215.0021   0.178   0.8589    
StimGPre-TPar:Block   -3.9913     4.2985 1215.0021  -0.929   0.3533    
StimTPre-GPar:Block  -10.0495     4.2985 1215.0021  -2.338   0.0196 *  
StimTpar-GBst:Block   -5.3108     4.4163 1215.0021  -1.203   0.2294    
StimTpre-GBst:Block   -2.1666     4.4163 1215.0021  -0.491   0.6238    
---
Signif. codes:  0 ‘***’ 0.001 ‘**’ 0.01 ‘*’ 0.05 ‘.’ 0.1 ‘ ’ 1

Correlation of Fixed Effects:
                   (Intr) StGP-TP StTP-GP StimTpar-GBst StimTpre-GBst Block  SGP-TP: STP-GP: StimTpar-GBst:Blck
StimGPr-TPr        -0.242                                                                                      
StimTPr-GPr        -0.242  0.498                                                                               
StimTpar-GBst      -0.272  0.398   0.399                                                                       
StimTpre-GBst      -0.272  0.398   0.399   0.647                                                               
Block              -0.301  0.623   0.621   0.497         0.497                                                 
StmGPr-TP:B         0.213 -0.882  -0.439  -0.351        -0.351        -0.707                                   
StmTPr-GP:B         0.213 -0.441  -0.878  -0.351        -0.351        -0.707  0.500                            
StimTpar-GBst:Blck  0.207 -0.429  -0.427  -0.722        -0.342        -0.688  0.487   0.487                    
StimTpre-GBst:Blck  0.207 -0.429  -0.427  -0.342        -0.722        -0.688  0.487   0.487   0.474
```


```
anova(m_speed_3back)
```


```
Type III Analysis of Variance Table with Satterthwaite's method
           Sum Sq Mean Sq NumDF   DenDF F value   Pr(>F)   
Stim       578269  144567     4   54.84  4.2989 0.004264 **
Block      246757  246757     1 1215.00  7.3377 0.006847 **
Stim:Block 206222   51556     4 1215.00  1.5331 0.190227   
---
Signif. codes:  0 ‘***’ 0.001 ‘**’ 0.01 ‘*’ 0.05 ‘.’ 0.1 ‘ ’ 1
```


```
eta_squared(m_speed_3back)
```


```
# Effect Size for ANOVA (Type III)

Parameter  | Eta2 (partial) |       95% CI
------------------------------------------
Stim       |           0.24 | [0.06, 1.00]
Block      |       6.00e-03 | [0.00, 1.00]
Stim:Block |       5.02e-03 | [0.00, 1.00]

- One-sided CIs: upper bound fixed at [1.00].
```


There is a significant effect of stimulation in the 3-back task as
well. Now the post-hoc tests:


```
my_model <- m_speed_3back
level_1 <- "Stim"

my_model.compare <- emmeans(my_model, level_1, by="Block")
my_model.compare.pairs <- pairs(my_model.compare, adjust='tukey')
test(my_model.compare.pairs, side='two-sided')
```


```
Block = 7:
 contrast                  estimate   SE      df t.ratio p.value
 Placebo - (GPre-TPar)        19.74 16.1 1215.87   1.225  0.7365
 Placebo - (TPre-GPar)         5.92 16.5 1203.43   0.358  0.9965
 Placebo - (Tpar-GBst)       136.47 29.6    4.54   4.605  0.0352
 Placebo - (Tpre-GBst)       108.90 29.6    4.54   3.675  0.0777
 (GPre-TPar) - (TPre-GPar)   -13.82 16.5 1203.23  -0.836  0.9194
 (GPre-TPar) - (Tpar-GBst)   116.73 29.6    4.54   3.939  0.0615
 (GPre-TPar) - (Tpre-GBst)    89.17 29.6    4.54   3.009  0.1450
 (TPre-GPar) - (Tpar-GBst)   130.55 29.7    4.58   4.393  0.0413
 (TPre-GPar) - (Tpre-GBst)   102.98 29.7    4.58   3.465  0.0934
 (Tpar-GBst) - (Tpre-GBst)   -27.56 17.0 1215.00  -1.626  0.4810

Degrees-of-freedom method: kenward-roger 
P value adjustment: tukey method for comparing a family of 5 estimates
```


```
confint(my_model.compare, calc = c(n = ~.wgt.))
```


```
Block = 7:
 Stim      emmean   SE   df   n lower.CL upper.CL
 Placebo     1182 67.4 21.6 260     1042     1322
 GPre-TPar   1162 67.4 21.6 260     1022     1302
 TPre-GPar   1176 67.4 21.6 259     1036     1316
 Tpar-GBst   1045 68.4 22.1 234      904     1187
 Tpre-GBst   1073 68.4 22.1 234      931     1215

Degrees-of-freedom method: kenward-roger 
Confidence level used: 0.95
```


```
eff_size(my_model.compare, sigma = sigma(my_model), edf = 23)
```


```
Block = 7:
 contrast                  effect.size     SE   df lower.CL upper.CL
 Placebo - (GPre-TPar)          0.1076 0.0892 21.6  -0.0777   0.2929
 Placebo - (TPre-GPar)          0.0323 0.0903 21.6  -0.1551   0.2197
 Placebo - (Tpar-GBst)          0.7442 0.1953 21.6   0.3386   1.1497
 Placebo - (Tpre-GBst)          0.5938 0.1838 21.6   0.2123   0.9754
 (GPre-TPar) - (TPre-GPar)     -0.0754 0.0908 21.6  -0.2639   0.1132
 (GPre-TPar) - (Tpar-GBst)      0.6365 0.1869 21.6   0.2486   1.0245
 (GPre-TPar) - (Tpre-GBst)      0.4862 0.1768 21.6   0.1192   0.8533
 (TPre-GPar) - (Tpar-GBst)      0.7119 0.1931 21.6   0.3111   1.1127
 (TPre-GPar) - (Tpre-GBst)      0.5616 0.1820 21.6   0.1838   0.9394
 (Tpar-GBst) - (Tpre-GBst)     -0.1503 0.0951 22.1  -0.3474   0.0468

sigma used for effect sizes: 183.4 
Degrees-of-freedom method: inherited from kenward-roger when re-gridding 
Confidence level used: 0.95
```


The results show participants were significantly faster when
receiving theta-parietal-gammaburst-prefrontal compared to when they
received the placebo. In turn, their speed was significantly higher to
the one they had when receiving theta-prefrontal-gamma-parietal
stimulation.

## Normalized Speed

Group averages


```
m0 <- lm(formula = Speed_norm ~ Stim*Task, data=df_epfl)
summary(m0)
```


```
Call:
lm(formula = Speed_norm ~ Stim * Task, data = df_epfl)

Residuals:
     Min       1Q   Median       3Q      Max 
-0.62012 -0.14275 -0.00588  0.11644  1.76880 

Coefficients:
                        Estimate Std. Error t value Pr(>|t|)    
(Intercept)              0.98979    0.01326  74.655  < 2e-16 ***
StimGPre-TPar           -0.09525    0.01875  -5.080 4.05e-07 ***
StimTPre-GPar           -0.01572    0.01875  -0.839   0.4017    
StimTpar-GBst           -0.09183    0.01956  -4.695 2.81e-06 ***
StimTpre-GBst           -0.01354    0.01926  -0.703   0.4821    
Task3back                0.01275    0.01875   0.680   0.4966    
StimGPre-TPar:Task3back  0.05933    0.02652   2.237   0.0254 *  
StimTPre-GPar:Task3back -0.03659    0.02653  -1.379   0.1680    
StimTpar-GBst:Task3back  0.03669    0.02745   1.336   0.1816    
StimTpre-GBst:Task3back  0.04255    0.02724   1.562   0.1184    
---
Signif. codes:  0 ‘***’ 0.001 ‘**’ 0.01 ‘*’ 0.05 ‘.’ 0.1 ‘ ’ 1

Residual standard error: 0.2138 on 2472 degrees of freedom
  (118 observations deleted due to missingness)
Multiple R-squared:  0.03437,   Adjusted R-squared:  0.03086 
F-statistic: 9.777 on 9 and 2472 DF,  p-value: 7.874e-15
```


```
anova(m0)
```


```
Analysis of Variance Table

Response: Speed_norm
            Df  Sum Sq Mean Sq F value    Pr(>F)    
Stim         4   2.616 0.65390 14.3077   1.5e-11 ***
Task         1   0.646 0.64629 14.1413 0.0001735 ***
Stim:Task    4   0.760 0.18988  4.1547 0.0023424 ** 
Residuals 2472 112.977 0.04570                      
---
Signif. codes:  0 ‘***’ 0.001 ‘**’ 0.01 ‘*’ 0.05 ‘.’ 0.1 ‘ ’ 1
```


```
m1 <- lm(formula = Speed_norm ~ Stim*Task*Block, data=df_epfl)
summary(m1)
```


```
Call:
lm(formula = Speed_norm ~ Stim * Task * Block, data = df_epfl)

Residuals:
     Min       1Q   Median       3Q      Max 
-0.61913 -0.13756 -0.00938  0.11579  1.78341 

Coefficients:
                                Estimate Std. Error t value Pr(>|t|)    
(Intercept)                    1.0409179  0.0280447  37.116   <2e-16 ***
StimGPre-TPar                 -0.0689104  0.0396612  -1.737   0.0824 .  
StimTPre-GPar                 -0.0339654  0.0396612  -0.856   0.3919    
StimTpar-GBst                 -0.0965955  0.0413740  -2.335   0.0196 *  
StimTpre-GBst                 -0.0401059  0.0407480  -0.984   0.3251    
Task3back                     -0.0483669  0.0396612  -1.220   0.2228    
Block                         -0.0073038  0.0035333  -2.067   0.0388 *  
StimGPre-TPar:Task3back        0.0499231  0.0560895   0.890   0.3735    
StimTPre-GPar:Task3back        0.0346232  0.0560955   0.617   0.5371    
StimTpar-GBst:Task3back        0.0776297  0.0580708   1.337   0.1814    
StimTpre-GBst:Task3back        0.0890318  0.0576264   1.545   0.1225    
StimGPre-TPar:Block           -0.0037634  0.0049968  -0.753   0.4514    
StimTPre-GPar:Block            0.0026058  0.0049968   0.521   0.6021    
StimTpar-GBst:Block            0.0006805  0.0052126   0.131   0.8961    
StimTpre-GBst:Block            0.0037949  0.0051338   0.739   0.4599    
Task3back:Block                0.0087311  0.0049968   1.747   0.0807 .  
StimGPre-TPar:Task3back:Block  0.0013434  0.0070666   0.190   0.8492    
StimTPre-GPar:Task3back:Block -0.0101728  0.0070666  -1.440   0.1501    
StimTpar-GBst:Task3back:Block -0.0058491  0.0073162  -0.799   0.4241    
StimTpre-GBst:Task3back:Block -0.0066401  0.0072602  -0.915   0.3605    
---
Signif. codes:  0 ‘***’ 0.001 ‘**’ 0.01 ‘*’ 0.05 ‘.’ 0.1 ‘ ’ 1

Residual standard error: 0.2132 on 2462 degrees of freedom
  (118 observations deleted due to missingness)
Multiple R-squared:  0.04375,   Adjusted R-squared:  0.03637 
F-statistic: 5.928 on 19 and 2462 DF,  p-value: 6.409e-15
```


```
anova(m1)
```


```
Analysis of Variance Table

Response: Speed_norm
                  Df  Sum Sq Mean Sq F value    Pr(>F)    
Stim               4   2.616 0.65390 14.3896 1.287e-11 ***
Task               1   0.646 0.64629 14.2221 0.0001663 ***
Block              1   0.679 0.67893 14.9404 0.0001138 ***
Stim:Task          4   0.760 0.18988  4.1785 0.0022459 ** 
Stim:Block         4   0.071 0.01763  0.3879 0.8174219    
Task:Block         1   0.181 0.18059  3.9740 0.0463170 *  
Stim:Task:Block    4   0.167 0.04176  0.9189 0.4518587    
Residuals       2462 111.880 0.04544                      
---
Signif. codes:  0 ‘***’ 0.001 ‘**’ 0.01 ‘*’ 0.05 ‘.’ 0.1 ‘ ’ 1
```


Compare the models


```
anova(m0, m1)
```


```
Analysis of Variance Table

Model 1: Speed_norm ~ Stim * Task
Model 2: Speed_norm ~ Stim * Task * Block
  Res.Df    RSS Df Sum of Sq      F   Pr(>F)   
1   2472 112.98                                
2   2462 111.88 10    1.0971 2.4142 0.007434 **
---
Signif. codes:  0 ‘***’ 0.001 ‘**’ 0.01 ‘*’ 0.05 ‘.’ 0.1 ‘ ’ 1
```


### Note:

The model including the blocks significantly improves the proportion
of the data explained by the model. We will now assess whether the
addition of random effects improves the linear model.

Include random effects Random intercept per subject


```
m2 <- lmer(formula = Speed_norm ~ Stim*Task*Block + (1 | ID), data=df_epfl)
summary(m2)
```


```
Linear mixed model fit by REML. t-tests use Satterthwaite's method ['lmerModLmerTest']
Formula: Speed_norm ~ Stim * Task * Block + (1 | ID)
   Data: df_epfl

REML criterion at convergence: -537.8

Scaled residuals: 
    Min      1Q  Median      3Q     Max 
-2.7053 -0.6364 -0.0674  0.5098  8.3717 

Random effects:
 Groups   Name        Variance Std.Dev.
 ID       (Intercept) 0.001578 0.03972 
 Residual             0.043944 0.20963 
Number of obs: 2482, groups:  ID, 20

Fixed effects:
                                Estimate Std. Error         df t value Pr(>|t|)    
(Intercept)                    1.041e+00  2.897e-02  9.766e+02  35.927   <2e-16 ***
StimGPre-TPar                 -6.891e-02  3.900e-02  2.443e+03  -1.767   0.0774 .  
StimTPre-GPar                 -3.397e-02  3.900e-02  2.443e+03  -0.871   0.3839    
StimTpar-GBst                 -9.489e-02  4.073e-02  2.446e+03  -2.330   0.0199 *  
StimTpre-GBst                 -4.022e-02  4.010e-02  2.445e+03  -1.003   0.3160    
Task3back                     -4.837e-02  3.900e-02  2.443e+03  -1.240   0.2150    
Block                         -7.304e-03  3.475e-03  2.443e+03  -2.102   0.0356 *  
StimGPre-TPar:Task3back        4.992e-02  5.516e-02  2.443e+03   0.905   0.3655    
StimTPre-GPar:Task3back        3.442e-02  5.516e-02  2.443e+03   0.624   0.5327    
StimTpar-GBst:Task3back        7.581e-02  5.711e-02  2.443e+03   1.327   0.1845    
StimTpre-GBst:Task3back        8.903e-02  5.667e-02  2.443e+03   1.571   0.1163    
StimGPre-TPar:Block           -3.763e-03  4.914e-03  2.443e+03  -0.766   0.4438    
StimTPre-GPar:Block            2.606e-03  4.914e-03  2.443e+03   0.530   0.5959    
StimTpar-GBst:Block            6.805e-04  5.126e-03  2.443e+03   0.133   0.8944    
StimTpre-GBst:Block            3.795e-03  5.048e-03  2.443e+03   0.752   0.4523    
Task3back:Block                8.731e-03  4.914e-03  2.443e+03   1.777   0.0757 .  
StimGPre-TPar:Task3back:Block  1.343e-03  6.949e-03  2.443e+03   0.193   0.8467    
StimTPre-GPar:Task3back:Block -1.017e-02  6.949e-03  2.443e+03  -1.464   0.1434    
StimTpar-GBst:Task3back:Block -5.849e-03  7.195e-03  2.443e+03  -0.813   0.4163    
StimTpre-GBst:Task3back:Block -6.640e-03  7.140e-03  2.443e+03  -0.930   0.3524    
---
Signif. codes:  0 ‘***’ 0.001 ‘**’ 0.01 ‘*’ 0.05 ‘.’ 0.1 ‘ ’ 1
```


```
Correlation matrix not shown by default, as p = 20 > 12.
Use print(x, correlation=TRUE)  or
    vcov(x)        if you need it
```


```
anova(m2)
```


```
Type III Analysis of Variance Table with Satterthwaite's method
                 Sum Sq Mean Sq NumDF  DenDF F value    Pr(>F)    
Stim            0.31019 0.07755     4 2445.5  1.7647   0.13319    
Task            0.00030 0.00030     1 2443.1  0.0068   0.93443    
Block           0.67251 0.67251     1 2442.9 15.3039 9.402e-05 ***
Stim:Task       0.13528 0.03382     4 2443.1  0.7696   0.54490    
Stim:Block      0.07090 0.01772     4 2442.9  0.4033   0.80637    
Task:Block      0.17280 0.17280     1 2442.9  3.9323   0.04748 *  
Stim:Task:Block 0.16703 0.04176     4 2442.9  0.9503   0.43380    
---
Signif. codes:  0 ‘***’ 0.001 ‘**’ 0.01 ‘*’ 0.05 ‘.’ 0.1 ‘ ’ 1
```


When adding a random intercept for each subject, there is no longer a
significant effect of of stimulation; the only relevant difference that
remains is a difference in slope among the tasks. We will now compare
the models to see which one is a better fit.


```
AIC(m1, m2)
```


```
BIC(m1, m2)
```

### Note:

The model including the random intercept does not improve the amount
of variance explained by the model.


```
m3 <- lmer(formula = Speed_norm ~ Stim*Task*Block + (1 + Block|ID), data=df_epfl)
```


```
boundary (singular) fit: see help('isSingular')
```


```
summary(m3)
```


```
Linear mixed model fit by REML. t-tests use Satterthwaite's method ['lmerModLmerTest']
Formula: Speed_norm ~ Stim * Task * Block + (1 + Block | ID)
   Data: df_epfl

REML criterion at convergence: -544.5

Scaled residuals: 
    Min      1Q  Median      3Q     Max 
-2.7209 -0.6409 -0.0537  0.5058  8.3607 

Random effects:
 Groups   Name        Variance  Std.Dev. Corr
 ID       (Intercept) 3.245e-04 0.018014     
          Block       9.887e-06 0.003144 1.00
 Residual             4.380e-02 0.209276     
Number of obs: 2482, groups:  ID, 20

Fixed effects:
                                Estimate Std. Error         df t value Pr(>|t|)    
(Intercept)                    1.041e+00  2.783e-02  1.513e+03  37.409   <2e-16 ***
StimGPre-TPar                 -6.891e-02  3.894e-02  2.443e+03  -1.770   0.0769 .  
StimTPre-GPar                 -3.397e-02  3.894e-02  2.443e+03  -0.872   0.3831    
StimTpar-GBst                 -9.593e-02  4.063e-02  2.443e+03  -2.361   0.0183 *  
StimTpre-GBst                 -4.026e-02  4.001e-02  2.443e+03  -1.006   0.3143    
Task3back                     -4.837e-02  3.894e-02  2.443e+03  -1.242   0.2143    
Block                         -7.304e-03  3.539e-03  1.091e+03  -2.064   0.0393 *  
StimGPre-TPar:Task3back        4.992e-02  5.506e-02  2.443e+03   0.907   0.3647    
StimTPre-GPar:Task3back        3.441e-02  5.507e-02  2.443e+03   0.625   0.5321    
StimTpar-GBst:Task3back        7.681e-02  5.701e-02  2.443e+03   1.347   0.1780    
StimTpre-GBst:Task3back        8.903e-02  5.657e-02  2.443e+03   1.574   0.1157    
StimGPre-TPar:Block           -3.763e-03  4.906e-03  2.443e+03  -0.767   0.4430    
StimTPre-GPar:Block            2.606e-03  4.906e-03  2.443e+03   0.531   0.5953    
StimTpar-GBst:Block            7.960e-04  5.119e-03  2.443e+03   0.155   0.8765    
StimTpre-GBst:Block            3.768e-03  5.041e-03  2.443e+03   0.747   0.4549    
Task3back:Block                8.731e-03  4.906e-03  2.443e+03   1.780   0.0752 .  
StimGPre-TPar:Task3back:Block  1.343e-03  6.937e-03  2.443e+03   0.194   0.8465    
StimTPre-GPar:Task3back:Block -1.017e-02  6.937e-03  2.443e+03  -1.466   0.1427    
StimTpar-GBst:Task3back:Block -5.992e-03  7.183e-03  2.443e+03  -0.834   0.4042    
StimTpre-GBst:Task3back:Block -6.640e-03  7.128e-03  2.443e+03  -0.932   0.3516    
---
Signif. codes:  0 ‘***’ 0.001 ‘**’ 0.01 ‘*’ 0.05 ‘.’ 0.1 ‘ ’ 1
```


```
Correlation matrix not shown by default, as p = 20 > 12.
Use print(x, correlation=TRUE)  or
    vcov(x)        if you need it
```


```
optimizer (nloptwrap) convergence code: 0 (OK)
boundary (singular) fit: see help('isSingular')
```


```
anova(m3)
```


```
Type III Analysis of Variance Table with Satterthwaite's method
                 Sum Sq Mean Sq NumDF   DenDF F value   Pr(>F)   
Stim            0.31384 0.07846     4 2442.43  1.7915 0.127750   
Task            0.00038 0.00038     1 2442.93  0.0087 0.925520   
Block           0.48216 0.48216     1   35.72 11.0092 0.002093 **
Stim:Task       0.13670 0.03417     4 2442.92  0.7803 0.537871   
Stim:Block      0.06976 0.01744     4 2442.13  0.3982 0.810041   
Task:Block      0.17059 0.17059     1 2442.95  3.8951 0.048541 * 
Stim:Task:Block 0.16782 0.04195     4 2442.95  0.9580 0.429447   
---
Signif. codes:  0 ‘***’ 0.001 ‘**’ 0.01 ‘*’ 0.05 ‘.’ 0.1 ‘ ’ 1
```


```
AIC(m1, m3)
```


```
BIC(m1, m3)
```

### Note:

The model including a random slope for individual is worse than the
linear model without any random effects, so that’s the one we will
keep.

### Check if adding a random intercept for the days improves the model


```
m4 <- lmer(formula = Speed_norm ~ Stim*Task*Block + (1 + (1|ID) + (1|Day)), data=df_epfl)
summary(m4)
```


```
Linear mixed model fit by REML. t-tests use Satterthwaite's method ['lmerModLmerTest']
Formula: Speed_norm ~ Stim * Task * Block + (1 + (1 | ID) + (1 | Day))
   Data: df_epfl

REML criterion at convergence: -567.8

Scaled residuals: 
    Min      1Q  Median      3Q     Max 
-2.7281 -0.6500 -0.0731  0.5227  8.2745 

Random effects:
 Groups   Name        Variance Std.Dev.
 ID       (Intercept) 0.001573 0.03966 
 Day      (Intercept) 0.001094 0.03308 
 Residual             0.043271 0.20802 
Number of obs: 2482, groups:  ID, 20; Day, 5

Fixed effects:
                                Estimate Std. Error         df t value Pr(>|t|)    
(Intercept)                    1.043e+00  3.454e-02  2.722e+01  30.197   <2e-16 ***
StimGPre-TPar                 -7.225e-02  3.871e-02  2.440e+03  -1.867   0.0621 .  
StimTPre-GPar                 -3.716e-02  3.880e-02  2.442e+03  -0.958   0.3383    
StimTpar-GBst                 -9.834e-02  5.046e-02  2.004e+01  -1.949   0.0655 .  
StimTpre-GBst                 -4.240e-02  4.996e-02  1.926e+01  -0.849   0.4065    
Task3back                     -4.837e-02  3.870e-02  2.440e+03  -1.250   0.2115    
Block                         -7.304e-03  3.448e-03  2.440e+03  -2.118   0.0342 *  
StimGPre-TPar:Task3back        4.992e-02  5.473e-02  2.440e+03   0.912   0.3618    
StimTPre-GPar:Task3back        3.454e-02  5.474e-02  2.440e+03   0.631   0.5281    
StimTpar-GBst:Task3back        7.708e-02  5.668e-02  2.440e+03   1.360   0.1740    
StimTpre-GBst:Task3back        8.903e-02  5.623e-02  2.440e+03   1.583   0.1135    
StimGPre-TPar:Block           -3.763e-03  4.876e-03  2.440e+03  -0.772   0.4403    
StimTPre-GPar:Block            2.606e-03  4.876e-03  2.440e+03   0.534   0.5931    
StimTpar-GBst:Block            6.805e-04  5.087e-03  2.440e+03   0.134   0.8936    
StimTpre-GBst:Block            3.795e-03  5.010e-03  2.440e+03   0.758   0.4488    
Task3back:Block                8.731e-03  4.876e-03  2.440e+03   1.791   0.0735 .  
StimGPre-TPar:Task3back:Block  1.343e-03  6.896e-03  2.440e+03   0.195   0.8456    
StimTPre-GPar:Task3back:Block -1.017e-02  6.896e-03  2.440e+03  -1.475   0.1403    
StimTpar-GBst:Task3back:Block -5.849e-03  7.139e-03  2.440e+03  -0.819   0.4127    
StimTpre-GBst:Task3back:Block -6.640e-03  7.085e-03  2.440e+03  -0.937   0.3487    
---
Signif. codes:  0 ‘***’ 0.001 ‘**’ 0.01 ‘*’ 0.05 ‘.’ 0.1 ‘ ’ 1
```


```
Correlation matrix not shown by default, as p = 20 > 12.
Use print(x, correlation=TRUE)  or
    vcov(x)        if you need it
```


```
anova(m4)
```


```
Type III Analysis of Variance Table with Satterthwaite's method
                 Sum Sq Mean Sq NumDF   DenDF F value    Pr(>F)    
Stim            0.32783 0.08196     4   30.23  1.8940   0.13714    
Task            0.00042 0.00042     1 2440.14  0.0097   0.92157    
Block           0.67251 0.67251     1 2439.98 15.5418 8.297e-05 ***
Stim:Task       0.13696 0.03424     4 2440.13  0.7913   0.53069    
Stim:Block      0.07090 0.01772     4 2439.98  0.4096   0.80185    
Task:Block      0.17280 0.17280     1 2439.98  3.9935   0.04579 *  
Stim:Task:Block 0.16703 0.04176     4 2439.98  0.9650   0.42547    
---
Signif. codes:  0 ‘***’ 0.001 ‘**’ 0.01 ‘*’ 0.05 ‘.’ 0.1 ‘ ’ 1
```


```
AIC(m1, m4)
```


```
BIC(m1, m4)
```


The inclussion of random effects is in this case not justified, so we
will keep a regular linear model.


```
m_speed_norm <- lm(formula = Speed_norm ~ Stim*Task*Block, data=df_epfl)
summary(m_speed_norm)
```


```
Call:
lm(formula = Speed_norm ~ Stim * Task * Block, data = df_epfl)

Residuals:
     Min       1Q   Median       3Q      Max 
-0.61913 -0.13756 -0.00938  0.11579  1.78341 

Coefficients:
                                Estimate Std. Error t value Pr(>|t|)    
(Intercept)                    1.0409179  0.0280447  37.116   <2e-16 ***
StimGPre-TPar                 -0.0689104  0.0396612  -1.737   0.0824 .  
StimTPre-GPar                 -0.0339654  0.0396612  -0.856   0.3919    
StimTpar-GBst                 -0.0965955  0.0413740  -2.335   0.0196 *  
StimTpre-GBst                 -0.0401059  0.0407480  -0.984   0.3251    
Task3back                     -0.0483669  0.0396612  -1.220   0.2228    
Block                         -0.0073038  0.0035333  -2.067   0.0388 *  
StimGPre-TPar:Task3back        0.0499231  0.0560895   0.890   0.3735    
StimTPre-GPar:Task3back        0.0346232  0.0560955   0.617   0.5371    
StimTpar-GBst:Task3back        0.0776297  0.0580708   1.337   0.1814    
StimTpre-GBst:Task3back        0.0890318  0.0576264   1.545   0.1225    
StimGPre-TPar:Block           -0.0037634  0.0049968  -0.753   0.4514    
StimTPre-GPar:Block            0.0026058  0.0049968   0.521   0.6021    
StimTpar-GBst:Block            0.0006805  0.0052126   0.131   0.8961    
StimTpre-GBst:Block            0.0037949  0.0051338   0.739   0.4599    
Task3back:Block                0.0087311  0.0049968   1.747   0.0807 .  
StimGPre-TPar:Task3back:Block  0.0013434  0.0070666   0.190   0.8492    
StimTPre-GPar:Task3back:Block -0.0101728  0.0070666  -1.440   0.1501    
StimTpar-GBst:Task3back:Block -0.0058491  0.0073162  -0.799   0.4241    
StimTpre-GBst:Task3back:Block -0.0066401  0.0072602  -0.915   0.3605    
---
Signif. codes:  0 ‘***’ 0.001 ‘**’ 0.01 ‘*’ 0.05 ‘.’ 0.1 ‘ ’ 1

Residual standard error: 0.2132 on 2462 degrees of freedom
  (118 observations deleted due to missingness)
Multiple R-squared:  0.04375,   Adjusted R-squared:  0.03637 
F-statistic: 5.928 on 19 and 2462 DF,  p-value: 6.409e-15
```


```
anova(m_speed_norm)
```


```
Analysis of Variance Table

Response: Speed_norm
                  Df  Sum Sq Mean Sq F value    Pr(>F)    
Stim               4   2.616 0.65390 14.3896 1.287e-11 ***
Task               1   0.646 0.64629 14.2221 0.0001663 ***
Block              1   0.679 0.67893 14.9404 0.0001138 ***
Stim:Task          4   0.760 0.18988  4.1785 0.0022459 ** 
Stim:Block         4   0.071 0.01763  0.3879 0.8174219    
Task:Block         1   0.181 0.18059  3.9740 0.0463170 *  
Stim:Task:Block    4   0.167 0.04176  0.9189 0.4518587    
Residuals       2462 111.880 0.04544                      
---
Signif. codes:  0 ‘***’ 0.001 ‘**’ 0.01 ‘*’ 0.05 ‘.’ 0.1 ‘ ’ 1
```


```
eta_squared(m_speed_norm)
```


```
# Effect Size for ANOVA (Type I)

Parameter       | Eta2 (partial) |       95% CI
-----------------------------------------------
Stim            |           0.02 | [0.01, 1.00]
Task            |       5.74e-03 | [0.00, 1.00]
Block           |       6.03e-03 | [0.00, 1.00]
Stim:Task       |       6.74e-03 | [0.00, 1.00]
Stim:Block      |       6.30e-04 | [0.00, 1.00]
Task:Block      |       1.61e-03 | [0.00, 1.00]
Stim:Task:Block |       1.49e-03 | [0.00, 1.00]

- One-sided CIs: upper bound fixed at [1.00].
```


The model shows a significant effect of stimulation.


```
my_model <- m_speed_norm
level_1 <- "Stim"

my_model.compare <- emmeans(my_model, level_1, by=c("Block", "Task"), at = list(Block = c(7, 13)))
my_model.compare.pairs <- pairs(my_model.compare, adjust='tukey')
test(my_model.compare.pairs, side='two-sided')
```


```
Block =  7, Task = 2back:
 contrast                   estimate     SE   df t.ratio p.value
 Placebo - (GPre-TPar)      9.53e-02 0.0187 2462   5.095  <.0001
 Placebo - (TPre-GPar)      1.57e-02 0.0187 2462   0.841  0.9178
 Placebo - (Tpar-GBst)      9.18e-02 0.0195 2462   4.708  <.0001
 Placebo - (Tpre-GBst)      1.35e-02 0.0192 2462   0.705  0.9554
 (GPre-TPar) - (TPre-GPar) -7.95e-02 0.0187 2462  -4.254  0.0002
 (GPre-TPar) - (Tpar-GBst) -3.42e-03 0.0195 2462  -0.175  0.9998
 (GPre-TPar) - (Tpre-GBst) -8.17e-02 0.0192 2462  -4.254  0.0002
 (TPre-GPar) - (Tpar-GBst)  7.61e-02 0.0195 2462   3.902  0.0009
 (TPre-GPar) - (Tpre-GBst) -2.18e-03 0.0192 2462  -0.114  1.0000
 (Tpar-GBst) - (Tpre-GBst) -7.83e-02 0.0200 2462  -3.915  0.0009

Block = 13, Task = 2back:
 contrast                   estimate     SE   df t.ratio p.value
 Placebo - (GPre-TPar)      1.18e-01 0.0353 2462   3.335  0.0077
 Placebo - (TPre-GPar)      9.01e-05 0.0353 2462   0.003  1.0000
 Placebo - (Tpar-GBst)      8.77e-02 0.0369 2462   2.381  0.1209
 Placebo - (Tpre-GBst)     -9.23e-03 0.0363 2462  -0.254  0.9991
 (GPre-TPar) - (TPre-GPar) -1.18e-01 0.0353 2462  -3.332  0.0078
 (GPre-TPar) - (Tpar-GBst) -3.01e-02 0.0369 2462  -0.816  0.9257
 (GPre-TPar) - (Tpre-GBst) -1.27e-01 0.0363 2462  -3.500  0.0043
 (TPre-GPar) - (Tpar-GBst)  8.77e-02 0.0369 2462   2.378  0.1216
 (TPre-GPar) - (Tpre-GBst) -9.32e-03 0.0363 2462  -0.257  0.9990
 (Tpar-GBst) - (Tpre-GBst) -9.70e-02 0.0378 2462  -2.566  0.0770

Block =  7, Task = 3back:
 contrast                   estimate     SE   df t.ratio p.value
 Placebo - (GPre-TPar)      3.59e-02 0.0187 2462   1.922  0.3060
 Placebo - (TPre-GPar)      5.23e-02 0.0187 2462   2.795  0.0417
 Placebo - (Tpar-GBst)      5.51e-02 0.0192 2462   2.871  0.0336
 Placebo - (Tpre-GBst)     -2.90e-02 0.0192 2462  -1.510  0.5559
 (GPre-TPar) - (TPre-GPar)  1.64e-02 0.0187 2462   0.875  0.9060
 (GPre-TPar) - (Tpar-GBst)  1.92e-02 0.0192 2462   1.000  0.8552
 (GPre-TPar) - (Tpre-GBst) -6.49e-02 0.0192 2462  -3.381  0.0066
 (TPre-GPar) - (Tpar-GBst)  2.83e-03 0.0192 2462   0.147  0.9999
 (TPre-GPar) - (Tpre-GBst) -8.13e-02 0.0192 2462  -4.230  0.0002
 (Tpar-GBst) - (Tpre-GBst) -8.42e-02 0.0197 2462  -4.270  0.0002

Block = 13, Task = 3back:
 contrast                   estimate     SE   df t.ratio p.value
 Placebo - (GPre-TPar)      5.04e-02 0.0353 2462   1.428  0.6097
 Placebo - (TPre-GPar)      9.77e-02 0.0353 2462   2.765  0.0454
 Placebo - (Tpar-GBst)      8.62e-02 0.0363 2462   2.373  0.1230
 Placebo - (Tpre-GBst)     -1.19e-02 0.0363 2462  -0.329  0.9975
 (GPre-TPar) - (TPre-GPar)  4.73e-02 0.0353 2462   1.337  0.6678
 (GPre-TPar) - (Tpar-GBst)  3.57e-02 0.0363 2462   0.984  0.8627
 (GPre-TPar) - (Tpre-GBst) -6.24e-02 0.0363 2462  -1.719  0.4226
 (TPre-GPar) - (Tpar-GBst) -1.16e-02 0.0363 2462  -0.318  0.9978
 (TPre-GPar) - (Tpre-GBst) -1.10e-01 0.0363 2462  -3.020  0.0215
 (Tpar-GBst) - (Tpre-GBst) -9.81e-02 0.0372 2462  -2.634  0.0647

P value adjustment: tukey method for comparing a family of 5 estimates
```


```
confint(my_model.compare, calc = c(n = ~.wgt.))
```


```
Block =  7, Task = 2back:
 Stim      emmean     SE   df   n lower.CL upper.CL
 Placebo    0.990 0.0132 2462 260    0.964    1.016
 GPre-TPar  0.895 0.0132 2462 260    0.869    0.920
 TPre-GPar  0.974 0.0132 2462 260    0.948    1.000
 Tpar-GBst  0.898 0.0143 2462 221    0.870    0.926
 Tpre-GBst  0.976 0.0139 2462 234    0.949    1.004

Block = 13, Task = 2back:
 Stim      emmean     SE   df   n lower.CL upper.CL
 Placebo    0.946 0.0250 2462 260    0.897    0.995
 GPre-TPar  0.828 0.0250 2462 260    0.779    0.877
 TPre-GPar  0.946 0.0250 2462 260    0.897    0.995
 Tpar-GBst  0.858 0.0271 2462 221    0.805    0.911
 Tpre-GBst  0.955 0.0263 2462 234    0.904    1.007

Block =  7, Task = 3back:
 Stim      emmean     SE   df   n lower.CL upper.CL
 Placebo    1.003 0.0132 2462 260    0.977    1.028
 GPre-TPar  0.967 0.0132 2462 260    0.941    0.993
 TPre-GPar  0.950 0.0132 2462 259    0.924    0.976
 Tpar-GBst  0.947 0.0139 2462 234    0.920    0.975
 Tpre-GBst  1.032 0.0139 2462 234    1.004    1.059

Block = 13, Task = 3back:
 Stim      emmean     SE   df   n lower.CL upper.CL
 Placebo    1.011 0.0250 2462 260    0.962    1.060
 GPre-TPar  0.961 0.0250 2462 260    0.912    1.010
 TPre-GPar  0.913 0.0250 2462 259    0.864    0.962
 Tpar-GBst  0.925 0.0263 2462 234    0.873    0.977
 Tpre-GBst  1.023 0.0263 2462 234    0.971    1.075

Confidence level used: 0.95
```


```
eff_size(my_model.compare, sigma = sigma(my_model), edf = 23)
```


```
Block =  7, Task = 2back:
 contrast                  effect.size     SE   df lower.CL upper.CL
 Placebo - (GPre-TPar)        0.446841 0.1097 2462   0.2317   0.6619
 Placebo - (TPre-GPar)        0.073766 0.0884 2462  -0.0995   0.2471
 Placebo - (Tpar-GBst)        0.430787 0.1114 2462   0.2124   0.6492
 Placebo - (Tpre-GBst)        0.063524 0.0906 2462  -0.1141   0.2412
 (GPre-TPar) - (TPre-GPar)   -0.373075 0.1035 2462  -0.5761  -0.1701
 (GPre-TPar) - (Tpar-GBst)   -0.016054 0.0915 2462  -0.1955   0.1634
 (GPre-TPar) - (Tpre-GBst)   -0.383317 0.1064 2462  -0.5919  -0.1747
 (TPre-GPar) - (Tpar-GBst)    0.357021 0.1056 2462   0.1500   0.5640
 (TPre-GPar) - (Tpre-GBst)   -0.010242 0.0901 2462  -0.1870   0.1665
 (Tpar-GBst) - (Tpre-GBst)   -0.367264 0.1083 2462  -0.5796  -0.1549

Block = 13, Task = 2back:
 contrast                  effect.size     SE   df lower.CL upper.CL
 Placebo - (GPre-TPar)        0.552767 0.1847 2462   0.1906   0.9150
 Placebo - (TPre-GPar)        0.000423 0.1657 2462  -0.3246   0.3254
 Placebo - (Tpar-GBst)        0.411634 0.1832 2462   0.0523   0.7710
 Placebo - (Tpre-GBst)       -0.043289 0.1704 2462  -0.3775   0.2909
 (GPre-TPar) - (TPre-GPar)   -0.552344 0.1847 2462  -0.9145  -0.1902
 (GPre-TPar) - (Tpar-GBst)   -0.141133 0.1742 2462  -0.4826   0.2004
 (GPre-TPar) - (Tpre-GBst)   -0.596056 0.1916 2462  -0.9718  -0.2203
 (TPre-GPar) - (Tpar-GBst)    0.411211 0.1832 2462   0.0519   0.7705
 (TPre-GPar) - (Tpre-GBst)   -0.043711 0.1704 2462  -0.3779   0.2905
 (Tpar-GBst) - (Tpre-GBst)   -0.454923 0.1895 2462  -0.8266  -0.0833

Block =  7, Task = 3back:
 contrast                  effect.size     SE   df lower.CL upper.CL
 Placebo - (GPre-TPar)        0.168537 0.0912 2462  -0.0102   0.3473
 Placebo - (TPre-GPar)        0.245393 0.0950 2462   0.0592   0.4316
 Placebo - (Tpar-GBst)        0.258691 0.0978 2462   0.0668   0.4506
 Placebo - (Tpre-GBst)       -0.136086 0.0923 2462  -0.3171   0.0449
 (GPre-TPar) - (TPre-GPar)    0.076856 0.0885 2462  -0.0967   0.2504
 (GPre-TPar) - (Tpar-GBst)    0.090154 0.0911 2462  -0.0885   0.2688
 (GPre-TPar) - (Tpre-GBst)   -0.304623 0.1007 2462  -0.5021  -0.1072
 (TPre-GPar) - (Tpar-GBst)    0.013297 0.0902 2462  -0.1636   0.1902
 (TPre-GPar) - (Tpre-GBst)   -0.381479 0.1063 2462  -0.5899  -0.1730
 (Tpar-GBst) - (Tpre-GBst)   -0.394776 0.1092 2462  -0.6090  -0.1805

Block = 13, Task = 3back:
 contrast                  effect.size     SE   df lower.CL upper.CL
 Placebo - (GPre-TPar)        0.236651 0.1694 2462  -0.0955   0.5688
 Placebo - (TPre-GPar)        0.458375 0.1790 2462   0.1073   0.8095
 Placebo - (Tpar-GBst)        0.404166 0.1804 2462   0.0504   0.7579
 Placebo - (Tpre-GBst)       -0.056005 0.1705 2462  -0.3903   0.2783
 (GPre-TPar) - (TPre-GPar)    0.221723 0.1690 2462  -0.1096   0.5531
 (GPre-TPar) - (Tpar-GBst)    0.167515 0.1721 2462  -0.1699   0.5049
 (GPre-TPar) - (Tpre-GBst)   -0.292657 0.1757 2462  -0.6371   0.0518
 (TPre-GPar) - (Tpar-GBst)   -0.054209 0.1705 2462  -0.3886   0.2802
 (TPre-GPar) - (Tpre-GBst)   -0.514380 0.1865 2462  -0.8800  -0.1488
 (Tpar-GBst) - (Tpre-GBst)   -0.460171 0.1874 2462  -0.8277  -0.0926

sigma used for effect sizes: 0.2132 
Confidence level used: 0.95
```


Normalized speed for the 2-back task


```
data_subset <- subset(df_epfl, Task == '2back')
data_subset <- droplevels(data_subset)
levels(data_subset$Task)
```


```
[1] "2back"
```


```
m_speed_norm_2back <- lm(formula = Speed_norm ~ Stim*Block, data=data_subset)
summary(m_speed_norm_2back)
```


```
Call:
lm(formula = Speed_norm ~ Stim * Block, data = data_subset)

Residuals:
     Min       1Q   Median       3Q      Max 
-0.53721 -0.14296 -0.01957  0.11322  1.78341 

Coefficients:
                      Estimate Std. Error t value Pr(>|t|)    
(Intercept)          1.0409179  0.0295819  35.188   <2e-16 ***
StimGPre-TPar       -0.0689104  0.0418351  -1.647   0.0998 .  
StimTPre-GPar       -0.0339654  0.0418351  -0.812   0.4170    
StimTpar-GBst       -0.0965955  0.0436417  -2.213   0.0271 *  
StimTpre-GBst       -0.0401059  0.0429815  -0.933   0.3510    
Block               -0.0073038  0.0037270  -1.960   0.0503 .  
StimGPre-TPar:Block -0.0037634  0.0052707  -0.714   0.4753    
StimTPre-GPar:Block  0.0026058  0.0052707   0.494   0.6211    
StimTpar-GBst:Block  0.0006805  0.0054983   0.124   0.9015    
StimTpre-GBst:Block  0.0037949  0.0054152   0.701   0.4836    
---
Signif. codes:  0 ‘***’ 0.001 ‘**’ 0.01 ‘*’ 0.05 ‘.’ 0.1 ‘ ’ 1

Residual standard error: 0.2249 on 1225 degrees of freedom
  (65 observations deleted due to missingness)
Multiple R-squared:  0.04635,   Adjusted R-squared:  0.03934 
F-statistic: 6.615 on 9 and 1225 DF,  p-value: 2.849e-09
```


```
anova(m_speed_norm_2back)
```


```
Analysis of Variance Table

Response: Speed_norm
             Df Sum Sq Mean Sq F value    Pr(>F)    
Stim          4  2.114 0.52841  10.451 2.514e-08 ***
Block         1  0.778 0.77769  15.381 9.272e-05 ***
Stim:Block    4  0.119 0.02968   0.587    0.6721    
Residuals  1225 61.937 0.05056                      
---
Signif. codes:  0 ‘***’ 0.001 ‘**’ 0.01 ‘*’ 0.05 ‘.’ 0.1 ‘ ’ 1
```


```
eta_squared(m_speed_norm_2back)
```


```
# Effect Size for ANOVA (Type I)

Parameter  | Eta2 (partial) |       95% CI
------------------------------------------
Stim       |           0.03 | [0.02, 1.00]
Block      |           0.01 | [0.00, 1.00]
Stim:Block |       1.91e-03 | [0.00, 1.00]

- One-sided CIs: upper bound fixed at [1.00].
```


```
my_model <- m_speed_norm_2back
level_1 <- "Stim"

my_model.compare <- emmeans(my_model, level_1, by="Block")
my_model.compare.pairs <- pairs(my_model.compare, adjust='tukey')
test(my_model.compare.pairs, side='two-sided')
```


```
Block = 7:
 contrast                  estimate     SE   df t.ratio p.value
 Placebo - (GPre-TPar)      0.09525 0.0197 1225   4.830  <.0001
 Placebo - (TPre-GPar)      0.01572 0.0197 1225   0.797  0.9314
 Placebo - (Tpar-GBst)      0.09183 0.0206 1225   4.464  0.0001
 Placebo - (Tpre-GBst)      0.01354 0.0203 1225   0.668  0.9631
 (GPre-TPar) - (TPre-GPar) -0.07953 0.0197 1225  -4.033  0.0006
 (GPre-TPar) - (Tpar-GBst) -0.00342 0.0206 1225  -0.166  0.9998
 (GPre-TPar) - (Tpre-GBst) -0.08171 0.0203 1225  -4.033  0.0006
 (TPre-GPar) - (Tpar-GBst)  0.07611 0.0206 1225   3.699  0.0021
 (TPre-GPar) - (Tpre-GBst) -0.00218 0.0203 1225  -0.108  1.0000
 (Tpar-GBst) - (Tpre-GBst) -0.07829 0.0211 1225  -3.712  0.0020

P value adjustment: tukey method for comparing a family of 5 estimates
```


```
confint(my_model.compare, calc = c(n = ~.wgt.))
```


```
Block = 7:
 Stim      emmean     SE   df   n lower.CL upper.CL
 Placebo    0.990 0.0139 1225 260    0.962    1.017
 GPre-TPar  0.895 0.0139 1225 260    0.867    0.922
 TPre-GPar  0.974 0.0139 1225 260    0.947    1.001
 Tpar-GBst  0.898 0.0151 1225 221    0.868    0.928
 Tpre-GBst  0.976 0.0147 1225 234    0.947    1.005

Confidence level used: 0.95
```


```
eff_size(my_model.compare, sigma = sigma(my_model), edf = 23)
```


```
Block = 7:
 contrast                  effect.size     SE   df lower.CL upper.CL
 Placebo - (GPre-TPar)         0.42362 0.1077 1225    0.212    0.635
 Placebo - (TPre-GPar)         0.06993 0.0883 1225   -0.103    0.243
 Placebo - (Tpar-GBst)         0.40840 0.1095 1225    0.194    0.623
 Placebo - (Tpre-GBst)         0.06022 0.0905 1225   -0.117    0.238
 (GPre-TPar) - (TPre-GPar)    -0.35369 0.1020 1225   -0.554   -0.154
 (GPre-TPar) - (Tpar-GBst)    -0.01522 0.0915 1225   -0.195    0.164
 (GPre-TPar) - (Tpre-GBst)    -0.36340 0.1048 1225   -0.569   -0.158
 (TPre-GPar) - (Tpar-GBst)     0.33847 0.1042 1225    0.134    0.543
 (TPre-GPar) - (Tpre-GBst)    -0.00971 0.0901 1225   -0.187    0.167
 (Tpar-GBst) - (Tpre-GBst)    -0.34818 0.1069 1225   -0.558   -0.138

sigma used for effect sizes: 0.2249 
Confidence level used: 0.95
```


3-back test


```
data_subset <- subset(df_epfl, Task == '3back')
data_subset <- droplevels(data_subset)
levels(data_subset$Task)
```


```
[1] "3back"
```


```
m_speed_norm_3back <- lm(formula = Speed_norm ~ Stim*Block, data=data_subset)
summary(m_speed_norm_3back)
```


```
Call:
lm(formula = Speed_norm ~ Stim * Block, data = data_subset)

Residuals:
     Min       1Q   Median       3Q      Max 
-0.61913 -0.12868  0.00177  0.11799  0.90431 

Coefficients:
                      Estimate Std. Error t value Pr(>|t|)    
(Intercept)          0.9925509  0.0264346  37.547   <2e-16 ***
StimGPre-TPar       -0.0189872  0.0373841  -0.508    0.612    
StimTPre-GPar        0.0006577  0.0373921   0.018    0.986    
StimTpar-GBst       -0.0189658  0.0384085  -0.494    0.622    
StimTpre-GBst        0.0489259  0.0384085   1.274    0.203    
Block                0.0014273  0.0033304   0.429    0.668    
StimGPre-TPar:Block -0.0024200  0.0047100  -0.514    0.607    
StimTPre-GPar:Block -0.0075670  0.0047100  -1.607    0.108    
StimTpar-GBst:Block -0.0051686  0.0048390  -1.068    0.286    
StimTpre-GBst:Block -0.0028452  0.0048390  -0.588    0.557    
---
Signif. codes:  0 ‘***’ 0.001 ‘**’ 0.01 ‘*’ 0.05 ‘.’ 0.1 ‘ ’ 1

Residual standard error: 0.2009 on 1237 degrees of freedom
  (53 observations deleted due to missingness)
Multiple R-squared:  0.02876,   Adjusted R-squared:  0.02169 
F-statistic:  4.07 on 9 and 1237 DF,  p-value: 3.655e-05
```


```
anova(m_speed_norm_3back)
```


```
Analysis of Variance Table

Response: Speed_norm
             Df Sum Sq Mean Sq F value    Pr(>F)    
Stim          4  1.278 0.31955  7.9147 2.677e-06 ***
Block         1  0.081 0.08121  2.0114    0.1564    
Stim:Block    4  0.119 0.02986  0.7396    0.5650    
Residuals  1237 49.943 0.04037                      
---
Signif. codes:  0 ‘***’ 0.001 ‘**’ 0.01 ‘*’ 0.05 ‘.’ 0.1 ‘ ’ 1
```


```
eta_squared(m_speed_norm_3back)
```


```
# Effect Size for ANOVA (Type I)

Parameter  | Eta2 (partial) |       95% CI
------------------------------------------
Stim       |           0.02 | [0.01, 1.00]
Block      |       1.62e-03 | [0.00, 1.00]
Stim:Block |       2.39e-03 | [0.00, 1.00]

- One-sided CIs: upper bound fixed at [1.00].
```


```
my_model <- m_speed_norm_3back
level_1 <- "Stim"

my_model.compare <- emmeans(my_model, level_1, by="Block")
my_model.compare.pairs <- pairs(my_model.compare, adjust='tukey')
test(my_model.compare.pairs, side='two-sided')
```


```
Block = 7:
 contrast                  estimate     SE   df t.ratio p.value
 Placebo - (GPre-TPar)      0.03593 0.0176 1237   2.039  0.2481
 Placebo - (TPre-GPar)      0.05231 0.0176 1237   2.965  0.0256
 Placebo - (Tpar-GBst)      0.05515 0.0181 1237   3.046  0.0200
 Placebo - (Tpre-GBst)     -0.02901 0.0181 1237  -1.602  0.4962
 (GPre-TPar) - (TPre-GPar)  0.01638 0.0176 1237   0.929  0.8858
 (GPre-TPar) - (Tpar-GBst)  0.01922 0.0181 1237   1.061  0.8262
 (GPre-TPar) - (Tpre-GBst) -0.06494 0.0181 1237  -3.587  0.0032
 (TPre-GPar) - (Tpar-GBst)  0.00283 0.0181 1237   0.156  0.9999
 (TPre-GPar) - (Tpre-GBst) -0.08132 0.0181 1237  -4.487  0.0001
 (Tpar-GBst) - (Tpre-GBst) -0.08416 0.0186 1237  -4.530  0.0001

P value adjustment: tukey method for comparing a family of 5 estimates
```


```
confint(my_model.compare, calc = c(n = ~.wgt.))
```


```
Block = 7:
 Stim      emmean     SE   df   n lower.CL upper.CL
 Placebo    1.003 0.0125 1237 260    0.978    1.027
 GPre-TPar  0.967 0.0125 1237 260    0.942    0.991
 TPre-GPar  0.950 0.0125 1237 259    0.926    0.975
 Tpar-GBst  0.947 0.0131 1237 234    0.922    0.973
 Tpre-GBst  1.032 0.0131 1237 234    1.006    1.057

Confidence level used: 0.95
```


```
eff_size(my_model.compare, sigma = sigma(my_model), edf = 23)
```


```
Block = 7:
 contrast                  effect.size     SE   df  lower.CL upper.CL
 Placebo - (GPre-TPar)          0.1788 0.0916 1237 -0.000871   0.3585
 Placebo - (TPre-GPar)          0.2603 0.0958 1237  0.072362   0.4483
 Placebo - (Tpar-GBst)          0.2744 0.0988 1237  0.080657   0.4682
 Placebo - (Tpre-GBst)         -0.1444 0.0926 1237 -0.326024   0.0373
 (GPre-TPar) - (TPre-GPar)      0.0815 0.0886 1237 -0.092304   0.2554
 (GPre-TPar) - (Tpar-GBst)      0.0956 0.0912 1237 -0.083290   0.2746
 (GPre-TPar) - (Tpre-GBst)     -0.3232 0.1019 1237 -0.523157  -0.1232
 (TPre-GPar) - (Tpar-GBst)      0.0141 0.0902 1237 -0.162885   0.1911
 (TPre-GPar) - (Tpre-GBst)     -0.4047 0.1081 1237 -0.616882  -0.1925
 (Tpar-GBst) - (Tpre-GBst)     -0.4188 0.1112 1237 -0.636939  -0.2007

sigma used for effect sizes: 0.2009 
Confidence level used: 0.95
```

## Statistical analysis relating the estimated induced electric fields and behavior.


```
file_name <- file.path(paste(data_directory, "Behavioral_and_efield_blocks_iCOG_Design_25_04_2023.txt", sep='/'))
df_efields <- read.delim(file_name, header = TRUE, na.strings = "NN")
head(df_efields, 5)
```


```
df_efields$ID <- as.factor(df_efields$ID)
df_efields$School <- as.factor(df_efields$School)
df_efields$Day <- as.factor(df_efields$Day)
df_efields$Task <- as.factor(df_efields$Task)
df_efields$Stim <- as.factor(df_efields$Stim)
df_efields$Acc_u <- as.numeric(df_efields$Acc_u)
df_efields$Speed_u <- as.numeric(df_efields$Speed_u)
df_efields$Acc_m <- as.numeric(df_efields$Acc_m)
df_efields$Acc_n_m <- as.numeric(df_efields$Acc_n_m)
df_efields$Speed_m <- as.numeric(df_efields$Speed_m)
df_efields$Speed_n_m <- as.numeric(df_efields$Speed_n_m)
df_efields$EF_front <- as.numeric(df_efields$EF_front)
df_efields$EF_par <- as.numeric(df_efields$EF_par)
df_efields$EF_mag <- as.numeric(df_efields$EF_mag)
df_efields$Error_front <- as.numeric(df_efields$Error_front)
df_efields$Error_par <- as.numeric(df_efields$Error_par)
```


```
df_ef_epfl <- subset(df_efields, School == 'EPFL')
df_ef_epfl <- droplevels(df_ef_epfl)
#levels(df_ef_epfl$School)
head(df_ef_epfl, 5)
```


```
df_ef_epfl$Stim <- factor(df_ef_epfl$Stim, levels(df_ef_epfl$Stim)[c(2, 1, 5, 3, 4)])
levels(df_ef_epfl$Stim)
```


```
[1] "Placebo"   "GPre-TPar" "TPre-GPar" "Tpar-GBst" "Tpre-GBst"
```


### Note about the analysis

The idea of this analysis is to verify whether there was a
differentiated effect of stimulation resulting from the magnitude of the
electric fields induced at both stimulation sites at an individual
level. The main analysis described above showed significant effects of
stimulation in the normalized data, which depicts relative changes in
performance for each individual. For this reason, we will assess the
effect of the magnitude of the induced electric fields on the change in
performance (i.e., in terms of speed and accuracy) within each training
session. This change in performance is described as the slope of the
line minimizing the error in a least-squared sense, fitted to each
individual.

This data frame contains the magnitude of the estimated electrical
field for each electrode (i.e., frontal and parietal). The electrode
locations were determined by having participants perform the 2-back task
inside the MRI scanner and finding the region were the task-related bold
signal peaked. This regions were then projected into the scalp of each
participant and the resulting coordinates were used to place the
electrodes. In the data frame, the columns labeled as “EF” contain the
magnitude of the electric field estimated for each intended target,
while the columns labeled with “error” include the distance between the
intended target and the location were the maximum electric field was
induced. The column labeled “EF\_mag” contains the magnitude of the
electric field induced on both sites (mag = sqrt(a^2 + b^2)). The column
“EF\_sym” contains the ratio of the electric field induced on the frontal
site to the electric field induced in the parietal site
(EF\_front/EF\_par), and constitutes a measure of how symmetric the
induced fields were. By design and under ideal circumstances, the
induced fields would be identical, but anatomical differences among the
sites often result in different fields being induced.

In the EPFL cohorts, the intensity of the applied current was not the
same on all sessions. In the first three sessions, the applied intensity
was of 2 mA peak-to-peak. The intensity meant to be applied in sessions
4 and 5 was of 4 mA peak-to-peak; however, not all participants could
withstand the tingling sensation at this intensity, which is why for
these participants lower currents were used, adjusted to their tolerance
threshold. The electric field simulations were done according to the
intensity of the applied currents on each day for each participant.

### The relationship between the intensity of the applied electric field on the target and the accuracy


```
data_subset <- subset(df_ef_epfl, Stim != 'Placebo')
data_subset <- droplevels(data_subset)
levels(data_subset$Stim)
```


```
[1] "GPre-TPar" "TPre-GPar" "Tpar-GBst" "Tpre-GBst"
```


We will now choose the model following the same criteria as we
applied to test all the previous aspects.


```
m0 <- lm(formula = Acc_m ~ Task*Stim*EF_front*EF_par, data=data_subset)
summary(m0)
```


```
Call:
lm(formula = Acc_m ~ Task * Stim * EF_front * EF_par, data = data_subset)

Residuals:
      Min        1Q    Median        3Q       Max 
-0.019913 -0.006663 -0.000467  0.004630  0.051126 

Coefficients:
                                          Estimate Std. Error t value Pr(>|t|)  
(Intercept)                              0.0048207  0.0111119   0.434   0.6652  
Task3back                                0.0071470  0.0157146   0.455   0.6501  
StimTPre-GPar                           -0.0133400  0.0157146  -0.849   0.3976  
StimTpar-GBst                            0.0002559  0.0156834   0.016   0.9870  
StimTpre-GBst                            0.0010174  0.0156834   0.065   0.9484  
EF_front                                 0.0661603  0.1320098   0.501   0.6172  
EF_par                                  -0.0310762  0.2062273  -0.151   0.8805  
Task3back:StimTPre-GPar                 -0.0214095  0.0222238  -0.963   0.3373  
Task3back:StimTpar-GBst                 -0.0081436  0.0221797  -0.367   0.7141  
Task3back:StimTpre-GBst                 -0.0067303  0.0221797  -0.303   0.7621  
Task3back:EF_front                      -0.2604781  0.1866900  -1.395   0.1655  
StimTPre-GPar:EF_front                  -0.0276789  0.1866900  -0.148   0.8824  
StimTpar-GBst:EF_front                  -0.0426955  0.1444433  -0.296   0.7681  
StimTpre-GBst:EF_front                  -0.1004683  0.1444433  -0.696   0.4881  
Task3back:EF_par                        -0.1177434  0.2916495  -0.404   0.6871  
StimTPre-GPar:EF_par                     0.4777911  0.2916495   1.638   0.1040  
StimTpar-GBst:EF_par                     0.0139238  0.2552723   0.055   0.9566  
StimTpre-GBst:EF_par                     0.0483089  0.2552723   0.189   0.8502  
EF_front:EF_par                         -0.0403873  1.6354058  -0.025   0.9803  
Task3back:StimTPre-GPar:EF_front         0.4631947  0.2640196   1.754   0.0819 .
Task3back:StimTpar-GBst:EF_front         0.2988942  0.2042737   1.463   0.1460  
Task3back:StimTpre-GBst:EF_front         0.2064798  0.2042737   1.011   0.3141  
Task3back:StimTPre-GPar:EF_par           0.1174745  0.4124547   0.285   0.7763  
Task3back:StimTpar-GBst:EF_par           0.0328692  0.3610095   0.091   0.9276  
Task3back:StimTpre-GBst:EF_par           0.0008542  0.3610095   0.002   0.9981  
Task3back:EF_front:EF_par                2.3359450  2.3128130   1.010   0.3145  
StimTPre-GPar:EF_front:EF_par           -2.1876460  2.3128130  -0.946   0.3461  
StimTpar-GBst:EF_front:EF_par           -0.1206603  1.7013665  -0.071   0.9436  
StimTpre-GBst:EF_front:EF_par            0.0297081  1.7013665   0.017   0.9861  
Task3back:StimTPre-GPar:EF_front:EF_par -3.9733969  3.2708115  -1.215   0.2268  
Task3back:StimTpar-GBst:EF_front:EF_par -2.2067235  2.4060955  -0.917   0.3609  
Task3back:StimTpre-GBst:EF_front:EF_par -1.5901852  2.4060955  -0.661   0.5099  
---
Signif. codes:  0 ‘***’ 0.001 ‘**’ 0.01 ‘*’ 0.05 ‘.’ 0.1 ‘ ’ 1

Residual standard error: 0.0105 on 120 degrees of freedom
Multiple R-squared:  0.2612,    Adjusted R-squared:  0.07039 
F-statistic: 1.369 on 31 and 120 DF,  p-value: 0.1177
```


```
anova(m0)
```


```
Analysis of Variance Table

Response: Acc_m
                           Df    Sum Sq    Mean Sq F value   Pr(>F)   
Task                        1 0.0009872 0.00098717  8.9625 0.003347 **
Stim                        3 0.0004679 0.00015596  1.4160 0.241494   
EF_front                    1 0.0000063 0.00000626  0.0568 0.812036   
EF_par                      1 0.0000179 0.00001793  0.1628 0.687326   
Task:Stim                   3 0.0002492 0.00008308  0.7543 0.521952   
Task:EF_front               1 0.0001985 0.00019845  1.8018 0.182034   
Stim:EF_front               3 0.0001160 0.00003868  0.3512 0.788371   
Task:EF_par                 1 0.0000067 0.00000667  0.0605 0.806108   
Stim:EF_par                 3 0.0007751 0.00025835  2.3455 0.076283 . 
EF_front:EF_par             1 0.0000054 0.00000538  0.0488 0.825520   
Task:Stim:EF_front          3 0.0001878 0.00006260  0.5683 0.636927   
Task:Stim:EF_par            3 0.0003406 0.00011352  1.0307 0.381639   
Task:EF_front:EF_par        1 0.0001005 0.00010051  0.9125 0.341365   
Stim:EF_front:EF_par        3 0.0010045 0.00033485  3.0400 0.031690 * 
Task:Stim:EF_front:EF_par   3 0.0002104 0.00007013  0.6367 0.592777   
Residuals                 120 0.0132174 0.00011015                    
---
Signif. codes:  0 ‘***’ 0.001 ‘**’ 0.01 ‘*’ 0.05 ‘.’ 0.1 ‘ ’ 1
```


```
m1 <- lmer(formula = Acc_m ~ Task*Stim*EF_front*EF_par + (1 | ID), data=data_subset)
summary(m1)
```


```
Linear mixed model fit by REML. t-tests use Satterthwaite's method ['lmerModLmerTest']
Formula: Acc_m ~ Task * Stim * EF_front * EF_par + (1 | ID)
   Data: data_subset

REML criterion at convergence: -854.2

Scaled residuals: 
    Min      1Q  Median      3Q     Max 
-1.8163 -0.6460 -0.0669  0.4568  4.7615 

Random effects:
 Groups   Name        Variance  Std.Dev.
 ID       (Intercept) 4.494e-06 0.00212 
 Residual             1.056e-04 0.01028 
Number of obs: 152, groups:  ID, 20

Fixed effects:
                                          Estimate Std. Error         df t value Pr(>|t|)  
(Intercept)                              5.013e-03  1.109e-02  1.200e+02   0.452   0.6520  
Task3back                                7.147e-03  1.539e-02  1.048e+02   0.464   0.6433  
StimTPre-GPar                           -1.334e-02  1.539e-02  1.048e+02  -0.867   0.3880  
StimTpar-GBst                            5.358e-04  1.549e-02  1.149e+02   0.035   0.9725  
StimTpre-GBst                            1.297e-03  1.549e-02  1.149e+02   0.084   0.9334  
EF_front                                 6.477e-02  1.319e-01  1.195e+02   0.491   0.6242  
EF_par                                  -3.386e-02  2.057e-01  1.200e+02  -0.165   0.8695  
Task3back:StimTPre-GPar                 -2.141e-02  2.176e-02  1.048e+02  -0.984   0.3275  
Task3back:StimTpar-GBst                 -8.144e-03  2.172e-02  1.048e+02  -0.375   0.7084  
Task3back:StimTpre-GBst                 -6.730e-03  2.172e-02  1.048e+02  -0.310   0.7573  
Task3back:EF_front                      -2.605e-01  1.828e-01  1.048e+02  -1.425   0.1572  
StimTPre-GPar:EF_front                  -2.768e-02  1.828e-01  1.048e+02  -0.151   0.8799  
StimTpar-GBst:EF_front                  -4.178e-02  1.425e-01  1.142e+02  -0.293   0.7699  
StimTpre-GBst:EF_front                  -9.955e-02  1.425e-01  1.142e+02  -0.699   0.4861  
Task3back:EF_par                        -1.177e-01  2.856e-01  1.048e+02  -0.412   0.6810  
StimTPre-GPar:EF_par                     4.778e-01  2.856e-01  1.048e+02   1.673   0.0973 .
StimTpar-GBst:EF_par                     8.394e-03  2.528e-01  1.167e+02   0.033   0.9736  
StimTpre-GBst:EF_par                     4.278e-02  2.528e-01  1.167e+02   0.169   0.8659  
EF_front:EF_par                         -2.614e-02  1.632e+00  1.199e+02  -0.016   0.9873  
Task3back:StimTPre-GPar:EF_front         4.632e-01  2.585e-01  1.048e+02   1.792   0.0761 .
Task3back:StimTpar-GBst:EF_front         2.989e-01  2.000e-01  1.048e+02   1.494   0.1381  
Task3back:StimTpre-GBst:EF_front         2.065e-01  2.000e-01  1.048e+02   1.032   0.3043  
Task3back:StimTPre-GPar:EF_par           1.175e-01  4.039e-01  1.048e+02   0.291   0.7717  
Task3back:StimTpar-GBst:EF_par           3.287e-02  3.535e-01  1.048e+02   0.093   0.9261  
Task3back:StimTpre-GBst:EF_par           8.542e-04  3.535e-01  1.048e+02   0.002   0.9981  
Task3back:EF_front:EF_par                2.336e+00  2.265e+00  1.048e+02   1.031   0.3047  
StimTPre-GPar:EF_front:EF_par           -2.188e+00  2.265e+00  1.048e+02  -0.966   0.3363  
StimTpar-GBst:EF_front:EF_par           -1.123e-01  1.688e+00  1.187e+02  -0.066   0.9471  
StimTpre-GBst:EF_front:EF_par            3.809e-02  1.688e+00  1.187e+02   0.023   0.9820  
Task3back:StimTPre-GPar:EF_front:EF_par -3.973e+00  3.203e+00  1.048e+02  -1.241   0.2175  
Task3back:StimTpar-GBst:EF_front:EF_par -2.207e+00  2.356e+00  1.048e+02  -0.937   0.3511  
Task3back:StimTpre-GBst:EF_front:EF_par -1.590e+00  2.356e+00  1.048e+02  -0.675   0.5012  
---
Signif. codes:  0 ‘***’ 0.001 ‘**’ 0.01 ‘*’ 0.05 ‘.’ 0.1 ‘ ’ 1
```


```
Correlation matrix not shown by default, as p = 32 > 12.
Use print(x, correlation=TRUE)  or
    vcov(x)        if you need it
```


```
anova(m1)
```


```
Type III Analysis of Variance Table with Satterthwaite's method
                              Sum Sq    Mean Sq NumDF   DenDF F value  Pr(>F)  
Task                      0.00000663 0.00000663     1 104.770  0.0628 0.80265  
Stim                      0.00065971 0.00021990     3 113.593  2.0823 0.10649  
EF_front                  0.00001186 0.00001186     1  22.697  0.1123 0.74061  
EF_par                    0.00007638 0.00007638     1  38.573  0.7233 0.40033  
Task:Stim                 0.00010745 0.00003582     3 104.770  0.3392 0.79705  
Task:EF_front             0.00000710 0.00000710     1 104.770  0.0672 0.79593  
Stim:EF_front             0.00065201 0.00021734     3 111.613  2.0580 0.10988  
Task:EF_par               0.00004321 0.00004321     1 104.770  0.4092 0.52379  
Stim:EF_par               0.00108752 0.00036251     3 112.063  3.4326 0.01948 *
EF_front:EF_par           0.00007902 0.00007902     1  38.339  0.7483 0.39240  
Task:Stim:EF_front        0.00040978 0.00013659     3 104.770  1.2934 0.28063  
Task:Stim:EF_par          0.00001315 0.00000438     3 104.770  0.0415 0.98868  
Task:EF_front:EF_par      0.00002355 0.00002355     1 104.770  0.2230 0.63773  
Stim:EF_front:EF_par      0.00098929 0.00032976     3 106.201  3.1226 0.02904 *
Task:Stim:EF_front:EF_par 0.00021040 0.00007013     3 104.770  0.6641 0.57595  
---
Signif. codes:  0 ‘***’ 0.001 ‘**’ 0.01 ‘*’ 0.05 ‘.’ 0.1 ‘ ’ 1
```


```
AIC(m0, m1)
```


```
BIC(m0, m1)
```


#### Model choice

The model including random effects does not improve the model, so we
will use the simpler model for this test.


```
m_acc_ef <- lm(formula = Acc_m ~ Task*Stim*EF_front*EF_par, data=data_subset)
summary(m_acc_ef)
```


```
Call:
lm(formula = Acc_m ~ Task * Stim * EF_front * EF_par, data = data_subset)

Residuals:
      Min        1Q    Median        3Q       Max 
-0.019913 -0.006663 -0.000467  0.004630  0.051126 

Coefficients:
                                          Estimate Std. Error t value Pr(>|t|)  
(Intercept)                              0.0048207  0.0111119   0.434   0.6652  
Task3back                                0.0071470  0.0157146   0.455   0.6501  
StimTPre-GPar                           -0.0133400  0.0157146  -0.849   0.3976  
StimTpar-GBst                            0.0002559  0.0156834   0.016   0.9870  
StimTpre-GBst                            0.0010174  0.0156834   0.065   0.9484  
EF_front                                 0.0661603  0.1320098   0.501   0.6172  
EF_par                                  -0.0310762  0.2062273  -0.151   0.8805  
Task3back:StimTPre-GPar                 -0.0214095  0.0222238  -0.963   0.3373  
Task3back:StimTpar-GBst                 -0.0081436  0.0221797  -0.367   0.7141  
Task3back:StimTpre-GBst                 -0.0067303  0.0221797  -0.303   0.7621  
Task3back:EF_front                      -0.2604781  0.1866900  -1.395   0.1655  
StimTPre-GPar:EF_front                  -0.0276789  0.1866900  -0.148   0.8824  
StimTpar-GBst:EF_front                  -0.0426955  0.1444433  -0.296   0.7681  
StimTpre-GBst:EF_front                  -0.1004683  0.1444433  -0.696   0.4881  
Task3back:EF_par                        -0.1177434  0.2916495  -0.404   0.6871  
StimTPre-GPar:EF_par                     0.4777911  0.2916495   1.638   0.1040  
StimTpar-GBst:EF_par                     0.0139238  0.2552723   0.055   0.9566  
StimTpre-GBst:EF_par                     0.0483089  0.2552723   0.189   0.8502  
EF_front:EF_par                         -0.0403873  1.6354058  -0.025   0.9803  
Task3back:StimTPre-GPar:EF_front         0.4631947  0.2640196   1.754   0.0819 .
Task3back:StimTpar-GBst:EF_front         0.2988942  0.2042737   1.463   0.1460  
Task3back:StimTpre-GBst:EF_front         0.2064798  0.2042737   1.011   0.3141  
Task3back:StimTPre-GPar:EF_par           0.1174745  0.4124547   0.285   0.7763  
Task3back:StimTpar-GBst:EF_par           0.0328692  0.3610095   0.091   0.9276  
Task3back:StimTpre-GBst:EF_par           0.0008542  0.3610095   0.002   0.9981  
Task3back:EF_front:EF_par                2.3359450  2.3128130   1.010   0.3145  
StimTPre-GPar:EF_front:EF_par           -2.1876460  2.3128130  -0.946   0.3461  
StimTpar-GBst:EF_front:EF_par           -0.1206603  1.7013665  -0.071   0.9436  
StimTpre-GBst:EF_front:EF_par            0.0297081  1.7013665   0.017   0.9861  
Task3back:StimTPre-GPar:EF_front:EF_par -3.9733969  3.2708115  -1.215   0.2268  
Task3back:StimTpar-GBst:EF_front:EF_par -2.2067235  2.4060955  -0.917   0.3609  
Task3back:StimTpre-GBst:EF_front:EF_par -1.5901852  2.4060955  -0.661   0.5099  
---
Signif. codes:  0 ‘***’ 0.001 ‘**’ 0.01 ‘*’ 0.05 ‘.’ 0.1 ‘ ’ 1

Residual standard error: 0.0105 on 120 degrees of freedom
Multiple R-squared:  0.2612,    Adjusted R-squared:  0.07039 
F-statistic: 1.369 on 31 and 120 DF,  p-value: 0.1177
```


```
anova(m_acc_ef)
```


```
Analysis of Variance Table

Response: Acc_m
                           Df    Sum Sq    Mean Sq F value   Pr(>F)   
Task                        1 0.0009872 0.00098717  8.9625 0.003347 **
Stim                        3 0.0004679 0.00015596  1.4160 0.241494   
EF_front                    1 0.0000063 0.00000626  0.0568 0.812036   
EF_par                      1 0.0000179 0.00001793  0.1628 0.687326   
Task:Stim                   3 0.0002492 0.00008308  0.7543 0.521952   
Task:EF_front               1 0.0001985 0.00019845  1.8018 0.182034   
Stim:EF_front               3 0.0001160 0.00003868  0.3512 0.788371   
Task:EF_par                 1 0.0000067 0.00000667  0.0605 0.806108   
Stim:EF_par                 3 0.0007751 0.00025835  2.3455 0.076283 . 
EF_front:EF_par             1 0.0000054 0.00000538  0.0488 0.825520   
Task:Stim:EF_front          3 0.0001878 0.00006260  0.5683 0.636927   
Task:Stim:EF_par            3 0.0003406 0.00011352  1.0307 0.381639   
Task:EF_front:EF_par        1 0.0001005 0.00010051  0.9125 0.341365   
Stim:EF_front:EF_par        3 0.0010045 0.00033485  3.0400 0.031690 * 
Task:Stim:EF_front:EF_par   3 0.0002104 0.00007013  0.6367 0.592777   
Residuals                 120 0.0132174 0.00011015                    
---
Signif. codes:  0 ‘***’ 0.001 ‘**’ 0.01 ‘*’ 0.05 ‘.’ 0.1 ‘ ’ 1
```


```
eta_squared(m_acc_ef)
```


```
# Effect Size for ANOVA (Type I)

Parameter                 | Eta2 (partial) |       95% CI
---------------------------------------------------------
Task                      |           0.07 | [0.01, 1.00]
Stim                      |           0.03 | [0.00, 1.00]
EF_front                  |       4.73e-04 | [0.00, 1.00]
EF_par                    |       1.35e-03 | [0.00, 1.00]
Task:Stim                 |           0.02 | [0.00, 1.00]
Task:EF_front             |           0.01 | [0.00, 1.00]
Stim:EF_front             |       8.70e-03 | [0.00, 1.00]
Task:EF_par               |       5.04e-04 | [0.00, 1.00]
Stim:EF_par               |           0.06 | [0.00, 1.00]
EF_front:EF_par           |       4.07e-04 | [0.00, 1.00]
Task:Stim:EF_front        |           0.01 | [0.00, 1.00]
Task:Stim:EF_par          |           0.03 | [0.00, 1.00]
Task:EF_front:EF_par      |       7.55e-03 | [0.00, 1.00]
Stim:EF_front:EF_par      |           0.07 | [0.00, 1.00]
Task:Stim:EF_front:EF_par |           0.02 | [0.00, 1.00]

- One-sided CIs: upper bound fixed at [1.00].
```

#### 2-back task

As there is a significant difference among the tasks (as in every
aspect tested previously), we will fit separate models to each task


```
data_subset <- subset(df_ef_epfl, Task == '2back' & Stim != 'Placebo')
data_subset <- droplevels(data_subset)
levels(data_subset$Task)
```


```
[1] "2back"
```


```
levels(data_subset$Stim)
```


```
[1] "GPre-TPar" "TPre-GPar" "Tpar-GBst" "Tpre-GBst"
```


```
m_acc_ef_2back <- lm(formula = Acc_m ~ Stim*EF_front*EF_par, data=data_subset)
summary(m_acc_ef_2back)
```


```
Call:
lm(formula = Acc_m ~ Stim * EF_front * EF_par, data = data_subset)

Residuals:
      Min        1Q    Median        3Q       Max 
-0.019913 -0.007919 -0.000228  0.004630  0.051126 

Coefficients:
                                Estimate Std. Error t value Pr(>|t|)
(Intercept)                    0.0048207  0.0123706   0.390    0.698
StimTPre-GPar                 -0.0133400  0.0174946  -0.763    0.449
StimTpar-GBst                  0.0002559  0.0174599   0.015    0.988
StimTpre-GBst                  0.0010174  0.0174599   0.058    0.954
EF_front                       0.0661603  0.1469628   0.450    0.654
EF_par                        -0.0310762  0.2295872  -0.135    0.893
StimTPre-GPar:EF_front        -0.0276789  0.2078369  -0.133    0.894
StimTpar-GBst:EF_front        -0.0426955  0.1608048  -0.266    0.792
StimTpre-GBst:EF_front        -0.1004683  0.1608048  -0.625    0.534
StimTPre-GPar:EF_par           0.4777911  0.3246853   1.472    0.146
StimTpar-GBst:EF_par           0.0139238  0.2841876   0.049    0.961
StimTpre-GBst:EF_par           0.0483089  0.2841876   0.170    0.866
EF_front:EF_par               -0.0403873  1.8206521  -0.022    0.982
StimTPre-GPar:EF_front:EF_par -2.1876460  2.5747909  -0.850    0.399
StimTpar-GBst:EF_front:EF_par -0.1206603  1.8940844  -0.064    0.949
StimTpre-GBst:EF_front:EF_par  0.0297081  1.8940844   0.016    0.988

Residual standard error: 0.01168 on 60 degrees of freedom
Multiple R-squared:  0.1642,    Adjusted R-squared:  -0.04478 
F-statistic: 0.7857 on 15 and 60 DF,  p-value: 0.6878
```


```
anova(m_acc_ef_2back)
```


```
Analysis of Variance Table

Response: Acc_m
                     Df    Sum Sq    Mean Sq F value Pr(>F)
Stim                  3 0.0005396 1.7986e-04  1.3176 0.2770
EF_front              1 0.0001376 1.3759e-04  1.0079 0.3194
EF_par                1 0.0000014 1.3660e-06  0.0100 0.9207
Stim:EF_front         3 0.0002146 7.1541e-05  0.5241 0.6674
Stim:EF_par           3 0.0004982 1.6606e-04  1.2164 0.3116
EF_front:EF_par       1 0.0000297 2.9697e-05  0.2175 0.6426
Stim:EF_front:EF_par  3 0.0001878 6.2614e-05  0.4587 0.7122
Residuals            60 0.0081907 1.3651e-04
```


```
data_subset <- subset(df_ef_epfl, Task == '3back' & Stim != 'Placebo')
data_subset <- droplevels(data_subset)
levels(data_subset$Task)
```


```
[1] "3back"
```


```
levels(data_subset$Stim)
```


```
[1] "GPre-TPar" "TPre-GPar" "Tpar-GBst" "Tpre-GBst"
```


```
m_acc_ef_3back <- lm(formula = Acc_m ~ Stim*EF_front*EF_par, data=data_subset)
summary(m_acc_ef_3back)
```


```
Call:
lm(formula = Acc_m ~ Stim * EF_front * EF_par, data = data_subset)

Residuals:
       Min         1Q     Median         3Q        Max 
-0.0166307 -0.0061036 -0.0006752  0.0047779  0.0177394 

Coefficients:
                               Estimate Std. Error t value Pr(>|t|)   
(Intercept)                    0.011968   0.009691   1.235  0.22168   
StimTPre-GPar                 -0.034749   0.013705  -2.535  0.01385 * 
StimTpar-GBst                 -0.007888   0.013678  -0.577  0.56632   
StimTpre-GBst                 -0.005713   0.013678  -0.418  0.67768   
EF_front                      -0.194318   0.115131  -1.688  0.09664 . 
EF_par                        -0.148820   0.179859  -0.827  0.41127   
StimTPre-GPar:EF_front         0.435516   0.162819   2.675  0.00962 **
StimTpar-GBst:EF_front         0.256199   0.125975   2.034  0.04641 * 
StimTpre-GBst:EF_front         0.106012   0.125975   0.842  0.40339   
StimTPre-GPar:EF_par           0.595266   0.254359   2.340  0.02261 * 
StimTpar-GBst:EF_par           0.046793   0.222633   0.210  0.83424   
StimTpre-GBst:EF_par           0.049163   0.222633   0.221  0.82598   
EF_front:EF_par                2.295558   1.426299   1.609  0.11277   
StimTPre-GPar:EF_front:EF_par -6.161043   2.017092  -3.054  0.00336 **
StimTpar-GBst:EF_front:EF_par -2.327384   1.483826  -1.569  0.12202   
StimTpre-GBst:EF_front:EF_par -1.560477   1.483826  -1.052  0.29717   
---
Signif. codes:  0 ‘***’ 0.001 ‘**’ 0.01 ‘*’ 0.05 ‘.’ 0.1 ‘ ’ 1

Residual standard error: 0.009153 on 60 degrees of freedom
Multiple R-squared:  0.2925,    Adjusted R-squared:  0.1156 
F-statistic: 1.653 on 15 and 60 DF,  p-value: 0.08642
```


```
anova(m_acc_ef_3back)
```


```
Analysis of Variance Table

Response: Acc_m
                     Df    Sum Sq    Mean Sq F value  Pr(>F)  
Stim                  3 0.0001775 0.00005918  0.7064 0.55196  
EF_front              1 0.0000671 0.00006712  0.8012 0.37433  
EF_par                1 0.0000232 0.00002323  0.2773 0.60044  
Stim:EF_front         3 0.0000892 0.00002974  0.3549 0.78572  
Stim:EF_par           3 0.0006175 0.00020582  2.4567 0.07163 .
EF_front:EF_par       1 0.0000762 0.00007619  0.9094 0.34410  
Stim:EF_front:EF_par  3 0.0010271 0.00034236  4.0865 0.01047 *
Residuals            60 0.0050267 0.00008378                  
---
Signif. codes:  0 ‘***’ 0.001 ‘**’ 0.01 ‘*’ 0.05 ‘.’ 0.1 ‘ ’ 1
```


```
eta_squared(m_acc_ef_3back)
```


```
# Effect Size for ANOVA (Type I)

Parameter            | Eta2 (partial) |       95% CI
----------------------------------------------------
Stim                 |           0.03 | [0.00, 1.00]
EF_front             |           0.01 | [0.00, 1.00]
EF_par               |       4.60e-03 | [0.00, 1.00]
Stim:EF_front        |           0.02 | [0.00, 1.00]
Stim:EF_par          |           0.11 | [0.00, 1.00]
EF_front:EF_par      |           0.01 | [0.00, 1.00]
Stim:EF_front:EF_par |           0.17 | [0.03, 1.00]

- One-sided CIs: upper bound fixed at [1.00].
```

#### Explore the triple interactions

The interpretation of triple interactions is complicated, but we will
try to provide a visualization for each group.


```
n_rows = 100
choose_model <- m_acc_ef_3back
stim_list <- levels(data_subset$Stim)
i_start = 0
i_stop = 0.3

columns = c("Stim","Task","EF_front", "EF_par") 
df_test = data.frame(matrix(nrow = n_rows, ncol = length(columns))) 
colnames(df_test) = columns

columns = c("Stim","Task","EF_front", "EF_par") 
df_temp = data.frame(matrix(nrow = n_rows, ncol = length(columns))) 
colnames(df_temp) = columns

for (s in stim_list){
  for (t in c("3back")){
    if (s == stim_list[1] & t == '3back'){
    df_test$Stim <- rep(s, n_rows)
    df_test$EF_front <- rep(seq(i_start, i_stop, length.out = 10), 10)
    df_test$EF_par <- rep(seq(i_start, i_stop, length.out = 10), each=10)
    #df_test$Task <- sample(c('2back', '3back'), 100, replace=TRUE)
    df_test$Task <- rep(t, 100)
    }
    else{
    df_temp$Stim <- rep(s, n_rows)
    df_temp$EF_front <- rep(seq(i_start, i_stop, length.out = 10), 10)
    df_temp$EF_par <- rep(seq(i_start, i_stop, length.out = 10), each=10)
    #df_temp$Task <- sample(c('2back', '3back'), 100, replace=TRUE)
    df_temp$Task <- rep(t, 100)
    df_test <- rbind(df_test, df_temp)
    }
  }
}
```


```
# Make predictions
preds <- predict(choose_model, df_test)
df_test$Pred <- preds
```


```
# Viewing angles for the plots
angle_columns = c("Stim","Azim","Colatitude") 
df_angles = data.frame(matrix(nrow = 4, ncol = length(angle_columns))) 
colnames(df_angles) = angle_columns

df_angles$Stim <- stim_list
df_angles$Azim <- c(0, 0, 0, 0) # Theta, default 0
df_angles$Colatitude <- c(15, 15, 15, 15) # Phi, default 15
```


```
# Make plots
for (s in stim_list){
  for (t in c("3back")){
    disp_df <- subset(df_test, Stim == s & Task == t)
    persp(x=seq(i_start, i_stop, length.out = 10), y=seq(i_start, i_stop, length.out = 10), z=matrix(disp_df$Pred, nrow=10, ncol=10,byrow=TRUE), 
          theta = subset(df_angles, Stim == s)$Azim, phi = subset(df_angles, Stim == s)$Colatitude,
          xlab="EF_front",ylab="EF_par",zlab="Pred_Acc_m", main=paste(s,t), zlim=c(-0.16, 0.12)) 
  }
}
```


```
# Export the data for plotting
save_file_name <- file.path(paste(data_directory, "EPFL_Efield_interactions.csv", sep='/'))
write.csv(df_test, save_file_name)
```

#### Note

The results show a triple interaction of the stimulation with the
magnitude of the electric fields at both stimulation sites. A comparison
of the rate of improvement in accuracy in this context is not so
straightforward, but a verbatim interpretation could be that there is a
correlation between the magnitude of both electric fields (which is
expected from the design), and a correlation between the stimulation
groups and these fields. To test this relationship, we will use an
additional measure we drew (described at the beginning of this section),
which we dub “symmetry”. As the electric fields are correlated and,
following the hypotheses justifying the application of bifocal
stimulation within this study, we could expect for the effects of
stimulation to be maximum when the magnitude of the induced electric
fields is comparable among sites (i.e., the application is symmetric, so
symmetry = 1), and when the magnitude of the induced field (EF\_mag)
approaches a maximum capped by the magnitude of the applied current. As
such, we will now assess the effects of these combined measures on the
change in performance.

#### Symmetry


```
data_subset <- subset(df_ef_epfl, Stim != 'Placebo')
data_subset <- droplevels(data_subset)
levels(data_subset$Stim)
```


```
[1] "GPre-TPar" "TPre-GPar" "Tpar-GBst" "Tpre-GBst"
```


```
m0 <- lm(formula = Acc_m ~ Task*Stim*EF_sym, data=data_subset)
summary(m0)
```


```
Call:
lm(formula = Acc_m ~ Task * Stim * EF_sym, data = data_subset)

Residuals:
      Min        1Q    Median        3Q       Max 
-0.023535 -0.006024 -0.001543  0.005545  0.052711 

Coefficients:
                                Estimate Std. Error t value Pr(>|t|)  
(Intercept)                     0.002874   0.006133   0.469   0.6401  
Task3back                       0.002274   0.008673   0.262   0.7936  
StimTPre-GPar                   0.017725   0.008673   2.044   0.0429 *
StimTpar-GBst                   0.001632   0.008884   0.184   0.8545  
StimTpre-GBst                   0.001366   0.008884   0.154   0.8781  
EF_sym                          0.003579   0.003876   0.923   0.3574  
Task3back:StimTPre-GPar        -0.018369   0.012266  -1.498   0.1366  
Task3back:StimTpar-GBst        -0.013489   0.012563  -1.074   0.2849  
Task3back:StimTpre-GBst        -0.002737   0.012563  -0.218   0.8279  
Task3back:EF_sym               -0.006594   0.005482  -1.203   0.2311  
StimTPre-GPar:EF_sym           -0.012299   0.005482  -2.244   0.0265 *
StimTpar-GBst:EF_sym           -0.004308   0.005552  -0.776   0.4391  
StimTpre-GBst:EF_sym           -0.005050   0.005552  -0.910   0.3646  
Task3back:StimTPre-GPar:EF_sym  0.012627   0.007753   1.629   0.1057  
Task3back:StimTpar-GBst:EF_sym  0.013353   0.007851   1.701   0.0913 .
Task3back:StimTpre-GBst:EF_sym  0.004143   0.007851   0.528   0.5985  
---
Signif. codes:  0 ‘***’ 0.001 ‘**’ 0.01 ‘*’ 0.05 ‘.’ 0.1 ‘ ’ 1

Residual standard error: 0.01051 on 136 degrees of freedom
Multiple R-squared:  0.1598,    Adjusted R-squared:  0.06712 
F-statistic: 1.724 on 15 and 136 DF,  p-value: 0.05295
```


```
anova(m0)
```


```
Analysis of Variance Table

Response: Acc_m
                  Df    Sum Sq    Mean Sq F value   Pr(>F)   
Task               1 0.0009872 0.00098717  8.9310 0.003328 **
Stim               3 0.0004679 0.00015596  1.4110 0.242302   
EF_sym             1 0.0001126 0.00011256  1.0183 0.314707   
Task:Stim          3 0.0002492 0.00008308  0.7516 0.523209   
Task:EF_sym        1 0.0000118 0.00001179  0.1066 0.744521   
Stim:EF_sym        3 0.0005673 0.00018909  1.7107 0.167729   
Task:Stim:EF_sym   3 0.0004629 0.00015430  1.3960 0.246764   
Residuals        136 0.0150325 0.00011053                    
---
Signif. codes:  0 ‘***’ 0.001 ‘**’ 0.01 ‘*’ 0.05 ‘.’ 0.1 ‘ ’ 1
```


```
m1 <- lmer(formula = Acc_m ~ Task*Stim*EF_sym + (1 | ID), data=data_subset)
summary(m1)
```


```
Linear mixed model fit by REML. t-tests use Satterthwaite's method ['lmerModLmerTest']
Formula: Acc_m ~ Task * Stim * EF_sym + (1 | ID)
   Data: data_subset

REML criterion at convergence: -814

Scaled residuals: 
    Min      1Q  Median      3Q     Max 
-2.1794 -0.5895 -0.1616  0.4974  4.9390 

Random effects:
 Groups   Name        Variance  Std.Dev.
 ID       (Intercept) 2.584e-06 0.001608
 Residual             1.079e-04 0.010390
Number of obs: 152, groups:  ID, 20

Fixed effects:
                                 Estimate Std. Error         df t value Pr(>|t|)  
(Intercept)                      0.002874   0.006133 135.505602   0.469   0.6401  
Task3back                        0.002274   0.008571 118.604450   0.265   0.7913  
StimTPre-GPar                    0.017725   0.008571 118.604451   2.068   0.0408 *
StimTpar-GBst                    0.001575   0.008783 119.988205   0.179   0.8580  
StimTpre-GBst                    0.001308   0.008783 119.988205   0.149   0.8819  
EF_sym                           0.003579   0.003876 135.505602   0.923   0.3574  
Task3back:StimTPre-GPar         -0.018369   0.012121 118.604450  -1.515   0.1323  
Task3back:StimTpar-GBst         -0.013489   0.012415 118.604450  -1.086   0.2795  
Task3back:StimTpre-GBst         -0.002737   0.012415 118.604451  -0.220   0.8259  
Task3back:EF_sym                -0.006594   0.005417 118.604450  -1.217   0.2259  
StimTPre-GPar:EF_sym            -0.012299   0.005417 118.604451  -2.270   0.0250 *
StimTpar-GBst:EF_sym            -0.004270   0.005488 119.345413  -0.778   0.4380  
StimTpre-GBst:EF_sym            -0.005012   0.005488 119.345413  -0.913   0.3629  
Task3back:StimTPre-GPar:EF_sym   0.012627   0.007661 118.604451   1.648   0.1020  
Task3back:StimTpar-GBst:EF_sym   0.013353   0.007759 118.604450   1.721   0.0878 .
Task3back:StimTpre-GBst:EF_sym   0.004143   0.007759 118.604451   0.534   0.5943  
---
Signif. codes:  0 ‘***’ 0.001 ‘**’ 0.01 ‘*’ 0.05 ‘.’ 0.1 ‘ ’ 1
```


```
Correlation matrix not shown by default, as p = 16 > 12.
Use print(x, correlation=TRUE)  or
    vcov(x)        if you need it
```


```
anova(m1)
```


```
Type III Analysis of Variance Table with Satterthwaite's method
                     Sum Sq    Mean Sq NumDF   DenDF F value Pr(>F)
Task             0.00022768 0.00022768     1 118.604  2.1092 0.1491
Stim             0.00054872 0.00018290     3 120.399  1.6944 0.1718
EF_sym           0.00008768 0.00008768     1  17.208  0.8123 0.3799
Task:Stim        0.00032684 0.00010895     3 118.604  1.0093 0.3913
Task:EF_sym      0.00001258 0.00001258     1 118.604  0.1165 0.7334
Stim:EF_sym      0.00056998 0.00018999     3 119.578  1.7601 0.1585
Task:Stim:EF_sym 0.00046290 0.00015430     3 118.604  1.4294 0.2377
```


```
AIC(m0, m1)
```


```
BIC(m0, m1)
```


```
m_acc_m_sym <- lm(formula = Acc_m ~ Task*Stim*EF_sym, data=data_subset)
summary(m_acc_m_sym)
```


```
Call:
lm(formula = Acc_m ~ Task * Stim * EF_sym, data = data_subset)

Residuals:
      Min        1Q    Median        3Q       Max 
-0.023535 -0.006024 -0.001543  0.005545  0.052711 

Coefficients:
                                Estimate Std. Error t value Pr(>|t|)  
(Intercept)                     0.002874   0.006133   0.469   0.6401  
Task3back                       0.002274   0.008673   0.262   0.7936  
StimTPre-GPar                   0.017725   0.008673   2.044   0.0429 *
StimTpar-GBst                   0.001632   0.008884   0.184   0.8545  
StimTpre-GBst                   0.001366   0.008884   0.154   0.8781  
EF_sym                          0.003579   0.003876   0.923   0.3574  
Task3back:StimTPre-GPar        -0.018369   0.012266  -1.498   0.1366  
Task3back:StimTpar-GBst        -0.013489   0.012563  -1.074   0.2849  
Task3back:StimTpre-GBst        -0.002737   0.012563  -0.218   0.8279  
Task3back:EF_sym               -0.006594   0.005482  -1.203   0.2311  
StimTPre-GPar:EF_sym           -0.012299   0.005482  -2.244   0.0265 *
StimTpar-GBst:EF_sym           -0.004308   0.005552  -0.776   0.4391  
StimTpre-GBst:EF_sym           -0.005050   0.005552  -0.910   0.3646  
Task3back:StimTPre-GPar:EF_sym  0.012627   0.007753   1.629   0.1057  
Task3back:StimTpar-GBst:EF_sym  0.013353   0.007851   1.701   0.0913 .
Task3back:StimTpre-GBst:EF_sym  0.004143   0.007851   0.528   0.5985  
---
Signif. codes:  0 ‘***’ 0.001 ‘**’ 0.01 ‘*’ 0.05 ‘.’ 0.1 ‘ ’ 1

Residual standard error: 0.01051 on 136 degrees of freedom
Multiple R-squared:  0.1598,    Adjusted R-squared:  0.06712 
F-statistic: 1.724 on 15 and 136 DF,  p-value: 0.05295
```


```
anova(m_acc_m_sym)
```


```
Analysis of Variance Table

Response: Acc_m
                  Df    Sum Sq    Mean Sq F value   Pr(>F)   
Task               1 0.0009872 0.00098717  8.9310 0.003328 **
Stim               3 0.0004679 0.00015596  1.4110 0.242302   
EF_sym             1 0.0001126 0.00011256  1.0183 0.314707   
Task:Stim          3 0.0002492 0.00008308  0.7516 0.523209   
Task:EF_sym        1 0.0000118 0.00001179  0.1066 0.744521   
Stim:EF_sym        3 0.0005673 0.00018909  1.7107 0.167729   
Task:Stim:EF_sym   3 0.0004629 0.00015430  1.3960 0.246764   
Residuals        136 0.0150325 0.00011053                    
---
Signif. codes:  0 ‘***’ 0.001 ‘**’ 0.01 ‘*’ 0.05 ‘.’ 0.1 ‘ ’ 1
```


```
data_subset <- subset(df_ef_epfl, Task == '2back' & Stim != 'Placebo')
data_subset <- droplevels(data_subset)
levels(data_subset$Task)
```


```
[1] "2back"
```


```
levels(data_subset$Stim)
```


```
[1] "GPre-TPar" "TPre-GPar" "Tpar-GBst" "Tpre-GBst"
```


```
m_acc_m_sym_2back <- lm(formula = Acc_m ~ Stim*EF_sym, data=data_subset)
summary(m_acc_m_sym_2back)
```


```
Call:
lm(formula = Acc_m ~ Stim * EF_sym, data = data_subset)

Residuals:
      Min        1Q    Median        3Q       Max 
-0.023535 -0.007482 -0.001407  0.004140  0.052711 

Coefficients:
                      Estimate Std. Error t value Pr(>|t|)  
(Intercept)           0.002874   0.006555   0.438   0.6625  
StimTPre-GPar         0.017725   0.009271   1.912   0.0601 .
StimTpar-GBst         0.001632   0.009496   0.172   0.8640  
StimTpre-GBst         0.001366   0.009496   0.144   0.8861  
EF_sym                0.003579   0.004143   0.864   0.3907  
StimTPre-GPar:EF_sym -0.012299   0.005860  -2.099   0.0395 *
StimTpar-GBst:EF_sym -0.004308   0.005934  -0.726   0.4703  
StimTpre-GBst:EF_sym -0.005050   0.005934  -0.851   0.3977  
---
Signif. codes:  0 ‘***’ 0.001 ‘**’ 0.01 ‘*’ 0.05 ‘.’ 0.1 ‘ ’ 1

Residual standard error: 0.01124 on 68 degrees of freedom
Multiple R-squared:  0.1237,    Adjusted R-squared:  0.03347 
F-statistic: 1.371 on 7 and 68 DF,  p-value: 0.2318
```


```
anova(m_acc_m_sym_2back)
```


```
Analysis of Variance Table

Response: Acc_m
            Df    Sum Sq    Mean Sq F value Pr(>F)
Stim         3 0.0005396 1.7986e-04  1.4242 0.2433
EF_sym       1 0.0000986 9.8595e-05  0.7807 0.3800
Stim:EF_sym  3 0.0005738 1.9127e-04  1.5146 0.2186
Residuals   68 0.0085875 1.2629e-04
```


```
data_subset <- subset(df_ef_epfl, Task == '3back' & Stim != 'Placebo')
data_subset <- droplevels(data_subset)
levels(data_subset$Task)
```


```
[1] "3back"
```


```
levels(data_subset$Stim)
```


```
[1] "GPre-TPar" "TPre-GPar" "Tpar-GBst" "Tpre-GBst"
```


```
m_acc_m_sym_3back <- lm(formula = Acc_m ~ Stim*EF_sym, data=data_subset)
summary(m_acc_m_sym_3back)
```


```
Call:
lm(formula = Acc_m ~ Stim * EF_sym, data = data_subset)

Residuals:
      Min        1Q    Median        3Q       Max 
-0.018702 -0.005224 -0.002210  0.006296  0.020996 

Coefficients:
                       Estimate Std. Error t value Pr(>|t|)  
(Intercept)           0.0051476  0.0056790   0.906    0.368  
StimTPre-GPar        -0.0006440  0.0080313  -0.080    0.936  
StimTpar-GBst        -0.0118563  0.0082261  -1.441    0.154  
StimTpre-GBst        -0.0013715  0.0082261  -0.167    0.868  
EF_sym               -0.0030150  0.0035894  -0.840    0.404  
StimTPre-GPar:EF_sym  0.0003279  0.0050762   0.065    0.949  
StimTpar-GBst:EF_sym  0.0090453  0.0051407   1.760    0.083 .
StimTpre-GBst:EF_sym -0.0009066  0.0051407  -0.176    0.861  
---
Signif. codes:  0 ‘***’ 0.001 ‘**’ 0.01 ‘*’ 0.05 ‘.’ 0.1 ‘ ’ 1

Residual standard error: 0.009735 on 68 degrees of freedom
Multiple R-squared:  0.09285,   Adjusted R-squared:  -0.0005335 
F-statistic: 0.9943 on 7 and 68 DF,  p-value: 0.443
```


```
anova(m_acc_m_sym_3back)
```


```
Analysis of Variance Table

Response: Acc_m
            Df    Sum Sq    Mean Sq F value Pr(>F)
Stim         3 0.0001775 5.9180e-05  0.6244 0.6017
EF_sym       1 0.0000258 2.5750e-05  0.2717 0.6039
Stim:EF_sym  3 0.0004564 1.5212e-04  1.6050 0.1963
Residuals   68 0.0064449 9.4778e-05
```

#### E-field magnitude

The results for the tests on the symmetry of the applied magnetic
fields did not yield statistically significant results, which may
suggest this measure is not capturing the interaction we saw when
including the magnitude of both electric fields separately. A possible
explanation for this is that what’s having a significant effect on the
rate of change in accuracy is not the ratio of the induced field in one
site with respect to the other, but rather the total amount of energy
that’s being injected. Next, we will use the total magnitude of the
combined electric fields as a predictor for changes in performance.


```
data_subset <- subset(df_ef_epfl, Stim != 'Placebo')
data_subset <- droplevels(data_subset)
levels(data_subset$Stim)
```


```
[1] "GPre-TPar" "TPre-GPar" "Tpar-GBst" "Tpre-GBst"
```


```
m0 <- lm(formula = Acc_m ~ Task*Stim*EF_mag, data=data_subset)
summary(m0)
```


```
Call:
lm(formula = Acc_m ~ Task * Stim * EF_mag, data = data_subset)

Residuals:
      Min        1Q    Median        3Q       Max 
-0.019737 -0.006423 -0.001419  0.006117  0.051993 

Coefficients:
                                 Estimate Std. Error t value Pr(>|t|)
(Intercept)                     0.0044746  0.0052149   0.858    0.392
Task3back                      -0.0049645  0.0073749  -0.673    0.502
StimTPre-GPar                   0.0028591  0.0073749   0.388    0.699
StimTpar-GBst                   0.0032975  0.0071821   0.459    0.647
StimTpre-GBst                   0.0021279  0.0071821   0.296    0.767
EF_mag                          0.0358860  0.0457874   0.784    0.435
Task3back:StimTPre-GPar        -0.0021857  0.0104297  -0.210    0.834
Task3back:StimTpar-GBst         0.0003384  0.0101571   0.033    0.973
Task3back:StimTpre-GBst        -0.0095713  0.0101571  -0.942    0.348
Task3back:EF_mag               -0.0237096  0.0647532  -0.366    0.715
StimTPre-GPar:EF_mag           -0.0307105  0.0647532  -0.474    0.636
StimTpar-GBst:EF_mag           -0.0585579  0.0508411  -1.152    0.251
StimTpre-GBst:EF_mag           -0.0596205  0.0508411  -1.173    0.243
Task3back:StimTPre-GPar:EF_mag  0.0224241  0.0915748   0.245    0.807
Task3back:StimTpar-GBst:EF_mag  0.0419202  0.0719002   0.583    0.561
Task3back:StimTpre-GBst:EF_mag  0.0779505  0.0719002   1.084    0.280

Residual standard error: 0.01072 on 136 degrees of freedom
Multiple R-squared:  0.1265,    Adjusted R-squared:  0.03011 
F-statistic: 1.313 on 15 and 136 DF,  p-value: 0.2027
```


```
anova(m0)
```


```
Analysis of Variance Table

Response: Acc_m
                  Df    Sum Sq    Mean Sq F value   Pr(>F)   
Task               1 0.0009872 0.00098717  8.5903 0.003966 **
Stim               3 0.0004679 0.00015596  1.3572 0.258630   
EF_mag             1 0.0000024 0.00000237  0.0207 0.885907   
Task:Stim          3 0.0002492 0.00008308  0.7230 0.539934   
Task:EF_mag        1 0.0002118 0.00021184  1.8434 0.176801   
Stim:EF_mag        3 0.0001552 0.00005174  0.4502 0.717561   
Task:Stim:EF_mag   3 0.0001888 0.00006294  0.5477 0.650551   
Residuals        136 0.0156288 0.00011492                    
---
Signif. codes:  0 ‘***’ 0.001 ‘**’ 0.01 ‘*’ 0.05 ‘.’ 0.1 ‘ ’ 1
```


```
m1 <- lmer(formula = Acc_m ~ Task*Stim*EF_mag + (1 | ID), data=data_subset)
summary(m1)
```


```
Linear mixed model fit by REML. t-tests use Satterthwaite's method ['lmerModLmerTest']
Formula: Acc_m ~ Task * Stim * EF_mag + (1 | ID)
   Data: data_subset

REML criterion at convergence: -841.8

Scaled residuals: 
    Min      1Q  Median      3Q     Max 
-1.7954 -0.5933 -0.1347  0.5951  4.7859 

Random effects:
 Groups   Name        Variance  Std.Dev.
 ID       (Intercept) 2.352e-06 0.001534
 Residual             1.126e-04 0.010609
Number of obs: 152, groups:  ID, 20

Fixed effects:
                                 Estimate Std. Error         df t value Pr(>|t|)
(Intercept)                     4.529e-03  5.214e-03  1.357e+02   0.869    0.387
Task3back                      -4.965e-03  7.299e-03  1.188e+02  -0.680    0.498
StimTPre-GPar                   2.859e-03  7.299e-03  1.188e+02   0.392    0.696
StimTpar-GBst                   3.162e-03  7.116e-03  1.220e+02   0.444    0.658
StimTpre-GBst                   1.992e-03  7.116e-03  1.220e+02   0.280    0.780
EF_mag                          3.535e-02  4.578e-02  1.357e+02   0.772    0.441
Task3back:StimTPre-GPar        -2.186e-03  1.032e-02  1.188e+02  -0.212    0.833
Task3back:StimTpar-GBst         3.384e-04  1.005e-02  1.188e+02   0.034    0.973
Task3back:StimTpre-GBst        -9.571e-03  1.005e-02  1.188e+02  -0.952    0.343
Task3back:EF_mag               -2.371e-02  6.409e-02  1.188e+02  -0.370    0.712
StimTPre-GPar:EF_mag           -3.071e-02  6.409e-02  1.188e+02  -0.479    0.633
StimTpar-GBst:EF_mag           -5.755e-02  5.046e-02  1.273e+02  -1.140    0.256
StimTpre-GBst:EF_mag           -5.861e-02  5.046e-02  1.273e+02  -1.161    0.248
Task3back:StimTPre-GPar:EF_mag  2.242e-02  9.063e-02  1.188e+02   0.247    0.805
Task3back:StimTpar-GBst:EF_mag  4.192e-02  7.116e-02  1.188e+02   0.589    0.557
Task3back:StimTpre-GBst:EF_mag  7.795e-02  7.116e-02  1.188e+02   1.095    0.276
```


```
Correlation matrix not shown by default, as p = 16 > 12.
Use print(x, correlation=TRUE)  or
    vcov(x)        if you need it
```


```
anova(m1)
```


```
Type III Analysis of Variance Table with Satterthwaite's method
                     Sum Sq    Mean Sq NumDF   DenDF F value  Pr(>F)  
Task             0.00054482 0.00054482     1 118.779  4.8402 0.02974 *
Stim             0.00019325 0.00006442     3 122.746  0.5723 0.63429  
EF_mag           0.00001296 0.00001296     1  22.813  0.1151 0.73751  
Task:Stim        0.00014839 0.00004946     3 118.779  0.4394 0.72521  
Task:EF_mag      0.00002503 0.00002503     1 118.779  0.2224 0.63810  
Stim:EF_mag      0.00014707 0.00004902     3 126.353  0.4355 0.72795  
Task:Stim:EF_mag 0.00018881 0.00006294     3 118.779  0.5591 0.64303  
---
Signif. codes:  0 ‘***’ 0.001 ‘**’ 0.01 ‘*’ 0.05 ‘.’ 0.1 ‘ ’ 1
```


```
AIC(m0, m1)
```


```
BIC(m0, m1)
```


```
m_acc_m_mag <- lm(formula = Acc_m ~ Task*Stim*EF_mag, data=data_subset)
summary(m_acc_m_mag)
```


```
Call:
lm(formula = Acc_m ~ Task * Stim * EF_mag, data = data_subset)

Residuals:
      Min        1Q    Median        3Q       Max 
-0.019737 -0.006423 -0.001419  0.006117  0.051993 

Coefficients:
                                 Estimate Std. Error t value Pr(>|t|)
(Intercept)                     0.0044746  0.0052149   0.858    0.392
Task3back                      -0.0049645  0.0073749  -0.673    0.502
StimTPre-GPar                   0.0028591  0.0073749   0.388    0.699
StimTpar-GBst                   0.0032975  0.0071821   0.459    0.647
StimTpre-GBst                   0.0021279  0.0071821   0.296    0.767
EF_mag                          0.0358860  0.0457874   0.784    0.435
Task3back:StimTPre-GPar        -0.0021857  0.0104297  -0.210    0.834
Task3back:StimTpar-GBst         0.0003384  0.0101571   0.033    0.973
Task3back:StimTpre-GBst        -0.0095713  0.0101571  -0.942    0.348
Task3back:EF_mag               -0.0237096  0.0647532  -0.366    0.715
StimTPre-GPar:EF_mag           -0.0307105  0.0647532  -0.474    0.636
StimTpar-GBst:EF_mag           -0.0585579  0.0508411  -1.152    0.251
StimTpre-GBst:EF_mag           -0.0596205  0.0508411  -1.173    0.243
Task3back:StimTPre-GPar:EF_mag  0.0224241  0.0915748   0.245    0.807
Task3back:StimTpar-GBst:EF_mag  0.0419202  0.0719002   0.583    0.561
Task3back:StimTpre-GBst:EF_mag  0.0779505  0.0719002   1.084    0.280

Residual standard error: 0.01072 on 136 degrees of freedom
Multiple R-squared:  0.1265,    Adjusted R-squared:  0.03011 
F-statistic: 1.313 on 15 and 136 DF,  p-value: 0.2027
```


```
anova(m_acc_m_mag)
```


```
Analysis of Variance Table

Response: Acc_m
                  Df    Sum Sq    Mean Sq F value   Pr(>F)   
Task               1 0.0009872 0.00098717  8.5903 0.003966 **
Stim               3 0.0004679 0.00015596  1.3572 0.258630   
EF_mag             1 0.0000024 0.00000237  0.0207 0.885907   
Task:Stim          3 0.0002492 0.00008308  0.7230 0.539934   
Task:EF_mag        1 0.0002118 0.00021184  1.8434 0.176801   
Stim:EF_mag        3 0.0001552 0.00005174  0.4502 0.717561   
Task:Stim:EF_mag   3 0.0001888 0.00006294  0.5477 0.650551   
Residuals        136 0.0156288 0.00011492                    
---
Signif. codes:  0 ‘***’ 0.001 ‘**’ 0.01 ‘*’ 0.05 ‘.’ 0.1 ‘ ’ 1
```


```
data_subset <- subset(df_ef_epfl, Task == '2back' & Stim != 'Placebo')
data_subset <- droplevels(data_subset)
levels(data_subset$Task)
```


```
[1] "2back"
```


```
levels(data_subset$Stim)
```


```
[1] "GPre-TPar" "TPre-GPar" "Tpar-GBst" "Tpre-GBst"
```


```
m_acc_m_mag_2back <- lm(formula = Acc_m ~ Stim*EF_mag, data=data_subset)
summary(m_acc_m_mag_2back)
```


```
Call:
lm(formula = Acc_m ~ Stim * EF_mag, data = data_subset)

Residuals:
      Min        1Q    Median        3Q       Max 
-0.019737 -0.006919 -0.001423  0.005244  0.051993 

Coefficients:
                      Estimate Std. Error t value Pr(>|t|)
(Intercept)           0.004475   0.005576   0.802    0.425
StimTPre-GPar         0.002859   0.007886   0.363    0.718
StimTpar-GBst         0.003297   0.007680   0.429    0.669
StimTpre-GBst         0.002128   0.007680   0.277    0.783
EF_mag                0.035886   0.048959   0.733    0.466
StimTPre-GPar:EF_mag -0.030711   0.069238  -0.444    0.659
StimTpar-GBst:EF_mag -0.058558   0.054363  -1.077    0.285
StimTpre-GBst:EF_mag -0.059620   0.054363  -1.097    0.277

Residual standard error: 0.01146 on 68 degrees of freedom
Multiple R-squared:  0.08829,   Adjusted R-squared:  -0.005566 
F-statistic: 0.9407 on 7 and 68 DF,  p-value: 0.4814
```


```
anova(m_acc_m_mag_2back)
```


```
Analysis of Variance Table

Response: Acc_m
            Df    Sum Sq    Mean Sq F value Pr(>F)
Stim         3 0.0005396 0.00017986  1.3689 0.2597
EF_mag       1 0.0001295 0.00012954  0.9859 0.3243
Stim:EF_mag  3 0.0001960 0.00006535  0.4974 0.6853
Residuals   68 0.0089344 0.00013139
```


```
data_subset <- subset(df_ef_epfl, Task == '3back' & Stim != 'Placebo')
data_subset <- droplevels(data_subset)
levels(data_subset$Task)
```


```
[1] "3back"
```


```
levels(data_subset$Stim)
```


```
[1] "GPre-TPar" "TPre-GPar" "Tpar-GBst" "Tpre-GBst"
```


```
m_acc_m_mag_3back <- lm(formula = Acc_m ~ Stim*EF_mag, data=data_subset)
summary(m_acc_m_mag_3back)
```


```
Call:
lm(formula = Acc_m ~ Stim * EF_mag, data = data_subset)

Residuals:
      Min        1Q    Median        3Q       Max 
-0.019332 -0.006048 -0.001419  0.007114  0.018697 

Coefficients:
                       Estimate Std. Error t value Pr(>|t|)
(Intercept)          -0.0004899  0.0048267  -0.101    0.919
StimTPre-GPar         0.0006733  0.0068260   0.099    0.922
StimTpar-GBst         0.0036359  0.0066476   0.547    0.586
StimTpre-GBst        -0.0074434  0.0066476  -1.120    0.267
EF_mag                0.0121764  0.0423793   0.287    0.775
StimTPre-GPar:EF_mag -0.0082865  0.0599334  -0.138    0.890
StimTpar-GBst:EF_mag -0.0166377  0.0470569  -0.354    0.725
StimTpre-GBst:EF_mag  0.0183301  0.0470569   0.390    0.698

Residual standard error: 0.009922 on 68 degrees of freedom
Multiple R-squared:  0.05774,   Adjusted R-squared:  -0.03926 
F-statistic: 0.5952 on 7 and 68 DF,  p-value: 0.7576
```


```
anova(m_acc_m_mag_3back)
```


```
Analysis of Variance Table

Response: Acc_m
            Df    Sum Sq    Mean Sq F value Pr(>F)
Stim         3 0.0001775 5.9180e-05  0.6011 0.6165
EF_mag       1 0.0000847 8.4678e-05  0.8601 0.3570
Stim:EF_mag  3 0.0001480 4.9323e-05  0.5010 0.6828
Residuals   68 0.0066944 9.8447e-05
```

### Normalized accuracy and the magnitude of the induced electric fields


```
data_subset <- subset(df_ef_epfl, Stim != 'Placebo')
data_subset <- droplevels(data_subset)
levels(data_subset$Stim)
```


```
[1] "GPre-TPar" "TPre-GPar" "Tpar-GBst" "Tpre-GBst"
```


```
m0 <- lm(formula = Acc_n_m ~ Task*Stim*EF_front*EF_par, data=data_subset)
summary(m0)
```


```
Call:
lm(formula = Acc_n_m ~ Task * Stim * EF_front * EF_par, data = data_subset)

Residuals:
      Min        1Q    Median        3Q       Max 
-0.041990 -0.010182 -0.002017  0.006833  0.118010 

Coefficients:
                                          Estimate Std. Error t value Pr(>|t|)  
(Intercept)                               0.027671   0.022450   1.233   0.2201  
Task3back                                -0.008159   0.031749  -0.257   0.7976  
StimTPre-GPar                            -0.063180   0.031749  -1.990   0.0489 *
StimTpar-GBst                            -0.021082   0.031686  -0.665   0.5071  
StimTpre-GBst                            -0.020268   0.031686  -0.640   0.5236  
EF_front                                 -0.086907   0.266705  -0.326   0.7451  
EF_par                                   -0.401667   0.416649  -0.964   0.3370  
Task3back:StimTPre-GPar                   0.013655   0.044900   0.304   0.7616  
Task3back:StimTpar-GBst                   0.008110   0.044811   0.181   0.8567  
Task3back:StimTpre-GBst                   0.008427   0.044811   0.188   0.8512  
Task3back:EF_front                       -0.244806   0.377177  -0.649   0.5175  
StimTPre-GPar:EF_front                    0.391335   0.377177   1.038   0.3016  
StimTpar-GBst:EF_front                    0.075321   0.291825   0.258   0.7968  
StimTpre-GBst:EF_front                    0.058409   0.291825   0.200   0.8417  
Task3back:EF_par                          0.143250   0.589231   0.243   0.8083  
StimTPre-GPar:EF_par                      1.529212   0.589231   2.595   0.0106 *
StimTpar-GBst:EF_par                      0.341062   0.515737   0.661   0.5097  
StimTpre-GBst:EF_par                      0.475394   0.515737   0.922   0.3585  
EF_front:EF_par                           4.232322   3.304077   1.281   0.2027  
Task3back:StimTPre-GPar:EF_front          0.238506   0.533409   0.447   0.6556  
Task3back:StimTpar-GBst:EF_front          0.311272   0.412703   0.754   0.4522  
Task3back:StimTpre-GBst:EF_front          0.156311   0.412703   0.379   0.7055  
Task3back:StimTPre-GPar:EF_par           -0.637102   0.833299  -0.765   0.4460  
Task3back:StimTpar-GBst:EF_par           -0.199735   0.729362  -0.274   0.7847  
Task3back:StimTpre-GBst:EF_par           -0.324262   0.729362  -0.445   0.6574  
Task3back:EF_front:EF_par                -0.053112   4.672670  -0.011   0.9909  
StimTPre-GPar:EF_front:EF_par           -10.839561   4.672670  -2.320   0.0220 *
StimTpar-GBst:EF_front:EF_par            -4.125344   3.437340  -1.200   0.2324  
StimTpre-GBst:EF_front:EF_par            -4.521532   3.437340  -1.315   0.1909  
Task3back:StimTPre-GPar:EF_front:EF_par   1.470894   6.608153   0.223   0.8242  
Task3back:StimTpar-GBst:EF_front:EF_par  -0.009014   4.861133  -0.002   0.9985  
Task3back:StimTpre-GBst:EF_front:EF_par   1.297504   4.861133   0.267   0.7900  
---
Signif. codes:  0 ‘***’ 0.001 ‘**’ 0.01 ‘*’ 0.05 ‘.’ 0.1 ‘ ’ 1

Residual standard error: 0.0212 on 120 degrees of freedom
Multiple R-squared:  0.2778,    Adjusted R-squared:  0.09122 
F-statistic: 1.489 on 31 and 120 DF,  p-value: 0.06674
```


```
anova(m0)
```


```
Analysis of Variance Table

Response: Acc_n_m
                           Df   Sum Sq   Mean Sq F value   Pr(>F)   
Task                        1 0.003744 0.0037439  8.3275 0.004632 **
Stim                        3 0.002773 0.0009242  2.0557 0.109738   
EF_front                    1 0.000080 0.0000804  0.1788 0.673197   
EF_par                      1 0.000326 0.0003264  0.7260 0.395866   
Task:Stim                   3 0.002988 0.0009960  2.2154 0.089841 . 
Task:EF_front               1 0.000155 0.0001553  0.3454 0.557809   
Stim:EF_front               3 0.001588 0.0005294  1.1775 0.321273   
Task:EF_par                 1 0.000012 0.0000124  0.0277 0.868202   
Stim:EF_par                 3 0.001471 0.0004902  1.0903 0.355998   
EF_front:EF_par             1 0.000034 0.0000336  0.0748 0.784995   
Task:Stim:EF_front          3 0.001731 0.0005772  1.2837 0.283140   
Task:Stim:EF_par            3 0.001013 0.0003376  0.7508 0.523960   
Task:EF_front:EF_par        1 0.000194 0.0001937  0.4309 0.512800   
Stim:EF_front:EF_par        3 0.004406 0.0014685  3.2664 0.023778 * 
Task:Stim:EF_front:EF_par   3 0.000236 0.0000787  0.1751 0.913078   
Residuals                 120 0.053950 0.0004496                    
---
Signif. codes:  0 ‘***’ 0.001 ‘**’ 0.01 ‘*’ 0.05 ‘.’ 0.1 ‘ ’ 1
```


```
m1 <- lmer(formula = Acc_n_m ~ Task*Stim*EF_front*EF_par + (1 | ID), data=data_subset)
summary(m1)
```


```
Linear mixed model fit by REML. t-tests use Satterthwaite's method ['lmerModLmerTest']
Formula: Acc_n_m ~ Task * Stim * EF_front * EF_par + (1 | ID)
   Data: data_subset

REML criterion at convergence: -685.6

Scaled residuals: 
    Min      1Q  Median      3Q     Max 
-1.9382 -0.4874 -0.1388  0.3078  5.3685 

Random effects:
 Groups   Name        Variance  Std.Dev.
 ID       (Intercept) 2.119e-05 0.004603
 Residual             4.281e-04 0.020692
Number of obs: 152, groups:  ID, 20

Fixed effects:
                                          Estimate Std. Error         df t value Pr(>|t|)   
(Intercept)                               0.027822   0.022381 119.987012   1.243  0.21625   
Task3back                                -0.008159   0.030982 104.822414  -0.263  0.79281   
StimTPre-GPar                            -0.063180   0.030982 104.822422  -2.039  0.04394 * 
StimTpar-GBst                            -0.021329   0.031234 114.691630  -0.683  0.49605   
StimTpre-GBst                            -0.020516   0.031234 114.691629  -0.657  0.51260   
EF_front                                 -0.087975   0.266343 119.334084  -0.330  0.74175   
EF_par                                   -0.404152   0.415144 119.985480  -0.974  0.33225   
Task3back:StimTPre-GPar                   0.013655   0.043816 104.822416   0.312  0.75593   
Task3back:StimTpar-GBst                   0.008110   0.043729 104.822415   0.185  0.85323   
Task3back:StimTpre-GBst                   0.008427   0.043729 104.822412   0.193  0.84756   
Task3back:EF_front                       -0.244806   0.368071 104.822419  -0.665  0.50744   
StimTPre-GPar:EF_front                    0.391335   0.368071 104.822422   1.063  0.29013   
StimTpar-GBst:EF_front                    0.078329   0.287159 114.281797   0.273  0.78552   
StimTpre-GBst:EF_front                    0.061417   0.287159 114.281797   0.214  0.83102   
Task3back:EF_par                          0.143250   0.575005 104.822411   0.249  0.80375   
StimTPre-GPar:EF_par                      1.529212   0.575005 104.822420   2.659  0.00905 **
StimTpar-GBst:EF_par                      0.336939   0.509640 116.494718   0.661  0.50983   
StimTpre-GBst:EF_par                      0.471270   0.509640 116.494717   0.925  0.35703   
EF_front:EF_par                           4.246158   3.295254 119.925644   1.289  0.20003   
Task3back:StimTPre-GPar:EF_front          0.238506   0.520531 104.822418   0.458  0.64776   
Task3back:StimTpar-GBst:EF_front          0.311272   0.402738 104.822419   0.773  0.44133   
Task3back:StimTpre-GBst:EF_front          0.156311   0.402738 104.822418   0.388  0.69871   
Task3back:StimTPre-GPar:EF_par           -0.637102   0.813179 104.822414  -0.783  0.43512   
Task3back:StimTpar-GBst:EF_par           -0.199735   0.711752 104.822411  -0.281  0.77955   
Task3back:StimTpre-GBst:EF_par           -0.324262   0.711752 104.822409  -0.456  0.64963   
Task3back:EF_front:EF_par                -0.053112   4.559851 104.822414  -0.012  0.99073   
StimTPre-GPar:EF_front:EF_par           -10.839561   4.559851 104.822422  -2.377  0.01926 * 
StimTpar-GBst:EF_front:EF_par            -4.115729   3.405868 118.684055  -1.208  0.22929   
StimTpre-GBst:EF_front:EF_par            -4.511917   3.405868 118.684055  -1.325  0.18780   
Task3back:StimTPre-GPar:EF_front:EF_par   1.470894   6.448603 104.822416   0.228  0.82002   
Task3back:StimTpar-GBst:EF_front:EF_par  -0.009014   4.743763 104.822413  -0.002  0.99849   
Task3back:StimTpre-GBst:EF_front:EF_par   1.297504   4.743763 104.822412   0.274  0.78499   
---
Signif. codes:  0 ‘***’ 0.001 ‘**’ 0.01 ‘*’ 0.05 ‘.’ 0.1 ‘ ’ 1
```


```
Correlation matrix not shown by default, as p = 32 > 12.
Use print(x, correlation=TRUE)  or
    vcov(x)        if you need it
```


```
anova(m1)
```


```
Type III Analysis of Variance Table with Satterthwaite's method
                             Sum Sq    Mean Sq NumDF   DenDF F value  Pr(>F)  
Task                      0.0000007 0.00000067     1 104.822  0.0016 0.96855  
Stim                      0.0030484 0.00101614     3 113.590  2.3734 0.07396 .
EF_front                  0.0000073 0.00000730     1  22.818  0.0171 0.89723  
EF_par                    0.0002486 0.00024862     1  39.861  0.5807 0.45052  
Task:Stim                 0.0000425 0.00001418     3 104.822  0.0331 0.99186  
Task:EF_front             0.0000985 0.00009846     1 104.822  0.2300 0.63254  
Stim:EF_front             0.0020262 0.00067540     3 111.666  1.5775 0.19882  
Task:EF_par               0.0001461 0.00014615     1 104.822  0.3414 0.56030  
Stim:EF_par               0.0044986 0.00149953     3 112.312  3.5024 0.01783 *
EF_front:EF_par           0.0000452 0.00004516     1  38.962  0.1055 0.74708  
Task:Stim:EF_front        0.0003515 0.00011717     3 104.822  0.2737 0.84427  
Task:Stim:EF_par          0.0002871 0.00009569     3 104.822  0.2235 0.87987  
Task:EF_front:EF_par      0.0000617 0.00006171     1 104.822  0.1441 0.70498  
Stim:EF_front:EF_par      0.0043898 0.00146327     3 106.584  3.4177 0.02002 *
Task:Stim:EF_front:EF_par 0.0002362 0.00007872     3 104.822  0.1839 0.90716  
---
Signif. codes:  0 ‘***’ 0.001 ‘**’ 0.01 ‘*’ 0.05 ‘.’ 0.1 ‘ ’ 1
```


```
AIC(m0, m1)
```


```
BIC(m0, m1)
```


```
m_acc_n_ef <- lm(formula = Acc_n_m ~ Task*Stim*EF_front*EF_par, data=data_subset)
summary(m_acc_n_ef)
```


```
Call:
lm(formula = Acc_n_m ~ Task * Stim * EF_front * EF_par, data = data_subset)

Residuals:
      Min        1Q    Median        3Q       Max 
-0.041990 -0.010182 -0.002017  0.006833  0.118010 

Coefficients:
                                          Estimate Std. Error t value Pr(>|t|)  
(Intercept)                               0.027671   0.022450   1.233   0.2201  
Task3back                                -0.008159   0.031749  -0.257   0.7976  
StimTPre-GPar                            -0.063180   0.031749  -1.990   0.0489 *
StimTpar-GBst                            -0.021082   0.031686  -0.665   0.5071  
StimTpre-GBst                            -0.020268   0.031686  -0.640   0.5236  
EF_front                                 -0.086907   0.266705  -0.326   0.7451  
EF_par                                   -0.401667   0.416649  -0.964   0.3370  
Task3back:StimTPre-GPar                   0.013655   0.044900   0.304   0.7616  
Task3back:StimTpar-GBst                   0.008110   0.044811   0.181   0.8567  
Task3back:StimTpre-GBst                   0.008427   0.044811   0.188   0.8512  
Task3back:EF_front                       -0.244806   0.377177  -0.649   0.5175  
StimTPre-GPar:EF_front                    0.391335   0.377177   1.038   0.3016  
StimTpar-GBst:EF_front                    0.075321   0.291825   0.258   0.7968  
StimTpre-GBst:EF_front                    0.058409   0.291825   0.200   0.8417  
Task3back:EF_par                          0.143250   0.589231   0.243   0.8083  
StimTPre-GPar:EF_par                      1.529212   0.589231   2.595   0.0106 *
StimTpar-GBst:EF_par                      0.341062   0.515737   0.661   0.5097  
StimTpre-GBst:EF_par                      0.475394   0.515737   0.922   0.3585  
EF_front:EF_par                           4.232322   3.304077   1.281   0.2027  
Task3back:StimTPre-GPar:EF_front          0.238506   0.533409   0.447   0.6556  
Task3back:StimTpar-GBst:EF_front          0.311272   0.412703   0.754   0.4522  
Task3back:StimTpre-GBst:EF_front          0.156311   0.412703   0.379   0.7055  
Task3back:StimTPre-GPar:EF_par           -0.637102   0.833299  -0.765   0.4460  
Task3back:StimTpar-GBst:EF_par           -0.199735   0.729362  -0.274   0.7847  
Task3back:StimTpre-GBst:EF_par           -0.324262   0.729362  -0.445   0.6574  
Task3back:EF_front:EF_par                -0.053112   4.672670  -0.011   0.9909  
StimTPre-GPar:EF_front:EF_par           -10.839561   4.672670  -2.320   0.0220 *
StimTpar-GBst:EF_front:EF_par            -4.125344   3.437340  -1.200   0.2324  
StimTpre-GBst:EF_front:EF_par            -4.521532   3.437340  -1.315   0.1909  
Task3back:StimTPre-GPar:EF_front:EF_par   1.470894   6.608153   0.223   0.8242  
Task3back:StimTpar-GBst:EF_front:EF_par  -0.009014   4.861133  -0.002   0.9985  
Task3back:StimTpre-GBst:EF_front:EF_par   1.297504   4.861133   0.267   0.7900  
---
Signif. codes:  0 ‘***’ 0.001 ‘**’ 0.01 ‘*’ 0.05 ‘.’ 0.1 ‘ ’ 1

Residual standard error: 0.0212 on 120 degrees of freedom
Multiple R-squared:  0.2778,    Adjusted R-squared:  0.09122 
F-statistic: 1.489 on 31 and 120 DF,  p-value: 0.06674
```


```
anova(m_acc_n_ef)
```


```
Analysis of Variance Table

Response: Acc_n_m
                           Df   Sum Sq   Mean Sq F value   Pr(>F)   
Task                        1 0.003744 0.0037439  8.3275 0.004632 **
Stim                        3 0.002773 0.0009242  2.0557 0.109738   
EF_front                    1 0.000080 0.0000804  0.1788 0.673197   
EF_par                      1 0.000326 0.0003264  0.7260 0.395866   
Task:Stim                   3 0.002988 0.0009960  2.2154 0.089841 . 
Task:EF_front               1 0.000155 0.0001553  0.3454 0.557809   
Stim:EF_front               3 0.001588 0.0005294  1.1775 0.321273   
Task:EF_par                 1 0.000012 0.0000124  0.0277 0.868202   
Stim:EF_par                 3 0.001471 0.0004902  1.0903 0.355998   
EF_front:EF_par             1 0.000034 0.0000336  0.0748 0.784995   
Task:Stim:EF_front          3 0.001731 0.0005772  1.2837 0.283140   
Task:Stim:EF_par            3 0.001013 0.0003376  0.7508 0.523960   
Task:EF_front:EF_par        1 0.000194 0.0001937  0.4309 0.512800   
Stim:EF_front:EF_par        3 0.004406 0.0014685  3.2664 0.023778 * 
Task:Stim:EF_front:EF_par   3 0.000236 0.0000787  0.1751 0.913078   
Residuals                 120 0.053950 0.0004496                    
---
Signif. codes:  0 ‘***’ 0.001 ‘**’ 0.01 ‘*’ 0.05 ‘.’ 0.1 ‘ ’ 1
```


```
data_subset <- subset(df_ef_epfl, Task == '2back' & Stim != 'Placebo')
data_subset <- droplevels(data_subset)
levels(data_subset$Task)
```


```
[1] "2back"
```


```
levels(data_subset$Stim)
```


```
[1] "GPre-TPar" "TPre-GPar" "Tpar-GBst" "Tpre-GBst"
```


```
m_acc_n_ef_2back <- lm(formula = Acc_n_m ~ Stim*EF_front*EF_par, data=data_subset)
summary(m_acc_n_ef_2back)
```


```
Call:
lm(formula = Acc_n_m ~ Stim * EF_front * EF_par, data = data_subset)

Residuals:
      Min        1Q    Median        3Q       Max 
-0.041990 -0.012666 -0.004833  0.006357  0.118010 

Coefficients:
                               Estimate Std. Error t value Pr(>|t|)  
(Intercept)                     0.02767    0.02842   0.974   0.3341  
StimTPre-GPar                  -0.06318    0.04019  -1.572   0.1212  
StimTpar-GBst                  -0.02108    0.04011  -0.526   0.6011  
StimTpre-GBst                  -0.02027    0.04011  -0.505   0.6152  
EF_front                       -0.08691    0.33761  -0.257   0.7977  
EF_par                         -0.40167    0.52741  -0.762   0.4493  
StimTPre-GPar:EF_front          0.39133    0.47745   0.820   0.4157  
StimTpar-GBst:EF_front          0.07532    0.36940   0.204   0.8391  
StimTpre-GBst:EF_front          0.05841    0.36940   0.158   0.8749  
StimTPre-GPar:EF_par            1.52921    0.74587   2.050   0.0447 *
StimTpar-GBst:EF_par            0.34106    0.65284   0.522   0.6033  
StimTpre-GBst:EF_par            0.47539    0.65284   0.728   0.4693  
EF_front:EF_par                 4.23232    4.18244   1.012   0.3156  
StimTPre-GPar:EF_front:EF_par -10.83956    5.91486  -1.833   0.0718 .
StimTpar-GBst:EF_front:EF_par  -4.12534    4.35113  -0.948   0.3469  
StimTpre-GBst:EF_front:EF_par  -4.52153    4.35113  -1.039   0.3029  
---
Signif. codes:  0 ‘***’ 0.001 ‘**’ 0.01 ‘*’ 0.05 ‘.’ 0.1 ‘ ’ 1

Residual standard error: 0.02684 on 60 degrees of freedom
Multiple R-squared:  0.2283,    Adjusted R-squared:  0.0354 
F-statistic: 1.183 on 15 and 60 DF,  p-value: 0.3092
```


```
anova(m_acc_n_ef_2back)
```


```
Analysis of Variance Table

Response: Acc_n_m
                     Df   Sum Sq    Mean Sq F value  Pr(>F)  
Stim                  3 0.005545 0.00184818  2.5655 0.06291 .
EF_front              1 0.000006 0.00000612  0.0085 0.92690  
EF_par                1 0.000106 0.00010572  0.1468 0.70301  
Stim:EF_front         3 0.003065 0.00102173  1.4183 0.24627  
Stim:EF_par           3 0.001484 0.00049469  0.6867 0.56369  
EF_front:EF_par       1 0.000033 0.00003298  0.0458 0.83131  
Stim:EF_front:EF_par  3 0.002550 0.00085005  1.1800 0.32497  
Residuals            60 0.043224 0.00072040                  
---
Signif. codes:  0 ‘***’ 0.001 ‘**’ 0.01 ‘*’ 0.05 ‘.’ 0.1 ‘ ’ 1
```


```
data_subset <- subset(df_ef_epfl, Task == '3back' & Stim != 'Placebo')
data_subset <- droplevels(data_subset)
levels(data_subset$Task)
```


```
[1] "3back"
```


```
levels(data_subset$Stim)
```


```
[1] "GPre-TPar" "TPre-GPar" "Tpar-GBst" "Tpre-GBst"
```


```
m_acc_n_ef_3back <- lm(formula = Acc_n_m ~ Stim*EF_front*EF_par, data=data_subset)
summary(m_acc_n_ef_3back)
```


```
Call:
lm(formula = Acc_n_m ~ Stim * EF_front * EF_par, data = data_subset)

Residuals:
      Min        1Q    Median        3Q       Max 
-0.023528 -0.008848 -0.001358  0.006833  0.034642 

Coefficients:
                              Estimate Std. Error t value Pr(>|t|)   
(Intercept)                    0.01951    0.01416   1.378  0.17323   
StimTPre-GPar                 -0.04952    0.02002  -2.474  0.01622 * 
StimTpar-GBst                 -0.01297    0.01998  -0.649  0.51867   
StimTpre-GBst                 -0.01184    0.01998  -0.593  0.55564   
EF_front                      -0.33171    0.16818  -1.972  0.05318 . 
EF_par                        -0.25842    0.26273  -0.984  0.32928   
StimTPre-GPar:EF_front         0.62984    0.23784   2.648  0.01033 * 
StimTpar-GBst:EF_front         0.38659    0.18402   2.101  0.03987 * 
StimTpre-GBst:EF_front         0.21472    0.18402   1.167  0.24790   
StimTPre-GPar:EF_par           0.89211    0.37156   2.401  0.01947 * 
StimTpar-GBst:EF_par           0.14133    0.32522   0.435  0.66544   
StimTpre-GBst:EF_par           0.15113    0.32522   0.465  0.64382   
EF_front:EF_par                4.17921    2.08352   2.006  0.04939 * 
StimTPre-GPar:EF_front:EF_par -9.36867    2.94654  -3.180  0.00233 **
StimTpar-GBst:EF_front:EF_par -4.13436    2.16755  -1.907  0.06126 . 
StimTpre-GBst:EF_front:EF_par -3.22403    2.16755  -1.487  0.14214   
---
Signif. codes:  0 ‘***’ 0.001 ‘**’ 0.01 ‘*’ 0.05 ‘.’ 0.1 ‘ ’ 1

Residual standard error: 0.01337 on 60 degrees of freedom
Multiple R-squared:  0.2823,    Adjusted R-squared:  0.1028 
F-statistic: 1.573 on 15 and 60 DF,  p-value: 0.1091
```


```
anova(m_acc_n_ef_3back)
```


```
Analysis of Variance Table

Response: Acc_n_m
                     Df    Sum Sq    Mean Sq F value Pr(>F)  
Stim                  3 0.0002162 0.00007207  0.4031 0.7513  
EF_front              1 0.0002296 0.00022956  1.2841 0.2617  
EF_par                1 0.0002331 0.00023313  1.3041 0.2580  
Stim:EF_front         3 0.0002545 0.00008483  0.4745 0.7012  
Stim:EF_par           3 0.0009992 0.00033306  1.8630 0.1455  
EF_front:EF_par       1 0.0001944 0.00019437  1.0872 0.3013  
Stim:EF_front:EF_par  3 0.0020916 0.00069719  3.8998 0.0130 *
Residuals            60 0.0107265 0.00017878                 
---
Signif. codes:  0 ‘***’ 0.001 ‘**’ 0.01 ‘*’ 0.05 ‘.’ 0.1 ‘ ’ 1
```


#### Explore the triple interaction


```
n_rows = 100
choose_model <- m_acc_n_ef_3back
stim_list <- levels(data_subset$Stim)
i_start = 0
i_stop = 0.3

columns = c("Stim","Task","EF_front", "EF_par") 
df_test = data.frame(matrix(nrow = n_rows, ncol = length(columns))) 
colnames(df_test) = columns

columns = c("Stim","Task","EF_front", "EF_par") 
df_temp = data.frame(matrix(nrow = n_rows, ncol = length(columns))) 
colnames(df_temp) = columns

for (s in stim_list){
  for (t in c("3back")){
    if (s == stim_list[1] & t == '3back'){
    df_test$Stim <- rep(s, n_rows)
    df_test$EF_front <- rep(seq(i_start, i_stop, length.out = 10), 10)
    df_test$EF_par <- rep(seq(i_start, i_stop, length.out = 10), each=10)
    #df_test$Task <- sample(c('2back', '3back'), 100, replace=TRUE)
    df_test$Task <- rep(t, 100)
    }
    else{
    df_temp$Stim <- rep(s, n_rows)
    df_temp$EF_front <- rep(seq(i_start, i_stop, length.out = 10), 10)
    df_temp$EF_par <- rep(seq(i_start, i_stop, length.out = 10), each=10)
    #df_temp$Task <- sample(c('2back', '3back'), 100, replace=TRUE)
    df_temp$Task <- rep(t, 100)
    df_test <- rbind(df_test, df_temp)
    }
  }
}
```


```
# Make predictions
preds <- predict(choose_model, df_test)
df_test$Pred <- preds
```


```
# Viewing angles for the plots
angle_columns = c("Stim","Azim","Colatitude") 
df_angles = data.frame(matrix(nrow = 4, ncol = length(angle_columns))) 
colnames(df_angles) = angle_columns

df_angles$Stim <- stim_list
df_angles$Azim <- c(0, 0, 0, 0) # Theta, default 0
df_angles$Colatitude <- c(15, 15, 15, 15) # Phi, default 15
```


```
# Make plots
for (s in stim_list){
  for (t in c("3back")){
    disp_df <- subset(df_test, Stim == s & Task == t)
    persp(x=seq(i_start, i_stop, length.out = 10), y=seq(i_start, i_stop, length.out = 10), z=matrix(disp_df$Pred, nrow=10, ncol=10,byrow=TRUE), 
          theta = subset(df_angles, Stim == s)$Azim, phi = subset(df_angles, Stim == s)$Colatitude,
          xlab="EF_front",ylab="EF_par",zlab="Pred_Acc_n_m", main=paste(s,t), zlim=c(-0.16, 0.2)) 
  }
}
```


```
Warning: surface extends beyond the box
```

### Symmetry


```
data_subset <- subset(df_ef_epfl, Stim != 'Placebo')
data_subset <- droplevels(data_subset)
levels(data_subset$Stim)
```


```
[1] "GPre-TPar" "TPre-GPar" "Tpar-GBst" "Tpre-GBst"
```


```
m0 <- lm(formula = Acc_n_m ~ Task*Stim*EF_sym, data=data_subset)
summary(m0)
```


```
Call:
lm(formula = Acc_n_m ~ Task * Stim * EF_sym, data = data_subset)

Residuals:
      Min        1Q    Median        3Q       Max 
-0.036133 -0.011175 -0.004397  0.008358  0.130186 

Coefficients:
                                 Estimate Std. Error t value Pr(>|t|)  
(Intercept)                     0.0101916  0.0124855   0.816   0.4158  
Task3back                      -0.0006159  0.0176572  -0.035   0.9722  
StimTPre-GPar                   0.0272899  0.0176572   1.546   0.1245  
StimTpar-GBst                  -0.0056842  0.0180856  -0.314   0.7538  
StimTpre-GBst                  -0.0033442  0.0180856  -0.185   0.8536  
EF_sym                          0.0076646  0.0078916   0.971   0.3332  
Task3back:StimTPre-GPar        -0.0276450  0.0249710  -1.107   0.2702  
Task3back:StimTpar-GBst        -0.0096396  0.0255770  -0.377   0.7068  
Task3back:StimTpre-GBst         0.0009052  0.0255770   0.035   0.9718  
Task3back:EF_sym               -0.0131408  0.0111604  -1.177   0.2411  
StimTPre-GPar:EF_sym           -0.0217467  0.0111604  -1.949   0.0534 .
StimTpar-GBst:EF_sym           -0.0102213  0.0113021  -0.904   0.3674  
StimTpre-GBst:EF_sym           -0.0092975  0.0113021  -0.823   0.4122  
Task3back:StimTPre-GPar:EF_sym  0.0217858  0.0157832   1.380   0.1698  
Task3back:StimTpar-GBst:EF_sym  0.0220515  0.0159836   1.380   0.1700  
Task3back:StimTpre-GBst:EF_sym  0.0092173  0.0159836   0.577   0.5651  
---
Signif. codes:  0 ‘***’ 0.001 ‘**’ 0.01 ‘*’ 0.05 ‘.’ 0.1 ‘ ’ 1

Residual standard error: 0.0214 on 136 degrees of freedom
Multiple R-squared:  0.1659,    Adjusted R-squared:  0.07396 
F-statistic: 1.804 on 15 and 136 DF,  p-value: 0.03986
```


```
anova(m0)
```


```
Analysis of Variance Table

Response: Acc_n_m
                  Df   Sum Sq   Mean Sq F value   Pr(>F)   
Task               1 0.003744 0.0037439  8.1723 0.004924 **
Stim               3 0.002773 0.0009242  2.0174 0.114414   
EF_sym             1 0.000398 0.0003983  0.8695 0.352759   
Task:Stim          3 0.002988 0.0009960  2.1741 0.093934 . 
Task:EF_sym        1 0.000000 0.0000001  0.0001 0.991014   
Stim:EF_sym        3 0.001252 0.0004173  0.9110 0.437569   
Task:Stim:EF_sym   3 0.001242 0.0004139  0.9034 0.441333   
Residuals        136 0.062305 0.0004581                    
---
Signif. codes:  0 ‘***’ 0.001 ‘**’ 0.01 ‘*’ 0.05 ‘.’ 0.1 ‘ ’ 1
```


```
m1 <- lmer(formula = Acc_n_m ~ Task*Stim*EF_sym + (1 | ID), data=data_subset)
summary(m1)
```


```
Linear mixed model fit by REML. t-tests use Satterthwaite's method ['lmerModLmerTest']
Formula: Acc_n_m ~ Task * Stim * EF_sym + (1 | ID)
   Data: data_subset

REML criterion at convergence: -621

Scaled residuals: 
    Min      1Q  Median      3Q     Max 
-1.6353 -0.4721 -0.1992  0.3796  5.8889 

Random effects:
 Groups   Name        Variance  Std.Dev.
 ID       (Intercept) 1.828e-05 0.004276
 Residual             4.398e-04 0.020971
Number of obs: 152, groups:  ID, 20

Fixed effects:
                                 Estimate Std. Error         df t value Pr(>|t|)  
(Intercept)                     1.019e-02  1.248e-02  1.346e+02   0.816    0.416  
Task3back                      -6.159e-04  1.730e-02  1.188e+02  -0.036    0.972  
StimTPre-GPar                   2.729e-02  1.730e-02  1.188e+02   1.577    0.117  
StimTpar-GBst                  -5.907e-03  1.773e-02  1.201e+02  -0.333    0.740  
StimTpre-GBst                  -3.567e-03  1.773e-02  1.201e+02  -0.201    0.841  
EF_sym                          7.665e-03  7.891e-03  1.346e+02   0.971    0.333  
Task3back:StimTPre-GPar        -2.764e-02  2.447e-02  1.188e+02  -1.130    0.261  
Task3back:StimTpar-GBst        -9.640e-03  2.506e-02  1.188e+02  -0.385    0.701  
Task3back:StimTpre-GBst         9.052e-04  2.506e-02  1.188e+02   0.036    0.971  
Task3back:EF_sym               -1.314e-02  1.093e-02  1.188e+02  -1.202    0.232  
StimTPre-GPar:EF_sym           -2.175e-02  1.093e-02  1.188e+02  -1.989    0.049 *
StimTpar-GBst:EF_sym           -1.009e-02  1.108e-02  1.195e+02  -0.911    0.364  
StimTpre-GBst:EF_sym           -9.170e-03  1.108e-02  1.195e+02  -0.828    0.409  
Task3back:StimTPre-GPar:EF_sym  2.179e-02  1.546e-02  1.188e+02   1.409    0.162  
Task3back:StimTpar-GBst:EF_sym  2.205e-02  1.566e-02  1.188e+02   1.408    0.162  
Task3back:StimTpre-GBst:EF_sym  9.217e-03  1.566e-02  1.188e+02   0.589    0.557  
---
Signif. codes:  0 ‘***’ 0.001 ‘**’ 0.01 ‘*’ 0.05 ‘.’ 0.1 ‘ ’ 1
```


```
Correlation matrix not shown by default, as p = 16 > 12.
Use print(x, correlation=TRUE)  or
    vcov(x)        if you need it
```


```
anova(m1)
```


```
Type III Analysis of Variance Table with Satterthwaite's method
                     Sum Sq    Mean Sq NumDF   DenDF F value Pr(>F)
Task             0.00052830 0.00052830     1 118.785  1.2013 0.2753
Stim             0.00172566 0.00057522     3 120.532  1.3080 0.2750
EF_sym           0.00027595 0.00027595     1  17.496  0.6275 0.4389
Task:Stim        0.00076109 0.00025370     3 118.785  0.5769 0.6313
Task:EF_sym      0.00000022 0.00000022     1 118.785  0.0005 0.9823
Stim:EF_sym      0.00126429 0.00042143     3 119.734  0.9583 0.4149
Task:Stim:EF_sym 0.00124167 0.00041389     3 118.785  0.9411 0.4232
```


```
AIC(m0, m1)
```


```
BIC(m0, m1)
```


```
m_acc_n_ef_sym <- lm(formula = Acc_n_m ~ Task*Stim*EF_sym, data=data_subset)
summary(m_acc_n_ef_sym)
```


```
Call:
lm(formula = Acc_n_m ~ Task * Stim * EF_sym, data = data_subset)

Residuals:
      Min        1Q    Median        3Q       Max 
-0.036133 -0.011175 -0.004397  0.008358  0.130186 

Coefficients:
                                 Estimate Std. Error t value Pr(>|t|)  
(Intercept)                     0.0101916  0.0124855   0.816   0.4158  
Task3back                      -0.0006159  0.0176572  -0.035   0.9722  
StimTPre-GPar                   0.0272899  0.0176572   1.546   0.1245  
StimTpar-GBst                  -0.0056842  0.0180856  -0.314   0.7538  
StimTpre-GBst                  -0.0033442  0.0180856  -0.185   0.8536  
EF_sym                          0.0076646  0.0078916   0.971   0.3332  
Task3back:StimTPre-GPar        -0.0276450  0.0249710  -1.107   0.2702  
Task3back:StimTpar-GBst        -0.0096396  0.0255770  -0.377   0.7068  
Task3back:StimTpre-GBst         0.0009052  0.0255770   0.035   0.9718  
Task3back:EF_sym               -0.0131408  0.0111604  -1.177   0.2411  
StimTPre-GPar:EF_sym           -0.0217467  0.0111604  -1.949   0.0534 .
StimTpar-GBst:EF_sym           -0.0102213  0.0113021  -0.904   0.3674  
StimTpre-GBst:EF_sym           -0.0092975  0.0113021  -0.823   0.4122  
Task3back:StimTPre-GPar:EF_sym  0.0217858  0.0157832   1.380   0.1698  
Task3back:StimTpar-GBst:EF_sym  0.0220515  0.0159836   1.380   0.1700  
Task3back:StimTpre-GBst:EF_sym  0.0092173  0.0159836   0.577   0.5651  
---
Signif. codes:  0 ‘***’ 0.001 ‘**’ 0.01 ‘*’ 0.05 ‘.’ 0.1 ‘ ’ 1

Residual standard error: 0.0214 on 136 degrees of freedom
Multiple R-squared:  0.1659,    Adjusted R-squared:  0.07396 
F-statistic: 1.804 on 15 and 136 DF,  p-value: 0.03986
```


```
anova(m_acc_n_ef_sym)
```


```
Analysis of Variance Table

Response: Acc_n_m
                  Df   Sum Sq   Mean Sq F value   Pr(>F)   
Task               1 0.003744 0.0037439  8.1723 0.004924 **
Stim               3 0.002773 0.0009242  2.0174 0.114414   
EF_sym             1 0.000398 0.0003983  0.8695 0.352759   
Task:Stim          3 0.002988 0.0009960  2.1741 0.093934 . 
Task:EF_sym        1 0.000000 0.0000001  0.0001 0.991014   
Stim:EF_sym        3 0.001252 0.0004173  0.9110 0.437569   
Task:Stim:EF_sym   3 0.001242 0.0004139  0.9034 0.441333   
Residuals        136 0.062305 0.0004581                    
---
Signif. codes:  0 ‘***’ 0.001 ‘**’ 0.01 ‘*’ 0.05 ‘.’ 0.1 ‘ ’ 1
```


```
data_subset <- subset(df_ef_epfl, Task == '2back' & Stim != 'Placebo')
data_subset <- droplevels(data_subset)
levels(data_subset$Task)
```


```
[1] "2back"
```


```
levels(data_subset$Stim)
```


```
[1] "GPre-TPar" "TPre-GPar" "Tpar-GBst" "Tpre-GBst"
```


```
m_acc_n_ef_sym_2back <- lm(formula = Acc_n_m ~ Stim*EF_sym, data=data_subset)
summary(m_acc_n_ef_sym_2back)
```


```
Call:
lm(formula = Acc_n_m ~ Stim * EF_sym, data = data_subset)

Residuals:
      Min        1Q    Median        3Q       Max 
-0.036133 -0.013646 -0.005958  0.008358  0.130186 

Coefficients:
                      Estimate Std. Error t value Pr(>|t|)
(Intercept)           0.010192   0.015581   0.654    0.515
StimTPre-GPar         0.027290   0.022035   1.239    0.220
StimTpar-GBst        -0.005684   0.022569  -0.252    0.802
StimTpre-GBst        -0.003344   0.022569  -0.148    0.883
EF_sym                0.007665   0.009848   0.778    0.439
StimTPre-GPar:EF_sym -0.021747   0.013927  -1.561    0.123
StimTpar-GBst:EF_sym -0.010221   0.014104  -0.725    0.471
StimTpre-GBst:EF_sym -0.009298   0.014104  -0.659    0.512

Residual standard error: 0.02671 on 68 degrees of freedom
Multiple R-squared:  0.1339,    Adjusted R-squared:  0.04474 
F-statistic: 1.502 on 7 and 68 DF,  p-value: 0.1815
```


```
anova(m_acc_n_ef_sym_2back)
```


```
Analysis of Variance Table

Response: Acc_n_m
            Df   Sum Sq    Mean Sq F value  Pr(>F)  
Stim         3 0.005545 0.00184818  2.5906 0.05986 .
EF_sym       1 0.000204 0.00020401  0.2860 0.59457  
Stim:EF_sym  3 0.001751 0.00058378  0.8183 0.48823  
Residuals   68 0.048513 0.00071342                  
---
Signif. codes:  0 ‘***’ 0.001 ‘**’ 0.01 ‘*’ 0.05 ‘.’ 0.1 ‘ ’ 1
```


```
data_subset <- subset(df_ef_epfl, Task == '3back' & Stim != 'Placebo')
data_subset <- droplevels(data_subset)
levels(data_subset$Task)
```


```
[1] "3back"
```


```
levels(data_subset$Stim)
```


```
[1] "GPre-TPar" "TPre-GPar" "Tpar-GBst" "Tpre-GBst"
```


```
m_acc_n_ef_sym_3back <- lm(formula = Acc_n_m ~ Stim*EF_sym, data=data_subset)
summary(m_acc_n_ef_sym_3back)
```


```
Call:
lm(formula = Acc_n_m ~ Stim * EF_sym, data = data_subset)

Residuals:
      Min        1Q    Median        3Q       Max 
-0.025665 -0.008292 -0.003047  0.008396  0.039920 

Coefficients:
                       Estimate Std. Error t value Pr(>|t|)
(Intercept)           9.576e-03  8.308e-03   1.153    0.253
StimTPre-GPar        -3.551e-04  1.175e-02  -0.030    0.976
StimTpar-GBst        -1.532e-02  1.203e-02  -1.273    0.207
StimTpre-GBst        -2.439e-03  1.203e-02  -0.203    0.840
EF_sym               -5.476e-03  5.251e-03  -1.043    0.301
StimTPre-GPar:EF_sym  3.902e-05  7.426e-03   0.005    0.996
StimTpar-GBst:EF_sym  1.183e-02  7.520e-03   1.573    0.120
StimTpre-GBst:EF_sym -8.019e-05  7.520e-03  -0.011    0.992

Residual standard error: 0.01424 on 68 degrees of freedom
Multiple R-squared:  0.07714,   Adjusted R-squared:  -0.01786 
F-statistic: 0.812 on 7 and 68 DF,  p-value: 0.5804
```


```
anova(m_acc_n_ef_sym_3back)
```


```
Analysis of Variance Table

Response: Acc_n_m
            Df    Sum Sq    Mean Sq F value Pr(>F)
Stim         3 0.0002162 7.2071e-05  0.3553 0.7854
EF_sym       1 0.0001944 1.9437e-04  0.9583 0.3311
Stim:EF_sym  3 0.0007423 2.4744e-04  1.2200 0.3092
Residuals   68 0.0137922 2.0283e-04
```

### Normalized accuracy and magnitude of combined electric fields


```
data_subset <- subset(df_ef_epfl, Stim != 'Placebo')
data_subset <- droplevels(data_subset)
levels(data_subset$Stim)
```


```
[1] "GPre-TPar" "TPre-GPar" "Tpar-GBst" "Tpre-GBst"
```


```
m0 <- lm(formula = Acc_n_m ~ Task*Stim*EF_mag, data=data_subset)
summary(m0)
```


```
Call:
lm(formula = Acc_n_m ~ Task * Stim * EF_mag, data = data_subset)

Residuals:
     Min       1Q   Median       3Q      Max 
-0.03612 -0.01193 -0.00361  0.00801  0.12810 

Coefficients:
                                Estimate Std. Error t value Pr(>|t|)  
(Intercept)                     0.002035   0.010344   0.197   0.8443  
Task3back                      -0.005333   0.014629  -0.365   0.7160  
StimTPre-GPar                   0.006824   0.014629   0.467   0.6416  
StimTpar-GBst                   0.001868   0.014246   0.131   0.8959  
StimTpre-GBst                   0.012033   0.014246   0.845   0.3998  
EF_mag                          0.191365   0.090823   2.107   0.0370 *
Task3back:StimTPre-GPar        -0.002256   0.020688  -0.109   0.9133  
Task3back:StimTpar-GBst         0.005504   0.020147   0.273   0.7851  
Task3back:StimTpre-GBst        -0.019262   0.020147  -0.956   0.3407  
Task3back:EF_mag               -0.143207   0.128444  -1.115   0.2668  
StimTPre-GPar:EF_mag           -0.111840   0.128444  -0.871   0.3854  
StimTpar-GBst:EF_mag           -0.208087   0.100848  -2.063   0.0410 *
StimTpre-GBst:EF_mag           -0.241663   0.100848  -2.396   0.0179 *
Task3back:StimTPre-GPar:EF_mag  0.063727   0.181647   0.351   0.7263  
Task3back:StimTpar-GBst:EF_mag  0.158153   0.142621   1.109   0.2694  
Task3back:StimTpre-GBst:EF_mag  0.242314   0.142621   1.699   0.0916 .
---
Signif. codes:  0 ‘***’ 0.001 ‘**’ 0.01 ‘*’ 0.05 ‘.’ 0.1 ‘ ’ 1

Residual standard error: 0.02126 on 136 degrees of freedom
Multiple R-squared:  0.1768,    Adjusted R-squared:  0.08602 
F-statistic: 1.947 on 15 and 136 DF,  p-value: 0.02357
```


```
anova(m0)
```


```
Analysis of Variance Table

Response: Acc_n_m
                  Df   Sum Sq   Mean Sq F value   Pr(>F)   
Task               1 0.003744 0.0037439  8.2802 0.004656 **
Stim               3 0.002773 0.0009242  2.0440 0.110651   
EF_mag             1 0.000141 0.0001409  0.3116 0.577617   
Task:Stim          3 0.002988 0.0009960  2.2028 0.090594 . 
Task:EF_mag        1 0.000185 0.0001845  0.4081 0.524029   
Stim:EF_mag        3 0.001646 0.0005485  1.2131 0.307451   
Task:Stim:EF_mag   3 0.001733 0.0005776  1.2774 0.284715   
Residuals        136 0.061493 0.0004522                    
---
Signif. codes:  0 ‘***’ 0.001 ‘**’ 0.01 ‘*’ 0.05 ‘.’ 0.1 ‘ ’ 1
```


```
m1 <- lmer(formula = Acc_n_m ~ Task*Stim*EF_mag + (1 | ID), data=data_subset)
summary(m1)
```


```
Linear mixed model fit by REML. t-tests use Satterthwaite's method ['lmerModLmerTest']
Formula: Acc_n_m ~ Task * Stim * EF_mag + (1 | ID)
   Data: data_subset

REML criterion at convergence: -655.8

Scaled residuals: 
    Min      1Q  Median      3Q     Max 
-1.6181 -0.5286 -0.1341  0.3641  5.8632 

Random effects:
 Groups   Name        Variance  Std.Dev.
 ID       (Intercept) 1.561e-05 0.003951
 Residual             4.365e-04 0.020893
Number of obs: 152, groups:  ID, 20

Fixed effects:
                                 Estimate Std. Error         df t value Pr(>|t|)  
(Intercept)                      0.002105   0.010341 135.207939   0.204   0.8390  
Task3back                       -0.005333   0.014374 118.485024  -0.371   0.7113  
StimTPre-GPar                    0.006824   0.014374 118.485025   0.475   0.6358  
StimTpar-GBst                    0.001202   0.014023 121.628385   0.086   0.9318  
StimTpre-GBst                    0.011367   0.014023 121.628385   0.811   0.4191  
EF_mag                           0.190680   0.090793 135.274854   2.100   0.0376 *
Task3back:StimTPre-GPar         -0.002256   0.020328 118.485024  -0.111   0.9118  
Task3back:StimTpar-GBst          0.005504   0.019796 118.485024   0.278   0.7815  
Task3back:StimTpre-GBst         -0.019262   0.019796 118.485024  -0.973   0.3325  
Task3back:EF_mag                -0.143207   0.126204 118.485024  -1.135   0.2588  
StimTPre-GPar:EF_mag            -0.111840   0.126204 118.485025  -0.886   0.3773  
StimTpar-GBst:EF_mag            -0.204180   0.099575 127.526741  -2.051   0.0424 *
StimTpre-GBst:EF_mag            -0.237757   0.099575 127.526741  -2.388   0.0184 *
Task3back:StimTPre-GPar:EF_mag   0.063727   0.178480 118.485024   0.357   0.7217  
Task3back:StimTpar-GBst:EF_mag   0.158153   0.140134 118.485024   1.129   0.2614  
Task3back:StimTpre-GBst:EF_mag   0.242314   0.140134 118.485024   1.729   0.0864 .
---
Signif. codes:  0 ‘***’ 0.001 ‘**’ 0.01 ‘*’ 0.05 ‘.’ 0.1 ‘ ’ 1
```


```
Correlation matrix not shown by default, as p = 16 > 12.
Use print(x, correlation=TRUE)  or
    vcov(x)        if you need it
```


```
anova(m1)
```


```
Type III Analysis of Variance Table with Satterthwaite's method
                     Sum Sq    Mean Sq NumDF   DenDF F value Pr(>F)
Task             0.00077681 0.00077681     1 118.485  1.7795 0.1848
Stim             0.00016029 0.00005343     3 122.367  0.1224 0.9467
EF_mag           0.00085629 0.00085629     1  22.424  1.9616 0.1750
Task:Stim        0.00080157 0.00026719     3 118.485  0.6121 0.6085
Task:EF_mag      0.00013117 0.00013117     1 118.485  0.3005 0.5846
Stim:EF_mag      0.00148645 0.00049548     3 126.295  1.1351 0.3376
Task:Stim:EF_mag 0.00173269 0.00057756     3 118.485  1.3231 0.2702
```


```
AIC(m0, m1)
```


```
BIC(m0, m1)
```


```
m_acc_n_ef_mag <- lm(formula = Acc_n_m ~ Task*Stim*EF_mag, data=data_subset)
summary(m_acc_n_ef_mag)
```


```
Call:
lm(formula = Acc_n_m ~ Task * Stim * EF_mag, data = data_subset)

Residuals:
     Min       1Q   Median       3Q      Max 
-0.03612 -0.01193 -0.00361  0.00801  0.12810 

Coefficients:
                                Estimate Std. Error t value Pr(>|t|)  
(Intercept)                     0.002035   0.010344   0.197   0.8443  
Task3back                      -0.005333   0.014629  -0.365   0.7160  
StimTPre-GPar                   0.006824   0.014629   0.467   0.6416  
StimTpar-GBst                   0.001868   0.014246   0.131   0.8959  
StimTpre-GBst                   0.012033   0.014246   0.845   0.3998  
EF_mag                          0.191365   0.090823   2.107   0.0370 *
Task3back:StimTPre-GPar        -0.002256   0.020688  -0.109   0.9133  
Task3back:StimTpar-GBst         0.005504   0.020147   0.273   0.7851  
Task3back:StimTpre-GBst        -0.019262   0.020147  -0.956   0.3407  
Task3back:EF_mag               -0.143207   0.128444  -1.115   0.2668  
StimTPre-GPar:EF_mag           -0.111840   0.128444  -0.871   0.3854  
StimTpar-GBst:EF_mag           -0.208087   0.100848  -2.063   0.0410 *
StimTpre-GBst:EF_mag           -0.241663   0.100848  -2.396   0.0179 *
Task3back:StimTPre-GPar:EF_mag  0.063727   0.181647   0.351   0.7263  
Task3back:StimTpar-GBst:EF_mag  0.158153   0.142621   1.109   0.2694  
Task3back:StimTpre-GBst:EF_mag  0.242314   0.142621   1.699   0.0916 .
---
Signif. codes:  0 ‘***’ 0.001 ‘**’ 0.01 ‘*’ 0.05 ‘.’ 0.1 ‘ ’ 1

Residual standard error: 0.02126 on 136 degrees of freedom
Multiple R-squared:  0.1768,    Adjusted R-squared:  0.08602 
F-statistic: 1.947 on 15 and 136 DF,  p-value: 0.02357
```


```
anova(m_acc_n_ef_mag)
```


```
Analysis of Variance Table

Response: Acc_n_m
                  Df   Sum Sq   Mean Sq F value   Pr(>F)   
Task               1 0.003744 0.0037439  8.2802 0.004656 **
Stim               3 0.002773 0.0009242  2.0440 0.110651   
EF_mag             1 0.000141 0.0001409  0.3116 0.577617   
Task:Stim          3 0.002988 0.0009960  2.2028 0.090594 . 
Task:EF_mag        1 0.000185 0.0001845  0.4081 0.524029   
Stim:EF_mag        3 0.001646 0.0005485  1.2131 0.307451   
Task:Stim:EF_mag   3 0.001733 0.0005776  1.2774 0.284715   
Residuals        136 0.061493 0.0004522                    
---
Signif. codes:  0 ‘***’ 0.001 ‘**’ 0.01 ‘*’ 0.05 ‘.’ 0.1 ‘ ’ 1
```


```
data_subset <- subset(df_ef_epfl, Stim != 'Placebo' & Task == '2back')
data_subset <- droplevels(data_subset)
levels(data_subset$Stim)
```


```
[1] "GPre-TPar" "TPre-GPar" "Tpar-GBst" "Tpre-GBst"
```


```
levels(data_subset$Task)
```


```
[1] "2back"
```


```
m_acc_n_ef_mag_2back <- lm(formula = Acc_n_m ~ Stim*EF_mag, data=data_subset)
summary(m_acc_n_ef_mag_2back)
```


```
Call:
lm(formula = Acc_n_m ~ Stim * EF_mag, data = data_subset)

Residuals:
      Min        1Q    Median        3Q       Max 
-0.036116 -0.012635 -0.004886  0.007364  0.128103 

Coefficients:
                      Estimate Std. Error t value Pr(>|t|)  
(Intercept)           0.002035   0.012851   0.158   0.8746  
StimTPre-GPar         0.006824   0.018174   0.376   0.7085  
StimTpar-GBst         0.001868   0.017699   0.106   0.9163  
StimTpre-GBst         0.012033   0.017699   0.680   0.4989  
EF_mag                0.191365   0.112832   1.696   0.0945 .
StimTPre-GPar:EF_mag -0.111840   0.159568  -0.701   0.4858  
StimTpar-GBst:EF_mag -0.208087   0.125285  -1.661   0.1013  
StimTpre-GBst:EF_mag -0.241663   0.125285  -1.929   0.0579 .
---
Signif. codes:  0 ‘***’ 0.001 ‘**’ 0.01 ‘*’ 0.05 ‘.’ 0.1 ‘ ’ 1

Residual standard error: 0.02642 on 68 degrees of freedom
Multiple R-squared:  0.1528,    Adjusted R-squared:  0.06561 
F-statistic: 1.752 on 7 and 68 DF,  p-value: 0.1115
```


```
anova(m_acc_n_ef_mag_2back)
```


```
Analysis of Variance Table

Response: Acc_n_m
            Df   Sum Sq    Mean Sq F value  Pr(>F)  
Stim         3 0.005545 0.00184818  2.6484 0.05582 .
EF_mag       1 0.000001 0.00000147  0.0021 0.96355  
Stim:EF_mag  3 0.003014 0.00100456  1.4395 0.23892  
Residuals   68 0.047453 0.00069784                  
---
Signif. codes:  0 ‘***’ 0.001 ‘**’ 0.01 ‘*’ 0.05 ‘.’ 0.1 ‘ ’ 1
```


```
data_subset <- subset(df_ef_epfl, Stim != 'Placebo' & Task == '3back')
data_subset <- droplevels(data_subset)
levels(data_subset$Stim)
```


```
[1] "GPre-TPar" "TPre-GPar" "Tpar-GBst" "Tpre-GBst"
```


```
levels(data_subset$Task)
```


```
[1] "3back"
```


```
m_acc_n_ef_mag_3back <- lm(formula = Acc_n_m ~ Stim*EF_mag, data=data_subset)
summary(m_acc_n_ef_mag_3back)
```


```
Call:
lm(formula = Acc_n_m ~ Stim * EF_mag, data = data_subset)

Residuals:
      Min        1Q    Median        3Q       Max 
-0.024957 -0.009218 -0.002388  0.010814  0.034990 

Coefficients:
                       Estimate Std. Error t value Pr(>|t|)
(Intercept)          -0.0032977  0.0069901  -0.472    0.639
StimTPre-GPar         0.0045685  0.0098855   0.462    0.645
StimTpar-GBst         0.0073716  0.0096271   0.766    0.446
StimTpre-GBst        -0.0072286  0.0096271  -0.751    0.455
EF_mag                0.0481580  0.0613745   0.785    0.435
StimTPre-GPar:EF_mag -0.0481134  0.0867967  -0.554    0.581
StimTpar-GBst:EF_mag -0.0499338  0.0681486  -0.733    0.466
StimTpre-GBst:EF_mag  0.0006509  0.0681486   0.010    0.992

Residual standard error: 0.01437 on 68 degrees of freedom
Multiple R-squared:  0.06053,   Adjusted R-squared:  -0.03617 
F-statistic: 0.6259 on 7 and 68 DF,  p-value: 0.7327
```


```
anova(m_acc_n_ef_mag_3back)
```


```
Analysis of Variance Table

Response: Acc_n_m
            Df    Sum Sq    Mean Sq F value Pr(>F)
Stim         3 0.0002162 0.00007207  0.3491 0.7899
EF_mag       1 0.0003239 0.00032393  1.5689 0.2147
Stim:EF_mag  3 0.0003646 0.00012152  0.5885 0.6246
Residuals   68 0.0140404 0.00020648
```

### The relationship between the intensity of the applied electric field on the target and the response time (i.e., the speed)


```
data_subset <- subset(df_ef_epfl, Stim != 'Placebo')
data_subset <- droplevels(data_subset)
levels(data_subset$Stim)
```


```
[1] "GPre-TPar" "TPre-GPar" "Tpar-GBst" "Tpre-GBst"
```


```
m0 <- lm(formula = Speed_m ~ Task*Stim*EF_front*EF_par, data=data_subset)
summary(m0)
```


```
Call:
lm(formula = Speed_m ~ Task * Stim * EF_front * EF_par, data = data_subset)

Residuals:
    Min      1Q  Median      3Q     Max 
-54.629  -7.280   0.399   9.061  40.633 

Coefficients:
                                         Estimate Std. Error t value Pr(>|t|)
(Intercept)                                -6.139     17.832  -0.344    0.731
Task3back                                   1.326     25.219   0.053    0.958
StimTPre-GPar                              22.856     25.219   0.906    0.367
StimTpar-GBst                               0.188     25.169   0.007    0.994
StimTpre-GBst                              11.125     25.169   0.442    0.659
EF_front                                  -36.605    211.851  -0.173    0.863
EF_par                                   -337.462    330.957  -1.020    0.310
Task3back:StimTPre-GPar                   -35.002     35.665  -0.981    0.328
Task3back:StimTpar-GBst                    -8.569     35.594  -0.241    0.810
Task3back:StimTpre-GBst                    -5.398     35.594  -0.152    0.880
Task3back:EF_front                        -14.800    299.603  -0.049    0.961
StimTPre-GPar:EF_front                   -119.379    299.603  -0.398    0.691
StimTpar-GBst:EF_front                     98.675    231.805   0.426    0.671
StimTpre-GBst:EF_front                    -16.559    231.805  -0.071    0.943
Task3back:EF_par                          510.196    468.044   1.090    0.278
StimTPre-GPar:EF_par                     -412.668    468.044  -0.882    0.380
StimTpar-GBst:EF_par                      216.207    409.665   0.528    0.599
StimTpre-GBst:EF_par                      300.394    409.665   0.733    0.465
EF_front:EF_par                          2436.539   2624.524   0.928    0.355
Task3back:StimTPre-GPar:EF_front          139.006    423.703   0.328    0.743
Task3back:StimTpar-GBst:EF_front            5.929    327.822   0.018    0.986
Task3back:StimTpre-GBst:EF_front           14.576    327.822   0.044    0.965
Task3back:StimTPre-GPar:EF_par            571.240    661.914   0.863    0.390
Task3back:StimTpar-GBst:EF_par           -345.401    579.354  -0.596    0.552
Task3back:StimTpre-GBst:EF_par           -541.664    579.354  -0.935    0.352
Task3back:EF_front:EF_par               -3235.224   3711.638  -0.872    0.385
StimTPre-GPar:EF_front:EF_par            3585.815   3711.638   0.966    0.336
StimTpar-GBst:EF_front:EF_par           -2438.914   2730.379  -0.893    0.374
StimTpre-GBst:EF_front:EF_par           -2336.209   2730.379  -0.856    0.394
Task3back:StimTPre-GPar:EF_front:EF_par -4419.837   5249.048  -0.842    0.401
Task3back:StimTpar-GBst:EF_front:EF_par  3030.231   3861.339   0.785    0.434
Task3back:StimTpre-GBst:EF_front:EF_par  3733.532   3861.339   0.967    0.336

Residual standard error: 16.84 on 120 degrees of freedom
Multiple R-squared:  0.2144,    Adjusted R-squared:  0.0114 
F-statistic: 1.056 on 31 and 120 DF,  p-value: 0.402
```


```
anova(m0)
```


```
Analysis of Variance Table

Response: Speed_m
                           Df Sum Sq Mean Sq F value  Pr(>F)  
Task                        1    519  518.66  1.8284 0.17886  
Stim                        3    660  220.02  0.7756 0.50983  
EF_front                    1    159  159.08  0.5608 0.45541  
EF_par                      1      5    4.97  0.0175 0.89487  
Task:Stim                   3   1246  415.50  1.4647 0.22763  
Task:EF_front               1     14   14.37  0.0507 0.82230  
Stim:EF_front               3    323  107.60  0.3793 0.76809  
Task:EF_par                 1    812  812.46  2.8641 0.09317 .
Stim:EF_par                 3    318  105.96  0.3735 0.77225  
EF_front:EF_par             1    112  112.21  0.3956 0.53058  
Task:Stim:EF_front          3   3054 1018.04  3.5888 0.01579 *
Task:Stim:EF_par            3     97   32.28  0.1138 0.95188  
Task:EF_front:EF_par        1     42   42.01  0.1481 0.70103  
Stim:EF_front:EF_par        3    473  157.69  0.5559 0.64519  
Task:Stim:EF_front:EF_par   3   1453  484.27  1.7071 0.16919  
Residuals                 120  34041  283.67                  
---
Signif. codes:  0 ‘***’ 0.001 ‘**’ 0.01 ‘*’ 0.05 ‘.’ 0.1 ‘ ’ 1
```


```
m1 <- lmer(formula = Speed_m ~ Task*Stim*EF_front*EF_par + (1 | ID), data=data_subset)
summary(m1)
```


```
Linear mixed model fit by REML. t-tests use Satterthwaite's method ['lmerModLmerTest']
Formula: Speed_m ~ Task * Stim * EF_front * EF_par + (1 | ID)
   Data: data_subset

REML criterion at convergence: 917.5

Scaled residuals: 
    Min      1Q  Median      3Q     Max 
-3.1661 -0.4110 -0.0119  0.5734  2.4071 

Random effects:
 Groups   Name        Variance Std.Dev.
 ID       (Intercept)   8.245   2.871  
 Residual             275.329  16.593  
Number of obs: 152, groups:  ID, 20

Fixed effects:
                                          Estimate Std. Error         df t value Pr(>|t|)
(Intercept)                             -6.024e+00  1.781e+01  1.200e+02  -0.338    0.736
Task3back                                1.326e+00  2.485e+01  1.054e+02   0.053    0.958
StimTPre-GPar                            2.286e+01  2.485e+01  1.054e+02   0.920    0.360
StimTpar-GBst                            8.083e-02  2.496e+01  1.154e+02   0.003    0.997
StimTpre-GBst                            1.102e+01  2.496e+01  1.154e+02   0.441    0.660
EF_front                                -3.746e+01  2.117e+02  1.198e+02  -0.177    0.860
EF_par                                  -3.428e+02  3.305e+02  1.200e+02  -1.037    0.302
Task3back:StimTPre-GPar                 -3.500e+01  3.514e+01  1.054e+02  -0.996    0.321
Task3back:StimTpar-GBst                 -8.568e+00  3.507e+01  1.054e+02  -0.244    0.807
Task3back:StimTpre-GBst                 -5.398e+00  3.507e+01  1.054e+02  -0.154    0.878
Task3back:EF_front                      -1.480e+01  2.952e+02  1.054e+02  -0.050    0.960
StimTPre-GPar:EF_front                  -1.194e+02  2.952e+02  1.054e+02  -0.404    0.687
StimTpar-GBst:EF_front                   9.998e+01  2.296e+02  1.143e+02   0.436    0.664
StimTpre-GBst:EF_front                  -1.525e+01  2.296e+02  1.143e+02  -0.066    0.947
Task3back:EF_par                         5.102e+02  4.611e+02  1.054e+02   1.106    0.271
StimTPre-GPar:EF_par                    -4.127e+02  4.611e+02  1.054e+02  -0.895    0.373
StimTpar-GBst:EF_par                     2.203e+02  4.070e+02  1.172e+02   0.541    0.589
StimTpre-GBst:EF_par                     3.045e+02  4.070e+02  1.172e+02   0.748    0.456
EF_front:EF_par                          2.483e+03  2.622e+03  1.200e+02   0.947    0.345
Task3back:StimTPre-GPar:EF_front         1.390e+02  4.174e+02  1.054e+02   0.333    0.740
Task3back:StimTpar-GBst:EF_front         5.929e+00  3.230e+02  1.054e+02   0.018    0.985
Task3back:StimTpre-GBst:EF_front         1.458e+01  3.230e+02  1.054e+02   0.045    0.964
Task3back:StimTPre-GPar:EF_par           5.712e+02  6.521e+02  1.054e+02   0.876    0.383
Task3back:StimTpar-GBst:EF_par          -3.454e+02  5.708e+02  1.054e+02  -0.605    0.546
Task3back:StimTpre-GBst:EF_par          -5.417e+02  5.708e+02  1.054e+02  -0.949    0.345
Task3back:EF_front:EF_par               -3.235e+03  3.657e+03  1.054e+02  -0.885    0.378
StimTPre-GPar:EF_front:EF_par            3.586e+03  3.657e+03  1.054e+02   0.981    0.329
StimTpar-GBst:EF_front:EF_par           -2.482e+03  2.716e+03  1.187e+02  -0.914    0.363
StimTpre-GBst:EF_front:EF_par           -2.379e+03  2.716e+03  1.187e+02  -0.876    0.383
Task3back:StimTPre-GPar:EF_front:EF_par -4.420e+03  5.171e+03  1.054e+02  -0.855    0.395
Task3back:StimTpar-GBst:EF_front:EF_par  3.030e+03  3.804e+03  1.054e+02   0.797    0.427
Task3back:StimTpre-GBst:EF_front:EF_par  3.734e+03  3.804e+03  1.054e+02   0.981    0.329
```


```
Correlation matrix not shown by default, as p = 32 > 12.
Use print(x, correlation=TRUE)  or
    vcov(x)        if you need it
```


```
anova(m1)
```


```
Type III Analysis of Variance Table with Satterthwaite's method
                           Sum Sq Mean Sq NumDF   DenDF F value  Pr(>F)  
Task                       213.44  213.44     1 105.420  0.7752 0.38061  
Stim                       167.22   55.74     3 113.886  0.2025 0.89451  
EF_front                    79.98   79.98     1  23.529  0.2905 0.59497  
EF_par                     231.26  231.26     1  37.697  0.8399 0.36524  
Task:Stim                  325.03  108.34     3 105.420  0.3935 0.75793  
Task:EF_front               13.28   13.28     1 105.420  0.0482 0.82659  
Stim:EF_front              516.19  172.06     3 111.951  0.6249 0.60034  
Task:EF_par               1257.35 1257.35     1 105.420  4.5667 0.03491 *
Stim:EF_par                111.49   37.16     3 111.911  0.1350 0.93900  
EF_front:EF_par            375.13  375.13     1  38.646  1.3625 0.25026  
Task:Stim:EF_front          49.66   16.55     3 105.420  0.0601 0.98058  
Task:Stim:EF_par          1156.80  385.60     3 105.420  1.4005 0.24681  
Task:EF_front:EF_par      1068.25 1068.25     1 105.420  3.8799 0.05149 .
Stim:EF_front:EF_par       468.82  156.27     3 106.133  0.5676 0.63757  
Task:Stim:EF_front:EF_par 1452.81  484.27     3 105.420  1.7589 0.15956  
---
Signif. codes:  0 ‘***’ 0.001 ‘**’ 0.01 ‘*’ 0.05 ‘.’ 0.1 ‘ ’ 1
```


```
AIC(m0, m1)
```


```
BIC(m0, m1)
```


```
m_speed_ef <- lmer(formula = Speed_m ~ Task*Stim*EF_front*EF_par + (1 | ID), data=data_subset)
summary(m_speed_ef)
```


```
Linear mixed model fit by REML. t-tests use Satterthwaite's method ['lmerModLmerTest']
Formula: Speed_m ~ Task * Stim * EF_front * EF_par + (1 | ID)
   Data: data_subset

REML criterion at convergence: 917.5

Scaled residuals: 
    Min      1Q  Median      3Q     Max 
-3.1661 -0.4110 -0.0119  0.5734  2.4071 

Random effects:
 Groups   Name        Variance Std.Dev.
 ID       (Intercept)   8.245   2.871  
 Residual             275.329  16.593  
Number of obs: 152, groups:  ID, 20

Fixed effects:
                                          Estimate Std. Error         df t value Pr(>|t|)
(Intercept)                             -6.024e+00  1.781e+01  1.200e+02  -0.338    0.736
Task3back                                1.326e+00  2.485e+01  1.054e+02   0.053    0.958
StimTPre-GPar                            2.286e+01  2.485e+01  1.054e+02   0.920    0.360
StimTpar-GBst                            8.083e-02  2.496e+01  1.154e+02   0.003    0.997
StimTpre-GBst                            1.102e+01  2.496e+01  1.154e+02   0.441    0.660
EF_front                                -3.746e+01  2.117e+02  1.198e+02  -0.177    0.860
EF_par                                  -3.428e+02  3.305e+02  1.200e+02  -1.037    0.302
Task3back:StimTPre-GPar                 -3.500e+01  3.514e+01  1.054e+02  -0.996    0.321
Task3back:StimTpar-GBst                 -8.568e+00  3.507e+01  1.054e+02  -0.244    0.807
Task3back:StimTpre-GBst                 -5.398e+00  3.507e+01  1.054e+02  -0.154    0.878
Task3back:EF_front                      -1.480e+01  2.952e+02  1.054e+02  -0.050    0.960
StimTPre-GPar:EF_front                  -1.194e+02  2.952e+02  1.054e+02  -0.404    0.687
StimTpar-GBst:EF_front                   9.998e+01  2.296e+02  1.143e+02   0.436    0.664
StimTpre-GBst:EF_front                  -1.525e+01  2.296e+02  1.143e+02  -0.066    0.947
Task3back:EF_par                         5.102e+02  4.611e+02  1.054e+02   1.106    0.271
StimTPre-GPar:EF_par                    -4.127e+02  4.611e+02  1.054e+02  -0.895    0.373
StimTpar-GBst:EF_par                     2.203e+02  4.070e+02  1.172e+02   0.541    0.589
StimTpre-GBst:EF_par                     3.045e+02  4.070e+02  1.172e+02   0.748    0.456
EF_front:EF_par                          2.483e+03  2.622e+03  1.200e+02   0.947    0.345
Task3back:StimTPre-GPar:EF_front         1.390e+02  4.174e+02  1.054e+02   0.333    0.740
Task3back:StimTpar-GBst:EF_front         5.929e+00  3.230e+02  1.054e+02   0.018    0.985
Task3back:StimTpre-GBst:EF_front         1.458e+01  3.230e+02  1.054e+02   0.045    0.964
Task3back:StimTPre-GPar:EF_par           5.712e+02  6.521e+02  1.054e+02   0.876    0.383
Task3back:StimTpar-GBst:EF_par          -3.454e+02  5.708e+02  1.054e+02  -0.605    0.546
Task3back:StimTpre-GBst:EF_par          -5.417e+02  5.708e+02  1.054e+02  -0.949    0.345
Task3back:EF_front:EF_par               -3.235e+03  3.657e+03  1.054e+02  -0.885    0.378
StimTPre-GPar:EF_front:EF_par            3.586e+03  3.657e+03  1.054e+02   0.981    0.329
StimTpar-GBst:EF_front:EF_par           -2.482e+03  2.716e+03  1.187e+02  -0.914    0.363
StimTpre-GBst:EF_front:EF_par           -2.379e+03  2.716e+03  1.187e+02  -0.876    0.383
Task3back:StimTPre-GPar:EF_front:EF_par -4.420e+03  5.171e+03  1.054e+02  -0.855    0.395
Task3back:StimTpar-GBst:EF_front:EF_par  3.030e+03  3.804e+03  1.054e+02   0.797    0.427
Task3back:StimTpre-GBst:EF_front:EF_par  3.734e+03  3.804e+03  1.054e+02   0.981    0.329
```


```
Correlation matrix not shown by default, as p = 32 > 12.
Use print(x, correlation=TRUE)  or
    vcov(x)        if you need it
```


```
anova(m_speed_ef)
```


```
Type III Analysis of Variance Table with Satterthwaite's method
                           Sum Sq Mean Sq NumDF   DenDF F value  Pr(>F)  
Task                       213.44  213.44     1 105.420  0.7752 0.38061  
Stim                       167.22   55.74     3 113.886  0.2025 0.89451  
EF_front                    79.98   79.98     1  23.529  0.2905 0.59497  
EF_par                     231.26  231.26     1  37.697  0.8399 0.36524  
Task:Stim                  325.03  108.34     3 105.420  0.3935 0.75793  
Task:EF_front               13.28   13.28     1 105.420  0.0482 0.82659  
Stim:EF_front              516.19  172.06     3 111.951  0.6249 0.60034  
Task:EF_par               1257.35 1257.35     1 105.420  4.5667 0.03491 *
Stim:EF_par                111.49   37.16     3 111.911  0.1350 0.93900  
EF_front:EF_par            375.13  375.13     1  38.646  1.3625 0.25026  
Task:Stim:EF_front          49.66   16.55     3 105.420  0.0601 0.98058  
Task:Stim:EF_par          1156.80  385.60     3 105.420  1.4005 0.24681  
Task:EF_front:EF_par      1068.25 1068.25     1 105.420  3.8799 0.05149 .
Stim:EF_front:EF_par       468.82  156.27     3 106.133  0.5676 0.63757  
Task:Stim:EF_front:EF_par 1452.81  484.27     3 105.420  1.7589 0.15956  
---
Signif. codes:  0 ‘***’ 0.001 ‘**’ 0.01 ‘*’ 0.05 ‘.’ 0.1 ‘ ’ 1
```


```
data_subset <- subset(df_ef_epfl, Stim != 'Placebo' & Task == '2back')
data_subset <- droplevels(data_subset)
levels(data_subset$Stim)
```


```
[1] "GPre-TPar" "TPre-GPar" "Tpar-GBst" "Tpre-GBst"
```


```
levels(data_subset$Task)
```


```
[1] "2back"
```


```
m_speed_ef_2back <- lmer(formula = Speed_m ~ Stim*EF_front*EF_par + (1 | ID), data=data_subset)
summary(m_speed_ef_2back)
```


```
Linear mixed model fit by REML. t-tests use Satterthwaite's method ['lmerModLmerTest']
Formula: Speed_m ~ Stim * EF_front * EF_par + (1 | ID)
   Data: data_subset

REML criterion at convergence: 465.8

Scaled residuals: 
     Min       1Q   Median       3Q      Max 
-2.16709 -0.40544  0.01106  0.48382  2.23713 

Random effects:
 Groups   Name        Variance Std.Dev.
 ID       (Intercept)   8.693   2.948  
 Residual             309.627  17.596  
Number of obs: 76, groups:  ID, 20

Fixed effects:
                                Estimate Std. Error         df t value Pr(>|t|)
(Intercept)                      -6.4845    18.8806    59.9998  -0.343    0.732
StimTPre-GPar                    22.8560    26.3476    43.3933   0.867    0.390
StimTpar-GBst                    -0.6415    26.4734    54.5807  -0.024    0.981
StimTpre-GBst                    10.2952    26.4734    54.5807   0.389    0.699
EF_front                        -34.0366   224.3726    59.9543  -0.152    0.880
EF_par                         -332.8810   350.3709    59.9919  -0.950    0.346
StimTPre-GPar:EF_front         -119.3788   313.0100    43.3933  -0.381    0.705
StimTpar-GBst:EF_front           99.2896   243.4189    52.4517   0.408    0.685
StimTpre-GBst:EF_front          -15.9452   243.4189    52.4517  -0.066    0.948
StimTPre-GPar:EF_par           -412.6685   488.9882    43.3933  -0.844    0.403
StimTpar-GBst:EF_par            226.9977   431.6013    56.6906   0.526    0.601
StimTpre-GBst:EF_par            311.1848   431.6013    56.6906   0.721    0.474
EF_front:EF_par                2414.1665  2778.9665    59.9986   0.869    0.388
StimTPre-GPar:EF_front:EF_par  3585.8151  3877.7307    43.3933   0.925    0.360
StimTpar-GBst:EF_front:EF_par -2459.9347  2880.1232    58.2117  -0.854    0.397
StimTpre-GBst:EF_front:EF_par -2357.2288  2880.1232    58.2117  -0.818    0.416
```


```
Correlation matrix not shown by default, as p = 16 > 12.
Use print(x, correlation=TRUE)  or
    vcov(x)        if you need it
```


```
anova(m_speed_ef_2back)
```


```
Type III Analysis of Variance Table with Satterthwaite's method
                      Sum Sq Mean Sq NumDF  DenDF F value  Pr(>F)  
Stim                  324.77  108.26     3 49.913  0.3496 0.78956  
EF_front               71.77   71.77     1 20.639  0.2318 0.63525  
EF_par               1145.19 1145.19     1 30.343  3.6986 0.06389 .
Stim:EF_front         364.09  121.36     3 48.384  0.3920 0.75931  
Stim:EF_par           976.87  325.62     3 50.362  1.0517 0.37795  
EF_front:EF_par      1262.44 1262.44     1 32.200  4.0773 0.05185 .
Stim:EF_front:EF_par 1560.58  520.19     3 49.742  1.6801 0.18324  
---
Signif. codes:  0 ‘***’ 0.001 ‘**’ 0.01 ‘*’ 0.05 ‘.’ 0.1 ‘ ’ 1
```


```
data_subset <- subset(df_ef_epfl, Stim != 'Placebo' & Task == '3back')
data_subset <- droplevels(data_subset)
levels(data_subset$Stim)
```


```
[1] "GPre-TPar" "TPre-GPar" "Tpar-GBst" "Tpre-GBst"
```


```
levels(data_subset$Task)
```


```
[1] "3back"
```


```
m_speed_ef_3back <- lmer(formula = Speed_m ~ Stim*EF_front*EF_par + (1 | ID), data=data_subset)
summary(m_speed_ef_3back)
```


```
Linear mixed model fit by REML. t-tests use Satterthwaite's method ['lmerModLmerTest']
Formula: Speed_m ~ Stim * EF_front * EF_par + (1 | ID)
   Data: data_subset

REML criterion at convergence: 451.1

Scaled residuals: 
    Min      1Q  Median      3Q     Max 
-3.4364 -0.4313  0.0540  0.6146  2.0309 

Random effects:
 Groups   Name        Variance Std.Dev.
 ID       (Intercept)   2.148   1.466  
 Residual             246.888  15.713  
Number of obs: 76, groups:  ID, 20

Fixed effects:
                              Estimate Std. Error       df t value Pr(>|t|)
(Intercept)                     -4.669     16.708   60.000  -0.279    0.781
StimTPre-GPar                  -12.146     23.527   42.793  -0.516    0.608
StimTpar-GBst                   -8.145     23.532   54.744  -0.346    0.731
StimTpre-GBst                    5.962     23.532   54.744   0.253    0.801
EF_front                       -52.485    198.493   59.995  -0.264    0.792
EF_par                         169.654    310.075   59.999   0.547    0.586
StimTPre-GPar:EF_front          19.627    279.504   42.793   0.070    0.944
StimTpar-GBst:EF_front         104.803    216.607   52.000   0.484    0.631
StimTpre-GBst:EF_front          -1.785    216.607   52.000  -0.008    0.993
StimTPre-GPar:EF_par           158.572    436.645   42.793   0.363    0.718
StimTpar-GBst:EF_par          -131.415    383.233   56.948  -0.343    0.733
StimTpre-GBst:EF_par          -243.491    383.233   56.948  -0.635    0.528
EF_front:EF_par               -777.290   2458.976   60.000  -0.316    0.753
StimTPre-GPar:EF_front:EF_par -834.022   3462.644   42.793  -0.241    0.811
StimTpar-GBst:EF_front:EF_par  584.847   2555.105   58.187   0.229    0.820
StimTpre-GBst:EF_front:EF_par 1390.854   2555.105   58.187   0.544    0.588
```


```
Correlation matrix not shown by default, as p = 16 > 12.
Use print(x, correlation=TRUE)  or
    vcov(x)        if you need it
```


```
anova(m_speed_ef_3back)
```


```
Type III Analysis of Variance Table with Satterthwaite's method
                     Sum Sq Mean Sq NumDF  DenDF F value Pr(>F)
Stim                 177.31  59.105     3 49.712  0.2394 0.8685
EF_front              19.56  19.564     1 19.896  0.0792 0.7812
EF_par               176.50 176.499     1 28.255  0.7149 0.4049
Stim:EF_front        201.48  67.160     3 47.875  0.2720 0.8453
Stim:EF_par          301.03 100.343     3 50.063  0.4064 0.7490
EF_front:EF_par       72.06  72.061     1 30.789  0.2919 0.5929
Stim:EF_front:EF_par 325.24 108.412     3 49.230  0.4391 0.7260
```


#### Note

As there is no significant interaction among the induced fields and
the rate of improvement in speed, we will not do the explorations we did
for the accuracy.

### The relationship between the intensity of the applied electric field on the target and the normalized speed


```
data_subset <- subset(df_ef_epfl, Stim != 'Placebo')
data_subset <- droplevels(data_subset)
levels(data_subset$Stim)
```


```
[1] "GPre-TPar" "TPre-GPar" "Tpar-GBst" "Tpre-GBst"
```


```
m0 <- lm(formula = Speed_n_m ~ Task*Stim*EF_front*EF_par, data=data_subset)
summary(m0)
```


```
Call:
lm(formula = Speed_n_m ~ Task * Stim * EF_front * EF_par, data = data_subset)

Residuals:
      Min        1Q    Median        3Q       Max 
-0.029757 -0.007088 -0.000560  0.007446  0.034725 

Coefficients:
                                         Estimate Std. Error t value Pr(>|t|)
(Intercept)                             -0.008379   0.014040  -0.597    0.552
Task3back                                0.008830   0.019855   0.445    0.657
StimTPre-GPar                            0.016983   0.019855   0.855    0.394
StimTpar-GBst                           -0.003357   0.019816  -0.169    0.866
StimTpre-GBst                            0.013689   0.019816   0.691    0.491
EF_front                                 0.021967   0.166795   0.132    0.895
EF_par                                  -0.200259   0.260569  -0.769    0.444
Task3back:StimTPre-GPar                 -0.031316   0.028080  -1.115    0.267
Task3back:StimTpar-GBst                 -0.014605   0.028024  -0.521    0.603
Task3back:StimTpre-GBst                 -0.012299   0.028024  -0.439    0.662
Task3back:EF_front                      -0.085893   0.235884  -0.364    0.716
StimTPre-GPar:EF_front                  -0.083609   0.235884  -0.354    0.724
StimTpar-GBst:EF_front                   0.039457   0.182505   0.216    0.829
StimTpre-GBst:EF_front                  -0.078680   0.182505  -0.431    0.667
Task3back:EF_par                         0.273156   0.368500   0.741    0.460
StimTPre-GPar:EF_par                    -0.298005   0.368500  -0.809    0.420
StimTpar-GBst:EF_par                     0.231470   0.322537   0.718    0.474
StimTpre-GBst:EF_par                     0.188048   0.322537   0.583    0.561
EF_front:EF_par                          1.263406   2.066341   0.611    0.542
Task3back:StimTPre-GPar:EF_front         0.170979   0.333590   0.513    0.609
Task3back:StimTpar-GBst:EF_front         0.080425   0.258101   0.312    0.756
Task3back:StimTpre-GBst:EF_front         0.093661   0.258101   0.363    0.717
Task3back:StimTPre-GPar:EF_par           0.462748   0.521138   0.888    0.376
Task3back:StimTpar-GBst:EF_par          -0.163289   0.456137  -0.358    0.721
Task3back:StimTpre-GBst:EF_par          -0.342370   0.456137  -0.751    0.454
Task3back:EF_front:EF_par               -1.358113   2.922248  -0.465    0.643
StimTPre-GPar:EF_front:EF_par            2.361642   2.922248   0.808    0.421
StimTpar-GBst:EF_front:EF_par           -1.604609   2.149683  -0.746    0.457
StimTpre-GBst:EF_front:EF_par           -1.200566   2.149683  -0.558    0.578
Task3back:StimTPre-GPar:EF_front:EF_par -3.673843   4.132682  -0.889    0.376
Task3back:StimTpar-GBst:EF_front:EF_par  1.244351   3.040111   0.409    0.683
Task3back:StimTpre-GBst:EF_front:EF_par  1.891580   3.040111   0.622    0.535

Residual standard error: 0.01326 on 120 degrees of freedom
Multiple R-squared:  0.1952,    Adjusted R-squared:  -0.01267 
F-statistic: 0.9391 on 31 and 120 DF,  p-value: 0.5645
```


```
anova(m0)
```


```
Analysis of Variance Table

Response: Speed_n_m
                           Df    Sum Sq    Mean Sq F value  Pr(>F)  
Task                        1 0.0004263 0.00042626  2.4241 0.12211  
Stim                        3 0.0002750 0.00009168  0.5214 0.66838  
EF_front                    1 0.0001113 0.00011129  0.6329 0.42787  
EF_par                      1 0.0000010 0.00000104  0.0059 0.93895  
Task:Stim                   3 0.0007072 0.00023573  1.3406 0.26448  
Task:EF_front               1 0.0000882 0.00008822  0.5017 0.48013  
Stim:EF_front               3 0.0002065 0.00006884  0.3915 0.75935  
Task:EF_par                 1 0.0004503 0.00045035  2.5611 0.11215  
Stim:EF_par                 3 0.0001297 0.00004325  0.2460 0.86407  
EF_front:EF_par             1 0.0000023 0.00000232  0.0132 0.90873  
Task:Stim:EF_front          3 0.0016355 0.00054515  3.1003 0.02936 *
Task:Stim:EF_par            3 0.0000572 0.00001908  0.1085 0.95499  
Task:EF_front:EF_par        1 0.0000013 0.00000130  0.0074 0.93156  
Stim:EF_front:EF_par        3 0.0003944 0.00013148  0.7477 0.52573  
Task:Stim:EF_front:EF_par   3 0.0006325 0.00021084  1.1990 0.31320  
Residuals                 120 0.0211008 0.00017584                  
---
Signif. codes:  0 ‘***’ 0.001 ‘**’ 0.01 ‘*’ 0.05 ‘.’ 0.1 ‘ ’ 1
```


```
m1 <- lmer(formula = Speed_n_m ~ Task*Stim*EF_front*EF_par + (1 | ID), data=data_subset)
summary(m1)
```


```
Linear mixed model fit by REML. t-tests use Satterthwaite's method ['lmerModLmerTest']
Formula: Speed_n_m ~ Task * Stim * EF_front * EF_par + (1 | ID)
   Data: data_subset

REML criterion at convergence: -797.8

Scaled residuals: 
     Min       1Q   Median       3Q      Max 
-2.16849 -0.53002 -0.04214  0.56368  2.69049 

Random effects:
 Groups   Name        Variance  Std.Dev.
 ID       (Intercept) 5.476e-06 0.00234 
 Residual             1.703e-04 0.01305 
Number of obs: 152, groups:  ID, 20

Fixed effects:
                                          Estimate Std. Error         df t value Pr(>|t|)
(Intercept)                              -0.007994   0.014020 119.993033  -0.570    0.570
Task3back                                 0.008830   0.019541 105.172976   0.452    0.652
StimTPre-GPar                             0.016983   0.019541 105.172978   0.869    0.387
StimTpar-GBst                            -0.004061   0.019640 115.296102  -0.207    0.837
StimTpre-GBst                             0.012985   0.019640 115.296096   0.661    0.510
EF_front                                  0.019087   0.166687 119.710924   0.115    0.909
EF_par                                   -0.210176   0.260133 119.995432  -0.808    0.421
Task3back:StimTPre-GPar                  -0.031316   0.027634 105.172973  -1.133    0.260
Task3back:StimTpar-GBst                  -0.014605   0.027580 105.172969  -0.530    0.598
Task3back:StimTpre-GBst                  -0.012299   0.027580 105.172965  -0.446    0.657
Task3back:EF_front                       -0.085893   0.232142 105.172979  -0.370    0.712
StimTPre-GPar:EF_front                   -0.083609   0.232142 105.172977  -0.360    0.719
StimTpar-GBst:EF_front                    0.043572   0.180622 114.219741   0.241    0.810
StimTpre-GBst:EF_front                   -0.074565   0.180622 114.219740  -0.413    0.681
Task3back:EF_par                          0.273156   0.362655 105.172968   0.753    0.453
StimTPre-GPar:EF_par                     -0.298005   0.362655 105.172974  -0.822    0.413
StimTpar-GBst:EF_par                      0.244796   0.320233 117.042171   0.764    0.446
StimTpre-GBst:EF_par                      0.201374   0.320233 117.042167   0.629    0.531
EF_front:EF_par                           1.337869   2.063766 119.965453   0.648    0.518
Task3back:StimTPre-GPar:EF_front          0.170979   0.328298 105.172973   0.521    0.604
Task3back:StimTpar-GBst:EF_front          0.080425   0.254007 105.172976   0.317    0.752
Task3back:StimTpre-GBst:EF_front          0.093661   0.254007 105.172976   0.369    0.713
Task3back:StimTPre-GPar:EF_par            0.462748   0.512872 105.172968   0.902    0.369
Task3back:StimTpar-GBst:EF_par           -0.163289   0.448902 105.172966  -0.364    0.717
Task3back:StimTpre-GBst:EF_par           -0.342370   0.448902 105.172961  -0.763    0.447
Task3back:EF_front:EF_par                -1.358113   2.875897 105.172976  -0.472    0.638
StimTPre-GPar:EF_front:EF_par             2.361642   2.875897 105.172979   0.821    0.413
StimTpar-GBst:EF_front:EF_par            -1.689112   2.137763 118.717696  -0.790    0.431
StimTpre-GBst:EF_front:EF_par            -1.285069   2.137763 118.717695  -0.601    0.549
Task3back:StimTPre-GPar:EF_front:EF_par  -3.673843   4.067132 105.172973  -0.903    0.368
Task3back:StimTpar-GBst:EF_front:EF_par   1.244351   2.991890 105.172976   0.416    0.678
Task3back:StimTpre-GBst:EF_front:EF_par   1.891580   2.991890 105.172973   0.632    0.529
```


```
Correlation matrix not shown by default, as p = 32 > 12.
Use print(x, correlation=TRUE)  or
    vcov(x)        if you need it
```


```
anova(m1)
```


```
Type III Analysis of Variance Table with Satterthwaite's method
                              Sum Sq    Mean Sq NumDF   DenDF F value Pr(>F)
Task                      0.00005872 0.00005872     1 105.173  0.3448 0.5583
Stim                      0.00031387 0.00010462     3 113.780  0.6143 0.6071
EF_front                  0.00000616 0.00000616     1  23.187  0.0362 0.8508
EF_par                    0.00004094 0.00004094     1  37.585  0.2404 0.6268
Task:Stim                 0.00022208 0.00007403     3 105.173  0.4347 0.7286
Task:EF_front             0.00000000 0.00000000     1 105.173  0.0000 0.9967
Stim:EF_front             0.00041336 0.00013779     3 111.813  0.8090 0.4914
Task:EF_par               0.00046563 0.00046563     1 105.173  2.7341 0.1012
Stim:EF_par               0.00020418 0.00006806     3 111.871  0.3996 0.7535
EF_front:EF_par           0.00010134 0.00010134     1  38.324  0.5950 0.4452
Task:Stim:EF_front        0.00004761 0.00001587     3 105.173  0.0932 0.9637
Task:Stim:EF_par          0.00057395 0.00019132     3 105.173  1.1234 0.3431
Task:EF_front:EF_par      0.00033909 0.00033909     1 105.173  1.9910 0.1612
Stim:EF_front:EF_par      0.00040716 0.00013572     3 106.022  0.7969 0.4983
Task:Stim:EF_front:EF_par 0.00063251 0.00021084     3 105.173  1.2380 0.2997
```


```
AIC(m0, m1)
```


```
BIC(m0, m1)
```


```
m_speed_n_ef <- lm(formula = Speed_n_m ~ Task*Stim*EF_front*EF_par, data=data_subset)
summary(m_speed_n_ef)
```


```
Call:
lm(formula = Speed_n_m ~ Task * Stim * EF_front * EF_par, data = data_subset)

Residuals:
      Min        1Q    Median        3Q       Max 
-0.029757 -0.007088 -0.000560  0.007446  0.034725 

Coefficients:
                                         Estimate Std. Error t value Pr(>|t|)
(Intercept)                             -0.008379   0.014040  -0.597    0.552
Task3back                                0.008830   0.019855   0.445    0.657
StimTPre-GPar                            0.016983   0.019855   0.855    0.394
StimTpar-GBst                           -0.003357   0.019816  -0.169    0.866
StimTpre-GBst                            0.013689   0.019816   0.691    0.491
EF_front                                 0.021967   0.166795   0.132    0.895
EF_par                                  -0.200259   0.260569  -0.769    0.444
Task3back:StimTPre-GPar                 -0.031316   0.028080  -1.115    0.267
Task3back:StimTpar-GBst                 -0.014605   0.028024  -0.521    0.603
Task3back:StimTpre-GBst                 -0.012299   0.028024  -0.439    0.662
Task3back:EF_front                      -0.085893   0.235884  -0.364    0.716
StimTPre-GPar:EF_front                  -0.083609   0.235884  -0.354    0.724
StimTpar-GBst:EF_front                   0.039457   0.182505   0.216    0.829
StimTpre-GBst:EF_front                  -0.078680   0.182505  -0.431    0.667
Task3back:EF_par                         0.273156   0.368500   0.741    0.460
StimTPre-GPar:EF_par                    -0.298005   0.368500  -0.809    0.420
StimTpar-GBst:EF_par                     0.231470   0.322537   0.718    0.474
StimTpre-GBst:EF_par                     0.188048   0.322537   0.583    0.561
EF_front:EF_par                          1.263406   2.066341   0.611    0.542
Task3back:StimTPre-GPar:EF_front         0.170979   0.333590   0.513    0.609
Task3back:StimTpar-GBst:EF_front         0.080425   0.258101   0.312    0.756
Task3back:StimTpre-GBst:EF_front         0.093661   0.258101   0.363    0.717
Task3back:StimTPre-GPar:EF_par           0.462748   0.521138   0.888    0.376
Task3back:StimTpar-GBst:EF_par          -0.163289   0.456137  -0.358    0.721
Task3back:StimTpre-GBst:EF_par          -0.342370   0.456137  -0.751    0.454
Task3back:EF_front:EF_par               -1.358113   2.922248  -0.465    0.643
StimTPre-GPar:EF_front:EF_par            2.361642   2.922248   0.808    0.421
StimTpar-GBst:EF_front:EF_par           -1.604609   2.149683  -0.746    0.457
StimTpre-GBst:EF_front:EF_par           -1.200566   2.149683  -0.558    0.578
Task3back:StimTPre-GPar:EF_front:EF_par -3.673843   4.132682  -0.889    0.376
Task3back:StimTpar-GBst:EF_front:EF_par  1.244351   3.040111   0.409    0.683
Task3back:StimTpre-GBst:EF_front:EF_par  1.891580   3.040111   0.622    0.535

Residual standard error: 0.01326 on 120 degrees of freedom
Multiple R-squared:  0.1952,    Adjusted R-squared:  -0.01267 
F-statistic: 0.9391 on 31 and 120 DF,  p-value: 0.5645
```


```
anova(m_speed_n_ef)
```


```
Analysis of Variance Table

Response: Speed_n_m
                           Df    Sum Sq    Mean Sq F value  Pr(>F)  
Task                        1 0.0004263 0.00042626  2.4241 0.12211  
Stim                        3 0.0002750 0.00009168  0.5214 0.66838  
EF_front                    1 0.0001113 0.00011129  0.6329 0.42787  
EF_par                      1 0.0000010 0.00000104  0.0059 0.93895  
Task:Stim                   3 0.0007072 0.00023573  1.3406 0.26448  
Task:EF_front               1 0.0000882 0.00008822  0.5017 0.48013  
Stim:EF_front               3 0.0002065 0.00006884  0.3915 0.75935  
Task:EF_par                 1 0.0004503 0.00045035  2.5611 0.11215  
Stim:EF_par                 3 0.0001297 0.00004325  0.2460 0.86407  
EF_front:EF_par             1 0.0000023 0.00000232  0.0132 0.90873  
Task:Stim:EF_front          3 0.0016355 0.00054515  3.1003 0.02936 *
Task:Stim:EF_par            3 0.0000572 0.00001908  0.1085 0.95499  
Task:EF_front:EF_par        1 0.0000013 0.00000130  0.0074 0.93156  
Stim:EF_front:EF_par        3 0.0003944 0.00013148  0.7477 0.52573  
Task:Stim:EF_front:EF_par   3 0.0006325 0.00021084  1.1990 0.31320  
Residuals                 120 0.0211008 0.00017584                  
---
Signif. codes:  0 ‘***’ 0.001 ‘**’ 0.01 ‘*’ 0.05 ‘.’ 0.1 ‘ ’ 1
```


```
data_subset <- subset(df_ef_epfl, Stim != 'Placebo' & Task == '2back')
data_subset <- droplevels(data_subset)
levels(data_subset$Stim)
```


```
[1] "GPre-TPar" "TPre-GPar" "Tpar-GBst" "Tpre-GBst"
```


```
levels(data_subset$Task)
```


```
[1] "2back"
```


```
m_speed_n_ef_2back <- lm(formula = Speed_n_m ~ Stim*EF_front*EF_par, data=data_subset)
summary(m_speed_n_ef_2back)
```


```
Call:
lm(formula = Speed_n_m ~ Stim * EF_front * EF_par, data = data_subset)

Residuals:
      Min        1Q    Median        3Q       Max 
-0.029757 -0.008165 -0.000641  0.007987  0.034725 

Coefficients:
                               Estimate Std. Error t value Pr(>|t|)
(Intercept)                   -0.008379   0.015211  -0.551    0.584
StimTPre-GPar                  0.016983   0.021511   0.789    0.433
StimTpar-GBst                 -0.003357   0.021469  -0.156    0.876
StimTpre-GBst                  0.013689   0.021469   0.638    0.526
EF_front                       0.021967   0.180705   0.122    0.904
EF_par                        -0.200259   0.282300  -0.709    0.481
StimTPre-GPar:EF_front        -0.083609   0.255556  -0.327    0.745
StimTpar-GBst:EF_front         0.039457   0.197725   0.200    0.843
StimTpre-GBst:EF_front        -0.078680   0.197725  -0.398    0.692
StimTPre-GPar:EF_par          -0.298005   0.399233  -0.746    0.458
StimTpar-GBst:EF_par           0.231470   0.349437   0.662    0.510
StimTpre-GBst:EF_par           0.188048   0.349437   0.538    0.592
EF_front:EF_par                1.263406   2.238672   0.564    0.575
StimTPre-GPar:EF_front:EF_par  2.361642   3.165961   0.746    0.459
StimTpar-GBst:EF_front:EF_par -1.604609   2.328965  -0.689    0.493
StimTpre-GBst:EF_front:EF_par -1.200566   2.328965  -0.515    0.608

Residual standard error: 0.01437 on 60 degrees of freedom
Multiple R-squared:  0.1995,    Adjusted R-squared:  -0.000619 
F-statistic: 0.9969 on 15 and 60 DF,  p-value: 0.4703
```


```
anova(m_speed_n_ef_2back)
```


```
Analysis of Variance Table

Response: Speed_n_m
                     Df    Sum Sq    Mean Sq F value  Pr(>F)  
Stim                  3 0.0006441 0.00021471  1.0403 0.38133  
EF_front              1 0.0000007 0.00000067  0.0032 0.95479  
EF_par                1 0.0002041 0.00020409  0.9889 0.32402  
Stim:EF_front         3 0.0014590 0.00048634  2.3564 0.08075 .
Stim:EF_par           3 0.0001112 0.00003708  0.1797 0.90979  
EF_front:EF_par       1 0.0000036 0.00000355  0.0172 0.89609  
Stim:EF_front:EF_par  3 0.0006636 0.00022120  1.0717 0.36792  
Residuals            60 0.0123836 0.00020639                  
---
Signif. codes:  0 ‘***’ 0.001 ‘**’ 0.01 ‘*’ 0.05 ‘.’ 0.1 ‘ ’ 1
```


```
data_subset <- subset(df_ef_epfl, Stim != 'Placebo' & Task == '3back')
data_subset <- droplevels(data_subset)
levels(data_subset$Stim)
```


```
[1] "GPre-TPar" "TPre-GPar" "Tpar-GBst" "Tpre-GBst"
```


```
levels(data_subset$Task)
```


```
[1] "3back"
```


```
m_speed_n_ef_3back <- lm(formula = Speed_n_m ~ Stim*EF_front*EF_par, data=data_subset)
summary(m_speed_n_ef_3back)
```


```
Call:
lm(formula = Speed_n_m ~ Stim * EF_front * EF_par, data = data_subset)

Residuals:
      Min        1Q    Median        3Q       Max 
-0.026302 -0.006813 -0.000151  0.006285  0.033174 

Coefficients:
                                Estimate Std. Error t value Pr(>|t|)
(Intercept)                    0.0004507  0.0127620   0.035    0.972
StimTPre-GPar                 -0.0143335  0.0180482  -0.794    0.430
StimTpar-GBst                 -0.0179614  0.0180124  -0.997    0.323
StimTpre-GBst                  0.0013898  0.0180124   0.077    0.939
EF_front                      -0.0639257  0.1516133  -0.422    0.675
EF_par                         0.0728973  0.2368523   0.308    0.759
StimTPre-GPar:EF_front         0.0873703  0.2144136   0.407    0.685
StimTpar-GBst:EF_front         0.1198812  0.1658933   0.723    0.473
StimTpre-GBst:EF_front         0.0149810  0.1658933   0.090    0.928
StimTPre-GPar:EF_par           0.1647425  0.3349597   0.492    0.625
StimTpar-GBst:EF_par           0.0681809  0.2931804   0.233    0.817
StimTpre-GBst:EF_par          -0.1543223  0.2931804  -0.526    0.601
EF_front:EF_par               -0.0947072  1.8782648  -0.050    0.960
StimTPre-GPar:EF_front:EF_par -1.3122010  2.6562675  -0.494    0.623
StimTpar-GBst:EF_front:EF_par -0.3602583  1.9540207  -0.184    0.854
StimTpre-GBst:EF_front:EF_par  0.6910139  1.9540207   0.354    0.725

Residual standard error: 0.01205 on 60 degrees of freedom
Multiple R-squared:  0.1556,    Adjusted R-squared:  -0.0555 
F-statistic: 0.7371 on 15 and 60 DF,  p-value: 0.7377
```


```
anova(m_speed_n_ef_3back)
```


```
Analysis of Variance Table

Response: Speed_n_m
                     Df    Sum Sq    Mean Sq F value Pr(>F)
Stim                  3 0.0003381 1.1270e-04  0.7757 0.5122
EF_front              1 0.0001988 1.9884e-04  1.3686 0.2467
EF_par                1 0.0002473 2.4729e-04  1.7021 0.1970
Stim:EF_front         3 0.0003829 1.2765e-04  0.8786 0.4573
Stim:EF_par           3 0.0000757 2.5247e-05  0.1738 0.9137
EF_front:EF_par       1 0.0000001 7.3000e-08  0.0005 0.9822
Stim:EF_front:EF_par  3 0.0003633 1.2112e-04  0.8336 0.4807
Residuals            60 0.0087172 1.4529e-04
```

## Relationship between behavior and the phase-amplitude coupling index (PAC)

As part of our analysis, we extracted the PAC at both stimulation
sites for each subject, as was done by Reinhart and Nguyen (2019). In
this paper, the authors looked at the PAC at the “memory ROI” (left
centro-temporal cluster), and they found high-performing young adults to
have high PAC values in this region. In contrast, older adults had low
PAC values in that region, as well as low performance in the WM task.
However, when stimulation was applied, performance improved shortly
after starting the stimulation, and the PAC was also high in older
adults. So, the objective of this analysis is to see whether there are
meaningful differences in PAC correlating with performance.

The PAC features we are using are a comparison between the 2-back and
the 3-back tasks (subtraction), and they are the following: - Memory ROI
(Like in Reinhart and Nguyen) - Frontal stimulation ROI - Parietal
stimulation ROI

The comparison between the 2-back and the 3-back task was done
through a subtraction in the PAC values. The rationale is that this
difference isolates the neural activity related to retaining information
in working memory for longer, which is assumed to be more difficult in
the 3-back compared to the 2-back task and is supported by the
behavioral differences seen in both the speed and the accuracy when
comparing performance in both tasks. Therefore, the resulting delta in
PAC among both tasks would be related to an individual’s ability to
perform the (harder) 3-back task.


```
data_directory <- file.path(paste(curr_dir, '../../../Code/Notebooks/Results', sep='/'))

file_name <- file.path(paste(data_directory, "Behavioral_and_PAC_blocks_iCOG_Design_22_06_2023.txt", sep='/'))
df_pac <- read.delim(file_name, header = TRUE, na.strings = "NN")
head(df_pac, 5)
```


```
df_pac$ID <- as.factor(df_pac$ID)
df_pac$School <- as.factor(df_pac$School)
df_pac$Day <- as.factor(df_pac$Day)
df_pac$Task <- as.factor(df_pac$Task)
df_pac$Stim <- as.factor(df_pac$Stim)
df_pac$Acc_u <- as.numeric(df_pac$Acc_u)
df_pac$Acc_n_u <- as.numeric(df_pac$Acc_n_u)
df_pac$Speed_u <- as.numeric(df_pac$Speed_u)
df_pac$Speed_n_u <- as.numeric(df_pac$Speed_n_u)
df_pac$Acc_m <- as.numeric(df_pac$Acc_m)
df_pac$Acc_n_m <- as.numeric(df_pac$Acc_n_m)
df_pac$Speed_m <- as.numeric(df_pac$Speed_m)
df_pac$Speed_n_m <- as.numeric(df_pac$Speed_n_m)
df_pac$PAC_mem <- as.numeric(df_pac$PAC_mem)
df_pac$PAC_front <- as.numeric(df_pac$PAC_front)
df_pac$PAC_par <- as.numeric(df_pac$PAC_par)
```


```
df_pac_epfl <- subset(df_pac, School == 'EPFL')
df_pac_epfl <- droplevels(df_pac_epfl)
df_pac_epfl$Stim <- factor(df_pac_epfl$Stim, levels(df_pac_epfl$Stim)[c(1, 3, 2, 6, 4, 5)])
levels(df_pac_epfl$Stim)
```


```
[1] "Baseline"  "Placebo"   "GPre-TPar" "TPre-GPar" "Tpar-GBst" "Tpre-GBst"
```


### Relationship between PAC and performance

Based on Reinhart’s and Nguyen’s findings, people with higher PAC
values at the “Memory ROI” would be expected to have a better
performance in the task. Therefore, we will first test the relationship
between the PAC and the performance at baseline. As the PAC values we
are using consist on the difference of values between the 2back and
3back conditions, which is meant to reflect the coupling related to
higher working memory demands, we will use the behavioral scores of the
3back task. #### Accuracy and memory ROI


```
data_subset <- subset(df_pac_epfl, Stim == "Baseline" & Task == "3back")
data_subset <- droplevels(data_subset)
levels(data_subset$Stim)
```


```
[1] "Baseline"
```


```
levels(data_subset$Task)
```


```
[1] "3back"
```


#### Average accuracy


```
m_pac_mem_acc_u_BL <- lm(formula = Acc_u ~ PAC_mem, data=data_subset)
summary(m_pac_mem_acc_u_BL)
```


```
Call:
lm(formula = Acc_u ~ PAC_mem, data = data_subset)

Residuals:
     Min       1Q   Median       3Q      Max 
-0.39709 -0.02212  0.02547  0.10567  0.19511 

Coefficients:
             Estimate Std. Error t value Pr(>|t|)    
(Intercept)   0.72602    0.04451  16.312 5.91e-11 ***
PAC_mem     -26.66551   11.50732  -2.317    0.035 *  
---
Signif. codes:  0 ‘***’ 0.001 ‘**’ 0.01 ‘*’ 0.05 ‘.’ 0.1 ‘ ’ 1

Residual standard error: 0.1711 on 15 degrees of freedom
  (3 observations deleted due to missingness)
Multiple R-squared:  0.2636,    Adjusted R-squared:  0.2145 
F-statistic:  5.37 on 1 and 15 DF,  p-value: 0.03503
```


```
anova(m_pac_mem_acc_u_BL)
```


```
Analysis of Variance Table

Response: Acc_u
          Df  Sum Sq  Mean Sq F value  Pr(>F)  
PAC_mem    1 0.15714 0.157142  5.3697 0.03503 *
Residuals 15 0.43897 0.029264                  
---
Signif. codes:  0 ‘***’ 0.001 ‘**’ 0.01 ‘*’ 0.05 ‘.’ 0.1 ‘ ’ 1
```


```
eta_squared(m_pac_mem_acc_u_BL)
```


```
For one-way between subjects designs, partial eta squared is equivalent to eta squared. Returning eta
  squared.
```


```
# Effect Size for ANOVA

Parameter | Eta2 |       95% CI
-------------------------------
PAC_mem   | 0.26 | [0.01, 1.00]

- One-sided CIs: upper bound fixed at [1.00].
```

#### Note

There is a significant effect of the PAC values estimated at the
“memory” target on the mean accuracy participants had at baseline. Based
on the estimated slope, which is negative, it would appear the direction
of the effect is the opposite to the one reported by Reinhart and
Nguyen, who reported people with higher PAC values also possesed higher
accuracy at baseline. However, we are using the difference in PAC among
conditions so the interpretation is less direct.

#### Accuracy and PAC at 10/20 EEG electrode-based targets


```
m_pac_targets_acc_u_BL <- lm(formula = Acc_u ~ PAC_front*PAC_par, data=data_subset)
summary(m_pac_targets_acc_u_BL)
```


```
Call:
lm(formula = Acc_u ~ PAC_front * PAC_par, data = data_subset)

Residuals:
      Min        1Q    Median        3Q       Max 
-0.308631 -0.007385  0.042728  0.081817  0.163703 

Coefficients:
                    Estimate Std. Error t value Pr(>|t|)    
(Intercept)        7.646e-01  4.876e-02  15.680 7.98e-10 ***
PAC_front          2.241e+01  1.938e+01   1.156   0.2684    
PAC_par           -2.039e+01  1.205e+01  -1.692   0.1144    
PAC_front:PAC_par -6.827e+03  2.471e+03  -2.763   0.0161 *  
---
Signif. codes:  0 ‘***’ 0.001 ‘**’ 0.01 ‘*’ 0.05 ‘.’ 0.1 ‘ ’ 1

Residual standard error: 0.1524 on 13 degrees of freedom
  (3 observations deleted due to missingness)
Multiple R-squared:  0.4933,    Adjusted R-squared:  0.3764 
F-statistic: 4.219 on 3 and 13 DF,  p-value: 0.02738
```


```
anova(m_pac_targets_acc_u_BL)
```


```
Analysis of Variance Table

Response: Acc_u
                  Df   Sum Sq  Mean Sq F value  Pr(>F)  
PAC_front          1 0.100290 0.100290  4.3164 0.05812 .
PAC_par            1 0.016419 0.016419  0.7067 0.41575  
PAC_front:PAC_par  1 0.177352 0.177352  7.6331 0.01614 *
Residuals         13 0.302048 0.023234                  
---
Signif. codes:  0 ‘***’ 0.001 ‘**’ 0.01 ‘*’ 0.05 ‘.’ 0.1 ‘ ’ 1
```


```
eta_squared(m_pac_targets_acc_u_BL)
```


```
# Effect Size for ANOVA (Type I)

Parameter         | Eta2 (partial) |       95% CI
-------------------------------------------------
PAC_front         |           0.25 | [0.00, 1.00]
PAC_par           |           0.05 | [0.00, 1.00]
PAC_front:PAC_par |           0.37 | [0.05, 1.00]

- One-sided CIs: upper bound fixed at [1.00].
```

#### Explore the interaction between the bi-focal PAC and the accuracy


```
n_rows = 100
choose_model <- m_pac_targets_acc_u_BL
stim_list <- levels(data_subset$Stim)
i_start = min(min(data_subset$PAC_front, na.rm=TRUE), min(data_subset$PAC_par, na.rm=TRUE))
i_stop = max(max(data_subset$PAC_front, na.rm=TRUE), max(data_subset$PAC_par, na.rm=TRUE))

columns = c("Stim","PAC_front", "PAC_par") 
df_temp = data.frame(matrix(nrow = n_rows, ncol = length(columns))) 
colnames(df_temp) = columns

for (s in stim_list){
    
  df_temp$Stim <- rep(s, n_rows)
  df_temp$PAC_front <- rep(seq(i_start, i_stop, length.out = 10), 10)
  df_temp$PAC_par <- rep(seq(i_start, i_stop, length.out = 10), each=10)
}
```


```
preds <- predict(choose_model, df_temp)
min_z <- min(preds)
max_z = max(preds)
df_temp$Pred <- preds
```


```
# Viewing angles for the plots
angle_columns = c("Stim","Azim","Colatitude") 
df_angles = data.frame(matrix(nrow = 4, ncol = length(angle_columns))) 
colnames(df_angles) = angle_columns

df_angles$Stim <- stim_list
df_angles$Azim <- c(0, 0, 0, 0) # Theta, default 0
df_angles$Colatitude <- c(15, 15, 15, 15) # Phi, default 15
```


```
# Make plots
for (s in stim_list){
  
  disp_df <- subset(df_temp, Stim == s)
  persp(x=seq(i_start, i_stop, length.out = 10), y=seq(i_start, i_stop, length.out = 10), z=matrix(disp_df$Pred, nrow=10, ncol=10,byrow=TRUE), 
        theta = subset(df_angles, Stim == s)$Azim, phi = subset(df_angles, Stim == s)$Colatitude,
        xlab="PAC_front",ylab="PAC_par",zlab="Mean accuracy", main=paste(s,"3back"), zlim=c(2*min_z,2*max_z)) 
}
```


```
Warning: surface extends beyond the box
```


```
# Export the data for plotting
save_file_name <- file.path(paste(data_directory, "EPFL_PAC_BLAcc_interactions.csv", sep='/'))
write.csv(df_temp, save_file_name)
```


```
data_subset <- subset(df_pac_epfl, Stim != "Baseline" & Stim != "Placebo" & Task == "3back")
data_subset <- droplevels(data_subset)
levels(data_subset$Stim)
```


```
[1] "GPre-TPar" "TPre-GPar" "Tpar-GBst" "Tpre-GBst"
```


```
levels(data_subset$Task)
```


```
[1] "3back"
```


```
m_pac_mem_acc_u_stim <- lm(formula = Acc_u ~ Stim*PAC_mem, data=data_subset)
summary(m_pac_mem_acc_u_stim)
```


```
Call:
lm(formula = Acc_u ~ Stim * PAC_mem, data = data_subset)

Residuals:
     Min       1Q   Median       3Q      Max 
-0.46385 -0.01669  0.02620  0.09423  0.18620 

Coefficients:
                        Estimate Std. Error t value Pr(>|t|)    
(Intercept)             0.773523   0.039797  19.437   <2e-16 ***
StimTPre-GPar          -0.005512   0.056281  -0.098    0.922    
StimTpar-GBst           0.030987   0.058013   0.534    0.595    
StimTpre-GBst           0.010588   0.058013   0.183    0.856    
PAC_mem               -11.792551  10.289233  -1.146    0.257    
StimTPre-GPar:PAC_mem  -2.526975  14.551173  -0.174    0.863    
StimTpar-GBst:PAC_mem  -3.731913  14.556491  -0.256    0.799    
StimTpre-GBst:PAC_mem  -3.101918  14.556491  -0.213    0.832    
---
Signif. codes:  0 ‘***’ 0.001 ‘**’ 0.01 ‘*’ 0.05 ‘.’ 0.1 ‘ ’ 1

Residual standard error: 0.153 on 56 degrees of freedom
  (12 observations deleted due to missingness)
Multiple R-squared:  0.1255,    Adjusted R-squared:  0.01616 
F-statistic: 1.148 on 7 and 56 DF,  p-value: 0.3473
```


```
anova(m_pac_mem_acc_u_stim)
```


```
Analysis of Variance Table

Response: Acc_u
             Df  Sum Sq  Mean Sq F value   Pr(>F)   
Stim          3 0.00980 0.003268  0.1397 0.935832   
PAC_mem       1 0.17642 0.176417  7.5402 0.008098 **
Stim:PAC_mem  3 0.00177 0.000591  0.0253 0.994508   
Residuals    56 1.31023 0.023397                    
---
Signif. codes:  0 ‘***’ 0.001 ‘**’ 0.01 ‘*’ 0.05 ‘.’ 0.1 ‘ ’ 1
```


```
m_pac_targets_acc_u_stims <- lm(formula = Acc_u ~ Stim*PAC_front*PAC_par, data=data_subset)
summary(m_pac_targets_acc_u_stims)
```


```
Call:
lm(formula = Acc_u ~ Stim * PAC_front * PAC_par, data = data_subset)

Residuals:
      Min        1Q    Median        3Q       Max 
-0.233170 -0.042918  0.003883  0.046753  0.141995 

Coefficients:
                                  Estimate Std. Error t value Pr(>|t|)    
(Intercept)                      8.098e-01  2.599e-02  31.152  < 2e-16 ***
StimTPre-GPar                   -1.040e-02  3.676e-02  -0.283    0.778    
StimTpar-GBst                    5.027e-03  3.855e-02   0.130    0.897    
StimTpre-GBst                   -3.663e-03  3.855e-02  -0.095    0.925    
PAC_front                        7.574e+00  1.033e+01   0.733    0.467    
PAC_par                         -9.462e+00  6.421e+00  -1.473    0.147    
StimTPre-GPar:PAC_front         -1.393e+00  1.461e+01  -0.095    0.924    
StimTpar-GBst:PAC_front         -1.543e+01  1.497e+01  -1.031    0.308    
StimTpre-GBst:PAC_front         -8.768e+00  1.497e+01  -0.586    0.561    
StimTPre-GPar:PAC_par            5.647e+00  9.081e+00   0.622    0.537    
StimTpar-GBst:PAC_par           -4.863e-01  9.145e+00  -0.053    0.958    
StimTpre-GBst:PAC_par            4.713e-01  9.145e+00   0.052    0.959    
PAC_front:PAC_par               -6.106e+03  1.317e+03  -4.635 2.76e-05 ***
StimTPre-GPar:PAC_front:PAC_par -7.331e+02  1.863e+03  -0.394    0.696    
StimTpar-GBst:PAC_front:PAC_par  2.134e+03  1.900e+03   1.123    0.267    
StimTpre-GBst:PAC_front:PAC_par  1.136e+03  1.900e+03   0.598    0.553    
---
Signif. codes:  0 ‘***’ 0.001 ‘**’ 0.01 ‘*’ 0.05 ‘.’ 0.1 ‘ ’ 1

Residual standard error: 0.08126 on 48 degrees of freedom
  (12 observations deleted due to missingness)
Multiple R-squared:  0.7884,    Adjusted R-squared:  0.7223 
F-statistic: 11.93 on 15 and 48 DF,  p-value: 1.874e-11
```


```
anova(m_pac_targets_acc_u_stims)
```


```
Analysis of Variance Table

Response: Acc_u
                       Df  Sum Sq Mean Sq  F value    Pr(>F)    
Stim                    3 0.00980 0.00327   0.4949    0.6875    
PAC_front               1 0.69356 0.69356 105.0352 1.126e-13 ***
PAC_par                 1 0.00036 0.00036   0.0548    0.8159    
Stim:PAC_front          3 0.00435 0.00145   0.2198    0.8822    
Stim:PAC_par            3 0.01072 0.00357   0.5412    0.6564    
PAC_front:PAC_par       1 0.44504 0.44504  67.3977 1.064e-10 ***
Stim:PAC_front:PAC_par  3 0.01743 0.00581   0.8801    0.4581    
Residuals              48 0.31695 0.00660                       
---
Signif. codes:  0 ‘***’ 0.001 ‘**’ 0.01 ‘*’ 0.05 ‘.’ 0.1 ‘ ’ 1
```


```
eta_squared(m_pac_targets_acc_u_stims)
```


```
# Effect Size for ANOVA (Type I)

Parameter              | Eta2 (partial) |       95% CI
------------------------------------------------------
Stim                   |           0.03 | [0.00, 1.00]
PAC_front              |           0.69 | [0.56, 1.00]
PAC_par                |       1.14e-03 | [0.00, 1.00]
Stim:PAC_front         |           0.01 | [0.00, 1.00]
Stim:PAC_par           |           0.03 | [0.00, 1.00]
PAC_front:PAC_par      |           0.58 | [0.43, 1.00]
Stim:PAC_front:PAC_par |           0.05 | [0.00, 1.00]

- One-sided CIs: upper bound fixed at [1.00].
```

#### Note

There is no difference among the stimulation groups.The next plots
show the relationship between PAC and accuracy in each group to
visualize the model estimates.


```
n_rows = 100
choose_model <- m_pac_targets_acc_u_stims
stim_list <- levels(data_subset$Stim)
i_start = min(min(data_subset$PAC_front, na.rm=TRUE), min(data_subset$PAC_par, na.rm=TRUE))
i_stop = max(max(data_subset$PAC_front, na.rm=TRUE), max(data_subset$PAC_par, na.rm=TRUE))

columns = c("Stim","PAC_front", "PAC_par") 
df_test = data.frame(matrix(nrow = n_rows, ncol = length(columns))) 
colnames(df_test) = columns

columns = c("Stim","PAC_front", "PAC_par") 
df_temp = data.frame(matrix(nrow = n_rows, ncol = length(columns))) 
colnames(df_temp) = columns

for (s in stim_list){
  if (s == stim_list[1]){
  df_test$Stim <- rep(s, n_rows)
  df_test$PAC_front <- rep(seq(i_start, i_stop, length.out = 10), 10)
  df_test$PAC_par <- rep(seq(i_start, i_stop, length.out = 10), each=10)
  #df_test$Task <- sample(c('2back', '3back'), 100, replace=TRUE)
  }
  else{
  df_temp$Stim <- rep(s, n_rows)
  df_temp$PAC_front <- rep(seq(i_start, i_stop, length.out = 10), 10)
  df_temp$PAC_par <- rep(seq(i_start, i_stop, length.out = 10), each=10)
  #df_temp$Task <- sample(c('2back', '3back'), 100, replace=TRUE)
  df_test <- rbind(df_test, df_temp)
  }
  
}
```


```
preds <- predict(choose_model, df_test)
min_z <- min(preds)
max_z = max(preds)
df_test$Pred <- preds
```


```
# Viewing angles for the plots
angle_columns = c("Stim","Azim","Colatitude") 
df_angles = data.frame(matrix(nrow = 4, ncol = length(angle_columns))) 
colnames(df_angles) = angle_columns

df_angles$Stim <- stim_list
df_angles$Azim <- c(0, 0, 0, 0) # Theta, default 0
df_angles$Colatitude <- c(15, 15, 15, 15) # Phi, default 15
```


```
# Make plots
for (s in stim_list){
  disp_df <- subset(df_test, Stim == s)
  persp(x=seq(i_start, i_stop, length.out = 10), y=seq(i_start, i_stop, length.out = 10), z=matrix(disp_df$Pred, nrow=10, ncol=10,byrow=TRUE), 
        theta = subset(df_angles, Stim == s)$Azim, phi = subset(df_angles, Stim == s)$Colatitude,
        xlab="PAC_front",ylab="PAC_par",zlab="Mean accuracy", main=paste(s,"3back"), zlim=c(min_z,max_z)) 
}
```

#### Normalized accuracy


```
data_subset <- subset(df_pac_epfl, Stim == "Placebo" & Task == "3back")
data_subset <- droplevels(data_subset)
levels(data_subset$Stim)
```


```
[1] "Placebo"
```


```
levels(data_subset$Task)
```


```
[1] "3back"
```


```
m_pac_mem_acc_n_u_placebo <- lm(formula = Acc_n_u ~ PAC_mem, data=data_subset)
summary(m_pac_mem_acc_n_u_placebo)
```


```
Call:
lm(formula = Acc_n_u ~ PAC_mem, data = data_subset)

Residuals:
     Min       1Q   Median       3Q      Max 
-0.62562 -0.28787 -0.05724  0.11121  1.55234 

Coefficients:
            Estimate Std. Error t value Pr(>|t|)    
(Intercept)   1.1079     0.1299   8.528 3.88e-07 ***
PAC_mem      58.6830    33.5887   1.747    0.101    
---
Signif. codes:  0 ‘***’ 0.001 ‘**’ 0.01 ‘*’ 0.05 ‘.’ 0.1 ‘ ’ 1

Residual standard error: 0.4993 on 15 degrees of freedom
  (3 observations deleted due to missingness)
Multiple R-squared:  0.1691,    Adjusted R-squared:  0.1137 
F-statistic: 3.052 on 1 and 15 DF,  p-value: 0.1011
```


```
anova(m_pac_mem_acc_n_u_placebo)
```


```
Analysis of Variance Table

Response: Acc_n_u
          Df Sum Sq Mean Sq F value Pr(>F)
PAC_mem    1 0.7611 0.76106  3.0524 0.1011
Residuals 15 3.7400 0.24933
```


```
m_pac_targets_acc_n_u_placebo <- lm(formula = Acc_n_u ~ PAC_front*PAC_par, data=data_subset)
summary(m_pac_targets_acc_n_u_placebo)
```


```
Call:
lm(formula = Acc_n_u ~ PAC_front * PAC_par, data = data_subset)

Residuals:
     Min       1Q   Median       3Q      Max 
-0.47262 -0.34393 -0.03823  0.11577  1.73071 

Coefficients:
                    Estimate Std. Error t value Pr(>|t|)    
(Intercept)        1.328e+00  1.778e-01   7.468 4.71e-06 ***
PAC_front          7.531e+01  7.066e+01   1.066    0.306    
PAC_par           -2.591e+01  4.392e+01  -0.590    0.565    
PAC_front:PAC_par -1.106e+04  9.009e+03  -1.228    0.241    
---
Signif. codes:  0 ‘***’ 0.001 ‘**’ 0.01 ‘*’ 0.05 ‘.’ 0.1 ‘ ’ 1

Residual standard error: 0.5557 on 13 degrees of freedom
  (3 observations deleted due to missingness)
Multiple R-squared:  0.108, Adjusted R-squared:  -0.09789 
F-statistic: 0.5245 on 3 and 13 DF,  p-value: 0.673
```


```
anova(m_pac_targets_acc_n_u_placebo)
```


```
Analysis of Variance Table

Response: Acc_n_u
                  Df Sum Sq Mean Sq F value Pr(>F)
PAC_front          1 0.0075 0.00751  0.0243 0.8785
PAC_par            1 0.0126 0.01259  0.0408 0.8431
PAC_front:PAC_par  1 0.4659 0.46587  1.5084 0.2412
Residuals         13 4.0151 0.30885
```

#### Stimulations


```
data_subset <- subset(df_pac_epfl, Stim != "Placebo" & Task == "3back")
data_subset <- droplevels(data_subset)
levels(data_subset$Stim)
```


```
[1] "Baseline"  "GPre-TPar" "TPre-GPar" "Tpar-GBst" "Tpre-GBst"
```


```
levels(data_subset$Task)
```


```
[1] "3back"
```


```
m_pac_mem_acc_n_u_stim <- lm(formula = Acc_n_u ~ Stim*PAC_mem, data=data_subset)
summary(m_pac_mem_acc_n_u_stim)
```


```
Call:
lm(formula = Acc_n_u ~ Stim * PAC_mem, data = data_subset)

Residuals:
     Min       1Q   Median       3Q      Max 
-0.32124 -0.11382 -0.04545  0.09272  0.39980 

Coefficients:
                       Estimate Std. Error t value Pr(>|t|)    
(Intercept)             1.08961    0.04492  24.257   <2e-16 ***
StimTPre-GPar          -0.01626    0.06352  -0.256   0.7989    
StimTpar-GBst          -0.05726    0.06548  -0.875   0.3856    
StimTpre-GBst          -0.12109    0.06548  -1.849   0.0697 .  
PAC_mem               -20.96123   11.61341  -1.805   0.0765 .  
StimTPre-GPar:PAC_mem  34.30379   16.42384   2.089   0.0413 *  
StimTpar-GBst:PAC_mem  19.71590   16.42984   1.200   0.2352    
StimTpre-GBst:PAC_mem  26.22029   16.42984   1.596   0.1161    
---
Signif. codes:  0 ‘***’ 0.001 ‘**’ 0.01 ‘*’ 0.05 ‘.’ 0.1 ‘ ’ 1

Residual standard error: 0.1726 on 56 degrees of freedom
  (32 observations deleted due to missingness)
Multiple R-squared:  0.134, Adjusted R-squared:  0.02569 
F-statistic: 1.237 on 7 and 56 DF,  p-value: 0.2983
```


```
anova(m_pac_mem_acc_n_u_stim)
```


```
Analysis of Variance Table

Response: Acc_n_u
             Df  Sum Sq  Mean Sq F value Pr(>F)
Stim          3 0.11528 0.038426  1.2892 0.2871
PAC_mem       1 0.00072 0.000721  0.0242 0.8770
Stim:PAC_mem  3 0.14217 0.047390  1.5899 0.2020
Residuals    56 1.66917 0.029807
```


```
m_pac_targets_acc_n_u_stim <- lm(formula = Acc_n_u ~ Stim*PAC_front*PAC_par, data=data_subset)
summary(m_pac_targets_acc_n_u_stim)
```


```
Call:
lm(formula = Acc_n_u ~ Stim * PAC_front * PAC_par, data = data_subset)

Residuals:
     Min       1Q   Median       3Q      Max 
-0.27706 -0.08792 -0.01744  0.05822  0.42800 

Coefficients:
                                  Estimate Std. Error t value Pr(>|t|)    
(Intercept)                      1.106e+00  5.259e-02  21.021   <2e-16 ***
StimTPre-GPar                    1.131e-02  7.438e-02   0.152   0.8798    
StimTpar-GBst                   -4.546e-02  7.801e-02  -0.583   0.5628    
StimTpre-GBst                   -1.637e-01  7.801e-02  -2.098   0.0412 *  
PAC_front                        2.024e+01  2.090e+01   0.968   0.3379    
PAC_par                         -1.059e+01  1.299e+01  -0.815   0.4192    
StimTPre-GPar:PAC_front         -3.915e+00  2.956e+01  -0.132   0.8952    
StimTpar-GBst:PAC_front          2.076e+01  3.029e+01   0.685   0.4964    
StimTpre-GBst:PAC_front         -2.286e+01  3.029e+01  -0.755   0.4542    
StimTPre-GPar:PAC_par            1.617e+00  1.837e+01   0.088   0.9302    
StimTpar-GBst:PAC_par            1.825e+01  1.850e+01   0.986   0.3290    
StimTpre-GBst:PAC_par            2.678e+01  1.850e+01   1.447   0.1543    
PAC_front:PAC_par               -3.728e+03  2.665e+03  -1.399   0.1683    
StimTPre-GPar:PAC_front:PAC_par  2.585e+03  3.769e+03   0.686   0.4962    
StimTpar-GBst:PAC_front:PAC_par  2.390e+03  3.844e+03   0.622   0.5370    
StimTpre-GBst:PAC_front:PAC_par  6.269e+03  3.844e+03   1.631   0.1094    
---
Signif. codes:  0 ‘***’ 0.001 ‘**’ 0.01 ‘*’ 0.05 ‘.’ 0.1 ‘ ’ 1

Residual standard error: 0.1644 on 48 degrees of freedom
  (32 observations deleted due to missingness)
Multiple R-squared:  0.3268,    Adjusted R-squared:  0.1164 
F-statistic: 1.553 on 15 and 48 DF,  p-value: 0.1241
```


```
anova(m_pac_targets_acc_n_u_stim)
```


```
Analysis of Variance Table

Response: Acc_n_u
                       Df  Sum Sq  Mean Sq F value  Pr(>F)  
Stim                    3 0.11528 0.038426  1.4215 0.24801  
PAC_front               1 0.16862 0.168620  6.2380 0.01598 *
PAC_par                 1 0.00465 0.004648  0.1719 0.68024  
Stim:PAC_front          3 0.20281 0.067603  2.5009 0.07056 .
Stim:PAC_par            3 0.05156 0.017186  0.6358 0.59562  
PAC_front:PAC_par       1 0.01396 0.013955  0.5163 0.47592  
Stim:PAC_front:PAC_par  3 0.07298 0.024326  0.8999 0.44817  
Residuals              48 1.29749 0.027031                  
---
Signif. codes:  0 ‘***’ 0.001 ‘**’ 0.01 ‘*’ 0.05 ‘.’ 0.1 ‘ ’ 1
```

### PAC and speed

#### Baseline


```
data_subset <- subset(df_pac_epfl, Stim == "Baseline" & Task == "3back")
data_subset <- droplevels(data_subset)
levels(data_subset$Stim)
```


```
[1] "Baseline"
```


```
levels(data_subset$Task)
```


```
[1] "3back"
```

#### Memory ROI


```
m_pac_mem_speed_u_BL <- lm(formula = Speed_u ~ PAC_mem, data=data_subset)
summary(m_pac_mem_speed_u_BL)
```


```
Call:
lm(formula = Speed_u ~ PAC_mem, data = data_subset)

Residuals:
    Min      1Q  Median      3Q     Max 
-486.03 -149.98   11.96  187.58  389.77 

Coefficients:
            Estimate Std. Error t value Pr(>|t|)    
(Intercept)  1208.65      68.64  17.608 1.98e-11 ***
PAC_mem     11654.33   17747.38   0.657    0.521    
---
Signif. codes:  0 ‘***’ 0.001 ‘**’ 0.01 ‘*’ 0.05 ‘.’ 0.1 ‘ ’ 1

Residual standard error: 263.8 on 15 degrees of freedom
  (3 observations deleted due to missingness)
Multiple R-squared:  0.02795,   Adjusted R-squared:  -0.03686 
F-statistic: 0.4312 on 1 and 15 DF,  p-value: 0.5213
```


```
anova(m_pac_mem_speed_u_BL)
```


```
Analysis of Variance Table

Response: Speed_u
          Df  Sum Sq Mean Sq F value Pr(>F)
PAC_mem    1   30017   30017  0.4312 0.5213
Residuals 15 1044124   69608
```

#### Frontal and parietal targets


```
m_pac_targets_speed_u_BL <- lm(formula = Speed_u ~ PAC_front*PAC_par, data=data_subset)
summary(m_pac_targets_speed_u_BL)
```


```
Call:
lm(formula = Speed_u ~ PAC_front * PAC_par, data = data_subset)

Residuals:
    Min      1Q  Median      3Q     Max 
-411.63 -142.93   12.58  114.48  448.84 

Coefficients:
                    Estimate Std. Error t value Pr(>|t|)    
(Intercept)          1218.60      77.54  15.717 7.75e-10 ***
PAC_front          -31996.83   30817.15  -1.038    0.318    
PAC_par             -8622.16   19153.85  -0.450    0.660    
PAC_front:PAC_par -456860.74 3929200.59  -0.116    0.909    
---
Signif. codes:  0 ‘***’ 0.001 ‘**’ 0.01 ‘*’ 0.05 ‘.’ 0.1 ‘ ’ 1

Residual standard error: 242.4 on 13 degrees of freedom
  (3 observations deleted due to missingness)
Multiple R-squared:  0.289, Adjusted R-squared:  0.1249 
F-statistic: 1.761 on 3 and 13 DF,  p-value: 0.204
```


```
anova(m_pac_targets_speed_u_BL)
```


```
Analysis of Variance Table

Response: Speed_u
                  Df Sum Sq Mean Sq F value Pr(>F)  
PAC_front          1 298448  298448  5.0801 0.0421 *
PAC_par            1  11169   11169  0.1901 0.6700  
PAC_front:PAC_par  1    794     794  0.0135 0.9092  
Residuals         13 763729   58748                 
---
Signif. codes:  0 ‘***’ 0.001 ‘**’ 0.01 ‘*’ 0.05 ‘.’ 0.1 ‘ ’ 1
```


```
eta_squared(m_pac_targets_speed_u_BL)
```


```
# Effect Size for ANOVA (Type I)

Parameter         | Eta2 (partial) |       95% CI
-------------------------------------------------
PAC_front         |           0.28 | [0.01, 1.00]
PAC_par           |           0.01 | [0.00, 1.00]
PAC_front:PAC_par |       1.04e-03 | [0.00, 1.00]

- One-sided CIs: upper bound fixed at [1.00].
```

#### Stimulation groups


```
data_subset <- subset(df_pac_epfl, Stim != "Baseline" & Stim != "Placebo" & Task == "3back")
data_subset <- droplevels(data_subset)
levels(data_subset$Stim)
```


```
[1] "GPre-TPar" "TPre-GPar" "Tpar-GBst" "Tpre-GBst"
```


```
levels(data_subset$Task)
```


```
[1] "3back"
```

#### Memory ROI


```
m_pac_mem_speed_u_stim <- lm(formula = Speed_u ~ Stim*PAC_mem, data=data_subset)
summary(m_pac_mem_speed_u_stim)
```


```
Call:
lm(formula = Speed_u ~ Stim * PAC_mem, data = data_subset)

Residuals:
    Min      1Q  Median      3Q     Max 
-550.65 -198.98   27.48  163.66  528.77 

Coefficients:
                      Estimate Std. Error t value Pr(>|t|)    
(Intercept)            1112.43      71.93  15.466   <2e-16 ***
StimTPre-GPar            13.69     101.72   0.135    0.893    
StimTpar-GBst          -110.18     104.85  -1.051    0.298    
StimTpre-GBst          -108.03     104.85  -1.030    0.307    
PAC_mem                 152.99   18596.30   0.008    0.993    
StimTPre-GPar:PAC_mem  3830.46   26299.13   0.146    0.885    
StimTpar-GBst:PAC_mem  4629.74   26308.75   0.176    0.861    
StimTpre-GBst:PAC_mem  9734.28   26308.75   0.370    0.713    
---
Signif. codes:  0 ‘***’ 0.001 ‘**’ 0.01 ‘*’ 0.05 ‘.’ 0.1 ‘ ’ 1

Residual standard error: 276.5 on 56 degrees of freedom
  (12 observations deleted due to missingness)
Multiple R-squared:  0.04901,   Adjusted R-squared:  -0.06986 
F-statistic: 0.4123 on 7 and 56 DF,  p-value: 0.8907
```


```
anova(m_pac_mem_speed_u_stim)
```


```
Analysis of Variance Table

Response: Speed_u
             Df  Sum Sq Mean Sq F value Pr(>F)
Stim          3  190458   63486  0.8307 0.4826
PAC_mem       1   19511   19511  0.2553 0.6154
Stim:PAC_mem  3   10622    3541  0.0463 0.9866
Residuals    56 4279896   76427
```

#### Frontal and parietal targets


```
m_pac_targets_speed_u_stim <- lm(formula = Speed_u ~ Stim*PAC_front*PAC_par, data=data_subset)
summary(m_pac_targets_speed_u_stim)
```


```
Call:
lm(formula = Speed_u ~ Stim * PAC_front * PAC_par, data = data_subset)

Residuals:
    Min      1Q  Median      3Q     Max 
-425.46 -162.62  -26.13  138.42  551.06 

Coefficients:
                                  Estimate Std. Error t value Pr(>|t|)    
(Intercept)                      1.148e+03  7.918e+01  14.502   <2e-16 ***
StimTPre-GPar                    1.118e+01  1.120e+02   0.100    0.921    
StimTpar-GBst                   -1.560e+02  1.174e+02  -1.328    0.190    
StimTpre-GBst                   -1.700e+02  1.174e+02  -1.447    0.154    
PAC_front                       -9.359e+03  3.147e+04  -0.297    0.767    
PAC_par                         -2.607e+04  1.956e+04  -1.333    0.189    
StimTPre-GPar:PAC_front         -4.234e+03  4.451e+04  -0.095    0.925    
StimTpar-GBst:PAC_front         -2.477e+04  4.560e+04  -0.543    0.589    
StimTpre-GBst:PAC_front         -3.914e+04  4.560e+04  -0.858    0.395    
StimTPre-GPar:PAC_par            8.634e+03  2.766e+04   0.312    0.756    
StimTpar-GBst:PAC_par            1.690e+04  2.786e+04   0.607    0.547    
StimTpre-GBst:PAC_par            2.062e+04  2.786e+04   0.740    0.463    
PAC_front:PAC_par               -2.473e+06  4.013e+06  -0.616    0.541    
StimTPre-GPar:PAC_front:PAC_par -4.038e+05  5.675e+06  -0.071    0.944    
StimTpar-GBst:PAC_front:PAC_par  2.860e+06  5.787e+06   0.494    0.623    
StimTpre-GBst:PAC_front:PAC_par  4.375e+06  5.787e+06   0.756    0.453    
---
Signif. codes:  0 ‘***’ 0.001 ‘**’ 0.01 ‘*’ 0.05 ‘.’ 0.1 ‘ ’ 1

Residual standard error: 247.5 on 48 degrees of freedom
  (12 observations deleted due to missingness)
Multiple R-squared:  0.3465,    Adjusted R-squared:  0.1423 
F-statistic: 1.697 on 15 and 48 DF,  p-value: 0.08376
```


```
anova(m_pac_targets_speed_u_stim)
```


```
Analysis of Variance Table

Response: Speed_u
                       Df  Sum Sq Mean Sq F value    Pr(>F)    
Stim                    3  190458   63486  1.0362    0.3850    
PAC_front               1 1150437 1150437 18.7768 7.471e-05 ***
PAC_par                 1  127461  127461  2.0804    0.1557    
Stim:PAC_front          3    2975     992  0.0162    0.9972    
Stim:PAC_par            3   20207    6736  0.1099    0.9539    
PAC_front:PAC_par       1   10303   10303  0.1682    0.6836    
Stim:PAC_front:PAC_par  3   57737   19246  0.3141    0.8151    
Residuals              48 2940909   61269                      
---
Signif. codes:  0 ‘***’ 0.001 ‘**’ 0.01 ‘*’ 0.05 ‘.’ 0.1 ‘ ’ 1
```

#### Normalized speed


```
data_subset <- subset(df_pac_epfl, Stim == "Placebo" & Task == "3back")
data_subset <- droplevels(data_subset)
levels(data_subset$Stim)
```


```
[1] "Placebo"
```


```
levels(data_subset$Task)
```


```
[1] "3back"
```


```
m_pac_mem_speed_n_u_placebo <- lm(formula = Speed_n_u ~ PAC_mem, data=data_subset)
summary(m_pac_mem_speed_n_u_placebo)
```


```
Call:
lm(formula = Speed_n_u ~ PAC_mem, data = data_subset)

Residuals:
     Min       1Q   Median       3Q      Max 
-0.27527 -0.12421  0.03861  0.11091  0.23503 

Coefficients:
            Estimate Std. Error t value Pr(>|t|)    
(Intercept)  1.00238    0.04155  24.123 2.05e-13 ***
PAC_mem      2.82519   10.74305   0.263    0.796    
---
Signif. codes:  0 ‘***’ 0.001 ‘**’ 0.01 ‘*’ 0.05 ‘.’ 0.1 ‘ ’ 1

Residual standard error: 0.1597 on 15 degrees of freedom
  (3 observations deleted due to missingness)
Multiple R-squared:  0.004589,  Adjusted R-squared:  -0.06177 
F-statistic: 0.06916 on 1 and 15 DF,  p-value: 0.7961
```


```
anova(m_pac_mem_speed_n_u_placebo)
```


```
Analysis of Variance Table

Response: Speed_n_u
          Df  Sum Sq  Mean Sq F value Pr(>F)
PAC_mem    1 0.00176 0.001764  0.0692 0.7961
Residuals 15 0.38259 0.025506
```


```
m_pac_targets_speed_n_u_placebo <- lm(formula = Speed_n_u ~ PAC_front*PAC_par, data=data_subset)
summary(m_pac_targets_speed_n_u_placebo)
```


```
Call:
lm(formula = Speed_n_u ~ PAC_front * PAC_par, data = data_subset)

Residuals:
     Min       1Q   Median       3Q      Max 
-0.25770 -0.06823 -0.02190  0.11166  0.20804 

Coefficients:
                    Estimate Std. Error t value Pr(>|t|)    
(Intercept)          0.96433    0.04887  19.734  4.5e-11 ***
PAC_front          -14.94914   19.42259  -0.770    0.455    
PAC_par              7.59509   12.07176   0.629    0.540    
PAC_front:PAC_par 4100.90011 2476.38924   1.656    0.122    
---
Signif. codes:  0 ‘***’ 0.001 ‘**’ 0.01 ‘*’ 0.05 ‘.’ 0.1 ‘ ’ 1

Residual standard error: 0.1528 on 13 degrees of freedom
  (3 observations deleted due to missingness)
Multiple R-squared:  0.2107,    Adjusted R-squared:  0.02857 
F-statistic: 1.157 on 3 and 13 DF,  p-value: 0.3635
```


```
anova(m_pac_targets_speed_n_u_placebo)
```


```
Analysis of Variance Table

Response: Speed_n_u
                  Df   Sum Sq  Mean Sq F value Pr(>F)
PAC_front          1 0.016778 0.016778  0.7190 0.4118
PAC_par            1 0.000217 0.000217  0.0093 0.9246
PAC_front:PAC_par  1 0.063995 0.063995  2.7423 0.1216
Residuals         13 0.303367 0.023336
```

#### Stimulations


```
data_subset <- subset(df_pac_epfl, Stim != "Placebo" & Stim != 'Baseline' & Task == "3back")
data_subset <- droplevels(data_subset)
levels(data_subset$Stim)
```


```
[1] "GPre-TPar" "TPre-GPar" "Tpar-GBst" "Tpre-GBst"
```


```
levels(data_subset$Task)
```


```
[1] "3back"
```


```
m_pac_mem_speed_n_u_stim <- lm(formula = Speed_n_u ~ Stim*PAC_mem, data=data_subset)
summary(m_pac_mem_speed_n_u_stim)
```


```
Call:
lm(formula = Speed_n_u ~ Stim * PAC_mem, data = data_subset)

Residuals:
     Min       1Q   Median       3Q      Max 
-0.43694 -0.08505 -0.01432  0.08328  0.34258 

Coefficients:
                       Estimate Std. Error t value Pr(>|t|)    
(Intercept)             0.99969    0.04011  24.925   <2e-16 ***
StimTPre-GPar          -0.05744    0.05672  -1.013    0.316    
StimTpar-GBst          -0.07218    0.05847  -1.234    0.222    
StimTpre-GBst           0.02131    0.05847   0.364    0.717    
PAC_mem               -10.12997   10.36965  -0.977    0.333    
StimTPre-GPar:PAC_mem  24.23308   14.66491   1.652    0.104    
StimTpar-GBst:PAC_mem  21.08972   14.67027   1.438    0.156    
StimTpre-GBst:PAC_mem  16.02645   14.67027   1.092    0.279    
---
Signif. codes:  0 ‘***’ 0.001 ‘**’ 0.01 ‘*’ 0.05 ‘.’ 0.1 ‘ ’ 1

Residual standard error: 0.1542 on 56 degrees of freedom
  (12 observations deleted due to missingness)
Multiple R-squared:  0.1096,    Adjusted R-squared:  -0.001732 
F-statistic: 0.9844 on 7 and 56 DF,  p-value: 0.4519
```


```
anova(m_pac_mem_speed_n_u_stim)
```


```
Analysis of Variance Table

Response: Speed_n_u
             Df  Sum Sq  Mean Sq F value Pr(>F)
Stim          3 0.06295 0.020982  0.8829 0.4556
PAC_mem       1 0.02393 0.023932  1.0071 0.3199
Stim:PAC_mem  3 0.07688 0.025627  1.0784 0.3657
Residuals    56 1.33079 0.023764
```


```
m_pac_targets_speed_n_u_stim <- lm(formula = Speed_n_u ~ Stim*PAC_front*PAC_par, data=data_subset)
summary(m_pac_targets_speed_n_u_stim)
```


```
Call:
lm(formula = Speed_n_u ~ Stim * PAC_front * PAC_par, data = data_subset)

Residuals:
     Min       1Q   Median       3Q      Max 
-0.28160 -0.07775 -0.02108  0.05980  0.29578 

Coefficients:
                                  Estimate Std. Error t value Pr(>|t|)    
(Intercept)                      1.043e+00  4.565e-02  22.851  < 2e-16 ***
StimTPre-GPar                   -8.710e-02  6.456e-02  -1.349  0.18360    
StimTpar-GBst                   -1.088e-01  6.771e-02  -1.607  0.11469    
StimTpre-GBst                   -2.880e-02  6.771e-02  -0.425  0.67244    
PAC_front                        1.153e+01  1.814e+01   0.635  0.52813    
PAC_par                         -7.477e+00  1.128e+01  -0.663  0.51048    
StimTPre-GPar:PAC_front         -2.643e+01  2.566e+01  -1.030  0.30812    
StimTpar-GBst:PAC_front         -1.973e+01  2.629e+01  -0.750  0.45663    
StimTpre-GBst:PAC_front         -7.943e+00  2.629e+01  -0.302  0.76386    
StimTPre-GPar:PAC_par            1.128e+01  1.595e+01   0.707  0.48294    
StimTpar-GBst:PAC_par            4.101e+00  1.606e+01   0.255  0.79953    
StimTpre-GBst:PAC_par            5.136e+00  1.606e+01   0.320  0.75050    
PAC_front:PAC_par               -6.813e+03  2.313e+03  -2.945  0.00497 ** 
StimTPre-GPar:PAC_front:PAC_par  5.921e+03  3.272e+03   1.810  0.07658 .  
StimTpar-GBst:PAC_front:PAC_par  7.714e+03  3.336e+03   2.312  0.02510 *  
StimTpre-GBst:PAC_front:PAC_par  9.457e+03  3.336e+03   2.835  0.00669 ** 
---
Signif. codes:  0 ‘***’ 0.001 ‘**’ 0.01 ‘*’ 0.05 ‘.’ 0.1 ‘ ’ 1

Residual standard error: 0.1427 on 48 degrees of freedom
  (12 observations deleted due to missingness)
Multiple R-squared:  0.346, Adjusted R-squared:  0.1416 
F-statistic: 1.693 on 15 and 48 DF,  p-value: 0.08472
```


```
anova(m_pac_targets_speed_n_u_stim)
```


```
Analysis of Variance Table

Response: Speed_n_u
                       Df  Sum Sq  Mean Sq F value  Pr(>F)  
Stim                    3 0.06295 0.020982  1.0304 0.38757  
PAC_front               1 0.05419 0.054186  2.6609 0.10938  
PAC_par                 1 0.00027 0.000266  0.0131 0.90948  
Stim:PAC_front          3 0.17416 0.058054  2.8509 0.04704 *
Stim:PAC_par            3 0.01839 0.006132  0.3011 0.82443  
PAC_front:PAC_par       1 0.01931 0.019315  0.9485 0.33499  
Stim:PAC_front:PAC_par  3 0.18783 0.062609  3.0746 0.03636 *
Residuals              48 0.97745 0.020364                  
---
Signif. codes:  0 ‘***’ 0.001 ‘**’ 0.01 ‘*’ 0.05 ‘.’ 0.1 ‘ ’ 1
```


```
n_rows = 100
choose_model <- m_pac_targets_speed_n_u_stim
stim_list <- levels(data_subset$Stim)
i_start = min(min(data_subset$PAC_front, na.rm=TRUE), min(data_subset$PAC_par, na.rm=TRUE))
i_stop = max(max(data_subset$PAC_front, na.rm=TRUE), max(data_subset$PAC_par, na.rm=TRUE))

columns = c("Stim","PAC_front", "PAC_par") 
df_test = data.frame(matrix(nrow = n_rows, ncol = length(columns))) 
colnames(df_test) = columns

columns = c("Stim","PAC_front", "PAC_par") 
df_temp = data.frame(matrix(nrow = n_rows, ncol = length(columns))) 
colnames(df_temp) = columns

for (s in stim_list){
  if (s == stim_list[1]){
  df_test$Stim <- rep(s, n_rows)
  df_test$PAC_front <- rep(seq(i_start, i_stop, length.out = 10), 10)
  df_test$PAC_par <- rep(seq(i_start, i_stop, length.out = 10), each=10)
  #df_test$Task <- sample(c('2back', '3back'), 100, replace=TRUE)
  }
  else{
  df_temp$Stim <- rep(s, n_rows)
  df_temp$PAC_front <- rep(seq(i_start, i_stop, length.out = 10), 10)
  df_temp$PAC_par <- rep(seq(i_start, i_stop, length.out = 10), each=10)
  #df_temp$Task <- sample(c('2back', '3back'), 100, replace=TRUE)
  df_test <- rbind(df_test, df_temp)
  }
  
}
```


```
preds <- predict(choose_model, df_test)
min_z <- min(preds)
max_z = max(preds)
df_test$Pred <- preds
```


```
# Viewing angles for the plots
angle_columns = c("Stim","Azim","Colatitude") 
df_angles = data.frame(matrix(nrow = 4, ncol = length(angle_columns))) 
colnames(df_angles) = angle_columns

df_angles$Stim <- stim_list
df_angles$Azim <- c(0, 0, 0, 0) # Theta, default 0
df_angles$Colatitude <- c(15, 15, 15, 15) # Phi, default 15
```


```
# Make plots
for (s in stim_list){
  disp_df <- subset(df_test, Stim == s)
  persp(x=seq(i_start, i_stop, length.out = 10), y=seq(i_start, i_stop, length.out = 10), z=matrix(disp_df$Pred, nrow=10, ncol=10,byrow=TRUE), 
        theta = subset(df_angles, Stim == s)$Azim, phi = subset(df_angles, Stim == s)$Colatitude,
        xlab="PAC_front",ylab="PAC_par",zlab="Mean normalized speed", main=paste(s,"3back"), zlim=c(min_z,max_z)) 
}
```

LS0tDQp0aXRsZTogImlDT0cgTVMgc3RhdHMgZm9yIHRoZSBFUEZMIGRhdGEiDQpvdXRwdXQ6IGh0bWxfbm90ZWJvb2sNCi0tLQ0KcmVudjo6YWN0aXZhdGUoIkM6L1VzZXJzL3BhYmxvLm1hY2VpcmEvc3dpdGNoZHJpdmUvU0RfQ29zbW9zL015X2RvY3VtZW50cy9Qcm9qZWN0cy9pQ09HL0FuYWx5c2lzL1N0YXRzL0NsZWFuLyIpDQpgYGB7cn0NCmxpYnJhcnkoTWF0cml4KQ0KbGlicmFyeShsbWU0KQ0KbGlicmFyeShjYXJEYXRhKQ0KbGlicmFyeShjYXIpDQpsaWJyYXJ5KGxtZXJUZXN0KQ0KbGlicmFyeShlbW1lYW5zKQ0KbGlicmFyeShlZmZlY3RzaXplKQ0KbGlicmFyeShnZ3Bsb3QyKQ0KbGlicmFyeShSbWlzYykNCiNsaWJyYXJ5KGZsZXhwbG90KQ0KYGBgDQpgYGB7cn0NCmN1cnJfZGlyIDwtIGdldHdkKCkNCmRhdGFfZGlyZWN0b3J5IDwtIGZpbGUucGF0aChwYXN0ZShjdXJyX2RpciwgJy4uLy4uLy4uL0NvZGUvTm90ZWJvb2tzL1Jlc3VsdHMnLCBzZXA9Jy8nKSkNCg0KZmlsZV9uYW1lIDwtIGZpbGUucGF0aChwYXN0ZShkYXRhX2RpcmVjdG9yeSwgImlDT0dfYWxsX2RhdGEudHh0Iiwgc2VwPScvJykpDQpkZiA8LSByZWFkLmRlbGltKGZpbGVfbmFtZSwgaGVhZGVyID0gVFJVRSwgbmEuc3RyaW5ncyA9ICJOTiIpDQpoZWFkKGRmLCA1KQ0KYGBgDQoNCmBgYHtyfQ0KZGYkSUQgPC0gYXMuZmFjdG9yKGRmJElEKQ0KZGYkU2Nob29sIDwtIGFzLmZhY3RvcihkZiRTY2hvb2wpDQpkZiREYXkgPC0gYXMuZmFjdG9yKGRmJERheSkNCmRmJFRhc2sgPC0gYXMuZmFjdG9yKGRmJFRhc2spDQpkZiRCbG9jayA8LSBhcy5udW1lcmljKGRmJEJsb2NrKQ0KZGYkU3RpbSA8LSBhcy5mYWN0b3IoZGYkU3RpbSkNCmRmJEFjYyA8LSBhcy5udW1lcmljKGRmJEFjYykNCmRmJFNwZWVkX2NvcnIgPC0gYXMubnVtZXJpYyhkZiRTcGVlZF9jb3JyKQ0KZGYkRF9wcmltZSA8LSBhcy5udW1lcmljKGRmJERfcHJpbWUpDQpkZiRBY2Nfbm9ybSA8LSBhcy5udW1lcmljKGRmJEFjY19ub3JtKQ0KZGYkU3BlZWRfbm9ybSA8LSBhcy5udW1lcmljKGRmJFNwZWVkX25vcm0pDQpkZiREX3ByaW1lX25vcm0gPC0gYXMubnVtZXJpYyhkZiREX3ByaW1lX25vcm0pDQoNCmBgYA0KIyBFUEZMIERhdGEgUmVzdWx0cw0KYGBge3J9DQpkZl9lcGZsIDwtIHN1YnNldChkZiwgU2Nob29sID09ICdFUEZMJykNCmRmX2VwZmwgPC0gZHJvcGxldmVscyhkZl9lcGZsKQ0KZGZfZXBmbCRTdGltIDwtIGZhY3RvcihkZl9lcGZsJFN0aW0sIGxldmVscyhkZl9lcGZsJFN0aW0pW2MoMiwgMSwgNSwgMywgNCldKQ0KbGV2ZWxzKGRmX2VwZmwkU3RpbSkNCmBgYA0KDQojIyBBY2N1cmFjeSBTdGF0cw0KIyMjIFNpbXBsZSBtb2RlbCwgYXZlcmFnZXMNCkhlcmUsIHdlIHByZXNlbnQgZGlmZmVyZW50IG1vZGVscyB0byBjaGFyYWN0ZXJpemUgZWFjaCByZWxldmFudCBwYXJhbWV0ZXIgcmVsYXRlZCB0byB0aGUgcGFydGljaXBhbnRzJyBwZXJmb3JtYW5jZS4gSW4gZWFjaCBjYXNlLCB0aGUgbW9kZWxzIHN0YXJ0IG9mZiBmcm9tIGEgc2ltcGxlDQpmb3JtIChpLmUuLCBncm91cCBhdmVyYWdlcyBwZXIgc2Vzc2lvbiksIGFuZCB0aGV5IGdyb3cgaW4gY29tcGxleGl0eSBhcyB3ZSBhZGQgb3RoZXIgZml4ZWQgZmFjdG9ycyAoZS5nLiwgdHJhaW5pbmcgYmxvY2tzKSBhbmQgcmFuZG9tIGVmZmVjdHMgdG8gYWNjb3VudCBmb3IgdGhlIA0KdmFyaWFiaWxpdHkgYWNyb3NzIHN1YmplY3RzLiBFYWNoIG1vZGVsIGlzIGNvbXBhcmVkIHN0YXRpc3RpY2FsbHkgdG8gaXRzIChzaW1wbGVyKSBwcmVkZWNlc3NvciB0byBhc3Nlc3Mgd2hldGhlciB0aGUgZXZlbnR1YWwgaW1wcm92ZW1lbnQgaW4gZGF0YSBleHBsYWluYWJpbGl0eSwgDQphcyBxdWFudGlmaWVkIGJ5IHR3byBjb252ZW50aW9uYWwgY3JpdGVyaWEgKGkuZS4sIEFrYWlrZSdzIGFuZCBCYXllcycgaW5mb3JtYXRpb24gY3JpdGVyaWEpLCBqdXN0aWZpZXMgdGhlIHVzZSBvZiBhIG1vcmUgY29tcGxleCBtb2RlbC4gDQpgYGB7cn0NCm0wIDwtIGxtKGZvcm11bGEgPSBBY2MgfiBTdGltKlRhc2ssIGRhdGE9ZGZfZXBmbCkNCnN1bW1hcnkobTApDQphbm92YShtMCkNCmBgYA0KDQpMaW5lYXIgbW9kZWwsIG5vIHJhbmRvbSBlZmZlY3RzDQpgYGB7cn0NCm0xIDwtIGxtKGZvcm11bGEgPSBBY2MgfiBTdGltKlRhc2sqQmxvY2ssIGRhdGE9ZGZfZXBmbCkNCnN1bW1hcnkobTEpDQphbm92YShtMSkNCmBgYA0KQ29tcGFyZSBtb2RlbHMNCmBgYHtyfQ0KYW5vdmEobTAsIG0xKQ0KYGBgDQoNCkxpbmVhciBtb2RlbCwgcmFuZG9tIGludGVyY2VwdCBhbmQgcmFuZG9tIHNsb3BlDQpSYW5kb20gSW50ZXJjZXB0IHBlciBzdWJqZWN0DQpgYGB7cn0NCm0yIDwtIGxtZXIoZm9ybXVsYSA9IEFjYyB+IFN0aW0qVGFzaypCbG9jayArICgxIHwgSUQpLCBkYXRhPWRmX2VwZmwpDQpzdW1tYXJ5KG0yKQ0KYW5vdmEobTIpDQpgYGANCiMjIyMgQ29tcGFyZSBtb2RlbHMNCldoZW4gY29tcGFyaW5nIGEgbGluZWFyIG1vZGVsIChsbSkgdG8gYSBsaW5lYXIgbWl4ZWQtZWZmZWN0IG1vZGVsIChsbWUpLCB0aGUgcmVndWxhciBBTk9WQSBkb2VzIG5vdCB3b3JrLCBzbyBJIHdpbGwgb25seSBsb29rIGF0IHRoZSBpbmZvcm1hdGlvbiBjcml0ZXJpYQ0KYGBge3J9DQpBSUMobTEsIG0yKQ0KQklDKG0xLCBtMikNCmBgYA0KIyMjIE5vdGU6DQpUaGUgbW9kZWwgaW5jbHVkaW5nIHJhbmRvbSBpbnRlcmNlcHRzIGlzIG11Y2ggYmV0dGVyIHRoYW4gdGhlIHJlZ3VsYXIgbGluZWFyIG1vZGVsLiBXZSB3aWxsIHNlZSB3aGV0aGVyIGluY2x1ZGluZyBhIHJhbmRvbSBzbG9wZSBpbXByb3ZlcyB0aGUgbW9kZWwuDQoNCiMjIyMgUmFuZG9tIGludGVyY2VwdCBhbmQgcmFuZG9tIHNsb3BlIHBlciBzdWJqZWN0DQpgYGB7cn0NCm0zIDwtIGxtZXIoZm9ybXVsYSA9IEFjYyB+IFN0aW0qVGFzaypCbG9jayArICgxICsgQmxvY2t8SUQpLCBkYXRhPWRmX2VwZmwpDQpzdW1tYXJ5KG0zKQ0KYW5vdmEobTMpDQpgYGANCiMjIyMgQ29tcGFyZSBtb2RlbHMsIGV2ZW4gdGhvdWdoIHRoaXMgbGFzdCBvbmUgZGlkIG5vdCBjb252ZXJnZQ0KYGBge3J9DQphbm92YShtMiwgbTMpDQpgYGANClRoZSBhZGRpdGlvbiBvZiBhIHJhbmRvbSBzbG9wZSByZXN1bHRzIGluIGEgbW9kZWwgdGhhdCBkb2VzIG5vdCBjb252ZXJnZS4gSW4gYWRkaXRpb24sIHRoaXMgbW9kZWwgZG9lcyBub3Qgc2lnbmlmaWNhbnRseSBpbXByb3ZlIHRoZSBwcmV2aW91cyBvbmUsIHNvIHdlIHdpbGwgbm90IGluY2x1ZGUgYSByYW5kb20gc2xvcGUuDQoNCk1vZGVsIHdpdGggcmFuZG9tIGludGVyY2VwdCBwZXIgcGFydGljaXBhbnQgYW5kIHBlciBkYXksIHRyeWluZyB0byBhY2NvdW50IGZvciBkaWZmZXJlbmNlcyBiZXR3ZWVuIHNlc3Npb24gZGF5cw0KYGBge3J9DQptNCA8LSBsbWVyKGZvcm11bGEgPSBBY2MgfiBTdGltKlRhc2sqQmxvY2sgKyAoMSArICgxfElEKSArICgxfERheSkpLCBkYXRhPWRmX2VwZmwpDQpzdW1tYXJ5KG00KQ0KYW5vdmEobTQpDQpgYGANCiMjIyMgQ29tcGFyZSBtb2RlbHMNCmBgYHtyfQ0KYW5vdmEobTIsIG00KQ0KYGBgDQoNCiMjIyBNb2RlbCBjaG9pY2UNCkFkZGluZyBhIHJhbmRvbSBpbnRlcmNlcHQgZm9yIHRoZSB0cmFpbmluZyBkYXkgc2lnbmlmaWNhbnRseSBpbXByb3ZlcyB0aGUgbW9kZWwsIHNvIHdlIHdpbGwgdXNlIHRoaXMgbW9kZWwuIA0KDQojIyMgDQoNCmBgYHtyfQ0KbV9hY2MgPC0gbG1lcihmb3JtdWxhID0gQWNjIH4gU3RpbSpUYXNrKkJsb2NrICsgKDEgKyAoMXxJRCkgKyAoMXxEYXkpKSwgZGF0YT1kZl9lcGZsKQ0Kc3VtbWFyeShtX2FjYykNCmFub3ZhKG1fYWNjKQ0KZXRhX3NxdWFyZWQobV9hY2MpDQpgYGANClRoZXJlIGlzIGEgc2lnbmlmaWNhbnQgaW50ZXJhY3Rpb24gb2YgdGFzayBhbmQgYmxvY2tzIHN1Z2dlc3RpbmcgZGlmZmVyZW50IHJhdGVzIGluIHBlcmZvcm1hbmNlIGNoYW5nZSBvbiBlYWNoIHRhc2suIEJhc2VkIG9uIHRoaXMsIGFuZCBvbiB0aGUgZXhwZWN0YXRpb24gZm9yIA0KZGlmZmVyZW50IHBlcmZvcm1hbmNlIGluIHRoZSAyLWJhY2sgY29tcGFyZWQgdG8gdGhlIDMtYmFjayB0YXNrIHJlc3VsdGluZyBmcm9tIHRoZSBkaWZmZXJlbnQgZGlmZmljdWx0eSBsZXZlbHMgaW4gYm90aCB0YXNrcywgd2Ugd2lsbCBjb21wYXJlIHRoZSBhY2N1cmFjeQ0KYW1vbmcgc3RpbXVsYXRpb24gY29uZGl0aW9ucyBmb3IgZWFjaCB0YXNrIHNlcGFyYXRlbHkuDQoNCiMjIyBBY2N1cmFjeSBpbiB0aGUgMi1iYWNrIHRhc2sNCmBgYHtyfQ0KZGF0YV9zdWJzZXQgPC0gc3Vic2V0KGRmX2VwZmwsIFRhc2sgPT0gJzJiYWNrJykNCmRhdGFfc3Vic2V0IDwtIGRyb3BsZXZlbHMoZGF0YV9zdWJzZXQpDQpsZXZlbHMoZGF0YV9zdWJzZXQkVGFzaykNCmBgYA0KYGBge3J9DQptX2FjY18yYmFjayA8LSBsbWVyKGZvcm11bGEgPSBBY2MgfiBTdGltKkJsb2NrICsgKDEgKyAoMXxJRCkgKyAoMXxEYXkpKSwgZGF0YT1kYXRhX3N1YnNldCkNCnN1bW1hcnkobV9hY2NfMmJhY2spDQphbm92YShtX2FjY18yYmFjaykNCmV0YV9zcXVhcmVkKG1fYWNjXzJiYWNrKQ0KYGBgDQpUaGVyZSBpcyBubyBzaWduaWZpY2FudCBlZmZlY3Qgb2Ygc3RpbXVsYXRpb24sIG5vciBhIHNpZ25pZmljYW50IGRpZmZlcmVuY2UgaW4gdGhlIHJhdGUgb2YgcGVyZm9ybWFuY2UgY2hhbmdlIGFjcm9zcyBjb25kaXRpb25zLiBUaGVyZSB3YXMgb25seSBhIHNpZ25pZmljYW50IGVmZmVjdCBvZg0KYmxvY2suDQoNCiMjIyBBY2N1cmFjeSBpbiB0aGUgMy1iYWNrIHRhc2sNCmBgYHtyfQ0KZGF0YV9zdWJzZXQgPC0gc3Vic2V0KGRmX2VwZmwsIFRhc2sgPT0gJzNiYWNrJykNCmRhdGFfc3Vic2V0IDwtIGRyb3BsZXZlbHMoZGF0YV9zdWJzZXQpDQpsZXZlbHMoZGF0YV9zdWJzZXQkVGFzaykNCmBgYA0KYGBge3J9DQptX2FjY18zYmFjayA8LSBsbWVyKGZvcm11bGEgPSBBY2MgfiBTdGltKkJsb2NrICsgKDEgKyAoMXxJRCkgKyAoMXxEYXkpKSwgZGF0YT1kYXRhX3N1YnNldCkNCnN1bW1hcnkobV9hY2NfM2JhY2spDQphbm92YShtX2FjY18zYmFjaykNCmV0YV9zcXVhcmVkKG1fYWNjXzNiYWNrKQ0KYGBgDQojIyMgTm90ZToNClRoZXJlIGlzIG5vIGV2aWRlbmNlIGZvciBzaWduaWZpY2FudCBkaWZmZXJlbmNlcyBpbiB0aGUgMy1iYWNrIHRhc2suDQoNCiMjIE5vcm1hbGl6ZWQgYWNjdXJhY3kNCkh1bWFuIGJlaGF2aW9yIHRlbmRzIHRvIGJlIGhpZ2hseSB2YXJpYWJsZSBhY3Jvc3Mgc3ViamVjdHMsIHdoaWNoIHNvbWV0aW1lcyBtYWtlcyBhIGRpcmVjdCBjb21wYXJpc29uIG9mIHBlcmZvcm1hbmNlIGNoYWxsZW5naW5nLiBGb3IgdGhpcyByZWFzb24sIHdlIHdpbGwgbm93DQphc3Nlc3MgdGhlIGNoYW5nZSBpbiBhY2N1cmFjeSBleHBlcmllbmNlZCBieSBlYWNoIHBhcnRpY2lwYW50LCB3aGljaCB3ZSBpbnRlbmQgdG8gdXNlIGFzIGEgY29ycmVjdGlvbiBmb3IgbmF0aXZlIGRpZmZlcmVuY2VzIHByZXNlbnQgYXQgdGhlIHN0YXJ0IG9mIGVhY2ggc2Vzc2lvbi4NClRoaXMgY29ycmVjdGlvbiB3YXMgZG9uZSBieSBkaXZpZGluZyB0aGUgYWNjdXJhY3kgc2NvcmUgb2YgZWFjaCBibG9jayBieSB0aGF0IG9mIHRoZSBmaXJzdCB0cmFpbmluZyBibG9jayBvZiBlYWNoIHNlc3Npb24uIFBsZWFzZSBub3RlIHRoaXMgY29ycmVjdGlvbiB3YXMgZG9uZSANCndpdGhpbiBlYWNoIHNlc3Npb24gKGkuZS4sIHVzaW5nIHRoZSBmaXJzdCB0cmFpbmluZyBibG9jayBvZiBlYWNoIHNlc3Npb24pLCBhcyBvcHBvc2VkIHRvIHVzaW5nIHRoZSBmaXJzdCBibG9jayBvZiB0cmFpbmluZyBldmVyIHBlcmZvcm1lZCBieSBlYWNoIHBhcnRpY2lwYW50LiANCg0KIyMjIEdyb3VwIGF2ZXJhZ2VzDQpgYGB7cn0NCm0wIDwtIGxtKGZvcm11bGEgPSBBY2Nfbm9ybSB+IFN0aW0qVGFzaywgZGF0YT1kZl9lcGZsKQ0Kc3VtbWFyeShtMCkNCmFub3ZhKG0wKQ0KYGBgDQojIyMgTm90ZTogDQpXZSB3aWxsIG5vdyB0ZXN0IGEgbW9kZWwgaW5jbHVkaW5nIGEgdGltZSBjb21wb25lbnQgKGkuZS4sIHRoZSB0cmFpbmluZyBibG9ja3MpLg0KYGBge3J9DQptMSA8LSBsbShmb3JtdWxhID0gQWNjX25vcm0gfiBTdGltKlRhc2sqQmxvY2ssIGRhdGE9ZGZfZXBmbCkNCnN1bW1hcnkobTEpDQphbm92YShtMSkNCmBgYA0KIyMjIyBDb21wYXJlIHRoZSBtb2RlbHMNCmBgYHtyfQ0KYW5vdmEobTAsIG0xKQ0KYGBgDQpUaGUgYWRkaXRpb24gb2YgdGhlIGJsb2NrcyBzaWduaWZpY2FudGx5IGltcHJvdmVzIHRoZSBtb2RlbC4gV2Ugd2lsbCB0ZXN0IHRoZSBpbmNsdXNpb24gb2YgcmFuZG9tIGVmZmVjdHMgbmV4dC4NCg0KSW5jbHVkZSByYW5kb20gZWZmZWN0cw0KUmFuZG9tIGludGVyY2VwdCBwZXIgc3ViamVjdA0KYGBge3J9DQptMiA8LSBsbWVyKGZvcm11bGEgPSBBY2Nfbm9ybSB+IFN0aW0qVGFzaypCbG9jayArICgxIHwgSUQpLCBkYXRhPWRmX2VwZmwpDQpzdW1tYXJ5KG0yKQ0KYW5vdmEobTIpDQpgYGANCg0KYGBge3J9DQpBSUMobTEsIG0yKQ0KQklDKG0xLCBtMikNCmBgYA0KVGhlIGFkZGl0aW9uIG9mIGEgcmFuZG9tIGludGVyY2VwdCBmb3Igc3ViamVjdHMgd29yc2VucyB0aGUgbW9kZWwuIFdlIHdpbGwgdGVzdCB0aGUgYWRkaXRpb24gb2YgYSByYW5kb20gc2xvcGUgbmV4dC4NCg0KIyMjIFRlc3QgYSBtb2RlbCB3aXRoIGEgcmFuZG9tIHNsb3BlDQpgYGB7cn0NCm0zIDwtIGxtZXIoZm9ybXVsYSA9IEFjY19ub3JtIH4gU3RpbSpUYXNrKkJsb2NrICsgKDEgKyBCbG9ja3xJRCksIGRhdGE9ZGZfZXBmbCkNCnN1bW1hcnkobTMpDQphbm92YShtMykNCmBgYA0KYGBge3J9DQpBSUMobTEsIG0zKQ0KQklDKG0xLCBtMykNCmBgYA0KVGhlIGFkZGl0aW9uIG9mIGEgcmFuZG9tIHNsb3BlIGFsc28gd29yc2VucyBtMS4gTmV4dCwgd2Ugd2lsbCB0ZXN0IHRoZSBhIG1vZGVsIGluY2x1ZGluZyBhIHJhbmRvbSBpbnRlcmNlcHQgZm9yIGRheXMgYXMgd2VsbC4NCkFkZCByYW5kb20gaW50ZXJjZXB0IHBlciBkYXkNCmBgYHtyfQ0KbTQgPC0gbG1lcihmb3JtdWxhID0gQWNjX25vcm0gfiBTdGltKlRhc2sqQmxvY2sgKyAoMSArICgxfElEKSArICgxfERheSkpLCBkYXRhPWRmX2VwZmwpDQpzdW1tYXJ5KG00KQ0KYW5vdmEobTQpDQpgYGANCmBgYHtyfQ0KQUlDKG0xLCBtNCkNCkJJQyhtMSwgbTQpDQpgYGANCg0KDQojIyMgTW9kZWwgY2hvaWNlDQpJbiB0aGUgY2FzZSBvZiB0aGUgbm9ybWFsaXplZCBhY2N1cmFjeSwgYWRkaW5nIHJhbmRvbSBlZmZlY3RzIGRvZXMgbm90IGltcHJvdmUgdGhlIG1vZGVsLiANCg0KYGBge3J9DQptX2FjY19ub3JtIDwtIGxtKGZvcm11bGEgPSBBY2Nfbm9ybSB+IFN0aW0qVGFzaypCbG9jaywgZGF0YT1kZl9lcGZsKQ0Kc3VtbWFyeShtX2FjY19ub3JtKQ0KYW5vdmEobV9hY2Nfbm9ybSkNCmV0YV9zcXVhcmVkKG1fYWNjX25vcm0pDQpgYGANCiMjIyBOb3RlOiANClRoZXJlIGlzIGFuIGVmZmVjdCBvZiBzdGltdWxhdGlvbiwgd2hpY2ggc2VlbXMgdG8gZGlmZmVyIGFtb25nIHRoZSB0YXNrcywgd2hpY2ggYXJlIGFsc28gZGlmZmVyZW50LiBGb2xsb3dpbmcgdGhlIHNhbWUgcmF0aW9uYWxlIGFzIHdlIA0KYXBwbGllZCB0byB0aGUgdW5jb3JyZWN0ZWQgYWNjdXJhY3ksIHdlIHdpbGwgdGVzdCB0aGUgdGFza3Mgc2VwYXJhdGVseSBuZXh0Lg0KDQojIyMgTm9ybWFsaXplZCBhY2N1cmFjeSBpbiB0aGUgMi1iYWNrIHRhc2sNCmBgYHtyfQ0KZGF0YV9zdWJzZXQgPC0gc3Vic2V0KGRmX2VwZmwsIFRhc2sgPT0gJzJiYWNrJykNCmRhdGFfc3Vic2V0IDwtIGRyb3BsZXZlbHMoZGF0YV9zdWJzZXQpDQpsZXZlbHMoZGF0YV9zdWJzZXQkVGFzaykNCmBgYA0KYGBge3J9DQptX2FjY19ub3JtXzJiYWNrIDwtIGxtKGZvcm11bGEgPSBBY2Nfbm9ybSB+IFN0aW0qQmxvY2ssIGRhdGE9ZGF0YV9zdWJzZXQpDQpzdW1tYXJ5KG1fYWNjX25vcm1fMmJhY2spDQphbm92YShtX2FjY19ub3JtXzJiYWNrKQ0KZXRhX3NxdWFyZWQobV9hY2Nfbm9ybV8yYmFjaykNCmBgYA0KIyMjIE5vdGU6DQpUaGVyZSBpcyBhbiBlZmZlY3Qgb2Ygc3RpbXVsYXRpb24gaW4gdGhlIDItYmFjayB0YXNrLiBXZSB3aWxsIG5vdyBkbyBwb3N0LWhvYyBjb21wYXJpc29ucyB0byBzZWUgd2hlcmUgdGhlc2UgZGlmZmVyZW5jZXMgY29tZSBmcm9tLg0KYGBge3J9DQpteV9tb2RlbCA8LSBtX2FjY19ub3JtXzJiYWNrDQpsZXZlbF8xIDwtICJTdGltIg0KDQpteV9tb2RlbC5jb21wYXJlIDwtIGVtbWVhbnMobXlfbW9kZWwsIGxldmVsXzEsIGJ5PSJCbG9jayIsIGF0ID0gbGlzdChCbG9jayA9IGMoNywgMTMpKSkNCm15X21vZGVsLmNvbXBhcmUucGFpcnMgPC0gcGFpcnMobXlfbW9kZWwuY29tcGFyZSwgYWRqdXN0PSd0dWtleScpDQp0ZXN0KG15X21vZGVsLmNvbXBhcmUucGFpcnMsIHNpZGU9J3R3by1zaWRlZCcpDQpjb25maW50KG15X21vZGVsLmNvbXBhcmUsIGNhbGMgPSBjKG4gPSB+LndndC4pKQ0KZWZmX3NpemUobXlfbW9kZWwuY29tcGFyZSwgc2lnbWEgPSBzaWdtYShteV9tb2RlbCksIGVkZiA9IDIzKQ0KYGBgDQojIyMgTm90ZToNClRoZSBwb3N0LWhvYyB0ZXN0cyByZXZlYWxlZCB0aGF0IHRoZSB0aGV0YS1wYXJpZXRhbC1nYW1tYS1idXJzdCB2YXJpYW50IHdhcyBzaWduaWZpY2FudGx5IHdvcnNlIHRoYW4gdGhlIHBsYWNlYm8sIGFzIHdlbGwgYXMgc2lnbmlmaWNhbnRseSB3b3JzZSB0aGFuIHRoZQ0KZ2FtbWEtcHJlZnJvbnRhbC10aGV0YS1wYXJpZXRhbCBhbmQgdGhlIHRoZXRhLXByZWZyb250YWwtZ2FtbWEtcGFyaWV0YWwuIE5vdGUgdGhhdCBpbiB0aGlzIG1vZGVsIHdlIHJ1biBwb3N0LWhvYyB0ZXN0cyBhdCBibG9jayA3IChpLmUuLCBtaWQtcG9pbnQgb2YgdGhlIA0KZml0dGVkIGxpbmUsIHdoaWNoIGlzIHRoZSBkZWZhdWx0IGluIGVtbWVhbnMpIGFuZCBhdCBibG9jayAxMywgd2hpY2ggd2FzIHRoZSBsYXN0IGJsb2NrIG9mIGVhY2ggc2Vzc2lvbi4gVGhlIHJlYXNvbiB3YXMgdG8gY29tcGFyZSB0aGUgcGVyZm9ybWFuY2Ugb2YgdGhlIA0KcGFydGljaXBhbnRzIGF0IHRoZSBlbmQgb2YgZWFjaCBzZXNzaW9uLCBhcyB0aGVyZSBzZWVtcyB0byBiZSBhIHN0ZWVwIGltcHJvdmVtZW50IGluIHBlcmZvcm1hbmNlIG92ZXIgdGhlIGNvdXJzZSBvZiB0cmFpbmluZywgb24gYXZlcmFnZS4gV2hlbiBsb29raW5nIGF0DQp0aGUgZW5kcG9pbnQgb2YgdGhlIGxpbmVzIGZpdHRlZCB0byB0aGUgbm9ybWFsaXplZCBhY2N1cmFjeSBhbmQsIGVzcGVjaWFsbHksIHdoZW4gY29tcGFyaW5nIHRoZSBnYW1tYS1wcmVmcm9udGFsLXRoZXRhLXBhcmlldGFsIGNvbmRpdGlvbiB0byBwbGFjZWJvIGluIHRoZQ0KMi1iYWNrIHRhc2ssIGl0IHNlZW1zIGxpa2UgcGFydGljaXBhbnRzIHJlYWNoIGEgaGlnaGVyIHBvaW50IGluIHBlcmZvcm1hbmNlIHRvd2FyZHMgdGhlIGVuZCBvZiB0cmFpbmluZy4gVGhpcyBkaWZmZXJlbmNlIHdhcyBub3Qgc3RhdGlzdGljYWxseSBzaWduaWZpY2FudCwNCmhvd2V2ZXIuIFdoZW4gcGxvdHRpbmcgaW5kaXZpZHVhbCBwb2ludHMgKHBsZWFzZSBzZWUgdGhlIHBsb3QgZm9yIHRoZSBub3JtYWxpemVkIGFjY3VyYWN5IGluIHRoZSBTdXBwbGVtZW50YXJ5IE1hdGVyaWFscyksIHdlIGNhbiBzZWUgYSBzaW5nbGUgbG9uZSBkYXRhIA0KcG9pbnQgYWJvdmUgNCwgd2hpY2ggbWF5IGJlIGRyaXZpbmcgdGhlIHN0ZWVwZXIgc2xvcGUgb2JzZXJ2ZWQuDQoNCiMjIyBOb3JtYWxpemVkIGFjY3VyYWN5IGluIHRoZSAzLWJhY2sgdGFzaw0KYGBge3J9DQpkYXRhX3N1YnNldCA8LSBzdWJzZXQoZGZfZXBmbCwgVGFzayA9PSAnM2JhY2snKQ0KZGF0YV9zdWJzZXQgPC0gZHJvcGxldmVscyhkYXRhX3N1YnNldCkNCmxldmVscyhkYXRhX3N1YnNldCRUYXNrKQ0KYGBgDQpgYGB7cn0NCm1fYWNjX25vcm1fM2JhY2sgPC0gbG0oZm9ybXVsYSA9IEFjY19ub3JtIH4gU3RpbSpCbG9jaywgZGF0YT1kYXRhX3N1YnNldCkNCnN1bW1hcnkobV9hY2Nfbm9ybV8zYmFjaykNCmFub3ZhKG1fYWNjX25vcm1fM2JhY2spDQpldGFfc3F1YXJlZChtX2FjY19ub3JtXzNiYWNrKQ0KYGBgDQpgYGB7cn0NCm15X21vZGVsIDwtIG1fYWNjX25vcm1fM2JhY2sNCmxldmVsXzEgPC0gIlN0aW0iDQoNCm15X21vZGVsLmNvbXBhcmUgPC0gZW1tZWFucyhteV9tb2RlbCwgbGV2ZWxfMSwgYnk9IkJsb2NrIiwgYXQgPSBsaXN0KEJsb2NrID0gYyg3LCAxMykpKQ0KbXlfbW9kZWwuY29tcGFyZS5wYWlycyA8LSBwYWlycyhteV9tb2RlbC5jb21wYXJlLCBhZGp1c3Q9J3R1a2V5JykNCnRlc3QobXlfbW9kZWwuY29tcGFyZS5wYWlycywgc2lkZT0ndHdvLXNpZGVkJykNCmNvbmZpbnQobXlfbW9kZWwuY29tcGFyZSwgY2FsYyA9IGMobiA9IH4ud2d0LikpDQplZmZfc2l6ZShteV9tb2RlbC5jb21wYXJlLCBzaWdtYSA9IHNpZ21hKG15X21vZGVsKSwgZWRmID0gMjMpDQpgYGANCg0KDQojIyBTcGVlZA0KTm93LCB3ZSBwcmVzZW50IHRoZSBzYW1lIHR5cGUgb2Ygc3RhdGlzdGljYWwgYW5hbHlzaXMgb24gdGhlIHNwZWVkIG9mIHRoZSBwYXJ0aWNpcGFudHMuIFBsZWFzZSBub3RlIGZvciB0aGlzIGNvbXBhcmlzb24sIHdlIGNvbnNpZGVyIHRoZSBzcGVlZCBvZiBjb3JyZWN0DQp0cmlhbHMgb25seS4NCg0KYGBge3J9DQptMCA8LSBsbShmb3JtdWxhID0gU3BlZWRfY29yciB+IFN0aW0qVGFzaywgZGF0YT1kZl9lcGZsKQ0Kc3VtbWFyeShtMCkNCmFub3ZhKG0wKQ0KYGBgDQojIyMgTGluZWFyIG1vZGVsLCBubyByYW5kb20gZWZmZWN0cw0KYGBge3J9DQptMSA8LSBsbShmb3JtdWxhID0gU3BlZWRfY29yciB+IFN0aW0qVGFzaypCbG9jaywgZGF0YT1kZl9lcGZsKQ0Kc3VtbWFyeShtMSkNCmFub3ZhKG0xKQ0KYGBgDQpDb21wYXJlIG1vZGVscw0KYGBge3J9DQphbm92YShtMCwgbTEpDQpgYGANCg0KTGluZWFyIG1vZGVsLCByYW5kb20gaW50ZXJjZXB0IGFuZCByYW5kb20gc2xvcGUNClJhbmRvbSBJbnRlcmNlcHQgcGVyIHN1YmplY3QNCmBgYHtyfQ0KbTIgPC0gbG1lcihmb3JtdWxhID0gU3BlZWRfY29yciB+IFN0aW0qVGFzaypCbG9jayArICgxIHwgSUQpLCBkYXRhPWRmX2VwZmwpDQpzdW1tYXJ5KG0yKQ0KYW5vdmEobTIpDQpgYGANCiMjIyMgQ29tcGFyZSBtb2RlbHMNCldoZW4gY29tcGFyaW5nIGEgbGluZWFyIG1vZGVsIChsbSkgdG8gYSBsaW5lYXIgbWl4ZWQtZWZmZWN0IG1vZGVsIChsbWUpLCB0aGUgcmVndWxhciBBTk9WQSBkb2VzIG5vdCB3b3JrLCBzbyBJIHdpbGwgb25seSBsb29rIGF0IHRoZSBpbmZvcm1hdGlvbiBjcml0ZXJpYQ0KYGBge3J9DQpBSUMobTEsIG0yKQ0KQklDKG0xLCBtMikNCmBgYA0KVGhlIG1vZGVsIGluY2x1ZGluZyByYW5kb20gaW50ZXJjZXB0cyBpcyBiZXR0ZXIgdGhhbiB0aGUgcmVndWxhciBsaW5lYXIgbW9kZWwgaW4gdGhpcyBjYXNlLg0KDQpSYW5kb20gaW50ZXJjZXB0IGFuZCByYW5kb20gc2xvcGUgcGVyIHN1YmplY3QNCmBgYHtyfQ0KbTMgPC0gbG1lcihmb3JtdWxhID0gU3BlZWRfY29yciB+IFN0aW0qVGFzaypCbG9jayArICgxICsgQmxvY2t8SUQpLCBkYXRhPWRmX2VwZmwpDQpzdW1tYXJ5KG0zKQ0KYW5vdmEobTMpDQpgYGANCiMjIyMgQ29tcGFyZSBtb2RlbHMsIGV2ZW4gdGhvdWdoIHRoaXMgbGFzdCBvbmUgZGlkIG5vdCBjb252ZXJnZQ0KYGBge3J9DQphbm92YShtMiwgbTMpDQpgYGANCiMjIyBOb3RlOg0KQWRkaW5nIGEgcmFuZG9tIHNsb3BlIGRvZXMgbm90IGltcHJvdmUgdGhlIGFtb3VudCBvZiB2YXJpYW5jZSBleHBsYWluZWQgYnkgdGhlIG1vZGVsLCBiZXNpZGVzIHRoZSBmYWN0IHRoYXQgdGhlIG1vZGVsIGluY2x1ZGluZyBhIHJhbmRvbSBzbG9wZSBkaWQgbm90IGNvbnZlcmdlLg0KDQojIyMgTW9kZWwgd2l0aCByYW5kb20gaW50ZXJjZXB0IHBlciBwYXJ0aWNpcGFudCBhbmQgcGVyIGRheSwgdHJ5aW5nIHRvIGFjY291bnQgZm9yIGRpZmZlcmVuY2VzIGJldHdlZW4gc2Vzc2lvbiBkYXlzDQpgYGB7cn0NCm00IDwtIGxtZXIoZm9ybXVsYSA9IFNwZWVkX2NvcnIgfiBTdGltKlRhc2sqQmxvY2sgKyAoMSArICgxfElEKSArICgxfERheSkpLCBkYXRhPWRmX2VwZmwpDQpzdW1tYXJ5KG00KQ0KYW5vdmEobTQpDQpgYGANCiMjIyMgQ29tcGFyZSBtb2RlbHMNCmBgYHtyfQ0KYW5vdmEobTIsIG00KQ0KYGBgDQoNCiMjIyBNb2RlbCBjaG9pY2UNClRoZSBtb2RlbCBpbmNsdWRpbmcgcmFuZG9tIGludGVyY2VwdHMgZm9yIGVhY2ggc3ViamVjdCBvbiBlYWNoIGRheSBzaWduaWZpY2FudGx5IGltcHJvdmVzIHRoZSBvbmUgd2l0aCBvbmx5IHJhbmRvbSBpbnRlcmNlcHRzIGZvciBpbmRpdmlkdWFscywgc28gdGhpcyBpcyB0aGUgDQptb2RlbCB3ZSB3aWxsIHVzZSBmb3IgdGhlIHNwZWVkLg0KDQoNCmBgYHtyfQ0KbV9zcGVlZCA8LSBsbWVyKGZvcm11bGEgPSBTcGVlZF9jb3JyIH4gU3RpbSpUYXNrKkJsb2NrICsgKDEgKyAoMXxJRCkgKyAoMXxEYXkpKSwgZGF0YT1kZl9lcGZsKQ0Kc3VtbWFyeShtX3NwZWVkKQ0KYW5vdmEobV9zcGVlZCkNCmV0YV9zcXVhcmVkKG1fc3BlZWQpIA0KYGBgDQpUaGVyZSBpcyBhIHNpZ25pZmljYW50IGVmZmVjdCBvZiBzdGltdWxhdGlvbiwgYXMgd2VsbCBhcyBhIHNpZ25pZmljYW50IGRpZmZlcmVuY2UgYmV0d2VlbiB0aGUgdGFza3MgYW5kIHRoZSBzbG9wZXMgZm9yIGVhY2ggdGFzay4gV2Ugd2lsbCBtYWtlIG1vZGVscyBmb3IgZWFjaA0KdGFzayBzZXBhcmF0ZWx5LiANCg0KIyMjIFNwZWVkIGluIHRoZSAyLWJhY2sgdGFzaw0KYGBge3J9DQpkYXRhX3N1YnNldCA8LSBzdWJzZXQoZGZfZXBmbCwgVGFzayA9PSAnMmJhY2snKQ0KZGF0YV9zdWJzZXQgPC0gZHJvcGxldmVscyhkYXRhX3N1YnNldCkNCmxldmVscyhkYXRhX3N1YnNldCRUYXNrKQ0KYGBgDQpgYGB7cn0NCm1fc3BlZWRfMmJhY2sgPC0gbG1lcihmb3JtdWxhID0gU3BlZWRfY29yciB+IFN0aW0qQmxvY2sgKyAoMSArICgxfElEKSArICgxfERheSkpLCBkYXRhPWRhdGFfc3Vic2V0KQ0Kc3VtbWFyeShtX3NwZWVkXzJiYWNrKQ0KYW5vdmEobV9zcGVlZF8yYmFjaykNCmV0YV9zcXVhcmVkKG1fc3BlZWRfMmJhY2spDQpgYGANClRoZXJlIGlzIGEgc2lnbmlmaWNhbnQgZWZmZWN0IG9mIHN0aW11bGF0aW9uLCBzbyB3ZSB3aWxsIG5vdyBkbyBwb3N0LWhvYyB0ZXN0cy4gVW5saWtlIHdoYXQgd2UgZGlkIGZvciB0aGUgbm9ybWFsaXplZCBhY2N1cmFjeSwgaGVyZSB3ZSB3aWxsIG9ubHkgZG8gdGhlDQpkZWZhdWx0IGNvbXBhcmlzb24gaGFsZndheSB0aHJvdWdoIHRoZSByZWdyZXNzaW9uIGxpbmVzLCBhcyB0aGUgZGF0YSBpcyBub3QgY29ycmVjdGVkIGZvciBkaWZmZXJlbmNlcyBpbiBpbml0aWFsIGxldmVscy4gV2Ugd2lsbCBhZGQgYSBjb21wYXJpc29uIG9mIHRoZSANCmxhc3QgdHJhaW5pbmcgYmxvY2ssIGlmIGFwcGxpY2FibGUsIHdoZW4gdGVzdGluZyB0aGUgbm9ybWFsaXplZCBkYXRhLg0KYGBge3J9DQpteV9tb2RlbCA8LSBtX3NwZWVkXzJiYWNrDQpsZXZlbF8xIDwtICJTdGltIg0KDQpteV9tb2RlbC5jb21wYXJlIDwtIGVtbWVhbnMobXlfbW9kZWwsIGxldmVsXzEsIGJ5PSJCbG9jayIpDQpteV9tb2RlbC5jb21wYXJlLnBhaXJzIDwtIHBhaXJzKG15X21vZGVsLmNvbXBhcmUsIGFkanVzdD0ndHVrZXknKQ0KdGVzdChteV9tb2RlbC5jb21wYXJlLnBhaXJzLCBzaWRlPSd0d28tc2lkZWQnKQ0KY29uZmludChteV9tb2RlbC5jb21wYXJlLCBjYWxjID0gYyhuID0gfi53Z3QuKSkNCmVmZl9zaXplKG15X21vZGVsLmNvbXBhcmUsIHNpZ21hID0gc2lnbWEobXlfbW9kZWwpLCBlZGYgPSAyMykNCmBgYA0KVGhlcmUgaXMgYSB0cmVuZCB0b3dhcmRzIHNpZ25pZmljYW5jZSBpbiB0aGUgY29tcGFyaXNvbiBiZXR3ZWVuIGdhbW1hLXByZWZyb250YWwtdGhldGEtcGFyaWV0YWwgYW5kIHRoZSBwbGFjZWJvLCBpbiB3aGljaCBwZW9wbGUgd291bGQgYmUgZmFzdGVyIHdoZW4gcmVjZWl2aW5nIA0Kc3RpbXVsYXRpb24uIA0KDQpTcGVlZCBpbiB0aGUgMy1iYWNrIHRhc2sNCmBgYHtyfQ0KZGF0YV9zdWJzZXQgPC0gc3Vic2V0KGRmX2VwZmwsIFRhc2sgPT0gJzNiYWNrJykNCmRhdGFfc3Vic2V0IDwtIGRyb3BsZXZlbHMoZGF0YV9zdWJzZXQpDQpsZXZlbHMoZGF0YV9zdWJzZXQkVGFzaykNCmBgYA0KYGBge3J9DQptX3NwZWVkXzNiYWNrIDwtIGxtZXIoZm9ybXVsYSA9IFNwZWVkX2NvcnIgfiBTdGltKkJsb2NrICsgKDEgKyAoMXxJRCkgKyAoMXxEYXkpKSwgZGF0YT1kYXRhX3N1YnNldCkNCnN1bW1hcnkobV9zcGVlZF8zYmFjaykNCmFub3ZhKG1fc3BlZWRfM2JhY2spDQpldGFfc3F1YXJlZChtX3NwZWVkXzNiYWNrKQ0KYGBgDQpUaGVyZSBpcyBhIHNpZ25pZmljYW50IGVmZmVjdCBvZiBzdGltdWxhdGlvbiBpbiB0aGUgMy1iYWNrIHRhc2sgYXMgd2VsbC4gTm93IHRoZSBwb3N0LWhvYyB0ZXN0czoNCmBgYHtyfQ0KbXlfbW9kZWwgPC0gbV9zcGVlZF8zYmFjaw0KbGV2ZWxfMSA8LSAiU3RpbSINCg0KbXlfbW9kZWwuY29tcGFyZSA8LSBlbW1lYW5zKG15X21vZGVsLCBsZXZlbF8xLCBieT0iQmxvY2siKQ0KbXlfbW9kZWwuY29tcGFyZS5wYWlycyA8LSBwYWlycyhteV9tb2RlbC5jb21wYXJlLCBhZGp1c3Q9J3R1a2V5JykNCnRlc3QobXlfbW9kZWwuY29tcGFyZS5wYWlycywgc2lkZT0ndHdvLXNpZGVkJykNCmNvbmZpbnQobXlfbW9kZWwuY29tcGFyZSwgY2FsYyA9IGMobiA9IH4ud2d0LikpDQplZmZfc2l6ZShteV9tb2RlbC5jb21wYXJlLCBzaWdtYSA9IHNpZ21hKG15X21vZGVsKSwgZWRmID0gMjMpDQpgYGANClRoZSByZXN1bHRzIHNob3cgcGFydGljaXBhbnRzIHdlcmUgc2lnbmlmaWNhbnRseSBmYXN0ZXIgd2hlbiByZWNlaXZpbmcgdGhldGEtcGFyaWV0YWwtZ2FtbWFidXJzdC1wcmVmcm9udGFsIGNvbXBhcmVkIHRvIHdoZW4gdGhleSByZWNlaXZlZCB0aGUgcGxhY2Viby4gSW4gdHVybiwgDQp0aGVpciBzcGVlZCB3YXMgc2lnbmlmaWNhbnRseSBoaWdoZXIgdG8gdGhlIG9uZSB0aGV5IGhhZCB3aGVuIHJlY2VpdmluZyB0aGV0YS1wcmVmcm9udGFsLWdhbW1hLXBhcmlldGFsIHN0aW11bGF0aW9uLg0KDQojIyBOb3JtYWxpemVkIFNwZWVkDQoNCkdyb3VwIGF2ZXJhZ2VzDQpgYGB7cn0NCm0wIDwtIGxtKGZvcm11bGEgPSBTcGVlZF9ub3JtIH4gU3RpbSpUYXNrLCBkYXRhPWRmX2VwZmwpDQpzdW1tYXJ5KG0wKQ0KYW5vdmEobTApDQpgYGANCg0KYGBge3J9DQptMSA8LSBsbShmb3JtdWxhID0gU3BlZWRfbm9ybSB+IFN0aW0qVGFzaypCbG9jaywgZGF0YT1kZl9lcGZsKQ0Kc3VtbWFyeShtMSkNCmFub3ZhKG0xKQ0KYGBgDQoNCkNvbXBhcmUgdGhlIG1vZGVscw0KYGBge3J9DQphbm92YShtMCwgbTEpDQpgYGANCiMjIyBOb3RlOg0KVGhlIG1vZGVsIGluY2x1ZGluZyB0aGUgYmxvY2tzIHNpZ25pZmljYW50bHkgaW1wcm92ZXMgdGhlIHByb3BvcnRpb24gb2YgdGhlIGRhdGEgZXhwbGFpbmVkIGJ5IHRoZSBtb2RlbC4gV2Ugd2lsbCBub3cgYXNzZXNzIHdoZXRoZXIgdGhlIGFkZGl0aW9uIG9mIHJhbmRvbSBlZmZlY3RzIA0KaW1wcm92ZXMgdGhlIGxpbmVhciBtb2RlbC4NCg0KSW5jbHVkZSByYW5kb20gZWZmZWN0cw0KUmFuZG9tIGludGVyY2VwdCBwZXIgc3ViamVjdA0KYGBge3J9DQptMiA8LSBsbWVyKGZvcm11bGEgPSBTcGVlZF9ub3JtIH4gU3RpbSpUYXNrKkJsb2NrICsgKDEgfCBJRCksIGRhdGE9ZGZfZXBmbCkNCnN1bW1hcnkobTIpDQphbm92YShtMikNCmBgYA0KV2hlbiBhZGRpbmcgYSByYW5kb20gaW50ZXJjZXB0IGZvciBlYWNoIHN1YmplY3QsIHRoZXJlIGlzIG5vIGxvbmdlciBhIHNpZ25pZmljYW50IGVmZmVjdCBvZiBvZiBzdGltdWxhdGlvbjsgdGhlIG9ubHkgcmVsZXZhbnQgZGlmZmVyZW5jZSB0aGF0IHJlbWFpbnMgaXMgYSBkaWZmZXJlbmNlDQppbiBzbG9wZSBhbW9uZyB0aGUgdGFza3MuIFdlIHdpbGwgbm93IGNvbXBhcmUgdGhlIG1vZGVscyB0byBzZWUgd2hpY2ggb25lIGlzIGEgYmV0dGVyIGZpdC4NCmBgYHtyfQ0KQUlDKG0xLCBtMikNCkJJQyhtMSwgbTIpDQpgYGANCg0KIyMjIE5vdGU6DQpUaGUgbW9kZWwgaW5jbHVkaW5nIHRoZSByYW5kb20gaW50ZXJjZXB0IGRvZXMgbm90IGltcHJvdmUgdGhlIGFtb3VudCBvZiB2YXJpYW5jZSBleHBsYWluZWQgYnkgdGhlIG1vZGVsLiANCmBgYHtyfQ0KbTMgPC0gbG1lcihmb3JtdWxhID0gU3BlZWRfbm9ybSB+IFN0aW0qVGFzaypCbG9jayArICgxICsgQmxvY2t8SUQpLCBkYXRhPWRmX2VwZmwpDQpzdW1tYXJ5KG0zKQ0KYW5vdmEobTMpDQpgYGANCg0KYGBge3J9DQpBSUMobTEsIG0zKQ0KQklDKG0xLCBtMykNCmBgYA0KIyMjIE5vdGU6DQpUaGUgbW9kZWwgaW5jbHVkaW5nIGEgcmFuZG9tIHNsb3BlIGZvciBpbmRpdmlkdWFsIGlzIHdvcnNlIHRoYW4gdGhlIGxpbmVhciBtb2RlbCB3aXRob3V0IGFueSByYW5kb20gZWZmZWN0cywgc28gdGhhdCdzIHRoZSBvbmUgd2Ugd2lsbCBrZWVwLg0KDQojIyMgQ2hlY2sgaWYgYWRkaW5nIGEgcmFuZG9tIGludGVyY2VwdCBmb3IgdGhlIGRheXMgaW1wcm92ZXMgdGhlIG1vZGVsDQpgYGB7cn0NCm00IDwtIGxtZXIoZm9ybXVsYSA9IFNwZWVkX25vcm0gfiBTdGltKlRhc2sqQmxvY2sgKyAoMSArICgxfElEKSArICgxfERheSkpLCBkYXRhPWRmX2VwZmwpDQpzdW1tYXJ5KG00KQ0KYW5vdmEobTQpDQpgYGANCmBgYHtyfQ0KQUlDKG0xLCBtNCkNCkJJQyhtMSwgbTQpDQpgYGANClRoZSBpbmNsdXNzaW9uIG9mIHJhbmRvbSBlZmZlY3RzIGlzIGluIHRoaXMgY2FzZSBub3QganVzdGlmaWVkLCBzbyB3ZSB3aWxsIGtlZXAgYSByZWd1bGFyIGxpbmVhciBtb2RlbC4NCg0KYGBge3J9DQptX3NwZWVkX25vcm0gPC0gbG0oZm9ybXVsYSA9IFNwZWVkX25vcm0gfiBTdGltKlRhc2sqQmxvY2ssIGRhdGE9ZGZfZXBmbCkNCnN1bW1hcnkobV9zcGVlZF9ub3JtKQ0KYW5vdmEobV9zcGVlZF9ub3JtKQ0KZXRhX3NxdWFyZWQobV9zcGVlZF9ub3JtKQ0KYGBgDQpUaGUgbW9kZWwgc2hvd3MgYSBzaWduaWZpY2FudCBlZmZlY3Qgb2Ygc3RpbXVsYXRpb24uDQoNCmBgYHtyfQ0KbXlfbW9kZWwgPC0gbV9zcGVlZF9ub3JtDQpsZXZlbF8xIDwtICJTdGltIg0KDQpteV9tb2RlbC5jb21wYXJlIDwtIGVtbWVhbnMobXlfbW9kZWwsIGxldmVsXzEsIGJ5PWMoIkJsb2NrIiwgIlRhc2siKSwgYXQgPSBsaXN0KEJsb2NrID0gYyg3LCAxMykpKQ0KbXlfbW9kZWwuY29tcGFyZS5wYWlycyA8LSBwYWlycyhteV9tb2RlbC5jb21wYXJlLCBhZGp1c3Q9J3R1a2V5JykNCnRlc3QobXlfbW9kZWwuY29tcGFyZS5wYWlycywgc2lkZT0ndHdvLXNpZGVkJykNCmNvbmZpbnQobXlfbW9kZWwuY29tcGFyZSwgY2FsYyA9IGMobiA9IH4ud2d0LikpDQplZmZfc2l6ZShteV9tb2RlbC5jb21wYXJlLCBzaWdtYSA9IHNpZ21hKG15X21vZGVsKSwgZWRmID0gMjMpDQpgYGANCk5vcm1hbGl6ZWQgc3BlZWQgZm9yIHRoZSAyLWJhY2sgdGFzaw0KYGBge3J9DQpkYXRhX3N1YnNldCA8LSBzdWJzZXQoZGZfZXBmbCwgVGFzayA9PSAnMmJhY2snKQ0KZGF0YV9zdWJzZXQgPC0gZHJvcGxldmVscyhkYXRhX3N1YnNldCkNCmxldmVscyhkYXRhX3N1YnNldCRUYXNrKQ0KYGBgDQpgYGB7cn0NCm1fc3BlZWRfbm9ybV8yYmFjayA8LSBsbShmb3JtdWxhID0gU3BlZWRfbm9ybSB+IFN0aW0qQmxvY2ssIGRhdGE9ZGF0YV9zdWJzZXQpDQpzdW1tYXJ5KG1fc3BlZWRfbm9ybV8yYmFjaykNCmFub3ZhKG1fc3BlZWRfbm9ybV8yYmFjaykNCmV0YV9zcXVhcmVkKG1fc3BlZWRfbm9ybV8yYmFjaykNCmBgYA0KYGBge3J9DQpteV9tb2RlbCA8LSBtX3NwZWVkX25vcm1fMmJhY2sNCmxldmVsXzEgPC0gIlN0aW0iDQoNCm15X21vZGVsLmNvbXBhcmUgPC0gZW1tZWFucyhteV9tb2RlbCwgbGV2ZWxfMSwgYnk9IkJsb2NrIikNCm15X21vZGVsLmNvbXBhcmUucGFpcnMgPC0gcGFpcnMobXlfbW9kZWwuY29tcGFyZSwgYWRqdXN0PSd0dWtleScpDQp0ZXN0KG15X21vZGVsLmNvbXBhcmUucGFpcnMsIHNpZGU9J3R3by1zaWRlZCcpDQpjb25maW50KG15X21vZGVsLmNvbXBhcmUsIGNhbGMgPSBjKG4gPSB+LndndC4pKQ0KZWZmX3NpemUobXlfbW9kZWwuY29tcGFyZSwgc2lnbWEgPSBzaWdtYShteV9tb2RlbCksIGVkZiA9IDIzKQ0KYGBgDQoNCjMtYmFjayB0ZXN0DQpgYGB7cn0NCmRhdGFfc3Vic2V0IDwtIHN1YnNldChkZl9lcGZsLCBUYXNrID09ICczYmFjaycpDQpkYXRhX3N1YnNldCA8LSBkcm9wbGV2ZWxzKGRhdGFfc3Vic2V0KQ0KbGV2ZWxzKGRhdGFfc3Vic2V0JFRhc2spDQpgYGANCmBgYHtyfQ0KbV9zcGVlZF9ub3JtXzNiYWNrIDwtIGxtKGZvcm11bGEgPSBTcGVlZF9ub3JtIH4gU3RpbSpCbG9jaywgZGF0YT1kYXRhX3N1YnNldCkNCnN1bW1hcnkobV9zcGVlZF9ub3JtXzNiYWNrKQ0KYW5vdmEobV9zcGVlZF9ub3JtXzNiYWNrKQ0KZXRhX3NxdWFyZWQobV9zcGVlZF9ub3JtXzNiYWNrKQ0KYGBgDQpgYGB7cn0NCm15X21vZGVsIDwtIG1fc3BlZWRfbm9ybV8zYmFjaw0KbGV2ZWxfMSA8LSAiU3RpbSINCg0KbXlfbW9kZWwuY29tcGFyZSA8LSBlbW1lYW5zKG15X21vZGVsLCBsZXZlbF8xLCBieT0iQmxvY2siKQ0KbXlfbW9kZWwuY29tcGFyZS5wYWlycyA8LSBwYWlycyhteV9tb2RlbC5jb21wYXJlLCBhZGp1c3Q9J3R1a2V5JykNCnRlc3QobXlfbW9kZWwuY29tcGFyZS5wYWlycywgc2lkZT0ndHdvLXNpZGVkJykNCmNvbmZpbnQobXlfbW9kZWwuY29tcGFyZSwgY2FsYyA9IGMobiA9IH4ud2d0LikpDQplZmZfc2l6ZShteV9tb2RlbC5jb21wYXJlLCBzaWdtYSA9IHNpZ21hKG15X21vZGVsKSwgZWRmID0gMjMpDQpgYGANCg0KIyMgU3RhdGlzdGljYWwgYW5hbHlzaXMgcmVsYXRpbmcgdGhlIGVzdGltYXRlZCBpbmR1Y2VkIGVsZWN0cmljIGZpZWxkcyBhbmQgYmVoYXZpb3IuDQpgYGB7cn0NCmZpbGVfbmFtZSA8LSBmaWxlLnBhdGgocGFzdGUoZGF0YV9kaXJlY3RvcnksICJCZWhhdmlvcmFsX2FuZF9lZmllbGRfYmxvY2tzX2lDT0dfRGVzaWduXzI1XzA0XzIwMjMudHh0Iiwgc2VwPScvJykpDQpkZl9lZmllbGRzIDwtIHJlYWQuZGVsaW0oZmlsZV9uYW1lLCBoZWFkZXIgPSBUUlVFLCBuYS5zdHJpbmdzID0gIk5OIikNCmhlYWQoZGZfZWZpZWxkcywgNSkNCmBgYA0KYGBge3J9DQpkZl9lZmllbGRzJElEIDwtIGFzLmZhY3RvcihkZl9lZmllbGRzJElEKQ0KZGZfZWZpZWxkcyRTY2hvb2wgPC0gYXMuZmFjdG9yKGRmX2VmaWVsZHMkU2Nob29sKQ0KZGZfZWZpZWxkcyREYXkgPC0gYXMuZmFjdG9yKGRmX2VmaWVsZHMkRGF5KQ0KZGZfZWZpZWxkcyRUYXNrIDwtIGFzLmZhY3RvcihkZl9lZmllbGRzJFRhc2spDQpkZl9lZmllbGRzJFN0aW0gPC0gYXMuZmFjdG9yKGRmX2VmaWVsZHMkU3RpbSkNCmRmX2VmaWVsZHMkQWNjX3UgPC0gYXMubnVtZXJpYyhkZl9lZmllbGRzJEFjY191KQ0KZGZfZWZpZWxkcyRTcGVlZF91IDwtIGFzLm51bWVyaWMoZGZfZWZpZWxkcyRTcGVlZF91KQ0KZGZfZWZpZWxkcyRBY2NfbSA8LSBhcy5udW1lcmljKGRmX2VmaWVsZHMkQWNjX20pDQpkZl9lZmllbGRzJEFjY19uX20gPC0gYXMubnVtZXJpYyhkZl9lZmllbGRzJEFjY19uX20pDQpkZl9lZmllbGRzJFNwZWVkX20gPC0gYXMubnVtZXJpYyhkZl9lZmllbGRzJFNwZWVkX20pDQpkZl9lZmllbGRzJFNwZWVkX25fbSA8LSBhcy5udW1lcmljKGRmX2VmaWVsZHMkU3BlZWRfbl9tKQ0KZGZfZWZpZWxkcyRFRl9mcm9udCA8LSBhcy5udW1lcmljKGRmX2VmaWVsZHMkRUZfZnJvbnQpDQpkZl9lZmllbGRzJEVGX3BhciA8LSBhcy5udW1lcmljKGRmX2VmaWVsZHMkRUZfcGFyKQ0KZGZfZWZpZWxkcyRFRl9tYWcgPC0gYXMubnVtZXJpYyhkZl9lZmllbGRzJEVGX21hZykNCmRmX2VmaWVsZHMkRXJyb3JfZnJvbnQgPC0gYXMubnVtZXJpYyhkZl9lZmllbGRzJEVycm9yX2Zyb250KQ0KZGZfZWZpZWxkcyRFcnJvcl9wYXIgPC0gYXMubnVtZXJpYyhkZl9lZmllbGRzJEVycm9yX3BhcikNCmBgYA0KYGBge3J9DQpkZl9lZl9lcGZsIDwtIHN1YnNldChkZl9lZmllbGRzLCBTY2hvb2wgPT0gJ0VQRkwnKQ0KZGZfZWZfZXBmbCA8LSBkcm9wbGV2ZWxzKGRmX2VmX2VwZmwpDQojbGV2ZWxzKGRmX2VmX2VwZmwkU2Nob29sKQ0KaGVhZChkZl9lZl9lcGZsLCA1KQ0KYGBgDQpgYGB7cn0NCmRmX2VmX2VwZmwkU3RpbSA8LSBmYWN0b3IoZGZfZWZfZXBmbCRTdGltLCBsZXZlbHMoZGZfZWZfZXBmbCRTdGltKVtjKDIsIDEsIDUsIDMsIDQpXSkNCmxldmVscyhkZl9lZl9lcGZsJFN0aW0pDQpgYGANCg0KIyMjIE5vdGUgYWJvdXQgdGhlIGFuYWx5c2lzDQpUaGUgaWRlYSBvZiB0aGlzIGFuYWx5c2lzIGlzIHRvIHZlcmlmeSB3aGV0aGVyIHRoZXJlIHdhcyBhIGRpZmZlcmVudGlhdGVkIGVmZmVjdCBvZiBzdGltdWxhdGlvbiByZXN1bHRpbmcgZnJvbSB0aGUgbWFnbml0dWRlIG9mIHRoZSBlbGVjdHJpYyBmaWVsZHMgaW5kdWNlZCBhdCBib3RoIHN0aW11bGF0aW9uIHNpdGVzIGF0IGFuIGluZGl2aWR1YWwgbGV2ZWwuIFRoZSBtYWluIGFuYWx5c2lzIGRlc2NyaWJlZCBhYm92ZSBzaG93ZWQgc2lnbmlmaWNhbnQgZWZmZWN0cyBvZiBzdGltdWxhdGlvbiBpbiB0aGUgbm9ybWFsaXplZCBkYXRhLCB3aGljaCBkZXBpY3RzIHJlbGF0aXZlIGNoYW5nZXMgaW4gcGVyZm9ybWFuY2UgZm9yIGVhY2ggaW5kaXZpZHVhbC4gRm9yIHRoaXMgcmVhc29uLCB3ZSB3aWxsIGFzc2VzcyB0aGUgZWZmZWN0IG9mIHRoZSBtYWduaXR1ZGUgb2YgdGhlIGluZHVjZWQgZWxlY3RyaWMgZmllbGRzIG9uIHRoZSBjaGFuZ2UgaW4gcGVyZm9ybWFuY2UgKGkuZS4sIGluIHRlcm1zIG9mIHNwZWVkIGFuZCBhY2N1cmFjeSkgd2l0aGluIGVhY2ggdHJhaW5pbmcgc2Vzc2lvbi4gVGhpcyBjaGFuZ2UgaW4gcGVyZm9ybWFuY2UgaXMgZGVzY3JpYmVkIGFzIHRoZSBzbG9wZSBvZiB0aGUgbGluZSBtaW5pbWl6aW5nIHRoZSBlcnJvciBpbiBhIGxlYXN0LXNxdWFyZWQgc2Vuc2UsIGZpdHRlZCB0byBlYWNoIGluZGl2aWR1YWwuIA0KDQpUaGlzIGRhdGEgZnJhbWUgY29udGFpbnMgdGhlIG1hZ25pdHVkZSBvZiB0aGUgZXN0aW1hdGVkIGVsZWN0cmljYWwgZmllbGQgZm9yIGVhY2ggZWxlY3Ryb2RlIChpLmUuLCBmcm9udGFsIGFuZCBwYXJpZXRhbCkuIFRoZSBlbGVjdHJvZGUgbG9jYXRpb25zIHdlcmUgZGV0ZXJtaW5lZCBieSBoYXZpbmcgcGFydGljaXBhbnRzIHBlcmZvcm0gdGhlIDItYmFjayB0YXNrIGluc2lkZSB0aGUgTVJJIHNjYW5uZXIgYW5kIGZpbmRpbmcgdGhlIHJlZ2lvbiB3ZXJlIHRoZSB0YXNrLXJlbGF0ZWQgYm9sZCBzaWduYWwgcGVha2VkLiBUaGlzIHJlZ2lvbnMgd2VyZSB0aGVuIHByb2plY3RlZCBpbnRvIHRoZSBzY2FscCBvZiBlYWNoIHBhcnRpY2lwYW50IGFuZCB0aGUgcmVzdWx0aW5nIGNvb3JkaW5hdGVzIHdlcmUgdXNlZCB0byBwbGFjZSB0aGUgZWxlY3Ryb2Rlcy4gSW4gdGhlIGRhdGEgZnJhbWUsIHRoZSBjb2x1bW5zIGxhYmVsZWQgYXMgIkVGIiBjb250YWluIHRoZSBtYWduaXR1ZGUgb2YgdGhlIGVsZWN0cmljIGZpZWxkIGVzdGltYXRlZCBmb3IgZWFjaCBpbnRlbmRlZCB0YXJnZXQsIHdoaWxlIHRoZSBjb2x1bW5zIGxhYmVsZWQgd2l0aCAiZXJyb3IiIGluY2x1ZGUgdGhlIGRpc3RhbmNlIGJldHdlZW4gdGhlIGludGVuZGVkIHRhcmdldCBhbmQgdGhlIGxvY2F0aW9uIHdlcmUgdGhlIG1heGltdW0gZWxlY3RyaWMgZmllbGQgd2FzIGluZHVjZWQuIFRoZSBjb2x1bW4gbGFiZWxlZCAiRUZfbWFnIiBjb250YWlucyB0aGUgbWFnbml0dWRlIG9mIHRoZSBlbGVjdHJpYyBmaWVsZCBpbmR1Y2VkIG9uIGJvdGggc2l0ZXMgKG1hZyA9IHNxcnQoYV4yICsgYl4yKSkuIFRoZSBjb2x1bW4gIkVGX3N5bSIgY29udGFpbnMgdGhlIHJhdGlvIG9mIHRoZSBlbGVjdHJpYyBmaWVsZCBpbmR1Y2VkIG9uIHRoZSBmcm9udGFsIHNpdGUgdG8gdGhlIGVsZWN0cmljIGZpZWxkIGluZHVjZWQgaW4gdGhlIHBhcmlldGFsIHNpdGUgKEVGX2Zyb250L0VGX3BhciksIGFuZCBjb25zdGl0dXRlcyBhIG1lYXN1cmUgb2YgaG93IHN5bW1ldHJpYyB0aGUgaW5kdWNlZCBmaWVsZHMgd2VyZS4gQnkgZGVzaWduIGFuZCB1bmRlciBpZGVhbCBjaXJjdW1zdGFuY2VzLCB0aGUgaW5kdWNlZCBmaWVsZHMgd291bGQgYmUgaWRlbnRpY2FsLCBidXQgYW5hdG9taWNhbCBkaWZmZXJlbmNlcyBhbW9uZyB0aGUgc2l0ZXMgb2Z0ZW4gcmVzdWx0IGluIGRpZmZlcmVudCBmaWVsZHMgYmVpbmcgaW5kdWNlZC4NCg0KSW4gdGhlIEVQRkwgY29ob3J0cywgdGhlIGludGVuc2l0eSBvZiB0aGUgYXBwbGllZCBjdXJyZW50IHdhcyBub3QgdGhlIHNhbWUgb24gYWxsIHNlc3Npb25zLiBJbiB0aGUgZmlyc3QgdGhyZWUgc2Vzc2lvbnMsIHRoZSBhcHBsaWVkIGludGVuc2l0eSB3YXMgb2YgMiBtQSBwZWFrLXRvLXBlYWsuIFRoZSBpbnRlbnNpdHkgbWVhbnQgdG8gYmUgYXBwbGllZCBpbiBzZXNzaW9ucyA0IGFuZCA1IHdhcyBvZiA0IG1BIHBlYWstdG8tcGVhazsgaG93ZXZlciwgbm90IGFsbCBwYXJ0aWNpcGFudHMgY291bGQgd2l0aHN0YW5kIHRoZSB0aW5nbGluZyBzZW5zYXRpb24gYXQgdGhpcyBpbnRlbnNpdHksIHdoaWNoIGlzIHdoeSBmb3IgdGhlc2UgcGFydGljaXBhbnRzIGxvd2VyIGN1cnJlbnRzIHdlcmUgdXNlZCwgYWRqdXN0ZWQgdG8gdGhlaXIgdG9sZXJhbmNlIHRocmVzaG9sZC4gVGhlIGVsZWN0cmljIGZpZWxkIHNpbXVsYXRpb25zIHdlcmUgZG9uZSBhY2NvcmRpbmcgdG8gdGhlIGludGVuc2l0eSBvZiB0aGUgYXBwbGllZCBjdXJyZW50cyBvbiBlYWNoIGRheSBmb3IgZWFjaCBwYXJ0aWNpcGFudC4gDQoNCg0KIyMjIFRoZSByZWxhdGlvbnNoaXAgYmV0d2VlbiB0aGUgaW50ZW5zaXR5IG9mIHRoZSBhcHBsaWVkIGVsZWN0cmljIGZpZWxkIG9uIHRoZSB0YXJnZXQgYW5kIHRoZSBhY2N1cmFjeQ0KDQpgYGB7cn0NCmRhdGFfc3Vic2V0IDwtIHN1YnNldChkZl9lZl9lcGZsLCBTdGltICE9ICdQbGFjZWJvJykNCmRhdGFfc3Vic2V0IDwtIGRyb3BsZXZlbHMoZGF0YV9zdWJzZXQpDQpsZXZlbHMoZGF0YV9zdWJzZXQkU3RpbSkNCmBgYA0KV2Ugd2lsbCBub3cgY2hvb3NlIHRoZSBtb2RlbCBmb2xsb3dpbmcgdGhlIHNhbWUgY3JpdGVyaWEgYXMgd2UgYXBwbGllZCB0byB0ZXN0IGFsbCB0aGUgcHJldmlvdXMgYXNwZWN0cy4NCg0KYGBge3J9DQptMCA8LSBsbShmb3JtdWxhID0gQWNjX20gfiBUYXNrKlN0aW0qRUZfZnJvbnQqRUZfcGFyLCBkYXRhPWRhdGFfc3Vic2V0KQ0Kc3VtbWFyeShtMCkNCmFub3ZhKG0wKQ0KYGBgDQpgYGB7cn0NCm0xIDwtIGxtZXIoZm9ybXVsYSA9IEFjY19tIH4gVGFzaypTdGltKkVGX2Zyb250KkVGX3BhciArICgxIHwgSUQpLCBkYXRhPWRhdGFfc3Vic2V0KQ0Kc3VtbWFyeShtMSkNCmFub3ZhKG0xKQ0KYGBgDQpgYGB7cn0NCkFJQyhtMCwgbTEpDQpCSUMobTAsIG0xKQ0KYGBgDQojIyMjIE1vZGVsIGNob2ljZQ0KVGhlIG1vZGVsIGluY2x1ZGluZyByYW5kb20gZWZmZWN0cyBkb2VzIG5vdCBpbXByb3ZlIHRoZSBtb2RlbCwgc28gd2Ugd2lsbCB1c2UgdGhlIHNpbXBsZXIgbW9kZWwgZm9yIHRoaXMgdGVzdC4NCmBgYHtyfQ0KbV9hY2NfZWYgPC0gbG0oZm9ybXVsYSA9IEFjY19tIH4gVGFzaypTdGltKkVGX2Zyb250KkVGX3BhciwgZGF0YT1kYXRhX3N1YnNldCkNCnN1bW1hcnkobV9hY2NfZWYpDQphbm92YShtX2FjY19lZikNCmV0YV9zcXVhcmVkKG1fYWNjX2VmKQ0KYGBgDQojIyMjIDItYmFjayB0YXNrDQpBcyB0aGVyZSBpcyBhIHNpZ25pZmljYW50IGRpZmZlcmVuY2UgYW1vbmcgdGhlIHRhc2tzIChhcyBpbiBldmVyeSBhc3BlY3QgdGVzdGVkIHByZXZpb3VzbHkpLCB3ZSB3aWxsIGZpdCBzZXBhcmF0ZSBtb2RlbHMgdG8gZWFjaCB0YXNrDQpgYGB7cn0NCmRhdGFfc3Vic2V0IDwtIHN1YnNldChkZl9lZl9lcGZsLCBUYXNrID09ICcyYmFjaycgJiBTdGltICE9ICdQbGFjZWJvJykNCmRhdGFfc3Vic2V0IDwtIGRyb3BsZXZlbHMoZGF0YV9zdWJzZXQpDQpsZXZlbHMoZGF0YV9zdWJzZXQkVGFzaykNCmxldmVscyhkYXRhX3N1YnNldCRTdGltKQ0KYGBgDQpgYGB7cn0NCm1fYWNjX2VmXzJiYWNrIDwtIGxtKGZvcm11bGEgPSBBY2NfbSB+IFN0aW0qRUZfZnJvbnQqRUZfcGFyLCBkYXRhPWRhdGFfc3Vic2V0KQ0Kc3VtbWFyeShtX2FjY19lZl8yYmFjaykNCmFub3ZhKG1fYWNjX2VmXzJiYWNrKQ0KYGBgDQpgYGB7cn0NCmRhdGFfc3Vic2V0IDwtIHN1YnNldChkZl9lZl9lcGZsLCBUYXNrID09ICczYmFjaycgJiBTdGltICE9ICdQbGFjZWJvJykNCmRhdGFfc3Vic2V0IDwtIGRyb3BsZXZlbHMoZGF0YV9zdWJzZXQpDQpsZXZlbHMoZGF0YV9zdWJzZXQkVGFzaykNCmxldmVscyhkYXRhX3N1YnNldCRTdGltKQ0KYGBgDQpgYGB7cn0NCm1fYWNjX2VmXzNiYWNrIDwtIGxtKGZvcm11bGEgPSBBY2NfbSB+IFN0aW0qRUZfZnJvbnQqRUZfcGFyLCBkYXRhPWRhdGFfc3Vic2V0KQ0Kc3VtbWFyeShtX2FjY19lZl8zYmFjaykNCmFub3ZhKG1fYWNjX2VmXzNiYWNrKQ0KZXRhX3NxdWFyZWQobV9hY2NfZWZfM2JhY2spDQpgYGANCiMjIyMgRXhwbG9yZSB0aGUgdHJpcGxlIGludGVyYWN0aW9ucw0KVGhlIGludGVycHJldGF0aW9uIG9mIHRyaXBsZSBpbnRlcmFjdGlvbnMgaXMgY29tcGxpY2F0ZWQsIGJ1dCB3ZSB3aWxsIHRyeSB0byBwcm92aWRlIGEgdmlzdWFsaXphdGlvbiBmb3IgZWFjaCBncm91cC4NCmBgYHtyfQ0Kbl9yb3dzID0gMTAwDQpjaG9vc2VfbW9kZWwgPC0gbV9hY2NfZWZfM2JhY2sNCnN0aW1fbGlzdCA8LSBsZXZlbHMoZGF0YV9zdWJzZXQkU3RpbSkNCmlfc3RhcnQgPSAwDQppX3N0b3AgPSAwLjMNCg0KY29sdW1ucyA9IGMoIlN0aW0iLCJUYXNrIiwiRUZfZnJvbnQiLCAiRUZfcGFyIikgDQpkZl90ZXN0ID0gZGF0YS5mcmFtZShtYXRyaXgobnJvdyA9IG5fcm93cywgbmNvbCA9IGxlbmd0aChjb2x1bW5zKSkpIA0KY29sbmFtZXMoZGZfdGVzdCkgPSBjb2x1bW5zDQoNCmNvbHVtbnMgPSBjKCJTdGltIiwiVGFzayIsIkVGX2Zyb250IiwgIkVGX3BhciIpIA0KZGZfdGVtcCA9IGRhdGEuZnJhbWUobWF0cml4KG5yb3cgPSBuX3Jvd3MsIG5jb2wgPSBsZW5ndGgoY29sdW1ucykpKSANCmNvbG5hbWVzKGRmX3RlbXApID0gY29sdW1ucw0KDQpmb3IgKHMgaW4gc3RpbV9saXN0KXsNCiAgZm9yICh0IGluIGMoIjNiYWNrIikpew0KICAgIGlmIChzID09IHN0aW1fbGlzdFsxXSAmIHQgPT0gJzNiYWNrJyl7DQogICAgZGZfdGVzdCRTdGltIDwtIHJlcChzLCBuX3Jvd3MpDQogICAgZGZfdGVzdCRFRl9mcm9udCA8LSByZXAoc2VxKGlfc3RhcnQsIGlfc3RvcCwgbGVuZ3RoLm91dCA9IDEwKSwgMTApDQogICAgZGZfdGVzdCRFRl9wYXIgPC0gcmVwKHNlcShpX3N0YXJ0LCBpX3N0b3AsIGxlbmd0aC5vdXQgPSAxMCksIGVhY2g9MTApDQogICAgI2RmX3Rlc3QkVGFzayA8LSBzYW1wbGUoYygnMmJhY2snLCAnM2JhY2snKSwgMTAwLCByZXBsYWNlPVRSVUUpDQogICAgZGZfdGVzdCRUYXNrIDwtIHJlcCh0LCAxMDApDQogICAgfQ0KICAgIGVsc2V7DQogICAgZGZfdGVtcCRTdGltIDwtIHJlcChzLCBuX3Jvd3MpDQogICAgZGZfdGVtcCRFRl9mcm9udCA8LSByZXAoc2VxKGlfc3RhcnQsIGlfc3RvcCwgbGVuZ3RoLm91dCA9IDEwKSwgMTApDQogICAgZGZfdGVtcCRFRl9wYXIgPC0gcmVwKHNlcShpX3N0YXJ0LCBpX3N0b3AsIGxlbmd0aC5vdXQgPSAxMCksIGVhY2g9MTApDQogICAgI2RmX3RlbXAkVGFzayA8LSBzYW1wbGUoYygnMmJhY2snLCAnM2JhY2snKSwgMTAwLCByZXBsYWNlPVRSVUUpDQogICAgZGZfdGVtcCRUYXNrIDwtIHJlcCh0LCAxMDApDQogICAgZGZfdGVzdCA8LSByYmluZChkZl90ZXN0LCBkZl90ZW1wKQ0KICAgIH0NCiAgfQ0KfQ0KYGBgDQoNCmBgYHtyfQ0KIyBNYWtlIHByZWRpY3Rpb25zDQpwcmVkcyA8LSBwcmVkaWN0KGNob29zZV9tb2RlbCwgZGZfdGVzdCkNCmRmX3Rlc3QkUHJlZCA8LSBwcmVkcw0KYGBgDQpgYGB7cn0NCiMgVmlld2luZyBhbmdsZXMgZm9yIHRoZSBwbG90cw0KYW5nbGVfY29sdW1ucyA9IGMoIlN0aW0iLCJBemltIiwiQ29sYXRpdHVkZSIpIA0KZGZfYW5nbGVzID0gZGF0YS5mcmFtZShtYXRyaXgobnJvdyA9IDQsIG5jb2wgPSBsZW5ndGgoYW5nbGVfY29sdW1ucykpKSANCmNvbG5hbWVzKGRmX2FuZ2xlcykgPSBhbmdsZV9jb2x1bW5zDQoNCmRmX2FuZ2xlcyRTdGltIDwtIHN0aW1fbGlzdA0KZGZfYW5nbGVzJEF6aW0gPC0gYygwLCAwLCAwLCAwKSAjIFRoZXRhLCBkZWZhdWx0IDANCmRmX2FuZ2xlcyRDb2xhdGl0dWRlIDwtIGMoMTUsIDE1LCAxNSwgMTUpICMgUGhpLCBkZWZhdWx0IDE1DQpgYGANCmBgYHtyfQ0KIyBNYWtlIHBsb3RzDQpmb3IgKHMgaW4gc3RpbV9saXN0KXsNCiAgZm9yICh0IGluIGMoIjNiYWNrIikpew0KICAgIGRpc3BfZGYgPC0gc3Vic2V0KGRmX3Rlc3QsIFN0aW0gPT0gcyAmIFRhc2sgPT0gdCkNCiAgICBwZXJzcCh4PXNlcShpX3N0YXJ0LCBpX3N0b3AsIGxlbmd0aC5vdXQgPSAxMCksIHk9c2VxKGlfc3RhcnQsIGlfc3RvcCwgbGVuZ3RoLm91dCA9IDEwKSwgej1tYXRyaXgoZGlzcF9kZiRQcmVkLCBucm93PTEwLCBuY29sPTEwLGJ5cm93PVRSVUUpLCANCiAgICAgICAgICB0aGV0YSA9IHN1YnNldChkZl9hbmdsZXMsIFN0aW0gPT0gcykkQXppbSwgcGhpID0gc3Vic2V0KGRmX2FuZ2xlcywgU3RpbSA9PSBzKSRDb2xhdGl0dWRlLA0KICAgICAgICAgIHhsYWI9IkVGX2Zyb250Iix5bGFiPSJFRl9wYXIiLHpsYWI9IlByZWRfQWNjX20iLCBtYWluPXBhc3RlKHMsdCksIHpsaW09YygtMC4xNiwgMC4xMikpIA0KICB9DQp9DQpgYGANCmBgYHtyfQ0KIyBFeHBvcnQgdGhlIGRhdGEgZm9yIHBsb3R0aW5nDQpzYXZlX2ZpbGVfbmFtZSA8LSBmaWxlLnBhdGgocGFzdGUoZGF0YV9kaXJlY3RvcnksICJFUEZMX0VmaWVsZF9pbnRlcmFjdGlvbnMuY3N2Iiwgc2VwPScvJykpDQp3cml0ZS5jc3YoZGZfdGVzdCwgc2F2ZV9maWxlX25hbWUpDQpgYGANCg0KIyMjIyBOb3RlDQpUaGUgcmVzdWx0cyBzaG93IGEgdHJpcGxlIGludGVyYWN0aW9uIG9mIHRoZSBzdGltdWxhdGlvbiB3aXRoIHRoZSBtYWduaXR1ZGUgb2YgdGhlIGVsZWN0cmljIGZpZWxkcyBhdCBib3RoIHN0aW11bGF0aW9uIHNpdGVzLiBBIGNvbXBhcmlzb24gb2YgdGhlIHJhdGUgb2YgaW1wcm92ZW1lbnQgaW4gYWNjdXJhY3kgaW4gdGhpcyBjb250ZXh0IGlzIG5vdCBzbyBzdHJhaWdodGZvcndhcmQsIGJ1dCBhIHZlcmJhdGltIGludGVycHJldGF0aW9uIGNvdWxkIGJlIHRoYXQgdGhlcmUgaXMgYSBjb3JyZWxhdGlvbiBiZXR3ZWVuIHRoZSBtYWduaXR1ZGUgb2YgYm90aCBlbGVjdHJpYyBmaWVsZHMgKHdoaWNoIGlzIGV4cGVjdGVkIGZyb20gdGhlIGRlc2lnbiksIGFuZCBhIGNvcnJlbGF0aW9uIGJldHdlZW4gdGhlIHN0aW11bGF0aW9uIGdyb3VwcyBhbmQgdGhlc2UgZmllbGRzLiBUbyB0ZXN0IHRoaXMgcmVsYXRpb25zaGlwLCB3ZSB3aWxsIHVzZSBhbiBhZGRpdGlvbmFsIG1lYXN1cmUgd2UgZHJldyAoZGVzY3JpYmVkIGF0IHRoZSBiZWdpbm5pbmcgb2YgdGhpcyBzZWN0aW9uKSwgd2hpY2ggd2UgZHViICJzeW1tZXRyeSIuIEFzIHRoZSBlbGVjdHJpYyBmaWVsZHMgYXJlIGNvcnJlbGF0ZWQgYW5kLCBmb2xsb3dpbmcgdGhlIGh5cG90aGVzZXMganVzdGlmeWluZyB0aGUgYXBwbGljYXRpb24gb2YgYmlmb2NhbCBzdGltdWxhdGlvbiB3aXRoaW4gdGhpcyBzdHVkeSwgd2UgY291bGQgZXhwZWN0IGZvciB0aGUgZWZmZWN0cyBvZiBzdGltdWxhdGlvbiB0byBiZSBtYXhpbXVtIHdoZW4gdGhlIG1hZ25pdHVkZSBvZiB0aGUgaW5kdWNlZCBlbGVjdHJpYyBmaWVsZHMgaXMgY29tcGFyYWJsZSBhbW9uZyBzaXRlcyAoaS5lLiwgdGhlIGFwcGxpY2F0aW9uIGlzIHN5bW1ldHJpYywgc28gc3ltbWV0cnkgPSAxKSwgYW5kIHdoZW4gdGhlIG1hZ25pdHVkZSBvZiB0aGUgaW5kdWNlZCBmaWVsZCAoRUZfbWFnKSBhcHByb2FjaGVzIGEgbWF4aW11bSBjYXBwZWQgYnkgdGhlIG1hZ25pdHVkZSBvZiB0aGUgYXBwbGllZCBjdXJyZW50LiBBcyBzdWNoLCB3ZSB3aWxsIG5vdyBhc3Nlc3MgdGhlIGVmZmVjdHMgb2YgdGhlc2UgY29tYmluZWQgbWVhc3VyZXMgb24gdGhlIGNoYW5nZSBpbiBwZXJmb3JtYW5jZS4NCg0KIyMjIyBTeW1tZXRyeQ0KDQpgYGB7cn0NCmRhdGFfc3Vic2V0IDwtIHN1YnNldChkZl9lZl9lcGZsLCBTdGltICE9ICdQbGFjZWJvJykNCmRhdGFfc3Vic2V0IDwtIGRyb3BsZXZlbHMoZGF0YV9zdWJzZXQpDQpsZXZlbHMoZGF0YV9zdWJzZXQkU3RpbSkNCmBgYA0KYGBge3J9DQptMCA8LSBsbShmb3JtdWxhID0gQWNjX20gfiBUYXNrKlN0aW0qRUZfc3ltLCBkYXRhPWRhdGFfc3Vic2V0KQ0Kc3VtbWFyeShtMCkNCmFub3ZhKG0wKQ0KYGBgDQpgYGB7cn0NCm0xIDwtIGxtZXIoZm9ybXVsYSA9IEFjY19tIH4gVGFzaypTdGltKkVGX3N5bSArICgxIHwgSUQpLCBkYXRhPWRhdGFfc3Vic2V0KQ0Kc3VtbWFyeShtMSkNCmFub3ZhKG0xKQ0KYGBgDQpgYGB7cn0NCkFJQyhtMCwgbTEpDQpCSUMobTAsIG0xKQ0KYGBgDQpgYGB7cn0NCm1fYWNjX21fc3ltIDwtIGxtKGZvcm11bGEgPSBBY2NfbSB+IFRhc2sqU3RpbSpFRl9zeW0sIGRhdGE9ZGF0YV9zdWJzZXQpDQpzdW1tYXJ5KG1fYWNjX21fc3ltKQ0KYW5vdmEobV9hY2NfbV9zeW0pDQpgYGANCmBgYHtyfQ0KZGF0YV9zdWJzZXQgPC0gc3Vic2V0KGRmX2VmX2VwZmwsIFRhc2sgPT0gJzJiYWNrJyAmIFN0aW0gIT0gJ1BsYWNlYm8nKQ0KZGF0YV9zdWJzZXQgPC0gZHJvcGxldmVscyhkYXRhX3N1YnNldCkNCmxldmVscyhkYXRhX3N1YnNldCRUYXNrKQ0KbGV2ZWxzKGRhdGFfc3Vic2V0JFN0aW0pDQpgYGANCmBgYHtyfQ0KbV9hY2NfbV9zeW1fMmJhY2sgPC0gbG0oZm9ybXVsYSA9IEFjY19tIH4gU3RpbSpFRl9zeW0sIGRhdGE9ZGF0YV9zdWJzZXQpDQpzdW1tYXJ5KG1fYWNjX21fc3ltXzJiYWNrKQ0KYW5vdmEobV9hY2NfbV9zeW1fMmJhY2spDQpgYGANCmBgYHtyfQ0KZGF0YV9zdWJzZXQgPC0gc3Vic2V0KGRmX2VmX2VwZmwsIFRhc2sgPT0gJzNiYWNrJyAmIFN0aW0gIT0gJ1BsYWNlYm8nKQ0KZGF0YV9zdWJzZXQgPC0gZHJvcGxldmVscyhkYXRhX3N1YnNldCkNCmxldmVscyhkYXRhX3N1YnNldCRUYXNrKQ0KbGV2ZWxzKGRhdGFfc3Vic2V0JFN0aW0pDQpgYGANCmBgYHtyfQ0KbV9hY2NfbV9zeW1fM2JhY2sgPC0gbG0oZm9ybXVsYSA9IEFjY19tIH4gU3RpbSpFRl9zeW0sIGRhdGE9ZGF0YV9zdWJzZXQpDQpzdW1tYXJ5KG1fYWNjX21fc3ltXzNiYWNrKQ0KYW5vdmEobV9hY2NfbV9zeW1fM2JhY2spDQpgYGANCiMjIyMgRS1maWVsZCBtYWduaXR1ZGUNClRoZSByZXN1bHRzIGZvciB0aGUgdGVzdHMgb24gdGhlIHN5bW1ldHJ5IG9mIHRoZSBhcHBsaWVkIG1hZ25ldGljIGZpZWxkcyBkaWQgbm90IHlpZWxkIHN0YXRpc3RpY2FsbHkgc2lnbmlmaWNhbnQgcmVzdWx0cywgd2hpY2ggbWF5IHN1Z2dlc3QgdGhpcyBtZWFzdXJlIGlzIG5vdCBjYXB0dXJpbmcgdGhlIGludGVyYWN0aW9uIHdlIHNhdyB3aGVuIGluY2x1ZGluZyB0aGUgbWFnbml0dWRlIG9mIGJvdGggZWxlY3RyaWMgZmllbGRzIHNlcGFyYXRlbHkuIEEgcG9zc2libGUgZXhwbGFuYXRpb24gZm9yIHRoaXMgaXMgdGhhdCB3aGF0J3MgaGF2aW5nIGEgc2lnbmlmaWNhbnQgZWZmZWN0IG9uIHRoZSByYXRlIG9mIGNoYW5nZSBpbiBhY2N1cmFjeSBpcyBub3QgdGhlIHJhdGlvIG9mIHRoZSBpbmR1Y2VkIGZpZWxkIGluIG9uZSBzaXRlIHdpdGggcmVzcGVjdCB0byB0aGUgb3RoZXIsIGJ1dCByYXRoZXIgdGhlIHRvdGFsIGFtb3VudCBvZiBlbmVyZ3kgdGhhdCdzIGJlaW5nIGluamVjdGVkLiBOZXh0LCB3ZSB3aWxsIHVzZSB0aGUgdG90YWwgbWFnbml0dWRlIG9mIHRoZSBjb21iaW5lZCBlbGVjdHJpYyBmaWVsZHMgYXMgYSBwcmVkaWN0b3IgZm9yIGNoYW5nZXMgaW4gcGVyZm9ybWFuY2UuDQpgYGB7cn0NCmRhdGFfc3Vic2V0IDwtIHN1YnNldChkZl9lZl9lcGZsLCBTdGltICE9ICdQbGFjZWJvJykNCmRhdGFfc3Vic2V0IDwtIGRyb3BsZXZlbHMoZGF0YV9zdWJzZXQpDQpsZXZlbHMoZGF0YV9zdWJzZXQkU3RpbSkNCmBgYA0KYGBge3J9DQptMCA8LSBsbShmb3JtdWxhID0gQWNjX20gfiBUYXNrKlN0aW0qRUZfbWFnLCBkYXRhPWRhdGFfc3Vic2V0KQ0Kc3VtbWFyeShtMCkNCmFub3ZhKG0wKQ0KYGBgDQpgYGB7cn0NCm0xIDwtIGxtZXIoZm9ybXVsYSA9IEFjY19tIH4gVGFzaypTdGltKkVGX21hZyArICgxIHwgSUQpLCBkYXRhPWRhdGFfc3Vic2V0KQ0Kc3VtbWFyeShtMSkNCmFub3ZhKG0xKQ0KYGBgDQpgYGB7cn0NCkFJQyhtMCwgbTEpDQpCSUMobTAsIG0xKQ0KYGBgDQpgYGB7cn0NCm1fYWNjX21fbWFnIDwtIGxtKGZvcm11bGEgPSBBY2NfbSB+IFRhc2sqU3RpbSpFRl9tYWcsIGRhdGE9ZGF0YV9zdWJzZXQpDQpzdW1tYXJ5KG1fYWNjX21fbWFnKQ0KYW5vdmEobV9hY2NfbV9tYWcpDQpgYGANCmBgYHtyfQ0KZGF0YV9zdWJzZXQgPC0gc3Vic2V0KGRmX2VmX2VwZmwsIFRhc2sgPT0gJzJiYWNrJyAmIFN0aW0gIT0gJ1BsYWNlYm8nKQ0KZGF0YV9zdWJzZXQgPC0gZHJvcGxldmVscyhkYXRhX3N1YnNldCkNCmxldmVscyhkYXRhX3N1YnNldCRUYXNrKQ0KbGV2ZWxzKGRhdGFfc3Vic2V0JFN0aW0pDQpgYGANCmBgYHtyfQ0KbV9hY2NfbV9tYWdfMmJhY2sgPC0gbG0oZm9ybXVsYSA9IEFjY19tIH4gU3RpbSpFRl9tYWcsIGRhdGE9ZGF0YV9zdWJzZXQpDQpzdW1tYXJ5KG1fYWNjX21fbWFnXzJiYWNrKQ0KYW5vdmEobV9hY2NfbV9tYWdfMmJhY2spDQpgYGANCmBgYHtyfQ0KZGF0YV9zdWJzZXQgPC0gc3Vic2V0KGRmX2VmX2VwZmwsIFRhc2sgPT0gJzNiYWNrJyAmIFN0aW0gIT0gJ1BsYWNlYm8nKQ0KZGF0YV9zdWJzZXQgPC0gZHJvcGxldmVscyhkYXRhX3N1YnNldCkNCmxldmVscyhkYXRhX3N1YnNldCRUYXNrKQ0KbGV2ZWxzKGRhdGFfc3Vic2V0JFN0aW0pDQpgYGANCmBgYHtyfQ0KbV9hY2NfbV9tYWdfM2JhY2sgPC0gbG0oZm9ybXVsYSA9IEFjY19tIH4gU3RpbSpFRl9tYWcsIGRhdGE9ZGF0YV9zdWJzZXQpDQpzdW1tYXJ5KG1fYWNjX21fbWFnXzNiYWNrKQ0KYW5vdmEobV9hY2NfbV9tYWdfM2JhY2spDQpgYGANCiMjIyBOb3JtYWxpemVkIGFjY3VyYWN5IGFuZCB0aGUgbWFnbml0dWRlIG9mIHRoZSBpbmR1Y2VkIGVsZWN0cmljIGZpZWxkcw0KYGBge3J9DQpkYXRhX3N1YnNldCA8LSBzdWJzZXQoZGZfZWZfZXBmbCwgU3RpbSAhPSAnUGxhY2VibycpDQpkYXRhX3N1YnNldCA8LSBkcm9wbGV2ZWxzKGRhdGFfc3Vic2V0KQ0KbGV2ZWxzKGRhdGFfc3Vic2V0JFN0aW0pDQpgYGANCmBgYHtyfQ0KbTAgPC0gbG0oZm9ybXVsYSA9IEFjY19uX20gfiBUYXNrKlN0aW0qRUZfZnJvbnQqRUZfcGFyLCBkYXRhPWRhdGFfc3Vic2V0KQ0Kc3VtbWFyeShtMCkNCmFub3ZhKG0wKQ0KYGBgDQpgYGB7cn0NCm0xIDwtIGxtZXIoZm9ybXVsYSA9IEFjY19uX20gfiBUYXNrKlN0aW0qRUZfZnJvbnQqRUZfcGFyICsgKDEgfCBJRCksIGRhdGE9ZGF0YV9zdWJzZXQpDQpzdW1tYXJ5KG0xKQ0KYW5vdmEobTEpDQpgYGANCmBgYHtyfQ0KQUlDKG0wLCBtMSkNCkJJQyhtMCwgbTEpDQpgYGANCmBgYHtyfQ0KbV9hY2Nfbl9lZiA8LSBsbShmb3JtdWxhID0gQWNjX25fbSB+IFRhc2sqU3RpbSpFRl9mcm9udCpFRl9wYXIsIGRhdGE9ZGF0YV9zdWJzZXQpDQpzdW1tYXJ5KG1fYWNjX25fZWYpDQphbm92YShtX2FjY19uX2VmKQ0KYGBgDQpgYGB7cn0NCmRhdGFfc3Vic2V0IDwtIHN1YnNldChkZl9lZl9lcGZsLCBUYXNrID09ICcyYmFjaycgJiBTdGltICE9ICdQbGFjZWJvJykNCmRhdGFfc3Vic2V0IDwtIGRyb3BsZXZlbHMoZGF0YV9zdWJzZXQpDQpsZXZlbHMoZGF0YV9zdWJzZXQkVGFzaykNCmxldmVscyhkYXRhX3N1YnNldCRTdGltKQ0KYGBgDQpgYGB7cn0NCm1fYWNjX25fZWZfMmJhY2sgPC0gbG0oZm9ybXVsYSA9IEFjY19uX20gfiBTdGltKkVGX2Zyb250KkVGX3BhciwgZGF0YT1kYXRhX3N1YnNldCkNCnN1bW1hcnkobV9hY2Nfbl9lZl8yYmFjaykNCmFub3ZhKG1fYWNjX25fZWZfMmJhY2spDQpgYGANCmBgYHtyfQ0KZGF0YV9zdWJzZXQgPC0gc3Vic2V0KGRmX2VmX2VwZmwsIFRhc2sgPT0gJzNiYWNrJyAmIFN0aW0gIT0gJ1BsYWNlYm8nKQ0KZGF0YV9zdWJzZXQgPC0gZHJvcGxldmVscyhkYXRhX3N1YnNldCkNCmxldmVscyhkYXRhX3N1YnNldCRUYXNrKQ0KbGV2ZWxzKGRhdGFfc3Vic2V0JFN0aW0pDQpgYGANCmBgYHtyfQ0KbV9hY2Nfbl9lZl8zYmFjayA8LSBsbShmb3JtdWxhID0gQWNjX25fbSB+IFN0aW0qRUZfZnJvbnQqRUZfcGFyLCBkYXRhPWRhdGFfc3Vic2V0KQ0Kc3VtbWFyeShtX2FjY19uX2VmXzNiYWNrKQ0KYW5vdmEobV9hY2Nfbl9lZl8zYmFjaykNCmBgYA0KIyMjIyBFeHBsb3JlIHRoZSB0cmlwbGUgaW50ZXJhY3Rpb24NCmBgYHtyfQ0Kbl9yb3dzID0gMTAwDQpjaG9vc2VfbW9kZWwgPC0gbV9hY2Nfbl9lZl8zYmFjaw0Kc3RpbV9saXN0IDwtIGxldmVscyhkYXRhX3N1YnNldCRTdGltKQ0KaV9zdGFydCA9IDANCmlfc3RvcCA9IDAuMw0KDQpjb2x1bW5zID0gYygiU3RpbSIsIlRhc2siLCJFRl9mcm9udCIsICJFRl9wYXIiKSANCmRmX3Rlc3QgPSBkYXRhLmZyYW1lKG1hdHJpeChucm93ID0gbl9yb3dzLCBuY29sID0gbGVuZ3RoKGNvbHVtbnMpKSkgDQpjb2xuYW1lcyhkZl90ZXN0KSA9IGNvbHVtbnMNCg0KY29sdW1ucyA9IGMoIlN0aW0iLCJUYXNrIiwiRUZfZnJvbnQiLCAiRUZfcGFyIikgDQpkZl90ZW1wID0gZGF0YS5mcmFtZShtYXRyaXgobnJvdyA9IG5fcm93cywgbmNvbCA9IGxlbmd0aChjb2x1bW5zKSkpIA0KY29sbmFtZXMoZGZfdGVtcCkgPSBjb2x1bW5zDQoNCmZvciAocyBpbiBzdGltX2xpc3Qpew0KICBmb3IgKHQgaW4gYygiM2JhY2siKSl7DQogICAgaWYgKHMgPT0gc3RpbV9saXN0WzFdICYgdCA9PSAnM2JhY2snKXsNCiAgICBkZl90ZXN0JFN0aW0gPC0gcmVwKHMsIG5fcm93cykNCiAgICBkZl90ZXN0JEVGX2Zyb250IDwtIHJlcChzZXEoaV9zdGFydCwgaV9zdG9wLCBsZW5ndGgub3V0ID0gMTApLCAxMCkNCiAgICBkZl90ZXN0JEVGX3BhciA8LSByZXAoc2VxKGlfc3RhcnQsIGlfc3RvcCwgbGVuZ3RoLm91dCA9IDEwKSwgZWFjaD0xMCkNCiAgICAjZGZfdGVzdCRUYXNrIDwtIHNhbXBsZShjKCcyYmFjaycsICczYmFjaycpLCAxMDAsIHJlcGxhY2U9VFJVRSkNCiAgICBkZl90ZXN0JFRhc2sgPC0gcmVwKHQsIDEwMCkNCiAgICB9DQogICAgZWxzZXsNCiAgICBkZl90ZW1wJFN0aW0gPC0gcmVwKHMsIG5fcm93cykNCiAgICBkZl90ZW1wJEVGX2Zyb250IDwtIHJlcChzZXEoaV9zdGFydCwgaV9zdG9wLCBsZW5ndGgub3V0ID0gMTApLCAxMCkNCiAgICBkZl90ZW1wJEVGX3BhciA8LSByZXAoc2VxKGlfc3RhcnQsIGlfc3RvcCwgbGVuZ3RoLm91dCA9IDEwKSwgZWFjaD0xMCkNCiAgICAjZGZfdGVtcCRUYXNrIDwtIHNhbXBsZShjKCcyYmFjaycsICczYmFjaycpLCAxMDAsIHJlcGxhY2U9VFJVRSkNCiAgICBkZl90ZW1wJFRhc2sgPC0gcmVwKHQsIDEwMCkNCiAgICBkZl90ZXN0IDwtIHJiaW5kKGRmX3Rlc3QsIGRmX3RlbXApDQogICAgfQ0KICB9DQp9DQpgYGANCg0KYGBge3J9DQojIE1ha2UgcHJlZGljdGlvbnMNCnByZWRzIDwtIHByZWRpY3QoY2hvb3NlX21vZGVsLCBkZl90ZXN0KQ0KZGZfdGVzdCRQcmVkIDwtIHByZWRzDQpgYGANCmBgYHtyfQ0KIyBWaWV3aW5nIGFuZ2xlcyBmb3IgdGhlIHBsb3RzDQphbmdsZV9jb2x1bW5zID0gYygiU3RpbSIsIkF6aW0iLCJDb2xhdGl0dWRlIikgDQpkZl9hbmdsZXMgPSBkYXRhLmZyYW1lKG1hdHJpeChucm93ID0gNCwgbmNvbCA9IGxlbmd0aChhbmdsZV9jb2x1bW5zKSkpIA0KY29sbmFtZXMoZGZfYW5nbGVzKSA9IGFuZ2xlX2NvbHVtbnMNCg0KZGZfYW5nbGVzJFN0aW0gPC0gc3RpbV9saXN0DQpkZl9hbmdsZXMkQXppbSA8LSBjKDAsIDAsIDAsIDApICMgVGhldGEsIGRlZmF1bHQgMA0KZGZfYW5nbGVzJENvbGF0aXR1ZGUgPC0gYygxNSwgMTUsIDE1LCAxNSkgIyBQaGksIGRlZmF1bHQgMTUNCmBgYA0KYGBge3J9DQojIE1ha2UgcGxvdHMNCmZvciAocyBpbiBzdGltX2xpc3Qpew0KICBmb3IgKHQgaW4gYygiM2JhY2siKSl7DQogICAgZGlzcF9kZiA8LSBzdWJzZXQoZGZfdGVzdCwgU3RpbSA9PSBzICYgVGFzayA9PSB0KQ0KICAgIHBlcnNwKHg9c2VxKGlfc3RhcnQsIGlfc3RvcCwgbGVuZ3RoLm91dCA9IDEwKSwgeT1zZXEoaV9zdGFydCwgaV9zdG9wLCBsZW5ndGgub3V0ID0gMTApLCB6PW1hdHJpeChkaXNwX2RmJFByZWQsIG5yb3c9MTAsIG5jb2w9MTAsYnlyb3c9VFJVRSksIA0KICAgICAgICAgIHRoZXRhID0gc3Vic2V0KGRmX2FuZ2xlcywgU3RpbSA9PSBzKSRBemltLCBwaGkgPSBzdWJzZXQoZGZfYW5nbGVzLCBTdGltID09IHMpJENvbGF0aXR1ZGUsDQogICAgICAgICAgeGxhYj0iRUZfZnJvbnQiLHlsYWI9IkVGX3BhciIsemxhYj0iUHJlZF9BY2Nfbl9tIiwgbWFpbj1wYXN0ZShzLHQpLCB6bGltPWMoLTAuMTYsIDAuMikpIA0KICB9DQp9DQpgYGANCg0KDQojIyMgU3ltbWV0cnkNCmBgYHtyfQ0KZGF0YV9zdWJzZXQgPC0gc3Vic2V0KGRmX2VmX2VwZmwsIFN0aW0gIT0gJ1BsYWNlYm8nKQ0KZGF0YV9zdWJzZXQgPC0gZHJvcGxldmVscyhkYXRhX3N1YnNldCkNCmxldmVscyhkYXRhX3N1YnNldCRTdGltKQ0KYGBgDQpgYGB7cn0NCm0wIDwtIGxtKGZvcm11bGEgPSBBY2Nfbl9tIH4gVGFzaypTdGltKkVGX3N5bSwgZGF0YT1kYXRhX3N1YnNldCkNCnN1bW1hcnkobTApDQphbm92YShtMCkNCmBgYA0KYGBge3J9DQptMSA8LSBsbWVyKGZvcm11bGEgPSBBY2Nfbl9tIH4gVGFzaypTdGltKkVGX3N5bSArICgxIHwgSUQpLCBkYXRhPWRhdGFfc3Vic2V0KQ0Kc3VtbWFyeShtMSkNCmFub3ZhKG0xKQ0KYGBgDQpgYGB7cn0NCkFJQyhtMCwgbTEpDQpCSUMobTAsIG0xKQ0KYGBgDQpgYGB7cn0NCm1fYWNjX25fZWZfc3ltIDwtIGxtKGZvcm11bGEgPSBBY2Nfbl9tIH4gVGFzaypTdGltKkVGX3N5bSwgZGF0YT1kYXRhX3N1YnNldCkNCnN1bW1hcnkobV9hY2Nfbl9lZl9zeW0pDQphbm92YShtX2FjY19uX2VmX3N5bSkNCmBgYA0KYGBge3J9DQpkYXRhX3N1YnNldCA8LSBzdWJzZXQoZGZfZWZfZXBmbCwgVGFzayA9PSAnMmJhY2snICYgU3RpbSAhPSAnUGxhY2VibycpDQpkYXRhX3N1YnNldCA8LSBkcm9wbGV2ZWxzKGRhdGFfc3Vic2V0KQ0KbGV2ZWxzKGRhdGFfc3Vic2V0JFRhc2spDQpsZXZlbHMoZGF0YV9zdWJzZXQkU3RpbSkNCmBgYA0KYGBge3J9DQptX2FjY19uX2VmX3N5bV8yYmFjayA8LSBsbShmb3JtdWxhID0gQWNjX25fbSB+IFN0aW0qRUZfc3ltLCBkYXRhPWRhdGFfc3Vic2V0KQ0Kc3VtbWFyeShtX2FjY19uX2VmX3N5bV8yYmFjaykNCmFub3ZhKG1fYWNjX25fZWZfc3ltXzJiYWNrKQ0KYGBgDQpgYGB7cn0NCmRhdGFfc3Vic2V0IDwtIHN1YnNldChkZl9lZl9lcGZsLCBUYXNrID09ICczYmFjaycgJiBTdGltICE9ICdQbGFjZWJvJykNCmRhdGFfc3Vic2V0IDwtIGRyb3BsZXZlbHMoZGF0YV9zdWJzZXQpDQpsZXZlbHMoZGF0YV9zdWJzZXQkVGFzaykNCmxldmVscyhkYXRhX3N1YnNldCRTdGltKQ0KYGBgDQpgYGB7cn0NCm1fYWNjX25fZWZfc3ltXzNiYWNrIDwtIGxtKGZvcm11bGEgPSBBY2Nfbl9tIH4gU3RpbSpFRl9zeW0sIGRhdGE9ZGF0YV9zdWJzZXQpDQpzdW1tYXJ5KG1fYWNjX25fZWZfc3ltXzNiYWNrKQ0KYW5vdmEobV9hY2Nfbl9lZl9zeW1fM2JhY2spDQpgYGANCiMjIyBOb3JtYWxpemVkIGFjY3VyYWN5IGFuZCBtYWduaXR1ZGUgb2YgY29tYmluZWQgZWxlY3RyaWMgZmllbGRzDQpgYGB7cn0NCmRhdGFfc3Vic2V0IDwtIHN1YnNldChkZl9lZl9lcGZsLCBTdGltICE9ICdQbGFjZWJvJykNCmRhdGFfc3Vic2V0IDwtIGRyb3BsZXZlbHMoZGF0YV9zdWJzZXQpDQpsZXZlbHMoZGF0YV9zdWJzZXQkU3RpbSkNCmBgYA0KYGBge3J9DQptMCA8LSBsbShmb3JtdWxhID0gQWNjX25fbSB+IFRhc2sqU3RpbSpFRl9tYWcsIGRhdGE9ZGF0YV9zdWJzZXQpDQpzdW1tYXJ5KG0wKQ0KYW5vdmEobTApDQpgYGANCmBgYHtyfQ0KbTEgPC0gbG1lcihmb3JtdWxhID0gQWNjX25fbSB+IFRhc2sqU3RpbSpFRl9tYWcgKyAoMSB8IElEKSwgZGF0YT1kYXRhX3N1YnNldCkNCnN1bW1hcnkobTEpDQphbm92YShtMSkNCmBgYA0KYGBge3J9DQpBSUMobTAsIG0xKQ0KQklDKG0wLCBtMSkNCmBgYA0KYGBge3J9DQptX2FjY19uX2VmX21hZyA8LSBsbShmb3JtdWxhID0gQWNjX25fbSB+IFRhc2sqU3RpbSpFRl9tYWcsIGRhdGE9ZGF0YV9zdWJzZXQpDQpzdW1tYXJ5KG1fYWNjX25fZWZfbWFnKQ0KYW5vdmEobV9hY2Nfbl9lZl9tYWcpDQpgYGANCmBgYHtyfQ0KZGF0YV9zdWJzZXQgPC0gc3Vic2V0KGRmX2VmX2VwZmwsIFN0aW0gIT0gJ1BsYWNlYm8nICYgVGFzayA9PSAnMmJhY2snKQ0KZGF0YV9zdWJzZXQgPC0gZHJvcGxldmVscyhkYXRhX3N1YnNldCkNCmxldmVscyhkYXRhX3N1YnNldCRTdGltKQ0KbGV2ZWxzKGRhdGFfc3Vic2V0JFRhc2spDQpgYGANCmBgYHtyfQ0KbV9hY2Nfbl9lZl9tYWdfMmJhY2sgPC0gbG0oZm9ybXVsYSA9IEFjY19uX20gfiBTdGltKkVGX21hZywgZGF0YT1kYXRhX3N1YnNldCkNCnN1bW1hcnkobV9hY2Nfbl9lZl9tYWdfMmJhY2spDQphbm92YShtX2FjY19uX2VmX21hZ18yYmFjaykNCmBgYA0KYGBge3J9DQpkYXRhX3N1YnNldCA8LSBzdWJzZXQoZGZfZWZfZXBmbCwgU3RpbSAhPSAnUGxhY2VibycgJiBUYXNrID09ICczYmFjaycpDQpkYXRhX3N1YnNldCA8LSBkcm9wbGV2ZWxzKGRhdGFfc3Vic2V0KQ0KbGV2ZWxzKGRhdGFfc3Vic2V0JFN0aW0pDQpsZXZlbHMoZGF0YV9zdWJzZXQkVGFzaykNCmBgYA0KYGBge3J9DQptX2FjY19uX2VmX21hZ18zYmFjayA8LSBsbShmb3JtdWxhID0gQWNjX25fbSB+IFN0aW0qRUZfbWFnLCBkYXRhPWRhdGFfc3Vic2V0KQ0Kc3VtbWFyeShtX2FjY19uX2VmX21hZ18zYmFjaykNCmFub3ZhKG1fYWNjX25fZWZfbWFnXzNiYWNrKQ0KYGBgDQojIyMgVGhlIHJlbGF0aW9uc2hpcCBiZXR3ZWVuIHRoZSBpbnRlbnNpdHkgb2YgdGhlIGFwcGxpZWQgZWxlY3RyaWMgZmllbGQgb24gdGhlIHRhcmdldCBhbmQgdGhlIHJlc3BvbnNlIHRpbWUgKGkuZS4sIHRoZSBzcGVlZCkNCmBgYHtyfQ0KZGF0YV9zdWJzZXQgPC0gc3Vic2V0KGRmX2VmX2VwZmwsIFN0aW0gIT0gJ1BsYWNlYm8nKQ0KZGF0YV9zdWJzZXQgPC0gZHJvcGxldmVscyhkYXRhX3N1YnNldCkNCmxldmVscyhkYXRhX3N1YnNldCRTdGltKQ0KYGBgDQpgYGB7cn0NCm0wIDwtIGxtKGZvcm11bGEgPSBTcGVlZF9tIH4gVGFzaypTdGltKkVGX2Zyb250KkVGX3BhciwgZGF0YT1kYXRhX3N1YnNldCkNCnN1bW1hcnkobTApDQphbm92YShtMCkNCmBgYA0KYGBge3J9DQptMSA8LSBsbWVyKGZvcm11bGEgPSBTcGVlZF9tIH4gVGFzaypTdGltKkVGX2Zyb250KkVGX3BhciArICgxIHwgSUQpLCBkYXRhPWRhdGFfc3Vic2V0KQ0Kc3VtbWFyeShtMSkNCmFub3ZhKG0xKQ0KYGBgDQpgYGB7cn0NCkFJQyhtMCwgbTEpDQpCSUMobTAsIG0xKQ0KYGBgDQpgYGB7cn0NCm1fc3BlZWRfZWYgPC0gbG1lcihmb3JtdWxhID0gU3BlZWRfbSB+IFRhc2sqU3RpbSpFRl9mcm9udCpFRl9wYXIgKyAoMSB8IElEKSwgZGF0YT1kYXRhX3N1YnNldCkNCnN1bW1hcnkobV9zcGVlZF9lZikNCmFub3ZhKG1fc3BlZWRfZWYpDQpgYGANCmBgYHtyfQ0KZGF0YV9zdWJzZXQgPC0gc3Vic2V0KGRmX2VmX2VwZmwsIFN0aW0gIT0gJ1BsYWNlYm8nICYgVGFzayA9PSAnMmJhY2snKQ0KZGF0YV9zdWJzZXQgPC0gZHJvcGxldmVscyhkYXRhX3N1YnNldCkNCmxldmVscyhkYXRhX3N1YnNldCRTdGltKQ0KbGV2ZWxzKGRhdGFfc3Vic2V0JFRhc2spDQpgYGANCmBgYHtyfQ0KbV9zcGVlZF9lZl8yYmFjayA8LSBsbWVyKGZvcm11bGEgPSBTcGVlZF9tIH4gU3RpbSpFRl9mcm9udCpFRl9wYXIgKyAoMSB8IElEKSwgZGF0YT1kYXRhX3N1YnNldCkNCnN1bW1hcnkobV9zcGVlZF9lZl8yYmFjaykNCmFub3ZhKG1fc3BlZWRfZWZfMmJhY2spDQpgYGANCmBgYHtyfQ0KZGF0YV9zdWJzZXQgPC0gc3Vic2V0KGRmX2VmX2VwZmwsIFN0aW0gIT0gJ1BsYWNlYm8nICYgVGFzayA9PSAnM2JhY2snKQ0KZGF0YV9zdWJzZXQgPC0gZHJvcGxldmVscyhkYXRhX3N1YnNldCkNCmxldmVscyhkYXRhX3N1YnNldCRTdGltKQ0KbGV2ZWxzKGRhdGFfc3Vic2V0JFRhc2spDQpgYGANCmBgYHtyfQ0KbV9zcGVlZF9lZl8zYmFjayA8LSBsbWVyKGZvcm11bGEgPSBTcGVlZF9tIH4gU3RpbSpFRl9mcm9udCpFRl9wYXIgKyAoMSB8IElEKSwgZGF0YT1kYXRhX3N1YnNldCkNCnN1bW1hcnkobV9zcGVlZF9lZl8zYmFjaykNCmFub3ZhKG1fc3BlZWRfZWZfM2JhY2spDQpgYGANCiMjIyMgTm90ZQ0KQXMgdGhlcmUgaXMgbm8gc2lnbmlmaWNhbnQgaW50ZXJhY3Rpb24gYW1vbmcgdGhlIGluZHVjZWQgZmllbGRzIGFuZCB0aGUgcmF0ZSBvZiBpbXByb3ZlbWVudCBpbiBzcGVlZCwgd2Ugd2lsbCBub3QgZG8gdGhlIGV4cGxvcmF0aW9ucyB3ZSBkaWQgZm9yIHRoZSBhY2N1cmFjeS4NCg0KIyMjIFRoZSByZWxhdGlvbnNoaXAgYmV0d2VlbiB0aGUgaW50ZW5zaXR5IG9mIHRoZSBhcHBsaWVkIGVsZWN0cmljIGZpZWxkIG9uIHRoZSB0YXJnZXQgYW5kIHRoZSBub3JtYWxpemVkIHNwZWVkDQpgYGB7cn0NCmRhdGFfc3Vic2V0IDwtIHN1YnNldChkZl9lZl9lcGZsLCBTdGltICE9ICdQbGFjZWJvJykNCmRhdGFfc3Vic2V0IDwtIGRyb3BsZXZlbHMoZGF0YV9zdWJzZXQpDQpsZXZlbHMoZGF0YV9zdWJzZXQkU3RpbSkNCmBgYA0KYGBge3J9DQptMCA8LSBsbShmb3JtdWxhID0gU3BlZWRfbl9tIH4gVGFzaypTdGltKkVGX2Zyb250KkVGX3BhciwgZGF0YT1kYXRhX3N1YnNldCkNCnN1bW1hcnkobTApDQphbm92YShtMCkNCmBgYA0KYGBge3J9DQptMSA8LSBsbWVyKGZvcm11bGEgPSBTcGVlZF9uX20gfiBUYXNrKlN0aW0qRUZfZnJvbnQqRUZfcGFyICsgKDEgfCBJRCksIGRhdGE9ZGF0YV9zdWJzZXQpDQpzdW1tYXJ5KG0xKQ0KYW5vdmEobTEpDQpgYGANCmBgYHtyfQ0KQUlDKG0wLCBtMSkNCkJJQyhtMCwgbTEpDQpgYGANCmBgYHtyfQ0KbV9zcGVlZF9uX2VmIDwtIGxtKGZvcm11bGEgPSBTcGVlZF9uX20gfiBUYXNrKlN0aW0qRUZfZnJvbnQqRUZfcGFyLCBkYXRhPWRhdGFfc3Vic2V0KQ0Kc3VtbWFyeShtX3NwZWVkX25fZWYpDQphbm92YShtX3NwZWVkX25fZWYpDQpgYGANCmBgYHtyfQ0KZGF0YV9zdWJzZXQgPC0gc3Vic2V0KGRmX2VmX2VwZmwsIFN0aW0gIT0gJ1BsYWNlYm8nICYgVGFzayA9PSAnMmJhY2snKQ0KZGF0YV9zdWJzZXQgPC0gZHJvcGxldmVscyhkYXRhX3N1YnNldCkNCmxldmVscyhkYXRhX3N1YnNldCRTdGltKQ0KbGV2ZWxzKGRhdGFfc3Vic2V0JFRhc2spDQpgYGANCmBgYHtyfQ0KbV9zcGVlZF9uX2VmXzJiYWNrIDwtIGxtKGZvcm11bGEgPSBTcGVlZF9uX20gfiBTdGltKkVGX2Zyb250KkVGX3BhciwgZGF0YT1kYXRhX3N1YnNldCkNCnN1bW1hcnkobV9zcGVlZF9uX2VmXzJiYWNrKQ0KYW5vdmEobV9zcGVlZF9uX2VmXzJiYWNrKQ0KYGBgDQpgYGB7cn0NCmRhdGFfc3Vic2V0IDwtIHN1YnNldChkZl9lZl9lcGZsLCBTdGltICE9ICdQbGFjZWJvJyAmIFRhc2sgPT0gJzNiYWNrJykNCmRhdGFfc3Vic2V0IDwtIGRyb3BsZXZlbHMoZGF0YV9zdWJzZXQpDQpsZXZlbHMoZGF0YV9zdWJzZXQkU3RpbSkNCmxldmVscyhkYXRhX3N1YnNldCRUYXNrKQ0KYGBgDQpgYGB7cn0NCm1fc3BlZWRfbl9lZl8zYmFjayA8LSBsbShmb3JtdWxhID0gU3BlZWRfbl9tIH4gU3RpbSpFRl9mcm9udCpFRl9wYXIsIGRhdGE9ZGF0YV9zdWJzZXQpDQpzdW1tYXJ5KG1fc3BlZWRfbl9lZl8zYmFjaykNCmFub3ZhKG1fc3BlZWRfbl9lZl8zYmFjaykNCmBgYA0KDQojIyBSZWxhdGlvbnNoaXAgYmV0d2VlbiBiZWhhdmlvciBhbmQgdGhlIHBoYXNlLWFtcGxpdHVkZSBjb3VwbGluZyBpbmRleCAoUEFDKQ0KDQpBcyBwYXJ0IG9mIG91ciBhbmFseXNpcywgd2UgZXh0cmFjdGVkIHRoZSBQQUMgYXQgYm90aCBzdGltdWxhdGlvbiBzaXRlcyBmb3IgZWFjaCBzdWJqZWN0LCBhcyB3YXMgZG9uZSBieSBSZWluaGFydCBhbmQgTmd1eWVuICgyMDE5KS4gSW4gdGhpcyBwYXBlciwgdGhlIGF1dGhvcnMgbG9va2VkIGF0IHRoZSBQQUMgYXQgdGhlICJtZW1vcnkgUk9JIiAobGVmdCBjZW50cm8tdGVtcG9yYWwgY2x1c3RlciksIGFuZCB0aGV5IGZvdW5kIGhpZ2gtcGVyZm9ybWluZyB5b3VuZyBhZHVsdHMgdG8gaGF2ZSBoaWdoIFBBQyB2YWx1ZXMgaW4gdGhpcyByZWdpb24uIEluIGNvbnRyYXN0LCBvbGRlciBhZHVsdHMgaGFkIGxvdyBQQUMgdmFsdWVzIGluIHRoYXQgcmVnaW9uLCBhcyB3ZWxsIGFzIGxvdyBwZXJmb3JtYW5jZSBpbiB0aGUgV00gdGFzay4gSG93ZXZlciwgd2hlbiBzdGltdWxhdGlvbiB3YXMgYXBwbGllZCwgcGVyZm9ybWFuY2UgaW1wcm92ZWQgc2hvcnRseSBhZnRlciBzdGFydGluZyB0aGUgc3RpbXVsYXRpb24sIGFuZCB0aGUgUEFDIHdhcyBhbHNvIGhpZ2ggaW4gb2xkZXIgYWR1bHRzLiBTbywgdGhlIG9iamVjdGl2ZSBvZiB0aGlzIGFuYWx5c2lzIGlzIHRvIHNlZSB3aGV0aGVyIHRoZXJlIGFyZSBtZWFuaW5nZnVsIGRpZmZlcmVuY2VzIGluIFBBQyBjb3JyZWxhdGluZyB3aXRoIHBlcmZvcm1hbmNlLiANCg0KVGhlIFBBQyBmZWF0dXJlcyB3ZSBhcmUgdXNpbmcgYXJlIGEgY29tcGFyaXNvbiBiZXR3ZWVuIHRoZSAyLWJhY2sgYW5kIHRoZSAzLWJhY2sgdGFza3MgKHN1YnRyYWN0aW9uKSwgYW5kIHRoZXkgYXJlIHRoZSBmb2xsb3dpbmc6DQotIE1lbW9yeSBST0kgKExpa2UgaW4gUmVpbmhhcnQgYW5kIE5ndXllbikNCi0gRnJvbnRhbCBzdGltdWxhdGlvbiBST0kNCi0gUGFyaWV0YWwgc3RpbXVsYXRpb24gUk9JDQoNClRoZSBjb21wYXJpc29uIGJldHdlZW4gdGhlIDItYmFjayBhbmQgdGhlIDMtYmFjayB0YXNrIHdhcyBkb25lIHRocm91Z2ggYSBzdWJ0cmFjdGlvbiBpbiB0aGUgUEFDIHZhbHVlcy4gVGhlIHJhdGlvbmFsZSBpcyB0aGF0IHRoaXMgZGlmZmVyZW5jZSBpc29sYXRlcyB0aGUgbmV1cmFsIGFjdGl2aXR5IHJlbGF0ZWQgdG8gcmV0YWluaW5nIGluZm9ybWF0aW9uIGluIHdvcmtpbmcgbWVtb3J5IGZvciBsb25nZXIsIHdoaWNoIGlzIGFzc3VtZWQgdG8gYmUgbW9yZSBkaWZmaWN1bHQgaW4gdGhlIDMtYmFjayBjb21wYXJlZCB0byB0aGUgMi1iYWNrIHRhc2sgYW5kIGlzIHN1cHBvcnRlZCBieSB0aGUgYmVoYXZpb3JhbCBkaWZmZXJlbmNlcyBzZWVuIGluIGJvdGggdGhlIHNwZWVkIGFuZCB0aGUgYWNjdXJhY3kgd2hlbiBjb21wYXJpbmcgcGVyZm9ybWFuY2UgaW4gYm90aCB0YXNrcy4gVGhlcmVmb3JlLCB0aGUgcmVzdWx0aW5nIGRlbHRhIGluIFBBQyBhbW9uZyBib3RoIHRhc2tzIHdvdWxkIGJlIHJlbGF0ZWQgdG8gYW4gaW5kaXZpZHVhbCdzIGFiaWxpdHkgdG8gcGVyZm9ybSB0aGUgKGhhcmRlcikgMy1iYWNrIHRhc2suDQoNCmBgYHtyfQ0KZGF0YV9kaXJlY3RvcnkgPC0gZmlsZS5wYXRoKHBhc3RlKGN1cnJfZGlyLCAnLi4vLi4vLi4vQ29kZS9Ob3RlYm9va3MvUmVzdWx0cycsIHNlcD0nLycpKQ0KDQpmaWxlX25hbWUgPC0gZmlsZS5wYXRoKHBhc3RlKGRhdGFfZGlyZWN0b3J5LCAiQmVoYXZpb3JhbF9hbmRfUEFDX2Jsb2Nrc19pQ09HX0Rlc2lnbl8yMl8wNl8yMDIzLnR4dCIsIHNlcD0nLycpKQ0KZGZfcGFjIDwtIHJlYWQuZGVsaW0oZmlsZV9uYW1lLCBoZWFkZXIgPSBUUlVFLCBuYS5zdHJpbmdzID0gIk5OIikNCmhlYWQoZGZfcGFjLCA1KQ0KYGBgDQpgYGB7cn0NCmRmX3BhYyRJRCA8LSBhcy5mYWN0b3IoZGZfcGFjJElEKQ0KZGZfcGFjJFNjaG9vbCA8LSBhcy5mYWN0b3IoZGZfcGFjJFNjaG9vbCkNCmRmX3BhYyREYXkgPC0gYXMuZmFjdG9yKGRmX3BhYyREYXkpDQpkZl9wYWMkVGFzayA8LSBhcy5mYWN0b3IoZGZfcGFjJFRhc2spDQpkZl9wYWMkU3RpbSA8LSBhcy5mYWN0b3IoZGZfcGFjJFN0aW0pDQpkZl9wYWMkQWNjX3UgPC0gYXMubnVtZXJpYyhkZl9wYWMkQWNjX3UpDQpkZl9wYWMkQWNjX25fdSA8LSBhcy5udW1lcmljKGRmX3BhYyRBY2Nfbl91KQ0KZGZfcGFjJFNwZWVkX3UgPC0gYXMubnVtZXJpYyhkZl9wYWMkU3BlZWRfdSkNCmRmX3BhYyRTcGVlZF9uX3UgPC0gYXMubnVtZXJpYyhkZl9wYWMkU3BlZWRfbl91KQ0KZGZfcGFjJEFjY19tIDwtIGFzLm51bWVyaWMoZGZfcGFjJEFjY19tKQ0KZGZfcGFjJEFjY19uX20gPC0gYXMubnVtZXJpYyhkZl9wYWMkQWNjX25fbSkNCmRmX3BhYyRTcGVlZF9tIDwtIGFzLm51bWVyaWMoZGZfcGFjJFNwZWVkX20pDQpkZl9wYWMkU3BlZWRfbl9tIDwtIGFzLm51bWVyaWMoZGZfcGFjJFNwZWVkX25fbSkNCmRmX3BhYyRQQUNfbWVtIDwtIGFzLm51bWVyaWMoZGZfcGFjJFBBQ19tZW0pDQpkZl9wYWMkUEFDX2Zyb250IDwtIGFzLm51bWVyaWMoZGZfcGFjJFBBQ19mcm9udCkNCmRmX3BhYyRQQUNfcGFyIDwtIGFzLm51bWVyaWMoZGZfcGFjJFBBQ19wYXIpDQpgYGANCg0KYGBge3J9DQpkZl9wYWNfZXBmbCA8LSBzdWJzZXQoZGZfcGFjLCBTY2hvb2wgPT0gJ0VQRkwnKQ0KZGZfcGFjX2VwZmwgPC0gZHJvcGxldmVscyhkZl9wYWNfZXBmbCkNCmRmX3BhY19lcGZsJFN0aW0gPC0gZmFjdG9yKGRmX3BhY19lcGZsJFN0aW0sIGxldmVscyhkZl9wYWNfZXBmbCRTdGltKVtjKDEsIDMsIDIsIDYsIDQsIDUpXSkNCmxldmVscyhkZl9wYWNfZXBmbCRTdGltKQ0KYGBgDQoNCg0KIyMjIFJlbGF0aW9uc2hpcCBiZXR3ZWVuIFBBQyBhbmQgcGVyZm9ybWFuY2UNCkJhc2VkIG9uIFJlaW5oYXJ0J3MgYW5kIE5ndXllbidzIGZpbmRpbmdzLCBwZW9wbGUgd2l0aCBoaWdoZXIgUEFDIHZhbHVlcyBhdCB0aGUgIk1lbW9yeSBST0kiIHdvdWxkIGJlIGV4cGVjdGVkIHRvIGhhdmUgYSBiZXR0ZXIgcGVyZm9ybWFuY2UgaW4gdGhlIHRhc2suIFRoZXJlZm9yZSwgd2Ugd2lsbCBmaXJzdCB0ZXN0IHRoZSByZWxhdGlvbnNoaXAgYmV0d2VlbiB0aGUgUEFDIGFuZCB0aGUgcGVyZm9ybWFuY2UgYXQgYmFzZWxpbmUuIEFzIHRoZSBQQUMgdmFsdWVzIHdlIGFyZSB1c2luZyBjb25zaXN0IG9uIHRoZSBkaWZmZXJlbmNlIG9mIHZhbHVlcyBiZXR3ZWVuIHRoZSAyYmFjayBhbmQgM2JhY2sgY29uZGl0aW9ucywgd2hpY2ggaXMgbWVhbnQgdG8gcmVmbGVjdCB0aGUgY291cGxpbmcgcmVsYXRlZCB0byBoaWdoZXIgd29ya2luZyBtZW1vcnkgZGVtYW5kcywgd2Ugd2lsbCB1c2UgdGhlIGJlaGF2aW9yYWwgc2NvcmVzIG9mIHRoZSAzYmFjayB0YXNrLg0KIyMjIyBBY2N1cmFjeSBhbmQgbWVtb3J5IFJPSQ0KYGBge3J9DQpkYXRhX3N1YnNldCA8LSBzdWJzZXQoZGZfcGFjX2VwZmwsIFN0aW0gPT0gIkJhc2VsaW5lIiAmIFRhc2sgPT0gIjNiYWNrIikNCmRhdGFfc3Vic2V0IDwtIGRyb3BsZXZlbHMoZGF0YV9zdWJzZXQpDQpsZXZlbHMoZGF0YV9zdWJzZXQkU3RpbSkNCmxldmVscyhkYXRhX3N1YnNldCRUYXNrKQ0KYGBgDQojIyMjIEF2ZXJhZ2UgYWNjdXJhY3kNCmBgYHtyfQ0KbV9wYWNfbWVtX2FjY191X0JMIDwtIGxtKGZvcm11bGEgPSBBY2NfdSB+IFBBQ19tZW0sIGRhdGE9ZGF0YV9zdWJzZXQpDQpzdW1tYXJ5KG1fcGFjX21lbV9hY2NfdV9CTCkNCmFub3ZhKG1fcGFjX21lbV9hY2NfdV9CTCkNCmV0YV9zcXVhcmVkKG1fcGFjX21lbV9hY2NfdV9CTCkNCmBgYA0KIyMjIyBOb3RlIA0KVGhlcmUgaXMgYSBzaWduaWZpY2FudCBlZmZlY3Qgb2YgdGhlIFBBQyB2YWx1ZXMgZXN0aW1hdGVkIGF0IHRoZSAibWVtb3J5IiB0YXJnZXQgb24gdGhlIG1lYW4gYWNjdXJhY3kgcGFydGljaXBhbnRzIGhhZCBhdCBiYXNlbGluZS4gQmFzZWQgb24gdGhlIGVzdGltYXRlZCBzbG9wZSwgd2hpY2ggaXMgbmVnYXRpdmUsIGl0IHdvdWxkIGFwcGVhciB0aGUgZGlyZWN0aW9uIG9mIHRoZSBlZmZlY3QgaXMgdGhlIG9wcG9zaXRlIHRvIHRoZSBvbmUgcmVwb3J0ZWQgYnkgUmVpbmhhcnQgYW5kIE5ndXllbiwgd2hvIHJlcG9ydGVkIHBlb3BsZSB3aXRoIGhpZ2hlciBQQUMgdmFsdWVzIGFsc28gcG9zc2VzZWQgaGlnaGVyIGFjY3VyYWN5IGF0IGJhc2VsaW5lLiBIb3dldmVyLCB3ZSBhcmUgdXNpbmcgdGhlIGRpZmZlcmVuY2UgaW4gUEFDIGFtb25nIGNvbmRpdGlvbnMgc28gdGhlIGludGVycHJldGF0aW9uIGlzIGxlc3MgZGlyZWN0LiANCg0KIyMjIyBBY2N1cmFjeSBhbmQgUEFDIGF0IDEwLzIwIEVFRyBlbGVjdHJvZGUtYmFzZWQgdGFyZ2V0cw0KYGBge3J9DQptX3BhY190YXJnZXRzX2FjY191X0JMIDwtIGxtKGZvcm11bGEgPSBBY2NfdSB+IFBBQ19mcm9udCpQQUNfcGFyLCBkYXRhPWRhdGFfc3Vic2V0KQ0Kc3VtbWFyeShtX3BhY190YXJnZXRzX2FjY191X0JMKQ0KYW5vdmEobV9wYWNfdGFyZ2V0c19hY2NfdV9CTCkNCmV0YV9zcXVhcmVkKG1fcGFjX3RhcmdldHNfYWNjX3VfQkwpDQpgYGANCiMjIyMgRXhwbG9yZSB0aGUgaW50ZXJhY3Rpb24gYmV0d2VlbiB0aGUgYmktZm9jYWwgUEFDIGFuZCB0aGUgYWNjdXJhY3kNCmBgYHtyfQ0Kbl9yb3dzID0gMTAwDQpjaG9vc2VfbW9kZWwgPC0gbV9wYWNfdGFyZ2V0c19hY2NfdV9CTA0Kc3RpbV9saXN0IDwtIGxldmVscyhkYXRhX3N1YnNldCRTdGltKQ0KaV9zdGFydCA9IG1pbihtaW4oZGF0YV9zdWJzZXQkUEFDX2Zyb250LCBuYS5ybT1UUlVFKSwgbWluKGRhdGFfc3Vic2V0JFBBQ19wYXIsIG5hLnJtPVRSVUUpKQ0KaV9zdG9wID0gbWF4KG1heChkYXRhX3N1YnNldCRQQUNfZnJvbnQsIG5hLnJtPVRSVUUpLCBtYXgoZGF0YV9zdWJzZXQkUEFDX3BhciwgbmEucm09VFJVRSkpDQoNCmNvbHVtbnMgPSBjKCJTdGltIiwiUEFDX2Zyb250IiwgIlBBQ19wYXIiKSANCmRmX3RlbXAgPSBkYXRhLmZyYW1lKG1hdHJpeChucm93ID0gbl9yb3dzLCBuY29sID0gbGVuZ3RoKGNvbHVtbnMpKSkgDQpjb2xuYW1lcyhkZl90ZW1wKSA9IGNvbHVtbnMNCg0KZm9yIChzIGluIHN0aW1fbGlzdCl7DQogICAgDQogIGRmX3RlbXAkU3RpbSA8LSByZXAocywgbl9yb3dzKQ0KICBkZl90ZW1wJFBBQ19mcm9udCA8LSByZXAoc2VxKGlfc3RhcnQsIGlfc3RvcCwgbGVuZ3RoLm91dCA9IDEwKSwgMTApDQogIGRmX3RlbXAkUEFDX3BhciA8LSByZXAoc2VxKGlfc3RhcnQsIGlfc3RvcCwgbGVuZ3RoLm91dCA9IDEwKSwgZWFjaD0xMCkNCn0NCmBgYA0KYGBge3J9DQpwcmVkcyA8LSBwcmVkaWN0KGNob29zZV9tb2RlbCwgZGZfdGVtcCkNCm1pbl96IDwtIG1pbihwcmVkcykNCm1heF96ID0gbWF4KHByZWRzKQ0KZGZfdGVtcCRQcmVkIDwtIHByZWRzDQpgYGANCmBgYHtyfQ0KIyBWaWV3aW5nIGFuZ2xlcyBmb3IgdGhlIHBsb3RzDQphbmdsZV9jb2x1bW5zID0gYygiU3RpbSIsIkF6aW0iLCJDb2xhdGl0dWRlIikgDQpkZl9hbmdsZXMgPSBkYXRhLmZyYW1lKG1hdHJpeChucm93ID0gNCwgbmNvbCA9IGxlbmd0aChhbmdsZV9jb2x1bW5zKSkpIA0KY29sbmFtZXMoZGZfYW5nbGVzKSA9IGFuZ2xlX2NvbHVtbnMNCg0KZGZfYW5nbGVzJFN0aW0gPC0gc3RpbV9saXN0DQpkZl9hbmdsZXMkQXppbSA8LSBjKDAsIDAsIDAsIDApICMgVGhldGEsIGRlZmF1bHQgMA0KZGZfYW5nbGVzJENvbGF0aXR1ZGUgPC0gYygxNSwgMTUsIDE1LCAxNSkgIyBQaGksIGRlZmF1bHQgMTUNCmBgYA0KYGBge3J9DQojIE1ha2UgcGxvdHMNCmZvciAocyBpbiBzdGltX2xpc3Qpew0KICANCiAgZGlzcF9kZiA8LSBzdWJzZXQoZGZfdGVtcCwgU3RpbSA9PSBzKQ0KICBwZXJzcCh4PXNlcShpX3N0YXJ0LCBpX3N0b3AsIGxlbmd0aC5vdXQgPSAxMCksIHk9c2VxKGlfc3RhcnQsIGlfc3RvcCwgbGVuZ3RoLm91dCA9IDEwKSwgej1tYXRyaXgoZGlzcF9kZiRQcmVkLCBucm93PTEwLCBuY29sPTEwLGJ5cm93PVRSVUUpLCANCiAgICAgICAgdGhldGEgPSBzdWJzZXQoZGZfYW5nbGVzLCBTdGltID09IHMpJEF6aW0sIHBoaSA9IHN1YnNldChkZl9hbmdsZXMsIFN0aW0gPT0gcykkQ29sYXRpdHVkZSwNCiAgICAgICAgeGxhYj0iUEFDX2Zyb250Iix5bGFiPSJQQUNfcGFyIix6bGFiPSJNZWFuIGFjY3VyYWN5IiwgbWFpbj1wYXN0ZShzLCIzYmFjayIpLCB6bGltPWMoMiptaW5feiwyKm1heF96KSkgDQp9DQpgYGANCmBgYHtyfQ0KIyBFeHBvcnQgdGhlIGRhdGEgZm9yIHBsb3R0aW5nDQpzYXZlX2ZpbGVfbmFtZSA8LSBmaWxlLnBhdGgocGFzdGUoZGF0YV9kaXJlY3RvcnksICJFUEZMX1BBQ19CTEFjY19pbnRlcmFjdGlvbnMuY3N2Iiwgc2VwPScvJykpDQp3cml0ZS5jc3YoZGZfdGVtcCwgc2F2ZV9maWxlX25hbWUpDQpgYGANCg0KDQoNCmBgYHtyfQ0KZGF0YV9zdWJzZXQgPC0gc3Vic2V0KGRmX3BhY19lcGZsLCBTdGltICE9ICJCYXNlbGluZSIgJiBTdGltICE9ICJQbGFjZWJvIiAmIFRhc2sgPT0gIjNiYWNrIikNCmRhdGFfc3Vic2V0IDwtIGRyb3BsZXZlbHMoZGF0YV9zdWJzZXQpDQpsZXZlbHMoZGF0YV9zdWJzZXQkU3RpbSkNCmxldmVscyhkYXRhX3N1YnNldCRUYXNrKQ0KYGBgDQpgYGB7cn0NCm1fcGFjX21lbV9hY2NfdV9zdGltIDwtIGxtKGZvcm11bGEgPSBBY2NfdSB+IFN0aW0qUEFDX21lbSwgZGF0YT1kYXRhX3N1YnNldCkNCnN1bW1hcnkobV9wYWNfbWVtX2FjY191X3N0aW0pDQphbm92YShtX3BhY19tZW1fYWNjX3Vfc3RpbSkNCmBgYA0KYGBge3J9DQptX3BhY190YXJnZXRzX2FjY191X3N0aW1zIDwtIGxtKGZvcm11bGEgPSBBY2NfdSB+IFN0aW0qUEFDX2Zyb250KlBBQ19wYXIsIGRhdGE9ZGF0YV9zdWJzZXQpDQpzdW1tYXJ5KG1fcGFjX3RhcmdldHNfYWNjX3Vfc3RpbXMpDQphbm92YShtX3BhY190YXJnZXRzX2FjY191X3N0aW1zKQ0KZXRhX3NxdWFyZWQobV9wYWNfdGFyZ2V0c19hY2NfdV9zdGltcykNCmBgYA0KIyMjIyBOb3RlDQpUaGVyZSBpcyBubyBkaWZmZXJlbmNlIGFtb25nIHRoZSBzdGltdWxhdGlvbiBncm91cHMuVGhlIG5leHQgcGxvdHMgc2hvdyB0aGUgcmVsYXRpb25zaGlwIGJldHdlZW4gUEFDIGFuZCBhY2N1cmFjeSBpbiBlYWNoIGdyb3VwIHRvIHZpc3VhbGl6ZSB0aGUgbW9kZWwgZXN0aW1hdGVzLg0KDQpgYGB7cn0NCm5fcm93cyA9IDEwMA0KY2hvb3NlX21vZGVsIDwtIG1fcGFjX3RhcmdldHNfYWNjX3Vfc3RpbXMNCnN0aW1fbGlzdCA8LSBsZXZlbHMoZGF0YV9zdWJzZXQkU3RpbSkNCmlfc3RhcnQgPSBtaW4obWluKGRhdGFfc3Vic2V0JFBBQ19mcm9udCwgbmEucm09VFJVRSksIG1pbihkYXRhX3N1YnNldCRQQUNfcGFyLCBuYS5ybT1UUlVFKSkNCmlfc3RvcCA9IG1heChtYXgoZGF0YV9zdWJzZXQkUEFDX2Zyb250LCBuYS5ybT1UUlVFKSwgbWF4KGRhdGFfc3Vic2V0JFBBQ19wYXIsIG5hLnJtPVRSVUUpKQ0KDQpjb2x1bW5zID0gYygiU3RpbSIsIlBBQ19mcm9udCIsICJQQUNfcGFyIikgDQpkZl90ZXN0ID0gZGF0YS5mcmFtZShtYXRyaXgobnJvdyA9IG5fcm93cywgbmNvbCA9IGxlbmd0aChjb2x1bW5zKSkpIA0KY29sbmFtZXMoZGZfdGVzdCkgPSBjb2x1bW5zDQoNCmNvbHVtbnMgPSBjKCJTdGltIiwiUEFDX2Zyb250IiwgIlBBQ19wYXIiKSANCmRmX3RlbXAgPSBkYXRhLmZyYW1lKG1hdHJpeChucm93ID0gbl9yb3dzLCBuY29sID0gbGVuZ3RoKGNvbHVtbnMpKSkgDQpjb2xuYW1lcyhkZl90ZW1wKSA9IGNvbHVtbnMNCg0KZm9yIChzIGluIHN0aW1fbGlzdCl7DQogIGlmIChzID09IHN0aW1fbGlzdFsxXSl7DQogIGRmX3Rlc3QkU3RpbSA8LSByZXAocywgbl9yb3dzKQ0KICBkZl90ZXN0JFBBQ19mcm9udCA8LSByZXAoc2VxKGlfc3RhcnQsIGlfc3RvcCwgbGVuZ3RoLm91dCA9IDEwKSwgMTApDQogIGRmX3Rlc3QkUEFDX3BhciA8LSByZXAoc2VxKGlfc3RhcnQsIGlfc3RvcCwgbGVuZ3RoLm91dCA9IDEwKSwgZWFjaD0xMCkNCiAgI2RmX3Rlc3QkVGFzayA8LSBzYW1wbGUoYygnMmJhY2snLCAnM2JhY2snKSwgMTAwLCByZXBsYWNlPVRSVUUpDQogIH0NCiAgZWxzZXsNCiAgZGZfdGVtcCRTdGltIDwtIHJlcChzLCBuX3Jvd3MpDQogIGRmX3RlbXAkUEFDX2Zyb250IDwtIHJlcChzZXEoaV9zdGFydCwgaV9zdG9wLCBsZW5ndGgub3V0ID0gMTApLCAxMCkNCiAgZGZfdGVtcCRQQUNfcGFyIDwtIHJlcChzZXEoaV9zdGFydCwgaV9zdG9wLCBsZW5ndGgub3V0ID0gMTApLCBlYWNoPTEwKQ0KICAjZGZfdGVtcCRUYXNrIDwtIHNhbXBsZShjKCcyYmFjaycsICczYmFjaycpLCAxMDAsIHJlcGxhY2U9VFJVRSkNCiAgZGZfdGVzdCA8LSByYmluZChkZl90ZXN0LCBkZl90ZW1wKQ0KICB9DQogIA0KfQ0KYGBgDQpgYGB7cn0NCnByZWRzIDwtIHByZWRpY3QoY2hvb3NlX21vZGVsLCBkZl90ZXN0KQ0KbWluX3ogPC0gbWluKHByZWRzKQ0KbWF4X3ogPSBtYXgocHJlZHMpDQpkZl90ZXN0JFByZWQgPC0gcHJlZHMNCmBgYA0KYGBge3J9DQojIFZpZXdpbmcgYW5nbGVzIGZvciB0aGUgcGxvdHMNCmFuZ2xlX2NvbHVtbnMgPSBjKCJTdGltIiwiQXppbSIsIkNvbGF0aXR1ZGUiKSANCmRmX2FuZ2xlcyA9IGRhdGEuZnJhbWUobWF0cml4KG5yb3cgPSA0LCBuY29sID0gbGVuZ3RoKGFuZ2xlX2NvbHVtbnMpKSkgDQpjb2xuYW1lcyhkZl9hbmdsZXMpID0gYW5nbGVfY29sdW1ucw0KDQpkZl9hbmdsZXMkU3RpbSA8LSBzdGltX2xpc3QNCmRmX2FuZ2xlcyRBemltIDwtIGMoMCwgMCwgMCwgMCkgIyBUaGV0YSwgZGVmYXVsdCAwDQpkZl9hbmdsZXMkQ29sYXRpdHVkZSA8LSBjKDE1LCAxNSwgMTUsIDE1KSAjIFBoaSwgZGVmYXVsdCAxNQ0KYGBgDQpgYGB7cn0NCiMgTWFrZSBwbG90cw0KZm9yIChzIGluIHN0aW1fbGlzdCl7DQogIGRpc3BfZGYgPC0gc3Vic2V0KGRmX3Rlc3QsIFN0aW0gPT0gcykNCiAgcGVyc3AoeD1zZXEoaV9zdGFydCwgaV9zdG9wLCBsZW5ndGgub3V0ID0gMTApLCB5PXNlcShpX3N0YXJ0LCBpX3N0b3AsIGxlbmd0aC5vdXQgPSAxMCksIHo9bWF0cml4KGRpc3BfZGYkUHJlZCwgbnJvdz0xMCwgbmNvbD0xMCxieXJvdz1UUlVFKSwgDQogICAgICAgIHRoZXRhID0gc3Vic2V0KGRmX2FuZ2xlcywgU3RpbSA9PSBzKSRBemltLCBwaGkgPSBzdWJzZXQoZGZfYW5nbGVzLCBTdGltID09IHMpJENvbGF0aXR1ZGUsDQogICAgICAgIHhsYWI9IlBBQ19mcm9udCIseWxhYj0iUEFDX3BhciIsemxhYj0iTWVhbiBhY2N1cmFjeSIsIG1haW49cGFzdGUocywiM2JhY2siKSwgemxpbT1jKG1pbl96LG1heF96KSkgDQp9DQpgYGANCiMjIyMgTm9ybWFsaXplZCBhY2N1cmFjeQ0KYGBge3J9DQpkYXRhX3N1YnNldCA8LSBzdWJzZXQoZGZfcGFjX2VwZmwsIFN0aW0gPT0gIlBsYWNlYm8iICYgVGFzayA9PSAiM2JhY2siKQ0KZGF0YV9zdWJzZXQgPC0gZHJvcGxldmVscyhkYXRhX3N1YnNldCkNCmxldmVscyhkYXRhX3N1YnNldCRTdGltKQ0KbGV2ZWxzKGRhdGFfc3Vic2V0JFRhc2spDQpgYGANCmBgYHtyfQ0KbV9wYWNfbWVtX2FjY19uX3VfcGxhY2VibyA8LSBsbShmb3JtdWxhID0gQWNjX25fdSB+IFBBQ19tZW0sIGRhdGE9ZGF0YV9zdWJzZXQpDQpzdW1tYXJ5KG1fcGFjX21lbV9hY2Nfbl91X3BsYWNlYm8pDQphbm92YShtX3BhY19tZW1fYWNjX25fdV9wbGFjZWJvKQ0KYGBgDQpgYGB7cn0NCm1fcGFjX3RhcmdldHNfYWNjX25fdV9wbGFjZWJvIDwtIGxtKGZvcm11bGEgPSBBY2Nfbl91IH4gUEFDX2Zyb250KlBBQ19wYXIsIGRhdGE9ZGF0YV9zdWJzZXQpDQpzdW1tYXJ5KG1fcGFjX3RhcmdldHNfYWNjX25fdV9wbGFjZWJvKQ0KYW5vdmEobV9wYWNfdGFyZ2V0c19hY2Nfbl91X3BsYWNlYm8pDQpgYGANCiMjIyMgU3RpbXVsYXRpb25zDQpgYGB7cn0NCmRhdGFfc3Vic2V0IDwtIHN1YnNldChkZl9wYWNfZXBmbCwgU3RpbSAhPSAiUGxhY2VibyIgJiBUYXNrID09ICIzYmFjayIpDQpkYXRhX3N1YnNldCA8LSBkcm9wbGV2ZWxzKGRhdGFfc3Vic2V0KQ0KbGV2ZWxzKGRhdGFfc3Vic2V0JFN0aW0pDQpsZXZlbHMoZGF0YV9zdWJzZXQkVGFzaykNCmBgYA0KYGBge3J9DQptX3BhY19tZW1fYWNjX25fdV9zdGltIDwtIGxtKGZvcm11bGEgPSBBY2Nfbl91IH4gU3RpbSpQQUNfbWVtLCBkYXRhPWRhdGFfc3Vic2V0KQ0Kc3VtbWFyeShtX3BhY19tZW1fYWNjX25fdV9zdGltKQ0KYW5vdmEobV9wYWNfbWVtX2FjY19uX3Vfc3RpbSkNCmBgYA0KYGBge3J9DQptX3BhY190YXJnZXRzX2FjY19uX3Vfc3RpbSA8LSBsbShmb3JtdWxhID0gQWNjX25fdSB+IFN0aW0qUEFDX2Zyb250KlBBQ19wYXIsIGRhdGE9ZGF0YV9zdWJzZXQpDQpzdW1tYXJ5KG1fcGFjX3RhcmdldHNfYWNjX25fdV9zdGltKQ0KYW5vdmEobV9wYWNfdGFyZ2V0c19hY2Nfbl91X3N0aW0pDQpgYGANCg0KIyMjIFBBQyBhbmQgc3BlZWQNCiMjIyMgQmFzZWxpbmUNCmBgYHtyfQ0KZGF0YV9zdWJzZXQgPC0gc3Vic2V0KGRmX3BhY19lcGZsLCBTdGltID09ICJCYXNlbGluZSIgJiBUYXNrID09ICIzYmFjayIpDQpkYXRhX3N1YnNldCA8LSBkcm9wbGV2ZWxzKGRhdGFfc3Vic2V0KQ0KbGV2ZWxzKGRhdGFfc3Vic2V0JFN0aW0pDQpsZXZlbHMoZGF0YV9zdWJzZXQkVGFzaykNCmBgYA0KIyMjIyBNZW1vcnkgUk9JDQpgYGB7cn0NCm1fcGFjX21lbV9zcGVlZF91X0JMIDwtIGxtKGZvcm11bGEgPSBTcGVlZF91IH4gUEFDX21lbSwgZGF0YT1kYXRhX3N1YnNldCkNCnN1bW1hcnkobV9wYWNfbWVtX3NwZWVkX3VfQkwpDQphbm92YShtX3BhY19tZW1fc3BlZWRfdV9CTCkNCmBgYA0KIyMjIyBGcm9udGFsIGFuZCBwYXJpZXRhbCB0YXJnZXRzDQpgYGB7cn0NCm1fcGFjX3RhcmdldHNfc3BlZWRfdV9CTCA8LSBsbShmb3JtdWxhID0gU3BlZWRfdSB+IFBBQ19mcm9udCpQQUNfcGFyLCBkYXRhPWRhdGFfc3Vic2V0KQ0Kc3VtbWFyeShtX3BhY190YXJnZXRzX3NwZWVkX3VfQkwpDQphbm92YShtX3BhY190YXJnZXRzX3NwZWVkX3VfQkwpDQpldGFfc3F1YXJlZChtX3BhY190YXJnZXRzX3NwZWVkX3VfQkwpDQpgYGANCiMjIyMgU3RpbXVsYXRpb24gZ3JvdXBzDQpgYGB7cn0NCmRhdGFfc3Vic2V0IDwtIHN1YnNldChkZl9wYWNfZXBmbCwgU3RpbSAhPSAiQmFzZWxpbmUiICYgU3RpbSAhPSAiUGxhY2VibyIgJiBUYXNrID09ICIzYmFjayIpDQpkYXRhX3N1YnNldCA8LSBkcm9wbGV2ZWxzKGRhdGFfc3Vic2V0KQ0KbGV2ZWxzKGRhdGFfc3Vic2V0JFN0aW0pDQpsZXZlbHMoZGF0YV9zdWJzZXQkVGFzaykNCmBgYA0KIyMjIyBNZW1vcnkgUk9JDQpgYGB7cn0NCm1fcGFjX21lbV9zcGVlZF91X3N0aW0gPC0gbG0oZm9ybXVsYSA9IFNwZWVkX3UgfiBTdGltKlBBQ19tZW0sIGRhdGE9ZGF0YV9zdWJzZXQpDQpzdW1tYXJ5KG1fcGFjX21lbV9zcGVlZF91X3N0aW0pDQphbm92YShtX3BhY19tZW1fc3BlZWRfdV9zdGltKQ0KYGBgDQojIyMjIEZyb250YWwgYW5kIHBhcmlldGFsIHRhcmdldHMNCmBgYHtyfQ0KbV9wYWNfdGFyZ2V0c19zcGVlZF91X3N0aW0gPC0gbG0oZm9ybXVsYSA9IFNwZWVkX3UgfiBTdGltKlBBQ19mcm9udCpQQUNfcGFyLCBkYXRhPWRhdGFfc3Vic2V0KQ0Kc3VtbWFyeShtX3BhY190YXJnZXRzX3NwZWVkX3Vfc3RpbSkNCmFub3ZhKG1fcGFjX3RhcmdldHNfc3BlZWRfdV9zdGltKQ0KYGBgDQojIyMjIE5vcm1hbGl6ZWQgc3BlZWQNCmBgYHtyfQ0KZGF0YV9zdWJzZXQgPC0gc3Vic2V0KGRmX3BhY19lcGZsLCBTdGltID09ICJQbGFjZWJvIiAmIFRhc2sgPT0gIjNiYWNrIikNCmRhdGFfc3Vic2V0IDwtIGRyb3BsZXZlbHMoZGF0YV9zdWJzZXQpDQpsZXZlbHMoZGF0YV9zdWJzZXQkU3RpbSkNCmxldmVscyhkYXRhX3N1YnNldCRUYXNrKQ0KYGBgDQpgYGB7cn0NCm1fcGFjX21lbV9zcGVlZF9uX3VfcGxhY2VibyA8LSBsbShmb3JtdWxhID0gU3BlZWRfbl91IH4gUEFDX21lbSwgZGF0YT1kYXRhX3N1YnNldCkNCnN1bW1hcnkobV9wYWNfbWVtX3NwZWVkX25fdV9wbGFjZWJvKQ0KYW5vdmEobV9wYWNfbWVtX3NwZWVkX25fdV9wbGFjZWJvKQ0KYGBgDQpgYGB7cn0NCm1fcGFjX3RhcmdldHNfc3BlZWRfbl91X3BsYWNlYm8gPC0gbG0oZm9ybXVsYSA9IFNwZWVkX25fdSB+IFBBQ19mcm9udCpQQUNfcGFyLCBkYXRhPWRhdGFfc3Vic2V0KQ0Kc3VtbWFyeShtX3BhY190YXJnZXRzX3NwZWVkX25fdV9wbGFjZWJvKQ0KYW5vdmEobV9wYWNfdGFyZ2V0c19zcGVlZF9uX3VfcGxhY2VibykNCmBgYA0KIyMjIyBTdGltdWxhdGlvbnMNCmBgYHtyfQ0KZGF0YV9zdWJzZXQgPC0gc3Vic2V0KGRmX3BhY19lcGZsLCBTdGltICE9ICJQbGFjZWJvIiAmIFN0aW0gIT0gJ0Jhc2VsaW5lJyAmIFRhc2sgPT0gIjNiYWNrIikNCmRhdGFfc3Vic2V0IDwtIGRyb3BsZXZlbHMoZGF0YV9zdWJzZXQpDQpsZXZlbHMoZGF0YV9zdWJzZXQkU3RpbSkNCmxldmVscyhkYXRhX3N1YnNldCRUYXNrKQ0KYGBgDQpgYGB7cn0NCm1fcGFjX21lbV9zcGVlZF9uX3Vfc3RpbSA8LSBsbShmb3JtdWxhID0gU3BlZWRfbl91IH4gU3RpbSpQQUNfbWVtLCBkYXRhPWRhdGFfc3Vic2V0KQ0Kc3VtbWFyeShtX3BhY19tZW1fc3BlZWRfbl91X3N0aW0pDQphbm92YShtX3BhY19tZW1fc3BlZWRfbl91X3N0aW0pDQpgYGANCmBgYHtyfQ0KbV9wYWNfdGFyZ2V0c19zcGVlZF9uX3Vfc3RpbSA8LSBsbShmb3JtdWxhID0gU3BlZWRfbl91IH4gU3RpbSpQQUNfZnJvbnQqUEFDX3BhciwgZGF0YT1kYXRhX3N1YnNldCkNCnN1bW1hcnkobV9wYWNfdGFyZ2V0c19zcGVlZF9uX3Vfc3RpbSkNCmFub3ZhKG1fcGFjX3RhcmdldHNfc3BlZWRfbl91X3N0aW0pDQpgYGANCmBgYHtyfQ0Kbl9yb3dzID0gMTAwDQpjaG9vc2VfbW9kZWwgPC0gbV9wYWNfdGFyZ2V0c19zcGVlZF9uX3Vfc3RpbQ0Kc3RpbV9saXN0IDwtIGxldmVscyhkYXRhX3N1YnNldCRTdGltKQ0KaV9zdGFydCA9IG1pbihtaW4oZGF0YV9zdWJzZXQkUEFDX2Zyb250LCBuYS5ybT1UUlVFKSwgbWluKGRhdGFfc3Vic2V0JFBBQ19wYXIsIG5hLnJtPVRSVUUpKQ0KaV9zdG9wID0gbWF4KG1heChkYXRhX3N1YnNldCRQQUNfZnJvbnQsIG5hLnJtPVRSVUUpLCBtYXgoZGF0YV9zdWJzZXQkUEFDX3BhciwgbmEucm09VFJVRSkpDQoNCmNvbHVtbnMgPSBjKCJTdGltIiwiUEFDX2Zyb250IiwgIlBBQ19wYXIiKSANCmRmX3Rlc3QgPSBkYXRhLmZyYW1lKG1hdHJpeChucm93ID0gbl9yb3dzLCBuY29sID0gbGVuZ3RoKGNvbHVtbnMpKSkgDQpjb2xuYW1lcyhkZl90ZXN0KSA9IGNvbHVtbnMNCg0KY29sdW1ucyA9IGMoIlN0aW0iLCJQQUNfZnJvbnQiLCAiUEFDX3BhciIpIA0KZGZfdGVtcCA9IGRhdGEuZnJhbWUobWF0cml4KG5yb3cgPSBuX3Jvd3MsIG5jb2wgPSBsZW5ndGgoY29sdW1ucykpKSANCmNvbG5hbWVzKGRmX3RlbXApID0gY29sdW1ucw0KDQpmb3IgKHMgaW4gc3RpbV9saXN0KXsNCiAgaWYgKHMgPT0gc3RpbV9saXN0WzFdKXsNCiAgZGZfdGVzdCRTdGltIDwtIHJlcChzLCBuX3Jvd3MpDQogIGRmX3Rlc3QkUEFDX2Zyb250IDwtIHJlcChzZXEoaV9zdGFydCwgaV9zdG9wLCBsZW5ndGgub3V0ID0gMTApLCAxMCkNCiAgZGZfdGVzdCRQQUNfcGFyIDwtIHJlcChzZXEoaV9zdGFydCwgaV9zdG9wLCBsZW5ndGgub3V0ID0gMTApLCBlYWNoPTEwKQ0KICAjZGZfdGVzdCRUYXNrIDwtIHNhbXBsZShjKCcyYmFjaycsICczYmFjaycpLCAxMDAsIHJlcGxhY2U9VFJVRSkNCiAgfQ0KICBlbHNlew0KICBkZl90ZW1wJFN0aW0gPC0gcmVwKHMsIG5fcm93cykNCiAgZGZfdGVtcCRQQUNfZnJvbnQgPC0gcmVwKHNlcShpX3N0YXJ0LCBpX3N0b3AsIGxlbmd0aC5vdXQgPSAxMCksIDEwKQ0KICBkZl90ZW1wJFBBQ19wYXIgPC0gcmVwKHNlcShpX3N0YXJ0LCBpX3N0b3AsIGxlbmd0aC5vdXQgPSAxMCksIGVhY2g9MTApDQogICNkZl90ZW1wJFRhc2sgPC0gc2FtcGxlKGMoJzJiYWNrJywgJzNiYWNrJyksIDEwMCwgcmVwbGFjZT1UUlVFKQ0KICBkZl90ZXN0IDwtIHJiaW5kKGRmX3Rlc3QsIGRmX3RlbXApDQogIH0NCiAgDQp9DQpgYGANCmBgYHtyfQ0KcHJlZHMgPC0gcHJlZGljdChjaG9vc2VfbW9kZWwsIGRmX3Rlc3QpDQptaW5feiA8LSBtaW4ocHJlZHMpDQptYXhfeiA9IG1heChwcmVkcykNCmRmX3Rlc3QkUHJlZCA8LSBwcmVkcw0KYGBgDQpgYGB7cn0NCiMgVmlld2luZyBhbmdsZXMgZm9yIHRoZSBwbG90cw0KYW5nbGVfY29sdW1ucyA9IGMoIlN0aW0iLCJBemltIiwiQ29sYXRpdHVkZSIpIA0KZGZfYW5nbGVzID0gZGF0YS5mcmFtZShtYXRyaXgobnJvdyA9IDQsIG5jb2wgPSBsZW5ndGgoYW5nbGVfY29sdW1ucykpKSANCmNvbG5hbWVzKGRmX2FuZ2xlcykgPSBhbmdsZV9jb2x1bW5zDQoNCmRmX2FuZ2xlcyRTdGltIDwtIHN0aW1fbGlzdA0KZGZfYW5nbGVzJEF6aW0gPC0gYygwLCAwLCAwLCAwKSAjIFRoZXRhLCBkZWZhdWx0IDANCmRmX2FuZ2xlcyRDb2xhdGl0dWRlIDwtIGMoMTUsIDE1LCAxNSwgMTUpICMgUGhpLCBkZWZhdWx0IDE1DQpgYGANCmBgYHtyfQ0KIyBNYWtlIHBsb3RzDQpmb3IgKHMgaW4gc3RpbV9saXN0KXsNCiAgZGlzcF9kZiA8LSBzdWJzZXQoZGZfdGVzdCwgU3RpbSA9PSBzKQ0KICBwZXJzcCh4PXNlcShpX3N0YXJ0LCBpX3N0b3AsIGxlbmd0aC5vdXQgPSAxMCksIHk9c2VxKGlfc3RhcnQsIGlfc3RvcCwgbGVuZ3RoLm91dCA9IDEwKSwgej1tYXRyaXgoZGlzcF9kZiRQcmVkLCBucm93PTEwLCBuY29sPTEwLGJ5cm93PVRSVUUpLCANCiAgICAgICAgdGhldGEgPSBzdWJzZXQoZGZfYW5nbGVzLCBTdGltID09IHMpJEF6aW0sIHBoaSA9IHN1YnNldChkZl9hbmdsZXMsIFN0aW0gPT0gcykkQ29sYXRpdHVkZSwNCiAgICAgICAgeGxhYj0iUEFDX2Zyb250Iix5bGFiPSJQQUNfcGFyIix6bGFiPSJNZWFuIG5vcm1hbGl6ZWQgc3BlZWQiLCBtYWluPXBhc3RlKHMsIjNiYWNrIiksIHpsaW09YyhtaW5feixtYXhfeikpIA0KfQ0KYGBgDQoNCg0KDQoNCg0KDQoNCg0KDQoNCg0KDQoNCg0KDQoNCg0KDQoNCg==
