# Supplementary material for: Physiology-inspired bifocal fronto-parietal tACS for working memory enhancement
Source: Heliyon. 2024 Sep 6;10(18):e37427. doi: 10.1016/j.heliyon.2024.e37427 (PMC11417162; doi:10.1016/j.heliyon.2024.e37427)
Supplement: Multimedia component 2 [file mmc2.docx]

Supplementary materials

1. **Supplementary methods**
   1. **Subjects**

Exclusion criteria were the presence of any psychiatric or neurological disorder, cognitive deficit based on neuropsychological examination, and drug or alcohol abuse. Neuropsychological examination assessed global cognition, visuo-spatial perception, memory, attention/psychomotor pace, executive functions, and language. Standardized age and education-based normative scores were calculated for each subject. Participants were not included in the study if they achieved a score below 1.5 SD from the norm in two or more scores from all domains. Daily life activities and depressive symptoms were also screened by self-assessment questionnaires. Participants with sub-normal scores were not enrolled in the study. All subjects had normal or corrected-to-normal visual acuity.

In the MUNI cohort, normalized behavioral data were discarded for one subject due to extreme values (3 SD from the group mean; see Fig. S1 for the normalized accuracies with the outlier), rs-fMRI data from two subjects were fully excluded due to excessive head movement. Three subjects from the EPFL cohort were discarded from electrode-level theta-gamma PAC analysis due to the absence of one experimental condition (3-back) for one subject, and bad signal quality for two subjects (more than 80% of the trials were bad).

- 1. **Blinding**

In the MUNI cohort, a single blinded design on the side of subjects was adopted. In the EPFL cohort, the experiment was double blinded in all conditions, although there was no sham control for the burst conditions.

- 1. **Behavioral task**

The visuo-spatial *n*-back task, our main behavioral outcome, was modified from Kramer et al. (2014) with two levels of difficulty. In this task, the participants viewed a stream of stimuli and were asked to indicate whether the current stimulus matched the one from *n* steps (2-back/3-back) earlier in the sequence. All participants were administered the two difficulty levels sequentially, with the difficulty order consistent for everyone, starting with the easier 2-back tests and ending with the more difficult 3-back tests. Participants experienced the same progression as the difficulty levels built on each other. The task items consisted of black squares appearing in nine different positions. The stimulus was present on the screen for 500 ms with an inter-stimulus interval of 2500 ms. A two-alternative forced choice design was used: the participants were asked to press the “YES” (left) button on a response pad if the current stimulus matched the stimulus presented *n* items previously, or press the “NO” (right) button if the stimuli did not match. The online behavioral and baseline EEG version of the task consisted of 130 trials divided into 13 blocks per difficulty level. At the beginning of each block, a fixation cross was displayed for 10 seconds. The baseline fMRI version consisted of 187 trials divided into 17 blocks of the 2-back difficulty only. Both difficulty levels were practiced by the participants during the baseline (opening) session to prevent high learning effects between the first and the second stimulation sessions. For each participant, target accuracy and reaction time (for correct responses) were determined on both *n*-back difficulty levels per block. Further, normalized accuracy and reaction times (e.g. the relative change from the first block) were calculated to control for the individual differences across sessions.

- 1. **Transcranial alternating current stimulation protocols**

Manufacturer information: tACS was performed through a battery-driven stimulator (MUNI cohort and EPFL cohort theta-gamma-burst: DS-5, Digitimer, Hertfordshire, United Kingdom Digitimer; EPFL cohort theta-gamma, gamma-theta, sham: DC-STIMULATOR PLUS, NeuroConn GmbH, Ilmenau, Germany) attached to two pairs of concentric electrodes (NeuroConn GmbH, Germany). We used the T1 MRI scan-based frameless stereotactic neuro-navigation targeting with Brainsight 2 (MUNI lab; Rogue Research Inc., Canada) and TMS navigator software (EPFL lab; Localite Gmbh, Germany). The electrodes were held in place by conductive paste (Ten20 Conductive Paste gel, Weaver and Company, USA).

- 1. **Baseline EEG acquisition details and detailed steps of EEG data preprocessing (e.g., demeaning, specific frequency filtering, visual inspection steps, ICA specifics)**

The monitoring seat was placed 180 cm from the screen and the room temperature was kept constant. Data were analyzed offline using Fieldtrip and Brainstorm toolboxes in Matlab. Raw scalp EEG was first epoched between -7 or -10 to +1 s relative to stimulus presentation, for 2-back and 3-back tasks respectively. Epoched data was demeaned, filtered between 0.1 and 170 Hz, and cleaned from artifacts based on visual inspection and independent component analysis (ICA). First, bad epochs and channels were visually identified by examining features derived from the dynamics of the signal (maximum absolute amplitude and variance of the signal, using Fieldtrip’s ft_rejectvisual function. Second, ICA was computed using the fastica approach (60 components for MUNI, full decomposition for EPFL). Bad components corresponding to eye movements (blinks and horizontal movements), cardiac or muscular activity were then detected and removed from the signal. Finally, the resulting epochs were visually checked one last time before bad channel interpolation (using the average from neighboring channels) and average re-referencing.

- 1. **Exact methodological specifications for theta-gamma PAC computation (e.g., frequency steps, electrode clusters used).**

In the MUNI cohort, participant performance under the sham condition was taken as the baseline, since performances were not recorded during the baseline session. The electrode-level theta-gamma PAC of the resulting clean signal was computed over the last inter-stimulus interval preceding stimulus presentation (i.e., on the -2.5 to 0 s time window). The maximum theta-gamma PAC value through the trials was extracted from each electrode using the Mean Vector Length method (Canolty et al., 2006) on phase frequencies between 4 and 7 Hz (1 Hz step) and amplitude frequencies between 18 and 45 Hz (1 Hz step). Finally, the maximum PAC values from the correct trials of the 3-back task were contrasted (using subtraction) with those of the 2-back task, which thus served as controls. The contrast between the two conditions was finally extracted from the frontal and parietal stimulation targets (“frontal and parietal ROI”, using F4 F6 FC4 FC6, and C4 C6 CP4 CP6 electrode clusters respectively). PAC scores were entered as an additional regressor in our statistical models to investigate their relationship to performance, as measured by the participants’ speed and accuracy.

- 1. **Pre-stimulation task EEG analysis for tACS individualization**

Before the stimulation sessions, the seven electrodes closest to the stimulated targets were analyzed to obtain individualized peak theta and gamma frequencies. EEG data were re-referenced to average reference. Selected EEG signals were filtered by third-order bandpass Butterworth filter in forward and backward directions. The segmentation procedure extracted the first 36 seconds in each task block. The power spectrum was calculated in each block using Fast Fourier Transform and then averaged across blocks. Peak theta and gamma values were identified as the local maximum within the 4-8 and 30-45 Hz frequency intervals.

- 1. **MRI sequence details**

Structural MRI sequence info (TR 2300 ms; TE 2.96 ms; voxel size 1 × 1 × 1 mm; FoV 256 × 256 mm; flip angle 9°; 192 transversal slices). Task-fMRI sequence info (task-fMRI; TR 1250 ms; TE 32 ms; voxel size 2 × 2 × 2 mm; FoV 224 mm; flip angle 65°; 76 transversal slices; 725 scans; multiband factor 4), rs-fMRI sequence info (rs-fMRI; TR 1250 ms; TE 32 ms; voxel size 2 × 2 × 2 mm; FoV 224 mm; flip angle 65°; 76 transversal slices; 480 scans; multiband factor 4). During the acquisition of rs-fMRI data, all subjects were instructed to close their eyes and to try not to think about any specific subject while not falling asleep.

- 1. **MRI data pre-processing**

The data preprocessing pipeline consisted of realign, spatial normalization, and spatial smoothing (FWHM 6 mm). In the rs-fMRI data for the MUNI cohort, this was additionally followed by a regression of nuisance variables (signal originating in white matter, in cerebrospinal fluid, and 24 movement parameters – translations and rotations, their differences, their squares, and squares of the differences) with general linear model and filtration with a high pass on cut-off frequency 1/128 Hz.

We controlled the data for spatial abnormalities (e.g., dropouts) with the Mask Explorer tool (Gajdoš, Mikl, and Mareček 2016) as well as for artifacts related to excessive head movement using framewise displacement (FD) criterion (Power et al. 2012). Each scan with FD > 1.5 mm was excluded from subsequent analyses. In rs-fMRI analysis, we excluded each dataset in which more than 20% of the scans exceeded FD > 0.5 mm (39 rs-fMRI sessions out of a total of 206 rs-fMRI sessions).

- 1. **Pre-stimulation task-fMRI data analysis for tACS**

The task consisted of 17 blocks of the 2-back task (active condition); each block involved 12 trials, in which a white square (0.5 s) was displayed on a black background, followed by followed by a plain black background while waiting for the response (2.5 s). The participants were instructed to decide whether the position of the square was identical to the position of the square presented two trials earlier. The blocks were separated by the display of a fixation cross (baseline condition) lasting for 15 s.

We analyzed the task fMRI data with a general linear model implemented in SPM12. The design matrix in the subject-level analysis contained time courses of task stimulation (block design with active condition and baseline condition) convolved with canonical hemodynamic function and head movement parameters (translations and rotations and their squares) with scans exceeding FD 1.5 mm as nuisance regressors.

A modified pre-stimulation task served for identifying the subject-specific coordinates for subsequent stimulation. We selected stimulation coordinates as the nearest local maxima to the rMFG (MNI: 36 28 36) and rIPL (MNI: 38 -50 36) coordinates on the parametric map of the active vs. baseline contrast, using a threshold of alpha<0.001.

- 1. **Resting state fMRI – Frontoparietal control network coordinates**

| MNI Code | MNI coordinate | Area |
| --- | --- | --- |
| laPFC | [-36 57 9] | left anterior prefrontal cortex |
| raPFC | [34 52 10] | right anterior prefrontal cortex |
| ACC | [3 31 27] | anterior cingulate cortex |
| laIPL | [-52 -49 47] | left anterior inferior parietal lobule |
| raIPL | [52 -46 46] | right anterior inferior parietal lobule |
| ldlPFC | [-50 20 34] | left dorsal lateral prefrontal cortex |
| rdlPFC | [46 14 43] | right dorsal lateral prefrontal cortex |
| lINS | [-31 21 -1] | left insula |
| rINS | [31 22 -2] | right insula |

**Table S1** Coordinates of the fronto-parietal control network as described in Gao and Lin, 2012.

1. **Supplementary results**

**
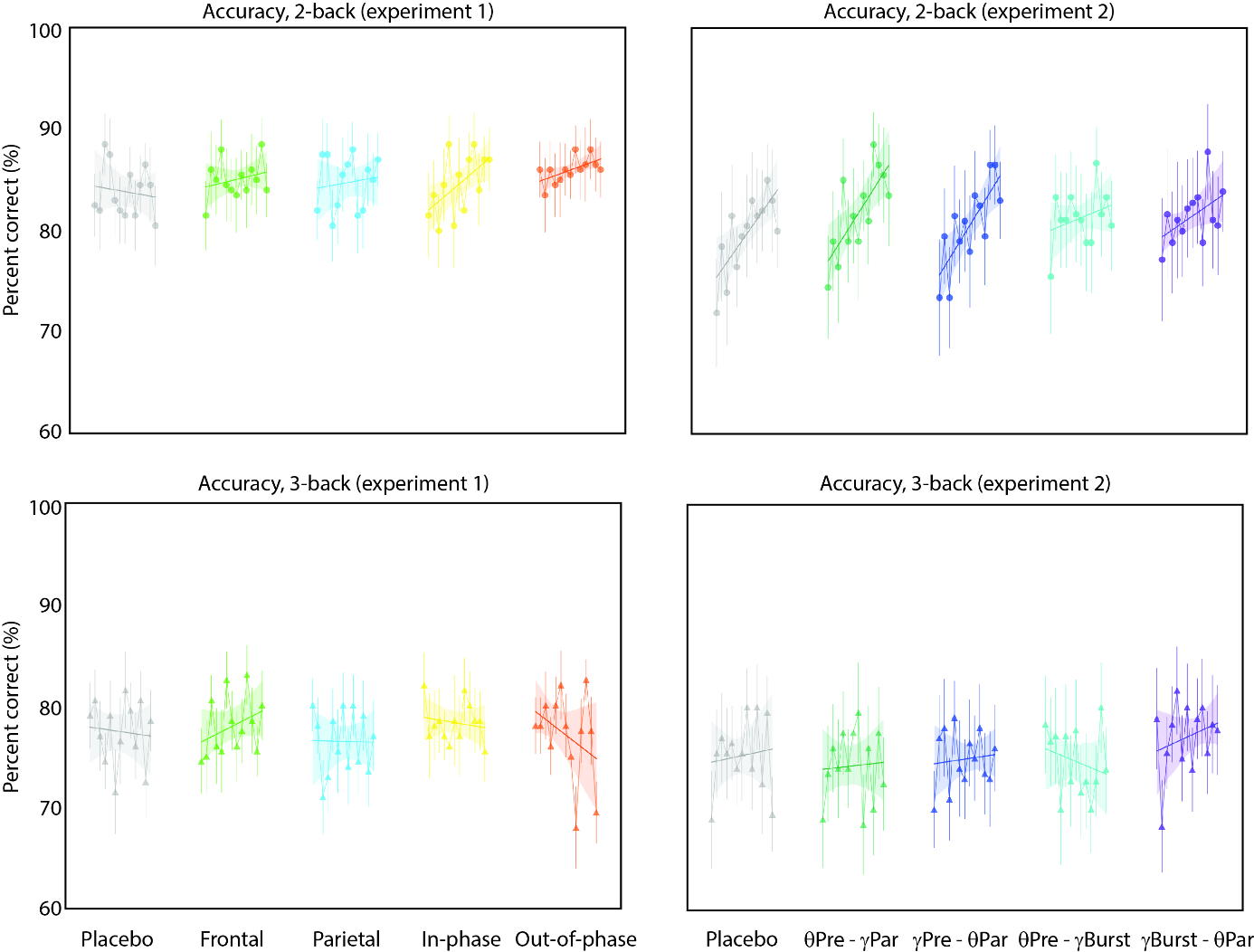
**

**Figure S1** – Uncorrected accuracy in the *n*-back tasks for the MUNI (experiment 1) and EPFL (experiment 2) cohorts.


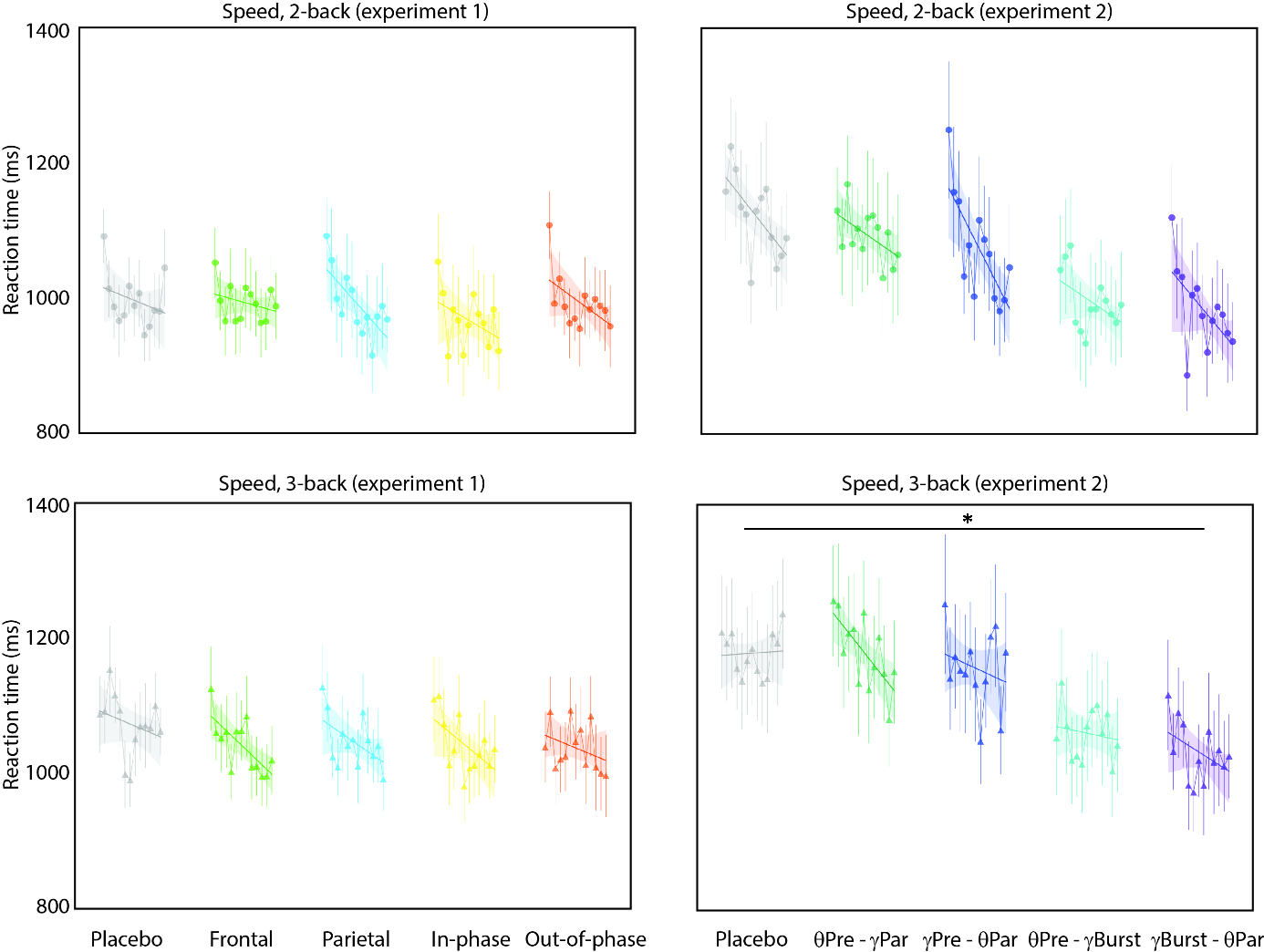


**Figure S2** – Uncorrected reaction times in the *n*-back tasks for the MUNI (experiment 1) and EPFL (experiment 2) cohorts. *sig. p<0.05


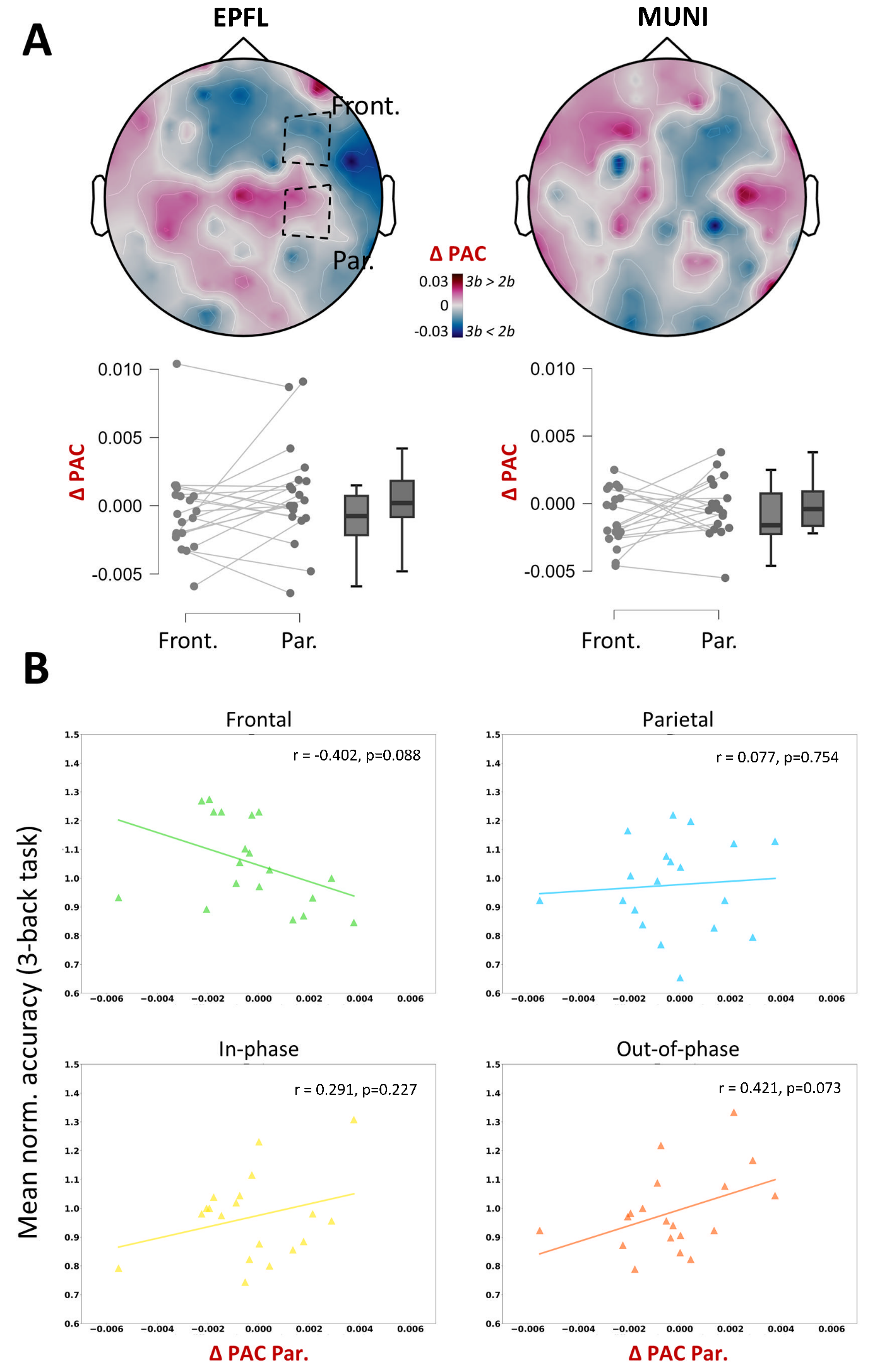


**Figure S3** Relationship between native θ-γ phase-amplitude coupling (PAC) and performance. **A** **(top).** Topography of the difference in native PAC between 3-back and 2-back tasks (Δ PAC) for the EPFL and MUNI cohorts (left and right column respectively). **A (bottom).** Distribution of Δ PAC across the two sites of interest: frontal stimulation (Front.) and parietal stimulation (Par.) sites (see supplementary methods section). **B.** Significant interactions between the mean normalized accuracy during the 3-back task and Δ PAC, as measured in the parietal site for the MUNI cohort.


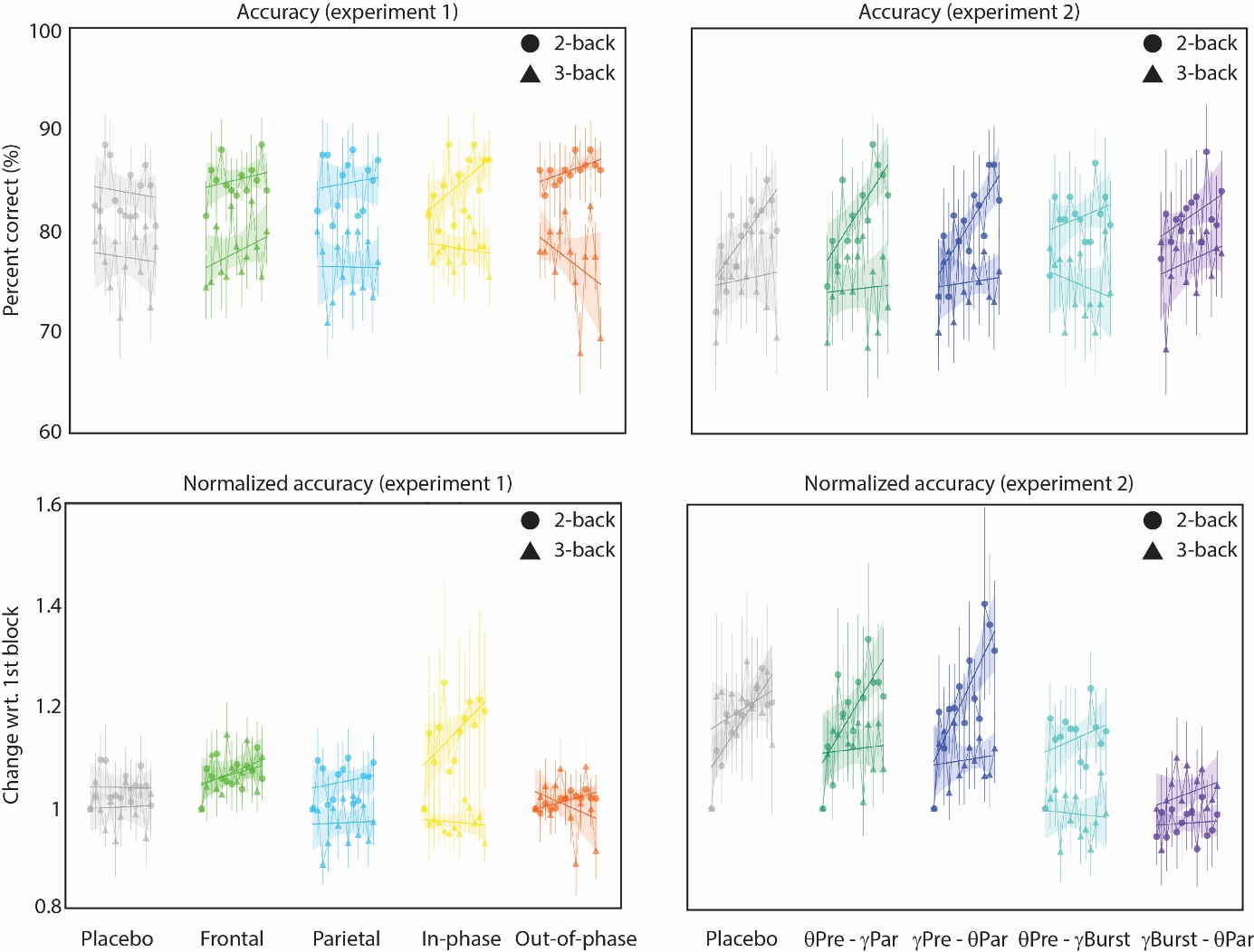
 **Figure S4** – *n*-back task accuracy including the normalized accuracy outlier data.

- 1. **Effect of monofocal and bifocal theta tACS upon rs-fMRI connectivity**

Two significant connections within the fronto-parietal network were found to be affected by the stimulation conditions: the connection between the right anterior prefrontal cortex (raPFC) and left anterior inferior parietal lobule (laIPL) (F[4]= 2.53, p=0.048), and the connection between the raPFC and the right dorsolateral prefrontal cortex (rdlPFC) (F[4]= 2.66, p=0.04). However, further analysis showed that only the connectivity change between raPFC and rdlPFC was significantly different from the sham stimulation condition (t[70]= 2.33, p=0.022). This difference resulted from a decrease in raPFC-rdlPFC connectivity following stimulation as compared to pre-stimulation levels (t[37]= 2.068, p=0.046, see Figure 5). The effect of stimulation on raPFC-laIPL connectivity was due to differences between the active stimulation protocols. These changes were not correlated with any behavioral changes caused by the stimulation.

| Cohort | TACS protocol at the electrode target |  | Induced Electric Fields (V/m) | | |
| --- | --- | --- | --- | --- | --- |
|  |  |  | Mean |  | SD |
| MUNI | Theta | Frontal target | 0.064 |  | 0.025 |
|  |  | Parietal target | 0.064 |  | 0.044 |
|  |  | Symmetry | 0.095 |  | 0.041 |
|  |  | Joint magnitude | 1.389 |  | 0.859 |
| EPFL | Continuous Theta/Gamma | Frontal target | 0.080 |  | 0.048 |
|  |  | Parietal target | 0.058 |  | 0.031 |
|  |  | Symmetry | 0.101 |  | 0.053 |
|  |  | Joint magnitude | 1.461 |  | 0.614 |
|  | Burst Gamma | Frontal target | 0.157 |  | 0.102 |
|  |  | Parietal target | 0.106 |  | 0.064 |
|  |  | Symmetry | 0.192 |  | 0.116 |
|  |  | Joint magnitude | 1.492 |  | 0.632 |

**Table S2** Induced electric fields under different stimulation protocols at both stimulation sites, measures of their symmetry and joint magnitudes.

Supplementary material literature

Canolty, Ryan T., et al. "High gamma power is phase-locked to theta oscillations in human neocortex." science 313.5793 (2006): 1626-1628.

Gajdoš, Martin, Michal Mikl, and Radek Mareček. "Mask_explorer: A tool for exploring brain masks in fMRI group analysis." Computer Methods and Programs in Biomedicine 134 (2016): 155-163.

Gao, Wei, and Weili Lin. "Frontal parietal control network regulates the anti‐correlated default and dorsal attention networks." Human brain mapping 33.1 (2012): 192-202.

Kramer, Joel H., et al. "NIH EXAMINER: conceptualization and development of an executive function battery." Journal of the international neuropsychological society 20.1 (2014): 11-19.

Power, J. D., Barnes, K. A., Snyder, A. Z., Schlaggar, B. L., & Petersen, S. E. (2012). Spurious but systematic correlations in functional connectivity MRI networks arise from subject motion. Neuroimage, 59(3), 2142-2154.

Reinhart, Robert MG, and John A. Nguyen. "Working memory revived in older adults by synchronizing rhythmic brain circuits." Nature neuroscience 22.5 (2019): 820-827.
