# Supplementary material for: Physiology-inspired bifocal fronto-parietal tACS for working memory enhancement
Source: Heliyon. 2024 Sep 6;10(18):e37427. doi: 10.1016/j.heliyon.2024.e37427 (PMC11417162; doi:10.1016/j.heliyon.2024.e37427)
Supplement: Multimedia component 3 [file mmc3.zip › Supplementary materials_Reports on statistics_MUNI.nb.html]

iCOG MS stats for the MUNI data


Code 

- Show All Code
- Hide All Code
- Download Rmd

# iCOG MS stats for the MUNI data


```
library(Matrix)
library(lme4)
library(carData)
library(car)
library(lmerTest)
```


```
Attaching package: ‘lmerTest’

The following object is masked from ‘package:lme4’:

    lmer

The following object is masked from ‘package:stats’:

    step
```


```
library(emmeans)
library(effectsize)
library(ggplot2)
library(Rmisc)
```


```
Loading required package: lattice
Loading required package: plyr
```


```
library(flexplot)
```


```
Attaching package: ‘flexplot’

The following object is masked from ‘package:ggplot2’:

    flip_data
```


```
curr_dir <- getwd()
data_directory <- file.path(paste(curr_dir, '../../../Code/Notebooks/Results', sep='/'))

file_name <- file.path(paste(data_directory, "iCOG_all_data.txt", sep='/'))
df <- read.delim(file_name, header = TRUE, na.strings = "NN")
head(df, 5)
```


```
df$ID <- as.factor(df$ID)
df$School <- as.factor(df$School)
df$Day <- as.factor(df$Day)
df$Task <- as.factor(df$Task)
df$Block <- as.numeric(df$Block)
df$Stim <- as.factor(df$Stim)
df$Acc <- as.numeric(df$Acc)
df$Speed_corr <- as.numeric(df$Speed_corr)
df$D_prime <- as.numeric(df$D_prime)
df$Acc_norm <- as.numeric(df$Acc_norm)
df$Speed_norm <- as.numeric(df$Speed_norm)
df$D_prime_norm <- as.numeric(df$D_prime_norm)
```


# MUNI Data Results


```
df_muni <- subset(df, School == 'MUNI')
df_muni <- droplevels(df_muni)
df_muni$Stim <- factor(df_muni$Stim, levels(df_muni$Stim)[c(1, 2, 5, 3, 4)])
levels(df_muni$Stim)
```


```
[1] "Placebo_loc"       "real_frontal"      "real_parietal"     "real_in-phase"     "real_out-of-phase"
```


## Accuracy Stats

### Simple model, averages

Here, we present different models to characterize each relevant
parameter related to the participants’ performance. In each case, the
models start off from a simple form (i.e., group averages per session),
and they grow in complexity as we add other fixed factors (e.g.,
training blocks) and random effects to account for the variability
across subjects. Each model is compared statistically to its (simpler)
predecessor to assess whether the eventual improvement in data
explainability, as quantified by two conventional criteria (i.e.,
Akaike’s and Bayes’ information criteria), justifies the use of a more
complex model.


```
m0 <- lm(formula = Acc ~ Stim*Task, data=df_muni)
summary(m0)
```


```
Call:
lm(formula = Acc ~ Stim * Task, data = df_muni)

Residuals:
     Min       1Q   Median       3Q      Max 
-0.64577 -0.07548  0.02923  0.12577  0.23538 

Coefficients:
                                 Estimate Std. Error t value Pr(>|t|)    
(Intercept)                      0.838462   0.008997  93.195  < 2e-16 ***
Stimreal_frontal                 0.011923   0.012724   0.937   0.3488    
Stimreal_parietal                0.008846   0.012724   0.695   0.4870    
Stimreal_in-phase                0.007308   0.012724   0.574   0.5658    
Stimreal_out-of-phase            0.021154   0.012724   1.663   0.0965 .  
Task3back                       -0.064231   0.012724  -5.048 4.77e-07 ***
Stimreal_frontal:Task3back      -0.006923   0.017994  -0.385   0.7005    
Stimreal_parietal:Task3back     -0.018462   0.017994  -1.026   0.3050    
Stimreal_in-phase:Task3back      0.001538   0.017994   0.085   0.9319    
Stimreal_out-of-phase:Task3back -0.024615   0.017994  -1.368   0.1714    
---
Signif. codes:  0 ‘***’ 0.001 ‘**’ 0.01 ‘*’ 0.05 ‘.’ 0.1 ‘ ’ 1

Residual standard error: 0.1451 on 2590 degrees of freedom
Multiple R-squared:  0.06304,   Adjusted R-squared:  0.05979 
F-statistic: 19.36 on 9 and 2590 DF,  p-value: < 2.2e-16
```


```
anova(m0)
```


```
Analysis of Variance Table

Response: Acc
            Df Sum Sq Mean Sq  F value Pr(>F)    
Stim         4  0.047  0.0117   0.5574 0.6936    
Task         1  3.552  3.5520 168.7775 <2e-16 ***
Stim:Task    4  0.069  0.0171   0.8144 0.5158    
Residuals 2590 54.508  0.0210                    
---
Signif. codes:  0 ‘***’ 0.001 ‘**’ 0.01 ‘*’ 0.05 ‘.’ 0.1 ‘ ’ 1
```

### Note:

Next, we will test a more complex model including the temporal
component of the task (i.e., the training blocks).

### Linear model, no random effects


```
m1 <- lm(formula = Acc ~ Stim*Task*Block, data=df_muni)
summary(m1)
```


```
Call:
lm(formula = Acc ~ Stim * Task * Block, data = df_muni)

Residuals:
     Min       1Q   Median       3Q      Max 
-0.64154 -0.07683  0.02935  0.12512  0.24846 

Coefficients:
                                        Estimate Std. Error t value Pr(>|t|)    
(Intercept)                            0.8446154  0.0190924  44.238   <2e-16 ***
Stimreal_frontal                      -0.0026923  0.0270008  -0.100   0.9206    
Stimreal_parietal                     -0.0036538  0.0270008  -0.135   0.8924    
Stimreal_in-phase                     -0.0284615  0.0270008  -1.054   0.2919    
Stimreal_out-of-phase                  0.0023077  0.0270008   0.085   0.9319    
Task3back                             -0.0651923  0.0270008  -2.414   0.0158 *  
Block                                 -0.0008791  0.0024054  -0.365   0.7148    
Stimreal_frontal:Task3back            -0.0151923  0.0381849  -0.398   0.6908    
Stimreal_parietal:Task3back           -0.0101923  0.0381849  -0.267   0.7896    
Stimreal_in-phase:Task3back            0.0378846  0.0381849   0.992   0.3212    
Stimreal_out-of-phase:Task3back        0.0159615  0.0381849   0.418   0.6760    
Stimreal_frontal:Block                 0.0020879  0.0034018   0.614   0.5394    
Stimreal_parietal:Block                0.0017857  0.0034018   0.525   0.5997    
Stimreal_in-phase:Block                0.0051099  0.0034018   1.502   0.1332    
Stimreal_out-of-phase:Block            0.0026923  0.0034018   0.791   0.4288    
Task3back:Block                        0.0001374  0.0034018   0.040   0.9678    
Stimreal_frontal:Task3back:Block       0.0011813  0.0048108   0.246   0.8060    
Stimreal_parietal:Task3back:Block     -0.0011813  0.0048108  -0.246   0.8060    
Stimreal_in-phase:Task3back:Block     -0.0051923  0.0048108  -1.079   0.2806    
Stimreal_out-of-phase:Task3back:Block -0.0057967  0.0048108  -1.205   0.2283    
---
Signif. codes:  0 ‘***’ 0.001 ‘**’ 0.01 ‘*’ 0.05 ‘.’ 0.1 ‘ ’ 1

Residual standard error: 0.1451 on 2580 degrees of freedom
Multiple R-squared:  0.06596,   Adjusted R-squared:  0.05908 
F-statistic:  9.59 on 19 and 2580 DF,  p-value: < 2.2e-16
```


```
anova(m1)
```


```
Analysis of Variance Table

Response: Acc
                  Df Sum Sq Mean Sq  F value Pr(>F)    
Stim               4  0.047  0.0117   0.5570 0.6939    
Task               1  3.552  3.5520 168.6515 <2e-16 ***
Block              1  0.007  0.0066   0.3134 0.5757    
Stim:Task          4  0.069  0.0171   0.8137 0.5162    
Stim:Block         4  0.053  0.0133   0.6328 0.6391    
Task:Block         1  0.039  0.0386   1.8343 0.1757    
Stim:Task:Block    4  0.071  0.0178   0.8469 0.4953    
Residuals       2580 54.338  0.0211                    
---
Signif. codes:  0 ‘***’ 0.001 ‘**’ 0.01 ‘*’ 0.05 ‘.’ 0.1 ‘ ’ 1
```

### Note:

When using this more complex model, there is only evidence for the
task difference the previous model had suggested. We will now compare
the two models statistically. #### Compare models


```
anova(m0, m1)
```


```
Analysis of Variance Table

Model 1: Acc ~ Stim * Task
Model 2: Acc ~ Stim * Task * Block
  Res.Df    RSS Df Sum of Sq      F Pr(>F)
1   2590 54.508                           
2   2580 54.338 10   0.16989 0.8066 0.6224
```

### Note:

The addition of the blocks to the model does not significantly
improve the amount of variance explained by the model. However, it may
still be that the addition of random effects will improve the model’s
ability to explain the variance in the data. We will assess the outcome
of this addition next.

#### Random Intercept per subject


```
m2 <- lmer(formula = Acc ~ Stim*Task + (1 | ID), data=df_muni)
summary(m2)
```


```
Linear mixed model fit by REML. t-tests use Satterthwaite's method ['lmerModLmerTest']
Formula: Acc ~ Stim * Task + (1 | ID)
   Data: df_muni

REML criterion at convergence: -3413.1

Scaled residuals: 
    Min      1Q  Median      3Q     Max 
-5.5346 -0.5582  0.1355  0.6863  2.6305 

Random effects:
 Groups   Name        Variance Std.Dev.
 ID       (Intercept) 0.006451 0.08032 
 Residual             0.014893 0.12204 
Number of obs: 2600, groups:  ID, 20

Fixed effects:
                                  Estimate Std. Error         df t value Pr(>|t|)    
(Intercept)                      8.385e-01  1.949e-02  2.543e+01  43.022  < 2e-16 ***
Stimreal_frontal                 1.192e-02  1.070e-02  2.571e+03   1.114   0.2654    
Stimreal_parietal                8.846e-03  1.070e-02  2.571e+03   0.826   0.4086    
Stimreal_in-phase                7.308e-03  1.070e-02  2.571e+03   0.683   0.4948    
Stimreal_out-of-phase            2.115e-02  1.070e-02  2.571e+03   1.976   0.0482 *  
Task3back                       -6.423e-02  1.070e-02  2.571e+03  -6.001 2.24e-09 ***
Stimreal_frontal:Task3back      -6.923e-03  1.514e-02  2.571e+03  -0.457   0.6475    
Stimreal_parietal:Task3back     -1.846e-02  1.514e-02  2.571e+03  -1.220   0.2227    
Stimreal_in-phase:Task3back      1.538e-03  1.514e-02  2.571e+03   0.102   0.9191    
Stimreal_out-of-phase:Task3back -2.462e-02  1.514e-02  2.571e+03  -1.626   0.1040    
---
Signif. codes:  0 ‘***’ 0.001 ‘**’ 0.01 ‘*’ 0.05 ‘.’ 0.1 ‘ ’ 1

Correlation of Fixed Effects:
            (Intr) Stmrl_f Stmrl_p Stmr_- Stm_-- Tsk3bc Stmrl_f:T3 Stmrl_p:T3 S_-:T3
Stmrl_frntl -0.275                                                                  
Stimrl_prtl -0.275  0.500                                                           
Stmrl_n-phs -0.275  0.500   0.500                                                   
Stmrl_t-f-p -0.275  0.500   0.500   0.500                                           
Task3back   -0.275  0.500   0.500   0.500  0.500                                    
Stmrl_fr:T3  0.194 -0.707  -0.354  -0.354 -0.354 -0.707                             
Stmrl_pr:T3  0.194 -0.354  -0.707  -0.354 -0.354 -0.707  0.500                      
Stmrl_n-:T3  0.194 -0.354  -0.354  -0.707 -0.354 -0.707  0.500      0.500           
Stmrl_--:T3  0.194 -0.354  -0.354  -0.354 -0.707 -0.707  0.500      0.500      0.500
```


```
anova(m2)
```


```
Type III Analysis of Variance Table with Satterthwaite's method
          Sum Sq Mean Sq NumDF DenDF  F value Pr(>F)    
Stim      0.0469  0.0117     4  2571   0.7876 0.5331    
Task      3.5520  3.5520     1  2571 238.4953 <2e-16 ***
Stim:Task 0.0686  0.0171     4  2571   1.1507 0.3308    
---
Signif. codes:  0 ‘***’ 0.001 ‘**’ 0.01 ‘*’ 0.05 ‘.’ 0.1 ‘ ’ 1
```

### Note:

When adding a random intercept to the model, there is a trend towards
a significant difference among the blocks. We will now assess whether
the amount of variance explained by this model is significantly larger
than the one explained by the simple model (m0), as our tests revealed
no improvement from the linear model (m1) #### Compare models When
comparing a linear model (lm) to a linear mixed-effect model (lme), the
regular ANOVA does not work, so I will only look at the information
criteria


```
AIC(m0, m2)
```


```
BIC(m0, m2)
```

### Note:

The model including random intercepts is better than the regular
linear model. We will see whether including a random slope improves the
model.

### Random intercept for subjects, and blocks


```
m3 <- lmer(formula = Acc ~ Stim*Task*Block + (1 | ID), data=df_muni)
summary(m3)
```


```
Linear mixed model fit by REML. t-tests use Satterthwaite's method ['lmerModLmerTest']
Formula: Acc ~ Stim * Task * Block + (1 | ID)
   Data: df_muni

REML criterion at convergence: -3318.8

Scaled residuals: 
    Min      1Q  Median      3Q     Max 
-5.5015 -0.5640  0.1303  0.6960  2.7258 

Random effects:
 Groups   Name        Variance Std.Dev.
 ID       (Intercept) 0.006451 0.08032 
 Residual             0.014885 0.12200 
Number of obs: 2600, groups:  ID, 20

Fixed effects:
                                        Estimate Std. Error         df t value Pr(>|t|)    
(Intercept)                            8.446e-01  2.409e-02  5.909e+01  35.065  < 2e-16 ***
Stimreal_frontal                      -2.692e-03  2.270e-02  2.561e+03  -0.119  0.90560    
Stimreal_parietal                     -3.654e-03  2.270e-02  2.561e+03  -0.161  0.87213    
Stimreal_in-phase                     -2.846e-02  2.270e-02  2.561e+03  -1.254  0.21001    
Stimreal_out-of-phase                  2.308e-03  2.270e-02  2.561e+03   0.102  0.91903    
Task3back                             -6.519e-02  2.270e-02  2.561e+03  -2.872  0.00411 ** 
Block                                 -8.791e-04  2.022e-03  2.561e+03  -0.435  0.66379    
Stimreal_frontal:Task3back            -1.519e-02  3.210e-02  2.561e+03  -0.473  0.63607    
Stimreal_parietal:Task3back           -1.019e-02  3.210e-02  2.561e+03  -0.318  0.75089    
Stimreal_in-phase:Task3back            3.788e-02  3.210e-02  2.561e+03   1.180  0.23805    
Stimreal_out-of-phase:Task3back        1.596e-02  3.210e-02  2.561e+03   0.497  0.61908    
Stimreal_frontal:Block                 2.088e-03  2.860e-03  2.561e+03   0.730  0.46541    
Stimreal_parietal:Block                1.786e-03  2.860e-03  2.561e+03   0.624  0.53241    
Stimreal_in-phase:Block                5.110e-03  2.860e-03  2.561e+03   1.787  0.07409 .  
Stimreal_out-of-phase:Block            2.692e-03  2.860e-03  2.561e+03   0.941  0.34658    
Task3back:Block                        1.374e-04  2.860e-03  2.561e+03   0.048  0.96169    
Stimreal_frontal:Task3back:Block       1.181e-03  4.044e-03  2.561e+03   0.292  0.77024    
Stimreal_parietal:Task3back:Block     -1.181e-03  4.044e-03  2.561e+03  -0.292  0.77024    
Stimreal_in-phase:Task3back:Block     -5.192e-03  4.044e-03  2.561e+03  -1.284  0.19932    
Stimreal_out-of-phase:Task3back:Block -5.797e-03  4.044e-03  2.561e+03  -1.433  0.15191    
---
Signif. codes:  0 ‘***’ 0.001 ‘**’ 0.01 ‘*’ 0.05 ‘.’ 0.1 ‘ ’ 1
```


```
Correlation matrix not shown by default, as p = 20 > 12.
Use print(x, correlation=TRUE)  or
    vcov(x)        if you need it
```


```
anova(m3)
```


```
Type III Analysis of Variance Table with Satterthwaite's method
                 Sum Sq Mean Sq NumDF DenDF F value    Pr(>F)    
Stim            0.03607 0.00902     4  2561  0.6058    0.6585    
Task            0.51137 0.51137     1  2561 34.3542 5.184e-09 ***
Block           0.00660 0.00660     1  2561  0.4434    0.5055    
Stim:Task       0.05381 0.01345     4  2561  0.9038    0.4608    
Stim:Block      0.05331 0.01333     4  2561  0.8953    0.4658    
Task:Block      0.03863 0.03863     1  2561  2.5954    0.1073    
Stim:Task:Block 0.07135 0.01784     4  2561  1.1983    0.3095    
---
Signif. codes:  0 ‘***’ 0.001 ‘**’ 0.01 ‘*’ 0.05 ‘.’ 0.1 ‘ ’ 1
```


```
anova(m2, m3)
```


```
refitting model(s) with ML (instead of REML)
```


```
Data: df_muni
Models:
m2: Acc ~ Stim * Task + (1 | ID)
m3: Acc ~ Stim * Task * Block + (1 | ID)
   npar     AIC     BIC logLik deviance  Chisq Df Pr(>Chisq)
m2   12 -3464.4 -3394.0 1744.2  -3488.4                     
m3   22 -3455.8 -3326.9 1749.9  -3499.8 11.472 10     0.3219
```


The inclusion of the blocks does not significantly improve the
model

#### Random intercept and random slope per subject


```
m4 <- lmer(formula = Acc ~ Stim*Task*Block + (1 + Block|ID), data=df_muni)
```


```
boundary (singular) fit: see help('isSingular')
```


```
summary(m4)
```


```
Linear mixed model fit by REML. t-tests use Satterthwaite's method ['lmerModLmerTest']
Formula: Acc ~ Stim * Task * Block + (1 + Block | ID)
   Data: df_muni

REML criterion at convergence: -3318.9

Scaled residuals: 
    Min      1Q  Median      3Q     Max 
-5.5023 -0.5659  0.1291  0.6929  2.7166 

Random effects:
 Groups   Name        Variance  Std.Dev.  Corr 
 ID       (Intercept) 6.625e-03 0.0813916      
          Block       2.336e-08 0.0001528 -1.00
 Residual             1.488e-02 0.1220036      
Number of obs: 2600, groups:  ID, 20

Fixed effects:
                                        Estimate Std. Error         df t value Pr(>|t|)    
(Intercept)                            8.446e-01  2.427e-02  5.170e+01  34.806  < 2e-16 ***
Stimreal_frontal                      -2.692e-03  2.270e-02  2.561e+03  -0.119  0.90559    
Stimreal_parietal                     -3.654e-03  2.270e-02  2.561e+03  -0.161  0.87213    
Stimreal_in-phase                     -2.846e-02  2.270e-02  2.561e+03  -1.254  0.21001    
Stimreal_out-of-phase                  2.308e-03  2.270e-02  2.561e+03   0.102  0.91903    
Task3back                             -6.519e-02  2.270e-02  2.561e+03  -2.872  0.00411 ** 
Block                                 -8.791e-04  2.022e-03  2.543e+03  -0.435  0.66384    
Stimreal_frontal:Task3back            -1.519e-02  3.210e-02  2.561e+03  -0.473  0.63607    
Stimreal_parietal:Task3back           -1.019e-02  3.210e-02  2.561e+03  -0.318  0.75089    
Stimreal_in-phase:Task3back            3.788e-02  3.210e-02  2.561e+03   1.180  0.23805    
Stimreal_out-of-phase:Task3back        1.596e-02  3.210e-02  2.561e+03   0.497  0.61907    
Stimreal_frontal:Block                 2.088e-03  2.860e-03  2.561e+03   0.730  0.46540    
Stimreal_parietal:Block                1.786e-03  2.860e-03  2.561e+03   0.624  0.53241    
Stimreal_in-phase:Block                5.110e-03  2.860e-03  2.561e+03   1.787  0.07409 .  
Stimreal_out-of-phase:Block            2.692e-03  2.860e-03  2.561e+03   0.941  0.34657    
Task3back:Block                        1.374e-04  2.860e-03  2.561e+03   0.048  0.96169    
Stimreal_frontal:Task3back:Block       1.181e-03  4.044e-03  2.561e+03   0.292  0.77024    
Stimreal_parietal:Task3back:Block     -1.181e-03  4.044e-03  2.561e+03  -0.292  0.77024    
Stimreal_in-phase:Task3back:Block     -5.192e-03  4.044e-03  2.561e+03  -1.284  0.19932    
Stimreal_out-of-phase:Task3back:Block -5.797e-03  4.044e-03  2.561e+03  -1.433  0.15190    
---
Signif. codes:  0 ‘***’ 0.001 ‘**’ 0.01 ‘*’ 0.05 ‘.’ 0.1 ‘ ’ 1
```


```
Correlation matrix not shown by default, as p = 20 > 12.
Use print(x, correlation=TRUE)  or
    vcov(x)        if you need it
```


```
optimizer (nloptwrap) convergence code: 0 (OK)
boundary (singular) fit: see help('isSingular')
```


```
anova(m4)
```


```
Type III Analysis of Variance Table with Satterthwaite's method
                 Sum Sq Mean Sq NumDF  DenDF F value    Pr(>F)    
Stim            0.03607 0.00902     4 2561.0  0.6058    0.6585    
Task            0.51137 0.51137     1 2561.0 34.3550 5.182e-09 ***
Block           0.00658 0.00658     1 1447.3  0.4422    0.5062    
Stim:Task       0.05381 0.01345     4 2561.0  0.9038    0.4608    
Stim:Block      0.05331 0.01333     4 2561.0  0.8953    0.4658    
Task:Block      0.03863 0.03863     1 2561.0  2.5955    0.1073    
Stim:Task:Block 0.07135 0.01784     4 2561.0  1.1983    0.3095    
---
Signif. codes:  0 ‘***’ 0.001 ‘**’ 0.01 ‘*’ 0.05 ‘.’ 0.1 ‘ ’ 1
```

#### Compare models, even though this last was singular


```
anova(m2, m4)
```


```
refitting model(s) with ML (instead of REML)
```


```
Data: df_muni
Models:
m2: Acc ~ Stim * Task + (1 | ID)
m4: Acc ~ Stim * Task * Block + (1 + Block | ID)
   npar     AIC     BIC logLik deviance  Chisq Df Pr(>Chisq)
m2   12 -3464.4 -3394.0 1744.2  -3488.4                     
m4   24 -3451.9 -3311.2 1750.0  -3499.9 11.527 12     0.4844
```

### Note:

The addition of a random slope for each subject does not
significantly improve the amount of variance explained by the model.

### Random intercept per subject and per day


```
m5 <- lmer(formula = Acc ~ Stim*Task + (1 + (1|ID) + (1|Day)), data=df_muni)
summary(m5)
```


```
Linear mixed model fit by REML. t-tests use Satterthwaite's method ['lmerModLmerTest']
Formula: Acc ~ Stim * Task + (1 + (1 | ID) + (1 | Day))
   Data: df_muni

REML criterion at convergence: -3426

Scaled residuals: 
    Min      1Q  Median      3Q     Max 
-5.5688 -0.5683  0.1397  0.7044  2.7254 

Random effects:
 Groups   Name        Variance  Std.Dev.
 ID       (Intercept) 0.0064519 0.08032 
 Day      (Intercept) 0.0001508 0.01228 
 Residual             0.0147769 0.12156 
Number of obs: 2600, groups:  ID, 20; Day, 5

Fixed effects:
                                  Estimate Std. Error         df t value Pr(>|t|)    
(Intercept)                      8.397e-01  2.024e-02  2.798e+01  41.487  < 2e-16 ***
Stimreal_frontal                 1.295e-02  1.067e-02  2.568e+03   1.214   0.2249    
Stimreal_parietal                8.343e-03  1.068e-02  2.570e+03   0.781   0.4349    
Stimreal_in-phase                1.871e-03  1.075e-02  2.566e+03   0.174   0.8619    
Stimreal_out-of-phase            1.969e-02  1.068e-02  2.570e+03   1.844   0.0654 .  
Task3back                       -6.423e-02  1.066e-02  2.567e+03  -6.025 1.94e-09 ***
Stimreal_frontal:Task3back      -6.923e-03  1.508e-02  2.567e+03  -0.459   0.6462    
Stimreal_parietal:Task3back     -1.846e-02  1.508e-02  2.567e+03  -1.224   0.2209    
Stimreal_in-phase:Task3back      1.538e-03  1.508e-02  2.567e+03   0.102   0.9187    
Stimreal_out-of-phase:Task3back -2.462e-02  1.508e-02  2.567e+03  -1.633   0.1027    
---
Signif. codes:  0 ‘***’ 0.001 ‘**’ 0.01 ‘*’ 0.05 ‘.’ 0.1 ‘ ’ 1

Correlation of Fixed Effects:
            (Intr) Stmrl_f Stmrl_p Stmr_- Stm_-- Tsk3bc Stmrl_f:T3 Stmrl_p:T3 S_-:T3
Stmrl_frntl -0.263                                                                  
Stimrl_prtl -0.263  0.500                                                           
Stmrl_n-phs -0.263  0.492   0.494                                                   
Stmrl_t-f-p -0.264  0.498   0.499   0.500                                           
Task3back   -0.263  0.500   0.499   0.496  0.499                                    
Stmrl_fr:T3  0.186 -0.707  -0.353  -0.351 -0.353 -0.707                             
Stmrl_pr:T3  0.186 -0.353  -0.706  -0.351 -0.353 -0.707  0.500                      
Stmrl_n-:T3  0.186 -0.353  -0.353  -0.701 -0.353 -0.707  0.500      0.500           
Stmrl_--:T3  0.186 -0.353  -0.353  -0.351 -0.706 -0.707  0.500      0.500      0.500
```


```
anova(m5)
```


```
Type III Analysis of Variance Table with Satterthwaite's method
          Sum Sq Mean Sq NumDF  DenDF  F value Pr(>F)    
Stim      0.0430  0.0107     4 2525.3   0.7274 0.5731    
Task      3.5520  3.5520     1 2567.0 240.3755 <2e-16 ***
Stim:Task 0.0686  0.0171     4 2567.0   1.1598 0.3266    
---
Signif. codes:  0 ‘***’ 0.001 ‘**’ 0.01 ‘*’ 0.05 ‘.’ 0.1 ‘ ’ 1
```


```
anova(m2, m5)
```


```
refitting model(s) with ML (instead of REML)
```


```
Data: df_muni
Models:
m2: Acc ~ Stim * Task + (1 | ID)
m5: Acc ~ Stim * Task + (1 + (1 | ID) + (1 | Day))
   npar     AIC   BIC logLik deviance  Chisq Df Pr(>Chisq)    
m2   12 -3464.4 -3394 1744.2  -3488.4                         
m5   13 -3475.2 -3399 1750.6  -3501.2 12.803  1   0.000346 ***
---
Signif. codes:  0 ‘***’ 0.001 ‘**’ 0.01 ‘*’ 0.05 ‘.’ 0.1 ‘ ’ 1
```

### Model choice

The model including a random intercept for day and for subject is
significantly better than the one including only the random intercept
for subject. As a last test, we will see if adding the blocks to m5
improves the model.


```
m6 <- lmer(formula = Acc ~ Stim*Task*Block + (1 + (1|ID) + (1|Day)), data=df_muni)
summary(m6)
```


```
Linear mixed model fit by REML. t-tests use Satterthwaite's method ['lmerModLmerTest']
Formula: Acc ~ Stim * Task * Block + (1 + (1 | ID) + (1 | Day))
   Data: df_muni

REML criterion at convergence: -3331.7

Scaled residuals: 
    Min      1Q  Median      3Q     Max 
-5.5356 -0.5813  0.1378  0.6998  2.8212 

Random effects:
 Groups   Name        Variance  Std.Dev.
 ID       (Intercept) 0.0064520 0.08032 
 Day      (Intercept) 0.0001508 0.01228 
 Residual             0.0147682 0.12152 
Number of obs: 2600, groups:  ID, 20; Day, 5

Fixed effects:
                                        Estimate Std. Error         df t value Pr(>|t|)    
(Intercept)                            8.459e-01  2.467e-02  6.148e+01  34.292  < 2e-16 ***
Stimreal_frontal                      -1.667e-03  2.261e-02  2.557e+03  -0.074  0.94123    
Stimreal_parietal                     -4.157e-03  2.262e-02  2.558e+03  -0.184  0.85422    
Stimreal_in-phase                     -3.390e-02  2.265e-02  2.560e+03  -1.497  0.13465    
Stimreal_out-of-phase                  8.413e-04  2.262e-02  2.558e+03   0.037  0.97033    
Task3back                             -6.519e-02  2.261e-02  2.557e+03  -2.883  0.00397 ** 
Block                                 -8.791e-04  2.014e-03  2.557e+03  -0.436  0.66255    
Stimreal_frontal:Task3back            -1.519e-02  3.198e-02  2.557e+03  -0.475  0.63474    
Stimreal_parietal:Task3back           -1.019e-02  3.198e-02  2.557e+03  -0.319  0.74994    
Stimreal_in-phase:Task3back            3.788e-02  3.198e-02  2.557e+03   1.185  0.23620    
Stimreal_out-of-phase:Task3back        1.596e-02  3.198e-02  2.557e+03   0.499  0.61769    
Stimreal_frontal:Block                 2.088e-03  2.849e-03  2.557e+03   0.733  0.46365    
Stimreal_parietal:Block                1.786e-03  2.849e-03  2.557e+03   0.627  0.53080    
Stimreal_in-phase:Block                5.110e-03  2.849e-03  2.557e+03   1.794  0.07296 .  
Stimreal_out-of-phase:Block            2.692e-03  2.849e-03  2.557e+03   0.945  0.34468    
Task3back:Block                        1.374e-04  2.849e-03  2.557e+03   0.048  0.96154    
Stimreal_frontal:Task3back:Block       1.181e-03  4.028e-03  2.557e+03   0.293  0.76936    
Stimreal_parietal:Task3back:Block     -1.181e-03  4.028e-03  2.557e+03  -0.293  0.76936    
Stimreal_in-phase:Task3back:Block     -5.192e-03  4.028e-03  2.557e+03  -1.289  0.19755    
Stimreal_out-of-phase:Task3back:Block -5.797e-03  4.028e-03  2.557e+03  -1.439  0.15029    
---
Signif. codes:  0 ‘***’ 0.001 ‘**’ 0.01 ‘*’ 0.05 ‘.’ 0.1 ‘ ’ 1
```


```
Correlation matrix not shown by default, as p = 20 > 12.
Use print(x, correlation=TRUE)  or
    vcov(x)        if you need it
```


```
anova(m6)
```


```
Type III Analysis of Variance Table with Satterthwaite's method
                 Sum Sq Mean Sq NumDF  DenDF F value    Pr(>F)    
Stim            0.04055 0.01014     4 2558.1  0.6864    0.6014    
Task            0.51137 0.51137     1 2557.0 34.6263 4.517e-09 ***
Block           0.00660 0.00660     1 2557.0  0.4469    0.5039    
Stim:Task       0.05381 0.01345     4 2557.0  0.9109    0.4565    
Stim:Block      0.05331 0.01333     4 2557.0  0.9024    0.4616    
Task:Block      0.03863 0.03863     1 2557.0  2.6160    0.1059    
Stim:Task:Block 0.07135 0.01784     4 2557.0  1.2078    0.3054    
---
Signif. codes:  0 ‘***’ 0.001 ‘**’ 0.01 ‘*’ 0.05 ‘.’ 0.1 ‘ ’ 1
```


```
anova(m5, m6)
```


```
refitting model(s) with ML (instead of REML)
```


```
Data: df_muni
Models:
m5: Acc ~ Stim * Task + (1 + (1 | ID) + (1 | Day))
m6: Acc ~ Stim * Task * Block + (1 + (1 | ID) + (1 | Day))
   npar     AIC     BIC logLik deviance  Chisq Df Pr(>Chisq)
m5   13 -3475.2 -3399.0 1750.6  -3501.2                     
m6   23 -3466.7 -3331.9 1756.4  -3512.7 11.563 10     0.3154
```

### Model choice

Adding the blocks to the model including random intercepts for
subjects and days does not improve the model. Therefore, we will keep m5
as the model for accuracy.

### Model for accuracy


```
m_acc <- lmer(formula = Acc ~ Stim*Task + (1 + (1|ID) + (1|Day)), data=df_muni)
summary(m_acc)
```


```
Linear mixed model fit by REML. t-tests use Satterthwaite's method ['lmerModLmerTest']
Formula: Acc ~ Stim * Task + (1 + (1 | ID) + (1 | Day))
   Data: df_muni

REML criterion at convergence: -3426

Scaled residuals: 
    Min      1Q  Median      3Q     Max 
-5.5688 -0.5683  0.1397  0.7044  2.7254 

Random effects:
 Groups   Name        Variance  Std.Dev.
 ID       (Intercept) 0.0064519 0.08032 
 Day      (Intercept) 0.0001508 0.01228 
 Residual             0.0147769 0.12156 
Number of obs: 2600, groups:  ID, 20; Day, 5

Fixed effects:
                                  Estimate Std. Error         df t value Pr(>|t|)    
(Intercept)                      8.397e-01  2.024e-02  2.798e+01  41.487  < 2e-16 ***
Stimreal_frontal                 1.295e-02  1.067e-02  2.568e+03   1.214   0.2249    
Stimreal_parietal                8.343e-03  1.068e-02  2.570e+03   0.781   0.4349    
Stimreal_in-phase                1.871e-03  1.075e-02  2.566e+03   0.174   0.8619    
Stimreal_out-of-phase            1.969e-02  1.068e-02  2.570e+03   1.844   0.0654 .  
Task3back                       -6.423e-02  1.066e-02  2.567e+03  -6.025 1.94e-09 ***
Stimreal_frontal:Task3back      -6.923e-03  1.508e-02  2.567e+03  -0.459   0.6462    
Stimreal_parietal:Task3back     -1.846e-02  1.508e-02  2.567e+03  -1.224   0.2209    
Stimreal_in-phase:Task3back      1.538e-03  1.508e-02  2.567e+03   0.102   0.9187    
Stimreal_out-of-phase:Task3back -2.462e-02  1.508e-02  2.567e+03  -1.633   0.1027    
---
Signif. codes:  0 ‘***’ 0.001 ‘**’ 0.01 ‘*’ 0.05 ‘.’ 0.1 ‘ ’ 1

Correlation of Fixed Effects:
            (Intr) Stmrl_f Stmrl_p Stmr_- Stm_-- Tsk3bc Stmrl_f:T3 Stmrl_p:T3 S_-:T3
Stmrl_frntl -0.263                                                                  
Stimrl_prtl -0.263  0.500                                                           
Stmrl_n-phs -0.263  0.492   0.494                                                   
Stmrl_t-f-p -0.264  0.498   0.499   0.500                                           
Task3back   -0.263  0.500   0.499   0.496  0.499                                    
Stmrl_fr:T3  0.186 -0.707  -0.353  -0.351 -0.353 -0.707                             
Stmrl_pr:T3  0.186 -0.353  -0.706  -0.351 -0.353 -0.707  0.500                      
Stmrl_n-:T3  0.186 -0.353  -0.353  -0.701 -0.353 -0.707  0.500      0.500           
Stmrl_--:T3  0.186 -0.353  -0.353  -0.351 -0.706 -0.707  0.500      0.500      0.500
```


```
anova(m_acc)
```


```
Type III Analysis of Variance Table with Satterthwaite's method
          Sum Sq Mean Sq NumDF  DenDF  F value Pr(>F)    
Stim      0.0430  0.0107     4 2525.3   0.7274 0.5731    
Task      3.5520  3.5520     1 2567.0 240.3755 <2e-16 ***
Stim:Task 0.0686  0.0171     4 2567.0   1.1598 0.3266    
---
Signif. codes:  0 ‘***’ 0.001 ‘**’ 0.01 ‘*’ 0.05 ‘.’ 0.1 ‘ ’ 1
```


```
eta_squared(m_acc)
```


```
# Effect Size for ANOVA (Type III)

Parameter | Eta2 (partial) |       95% CI
-----------------------------------------
Stim      |       1.15e-03 | [0.00, 1.00]
Task      |           0.09 | [0.07, 1.00]
Stim:Task |       1.80e-03 | [0.00, 1.00]

- One-sided CIs: upper bound fixed at [1.00].
```


There is a significant difference among the tasks, so we will test
them separately.

### Accuracy in the 2-back task


```
data_subset <- subset(df_muni, Task == '2back')
data_subset <- droplevels(data_subset)
levels(data_subset$Task)
```


```
[1] "2back"
```


```
m_acc_2back <- lmer(formula = Acc ~ Stim + (1 + (1|ID) + (1|Day)), data=data_subset)
summary(m_acc_2back)
```


```
Linear mixed model fit by REML. t-tests use Satterthwaite's method ['lmerModLmerTest']
Formula: Acc ~ Stim + (1 + (1 | ID) + (1 | Day))
   Data: data_subset

REML criterion at convergence: -1822.9

Scaled residuals: 
    Min      1Q  Median      3Q     Max 
-5.7142 -0.4594  0.1518  0.6200  2.5853 

Random effects:
 Groups   Name        Variance  Std.Dev.
 ID       (Intercept) 0.0077621 0.088103
 Day      (Intercept) 0.0000759 0.008712
 Residual             0.0132527 0.115120
Number of obs: 1300, groups:  ID, 20; Day, 5

Fixed effects:
                       Estimate Std. Error        df t value Pr(>|t|)    
(Intercept)           8.393e-01  2.132e-02 2.424e+01  39.375   <2e-16 ***
Stimreal_frontal      1.238e-02  1.010e-02 1.274e+03   1.225   0.2206    
Stimreal_parietal     7.995e-03  1.013e-02 1.276e+03   0.789   0.4300    
Stimreal_in-phase     4.341e-03  1.021e-02 1.209e+03   0.425   0.6709    
Stimreal_out-of-phase 2.008e-02  1.012e-02 1.276e+03   1.985   0.0474 *  
---
Signif. codes:  0 ‘***’ 0.001 ‘**’ 0.01 ‘*’ 0.05 ‘.’ 0.1 ‘ ’ 1

Correlation of Fixed Effects:
            (Intr) Stmrl_f Stmrl_p Stmr_-
Stmrl_frntl -0.236                       
Stimrl_prtl -0.237  0.500                
Stmrl_n-phs -0.236  0.488   0.491        
Stmrl_t-f-p -0.237  0.497   0.499   0.500
```


```
anova(m_acc_2back)
```


```
Type III Analysis of Variance Table with Satterthwaite's method
       Sum Sq  Mean Sq NumDF  DenDF F value Pr(>F)
Stim 0.061702 0.015425     4 1243.9  1.1639  0.325
```


```
eta_squared(m_acc_2back)
```


```
# Effect Size for ANOVA (Type III)

Parameter | Eta2 (partial) |       95% CI
-----------------------------------------
Stim      |       3.73e-03 | [0.00, 1.00]

- One-sided CIs: upper bound fixed at [1.00].
```

### Note:

There is no evidence for a significant effect of stimulation.

### Accuracy in the 3-back task


```
data_subset <- subset(df_muni, Task == '3back')
data_subset <- droplevels(data_subset)
levels(data_subset$Task)
```


```
[1] "3back"
```


```
m_acc_3back <- lmer(formula = Acc ~ Stim + (1 + (1|ID) + (1|Day)), data=data_subset)
summary(m_acc_3back)
```


```
Linear mixed model fit by REML. t-tests use Satterthwaite's method ['lmerModLmerTest']
Formula: Acc ~ Stim + (1 + (1 | ID) + (1 | Day))
   Data: data_subset

REML criterion at convergence: -1601.1

Scaled residuals: 
    Min      1Q  Median      3Q     Max 
-3.5095 -0.6907  0.0203  0.7066  2.4949 

Random effects:
 Groups   Name        Variance  Std.Dev.
 ID       (Intercept) 0.0056663 0.07527 
 Day      (Intercept) 0.0002133 0.01460 
 Residual             0.0158095 0.12574 
Number of obs: 1300, groups:  ID, 20; Day, 5

Fixed effects:
                        Estimate Std. Error         df t value Pr(>|t|)    
(Intercept)            7.754e-01  1.967e-02  2.791e+01  39.416   <2e-16 ***
Stimreal_frontal       6.276e-03  1.104e-02  1.273e+03   0.568    0.570    
Stimreal_parietal     -9.448e-03  1.107e-02  1.276e+03  -0.853    0.394    
Stimreal_in-phase      2.739e-03  1.120e-02  1.261e+03   0.245    0.807    
Stimreal_out-of-phase -4.757e-03  1.106e-02  1.275e+03  -0.430    0.667    
---
Signif. codes:  0 ‘***’ 0.001 ‘**’ 0.01 ‘*’ 0.05 ‘.’ 0.1 ‘ ’ 1

Correlation of Fixed Effects:
            (Intr) Stmrl_f Stmrl_p Stmr_-
Stmrl_frntl -0.279                       
Stimrl_prtl -0.280  0.500                
Stmrl_n-phs -0.279  0.485   0.488        
Stmrl_t-f-p -0.281  0.496   0.498   0.500
```


```
anova(m_acc_3back)
```


```
Type III Analysis of Variance Table with Satterthwaite's method
       Sum Sq   Mean Sq NumDF  DenDF F value Pr(>F)
Stim 0.039548 0.0098869     4 1268.3  0.6254 0.6445
```


```
eta_squared(m_acc_3back)
```


```
# Effect Size for ANOVA (Type III)

Parameter | Eta2 (partial) |       95% CI
-----------------------------------------
Stim      |       1.97e-03 | [0.00, 1.00]

- One-sided CIs: upper bound fixed at [1.00].
```

### Note:

There is no evidence for significant differences in the 3-back
task.

## Normalized accuracy

Human behavior tends to be highly variable across subjects, which
sometimes makes a direct comparison of performance challenging. For this
reason, we will now assess the change in accuracy experienced by each
participant, which we intend to use as a correction for native
differences present at the start of each session. This correction was
done by dividing the accuracy score of each block by that of the first
training block of each session. Please note this correction was done
within each session (i.e., using the first training block of each
session), as opposed to using the first block of training ever performed
by each participant.

### Group averages


```
m0 <- lm(formula = Acc_norm ~ Stim*Task, data=df_muni)
summary(m0)
```


```
Call:
lm(formula = Acc_norm ~ Stim * Task, data = df_muni)

Residuals:
    Min      1Q  Median      3Q     Max 
-0.8980 -0.1480 -0.0369  0.1011  3.8520 

Coefficients:
                                Estimate Std. Error t value Pr(>|t|)    
(Intercept)                      1.04174    0.01829  56.961  < 2e-16 ***
Stimreal_frontal                 0.02551    0.02586   0.986 0.324044    
Stimreal_parietal                0.01118    0.02586   0.432 0.665592    
Stimreal_in-phase                0.10630    0.02586   4.110 4.08e-05 ***
Stimreal_out-of-phase           -0.02629    0.02586  -1.017 0.309419    
Task3back                       -0.03848    0.02586  -1.488 0.136976    
Stimreal_frontal:Task3back       0.04234    0.03658   1.158 0.247103    
Stimreal_parietal:Task3back     -0.04242    0.03658  -1.160 0.246218    
Stimreal_in-phase:Task3back     -0.13618    0.03658  -3.723 0.000201 ***
Stimreal_out-of-phase:Task3back  0.03006    0.03658   0.822 0.411187    
---
Signif. codes:  0 ‘***’ 0.001 ‘**’ 0.01 ‘*’ 0.05 ‘.’ 0.1 ‘ ’ 1

Residual standard error: 0.2949 on 2590 degrees of freedom
Multiple R-squared:  0.02851,   Adjusted R-squared:  0.02514 
F-statistic: 8.446 on 9 and 2590 DF,  p-value: 1.568e-12
```


```
anova(m0)
```


```
Analysis of Variance Table

Response: Acc_norm
            Df  Sum Sq Mean Sq F value    Pr(>F)    
Stim         4   1.590 0.39751  4.5711  0.001114 ** 
Task         1   2.318 2.31777 26.6526 2.620e-07 ***
Stim:Task    4   2.702 0.67555  7.7683 3.208e-06 ***
Residuals 2590 225.232 0.08696                      
---
Signif. codes:  0 ‘***’ 0.001 ‘**’ 0.01 ‘*’ 0.05 ‘.’ 0.1 ‘ ’ 1
```


Include the blocks


```
m1 <- lm(formula = Acc_norm ~ Stim*Task*Block, data=df_muni)
summary(m1)
```


```
Call:
lm(formula = Acc_norm ~ Stim * Task * Block, data = df_muni)

Residuals:
    Min      1Q  Median      3Q     Max 
-0.8878 -0.1509 -0.0408  0.1014  3.8725 

Coefficients:
                                        Estimate Std. Error t value Pr(>|t|)    
(Intercept)                            1.0430952  0.0388198  26.870   <2e-16 ***
Stimreal_frontal                       0.0038889  0.0548995   0.071    0.944    
Stimreal_parietal                     -0.0042590  0.0548995  -0.078    0.938    
Stimreal_in-phase                      0.0331403  0.0548995   0.604    0.546    
Stimreal_out-of-phase                 -0.0471795  0.0548995  -0.859    0.390    
Task3back                             -0.0428793  0.0548995  -0.781    0.435    
Block                                 -0.0001936  0.0048908  -0.040    0.968    
Stimreal_frontal:Task3back             0.0384165  0.0776397   0.495    0.621    
Stimreal_parietal:Task3back           -0.0268666  0.0776397  -0.346    0.729    
Stimreal_in-phase:Task3back           -0.0537286  0.0776397  -0.692    0.489    
Stimreal_out-of-phase:Task3back        0.0845879  0.0776397   1.089    0.276    
Stimreal_frontal:Block                 0.0030889  0.0069167   0.447    0.655    
Stimreal_parietal:Block                0.0022055  0.0069167   0.319    0.750    
Stimreal_in-phase:Block                0.0104512  0.0069167   1.511    0.131    
Stimreal_out-of-phase:Block            0.0029836  0.0069167   0.431    0.666    
Task3back:Block                        0.0006291  0.0069167   0.091    0.928    
Stimreal_frontal:Task3back:Block       0.0005611  0.0097817   0.057    0.954    
Stimreal_parietal:Task3back:Block     -0.0022224  0.0097817  -0.227    0.820    
Stimreal_in-phase:Task3back:Block     -0.0117787  0.0097817  -1.204    0.229    
Stimreal_out-of-phase:Task3back:Block -0.0077891  0.0097817  -0.796    0.426    
---
Signif. codes:  0 ‘***’ 0.001 ‘**’ 0.01 ‘*’ 0.05 ‘.’ 0.1 ‘ ’ 1

Residual standard error: 0.2951 on 2580 degrees of freedom
Multiple R-squared:  0.03106,   Adjusted R-squared:  0.02393 
F-statistic: 4.353 on 19 and 2580 DF,  p-value: 9.547e-10
```


```
anova(m1)
```


```
Analysis of Variance Table

Response: Acc_norm
                  Df  Sum Sq Mean Sq F value    Pr(>F)    
Stim               4   1.590 0.39751  4.5655  0.001126 ** 
Task               1   2.318 2.31777 26.6196 2.665e-07 ***
Block              1   0.111 0.11070  1.2714  0.259614    
Stim:Task          4   2.702 0.67555  7.7587 3.267e-06 ***
Stim:Block         4   0.153 0.03826  0.4394  0.780208    
Task:Block         1   0.119 0.11903  1.3671  0.242421    
Stim:Task:Block    4   0.208 0.05211  0.5985  0.663756    
Residuals       2580 224.640 0.08707                      
---
Signif. codes:  0 ‘***’ 0.001 ‘**’ 0.01 ‘*’ 0.05 ‘.’ 0.1 ‘ ’ 1
```


```
anova(m0, m1)
```


```
Analysis of Variance Table

Model 1: Acc_norm ~ Stim * Task
Model 2: Acc_norm ~ Stim * Task * Block
  Res.Df    RSS Df Sum of Sq     F Pr(>F)
1   2590 225.23                          
2   2580 224.64 10   0.59121 0.679  0.745
```

### Note:

The inclusion of the blocks in the model did not significantly
improve the amount of variance explained by the model. We will now test
whether the inclusion of random effects brings any improvement. ###
Include random effects #### Random intercept per subject


```
m2 <- lmer(formula = Acc_norm ~ Stim*Task + (1 | ID), data=df_muni)
summary(m2)
```


```
Linear mixed model fit by REML. t-tests use Satterthwaite's method ['lmerModLmerTest']
Formula: Acc_norm ~ Stim * Task + (1 | ID)
   Data: df_muni

REML criterion at convergence: 989.1

Scaled residuals: 
    Min      1Q  Median      3Q     Max 
-3.1275 -0.5095 -0.0697  0.3502 12.6334 

Random effects:
 Groups   Name        Variance Std.Dev.
 ID       (Intercept) 0.004489 0.0670  
 Residual             0.082681 0.2875  
Number of obs: 2600, groups:  ID, 20

Fixed effects:
                                  Estimate Std. Error         df t value Pr(>|t|)    
(Intercept)                        1.04174    0.02329   84.37382  44.729  < 2e-16 ***
Stimreal_frontal                   0.02551    0.02522 2571.00000   1.012 0.311834    
Stimreal_parietal                  0.01118    0.02522 2571.00000   0.443 0.657583    
Stimreal_in-phase                  0.10630    0.02522 2571.00000   4.215 2.58e-05 ***
Stimreal_out-of-phase             -0.02629    0.02522 2571.00000  -1.043 0.297219    
Task3back                         -0.03848    0.02522 2571.00000  -1.526 0.127225    
Stimreal_frontal:Task3back         0.04234    0.03567 2571.00000   1.187 0.235233    
Stimreal_parietal:Task3back       -0.04242    0.03567 2571.00000  -1.189 0.234356    
Stimreal_in-phase:Task3back       -0.13618    0.03567 2571.00000  -3.818 0.000138 ***
Stimreal_out-of-phase:Task3back    0.03006    0.03567 2571.00000   0.843 0.399336    
---
Signif. codes:  0 ‘***’ 0.001 ‘**’ 0.01 ‘*’ 0.05 ‘.’ 0.1 ‘ ’ 1

Correlation of Fixed Effects:
            (Intr) Stmrl_f Stmrl_p Stmr_- Stm_-- Tsk3bc Stmrl_f:T3 Stmrl_p:T3 S_-:T3
Stmrl_frntl -0.541                                                                  
Stimrl_prtl -0.541  0.500                                                           
Stmrl_n-phs -0.541  0.500   0.500                                                   
Stmrl_t-f-p -0.541  0.500   0.500   0.500                                           
Task3back   -0.541  0.500   0.500   0.500  0.500                                    
Stmrl_fr:T3  0.383 -0.707  -0.354  -0.354 -0.354 -0.707                             
Stmrl_pr:T3  0.383 -0.354  -0.707  -0.354 -0.354 -0.707  0.500                      
Stmrl_n-:T3  0.383 -0.354  -0.354  -0.707 -0.354 -0.707  0.500      0.500           
Stmrl_--:T3  0.383 -0.354  -0.354  -0.354 -0.707 -0.707  0.500      0.500      0.500
```


```
anova(m2)
```


```
Type III Analysis of Variance Table with Satterthwaite's method
          Sum Sq Mean Sq NumDF DenDF F value    Pr(>F)    
Stim      1.5901 0.39751     4  2571  4.8078 0.0007287 ***
Task      2.3178 2.31777     1  2571 28.0325 1.294e-07 ***
Stim:Task 2.7022 0.67555     4  2571  8.1705 1.520e-06 ***
---
Signif. codes:  0 ‘***’ 0.001 ‘**’ 0.01 ‘*’ 0.05 ‘.’ 0.1 ‘ ’ 1
```

### Note:

The inclusion of a random intercept results in a model providing
evidence for a significant effect of stimulation, which differs among
the tasks. We will now compare this model to the best model we had
before (m0)


```
AIC(m0, m2)
```


```
BIC(m0, m2)
```

### Note:

In this case, the model including random effects improves the amount
of variance explained by the model.


```
m3 <- lmer(formula = Acc_norm ~ Stim*Task*Block + (1 | ID), data=df_muni)
summary(m3)
```


```
Linear mixed model fit by REML. t-tests use Satterthwaite's method ['lmerModLmerTest']
Formula: Acc_norm ~ Stim * Task * Block + (1 | ID)
   Data: df_muni

REML criterion at convergence: 1070.5

Scaled residuals: 
    Min      1Q  Median      3Q     Max 
-3.0902 -0.5097 -0.0707  0.3426 12.6978 

Random effects:
 Groups   Name        Variance Std.Dev.
 ID       (Intercept) 0.004488 0.06699 
 Residual             0.082773 0.28770 
Number of obs: 2600, groups:  ID, 20

Fixed effects:
                                        Estimate Std. Error         df t value Pr(>|t|)    
(Intercept)                            1.043e+00  4.071e-02  6.504e+02  25.625   <2e-16 ***
Stimreal_frontal                       3.889e-03  5.353e-02  2.561e+03   0.073    0.942    
Stimreal_parietal                     -4.259e-03  5.353e-02  2.561e+03  -0.080    0.937    
Stimreal_in-phase                      3.314e-02  5.353e-02  2.561e+03   0.619    0.536    
Stimreal_out-of-phase                 -4.718e-02  5.353e-02  2.561e+03  -0.881    0.378    
Task3back                             -4.288e-02  5.353e-02  2.561e+03  -0.801    0.423    
Block                                 -1.936e-04  4.769e-03  2.561e+03  -0.041    0.968    
Stimreal_frontal:Task3back             3.842e-02  7.570e-02  2.561e+03   0.507    0.612    
Stimreal_parietal:Task3back           -2.687e-02  7.570e-02  2.561e+03  -0.355    0.723    
Stimreal_in-phase:Task3back           -5.373e-02  7.570e-02  2.561e+03  -0.710    0.478    
Stimreal_out-of-phase:Task3back        8.459e-02  7.570e-02  2.561e+03   1.117    0.264    
Stimreal_frontal:Block                 3.089e-03  6.744e-03  2.561e+03   0.458    0.647    
Stimreal_parietal:Block                2.205e-03  6.744e-03  2.561e+03   0.327    0.744    
Stimreal_in-phase:Block                1.045e-02  6.744e-03  2.561e+03   1.550    0.121    
Stimreal_out-of-phase:Block            2.984e-03  6.744e-03  2.561e+03   0.442    0.658    
Task3back:Block                        6.291e-04  6.744e-03  2.561e+03   0.093    0.926    
Stimreal_frontal:Task3back:Block       5.611e-04  9.537e-03  2.561e+03   0.059    0.953    
Stimreal_parietal:Task3back:Block     -2.222e-03  9.537e-03  2.561e+03  -0.233    0.816    
Stimreal_in-phase:Task3back:Block     -1.178e-02  9.537e-03  2.561e+03  -1.235    0.217    
Stimreal_out-of-phase:Task3back:Block -7.789e-03  9.537e-03  2.561e+03  -0.817    0.414    
---
Signif. codes:  0 ‘***’ 0.001 ‘**’ 0.01 ‘*’ 0.05 ‘.’ 0.1 ‘ ’ 1
```


```
Correlation matrix not shown by default, as p = 20 > 12.
Use print(x, correlation=TRUE)  or
    vcov(x)        if you need it
```


```
anova(m3)
```


```
Type III Analysis of Variance Table with Satterthwaite's method
                 Sum Sq  Mean Sq NumDF DenDF F value Pr(>F)
Stim            0.10406 0.026015     4  2561  0.3143 0.8686
Task            0.17090 0.170904     1  2561  2.0647 0.1509
Block           0.11070 0.110699     1  2561  1.3374 0.2476
Stim:Task       0.34319 0.085799     4  2561  1.0365 0.3868
Stim:Block      0.15304 0.038259     4  2561  0.4622 0.7635
Task:Block      0.11903 0.119032     1  2561  1.4380 0.2306
Stim:Task:Block 0.20844 0.052110     4  2561  0.6295 0.6414
```


```
anova(m2, m3)
```


```
refitting model(s) with ML (instead of REML)
```


```
Data: df_muni
Models:
m2: Acc_norm ~ Stim * Task + (1 | ID)
m3: Acc_norm ~ Stim * Task * Block + (1 | ID)
   npar    AIC    BIC logLik deviance  Chisq Df Pr(>Chisq)
m2   12 952.99 1023.4 -464.5   928.99                     
m3   22 965.81 1094.8 -460.9   921.81 7.1855 10     0.7078
```


Including the blocks does not significantly improve the model. We
will now test whether a random intercept for the days improves it.


```
m4 <- lmer(formula = Acc_norm ~ Stim*Task + (1 + (1|ID) + (1|Day)), data=df_muni)
summary(m4)
```


```
Linear mixed model fit by REML. t-tests use Satterthwaite's method ['lmerModLmerTest']
Formula: Acc_norm ~ Stim * Task + (1 + (1 | ID) + (1 | Day))
   Data: df_muni

REML criterion at convergence: 958.2

Scaled residuals: 
    Min      1Q  Median      3Q     Max 
-3.1435 -0.5151 -0.0883  0.3620 12.5668 

Random effects:
 Groups   Name        Variance Std.Dev.
 ID       (Intercept) 0.004498 0.06707 
 Day      (Intercept) 0.001637 0.04046 
 Residual             0.081388 0.28529 
Number of obs: 2600, groups:  ID, 20; Day, 5

Fixed effects:
                                  Estimate Std. Error         df t value Pr(>|t|)    
(Intercept)                        1.04063    0.02943   20.95039  35.359  < 2e-16 ***
Stimreal_frontal                   0.02815    0.02504 2567.61391   1.124 0.260997    
Stimreal_parietal                  0.01867    0.02508 2568.97687   0.744 0.456642    
Stimreal_in-phase                  0.09798    0.02525 2570.86420   3.881 0.000107 ***
Stimreal_out-of-phase             -0.02256    0.02507 2568.58942  -0.900 0.368132    
Task3back                         -0.03848    0.02502 2566.99934  -1.538 0.124244    
Stimreal_frontal:Task3back         0.04234    0.03539 2566.99934   1.197 0.231548    
Stimreal_parietal:Task3back       -0.04242    0.03539 2566.99934  -1.199 0.230675    
Stimreal_in-phase:Task3back       -0.13618    0.03539 2566.99934  -3.848 0.000122 ***
Stimreal_out-of-phase:Task3back    0.03006    0.03539 2566.99934   0.850 0.395615    
---
Signif. codes:  0 ‘***’ 0.001 ‘**’ 0.01 ‘*’ 0.05 ‘.’ 0.1 ‘ ’ 1

Correlation of Fixed Effects:
            (Intr) Stmrl_f Stmrl_p Stmr_- Stm_-- Tsk3bc Stmrl_f:T3 Stmrl_p:T3 S_-:T3
Stmrl_frntl -0.424                                                                  
Stimrl_prtl -0.425  0.500                                                           
Stmrl_n-phs -0.424  0.491   0.493                                                   
Stmrl_t-f-p -0.426  0.498   0.499   0.500                                           
Task3back   -0.425  0.500   0.499   0.495  0.499                                    
Stmrl_fr:T3  0.301 -0.707  -0.353  -0.350 -0.353 -0.707                             
Stmrl_pr:T3  0.301 -0.353  -0.705  -0.350 -0.353 -0.707  0.500                      
Stmrl_n-:T3  0.301 -0.353  -0.353  -0.701 -0.353 -0.707  0.500      0.500           
Stmrl_--:T3  0.301 -0.353  -0.353  -0.350 -0.706 -0.707  0.500      0.500      0.500
```


```
anova(m4)
```


```
Type III Analysis of Variance Table with Satterthwaite's method
          Sum Sq Mean Sq NumDF DenDF F value    Pr(>F)    
Stim      1.2603 0.31508     4  2559  3.8713  0.003863 ** 
Task      2.3178 2.31777     1  2567 28.4780 1.031e-07 ***
Stim:Task 2.7022 0.67555     4  2567  8.3004 1.194e-06 ***
---
Signif. codes:  0 ‘***’ 0.001 ‘**’ 0.01 ‘*’ 0.05 ‘.’ 0.1 ‘ ’ 1
```


```
anova(m2, m4)
```


```
refitting model(s) with ML (instead of REML)
```


```
Data: df_muni
Models:
m2: Acc_norm ~ Stim * Task + (1 | ID)
m4: Acc_norm ~ Stim * Task + (1 + (1 | ID) + (1 | Day))
   npar    AIC    BIC  logLik deviance  Chisq Df Pr(>Chisq)    
m2   12 952.99 1023.4 -464.50   928.99                         
m4   13 924.87 1001.1 -449.43   898.87 30.127  1  4.048e-08 ***
---
Signif. codes:  0 ‘***’ 0.001 ‘**’ 0.01 ‘*’ 0.05 ‘.’ 0.1 ‘ ’ 1
```


Adding a random intercept for days improves the model.


```
m5 <- lmer(formula = Acc_norm ~ Stim*Task*Block + (1 + (1|ID) + (1|Day)), data=df_muni)
summary(m5)
```


```
Linear mixed model fit by REML. t-tests use Satterthwaite's method ['lmerModLmerTest']
Formula: Acc_norm ~ Stim * Task * Block + (1 + (1 | ID) + (1 | Day))
   Data: df_muni

REML criterion at convergence: 1039.7

Scaled residuals: 
    Min      1Q  Median      3Q     Max 
-3.1059 -0.5190 -0.0824  0.3649 12.6320 

Random effects:
 Groups   Name        Variance Std.Dev.
 ID       (Intercept) 0.004498 0.06707 
 Day      (Intercept) 0.001637 0.04046 
 Residual             0.081475 0.28544 
Number of obs: 2600, groups:  ID, 20; Day, 5

Fixed effects:
                                        Estimate Std. Error         df t value Pr(>|t|)    
(Intercept)                            1.042e+00  4.431e-02  1.056e+02  23.517   <2e-16 ***
Stimreal_frontal                       6.525e-03  5.311e-02  2.557e+03   0.123    0.902    
Stimreal_parietal                      3.232e-03  5.313e-02  2.557e+03   0.061    0.952    
Stimreal_in-phase                      2.483e-02  5.321e-02  2.559e+03   0.467    0.641    
Stimreal_out-of-phase                 -4.345e-02  5.313e-02  2.557e+03  -0.818    0.414    
Task3back                             -4.288e-02  5.311e-02  2.557e+03  -0.807    0.419    
Block                                 -1.936e-04  4.731e-03  2.557e+03  -0.041    0.967    
Stimreal_frontal:Task3back             3.842e-02  7.510e-02  2.557e+03   0.512    0.609    
Stimreal_parietal:Task3back           -2.687e-02  7.510e-02  2.557e+03  -0.358    0.721    
Stimreal_in-phase:Task3back           -5.373e-02  7.510e-02  2.557e+03  -0.715    0.474    
Stimreal_out-of-phase:Task3back        8.459e-02  7.510e-02  2.557e+03   1.126    0.260    
Stimreal_frontal:Block                 3.089e-03  6.691e-03  2.557e+03   0.462    0.644    
Stimreal_parietal:Block                2.205e-03  6.691e-03  2.557e+03   0.330    0.742    
Stimreal_in-phase:Block                1.045e-02  6.691e-03  2.557e+03   1.562    0.118    
Stimreal_out-of-phase:Block            2.984e-03  6.691e-03  2.557e+03   0.446    0.656    
Task3back:Block                        6.291e-04  6.691e-03  2.557e+03   0.094    0.925    
Stimreal_frontal:Task3back:Block       5.611e-04  9.462e-03  2.557e+03   0.059    0.953    
Stimreal_parietal:Task3back:Block     -2.222e-03  9.462e-03  2.557e+03  -0.235    0.814    
Stimreal_in-phase:Task3back:Block     -1.178e-02  9.462e-03  2.557e+03  -1.245    0.213    
Stimreal_out-of-phase:Task3back:Block -7.789e-03  9.462e-03  2.557e+03  -0.823    0.410    
---
Signif. codes:  0 ‘***’ 0.001 ‘**’ 0.01 ‘*’ 0.05 ‘.’ 0.1 ‘ ’ 1
```


```
Correlation matrix not shown by default, as p = 20 > 12.
Use print(x, correlation=TRUE)  or
    vcov(x)        if you need it
```


```
anova(m5)
```


```
Type III Analysis of Variance Table with Satterthwaite's method
                 Sum Sq  Mean Sq NumDF  DenDF F value Pr(>F)
Stim            0.08543 0.021356     4 2558.5  0.2621 0.9023
Task            0.17090 0.170904     1 2557.0  2.0976 0.1477
Block           0.11070 0.110699     1 2557.0  1.3587 0.2439
Stim:Task       0.34319 0.085799     4 2557.0  1.0531 0.3783
Stim:Block      0.15304 0.038259     4 2557.0  0.4696 0.7581
Task:Block      0.11903 0.119032     1 2557.0  1.4610 0.2269
Stim:Task:Block 0.20844 0.052110     4 2557.0  0.6396 0.6343
```


```
anova(m4, m5)
```


```
refitting model(s) with ML (instead of REML)
```


```
Data: df_muni
Models:
m4: Acc_norm ~ Stim * Task + (1 + (1 | ID) + (1 | Day))
m5: Acc_norm ~ Stim * Task * Block + (1 + (1 | ID) + (1 | Day))
   npar    AIC    BIC  logLik deviance  Chisq Df Pr(>Chisq)
m4   13 924.87 1001.1 -449.43   898.87                     
m5   23 937.57 1072.4 -445.78   891.57 7.2997 10     0.6969
```


Adding the blocks does not improve m5. We will last test the effect
of adding a random slope.


```
m6 <- lmer(formula = Acc_norm ~ Stim*Task*Block + (1 + Block|ID), data=df_muni)
```


```
boundary (singular) fit: see help('isSingular')
```


```
summary(m6)
```


```
Linear mixed model fit by REML. t-tests use Satterthwaite's method ['lmerModLmerTest']
Formula: Acc_norm ~ Stim * Task * Block + (1 + Block | ID)
   Data: df_muni

REML criterion at convergence: 1069.4

Scaled residuals: 
    Min      1Q  Median      3Q     Max 
-3.0881 -0.5092 -0.0725  0.3482 12.7383 

Random effects:
 Groups   Name        Variance  Std.Dev. Corr
 ID       (Intercept) 3.069e-03 0.055399     
          Block       2.757e-06 0.001661 1.00
 Residual             8.273e-02 0.287632     
Number of obs: 2600, groups:  ID, 20

Fixed effects:
                                        Estimate Std. Error         df t value Pr(>|t|)    
(Intercept)                            1.043e+00  3.982e-02  5.712e+02  26.198   <2e-16 ***
Stimreal_frontal                       3.889e-03  5.351e-02  2.561e+03   0.073    0.942    
Stimreal_parietal                     -4.259e-03  5.351e-02  2.561e+03  -0.080    0.937    
Stimreal_in-phase                      3.314e-02  5.351e-02  2.561e+03   0.619    0.536    
Stimreal_out-of-phase                 -4.718e-02  5.351e-02  2.561e+03  -0.882    0.378    
Task3back                             -4.288e-02  5.351e-02  2.561e+03  -0.801    0.423    
Block                                 -1.936e-04  4.782e-03  2.214e+03  -0.040    0.968    
Stimreal_frontal:Task3back             3.842e-02  7.568e-02  2.561e+03   0.508    0.612    
Stimreal_parietal:Task3back           -2.687e-02  7.568e-02  2.561e+03  -0.355    0.723    
Stimreal_in-phase:Task3back           -5.373e-02  7.568e-02  2.561e+03  -0.710    0.478    
Stimreal_out-of-phase:Task3back        8.459e-02  7.568e-02  2.561e+03   1.118    0.264    
Stimreal_frontal:Block                 3.089e-03  6.742e-03  2.561e+03   0.458    0.647    
Stimreal_parietal:Block                2.205e-03  6.742e-03  2.561e+03   0.327    0.744    
Stimreal_in-phase:Block                1.045e-02  6.742e-03  2.561e+03   1.550    0.121    
Stimreal_out-of-phase:Block            2.984e-03  6.742e-03  2.561e+03   0.443    0.658    
Task3back:Block                        6.291e-04  6.742e-03  2.561e+03   0.093    0.926    
Stimreal_frontal:Task3back:Block       5.611e-04  9.535e-03  2.561e+03   0.059    0.953    
Stimreal_parietal:Task3back:Block     -2.222e-03  9.535e-03  2.561e+03  -0.233    0.816    
Stimreal_in-phase:Task3back:Block     -1.178e-02  9.535e-03  2.561e+03  -1.235    0.217    
Stimreal_out-of-phase:Task3back:Block -7.789e-03  9.535e-03  2.561e+03  -0.817    0.414    
---
Signif. codes:  0 ‘***’ 0.001 ‘**’ 0.01 ‘*’ 0.05 ‘.’ 0.1 ‘ ’ 1
```


```
Correlation matrix not shown by default, as p = 20 > 12.
Use print(x, correlation=TRUE)  or
    vcov(x)        if you need it
```


```
optimizer (nloptwrap) convergence code: 0 (OK)
boundary (singular) fit: see help('isSingular')
```


```
anova(m6)
```


```
Type III Analysis of Variance Table with Satterthwaite's method
                 Sum Sq  Mean Sq NumDF   DenDF F value Pr(>F)
Stim            0.10406 0.026015     4 2561.00  0.3144 0.8685
Task            0.17090 0.170904     1 2561.00  2.0658 0.1508
Block           0.10437 0.104368     1  157.94  1.2615 0.2631
Stim:Task       0.34319 0.085799     4 2561.00  1.0371 0.3865
Stim:Block      0.15304 0.038259     4 2561.00  0.4624 0.7634
Task:Block      0.11903 0.119032     1 2561.00  1.4388 0.2305
Stim:Task:Block 0.20844 0.052110     4 2561.00  0.6299 0.6412
```

### Model choice

The fit of the model including a random slope is singular, which was
to be expected as the inclusion of the blocks in the different tested
models never improved the amount of variance explained. Therefore, we
will keep m4, which include random intercepts for individuals and for
the days.


```
m_acc_norm <- lmer(formula = Acc_norm ~ Stim*Task + (1 + (1|ID) + (1|Day)), data=df_muni)
summary(m_acc_norm)
```


```
Linear mixed model fit by REML. t-tests use Satterthwaite's method ['lmerModLmerTest']
Formula: Acc_norm ~ Stim * Task + (1 + (1 | ID) + (1 | Day))
   Data: df_muni

REML criterion at convergence: 958.2

Scaled residuals: 
    Min      1Q  Median      3Q     Max 
-3.1435 -0.5151 -0.0883  0.3620 12.5668 

Random effects:
 Groups   Name        Variance Std.Dev.
 ID       (Intercept) 0.004498 0.06707 
 Day      (Intercept) 0.001637 0.04046 
 Residual             0.081388 0.28529 
Number of obs: 2600, groups:  ID, 20; Day, 5

Fixed effects:
                                  Estimate Std. Error         df t value Pr(>|t|)    
(Intercept)                        1.04063    0.02943   20.95039  35.359  < 2e-16 ***
Stimreal_frontal                   0.02815    0.02504 2567.61391   1.124 0.260997    
Stimreal_parietal                  0.01867    0.02508 2568.97687   0.744 0.456642    
Stimreal_in-phase                  0.09798    0.02525 2570.86420   3.881 0.000107 ***
Stimreal_out-of-phase             -0.02256    0.02507 2568.58942  -0.900 0.368132    
Task3back                         -0.03848    0.02502 2566.99934  -1.538 0.124244    
Stimreal_frontal:Task3back         0.04234    0.03539 2566.99934   1.197 0.231548    
Stimreal_parietal:Task3back       -0.04242    0.03539 2566.99934  -1.199 0.230675    
Stimreal_in-phase:Task3back       -0.13618    0.03539 2566.99934  -3.848 0.000122 ***
Stimreal_out-of-phase:Task3back    0.03006    0.03539 2566.99934   0.850 0.395615    
---
Signif. codes:  0 ‘***’ 0.001 ‘**’ 0.01 ‘*’ 0.05 ‘.’ 0.1 ‘ ’ 1

Correlation of Fixed Effects:
            (Intr) Stmrl_f Stmrl_p Stmr_- Stm_-- Tsk3bc Stmrl_f:T3 Stmrl_p:T3 S_-:T3
Stmrl_frntl -0.424                                                                  
Stimrl_prtl -0.425  0.500                                                           
Stmrl_n-phs -0.424  0.491   0.493                                                   
Stmrl_t-f-p -0.426  0.498   0.499   0.500                                           
Task3back   -0.425  0.500   0.499   0.495  0.499                                    
Stmrl_fr:T3  0.301 -0.707  -0.353  -0.350 -0.353 -0.707                             
Stmrl_pr:T3  0.301 -0.353  -0.705  -0.350 -0.353 -0.707  0.500                      
Stmrl_n-:T3  0.301 -0.353  -0.353  -0.701 -0.353 -0.707  0.500      0.500           
Stmrl_--:T3  0.301 -0.353  -0.353  -0.350 -0.706 -0.707  0.500      0.500      0.500
```


```
anova(m_acc_norm)
```


```
Type III Analysis of Variance Table with Satterthwaite's method
          Sum Sq Mean Sq NumDF DenDF F value    Pr(>F)    
Stim      1.2603 0.31508     4  2559  3.8713  0.003863 ** 
Task      2.3178 2.31777     1  2567 28.4780 1.031e-07 ***
Stim:Task 2.7022 0.67555     4  2567  8.3004 1.194e-06 ***
---
Signif. codes:  0 ‘***’ 0.001 ‘**’ 0.01 ‘*’ 0.05 ‘.’ 0.1 ‘ ’ 1
```


```
eta_squared(m_acc_norm)
```


```
# Effect Size for ANOVA (Type III)

Parameter | Eta2 (partial) |       95% CI
-----------------------------------------
Stim      |       6.01e-03 | [0.00, 1.00]
Task      |           0.01 | [0.01, 1.00]
Stim:Task |           0.01 | [0.01, 1.00]

- One-sided CIs: upper bound fixed at [1.00].
```


```
my_model <- m_acc_norm
level_1 <- "Stim"

my_model.compare <- emmeans(my_model, level_1, by="Task")
my_model.compare.pairs <- pairs(my_model.compare, adjust='tukey')
test(my_model.compare.pairs, side='two-sided')
```


```
Task = 2back:
 contrast                              estimate     SE   df t.ratio p.value
 Placebo_loc - real_frontal            -0.02815 0.0250 2568  -1.124  0.7938
 Placebo_loc - real_parietal           -0.01867 0.0251 2569  -0.744  0.9460
 Placebo_loc - (real_in-phase)         -0.09798 0.0253 2571  -3.877  0.0010
 Placebo_loc - (real_out-of-phase)      0.02256 0.0251 2569   0.900  0.8970
 real_frontal - real_parietal           0.00948 0.0251 2569   0.378  0.9957
 real_frontal - (real_in-phase)        -0.06984 0.0254 2568  -2.749  0.0474
 real_frontal - (real_out-of-phase)     0.05071 0.0251 2570   2.019  0.2572
 real_parietal - (real_in-phase)       -0.07931 0.0254 2569  -3.126  0.0154
 real_parietal - (real_out-of-phase)    0.04123 0.0251 2569   1.643  0.4702
 (real_in-phase) - (real_out-of-phase)  0.12055 0.0252 2571   4.789  <.0001

Task = 3back:
 contrast                              estimate     SE   df t.ratio p.value
 Placebo_loc - real_frontal            -0.07049 0.0250 2568  -2.815  0.0394
 Placebo_loc - real_parietal            0.02375 0.0251 2569   0.947  0.8785
 Placebo_loc - (real_in-phase)          0.03820 0.0253 2571   1.511  0.5551
 Placebo_loc - (real_out-of-phase)     -0.00750 0.0251 2569  -0.299  0.9983
 real_frontal - real_parietal           0.09424 0.0251 2569   3.759  0.0016
 real_frontal - (real_in-phase)         0.10869 0.0254 2568   4.278  0.0002
 real_frontal - (real_out-of-phase)     0.06299 0.0251 2570   2.508  0.0892
 real_parietal - (real_in-phase)        0.01444 0.0254 2569   0.569  0.9795
 real_parietal - (real_out-of-phase)   -0.03125 0.0251 2569  -1.245  0.7249
 (real_in-phase) - (real_out-of-phase) -0.04570 0.0252 2571  -1.816  0.3647

Degrees-of-freedom method: kenward-roger 
P value adjustment: tukey method for comparing a family of 5 estimates
```


```
confint(my_model.compare, calc = c(n = ~.wgt.))
```


```
Task = 2back:
 Stim              emmean     SE   df   n lower.CL upper.CL
 Placebo_loc        1.041 0.0294 20.9 260    0.979     1.10
 real_frontal       1.069 0.0295 21.0 260    1.008     1.13
 real_parietal      1.059 0.0295 21.0 260    0.998     1.12
 real_in-phase      1.139 0.0296 21.2 260    1.077     1.20
 real_out-of-phase  1.018 0.0294 20.9 260    0.957     1.08

Task = 3back:
 Stim              emmean     SE   df   n lower.CL upper.CL
 Placebo_loc        1.002 0.0294 20.9 260    0.941     1.06
 real_frontal       1.073 0.0295 21.0 260    1.011     1.13
 real_parietal      0.978 0.0295 21.0 260    0.917     1.04
 real_in-phase      0.964 0.0296 21.2 260    0.903     1.03
 real_out-of-phase  1.010 0.0294 20.9 260    0.948     1.07

Degrees-of-freedom method: kenward-roger 
Confidence level used: 0.95
```


```
eff_size(my_model.compare, sigma = sigma(my_model), edf = 23)
```


```
Task = 2back:
 contrast                              effect.size     SE   df lower.CL upper.CL
 Placebo_loc - real_frontal                -0.0987 0.0890 20.9  -0.2837   0.0864
 Placebo_loc - real_parietal               -0.0654 0.0885 20.9  -0.2494   0.1185
 Placebo_loc - (real_in-phase)             -0.3435 0.1020 20.9  -0.5557  -0.1312
 Placebo_loc - (real_out-of-phase)          0.0791 0.0886 20.9  -0.1053   0.2635
 real_frontal - real_parietal               0.0332 0.0880 21.0  -0.1498   0.2162
 real_frontal - (real_in-phase)            -0.2448 0.0961 21.0  -0.4446  -0.0450
 real_frontal - (real_out-of-phase)         0.1778 0.0919 20.9  -0.0133   0.3688
 real_parietal - (real_in-phase)           -0.2780 0.0979 21.0  -0.4817  -0.0744
 real_parietal - (real_out-of-phase)        0.1445 0.0905 20.9  -0.0438   0.3328
 (real_in-phase) - (real_out-of-phase)      0.4225 0.1080 20.9   0.1979   0.6472

Task = 3back:
 contrast                              effect.size     SE   df lower.CL upper.CL
 Placebo_loc - real_frontal                -0.2471 0.0950 20.9  -0.4447  -0.0495
 Placebo_loc - real_parietal                0.0833 0.0888 20.9  -0.1014   0.2679
 Placebo_loc - (real_in-phase)              0.1339 0.0908 20.9  -0.0549   0.3227
 Placebo_loc - (real_out-of-phase)         -0.0263 0.0880 20.9  -0.2092   0.1567
 real_frontal - real_parietal               0.3304 0.1005 21.0   0.1214   0.5393
 real_frontal - (real_in-phase)             0.3810 0.1053 21.0   0.1620   0.5999
 real_frontal - (real_out-of-phase)         0.2208 0.0939 20.9   0.0255   0.4161
 real_parietal - (real_in-phase)            0.0506 0.0892 21.0  -0.1350   0.2362
 real_parietal - (real_out-of-phase)       -0.1096 0.0895 20.9  -0.2956   0.0765
 (real_in-phase) - (real_out-of-phase)     -0.1602 0.0913 20.9  -0.3502   0.0298

sigma used for effect sizes: 0.2853 
Degrees-of-freedom method: inherited from kenward-roger when re-gridding 
Confidence level used: 0.95
```

### Normalized accuracy in the 2-back task


```
data_subset <- subset(df_muni, Task == '2back')
data_subset <- droplevels(data_subset)
levels(data_subset$Task)
```


```
[1] "2back"
```


```
m_acc_norm_2back <- lmer(formula = Acc_norm ~ Stim + (1 + (1|ID) + (1|Day)), data=data_subset)
summary(m_acc_norm_2back)
```


```
Linear mixed model fit by REML. t-tests use Satterthwaite's method ['lmerModLmerTest']
Formula: Acc_norm ~ Stim + (1 + (1 | ID) + (1 | Day))
   Data: data_subset

REML criterion at convergence: 820.7

Scaled residuals: 
    Min      1Q  Median      3Q     Max 
-2.8774 -0.4837 -0.0394  0.3385 10.5104 

Random effects:
 Groups   Name        Variance Std.Dev.
 ID       (Intercept) 0.014534 0.12056 
 Day      (Intercept) 0.006298 0.07936 
 Residual             0.103495 0.32171 
Number of obs: 1300, groups:  ID, 20; Day, 5

Fixed effects:
                        Estimate Std. Error         df t value Pr(>|t|)    
(Intercept)              1.03928    0.04885   11.75536  21.275  9.5e-11 ***
Stimreal_frontal         0.03228    0.02825 1272.41808   1.142  0.25348    
Stimreal_parietal        0.02962    0.02835 1273.40041   1.045  0.29631    
Stimreal_in-phase        0.08822    0.02874 1275.82045   3.069  0.00219 ** 
Stimreal_out-of-phase   -0.02114    0.02832 1273.11246  -0.746  0.45556    
---
Signif. codes:  0 ‘***’ 0.001 ‘**’ 0.01 ‘*’ 0.05 ‘.’ 0.1 ‘ ’ 1

Correlation of Fixed Effects:
            (Intr) Stmrl_f Stmrl_p Stmr_-
Stmrl_frntl -0.288                       
Stimrl_prtl -0.289  0.499                
Stmrl_n-phs -0.288  0.481   0.486        
Stmrl_t-f-p -0.290  0.495   0.498   0.500
```


```
anova(m_acc_norm_2back)
```


```
Type III Analysis of Variance Table with Satterthwaite's method
     Sum Sq Mean Sq NumDF  DenDF F value   Pr(>F)   
Stim 1.7298 0.43244     4 1274.3  4.1784 0.002293 **
---
Signif. codes:  0 ‘***’ 0.001 ‘**’ 0.01 ‘*’ 0.05 ‘.’ 0.1 ‘ ’ 1
```


```
eta_squared(m_acc_norm_2back)
```


```
# Effect Size for ANOVA (Type III)

Parameter | Eta2 (partial) |       95% CI
-----------------------------------------
Stim      |           0.01 | [0.00, 1.00]

- One-sided CIs: upper bound fixed at [1.00].
```

### Note:

There is an effect of stimulation in the 2-back task. We will now do
post-hoc comparisons to see where these differences come from.


```
my_model <- m_acc_norm_2back
level_1 <- "Stim"

my_model.compare <- emmeans(my_model, level_1)
my_model.compare.pairs <- pairs(my_model.compare, adjust='tukey')
test(my_model.compare.pairs, side='two-sided')
```


```
 contrast                              estimate     SE   df t.ratio p.value
 Placebo_loc - real_frontal            -0.03228 0.0283 1272  -1.142  0.7839
 Placebo_loc - real_parietal           -0.02962 0.0284 1273  -1.044  0.8346
 Placebo_loc - (real_in-phase)         -0.08822 0.0288 1276  -3.065  0.0188
 Placebo_loc - (real_out-of-phase)      0.02114 0.0283 1273   0.746  0.9455
 real_frontal - real_parietal           0.00265 0.0283 1273   0.094  1.0000
 real_frontal - (real_in-phase)        -0.05594 0.0291 1276  -1.924  0.3050
 real_frontal - (real_out-of-phase)     0.05341 0.0284 1274   1.878  0.3296
 real_parietal - (real_in-phase)       -0.05860 0.0290 1276  -2.021  0.2566
 real_parietal - (real_out-of-phase)    0.05076 0.0284 1274   1.787  0.3814
 (real_in-phase) - (real_out-of-phase)  0.10935 0.0286 1275   3.830  0.0013

Degrees-of-freedom method: kenward-roger 
P value adjustment: tukey method for comparing a family of 5 estimates
```


```
confint(my_model.compare, calc = c(n = ~.wgt.))
```


```
 Stim              emmean     SE   df   n lower.CL upper.CL
 Placebo_loc         1.04 0.0489 11.8 260    0.933     1.15
 real_frontal        1.07 0.0489 11.8 260    0.965     1.18
 real_parietal       1.07 0.0489 11.8 260    0.962     1.18
 real_in-phase       1.13 0.0490 11.9 260    1.021     1.23
 real_out-of-phase   1.02 0.0489 11.8 260    0.911     1.12

Degrees-of-freedom method: kenward-roger 
Confidence level used: 0.95
```


```
eff_size(my_model.compare, sigma = sigma(my_model), edf = 23)
```


```
 contrast                              effect.size     SE   df lower.CL upper.CL
 Placebo_loc - real_frontal               -0.10033 0.0891 11.8  -0.2948   0.0942
 Placebo_loc - real_parietal              -0.09207 0.0892 11.8  -0.2868   0.1027
 Placebo_loc - (real_in-phase)            -0.27422 0.0982 11.8  -0.4886  -0.0598
 Placebo_loc - (real_out-of-phase)         0.06570 0.0886 11.8  -0.1277   0.2591
 real_frontal - real_parietal              0.00825 0.0880 11.8  -0.1839   0.2005
 real_frontal - (real_in-phase)           -0.17389 0.0940 11.8  -0.3790   0.0312
 real_frontal - (real_out-of-phase)        0.16603 0.0917 11.8  -0.0343   0.3663
 real_parietal - (real_in-phase)          -0.18215 0.0941 11.8  -0.3875   0.0232
 real_parietal - (real_out-of-phase)       0.15777 0.0913 11.8  -0.0416   0.3571
 (real_in-phase) - (real_out-of-phase)     0.33992 0.1019 11.8   0.1173   0.5625

sigma used for effect sizes: 0.3217 
Degrees-of-freedom method: inherited from kenward-roger when re-gridding 
Confidence level used: 0.95
```

### Note:

The post-hoc tests revealed that the improvement in the accuracy was
significantly higher when participants received in-phase stimulation.
However, exploration of the individual scores obtained by participants
revealed the presence of what could be an outlier, with a single
participant (9004) having a single score above 4 (i.e., 400% improvement
in one of the 13 blocks). Therefore, we will now show the same analysis
and the results obtained when excluding this participant.


```
data_subset <- subset(df_muni, Task == '2back' & ID != "9004")
data_subset <- droplevels(data_subset)
levels(data_subset$Task)
```


```
[1] "2back"
```


```
levels(data_subset$ID)
```


```
 [1] "9001" "9002" "9003" "9005" "9006" "9007" "9008" "9009" "9010" "9011" "9012" "9014" "9015" "9016" "9017" "9018" "9019" "9020" "9021"
```


```
m_acc_norm_2back_exclude_9004 <- lmer(formula = Acc_norm ~ Stim + (1 + (1|ID) + (1|Day)), data=data_subset)
summary(m_acc_norm_2back_exclude_9004)
```


```
Linear mixed model fit by REML. t-tests use Satterthwaite's method ['lmerModLmerTest']
Formula: Acc_norm ~ Stim + (1 + (1 | ID) + (1 | Day))
   Data: data_subset

REML criterion at convergence: -368.5

Scaled residuals: 
    Min      1Q  Median      3Q     Max 
-4.2514 -0.5446 -0.0461  0.4852  4.8056 

Random effects:
 Groups   Name        Variance Std.Dev.
 ID       (Intercept) 0.007055 0.08400 
 Day      (Intercept) 0.001054 0.03247 
 Residual             0.040639 0.20159 
Number of obs: 1235, groups:  ID, 19; Day, 5

Fixed effects:
                        Estimate Std. Error         df t value Pr(>|t|)    
(Intercept)              1.04352    0.02733   23.25271  38.180   <2e-16 ***
Stimreal_frontal         0.03380    0.01821 1210.46974   1.856   0.0637 .  
Stimreal_parietal        0.01872    0.01825 1211.31613   1.026   0.3051    
Stimreal_in-phase       -0.03062    0.01835 1211.93846  -1.669   0.0954 .  
Stimreal_out-of-phase   -0.01927    0.01818 1209.67400  -1.060   0.2894    
---
Signif. codes:  0 ‘***’ 0.001 ‘**’ 0.01 ‘*’ 0.05 ‘.’ 0.1 ‘ ’ 1

Correlation of Fixed Effects:
            (Intr) Stmrl_f Stmrl_p Stmr_-
Stmrl_frntl -0.330                       
Stimrl_prtl -0.331  0.498                
Stmrl_n-phs -0.330  0.480   0.489        
Stmrl_t-f-p -0.332  0.493   0.496   0.498
```


```
anova(m_acc_norm_2back_exclude_9004)
```


```
Type III Analysis of Variance Table with Satterthwaite's method
      Sum Sq Mean Sq NumDF  DenDF F value  Pr(>F)   
Stim 0.65426 0.16357     4 1210.9  4.0249 0.00301 **
---
Signif. codes:  0 ‘***’ 0.001 ‘**’ 0.01 ‘*’ 0.05 ‘.’ 0.1 ‘ ’ 1
```


```
eta_squared(m_acc_norm_2back_exclude_9004)
```


```
# Effect Size for ANOVA (Type III)

Parameter | Eta2 (partial) |       95% CI
-----------------------------------------
Stim      |           0.01 | [0.00, 1.00]

- One-sided CIs: upper bound fixed at [1.00].
```


```
my_model <- m_acc_norm_2back_exclude_9004
level_1 <- "Stim"

my_model.compare <- emmeans(my_model, level_1)
my_model.compare.pairs <- pairs(my_model.compare, adjust='tukey')
test(my_model.compare.pairs, side='two-sided')
```


```
 contrast                              estimate     SE   df t.ratio p.value
 Placebo_loc - real_frontal             -0.0338 0.0182 1210  -1.855  0.3425
 Placebo_loc - real_parietal            -0.0187 0.0183 1211  -1.025  0.8439
 Placebo_loc - (real_in-phase)           0.0306 0.0184 1212   1.666  0.4557
 Placebo_loc - (real_out-of-phase)       0.0193 0.0182 1210   1.060  0.8272
 real_frontal - real_parietal            0.0151 0.0183 1212   0.824  0.9232
 real_frontal - (real_in-phase)          0.0644 0.0187 1201   3.441  0.0054
 real_frontal - (real_out-of-phase)      0.0531 0.0184 1212   2.892  0.0318
 real_parietal - (real_in-phase)         0.0493 0.0186 1208   2.658  0.0610
 real_parietal - (real_out-of-phase)     0.0380 0.0183 1212   2.074  0.2320
 (real_in-phase) - (real_out-of-phase)  -0.0113 0.0183 1212  -0.619  0.9721

Degrees-of-freedom method: kenward-roger 
P value adjustment: tukey method for comparing a family of 5 estimates
```


```
confint(my_model.compare, calc = c(n = ~.wgt.))
```


```
 Stim              emmean     SE   df   n lower.CL upper.CL
 Placebo_loc         1.04 0.0273 23.2 247    0.987     1.10
 real_frontal        1.08 0.0274 23.4 247    1.021     1.13
 real_parietal       1.06 0.0274 23.3 247    1.006     1.12
 real_in-phase       1.01 0.0275 23.5 247    0.956     1.07
 real_out-of-phase   1.02 0.0273 23.2 247    0.968     1.08

Degrees-of-freedom method: kenward-roger 
Confidence level used: 0.95
```


```
eff_size(my_model.compare, sigma = sigma(my_model), edf = 23)
```


```
 contrast                              effect.size     SE   df lower.CL upper.CL
 Placebo_loc - real_frontal                -0.1677 0.0937 23.2  -0.3614   0.0261
 Placebo_loc - real_parietal               -0.0929 0.0917 23.2  -0.2824   0.0966
 Placebo_loc - (real_in-phase)              0.1519 0.0939 23.2  -0.0422   0.3460
 Placebo_loc - (real_out-of-phase)          0.0956 0.0913 23.2  -0.0932   0.2845
 real_frontal - real_parietal               0.0748 0.0914 23.3  -0.1142   0.2638
 real_frontal - (real_in-phase)             0.3195 0.1041 23.4   0.1043   0.5348
 real_frontal - (real_out-of-phase)         0.2633 0.0990 23.2   0.0587   0.4679
 real_parietal - (real_in-phase)            0.2448 0.0989 23.3   0.0403   0.4492
 real_parietal - (real_out-of-phase)        0.1885 0.0950 23.2  -0.0080   0.3850
 (real_in-phase) - (real_out-of-phase)     -0.0563 0.0913 23.2  -0.2450   0.1324

sigma used for effect sizes: 0.2016 
Degrees-of-freedom method: inherited from kenward-roger when re-gridding 
Confidence level used: 0.95
```

### Note:

The model revealed that, when excluding subject 9004 from the
analysis, the change in accuracy in the participants was no longer
significantly different when receiving in-phase stimulation compared to
when they received the placebo.

### Normalized accuracy in the 3-back task


```
data_subset <- subset(df_muni, Task == '3back')
data_subset <- droplevels(data_subset)
levels(data_subset$Task)
```


```
[1] "3back"
```


```
m_acc_norm_3back <- lmer(formula = Acc_norm ~ Stim + (1 + (1|ID) + (1|Day)), data=data_subset)
summary(m_acc_norm_3back)
```


```
Linear mixed model fit by REML. t-tests use Satterthwaite's method ['lmerModLmerTest']
Formula: Acc_norm ~ Stim + (1 + (1 | ID) + (1 | Day))
   Data: data_subset

REML criterion at convergence: -200.5

Scaled residuals: 
    Min      1Q  Median      3Q     Max 
-3.3879 -0.6784 -0.0783  0.5570  3.3835 

Random effects:
 Groups   Name        Variance  Std.Dev.
 ID       (Intercept) 0.0036721 0.06060 
 Day      (Intercept) 0.0007325 0.02706 
 Residual             0.0475753 0.21812 
Number of obs: 1300, groups:  ID, 20; Day, 5

Fixed effects:
                        Estimate Std. Error         df t value Pr(>|t|)    
(Intercept)              1.00342    0.02267   24.04685  44.266  < 2e-16 ***
Stimreal_frontal         0.06674    0.01915 1273.32329   3.485 0.000509 ***
Stimreal_parietal       -0.03378    0.01921 1275.52368  -1.759 0.078874 .  
Stimreal_in-phase       -0.02914    0.01943 1265.64564  -1.499 0.134001    
Stimreal_out-of-phase    0.00591    0.01919 1275.02502   0.308 0.758131    
---
Signif. codes:  0 ‘***’ 0.001 ‘**’ 0.01 ‘*’ 0.05 ‘.’ 0.1 ‘ ’ 1

Correlation of Fixed Effects:
            (Intr) Stmrl_f Stmrl_p Stmr_-
Stmrl_frntl -0.421                       
Stimrl_prtl -0.422  0.500                
Stmrl_n-phs -0.421  0.484   0.488        
Stmrl_t-f-p -0.423  0.496   0.498   0.500
```


```
anova(m_acc_norm_3back)
```


```
Type III Analysis of Variance Table with Satterthwaite's method
     Sum Sq Mean Sq NumDF  DenDF F value    Pr(>F)    
Stim 1.6494 0.41234     4 1270.2  8.6671 6.683e-07 ***
---
Signif. codes:  0 ‘***’ 0.001 ‘**’ 0.01 ‘*’ 0.05 ‘.’ 0.1 ‘ ’ 1
```


```
eta_squared(m_acc_norm_3back)
```


```
# Effect Size for ANOVA (Type III)

Parameter | Eta2 (partial) |       95% CI
-----------------------------------------
Stim      |           0.03 | [0.01, 1.00]

- One-sided CIs: upper bound fixed at [1.00].
```


```
my_model <- m_acc_norm_3back
level_1 <- "Stim"

my_model.compare <- emmeans(my_model, level_1)
my_model.compare.pairs <- pairs(my_model.compare, adjust='tukey')
test(my_model.compare.pairs, side='two-sided')
```


```
 contrast                              estimate     SE   df t.ratio p.value
 Placebo_loc - real_frontal            -0.06674 0.0192 1273  -3.484  0.0046
 Placebo_loc - real_parietal            0.03378 0.0192 1276   1.757  0.3995
 Placebo_loc - (real_in-phase)          0.02914 0.0195 1266   1.493  0.5670
 Placebo_loc - (real_out-of-phase)     -0.00591 0.0192 1275  -0.308  0.9981
 real_frontal - real_parietal           0.10052 0.0192 1275   5.235  <.0001
 real_frontal - (real_in-phase)         0.09588 0.0197 1242   4.864  <.0001
 real_frontal - (real_out-of-phase)     0.06083 0.0193 1276   3.155  0.0142
 real_parietal - (real_in-phase)       -0.00464 0.0197 1249  -0.236  0.9993
 real_parietal - (real_out-of-phase)   -0.03969 0.0193 1276  -2.061  0.2377
 (real_in-phase) - (real_out-of-phase) -0.03505 0.0194 1275  -1.811  0.3678

Degrees-of-freedom method: kenward-roger 
P value adjustment: tukey method for comparing a family of 5 estimates
```


```
confint(my_model.compare, calc = c(n = ~.wgt.))
```


```
 Stim              emmean     SE   df   n lower.CL upper.CL
 Placebo_loc        1.003 0.0227 23.9 260    0.957     1.05
 real_frontal       1.070 0.0227 24.0 260    1.023     1.12
 real_parietal      0.970 0.0227 24.0 260    0.923     1.02
 real_in-phase      0.974 0.0229 24.2 260    0.927     1.02
 real_out-of-phase  1.009 0.0227 23.9 260    0.963     1.06

Degrees-of-freedom method: kenward-roger 
Confidence level used: 0.95
```


```
eff_size(my_model.compare, sigma = sigma(my_model), edf = 23)
```


```
 contrast                              effect.size     SE   df lower.CL upper.CL
 Placebo_loc - real_frontal                -0.3060 0.0987 23.9  -0.5098 -0.10217
 Placebo_loc - real_parietal                0.1549 0.0911 23.9  -0.0331  0.34285
 Placebo_loc - (real_in-phase)              0.1336 0.0916 23.9  -0.0555  0.32267
 Placebo_loc - (real_out-of-phase)         -0.0271 0.0881 23.9  -0.2090  0.15484
 real_frontal - real_parietal               0.4609 0.1112 24.0   0.2313  0.69040
 real_frontal - (real_in-phase)             0.4396 0.1112 24.0   0.2100  0.66912
 real_frontal - (real_out-of-phase)         0.2789 0.0975 23.9   0.0776  0.48014
 real_parietal - (real_in-phase)           -0.0213 0.0902 24.0  -0.2074  0.16488
 real_parietal - (real_out-of-phase)       -0.1820 0.0923 23.9  -0.3724  0.00849
 (real_in-phase) - (real_out-of-phase)     -0.1607 0.0919 23.9  -0.3503  0.02893

sigma used for effect sizes: 0.2181 
Degrees-of-freedom method: inherited from kenward-roger when re-gridding 
Confidence level used: 0.95
```

## Speed

Now, we present the same type of statistical analysis on the speed of
the participants. Please note for this comparison, we consider the speed
of correct trials only.


```
m0 <- lm(formula = Speed_corr ~ Stim*Task, data=df_muni)
summary(m0)
```


```
Call:
lm(formula = Speed_corr ~ Stim * Task, data = df_muni)

Residuals:
    Min      1Q  Median      3Q     Max 
-558.39 -158.89  -22.66  132.07 1055.84 

Coefficients:
                                Estimate Std. Error t value Pr(>|t|)    
(Intercept)                     996.7585    14.6629  67.978  < 2e-16 ***
Stimreal_frontal                 -3.6758    20.7364  -0.177 0.859315    
Stimreal_parietal                -4.8459    20.7364  -0.234 0.815245    
Stimreal_in-phase               -29.0983    20.7364  -1.403 0.160663    
Stimreal_out-of-phase            -3.0461    20.7364  -0.147 0.883226    
Task3back                        76.4531    20.7364   3.687 0.000232 ***
Stimreal_frontal:Task3back      -27.5272    29.3257  -0.939 0.347988    
Stimreal_parietal:Task3back     -20.4748    29.3257  -0.698 0.485123    
Stimreal_in-phase:Task3back      -0.9617    29.3257  -0.033 0.973841    
Stimreal_out-of-phase:Task3back -32.1259    29.3257  -1.095 0.273406    
---
Signif. codes:  0 ‘***’ 0.001 ‘**’ 0.01 ‘*’ 0.05 ‘.’ 0.1 ‘ ’ 1

Residual standard error: 236.4 on 2590 degrees of freedom
Multiple R-squared:  0.01837,   Adjusted R-squared:  0.01496 
F-statistic: 5.387 on 9 and 2590 DF,  p-value: 2.449e-07
```


```
anova(m0)
```


```
Analysis of Variance Table

Response: Speed_corr
            Df    Sum Sq Mean Sq F value    Pr(>F)    
Stim         4    235393   58848  1.0527    0.3784    
Task         1   2358380 2358380 42.1894 9.899e-11 ***
Stim:Task    4    116331   29083  0.5203    0.7209    
Residuals 2590 144780633   55900                      
---
Signif. codes:  0 ‘***’ 0.001 ‘**’ 0.01 ‘*’ 0.05 ‘.’ 0.1 ‘ ’ 1
```


### Note: This model shows a signifcant difference among the tasks. We will now include the blocks in the model and compare both models statistically.

### Linear model, no random effects


```
m1 <- lm(formula = Speed_corr ~ Stim*Task*Block, data=df_muni)
summary(m1)
```


```
Call:
lm(formula = Speed_corr ~ Stim * Task * Block, data = df_muni)

Residuals:
   Min     1Q Median     3Q    Max 
-555.3 -159.6  -20.4  131.9 1047.3 

Coefficients:
                                        Estimate Std. Error t value Pr(>|t|)    
(Intercept)                           1018.39521   31.05881  32.789   <2e-16 ***
Stimreal_frontal                       -10.51399   43.92379  -0.239   0.8108    
Stimreal_parietal                       32.92708   43.92379   0.750   0.4535    
Stimreal_in-phase                      -19.80037   43.92379  -0.451   0.6522    
Stimreal_out-of-phase                   13.65219   43.92379   0.311   0.7560    
Task3back                               77.05366   43.92379   1.754   0.0795 .  
Block                                   -3.09096    3.91304  -0.790   0.4297    
Stimreal_frontal:Task3back               6.91608   62.11762   0.111   0.9114    
Stimreal_parietal:Task3back            -44.38341   62.11762  -0.715   0.4750    
Stimreal_in-phase:Task3back             10.51354   62.11762   0.169   0.8656    
Stimreal_out-of-phase:Task3back        -49.50932   62.11762  -0.797   0.4255    
Stimreal_frontal:Block                   0.97688    5.53388   0.177   0.8599    
Stimreal_parietal:Block                 -5.39614    5.53388  -0.975   0.3296    
Stimreal_in-phase:Block                 -1.32828    5.53388  -0.240   0.8103    
Stimreal_out-of-phase:Block             -2.38547    5.53388  -0.431   0.6665    
Task3back:Block                         -0.08579    5.53388  -0.016   0.9876    
Stimreal_frontal:Task3back:Block        -4.92047    7.82608  -0.629   0.5296    
Stimreal_parietal:Task3back:Block        3.41551    7.82608   0.436   0.6626    
Stimreal_in-phase:Task3back:Block       -1.63932    7.82608  -0.209   0.8341    
Stimreal_out-of-phase:Task3back:Block    2.48335    7.82608   0.317   0.7510    
---
Signif. codes:  0 ‘***’ 0.001 ‘**’ 0.01 ‘*’ 0.05 ‘.’ 0.1 ‘ ’ 1

Residual standard error: 236.1 on 2580 degrees of freedom
Multiple R-squared:  0.02504,   Adjusted R-squared:  0.01786 
F-statistic: 3.488 on 19 and 2580 DF,  p-value: 4.788e-07
```


```
anova(m1)
```


```
Analysis of Variance Table

Response: Speed_corr
                  Df    Sum Sq Mean Sq F value    Pr(>F)    
Stim               4    235393   58848  1.0559    0.3768    
Task               1   2358380 2358380 42.3139 9.304e-11 ***
Block              1    847961  847961 15.2141 9.844e-05 ***
Stim:Task          4    116331   29083  0.5218    0.7197    
Stim:Block         4     53874   13469  0.2417    0.9148    
Task:Block         1       432     432  0.0078    0.9298    
Stim:Task:Block    4     81252   20313  0.3645    0.8341    
Residuals       2580 143797114   55735                      
---
Signif. codes:  0 ‘***’ 0.001 ‘**’ 0.01 ‘*’ 0.05 ‘.’ 0.1 ‘ ’ 1
```


#### Compare models


```
anova(m0, m1)
```


```
Analysis of Variance Table

Model 1: Speed_corr ~ Stim * Task
Model 2: Speed_corr ~ Stim * Task * Block
  Res.Df       RSS Df Sum of Sq      F  Pr(>F)  
1   2590 144780633                              
2   2580 143797114 10    983519 1.7646 0.06185 .
---
Signif. codes:  0 ‘***’ 0.001 ‘**’ 0.01 ‘*’ 0.05 ‘.’ 0.1 ‘ ’ 1
```


The amount of variance explained by the model is not statistically
larger than in in the simpler model (m0), so the inclusion of the blocks
is not justified. We will now assess models including random
effects.


```
m2 <- lmer(formula = Speed_corr ~ Stim*Task + (1 | ID), data=df_muni)
summary(m2)
```


```
Linear mixed model fit by REML. t-tests use Satterthwaite's method ['lmerModLmerTest']
Formula: Speed_corr ~ Stim * Task + (1 | ID)
   Data: df_muni

REML criterion at convergence: 33856.1

Scaled residuals: 
    Min      1Q  Median      3Q     Max 
-3.0738 -0.7041 -0.0916  0.5726  5.0906 

Random effects:
 Groups   Name        Variance Std.Dev.
 ID       (Intercept) 31088    176.3   
 Residual             26253    162.0   
Number of obs: 2600, groups:  ID, 20

Fixed effects:
                                 Estimate Std. Error        df t value Pr(>|t|)    
(Intercept)                      996.7585    40.6860   21.2708  24.499  < 2e-16 ***
Stimreal_frontal                  -3.6758    14.2107 2571.0000  -0.259   0.7959    
Stimreal_parietal                 -4.8459    14.2107 2571.0000  -0.341   0.7331    
Stimreal_in-phase                -29.0983    14.2107 2571.0000  -2.048   0.0407 *  
Stimreal_out-of-phase             -3.0461    14.2107 2571.0000  -0.214   0.8303    
Task3back                         76.4531    14.2107 2571.0000   5.380 8.12e-08 ***
Stimreal_frontal:Task3back       -27.5272    20.0969 2571.0000  -1.370   0.1709    
Stimreal_parietal:Task3back      -20.4748    20.0969 2571.0000  -1.019   0.3084    
Stimreal_in-phase:Task3back       -0.9617    20.0969 2571.0000  -0.048   0.9618    
Stimreal_out-of-phase:Task3back  -32.1259    20.0969 2571.0000  -1.599   0.1100    
---
Signif. codes:  0 ‘***’ 0.001 ‘**’ 0.01 ‘*’ 0.05 ‘.’ 0.1 ‘ ’ 1

Correlation of Fixed Effects:
            (Intr) Stmrl_f Stmrl_p Stmr_- Stm_-- Tsk3bc Stmrl_f:T3 Stmrl_p:T3 S_-:T3
Stmrl_frntl -0.175                                                                  
Stimrl_prtl -0.175  0.500                                                           
Stmrl_n-phs -0.175  0.500   0.500                                                   
Stmrl_t-f-p -0.175  0.500   0.500   0.500                                           
Task3back   -0.175  0.500   0.500   0.500  0.500                                    
Stmrl_fr:T3  0.123 -0.707  -0.354  -0.354 -0.354 -0.707                             
Stmrl_pr:T3  0.123 -0.354  -0.707  -0.354 -0.354 -0.707  0.500                      
Stmrl_n-:T3  0.123 -0.354  -0.354  -0.707 -0.354 -0.707  0.500      0.500           
Stmrl_--:T3  0.123 -0.354  -0.354  -0.354 -0.707 -0.707  0.500      0.500      0.500
```


```
anova(m2)
```


```
Type III Analysis of Variance Table with Satterthwaite's method
           Sum Sq Mean Sq NumDF DenDF F value  Pr(>F)    
Stim       235393   58848     4  2571  2.2416 0.06225 .  
Task      2358380 2358380     1  2571 89.8339 < 2e-16 ***
Stim:Task  116331   29083     4  2571  1.1078 0.35103    
---
Signif. codes:  0 ‘***’ 0.001 ‘**’ 0.01 ‘*’ 0.05 ‘.’ 0.1 ‘ ’ 1
```


```
AIC(m0, m2)
```


```
BIC(m0, m2)
```


The model including random intercepts is better than the regular
linear model in this case.


```
m3 <- lmer(formula = Speed_corr ~ Stim*Task*Block + (1 | ID), data=df_muni)
summary(m3)
```


```
Linear mixed model fit by REML. t-tests use Satterthwaite's method ['lmerModLmerTest']
Formula: Speed_corr ~ Stim * Task * Block + (1 | ID)
   Data: df_muni

REML criterion at convergence: 33780.4

Scaled residuals: 
    Min      1Q  Median      3Q     Max 
-2.9724 -0.6924 -0.0972  0.5924  4.8548 

Random effects:
 Groups   Name        Variance Std.Dev.
 ID       (Intercept) 31090    176.3   
 Residual             25971    161.2   
Number of obs: 2600, groups:  ID, 20

Fixed effects:
                                        Estimate Std. Error         df t value Pr(>|t|)    
(Intercept)                           1018.39521   44.76590   31.15672  22.749   <2e-16 ***
Stimreal_frontal                       -10.51399   29.98335 2561.00000  -0.351   0.7259    
Stimreal_parietal                       32.92708   29.98335 2561.00000   1.098   0.2722    
Stimreal_in-phase                      -19.80037   29.98335 2561.00000  -0.660   0.5091    
Stimreal_out-of-phase                   13.65219   29.98335 2561.00000   0.455   0.6489    
Task3back                               77.05366   29.98335 2561.00000   2.570   0.0102 *  
Block                                   -3.09096    2.67113 2561.00000  -1.157   0.2473    
Stimreal_frontal:Task3back               6.91608   42.40286 2561.00000   0.163   0.8704    
Stimreal_parietal:Task3back            -44.38341   42.40286 2561.00000  -1.047   0.2953    
Stimreal_in-phase:Task3back             10.51354   42.40286 2561.00000   0.248   0.8042    
Stimreal_out-of-phase:Task3back        -49.50932   42.40286 2561.00000  -1.168   0.2431    
Stimreal_frontal:Block                   0.97688    3.77755 2561.00000   0.259   0.7960    
Stimreal_parietal:Block                 -5.39614    3.77755 2561.00000  -1.428   0.1533    
Stimreal_in-phase:Block                 -1.32828    3.77755 2561.00000  -0.352   0.7251    
Stimreal_out-of-phase:Block             -2.38547    3.77755 2561.00000  -0.631   0.5278    
Task3back:Block                         -0.08579    3.77755 2561.00000  -0.023   0.9819    
Stimreal_frontal:Task3back:Block        -4.92047    5.34226 2561.00000  -0.921   0.3571    
Stimreal_parietal:Task3back:Block        3.41551    5.34226 2561.00000   0.639   0.5227    
Stimreal_in-phase:Task3back:Block       -1.63932    5.34226 2561.00000  -0.307   0.7590    
Stimreal_out-of-phase:Task3back:Block    2.48335    5.34226 2561.00000   0.465   0.6421    
---
Signif. codes:  0 ‘***’ 0.001 ‘**’ 0.01 ‘*’ 0.05 ‘.’ 0.1 ‘ ’ 1
```


```
Correlation matrix not shown by default, as p = 20 > 12.
Use print(x, correlation=TRUE)  or
    vcov(x)        if you need it
```


```
anova(m3)
```


```
Type III Analysis of Variance Table with Satterthwaite's method
                Sum Sq Mean Sq NumDF DenDF F value    Pr(>F)    
Stim             46605   11651     4  2561  0.4486    0.7735    
Task            550973  550973     1  2561 21.2148 4.307e-06 ***
Block           847961  847961     1  2561 32.6501 1.231e-08 ***
Stim:Task        98514   24629     4  2561  0.9483    0.4349    
Stim:Block       53874   13469     4  2561  0.5186    0.7221    
Task:Block         432     432     1  2561  0.0166    0.8973    
Stim:Task:Block  81252   20313     4  2561  0.7821    0.5367    
---
Signif. codes:  0 ‘***’ 0.001 ‘**’ 0.01 ‘*’ 0.05 ‘.’ 0.1 ‘ ’ 1
```


```
anova(m2, m3)
```


```
refitting model(s) with ML (instead of REML)
```


```
Data: df_muni
Models:
m2: Speed_corr ~ Stim * Task + (1 | ID)
m3: Speed_corr ~ Stim * Task * Block + (1 | ID)
   npar   AIC   BIC logLik deviance  Chisq Df Pr(>Chisq)    
m2   12 33950 34020 -16963    33926                         
m3   22 33932 34061 -16944    33888 37.871 10  3.996e-05 ***
---
Signif. codes:  0 ‘***’ 0.001 ‘**’ 0.01 ‘*’ 0.05 ‘.’ 0.1 ‘ ’ 1
```


In this case, the model comparison seems to yield contradictory
results, as according to the AIC, the second model is better, while
according to the BIC, the first model is better. The BIC is considered
to be a more robust measure, as it takes into account the sample sizes
and is thought to be a better choice for model selection, while AIC is
considered better in selecting a model to make predictions (see
Chakrabarti and Ghosh, 2011). According to the BIC, the addition of the
blocks worsens the model, so we will leave them out.

#### Random intercept and random slope per subject


```
m4 <- lmer(formula = Speed_corr ~ Stim*Task*Block + (1 + Block|ID), data=df_muni)
summary(m4)
```


```
Linear mixed model fit by REML. t-tests use Satterthwaite's method ['lmerModLmerTest']
Formula: Speed_corr ~ Stim * Task * Block + (1 + Block | ID)
   Data: df_muni

REML criterion at convergence: 33763.3

Scaled residuals: 
    Min      1Q  Median      3Q     Max 
-2.9891 -0.6879 -0.1052  0.5824  4.7954 

Random effects:
 Groups   Name        Variance Std.Dev. Corr
 ID       (Intercept) 28736.13 169.517      
          Block          27.16   5.212  0.08
 Residual             25604.37 160.014      
Number of obs: 2600, groups:  ID, 20

Fixed effects:
                                        Estimate Std. Error         df t value Pr(>|t|)    
(Intercept)                           1018.39521   43.35849   30.59124  23.488   <2e-16 ***
Stimreal_frontal                       -10.51399   29.77088 2542.00099  -0.353   0.7240    
Stimreal_parietal                       32.92708   29.77088 2542.00099   1.106   0.2688    
Stimreal_in-phase                      -19.80037   29.77088 2542.00099  -0.665   0.5061    
Stimreal_out-of-phase                   13.65219   29.77088 2542.00099   0.459   0.6466    
Task3back                               77.05366   29.77088 2542.00098   2.588   0.0097 ** 
Block                                   -3.09096    2.89695  294.12519  -1.067   0.2869    
Stimreal_frontal:Task3back               6.91608   42.10238 2542.00098   0.164   0.8695    
Stimreal_parietal:Task3back            -44.38341   42.10238 2542.00098  -1.054   0.2919    
Stimreal_in-phase:Task3back             10.51354   42.10238 2542.00098   0.250   0.8028    
Stimreal_out-of-phase:Task3back        -49.50932   42.10238 2542.00098  -1.176   0.2397    
Stimreal_frontal:Block                   0.97688    3.75078 2542.00099   0.260   0.7945    
Stimreal_parietal:Block                 -5.39614    3.75078 2542.00099  -1.439   0.1504    
Stimreal_in-phase:Block                 -1.32828    3.75078 2542.00099  -0.354   0.7233    
Stimreal_out-of-phase:Block             -2.38547    3.75078 2542.00099  -0.636   0.5248    
Task3back:Block                         -0.08579    3.75078 2542.00098  -0.023   0.9818    
Stimreal_frontal:Task3back:Block        -4.92047    5.30440 2542.00098  -0.928   0.3537    
Stimreal_parietal:Task3back:Block        3.41551    5.30440 2542.00098   0.644   0.5197    
Stimreal_in-phase:Task3back:Block       -1.63932    5.30440 2542.00098  -0.309   0.7573    
Stimreal_out-of-phase:Task3back:Block    2.48335    5.30440 2542.00098   0.468   0.6397    
---
Signif. codes:  0 ‘***’ 0.001 ‘**’ 0.01 ‘*’ 0.05 ‘.’ 0.1 ‘ ’ 1
```


```
Correlation matrix not shown by default, as p = 20 > 12.
Use print(x, correlation=TRUE)  or
    vcov(x)        if you need it
```


```
anova(m4)
```


```
Type III Analysis of Variance Table with Satterthwaite's method
                Sum Sq Mean Sq NumDF DenDF F value    Pr(>F)    
Stim             46605   11651     4  2542  0.4550  0.768776    
Task            550973  550973     1  2542 21.5187 3.682e-06 ***
Block           289330  289330     1    19 11.3000  0.003277 ** 
Stim:Task        98514   24629     4  2542  0.9619  0.427227    
Stim:Block       53874   13469     4  2542  0.5260  0.716625    
Task:Block         432     432     1  2542  0.0169  0.896616    
Stim:Task:Block  81252   20313     4  2542  0.7933  0.529365    
---
Signif. codes:  0 ‘***’ 0.001 ‘**’ 0.01 ‘*’ 0.05 ‘.’ 0.1 ‘ ’ 1
```

#### Compare models, even though this last one did not converge


```
anova(m2, m4)
```


```
refitting model(s) with ML (instead of REML)
```


```
Data: df_muni
Models:
m2: Speed_corr ~ Stim * Task + (1 | ID)
m4: Speed_corr ~ Stim * Task * Block + (1 + Block | ID)
   npar   AIC   BIC logLik deviance  Chisq Df Pr(>Chisq)    
m2   12 33950 34020 -16963    33926                         
m4   24 33919 34060 -16936    33871 54.185 12  2.532e-07 ***
---
Signif. codes:  0 ‘***’ 0.001 ‘**’ 0.01 ‘*’ 0.05 ‘.’ 0.1 ‘ ’ 1
```


Based on the BIC, adding a random slope worsens the amount of
variance explained by the model including only a random intercept.


```
m5 <- lmer(formula = Speed_corr ~ Stim*Task + (1 + (1|ID) + (1|Day)), data=df_muni)
```


```
Warning: Model failed to converge with max|grad| = 0.0194515 (tol = 0.002, component 1)
```


```
summary(m5)
```


```
Linear mixed model fit by REML. t-tests use Satterthwaite's method ['lmerModLmerTest']
Formula: Speed_corr ~ Stim * Task + (1 + (1 | ID) + (1 | Day))
   Data: df_muni

REML criterion at convergence: 33834.7

Scaled residuals: 
    Min      1Q  Median      3Q     Max 
-2.9471 -0.6999 -0.0945  0.5706  5.1063 

Random effects:
 Groups   Name        Variance Std.Dev.
 ID       (Intercept) 31119.0  176.41  
 Day      (Intercept)   390.8   19.77  
 Residual             25947.1  161.08  
Number of obs: 2600, groups:  ID, 20; Day, 5

Fixed effects:
                                 Estimate Std. Error        df t value Pr(>|t|)    
(Intercept)                      994.1528    41.6429   22.9244  23.873  < 2e-16 ***
Stimreal_frontal                  -5.2980    14.1361 2567.7505  -0.375    0.708    
Stimreal_parietal                 -2.8791    14.1595 2569.4096  -0.203    0.839    
Stimreal_in-phase                -20.4056    14.2526 2569.9245  -1.432    0.152    
Stimreal_out-of-phase              0.9447    14.1519 2568.9539   0.067    0.947    
Task3back                         76.4531    14.1277 2566.9638   5.412 6.83e-08 ***
Stimreal_frontal:Task3back       -27.5272    19.9796 2566.9638  -1.378    0.168    
Stimreal_parietal:Task3back      -20.4748    19.9796 2566.9638  -1.025    0.306    
Stimreal_in-phase:Task3back       -0.9617    19.9796 2566.9638  -0.048    0.962    
Stimreal_out-of-phase:Task3back  -32.1259    19.9796 2566.9638  -1.608    0.108    
---
Signif. codes:  0 ‘***’ 0.001 ‘**’ 0.01 ‘*’ 0.05 ‘.’ 0.1 ‘ ’ 1

Correlation of Fixed Effects:
            (Intr) Stmrl_f Stmrl_p Stmr_- Stm_-- Tsk3bc Stmrl_f:T3 Stmrl_p:T3 S_-:T3
Stmrl_frntl -0.169                                                                  
Stimrl_prtl -0.170  0.500                                                           
Stmrl_n-phs -0.169  0.491   0.493                                                   
Stmrl_t-f-p -0.170  0.498   0.499   0.500                                           
Task3back   -0.170  0.500   0.499   0.496  0.499                                    
Stmrl_fr:T3  0.120 -0.707  -0.353  -0.350 -0.353 -0.707                             
Stmrl_pr:T3  0.120 -0.353  -0.706  -0.350 -0.353 -0.707  0.500                      
Stmrl_n-:T3  0.120 -0.353  -0.353  -0.701 -0.353 -0.707  0.500      0.500           
Stmrl_--:T3  0.120 -0.353  -0.353  -0.350 -0.706 -0.707  0.500      0.500      0.500
optimizer (nloptwrap) convergence code: 0 (OK)
Model failed to converge with max|grad| = 0.0194515 (tol = 0.002, component 1)
```


```
anova(m5)
```


```
Type III Analysis of Variance Table with Satterthwaite's method
           Sum Sq Mean Sq NumDF  DenDF F value Pr(>F)    
Stim       139160   34790     4 2549.8  1.3408 0.2523    
Task      2358380 2358380     1 2567.0 90.8920 <2e-16 ***
Stim:Task  116331   29083     4 2567.0  1.1209 0.3448    
---
Signif. codes:  0 ‘***’ 0.001 ‘**’ 0.01 ‘*’ 0.05 ‘.’ 0.1 ‘ ’ 1
```


```
anova(m2, m5)
```


```
refitting model(s) with ML (instead of REML)
```


```
Data: df_muni
Models:
m2: Speed_corr ~ Stim * Task + (1 | ID)
m5: Speed_corr ~ Stim * Task + (1 + (1 | ID) + (1 | Day))
   npar   AIC   BIC logLik deviance  Chisq Df Pr(>Chisq)    
m2   12 33950 34020 -16963    33926                         
m5   13 33930 34006 -16952    33904 21.427  1  3.675e-06 ***
---
Signif. codes:  0 ‘***’ 0.001 ‘**’ 0.01 ‘*’ 0.05 ‘.’ 0.1 ‘ ’ 1
```

### Model choice

Based on the BIC, which is thought to be a more robust parameter for
model selection than AIC (as mentioned previously), the best model is
m5, including random intercepts for both subjects and days. However, m5
did not converge, so we will test whether the addition of the blocks in
the model improves the estimate.


```
m6 <- lmer(formula = Speed_corr ~ Stim*Task*Block + (1 + (1|ID) + (1|Day)), data=df_muni)
summary(m6)
```


```
Linear mixed model fit by REML. t-tests use Satterthwaite's method ['lmerModLmerTest']
Formula: Speed_corr ~ Stim * Task * Block + (1 + (1 | ID) + (1 | Day))
   Data: df_muni

REML criterion at convergence: 33758.6

Scaled residuals: 
    Min      1Q  Median      3Q     Max 
-2.9821 -0.6931 -0.0892  0.5820  4.8696 

Random effects:
 Groups   Name        Variance Std.Dev.
 ID       (Intercept) 31092.4  176.33  
 Day      (Intercept)   395.5   19.89  
 Residual             25663.7  160.20  
Number of obs: 2600, groups:  ID, 20; Day, 5

Fixed effects:
                                        Estimate Std. Error         df t value Pr(>|t|)    
(Intercept)                           1015.78260   45.58650   32.99276  22.283  < 2e-16 ***
Stimreal_frontal                       -12.14074   29.80927 2557.15540  -0.407  0.68384    
Stimreal_parietal                       34.89880   29.82030 2557.60579   1.170  0.24199    
Stimreal_in-phase                      -11.08382   29.86423 2559.22914  -0.371  0.71056    
Stimreal_out-of-phase                   17.65373   29.81674 2557.46787   0.592  0.55385    
Task3back                               77.05366   29.80535 2556.97670   2.585  0.00979 ** 
Block                                   -3.09096    2.65527 2556.97670  -1.164  0.24450    
Stimreal_frontal:Task3back               6.91608   42.15113 2556.97670   0.164  0.86968    
Stimreal_parietal:Task3back            -44.38341   42.15113 2556.97670  -1.053  0.29246    
Stimreal_in-phase:Task3back             10.51354   42.15113 2556.97670   0.249  0.80305    
Stimreal_out-of-phase:Task3back        -49.50932   42.15113 2556.97670  -1.175  0.24028    
Stimreal_frontal:Block                   0.97688    3.75512 2556.97670   0.260  0.79477    
Stimreal_parietal:Block                 -5.39614    3.75512 2556.97670  -1.437  0.15084    
Stimreal_in-phase:Block                 -1.32828    3.75512 2556.97670  -0.354  0.72357    
Stimreal_out-of-phase:Block             -2.38547    3.75512 2556.97670  -0.635  0.52532    
Task3back:Block                         -0.08579    3.75512 2556.97670  -0.023  0.98177    
Stimreal_frontal:Task3back:Block        -4.92047    5.31054 2556.97671  -0.927  0.35425    
Stimreal_parietal:Task3back:Block        3.41551    5.31054 2556.97670   0.643  0.52018    
Stimreal_in-phase:Task3back:Block       -1.63932    5.31054 2556.97670  -0.309  0.75758    
Stimreal_out-of-phase:Task3back:Block    2.48335    5.31054 2556.97671   0.468  0.64009    
---
Signif. codes:  0 ‘***’ 0.001 ‘**’ 0.01 ‘*’ 0.05 ‘.’ 0.1 ‘ ’ 1
```


```
Correlation matrix not shown by default, as p = 20 > 12.
Use print(x, correlation=TRUE)  or
    vcov(x)        if you need it
```


```
anova(m6)
```


```
Type III Analysis of Variance Table with Satterthwaite's method
                Sum Sq Mean Sq NumDF  DenDF F value    Pr(>F)    
Stim             35182    8795     4 2558.5  0.3427    0.8492    
Task            550973  550973     1 2557.0 21.4689 3.776e-06 ***
Block           847961  847961     1 2557.0 33.0412 1.009e-08 ***
Stim:Task        98514   24629     4 2557.0  0.9597    0.4285    
Stim:Block       53874   13469     4 2557.0  0.5248    0.7175    
Task:Block         432     432     1 2557.0  0.0168    0.8967    
Stim:Task:Block  81252   20313     4 2557.0  0.7915    0.5306    
---
Signif. codes:  0 ‘***’ 0.001 ‘**’ 0.01 ‘*’ 0.05 ‘.’ 0.1 ‘ ’ 1
```


```
anova(m2, m6)
```


```
refitting model(s) with ML (instead of REML)
```


```
Data: df_muni
Models:
m2: Speed_corr ~ Stim * Task + (1 | ID)
m6: Speed_corr ~ Stim * Task * Block + (1 + (1 | ID) + (1 | Day))
   npar   AIC   BIC logLik deviance  Chisq Df Pr(>Chisq)    
m2   12 33950 34020 -16963    33926                         
m6   23 33912 34047 -16933    33866 59.749 11  1.032e-08 ***
---
Signif. codes:  0 ‘***’ 0.001 ‘**’ 0.01 ‘*’ 0.05 ‘.’ 0.1 ‘ ’ 1
```

### Model choice

The model including random intercepts for both subjects and days
significantly worsens the amount of variance explained by the model, so
we will keep m2, including only random intercepts for subjects.


```
m_speed <- lmer(formula = Speed_corr ~ Stim*Task + (1 | ID), data=df_muni)
summary(m_speed)
```


```
Linear mixed model fit by REML. t-tests use Satterthwaite's method ['lmerModLmerTest']
Formula: Speed_corr ~ Stim * Task + (1 | ID)
   Data: df_muni

REML criterion at convergence: 33856.1

Scaled residuals: 
    Min      1Q  Median      3Q     Max 
-3.0738 -0.7041 -0.0916  0.5726  5.0906 

Random effects:
 Groups   Name        Variance Std.Dev.
 ID       (Intercept) 31088    176.3   
 Residual             26253    162.0   
Number of obs: 2600, groups:  ID, 20

Fixed effects:
                                 Estimate Std. Error        df t value Pr(>|t|)    
(Intercept)                      996.7585    40.6860   21.2708  24.499  < 2e-16 ***
Stimreal_frontal                  -3.6758    14.2107 2571.0000  -0.259   0.7959    
Stimreal_parietal                 -4.8459    14.2107 2571.0000  -0.341   0.7331    
Stimreal_in-phase                -29.0983    14.2107 2571.0000  -2.048   0.0407 *  
Stimreal_out-of-phase             -3.0461    14.2107 2571.0000  -0.214   0.8303    
Task3back                         76.4531    14.2107 2571.0000   5.380 8.12e-08 ***
Stimreal_frontal:Task3back       -27.5272    20.0969 2571.0000  -1.370   0.1709    
Stimreal_parietal:Task3back      -20.4748    20.0969 2571.0000  -1.019   0.3084    
Stimreal_in-phase:Task3back       -0.9617    20.0969 2571.0000  -0.048   0.9618    
Stimreal_out-of-phase:Task3back  -32.1259    20.0969 2571.0000  -1.599   0.1100    
---
Signif. codes:  0 ‘***’ 0.001 ‘**’ 0.01 ‘*’ 0.05 ‘.’ 0.1 ‘ ’ 1

Correlation of Fixed Effects:
            (Intr) Stmrl_f Stmrl_p Stmr_- Stm_-- Tsk3bc Stmrl_f:T3 Stmrl_p:T3 S_-:T3
Stmrl_frntl -0.175                                                                  
Stimrl_prtl -0.175  0.500                                                           
Stmrl_n-phs -0.175  0.500   0.500                                                   
Stmrl_t-f-p -0.175  0.500   0.500   0.500                                           
Task3back   -0.175  0.500   0.500   0.500  0.500                                    
Stmrl_fr:T3  0.123 -0.707  -0.354  -0.354 -0.354 -0.707                             
Stmrl_pr:T3  0.123 -0.354  -0.707  -0.354 -0.354 -0.707  0.500                      
Stmrl_n-:T3  0.123 -0.354  -0.354  -0.707 -0.354 -0.707  0.500      0.500           
Stmrl_--:T3  0.123 -0.354  -0.354  -0.354 -0.707 -0.707  0.500      0.500      0.500
```


```
anova(m_speed)
```


```
Type III Analysis of Variance Table with Satterthwaite's method
           Sum Sq Mean Sq NumDF DenDF F value  Pr(>F)    
Stim       235393   58848     4  2571  2.2416 0.06225 .  
Task      2358380 2358380     1  2571 89.8339 < 2e-16 ***
Stim:Task  116331   29083     4  2571  1.1078 0.35103    
---
Signif. codes:  0 ‘***’ 0.001 ‘**’ 0.01 ‘*’ 0.05 ‘.’ 0.1 ‘ ’ 1
```


```
eta_squared(m_speed)
```


```
# Effect Size for ANOVA (Type III)

Parameter | Eta2 (partial) |       95% CI
-----------------------------------------
Stim      |       3.48e-03 | [0.00, 1.00]
Task      |           0.03 | [0.02, 1.00]
Stim:Task |       1.72e-03 | [0.00, 1.00]

- One-sided CIs: upper bound fixed at [1.00].
```

### Note:

There is a significant difference between the tasks. We will make
models for each task separately.

### Speed in the 2-back task


```
data_subset <- subset(df_muni, Task == '2back')
data_subset <- droplevels(data_subset)
levels(data_subset$Task)
```


```
[1] "2back"
```


```
m_speed_2back <- lmer(formula = Speed_corr ~ Stim + (1 | ID), data=data_subset)
summary(m_speed_2back)
```


```
Linear mixed model fit by REML. t-tests use Satterthwaite's method ['lmerModLmerTest']
Formula: Speed_corr ~ Stim + (1 | ID)
   Data: data_subset

REML criterion at convergence: 16781.6

Scaled residuals: 
    Min      1Q  Median      3Q     Max 
-3.2818 -0.7088 -0.1202  0.5989  5.2888 

Random effects:
 Groups   Name        Variance Std.Dev.
 ID       (Intercept) 37486    193.6   
 Residual             22712    150.7   
Number of obs: 1300, groups:  ID, 20

Fixed effects:
                      Estimate Std. Error       df t value Pr(>|t|)    
(Intercept)            996.758     44.291   20.429  22.505 6.79e-16 ***
Stimreal_frontal        -3.676     13.218 1276.000  -0.278   0.7810    
Stimreal_parietal       -4.846     13.218 1276.000  -0.367   0.7140    
Stimreal_in-phase      -29.098     13.218 1276.000  -2.201   0.0279 *  
Stimreal_out-of-phase   -3.046     13.218 1276.000  -0.230   0.8178    
---
Signif. codes:  0 ‘***’ 0.001 ‘**’ 0.01 ‘*’ 0.05 ‘.’ 0.1 ‘ ’ 1

Correlation of Fixed Effects:
            (Intr) Stmrl_f Stmrl_p Stmr_-
Stmrl_frntl -0.149                       
Stimrl_prtl -0.149  0.500                
Stmrl_n-phs -0.149  0.500   0.500        
Stmrl_t-f-p -0.149  0.500   0.500   0.500
```


```
anova(m_speed_2back)
```


```
Type III Analysis of Variance Table with Satterthwaite's method
     Sum Sq Mean Sq NumDF DenDF F value Pr(>F)
Stim 146182   36546     4  1276  1.6091 0.1696
```


```
eta_squared(m_speed_2back)
```


```
# Effect Size for ANOVA (Type III)

Parameter | Eta2 (partial) |       95% CI
-----------------------------------------
Stim      |       5.02e-03 | [0.00, 1.00]

- One-sided CIs: upper bound fixed at [1.00].
```

### Note:

There is no evidence for a significant effect of stimulation.

### Speed in the 3-back task


```
data_subset <- subset(df_muni, Task == '3back')
data_subset <- droplevels(data_subset)
levels(data_subset$Task)
```


```
[1] "3back"
```


```
m_speed_3back <- lmer(formula = Speed_corr ~ Stim + (1 | ID), data=data_subset)
summary(m_speed_3back)
```


```
Linear mixed model fit by REML. t-tests use Satterthwaite's method ['lmerModLmerTest']
Formula: Speed_corr ~ Stim + (1 | ID)
   Data: data_subset

REML criterion at convergence: 17010.2

Scaled residuals: 
    Min      1Q  Median      3Q     Max 
-2.5732 -0.7275 -0.0618  0.5702  4.4457 

Random effects:
 Groups   Name        Variance Std.Dev.
 ID       (Intercept) 27308    165.3   
 Residual             27295    165.2   
Number of obs: 1300, groups:  ID, 20

Fixed effects:
                      Estimate Std. Error      df t value Pr(>|t|)    
(Intercept)            1073.21      38.35   21.37  27.988   <2e-16 ***
Stimreal_frontal        -31.20      14.49 1276.00  -2.153   0.0315 *  
Stimreal_parietal       -25.32      14.49 1276.00  -1.747   0.0808 .  
Stimreal_in-phase       -30.06      14.49 1276.00  -2.075   0.0382 *  
Stimreal_out-of-phase   -35.17      14.49 1276.00  -2.427   0.0153 *  
---
Signif. codes:  0 ‘***’ 0.001 ‘**’ 0.01 ‘*’ 0.05 ‘.’ 0.1 ‘ ’ 1

Correlation of Fixed Effects:
            (Intr) Stmrl_f Stmrl_p Stmr_-
Stmrl_frntl -0.189                       
Stimrl_prtl -0.189  0.500                
Stmrl_n-phs -0.189  0.500   0.500        
Stmrl_t-f-p -0.189  0.500   0.500   0.500
```


```
anova(m_speed_3back)
```


```
Type III Analysis of Variance Table with Satterthwaite's method
     Sum Sq Mean Sq NumDF DenDF F value Pr(>F)
Stim 205543   51386     4  1276  1.8826 0.1111
```


```
eta_squared(m_speed_3back)
```


```
# Effect Size for ANOVA (Type III)

Parameter | Eta2 (partial) |       95% CI
-----------------------------------------
Stim      |       5.87e-03 | [0.00, 1.00]

- One-sided CIs: upper bound fixed at [1.00].
```

### Note:

There is no evidence for a significant effect of stimulation on the
speed of the 3-back task.

## Normalized Speed


```
m0 <- lm(formula = Speed_norm ~ Stim*Task, data=df_muni)
summary(m0)
```


```
Call:
lm(formula = Speed_norm ~ Stim * Task, data = df_muni)

Residuals:
     Min       1Q   Median       3Q      Max 
-0.55665 -0.14478 -0.01473  0.10802  0.90716 

Coefficients:
                                 Estimate Std. Error t value Pr(>|t|)    
(Intercept)                      0.929370   0.012555  74.026  < 2e-16 ***
Stimreal_frontal                 0.028304   0.017755   1.594 0.111022    
Stimreal_parietal               -0.009177   0.017755  -0.517 0.605309    
Stimreal_in-phase                0.004663   0.017755   0.263 0.792859    
Stimreal_out-of-phase           -0.019319   0.017755  -1.088 0.276664    
Task3back                        0.086277   0.017755   4.859 1.25e-06 ***
Stimreal_frontal:Task3back      -0.086572   0.025109  -3.448 0.000574 ***
Stimreal_parietal:Task3back     -0.049202   0.025109  -1.960 0.050160 .  
Stimreal_in-phase:Task3back     -0.057769   0.025109  -2.301 0.021488 *  
Stimreal_out-of-phase:Task3back  0.021496   0.025109   0.856 0.392031    
---
Signif. codes:  0 ‘***’ 0.001 ‘**’ 0.01 ‘*’ 0.05 ‘.’ 0.1 ‘ ’ 1

Residual standard error: 0.2024 on 2590 degrees of freedom
Multiple R-squared:  0.02859,   Adjusted R-squared:  0.02521 
F-statistic: 8.469 on 9 and 2590 DF,  p-value: 1.429e-12
```


```
anova(m0)
```


```
Analysis of Variance Table

Response: Speed_norm
            Df  Sum Sq Mean Sq F value    Pr(>F)    
Stim         4   0.362 0.09040  2.2059   0.06598 .  
Task         1   1.749 1.74868 42.6700 7.773e-11 ***
Stim:Task    4   1.013 0.25333  6.1816 6.002e-05 ***
Residuals 2590 106.142 0.04098                      
---
Signif. codes:  0 ‘***’ 0.001 ‘**’ 0.01 ‘*’ 0.05 ‘.’ 0.1 ‘ ’ 1
```


```
m1 <- lm(formula = Speed_norm ~ Stim*Task*Block, data=df_muni)
summary(m1)
```


```
Call:
lm(formula = Speed_norm ~ Stim * Task * Block, data = df_muni)

Residuals:
    Min      1Q  Median      3Q     Max 
-0.5420 -0.1420 -0.0101  0.1114  0.9042 

Coefficients:
                                        Estimate Std. Error t value Pr(>|t|)    
(Intercept)                            0.9464264  0.0266112  35.565   <2e-16 ***
Stimreal_frontal                       0.0198278  0.0376340   0.527   0.5983    
Stimreal_parietal                      0.0163632  0.0376340   0.435   0.6637    
Stimreal_in-phase                      0.0103790  0.0376340   0.276   0.7827    
Stimreal_out-of-phase                 -0.0002971  0.0376340  -0.008   0.9937    
Task3back                              0.0786683  0.0376340   2.090   0.0367 *  
Block                                 -0.0024366  0.0033527  -0.727   0.4674    
Stimreal_frontal:Task3back            -0.0484517  0.0532224  -0.910   0.3627    
Stimreal_parietal:Task3back           -0.0593184  0.0532224  -1.115   0.2652    
Stimreal_in-phase:Task3back           -0.0393914  0.0532224  -0.740   0.4593    
Stimreal_out-of-phase:Task3back        0.0140227  0.0532224   0.263   0.7922    
Stimreal_frontal:Block                 0.0012109  0.0047414   0.255   0.7984    
Stimreal_parietal:Block               -0.0036485  0.0047414  -0.770   0.4417    
Stimreal_in-phase:Block               -0.0008166  0.0047414  -0.172   0.8633    
Stimreal_out-of-phase:Block           -0.0027174  0.0047414  -0.573   0.5666    
Task3back:Block                        0.0010870  0.0047414   0.229   0.8187    
Stimreal_frontal:Task3back:Block      -0.0054457  0.0067054  -0.812   0.4168    
Stimreal_parietal:Task3back:Block      0.0014452  0.0067054   0.216   0.8294    
Stimreal_in-phase:Task3back:Block     -0.0026254  0.0067054  -0.392   0.6954    
Stimreal_out-of-phase:Task3back:Block  0.0010676  0.0067054   0.159   0.8735    
---
Signif. codes:  0 ‘***’ 0.001 ‘**’ 0.01 ‘*’ 0.05 ‘.’ 0.1 ‘ ’ 1

Residual standard error: 0.2023 on 2580 degrees of freedom
Multiple R-squared:  0.03389,   Adjusted R-squared:  0.02678 
F-statistic: 4.763 on 19 and 2580 DF,  p-value: 4.51e-11
```


```
anova(m1)
```


```
Analysis of Variance Table

Response: Speed_norm
                  Df  Sum Sq Mean Sq F value    Pr(>F)    
Stim               4   0.362 0.09040  2.2094  0.065597 .  
Task               1   1.749 1.74868 42.7386 7.515e-11 ***
Block              1   0.483 0.48315 11.8084  0.000599 ***
Stim:Task          4   1.013 0.25333  6.1916 5.895e-05 ***
Stim:Block         4   0.035 0.00880  0.2150  0.930189    
Task:Block         1   0.000 0.00001  0.0001  0.990717    
Stim:Task:Block    4   0.061 0.01529  0.3736  0.827591    
Residuals       2580 105.563 0.04092                      
---
Signif. codes:  0 ‘***’ 0.001 ‘**’ 0.01 ‘*’ 0.05 ‘.’ 0.1 ‘ ’ 1
```


```
anova(m0, m1)
```


```
Analysis of Variance Table

Model 1: Speed_norm ~ Stim * Task
Model 2: Speed_norm ~ Stim * Task * Block
  Res.Df    RSS Df Sum of Sq      F Pr(>F)
1   2590 106.14                           
2   2580 105.56 10   0.57949 1.4163 0.1664
```


The model including the blocks does not significantly improve the
proportion of the data explained by the model. We will now assess
whether the addition of random effects improves the linear model.


```
m2 <- lmer(formula = Speed_norm ~ Stim*Task + (1 | ID), data=df_muni)
summary(m2)
```


```
Linear mixed model fit by REML. t-tests use Satterthwaite's method ['lmerModLmerTest']
Formula: Speed_norm ~ Stim * Task + (1 | ID)
   Data: df_muni

REML criterion at convergence: -1128.3

Scaled residuals: 
    Min      1Q  Median      3Q     Max 
-2.7255 -0.6875 -0.0956  0.5683  4.9595 

Random effects:
 Groups   Name        Variance Std.Dev.
 ID       (Intercept) 0.004929 0.07021 
 Residual             0.036281 0.19048 
Number of obs: 2600, groups:  ID, 20

Fixed effects:
                                  Estimate Std. Error         df t value Pr(>|t|)    
(Intercept)                      9.294e-01  1.965e-02  4.167e+01  47.304  < 2e-16 ***
Stimreal_frontal                 2.830e-02  1.671e-02  2.571e+03   1.694 0.090331 .  
Stimreal_parietal               -9.177e-03  1.671e-02  2.571e+03  -0.549 0.582843    
Stimreal_in-phase                4.663e-03  1.671e-02  2.571e+03   0.279 0.780173    
Stimreal_out-of-phase           -1.932e-02  1.671e-02  2.571e+03  -1.156 0.247619    
Task3back                        8.628e-02  1.671e-02  2.571e+03   5.165  2.6e-07 ***
Stimreal_frontal:Task3back      -8.657e-02  2.363e-02  2.571e+03  -3.664 0.000253 ***
Stimreal_parietal:Task3back     -4.920e-02  2.363e-02  2.571e+03  -2.083 0.037388 *  
Stimreal_in-phase:Task3back     -5.777e-02  2.363e-02  2.571e+03  -2.445 0.014543 *  
Stimreal_out-of-phase:Task3back  2.150e-02  2.363e-02  2.571e+03   0.910 0.362985    
---
Signif. codes:  0 ‘***’ 0.001 ‘**’ 0.01 ‘*’ 0.05 ‘.’ 0.1 ‘ ’ 1

Correlation of Fixed Effects:
            (Intr) Stmrl_f Stmrl_p Stmr_- Stm_-- Tsk3bc Stmrl_f:T3 Stmrl_p:T3 S_-:T3
Stmrl_frntl -0.425                                                                  
Stimrl_prtl -0.425  0.500                                                           
Stmrl_n-phs -0.425  0.500   0.500                                                   
Stmrl_t-f-p -0.425  0.500   0.500   0.500                                           
Task3back   -0.425  0.500   0.500   0.500  0.500                                    
Stmrl_fr:T3  0.301 -0.707  -0.354  -0.354 -0.354 -0.707                             
Stmrl_pr:T3  0.301 -0.354  -0.707  -0.354 -0.354 -0.707  0.500                      
Stmrl_n-:T3  0.301 -0.354  -0.354  -0.707 -0.354 -0.707  0.500      0.500           
Stmrl_--:T3  0.301 -0.354  -0.354  -0.354 -0.707 -0.707  0.500      0.500      0.500
```


```
anova(m2)
```


```
Type III Analysis of Variance Table with Satterthwaite's method
          Sum Sq Mean Sq NumDF DenDF F value    Pr(>F)    
Stim      0.3616 0.09040     4  2571  2.4917   0.04125 *  
Task      1.7487 1.74868     1  2571 48.1985 4.859e-12 ***
Stim:Task 1.0133 0.25333     4  2571  6.9825 1.375e-05 ***
---
Signif. codes:  0 ‘***’ 0.001 ‘**’ 0.01 ‘*’ 0.05 ‘.’ 0.1 ‘ ’ 1
```


```
AIC(m0, m2)
```


```
BIC(m0, m2)
```


The addition of the random intercept improves the model. We will now
test if adding the blocks does as well.


```
m3 <- lmer(formula = Speed_norm ~ Stim*Task*Block + (1 | ID), data=df_muni)
summary(m3)
```


```
Linear mixed model fit by REML. t-tests use Satterthwaite's method ['lmerModLmerTest']
Formula: Speed_norm ~ Stim * Task * Block + (1 | ID)
   Data: df_muni

REML criterion at convergence: -1047.5

Scaled residuals: 
    Min      1Q  Median      3Q     Max 
-2.6518 -0.6873 -0.1008  0.5472  4.9496 

Random effects:
 Groups   Name        Variance Std.Dev.
 ID       (Intercept) 0.00493  0.07021 
 Residual             0.03620  0.19025 
Number of obs: 2600, groups:  ID, 20

Fixed effects:
                                        Estimate Std. Error         df t value Pr(>|t|)    
(Intercept)                            9.464e-01  2.955e-02  2.051e+02  32.032   <2e-16 ***
Stimreal_frontal                       1.983e-02  3.540e-02  2.561e+03   0.560   0.5754    
Stimreal_parietal                      1.636e-02  3.540e-02  2.561e+03   0.462   0.6439    
Stimreal_in-phase                      1.038e-02  3.540e-02  2.561e+03   0.293   0.7694    
Stimreal_out-of-phase                 -2.971e-04  3.540e-02  2.561e+03  -0.008   0.9933    
Task3back                              7.867e-02  3.540e-02  2.561e+03   2.222   0.0263 *  
Block                                 -2.437e-03  3.153e-03  2.561e+03  -0.773   0.4398    
Stimreal_frontal:Task3back            -4.845e-02  5.006e-02  2.561e+03  -0.968   0.3332    
Stimreal_parietal:Task3back           -5.932e-02  5.006e-02  2.561e+03  -1.185   0.2361    
Stimreal_in-phase:Task3back           -3.939e-02  5.006e-02  2.561e+03  -0.787   0.4314    
Stimreal_out-of-phase:Task3back        1.402e-02  5.006e-02  2.561e+03   0.280   0.7794    
Stimreal_frontal:Block                 1.211e-03  4.460e-03  2.561e+03   0.272   0.7860    
Stimreal_parietal:Block               -3.648e-03  4.460e-03  2.561e+03  -0.818   0.4134    
Stimreal_in-phase:Block               -8.166e-04  4.460e-03  2.561e+03  -0.183   0.8547    
Stimreal_out-of-phase:Block           -2.717e-03  4.460e-03  2.561e+03  -0.609   0.5424    
Task3back:Block                        1.087e-03  4.460e-03  2.561e+03   0.244   0.8074    
Stimreal_frontal:Task3back:Block      -5.446e-03  6.307e-03  2.561e+03  -0.863   0.3880    
Stimreal_parietal:Task3back:Block      1.445e-03  6.307e-03  2.561e+03   0.229   0.8188    
Stimreal_in-phase:Task3back:Block     -2.625e-03  6.307e-03  2.561e+03  -0.416   0.6772    
Stimreal_out-of-phase:Task3back:Block  1.068e-03  6.307e-03  2.561e+03   0.169   0.8656    
---
Signif. codes:  0 ‘***’ 0.001 ‘**’ 0.01 ‘*’ 0.05 ‘.’ 0.1 ‘ ’ 1
```


```
Correlation matrix not shown by default, as p = 20 > 12.
Use print(x, correlation=TRUE)  or
    vcov(x)        if you need it
```


```
anova(m3)
```


```
Type III Analysis of Variance Table with Satterthwaite's method
                 Sum Sq Mean Sq NumDF DenDF F value    Pr(>F)    
Stim            0.02838 0.00710     4  2561  0.1960 0.9405385    
Task            0.39119 0.39119     1  2561 10.8074 0.0010246 ** 
Block           0.48315 0.48315     1  2561 13.3481 0.0002639 ***
Stim:Task       0.11756 0.02939     4  2561  0.8120 0.5173636    
Stim:Block      0.03519 0.00880     4  2561  0.2431 0.9139479    
Task:Block      0.00001 0.00001     1  2561  0.0002 0.9901308    
Stim:Task:Block 0.06115 0.01529     4  2561  0.4223 0.7926317    
---
Signif. codes:  0 ‘***’ 0.001 ‘**’ 0.01 ‘*’ 0.05 ‘.’ 0.1 ‘ ’ 1
```


```
anova(m2, m3)
```


```
refitting model(s) with ML (instead of REML)
```


```
Data: df_muni
Models:
m2: Speed_norm ~ Stim * Task + (1 | ID)
m3: Speed_norm ~ Stim * Task * Block + (1 | ID)
   npar     AIC     BIC logLik deviance  Chisq Df Pr(>Chisq)  
m2   12 -1171.8 -1101.5 597.92  -1195.8                       
m3   22 -1167.9 -1038.9 605.96  -1211.9 16.078 10    0.09741 .
---
Signif. codes:  0 ‘***’ 0.001 ‘**’ 0.01 ‘*’ 0.05 ‘.’ 0.1 ‘ ’ 1
```


Adding the blocks to m2 does not significantly improve the model.
Next, we will test whether a random slope improves it.


```
m4 <- lmer(formula = Speed_norm ~ Stim*Task*Block + (1 + Block|ID), data=df_muni)
```


```
boundary (singular) fit: see help('isSingular')
```


```
summary(m4)
```


```
Linear mixed model fit by REML. t-tests use Satterthwaite's method ['lmerModLmerTest']
Formula: Speed_norm ~ Stim * Task * Block + (1 + Block | ID)
   Data: df_muni

REML criterion at convergence: -1065.3

Scaled residuals: 
    Min      1Q  Median      3Q     Max 
-2.6014 -0.6700 -0.0892  0.5270  4.9614 

Random effects:
 Groups   Name        Variance  Std.Dev. Corr
 ID       (Intercept) 0.0015551 0.039434     
          Block       0.0000194 0.004404 1.00
 Residual             0.0359315 0.189556     
Number of obs: 2600, groups:  ID, 20

Fixed effects:
                                        Estimate Std. Error         df t value Pr(>|t|)    
(Intercept)                            9.464e-01  2.645e-02  4.908e+02  35.781   <2e-16 ***
Stimreal_frontal                       1.983e-02  3.527e-02  2.561e+03   0.562   0.5740    
Stimreal_parietal                      1.636e-02  3.527e-02  2.561e+03   0.464   0.6427    
Stimreal_in-phase                      1.038e-02  3.527e-02  2.561e+03   0.294   0.7686    
Stimreal_out-of-phase                 -2.971e-04  3.527e-02  2.561e+03  -0.008   0.9933    
Task3back                              7.867e-02  3.527e-02  2.561e+03   2.231   0.0258 *  
Block                                 -2.437e-03  3.293e-03  6.118e+02  -0.740   0.4596    
Stimreal_frontal:Task3back            -4.845e-02  4.988e-02  2.561e+03  -0.971   0.3314    
Stimreal_parietal:Task3back           -5.932e-02  4.988e-02  2.561e+03  -1.189   0.2344    
Stimreal_in-phase:Task3back           -3.939e-02  4.988e-02  2.561e+03  -0.790   0.4297    
Stimreal_out-of-phase:Task3back        1.402e-02  4.988e-02  2.561e+03   0.281   0.7786    
Stimreal_frontal:Block                 1.211e-03  4.443e-03  2.561e+03   0.273   0.7852    
Stimreal_parietal:Block               -3.648e-03  4.443e-03  2.561e+03  -0.821   0.4116    
Stimreal_in-phase:Block               -8.166e-04  4.443e-03  2.561e+03  -0.184   0.8542    
Stimreal_out-of-phase:Block           -2.717e-03  4.443e-03  2.561e+03  -0.612   0.5409    
Task3back:Block                        1.087e-03  4.443e-03  2.561e+03   0.245   0.8068    
Stimreal_frontal:Task3back:Block      -5.446e-03  6.284e-03  2.561e+03  -0.867   0.3862    
Stimreal_parietal:Task3back:Block      1.445e-03  6.284e-03  2.561e+03   0.230   0.8181    
Stimreal_in-phase:Task3back:Block     -2.625e-03  6.284e-03  2.561e+03  -0.418   0.6761    
Stimreal_out-of-phase:Task3back:Block  1.068e-03  6.284e-03  2.561e+03   0.170   0.8651    
---
Signif. codes:  0 ‘***’ 0.001 ‘**’ 0.01 ‘*’ 0.05 ‘.’ 0.1 ‘ ’ 1
```


```
Correlation matrix not shown by default, as p = 20 > 12.
Use print(x, correlation=TRUE)  or
    vcov(x)        if you need it
```


```
optimizer (nloptwrap) convergence code: 0 (OK)
boundary (singular) fit: see help('isSingular')
```


```
anova(m4)
```


```
Type III Analysis of Variance Table with Satterthwaite's method
                 Sum Sq Mean Sq NumDF   DenDF F value    Pr(>F)    
Stim            0.02838 0.00710     4 2561.00  0.1975 0.9397715    
Task            0.39119 0.39119     1 2561.00 10.8871 0.0009817 ***
Block           0.24372 0.24372     1   24.74  6.7829 0.0153417 *  
Stim:Task       0.11756 0.02939     4 2561.00  0.8179 0.5135456    
Stim:Block      0.03519 0.00880     4 2561.00  0.2448 0.9128751    
Task:Block      0.00001 0.00001     1 2561.00  0.0002 0.9900945    
Stim:Task:Block 0.06115 0.01529     4 2561.00  0.4255 0.7903725    
---
Signif. codes:  0 ‘***’ 0.001 ‘**’ 0.01 ‘*’ 0.05 ‘.’ 0.1 ‘ ’ 1
```


```
anova(m2, m4)
```


```
refitting model(s) with ML (instead of REML)
```


```
Data: df_muni
Models:
m2: Speed_norm ~ Stim * Task + (1 | ID)
m4: Speed_norm ~ Stim * Task * Block + (1 + Block | ID)
   npar     AIC     BIC logLik deviance  Chisq Df Pr(>Chisq)    
m2   12 -1171.8 -1101.5 597.92  -1195.8                         
m4   24 -1181.8 -1041.1 614.91  -1229.8 33.981 12  0.0006792 ***
---
Signif. codes:  0 ‘***’ 0.001 ‘**’ 0.01 ‘*’ 0.05 ‘.’ 0.1 ‘ ’ 1
```


Adding a random slope significantly worsens the model according to
the BIC. Next, we will test the addition of a random intercept for the
days.


```
m5 <- lmer(formula = Speed_norm ~ Stim*Task + (1 + (1|ID) + (1|Day)), data=df_muni)
summary(m5)
```


```
Linear mixed model fit by REML. t-tests use Satterthwaite's method ['lmerModLmerTest']
Formula: Speed_norm ~ Stim * Task + (1 + (1 | ID) + (1 | Day))
   Data: df_muni

REML criterion at convergence: -1140.2

Scaled residuals: 
    Min      1Q  Median      3Q     Max 
-2.8861 -0.6938 -0.0976  0.5732  4.9741 

Random effects:
 Groups   Name        Variance  Std.Dev.
 ID       (Intercept) 0.0049312 0.07022 
 Day      (Intercept) 0.0003415 0.01848 
 Residual             0.0360146 0.18978 
Number of obs: 2600, groups:  ID, 20; Day, 5

Fixed effects:
                                  Estimate Std. Error         df t value Pr(>|t|)    
(Intercept)                      9.296e-01  2.130e-02  3.892e+01  43.644  < 2e-16 ***
Stimreal_frontal                 2.993e-02  1.665e-02  2.568e+03   1.797  0.07239 .  
Stimreal_parietal               -6.359e-03  1.668e-02  2.570e+03  -0.381  0.70307    
Stimreal_in-phase               -1.181e-03  1.678e-02  2.565e+03  -0.070  0.94388    
Stimreal_out-of-phase           -1.902e-02  1.667e-02  2.570e+03  -1.141  0.25409    
Task3back                        8.628e-02  1.664e-02  2.567e+03   5.184 2.35e-07 ***
Stimreal_frontal:Task3back      -8.657e-02  2.354e-02  2.567e+03  -3.678  0.00024 ***
Stimreal_parietal:Task3back     -4.920e-02  2.354e-02  2.567e+03  -2.090  0.03669 *  
Stimreal_in-phase:Task3back     -5.777e-02  2.354e-02  2.567e+03  -2.454  0.01418 *  
Stimreal_out-of-phase:Task3back  2.150e-02  2.354e-02  2.567e+03   0.913  0.36122    
---
Signif. codes:  0 ‘***’ 0.001 ‘**’ 0.01 ‘*’ 0.05 ‘.’ 0.1 ‘ ’ 1

Correlation of Fixed Effects:
            (Intr) Stmrl_f Stmrl_p Stmr_- Stm_-- Tsk3bc Stmrl_f:T3 Stmrl_p:T3 S_-:T3
Stmrl_frntl -0.390                                                                  
Stimrl_prtl -0.391  0.500                                                           
Stmrl_n-phs -0.390  0.492   0.494                                                   
Stmrl_t-f-p -0.391  0.498   0.499   0.500                                           
Task3back   -0.391  0.500   0.499   0.496  0.499                                    
Stmrl_fr:T3  0.276 -0.707  -0.353  -0.351 -0.353 -0.707                             
Stmrl_pr:T3  0.276 -0.353  -0.706  -0.351 -0.353 -0.707  0.500                      
Stmrl_n-:T3  0.276 -0.353  -0.353  -0.701 -0.353 -0.707  0.500      0.500           
Stmrl_--:T3  0.276 -0.353  -0.353  -0.351 -0.706 -0.707  0.500      0.500      0.500
```


```
anova(m5)
```


```
Type III Analysis of Variance Table with Satterthwaite's method
           Sum Sq Mean Sq NumDF  DenDF F value    Pr(>F)    
Stim      0.38136 0.09534     4 2518.9  2.6472   0.03183 *  
Task      1.74868 1.74868     1 2567.0 48.5547 4.067e-12 ***
Stim:Task 1.01333 0.25333     4 2567.0  7.0341 1.251e-05 ***
---
Signif. codes:  0 ‘***’ 0.001 ‘**’ 0.01 ‘*’ 0.05 ‘.’ 0.1 ‘ ’ 1
```


```
anova(m2, m5)
```


```
refitting model(s) with ML (instead of REML)
```


```
Data: df_muni
Models:
m2: Speed_norm ~ Stim * Task + (1 | ID)
m5: Speed_norm ~ Stim * Task + (1 + (1 | ID) + (1 | Day))
   npar     AIC     BIC logLik deviance  Chisq Df Pr(>Chisq)    
m2   12 -1171.8 -1101.5 597.92  -1195.8                         
m5   13 -1181.5 -1105.3 603.74  -1207.5 11.641  1  0.0006452 ***
---
Signif. codes:  0 ‘***’ 0.001 ‘**’ 0.01 ‘*’ 0.05 ‘.’ 0.1 ‘ ’ 1
```


The model including random intercepts for days and subjects improves
the one including only a random intercept for subjects.


```
m6 <- lmer(formula = Speed_norm ~ Stim*Task*Block + (1 + (1|ID) + (1|Day)), data=df_muni)
summary(m6)
```


```
Linear mixed model fit by REML. t-tests use Satterthwaite's method ['lmerModLmerTest']
Formula: Speed_norm ~ Stim * Task * Block + (1 + (1 | ID) + (1 | Day))
   Data: df_muni

REML criterion at convergence: -1059.5

Scaled residuals: 
    Min      1Q  Median      3Q     Max 
-2.8125 -0.6859 -0.0945  0.5523  4.9643 

Random effects:
 Groups   Name        Variance  Std.Dev.
 ID       (Intercept) 0.0049318 0.07023 
 Day      (Intercept) 0.0003417 0.01848 
 Residual             0.0359289 0.18955 
Number of obs: 2600, groups:  ID, 20; Day, 5

Fixed effects:
                                        Estimate Std. Error         df t value Pr(>|t|)    
(Intercept)                            9.466e-01  3.061e-02  1.620e+02  30.925   <2e-16 ***
Stimreal_frontal                       2.146e-02  3.527e-02  2.557e+03   0.608   0.5430    
Stimreal_parietal                      1.918e-02  3.528e-02  2.558e+03   0.544   0.5867    
Stimreal_in-phase                      4.532e-03  3.533e-02  2.560e+03   0.128   0.8980    
Stimreal_out-of-phase                  4.442e-06  3.528e-02  2.558e+03   0.000   0.9999    
Task3back                              7.867e-02  3.527e-02  2.557e+03   2.231   0.0258 *  
Block                                 -2.437e-03  3.142e-03  2.557e+03  -0.776   0.4381    
Stimreal_frontal:Task3back            -4.845e-02  4.987e-02  2.557e+03  -0.971   0.3314    
Stimreal_parietal:Task3back           -5.932e-02  4.987e-02  2.557e+03  -1.189   0.2344    
Stimreal_in-phase:Task3back           -3.939e-02  4.987e-02  2.557e+03  -0.790   0.4297    
Stimreal_out-of-phase:Task3back        1.402e-02  4.987e-02  2.557e+03   0.281   0.7786    
Stimreal_frontal:Block                 1.211e-03  4.443e-03  2.557e+03   0.273   0.7852    
Stimreal_parietal:Block               -3.649e-03  4.443e-03  2.557e+03  -0.821   0.4116    
Stimreal_in-phase:Block               -8.166e-04  4.443e-03  2.557e+03  -0.184   0.8542    
Stimreal_out-of-phase:Block           -2.717e-03  4.443e-03  2.557e+03  -0.612   0.5409    
Task3back:Block                        1.087e-03  4.443e-03  2.557e+03   0.245   0.8067    
Stimreal_frontal:Task3back:Block      -5.446e-03  6.283e-03  2.557e+03  -0.867   0.3862    
Stimreal_parietal:Task3back:Block      1.445e-03  6.283e-03  2.557e+03   0.230   0.8181    
Stimreal_in-phase:Task3back:Block     -2.625e-03  6.283e-03  2.557e+03  -0.418   0.6761    
Stimreal_out-of-phase:Task3back:Block  1.068e-03  6.283e-03  2.557e+03   0.170   0.8651    
---
Signif. codes:  0 ‘***’ 0.001 ‘**’ 0.01 ‘*’ 0.05 ‘.’ 0.1 ‘ ’ 1
```


```
Correlation matrix not shown by default, as p = 20 > 12.
Use print(x, correlation=TRUE)  or
    vcov(x)        if you need it
```


```
anova(m6)
```


```
Type III Analysis of Variance Table with Satterthwaite's method
                 Sum Sq Mean Sq NumDF DenDF F value    Pr(>F)    
Stim            0.03514 0.00879     4  2558  0.2445 0.9130745    
Task            0.39119 0.39119     1  2557 10.8878 0.0009813 ***
Block           0.48315 0.48315     1  2557 13.4474 0.0002503 ***
Stim:Task       0.11756 0.02939     4  2557  0.8180 0.5135080    
Stim:Block      0.03519 0.00880     4  2557  0.2449 0.9128644    
Task:Block      0.00001 0.00001     1  2557  0.0002 0.9900942    
Stim:Task:Block 0.06115 0.01529     4  2557  0.4255 0.7903500    
---
Signif. codes:  0 ‘***’ 0.001 ‘**’ 0.01 ‘*’ 0.05 ‘.’ 0.1 ‘ ’ 1
```


```
anova(m5, m6)
```


```
refitting model(s) with ML (instead of REML)
```


```
Data: df_muni
Models:
m5: Speed_norm ~ Stim * Task + (1 + (1 | ID) + (1 | Day))
m6: Speed_norm ~ Stim * Task * Block + (1 + (1 | ID) + (1 | Day))
   npar     AIC     BIC logLik deviance  Chisq Df Pr(>Chisq)  
m5   13 -1181.5 -1105.3 603.74  -1207.5                       
m6   23 -1177.7 -1042.8 611.84  -1223.7 16.197 10    0.09412 .
---
Signif. codes:  0 ‘***’ 0.001 ‘**’ 0.01 ‘*’ 0.05 ‘.’ 0.1 ‘ ’ 1
```


Adding the blocks to m5 does not significantly improve it, so we will
keep m5.


```
m_speed_norm <- lmer(formula = Speed_norm ~ Stim*Task + (1 + (1|ID) + (1|Day)), data=df_muni)
summary(m_speed_norm)
```


```
Linear mixed model fit by REML. t-tests use Satterthwaite's method ['lmerModLmerTest']
Formula: Speed_norm ~ Stim * Task + (1 + (1 | ID) + (1 | Day))
   Data: df_muni

REML criterion at convergence: -1140.2

Scaled residuals: 
    Min      1Q  Median      3Q     Max 
-2.8861 -0.6938 -0.0976  0.5732  4.9741 

Random effects:
 Groups   Name        Variance  Std.Dev.
 ID       (Intercept) 0.0049312 0.07022 
 Day      (Intercept) 0.0003415 0.01848 
 Residual             0.0360146 0.18978 
Number of obs: 2600, groups:  ID, 20; Day, 5

Fixed effects:
                                  Estimate Std. Error         df t value Pr(>|t|)    
(Intercept)                      9.296e-01  2.130e-02  3.892e+01  43.644  < 2e-16 ***
Stimreal_frontal                 2.993e-02  1.665e-02  2.568e+03   1.797  0.07239 .  
Stimreal_parietal               -6.359e-03  1.668e-02  2.570e+03  -0.381  0.70307    
Stimreal_in-phase               -1.181e-03  1.678e-02  2.565e+03  -0.070  0.94388    
Stimreal_out-of-phase           -1.902e-02  1.667e-02  2.570e+03  -1.141  0.25409    
Task3back                        8.628e-02  1.664e-02  2.567e+03   5.184 2.35e-07 ***
Stimreal_frontal:Task3back      -8.657e-02  2.354e-02  2.567e+03  -3.678  0.00024 ***
Stimreal_parietal:Task3back     -4.920e-02  2.354e-02  2.567e+03  -2.090  0.03669 *  
Stimreal_in-phase:Task3back     -5.777e-02  2.354e-02  2.567e+03  -2.454  0.01418 *  
Stimreal_out-of-phase:Task3back  2.150e-02  2.354e-02  2.567e+03   0.913  0.36122    
---
Signif. codes:  0 ‘***’ 0.001 ‘**’ 0.01 ‘*’ 0.05 ‘.’ 0.1 ‘ ’ 1

Correlation of Fixed Effects:
            (Intr) Stmrl_f Stmrl_p Stmr_- Stm_-- Tsk3bc Stmrl_f:T3 Stmrl_p:T3 S_-:T3
Stmrl_frntl -0.390                                                                  
Stimrl_prtl -0.391  0.500                                                           
Stmrl_n-phs -0.390  0.492   0.494                                                   
Stmrl_t-f-p -0.391  0.498   0.499   0.500                                           
Task3back   -0.391  0.500   0.499   0.496  0.499                                    
Stmrl_fr:T3  0.276 -0.707  -0.353  -0.351 -0.353 -0.707                             
Stmrl_pr:T3  0.276 -0.353  -0.706  -0.351 -0.353 -0.707  0.500                      
Stmrl_n-:T3  0.276 -0.353  -0.353  -0.701 -0.353 -0.707  0.500      0.500           
Stmrl_--:T3  0.276 -0.353  -0.353  -0.351 -0.706 -0.707  0.500      0.500      0.500
```


```
anova(m_speed_norm)
```


```
Type III Analysis of Variance Table with Satterthwaite's method
           Sum Sq Mean Sq NumDF  DenDF F value    Pr(>F)    
Stim      0.38136 0.09534     4 2518.9  2.6472   0.03183 *  
Task      1.74868 1.74868     1 2567.0 48.5547 4.067e-12 ***
Stim:Task 1.01333 0.25333     4 2567.0  7.0341 1.251e-05 ***
---
Signif. codes:  0 ‘***’ 0.001 ‘**’ 0.01 ‘*’ 0.05 ‘.’ 0.1 ‘ ’ 1
```


```
eta_squared(m_speed_norm)
```


```
# Effect Size for ANOVA (Type III)

Parameter | Eta2 (partial) |       95% CI
-----------------------------------------
Stim      |       4.19e-03 | [0.00, 1.00]
Task      |           0.02 | [0.01, 1.00]
Stim:Task |           0.01 | [0.00, 1.00]

- One-sided CIs: upper bound fixed at [1.00].
```


### Normalized speed in the 2-back task


```
data_subset <- subset(df_muni, Task == '2back')
data_subset <- droplevels(data_subset)
levels(data_subset$Task)
```


```
[1] "2back"
```


```
m_speed_norm_2back <- lmer(formula = Speed_norm ~ Stim + (1 + (1|ID) + (1|Day)), data=data_subset)
summary(m_speed_norm_2back)
```


```
Linear mixed model fit by REML. t-tests use Satterthwaite's method ['lmerModLmerTest']
Formula: Speed_norm ~ Stim + (1 + (1 | ID) + (1 | Day))
   Data: data_subset

REML criterion at convergence: -955.2

Scaled residuals: 
    Min      1Q  Median      3Q     Max 
-2.6862 -0.7073 -0.0722  0.6176  3.2645 

Random effects:
 Groups   Name        Variance Std.Dev.
 ID       (Intercept) 0.009690 0.09844 
 Day      (Intercept) 0.001263 0.03554 
 Residual             0.025929 0.16103 
Number of obs: 1300, groups:  ID, 20; Day, 5

Fixed effects:
                        Estimate Std. Error         df t value Pr(>|t|)    
(Intercept)            9.255e-01  2.894e-02  2.188e+01  31.985   <2e-16 ***
Stimreal_frontal       2.617e-02  1.414e-02  1.273e+03   1.851   0.0644 .  
Stimreal_parietal     -5.769e-03  1.419e-02  1.274e+03  -0.407   0.6844    
Stimreal_in-phase      1.663e-02  1.438e-02  1.276e+03   1.156   0.2478    
Stimreal_out-of-phase -1.338e-02  1.417e-02  1.273e+03  -0.944   0.3454    
---
Signif. codes:  0 ‘***’ 0.001 ‘**’ 0.01 ‘*’ 0.05 ‘.’ 0.1 ‘ ’ 1

Correlation of Fixed Effects:
            (Intr) Stmrl_f Stmrl_p Stmr_-
Stmrl_frntl -0.243                       
Stimrl_prtl -0.244  0.499                
Stmrl_n-phs -0.243  0.482   0.486        
Stmrl_t-f-p -0.245  0.495   0.498   0.500
```


```
anova(m_speed_norm_2back)
```


```
Type III Analysis of Variance Table with Satterthwaite's method
     Sum Sq  Mean Sq NumDF  DenDF F value  Pr(>F)  
Stim 0.2746 0.068651     4 1274.5  2.6476 0.03205 *
---
Signif. codes:  0 ‘***’ 0.001 ‘**’ 0.01 ‘*’ 0.05 ‘.’ 0.1 ‘ ’ 1
```


```
eta_squared(m_speed_norm_2back)
```


```
# Effect Size for ANOVA (Type III)

Parameter | Eta2 (partial) |       95% CI
-----------------------------------------
Stim      |       8.24e-03 | [0.00, 1.00]

- One-sided CIs: upper bound fixed at [1.00].
```


```
my_model <- m_speed_norm_2back
level_1 <- "Stim"

my_model.compare <- emmeans(my_model, level_1)
my_model.compare.pairs <- pairs(my_model.compare, adjust='tukey')
test(my_model.compare.pairs, side='two-sided')
```


```
 contrast                              estimate     SE   df t.ratio p.value
 Placebo_loc - real_frontal            -0.02617 0.0141 1273  -1.851  0.3449
 Placebo_loc - real_parietal            0.00577 0.0142 1274   0.406  0.9943
 Placebo_loc - (real_in-phase)         -0.01663 0.0144 1276  -1.154  0.7773
 Placebo_loc - (real_out-of-phase)      0.01338 0.0142 1273   0.944  0.8797
 real_frontal - real_parietal           0.03194 0.0142 1273   2.253  0.1611
 real_frontal - (real_in-phase)         0.00954 0.0146 1275   0.656  0.9656
 real_frontal - (real_out-of-phase)     0.03955 0.0142 1274   2.779  0.0439
 real_parietal - (real_in-phase)       -0.02240 0.0145 1276  -1.543  0.5346
 real_parietal - (real_out-of-phase)    0.00761 0.0142 1274   0.535  0.9837
 (real_in-phase) - (real_out-of-phase)  0.03001 0.0143 1275   2.100  0.2207

Degrees-of-freedom method: kenward-roger 
P value adjustment: tukey method for comparing a family of 5 estimates
```


```
confint(my_model.compare, calc = c(n = ~.wgt.))
```


```
 Stim              emmean     SE   df   n lower.CL upper.CL
 Placebo_loc        0.926 0.0289 21.9 260    0.866    0.986
 real_frontal       0.952 0.0290 21.9 260    0.892    1.012
 real_parietal      0.920 0.0290 21.9 260    0.860    0.980
 real_in-phase      0.942 0.0290 22.1 260    0.882    1.002
 real_out-of-phase  0.912 0.0289 21.9 260    0.852    0.972

Degrees-of-freedom method: kenward-roger 
Confidence level used: 0.95
```


```
eff_size(my_model.compare, sigma = sigma(my_model), edf = 23)
```


```
 contrast                              effect.size     SE   df lower.CL upper.CL
 Placebo_loc - real_frontal                -0.1625 0.0910 21.9 -0.35139   0.0263
 Placebo_loc - real_parietal                0.0358 0.0883 21.9 -0.14737   0.2190
 Placebo_loc - (real_in-phase)             -0.1033 0.0907 21.9 -0.29152   0.0850
 Placebo_loc - (real_out-of-phase)          0.0831 0.0889 21.9 -0.10132   0.2675
 real_frontal - real_parietal               0.1984 0.0928 21.9  0.00593   0.3908
 real_frontal - (real_in-phase)             0.0593 0.0908 21.9 -0.12909   0.2476
 real_frontal - (real_out-of-phase)         0.2456 0.0955 21.9  0.04745   0.4438
 real_parietal - (real_in-phase)           -0.1391 0.0925 21.9 -0.33086   0.0527
 real_parietal - (real_out-of-phase)        0.0473 0.0886 21.9 -0.13644   0.2310
 (real_in-phase) - (real_out-of-phase)      0.1864 0.0929 21.9 -0.00638   0.3791

sigma used for effect sizes: 0.161 
Degrees-of-freedom method: inherited from kenward-roger when re-gridding 
Confidence level used: 0.95
```


Even though there is a significant effect of stimulation, there is no
evidence for difference between any of the stimulation variants and the
placebo.

### Normalized accuracy in the 3-back task


```
data_subset <- subset(df_muni, Task == '3back')
data_subset <- droplevels(data_subset)
levels(data_subset$Task)
```


```
[1] "3back"
```


```
m_speed_norm_3back <- lmer(formula = Speed_norm ~ Stim + (1 + (1|ID) + (1|Day)), data=data_subset)
summary(m_speed_norm_3back)
```


```
Linear mixed model fit by REML. t-tests use Satterthwaite's method ['lmerModLmerTest']
Formula: Speed_norm ~ Stim + (1 + (1 | ID) + (1 | Day))
   Data: data_subset

REML criterion at convergence: -486.5

Scaled residuals: 
    Min      1Q  Median      3Q     Max 
-2.6352 -0.6840 -0.0608  0.5547  4.1908 

Random effects:
 Groups   Name        Variance Std.Dev.
 ID       (Intercept) 0.006574 0.08108 
 Day      (Intercept) 0.002579 0.05078 
 Residual             0.037594 0.19389 
Number of obs: 1300, groups:  ID, 20; Day, 5

Fixed effects:
                        Estimate Std. Error         df t value Pr(>|t|)    
(Intercept)            1.020e+00  3.146e-02  1.202e+01  32.424 4.49e-13 ***
Stimreal_frontal      -5.234e-02  1.703e-02  1.272e+03  -3.074  0.00216 ** 
Stimreal_parietal     -5.541e-02  1.709e-02  1.273e+03  -3.243  0.00121 ** 
Stimreal_in-phase     -7.879e-02  1.733e-02  1.276e+03  -4.548 5.94e-06 ***
Stimreal_out-of-phase -3.242e-03  1.707e-02  1.273e+03  -0.190  0.84939    
---
Signif. codes:  0 ‘***’ 0.001 ‘**’ 0.01 ‘*’ 0.05 ‘.’ 0.1 ‘ ’ 1

Correlation of Fixed Effects:
            (Intr) Stmrl_f Stmrl_p Stmr_-
Stmrl_frntl -0.269                       
Stimrl_prtl -0.270  0.499                
Stmrl_n-phs -0.269  0.481   0.486        
Stmrl_t-f-p -0.271  0.495   0.498   0.500
```


```
anova(m_speed_norm_3back)
```


```
Type III Analysis of Variance Table with Satterthwaite's method
     Sum Sq Mean Sq NumDF  DenDF F value    Pr(>F)    
Stim 1.2397 0.30991     4 1274.2  8.2438 1.455e-06 ***
---
Signif. codes:  0 ‘***’ 0.001 ‘**’ 0.01 ‘*’ 0.05 ‘.’ 0.1 ‘ ’ 1
```


```
eta_squared(m_speed_norm_3back)
```


```
# Effect Size for ANOVA (Type III)

Parameter | Eta2 (partial) |       95% CI
-----------------------------------------
Stim      |           0.03 | [0.01, 1.00]

- One-sided CIs: upper bound fixed at [1.00].
```


```
my_model <- m_speed_norm_3back
level_1 <- "Stim"

my_model.compare <- emmeans(my_model, level_1)
my_model.compare.pairs <- pairs(my_model.compare, adjust='tukey')
test(my_model.compare.pairs, side='two-sided')
```


```
 contrast                              estimate     SE   df t.ratio p.value
 Placebo_loc - real_frontal             0.05234 0.0170 1272   3.074  0.0183
 Placebo_loc - real_parietal            0.05541 0.0171 1273   3.242  0.0107
 Placebo_loc - (real_in-phase)          0.07879 0.0173 1276   4.543  0.0001
 Placebo_loc - (real_out-of-phase)      0.00324 0.0171 1273   0.190  0.9997
 real_frontal - real_parietal           0.00307 0.0171 1273   0.180  0.9998
 real_frontal - (real_in-phase)         0.02645 0.0175 1276   1.509  0.5566
 real_frontal - (real_out-of-phase)    -0.04910 0.0171 1274  -2.865  0.0344
 real_parietal - (real_in-phase)        0.02338 0.0175 1276   1.338  0.6677
 real_parietal - (real_out-of-phase)   -0.05217 0.0171 1274  -3.048  0.0199
 (real_in-phase) - (real_out-of-phase) -0.07555 0.0172 1275  -4.390  0.0001

Degrees-of-freedom method: kenward-roger 
P value adjustment: tukey method for comparing a family of 5 estimates
```


```
confint(my_model.compare, calc = c(n = ~.wgt.))
```


```
 Stim              emmean     SE   df   n lower.CL upper.CL
 Placebo_loc        1.020 0.0315 12.1 260    0.952     1.09
 real_frontal       0.968 0.0315 12.1 260    0.899     1.04
 real_parietal      0.965 0.0315 12.1 260    0.896     1.03
 real_in-phase      0.941 0.0316 12.2 260    0.873     1.01
 real_out-of-phase  1.017 0.0315 12.1 260    0.948     1.09

Degrees-of-freedom method: kenward-roger 
Confidence level used: 0.95
```


```
eff_size(my_model.compare, sigma = sigma(my_model), edf = 23)
```


```
 contrast                              effect.size     SE   df lower.CL upper.CL
 Placebo_loc - real_frontal                 0.2700 0.0964 12.1   0.0600   0.4799
 Placebo_loc - real_parietal                0.2858 0.0977 12.1   0.0731   0.4985
 Placebo_loc - (real_in-phase)              0.4064 0.1077 12.1   0.1719   0.6408
 Placebo_loc - (real_out-of-phase)          0.0167 0.0881 12.1  -0.1751   0.2085
 real_frontal - real_parietal               0.0158 0.0881 12.1  -0.1759   0.2075
 real_frontal - (real_in-phase)             0.1364 0.0926 12.1  -0.0651   0.3380
 real_frontal - (real_out-of-phase)        -0.2532 0.0960 12.1  -0.4622  -0.0443
 real_parietal - (real_in-phase)            0.1206 0.0919 12.1  -0.0794   0.3206
 real_parietal - (real_out-of-phase)       -0.2691 0.0968 12.1  -0.4798  -0.0583
 (real_in-phase) - (real_out-of-phase)     -0.3897 0.1057 12.1  -0.6198  -0.1595

sigma used for effect sizes: 0.1939 
Degrees-of-freedom method: inherited from kenward-roger when re-gridding 
Confidence level used: 0.95
```

## Statistical analysis relating the estimated induced electric fields and behavior.


```
file_name <- file.path(paste(data_directory, "Behavioral_and_efield_blocks_iCOG_Design_25_04_2023.txt", sep='/'))
df_efields <- read.delim(file_name, header = TRUE, na.strings = "NN")
head(df_efields, 5)
```


```
df_efields$ID <- as.factor(df_efields$ID)
df_efields$School <- as.factor(df_efields$School)
df_efields$Day <- as.factor(df_efields$Day)
df_efields$Task <- as.factor(df_efields$Task)
df_efields$Stim <- as.factor(df_efields$Stim)
df_efields$Acc_u <- as.numeric(df_efields$Acc_u)
df_efields$Speed_u <- as.numeric(df_efields$Speed_u)
df_efields$Acc_m <- as.numeric(df_efields$Acc_m)
df_efields$Acc_n_m <- as.numeric(df_efields$Acc_n_m)
df_efields$Speed_m <- as.numeric(df_efields$Speed_m)
df_efields$Speed_n_m <- as.numeric(df_efields$Speed_n_m)
df_efields$EF_front <- as.numeric(df_efields$EF_front)
df_efields$EF_par <- as.numeric(df_efields$EF_par)
df_efields$EF_mag <- as.numeric(df_efields$EF_mag)
df_efields$Error_front <- as.numeric(df_efields$Error_front)
df_efields$Error_par <- as.numeric(df_efields$Error_par)
```


```
df_ef_muni <- subset(df_efields, School == 'MUNI')
df_ef_muni <- droplevels(df_ef_muni)
#levels(df_ef_epfl$School)
head(df_ef_muni, 5)
```


```
df_ef_muni$Stim <- factor(df_ef_muni$Stim, levels(df_ef_muni$Stim)[c(1, 2, 5, 3, 4)])
levels(df_ef_muni$Stim)
```


```
[1] "Placebo_loc"       "real_frontal"      "real_parietal"     "real_in-phase"     "real_out-of-phase"
```


### Note about the analysis

The idea of this analysis is to verify whether there was a
differentiated effect of stimulation resulting from the magnitude of the
electric fields induced at both stimulation sites at an individual
level. The main analysis described above showed significant effects of
stimulation in the normalized data, which depicts relative changes in
performance for each individual. For this reason, we will assess the
effect of the magnitude of the induced electric fields on the change in
performance (i.e., in terms of speed and accuracy) within each training
session. This change in performance is described as the slope of the
line minimizing the error in a least-squared sense, fitted to each
individual.

This data frame contains the magnitude of the estimated electrical
field for each electrode (i.e., frontal and parietal). The electrode
locations were determined by having participants perform the 2-back task
inside the MRI scanner and finding the region were the task-related bold
signal peaked. This regions were then projected into the scalp of each
participant and the resulting coordinates were used to place the
electrodes. In the data frame, the columns labeled as “EF” contain the
magnitude of the electric field estimated for each intended target,
while the columns labeled with “error” include the distance between the
intended target and the location were the maximum electric field was
induced. The column labeled “EF\_mag” contains the magnitude of the
electric field induced on both sites (mag = sqrt(a^2 + b^2)). The column
“EF\_sym” contains the ratio of the electric field induced on the frontal
site to the electric field induced in the parietal site
(EF\_front/EF\_par), and constitutes a measure of how symmetric the
induced fields were. By design and under ideal circumstances, the
induced fields would be identical, but anatomical differences among the
sites often result in different fields being induced.

In the MUNI cohorts, the intensity of the applied current was of 2 mA
peak-to-peak. The first two stimulation conditions involved monofocal
stimulation (i.e., either frontal or parietal), while condition 3 and
condition 4 involved bifocal stimulation, applied either in phase
(condition 3) or out of phase (condition 4). We will assess the
influence of the magnitude of the induced electric fields on behavior
considering the monofocal and the bifocal stimulation conditions
separately.

### The relationship between the intensity of the applied electric field on the target and the accuracy

### Monofocal conditions

For the monofocal conditions, the variable we will be using for the
fields is “EF\_mag”. This variable is the magnitude of the vector
describing the electric field in terms of a frontal and a parietal
component, but as these stimulation variants are monofocal, the value
for EF\_mag is the same as for EF\_front in the frontal condition and
EF\_par in the parietal condition.


```
data_subset <- subset(df_ef_muni, Stim == "real_frontal" | Stim == "real_parietal")
data_subset <- droplevels(data_subset)
levels(data_subset$Stim)
```


```
[1] "real_frontal"  "real_parietal"
```


```
m0 <- lm(formula = Acc_m ~ Task*Stim*EF_mag, data=data_subset)
summary(m0)
```


```
Call:
lm(formula = Acc_m ~ Task * Stim * EF_mag, data = data_subset)

Residuals:
       Min         1Q     Median         3Q        Max 
-0.0209062 -0.0036047  0.0000508  0.0066803  0.0149306 

Coefficients:
                                     Estimate Std. Error t value Pr(>|t|)
(Intercept)                         0.0015322  0.0054562   0.281    0.780
Task3back                          -0.0006028  0.0077162  -0.078    0.938
Stimreal_parietal                  -0.0055611  0.0064824  -0.858    0.394
EF_mag                             -0.0050237  0.0791549  -0.063    0.950
Task3back:Stimreal_parietal         0.0057408  0.0091675   0.626    0.533
Task3back:EF_mag                    0.0298517  0.1119420   0.267    0.790
Stimreal_parietal:EF_mag            0.0818939  0.0911747   0.898    0.372
Task3back:Stimreal_parietal:EF_mag -0.1261343  0.1289406  -0.978    0.331

Residual standard error: 0.008731 on 72 degrees of freedom
Multiple R-squared:  0.05414,   Adjusted R-squared:  -0.03782 
F-statistic: 0.5887 on 7 and 72 DF,  p-value: 0.763
```


```
anova(m0)
```


```
Analysis of Variance Table

Response: Acc_m
                 Df    Sum Sq    Mean Sq F value Pr(>F)
Task              1 0.0000004 3.7700e-07  0.0050 0.9441
Stim              1 0.0000440 4.4016e-05  0.5774 0.4498
EF_mag            1 0.0000573 5.7350e-05  0.7523 0.3886
Task:Stim         1 0.0000279 2.7910e-05  0.3661 0.5470
Task:EF_mag       1 0.0001051 1.0506e-04  1.3782 0.2443
Stim:EF_mag       1 0.0000065 6.5010e-06  0.0853 0.7711
Task:Stim:EF_mag  1 0.0000730 7.2951e-05  0.9569 0.3312
Residuals        72 0.0054888 7.6234e-05
```


```
m1 <- lmer(formula = Acc_m ~ Task*Stim*EF_mag + (1 | ID), data=data_subset)
summary(m1)
```


```
Linear mixed model fit by REML. t-tests use Satterthwaite's method ['lmerModLmerTest']
Formula: Acc_m ~ Task * Stim * EF_mag + (1 | ID)
   Data: data_subset

REML criterion at convergence: -482.6

Scaled residuals: 
     Min       1Q   Median       3Q      Max 
-2.44076 -0.44553  0.06946  0.72067  1.79950 

Random effects:
 Groups   Name        Variance Std.Dev.
 ID       (Intercept) 7.3e-06  0.002702
 Residual             6.9e-05  0.008307
Number of obs: 80, groups:  ID, 20

Fixed effects:
                                     Estimate Std. Error         df t value Pr(>|t|)
(Intercept)                         0.0003057  0.0054193 71.8997292   0.056    0.955
Task3back                          -0.0006028  0.0073411 52.3071849  -0.082    0.935
Stimreal_parietal                  -0.0040772  0.0063577 68.6088040  -0.641    0.523
EF_mag                              0.0140304  0.0785367 71.6676593   0.179    0.859
Task3back:Stimreal_parietal         0.0057408  0.0087219 52.3071849   0.658    0.513
Task3back:EF_mag                    0.0298517  0.1065008 52.3071849   0.280    0.780
Stimreal_parietal:EF_mag            0.0588289  0.0900034 70.5793203   0.654    0.515
Task3back:Stimreal_parietal:EF_mag -0.1261343  0.1226732 52.3071849  -1.028    0.309

Correlation of Fixed Effects:
            (Intr) Tsk3bc Stmrl_ EF_mag Ts3:S_ T3:EF_ S_:EF_
Task3back   -0.677                                          
Stimrl_prtl -0.837  0.577                                   
EF_mag      -0.933  0.633  0.790                            
Tsk3bck:St_  0.570 -0.842 -0.686 -0.533                     
Tsk3bck:EF_  0.632 -0.934 -0.539 -0.678  0.786              
Stmrl_p:EF_  0.808 -0.552 -0.911 -0.867  0.617  0.592       
Tsk3:S_:EF_ -0.549  0.811  0.621  0.589 -0.905 -0.868 -0.681
```


```
anova(m1)
```


```
Type III Analysis of Variance Table with Satterthwaite's method
                     Sum Sq    Mean Sq NumDF  DenDF F value Pr(>F)
Task             1.8657e-05 1.8657e-05     1 52.307  0.2704 0.6053
Stim             4.6950e-06 4.6950e-06     1 70.886  0.0680 0.7950
EF_mag           4.4135e-05 4.4135e-05     1 56.665  0.6396 0.4272
Task:Stim        2.9894e-05 2.9894e-05     1 52.307  0.4332 0.5133
Task:EF_mag      2.0235e-05 2.0235e-05     1 52.307  0.2933 0.5904
Stim:EF_mag      2.8600e-07 2.8600e-07     1 67.203  0.0041 0.9489
Task:Stim:EF_mag 7.2951e-05 7.2951e-05     1 52.307  1.0572 0.3086
```


```
AIC(m0, m1)
```


```
BIC(m0, m1)
```


```
m_acc_ef <- lm(formula = Acc_m ~ Task*Stim*EF_mag, data=data_subset)
summary(m_acc_ef)
```


```
Call:
lm(formula = Acc_m ~ Task * Stim * EF_mag, data = data_subset)

Residuals:
       Min         1Q     Median         3Q        Max 
-0.0209062 -0.0036047  0.0000508  0.0066803  0.0149306 

Coefficients:
                                     Estimate Std. Error t value Pr(>|t|)
(Intercept)                         0.0015322  0.0054562   0.281    0.780
Task3back                          -0.0006028  0.0077162  -0.078    0.938
Stimreal_parietal                  -0.0055611  0.0064824  -0.858    0.394
EF_mag                             -0.0050237  0.0791549  -0.063    0.950
Task3back:Stimreal_parietal         0.0057408  0.0091675   0.626    0.533
Task3back:EF_mag                    0.0298517  0.1119420   0.267    0.790
Stimreal_parietal:EF_mag            0.0818939  0.0911747   0.898    0.372
Task3back:Stimreal_parietal:EF_mag -0.1261343  0.1289406  -0.978    0.331

Residual standard error: 0.008731 on 72 degrees of freedom
Multiple R-squared:  0.05414,   Adjusted R-squared:  -0.03782 
F-statistic: 0.5887 on 7 and 72 DF,  p-value: 0.763
```


```
anova(m_acc_ef)
```


```
Analysis of Variance Table

Response: Acc_m
                 Df    Sum Sq    Mean Sq F value Pr(>F)
Task              1 0.0000004 3.7700e-07  0.0050 0.9441
Stim              1 0.0000440 4.4016e-05  0.5774 0.4498
EF_mag            1 0.0000573 5.7350e-05  0.7523 0.3886
Task:Stim         1 0.0000279 2.7910e-05  0.3661 0.5470
Task:EF_mag       1 0.0001051 1.0506e-04  1.3782 0.2443
Stim:EF_mag       1 0.0000065 6.5010e-06  0.0853 0.7711
Task:Stim:EF_mag  1 0.0000730 7.2951e-05  0.9569 0.3312
Residuals        72 0.0054888 7.6234e-05
```


```
eta_squared(m_acc_ef)
```


```
# Effect Size for ANOVA (Type I)

Parameter        | Eta2 (partial) |       95% CI
------------------------------------------------
Task             |       6.87e-05 | [0.00, 1.00]
Stim             |       7.96e-03 | [0.00, 1.00]
EF_mag           |           0.01 | [0.00, 1.00]
Task:Stim        |       5.06e-03 | [0.00, 1.00]
Task:EF_mag      |           0.02 | [0.00, 1.00]
Stim:EF_mag      |       1.18e-03 | [0.00, 1.00]
Task:Stim:EF_mag |           0.01 | [0.00, 1.00]

- One-sided CIs: upper bound fixed at [1.00].
```


```
m0 <- lm(formula = Acc_n_m ~ Task*Stim*EF_mag, data=data_subset)
summary(m0)
```


```
Call:
lm(formula = Acc_n_m ~ Task * Stim * EF_mag, data = data_subset)

Residuals:
       Min         1Q     Median         3Q        Max 
-0.0231845 -0.0057477 -0.0006043  0.0090697  0.0217196 

Coefficients:
                                    Estimate Std. Error t value Pr(>|t|)
(Intercept)                         0.004794   0.007216   0.664    0.509
Task3back                          -0.002998   0.010205  -0.294    0.770
Stimreal_parietal                  -0.010199   0.008573  -1.190    0.238
EF_mag                             -0.029495   0.104684  -0.282    0.779
Task3back:Stimreal_parietal         0.011136   0.012124   0.919    0.361
Task3back:EF_mag                    0.065065   0.148045   0.439    0.662
Stimreal_parietal:EF_mag            0.145021   0.120580   1.203    0.233
Task3back:Stimreal_parietal:EF_mag -0.216634   0.170526  -1.270    0.208

Residual standard error: 0.01155 on 72 degrees of freedom
Multiple R-squared:  0.06926,   Adjusted R-squared:  -0.02123 
F-statistic: 0.7654 on 7 and 72 DF,  p-value: 0.618
```


```
anova(m0)
```


```
Analysis of Variance Table

Response: Acc_n_m
                 Df    Sum Sq    Mean Sq F value Pr(>F)
Task              1 0.0000008 8.1200e-07  0.0061 0.9380
Stim              1 0.0001035 1.0353e-04  0.7764 0.3812
EF_mag            1 0.0000931 9.3132e-05  0.6985 0.4061
Task:Stim         1 0.0000387 3.8741e-05  0.2906 0.5915
Task:EF_mag       1 0.0002383 2.3828e-04  1.7870 0.1855
Stim:EF_mag       1 0.0000247 2.4709e-05  0.1853 0.6681
Task:Stim:EF_mag  1 0.0002152 2.1519e-04  1.6139 0.2080
Residuals        72 0.0096002 1.3334e-04
```


```
m1 <- lmer(formula = Acc_n_m ~ Task*Stim*EF_mag + (1 | ID), data=data_subset)
summary(m1)
```


```
Linear mixed model fit by REML. t-tests use Satterthwaite's method ['lmerModLmerTest']
Formula: Acc_n_m ~ Task * Stim * EF_mag + (1 | ID)
   Data: data_subset

REML criterion at convergence: -442.4

Scaled residuals: 
     Min       1Q   Median       3Q      Max 
-1.97788 -0.54504  0.01586  0.72828  1.85105 

Random effects:
 Groups   Name        Variance  Std.Dev.
 ID       (Intercept) 1.394e-05 0.003734
 Residual             1.196e-04 0.010936
Number of obs: 80, groups:  ID, 20

Fixed effects:
                                    Estimate Std. Error        df t value Pr(>|t|)
(Intercept)                         0.003032   0.007160 71.875670   0.424    0.673
Task3back                          -0.002998   0.009664 51.909555  -0.310    0.758
Stimreal_parietal                  -0.007846   0.008390 68.356664  -0.935    0.353
EF_mag                             -0.002126   0.103737 71.589212  -0.020    0.984
Task3back:Stimreal_parietal         0.011136   0.011482 51.909555   0.970    0.337
Task3back:EF_mag                    0.065065   0.140206 51.909555   0.464    0.645
Stimreal_parietal:EF_mag            0.108442   0.118829 70.397338   0.913    0.365
Task3back:Stimreal_parietal:EF_mag -0.216634   0.161496 51.909555  -1.341    0.186

Correlation of Fixed Effects:
            (Intr) Tsk3bc Stmrl_ EF_mag Ts3:S_ T3:EF_ S_:EF_
Task3back   -0.675                                          
Stimrl_prtl -0.836  0.576                                   
EF_mag      -0.933  0.631  0.790                            
Tsk3bck:St_  0.568 -0.842 -0.684 -0.531                     
Tsk3bck:EF_  0.630 -0.934 -0.538 -0.676  0.786              
Stmrl_p:EF_  0.808 -0.551 -0.911 -0.867  0.615  0.590       
Tsk3:S_:EF_ -0.547  0.811  0.619  0.587 -0.905 -0.868 -0.680
```


```
anova(m1)
```


```
Type III Analysis of Variance Table with Satterthwaite's method
                     Sum Sq    Mean Sq NumDF  DenDF F value Pr(>F)
Task             2.3972e-05 2.3972e-05     1 51.910  0.2005 0.6562
Stim             1.6585e-05 1.6585e-05     1 71.082  0.1387 0.7107
EF_mag           5.6105e-05 5.6105e-05     1 57.213  0.4691 0.4961
Task:Stim        1.1249e-04 1.1249e-04     1 51.910  0.9406 0.3366
Task:EF_mag      3.4312e-05 3.4312e-05     1 51.910  0.2869 0.5945
Stim:EF_mag      0.0000e+00 0.0000e+00     1 67.656  0.0000 0.9989
Task:Stim:EF_mag 2.1519e-04 2.1519e-04     1 51.910  1.7994 0.1856
```


```
AIC(m0, m1)
```


```
BIC(m0, m1)
```


```
m_acc_n_ef <- lm(formula = Acc_n_m ~ Task*Stim*EF_mag, data=data_subset)
summary(m_acc_n_ef)
```


```
Call:
lm(formula = Acc_n_m ~ Task * Stim * EF_mag, data = data_subset)

Residuals:
       Min         1Q     Median         3Q        Max 
-0.0231845 -0.0057477 -0.0006043  0.0090697  0.0217196 

Coefficients:
                                    Estimate Std. Error t value Pr(>|t|)
(Intercept)                         0.004794   0.007216   0.664    0.509
Task3back                          -0.002998   0.010205  -0.294    0.770
Stimreal_parietal                  -0.010199   0.008573  -1.190    0.238
EF_mag                             -0.029495   0.104684  -0.282    0.779
Task3back:Stimreal_parietal         0.011136   0.012124   0.919    0.361
Task3back:EF_mag                    0.065065   0.148045   0.439    0.662
Stimreal_parietal:EF_mag            0.145021   0.120580   1.203    0.233
Task3back:Stimreal_parietal:EF_mag -0.216634   0.170526  -1.270    0.208

Residual standard error: 0.01155 on 72 degrees of freedom
Multiple R-squared:  0.06926,   Adjusted R-squared:  -0.02123 
F-statistic: 0.7654 on 7 and 72 DF,  p-value: 0.618
```


```
anova(m_acc_n_ef)
```


```
Analysis of Variance Table

Response: Acc_n_m
                 Df    Sum Sq    Mean Sq F value Pr(>F)
Task              1 0.0000008 8.1200e-07  0.0061 0.9380
Stim              1 0.0001035 1.0353e-04  0.7764 0.3812
EF_mag            1 0.0000931 9.3132e-05  0.6985 0.4061
Task:Stim         1 0.0000387 3.8741e-05  0.2906 0.5915
Task:EF_mag       1 0.0002383 2.3828e-04  1.7870 0.1855
Stim:EF_mag       1 0.0000247 2.4709e-05  0.1853 0.6681
Task:Stim:EF_mag  1 0.0002152 2.1519e-04  1.6139 0.2080
Residuals        72 0.0096002 1.3334e-04
```


```
eta_squared(m_acc_n_ef)
```


```
# Effect Size for ANOVA (Type I)

Parameter        | Eta2 (partial) |       95% CI
------------------------------------------------
Task             |       8.46e-05 | [0.00, 1.00]
Stim             |           0.01 | [0.00, 1.00]
EF_mag           |       9.61e-03 | [0.00, 1.00]
Task:Stim        |       4.02e-03 | [0.00, 1.00]
Task:EF_mag      |           0.02 | [0.00, 1.00]
Stim:EF_mag      |       2.57e-03 | [0.00, 1.00]
Task:Stim:EF_mag |           0.02 | [0.00, 1.00]

- One-sided CIs: upper bound fixed at [1.00].
```


```
m0 <- lm(formula = Speed_m ~ Task*Stim*EF_mag, data=data_subset)
summary(m0)
```


```
Call:
lm(formula = Speed_m ~ Task * Stim * EF_mag, data = data_subset)

Residuals:
    Min      1Q  Median      3Q     Max 
-42.492  -8.908   0.442   9.209  25.400 

Coefficients:
                                   Estimate Std. Error t value Pr(>|t|)
(Intercept)                           3.232      8.536   0.379    0.706
Task3back                           -17.464     12.072  -1.447    0.152
Stimreal_parietal                    -1.450     10.142  -0.143    0.887
EF_mag                              -83.058    123.840  -0.671    0.505
Task3back:Stimreal_parietal           4.423     14.343   0.308    0.759
Task3back:EF_mag                    193.546    175.136   1.105    0.273
Stimreal_parietal:EF_mag            -76.883    142.645  -0.539    0.592
Task3back:Stimreal_parietal:EF_mag   61.428    201.731   0.305    0.762

Residual standard error: 13.66 on 72 degrees of freedom
Multiple R-squared:  0.1284,    Adjusted R-squared:  0.04371 
F-statistic: 1.516 on 7 and 72 DF,  p-value: 0.1757
```


```
anova(m0)
```


```
Analysis of Variance Table

Response: Speed_m
                 Df  Sum Sq Mean Sq F value   Pr(>F)   
Task              1    14.1   14.05  0.0753 0.784535   
Stim              1    97.2   97.24  0.5211 0.472698   
EF_mag            1    43.9   43.92  0.2354 0.629045   
Task:Stim         1   347.4  347.44  1.8620 0.176649   
Task:EF_mag       1  1421.0 1420.96  7.6150 0.007335 **
Stim:EF_mag       1    39.1   39.09  0.2095 0.648530   
Task:Stim:EF_mag  1    17.3   17.30  0.0927 0.761621   
Residuals        72 13435.2  186.60                    
---
Signif. codes:  0 ‘***’ 0.001 ‘**’ 0.01 ‘*’ 0.05 ‘.’ 0.1 ‘ ’ 1
```


```
m1 <- lmer(formula = Speed_m ~ Task*Stim*EF_mag + (1 | ID), data=data_subset)
summary(m1)
```


```
Linear mixed model fit by REML. t-tests use Satterthwaite's method ['lmerModLmerTest']
Formula: Speed_m ~ Task * Stim * EF_mag + (1 | ID)
   Data: data_subset

REML criterion at convergence: 569.8

Scaled residuals: 
     Min       1Q   Median       3Q      Max 
-2.56139 -0.55822  0.07293  0.61558  1.51378 

Random effects:
 Groups   Name        Variance Std.Dev.
 ID       (Intercept)  54.38    7.374  
 Residual             131.05   11.448  
Number of obs: 80, groups:  ID, 20

Fixed effects:
                                   Estimate Std. Error      df t value Pr(>|t|)  
(Intercept)                           3.786      8.007  71.199   0.473    0.638  
Task3back                           -17.464     10.117  53.835  -1.726    0.090 .
Stimreal_parietal                    -1.225      9.148  65.284  -0.134    0.894  
EF_mag                              -91.657    115.057  69.073  -0.797    0.428  
Task3back:Stimreal_parietal           4.423     12.020  53.835   0.368    0.714  
Task3back:EF_mag                    193.546    146.770  53.835   1.319    0.193  
Stimreal_parietal:EF_mag            -80.407    130.601  67.022  -0.616    0.540  
Task3back:Stimreal_parietal:EF_mag   61.428    169.057  53.835   0.363    0.718  
---
Signif. codes:  0 ‘***’ 0.001 ‘**’ 0.01 ‘*’ 0.05 ‘.’ 0.1 ‘ ’ 1

Correlation of Fixed Effects:
            (Intr) Tsk3bc Stmrl_ EF_mag Ts3:S_ T3:EF_ S_:EF_
Task3back   -0.632                                          
Stimrl_prtl -0.824  0.553                                   
EF_mag      -0.925  0.596  0.794                            
Tsk3bck:St_  0.532 -0.842 -0.657 -0.501                     
Tsk3bck:EF_  0.590 -0.934 -0.516 -0.638  0.786              
Stmrl_p:EF_  0.799 -0.525 -0.918 -0.864  0.586  0.562       
Tsk3:S_:EF_ -0.512  0.811  0.594  0.554 -0.905 -0.868 -0.647
```


```
anova(m1)
```


```
Type III Analysis of Variance Table with Satterthwaite's method
                 Sum Sq Mean Sq NumDF  DenDF F value  Pr(>F)  
Task             844.11  844.11     1 53.835  6.4412 0.01408 *
Stim               2.68    2.68     1 70.923  0.0205 0.88668  
EF_mag            18.68   18.68     1 70.548  0.1425 0.70691  
Task:Stim         17.74   17.74     1 53.835  0.1354 0.71435  
Task:EF_mag      922.42  922.42     1 53.835  7.0388 0.01046 *
Stim:EF_mag       32.65   32.65     1 71.819  0.2491 0.61921  
Task:Stim:EF_mag  17.30   17.30     1 53.835  0.1320 0.71776  
---
Signif. codes:  0 ‘***’ 0.001 ‘**’ 0.01 ‘*’ 0.05 ‘.’ 0.1 ‘ ’ 1
```


```
AIC(m0, m1)
```


```
BIC(m0, m1)
```


```
m_speed_ef <- lmer(formula = Speed_m ~ Task*Stim*EF_mag + (1 | ID), data=data_subset)
summary(m_speed_ef)
```


```
Linear mixed model fit by REML. t-tests use Satterthwaite's method ['lmerModLmerTest']
Formula: Speed_m ~ Task * Stim * EF_mag + (1 | ID)
   Data: data_subset

REML criterion at convergence: 569.8

Scaled residuals: 
     Min       1Q   Median       3Q      Max 
-2.56139 -0.55822  0.07293  0.61558  1.51378 

Random effects:
 Groups   Name        Variance Std.Dev.
 ID       (Intercept)  54.38    7.374  
 Residual             131.05   11.448  
Number of obs: 80, groups:  ID, 20

Fixed effects:
                                   Estimate Std. Error      df t value Pr(>|t|)  
(Intercept)                           3.786      8.007  71.199   0.473    0.638  
Task3back                           -17.464     10.117  53.835  -1.726    0.090 .
Stimreal_parietal                    -1.225      9.148  65.284  -0.134    0.894  
EF_mag                              -91.657    115.057  69.073  -0.797    0.428  
Task3back:Stimreal_parietal           4.423     12.020  53.835   0.368    0.714  
Task3back:EF_mag                    193.546    146.770  53.835   1.319    0.193  
Stimreal_parietal:EF_mag            -80.407    130.601  67.022  -0.616    0.540  
Task3back:Stimreal_parietal:EF_mag   61.428    169.057  53.835   0.363    0.718  
---
Signif. codes:  0 ‘***’ 0.001 ‘**’ 0.01 ‘*’ 0.05 ‘.’ 0.1 ‘ ’ 1

Correlation of Fixed Effects:
            (Intr) Tsk3bc Stmrl_ EF_mag Ts3:S_ T3:EF_ S_:EF_
Task3back   -0.632                                          
Stimrl_prtl -0.824  0.553                                   
EF_mag      -0.925  0.596  0.794                            
Tsk3bck:St_  0.532 -0.842 -0.657 -0.501                     
Tsk3bck:EF_  0.590 -0.934 -0.516 -0.638  0.786              
Stmrl_p:EF_  0.799 -0.525 -0.918 -0.864  0.586  0.562       
Tsk3:S_:EF_ -0.512  0.811  0.594  0.554 -0.905 -0.868 -0.647
```


```
anova(m_speed_ef)
```


```
Type III Analysis of Variance Table with Satterthwaite's method
                 Sum Sq Mean Sq NumDF  DenDF F value  Pr(>F)  
Task             844.11  844.11     1 53.835  6.4412 0.01408 *
Stim               2.68    2.68     1 70.923  0.0205 0.88668  
EF_mag            18.68   18.68     1 70.548  0.1425 0.70691  
Task:Stim         17.74   17.74     1 53.835  0.1354 0.71435  
Task:EF_mag      922.42  922.42     1 53.835  7.0388 0.01046 *
Stim:EF_mag       32.65   32.65     1 71.819  0.2491 0.61921  
Task:Stim:EF_mag  17.30   17.30     1 53.835  0.1320 0.71776  
---
Signif. codes:  0 ‘***’ 0.001 ‘**’ 0.01 ‘*’ 0.05 ‘.’ 0.1 ‘ ’ 1
```


```
eta_squared(m_speed_ef)
```


```
# Effect Size for ANOVA (Type III)

Parameter        | Eta2 (partial) |       95% CI
------------------------------------------------
Task             |           0.11 | [0.01, 1.00]
Stim             |       2.88e-04 | [0.00, 1.00]
EF_mag           |       2.02e-03 | [0.00, 1.00]
Task:Stim        |       2.51e-03 | [0.00, 1.00]
Task:EF_mag      |           0.12 | [0.02, 1.00]
Stim:EF_mag      |       3.46e-03 | [0.00, 1.00]
Task:Stim:EF_mag |       2.45e-03 | [0.00, 1.00]

- One-sided CIs: upper bound fixed at [1.00].
```


```
data_subset <- subset(df_ef_muni, (Stim == "real_frontal" | Stim == "real_parietal") & Task == '2back')
data_subset <- droplevels(data_subset)
levels(data_subset$Stim)
```


```
[1] "real_frontal"  "real_parietal"
```


```
levels(data_subset$Task)
```


```
[1] "2back"
```


```
m_speed_ef_2back <- lmer(formula = Speed_m ~ Stim*EF_mag + (1 | ID), data=data_subset)
summary(m_speed_ef_2back)
```


```
Linear mixed model fit by REML. t-tests use Satterthwaite's method ['lmerModLmerTest']
Formula: Speed_m ~ Stim * EF_mag + (1 | ID)
   Data: data_subset

REML criterion at convergence: 285.6

Scaled residuals: 
     Min       1Q   Median       3Q      Max 
-2.20363 -0.54167 -0.03388  0.47180  1.18196 

Random effects:
 Groups   Name        Variance Std.Dev.
 ID       (Intercept) 141.79   11.908  
 Residual              78.59    8.865  
Number of obs: 40, groups:  ID, 20

Fixed effects:
                         Estimate Std. Error       df t value Pr(>|t|)
(Intercept)                3.9632     7.4350  30.8365   0.533    0.598
Stimreal_parietal         -0.2305     7.9035  22.6993  -0.029    0.977
EF_mag                   -94.4180   103.3592  25.0515  -0.913    0.370
Stimreal_parietal:EF_mag -95.9020   114.8706  23.5286  -0.835    0.412

Correlation of Fixed Effects:
            (Intr) Stmrl_ EF_mag
Stimrl_prtl -0.786              
EF_mag      -0.895  0.803       
Stmrl_p:EF_  0.768 -0.935 -0.859
```


```
anova(m_speed_ef_2back)
```


```
Type III Analysis of Variance Table with Satterthwaite's method
            Sum Sq Mean Sq NumDF  DenDF F value  Pr(>F)  
Stim          0.07    0.07     1 22.699  0.0009 0.97699  
EF_mag      420.48  420.48     1 26.406  5.3506 0.02876 *
Stim:EF_mag  54.78   54.78     1 23.529  0.6970 0.41219  
---
Signif. codes:  0 ‘***’ 0.001 ‘**’ 0.01 ‘*’ 0.05 ‘.’ 0.1 ‘ ’ 1
```


```
eta_squared(m_speed_ef_2back)
```


```
# Effect Size for ANOVA (Type III)

Parameter   | Eta2 (partial) |       95% CI
-------------------------------------------
Stim        |       3.75e-05 | [0.00, 1.00]
EF_mag      |           0.17 | [0.01, 1.00]
Stim:EF_mag |           0.03 | [0.00, 1.00]

- One-sided CIs: upper bound fixed at [1.00].
```


```
data_subset <- subset(df_ef_muni, (Stim == "real_frontal" | Stim == "real_parietal") & Task == '3back')
data_subset <- droplevels(data_subset)
levels(data_subset$Stim)
```


```
[1] "real_frontal"  "real_parietal"
```


```
levels(data_subset$Task)
```


```
[1] "3back"
```


```
m_speed_ef_3back <- lmer(formula = Speed_m ~ Stim*EF_mag + (1 | ID), data=data_subset)
summary(m_speed_ef_3back)
```


```
Linear mixed model fit by REML. t-tests use Satterthwaite's method ['lmerModLmerTest']
Formula: Speed_m ~ Stim * EF_mag + (1 | ID)
   Data: data_subset

REML criterion at convergence: 280.1

Scaled residuals: 
    Min      1Q  Median      3Q     Max 
-1.7297 -0.7075  0.1750  0.6596  1.5165 

Random effects:
 Groups   Name        Variance Std.Dev.
 ID       (Intercept)  22.69    4.763  
 Residual             125.93   11.222  
Number of obs: 40, groups:  ID, 20

Fixed effects:
                         Estimate Std. Error      df t value Pr(>|t|)  
(Intercept)               -14.018      7.541  35.536  -1.859   0.0714 .
Stimreal_parietal           2.818      8.777  31.556   0.321   0.7503  
EF_mag                    107.158    109.243  35.181   0.981   0.3333  
Stimreal_parietal:EF_mag  -13.052    124.797  33.777  -0.105   0.9173  
---
Signif. codes:  0 ‘***’ 0.001 ‘**’ 0.01 ‘*’ 0.05 ‘.’ 0.1 ‘ ’ 1

Correlation of Fixed Effects:
            (Intr) Stmrl_ EF_mag
Stimrl_prtl -0.834              
EF_mag      -0.932  0.792       
Stmrl_p:EF_  0.807 -0.915 -0.866
```


```
anova(m_speed_ef_3back)
```


```
Type III Analysis of Variance Table with Satterthwaite's method
            Sum Sq Mean Sq NumDF  DenDF F value Pr(>F)
Stim         12.98   12.98     1 31.556  0.1031 0.7503
EF_mag      316.96  316.96     1 35.903  2.5171 0.1214
Stim:EF_mag   1.38    1.38     1 33.777  0.0109 0.9173
```


```
data_subset <- subset(df_ef_muni, Stim == "real_frontal" | Stim == "real_parietal")
data_subset <- droplevels(data_subset)
levels(data_subset$Stim)
```


```
[1] "real_frontal"  "real_parietal"
```


```
m0 <- lm(formula = Speed_n_m ~ Task*Stim*EF_mag, data=data_subset)
summary(m0)
```


```
Call:
lm(formula = Speed_n_m ~ Task * Stim * EF_mag, data = data_subset)

Residuals:
      Min        1Q    Median        3Q       Max 
-0.028775 -0.008849 -0.000044  0.008232  0.028886 

Coefficients:
                                    Estimate Std. Error t value Pr(>|t|)
(Intercept)                         0.003609   0.007812   0.462    0.646
Task3back                          -0.015769   0.011048  -1.427    0.158
Stimreal_parietal                  -0.002830   0.009282  -0.305    0.761
EF_mag                             -0.075108   0.113337  -0.663    0.510
Task3back:Stimreal_parietal         0.006051   0.013126   0.461    0.646
Task3back:EF_mag                    0.177266   0.160282   1.106    0.272
Stimreal_parietal:EF_mag           -0.031796   0.130547  -0.244    0.808
Task3back:Stimreal_parietal:EF_mag  0.013517   0.184622   0.073    0.942

Residual standard error: 0.0125 on 72 degrees of freedom
Multiple R-squared:  0.09471,   Adjusted R-squared:  0.006694 
F-statistic: 1.076 on 7 and 72 DF,  p-value: 0.3877
```


```
anova(m0)
```


```
Analysis of Variance Table

Response: Speed_n_m
                 Df    Sum Sq    Mean Sq F value  Pr(>F)  
Task              1 0.0000167 0.00001668  0.1067 0.74484  
Stim              1 0.0000400 0.00003999  0.2559 0.61451  
EF_mag            1 0.0000028 0.00000282  0.0181 0.89345  
Task:Stim         1 0.0002374 0.00023742  1.5191 0.22176  
Task:EF_mag       1 0.0008680 0.00086799  5.5537 0.02117 *
Stim:EF_mag       1 0.0000115 0.00001150  0.0736 0.78699  
Task:Stim:EF_mag  1 0.0000008 0.00000084  0.0054 0.94184  
Residuals        72 0.0112529 0.00015629                  
---
Signif. codes:  0 ‘***’ 0.001 ‘**’ 0.01 ‘*’ 0.05 ‘.’ 0.1 ‘ ’ 1
```


```
m1 <- lmer(formula = Speed_n_m ~ Task*Stim*EF_mag + (1 | ID), data=data_subset)
summary(m1)
```


```
Linear mixed model fit by REML. t-tests use Satterthwaite's method ['lmerModLmerTest']
Formula: Speed_n_m ~ Task * Stim * EF_mag + (1 | ID)
   Data: data_subset

REML criterion at convergence: -437.3

Scaled residuals: 
     Min       1Q   Median       3Q      Max 
-1.81507 -0.52314 -0.05321  0.55122  2.09272 

Random effects:
 Groups   Name        Variance  Std.Dev.
 ID       (Intercept) 4.407e-05 0.006639
 Residual             1.112e-04 0.010544
Number of obs: 80, groups:  ID, 20

Fixed effects:
                                    Estimate Std. Error        df t value Pr(>|t|)  
(Intercept)                         0.004102   0.007354 71.243510   0.558   0.5787  
Task3back                          -0.015769   0.009319 53.926893  -1.692   0.0964 .
Stimreal_parietal                  -0.003065   0.008413 65.508321  -0.364   0.7168  
EF_mag                             -0.082771   0.105729 69.265402  -0.783   0.4364  
Task3back:Stimreal_parietal         0.006051   0.011071 53.926893   0.547   0.5869  
Task3back:EF_mag                    0.177266   0.135190 53.926893   1.311   0.1953  
Stimreal_parietal:EF_mag           -0.028153   0.120064 67.253750  -0.234   0.8153  
Task3back:Stimreal_parietal:EF_mag  0.013517   0.155719 53.926893   0.087   0.9311  
---
Signif. codes:  0 ‘***’ 0.001 ‘**’ 0.01 ‘*’ 0.05 ‘.’ 0.1 ‘ ’ 1

Correlation of Fixed Effects:
            (Intr) Tsk3bc Stmrl_ EF_mag Ts3:S_ T3:EF_ S_:EF_
Task3back   -0.634                                          
Stimrl_prtl -0.825  0.554                                   
EF_mag      -0.925  0.597  0.794                            
Tsk3bck:St_  0.533 -0.842 -0.658 -0.502                     
Tsk3bck:EF_  0.592 -0.934 -0.517 -0.639  0.786              
Stmrl_p:EF_  0.800 -0.526 -0.918 -0.864  0.587  0.563       
Tsk3:S_:EF_ -0.514  0.811  0.595  0.555 -0.905 -0.868 -0.648
```


```
anova(m1)
```


```
Type III Analysis of Variance Table with Satterthwaite's method
                     Sum Sq    Mean Sq NumDF  DenDF F value  Pr(>F)  
Task             0.00058918 0.00058918     1 53.927  5.2991 0.02523 *
Stim             0.00000000 0.00000000     1 71.091  0.0000 0.99505  
EF_mag           0.00000113 0.00000113     1 70.261  0.0102 0.91992  
Task:Stim        0.00003322 0.00003322     1 53.927  0.2987 0.58693  
Task:EF_mag      0.00062113 0.00062113     1 53.927  5.5864 0.02173 *
Stim:EF_mag      0.00000609 0.00000609     1 71.894  0.0548 0.81558  
Task:Stim:EF_mag 0.00000084 0.00000084     1 53.927  0.0075 0.93115  
---
Signif. codes:  0 ‘***’ 0.001 ‘**’ 0.01 ‘*’ 0.05 ‘.’ 0.1 ‘ ’ 1
```


```
AIC(m0, m1)
```


```
BIC(m0, m1)
```


```
m_speed_n_ef <- lm(formula = Speed_n_m ~ Task*Stim*EF_mag, data=data_subset)
summary(m_speed_n_ef)
```


```
Call:
lm(formula = Speed_n_m ~ Task * Stim * EF_mag, data = data_subset)

Residuals:
      Min        1Q    Median        3Q       Max 
-0.028775 -0.008849 -0.000044  0.008232  0.028886 

Coefficients:
                                    Estimate Std. Error t value Pr(>|t|)
(Intercept)                         0.003609   0.007812   0.462    0.646
Task3back                          -0.015769   0.011048  -1.427    0.158
Stimreal_parietal                  -0.002830   0.009282  -0.305    0.761
EF_mag                             -0.075108   0.113337  -0.663    0.510
Task3back:Stimreal_parietal         0.006051   0.013126   0.461    0.646
Task3back:EF_mag                    0.177266   0.160282   1.106    0.272
Stimreal_parietal:EF_mag           -0.031796   0.130547  -0.244    0.808
Task3back:Stimreal_parietal:EF_mag  0.013517   0.184622   0.073    0.942

Residual standard error: 0.0125 on 72 degrees of freedom
Multiple R-squared:  0.09471,   Adjusted R-squared:  0.006694 
F-statistic: 1.076 on 7 and 72 DF,  p-value: 0.3877
```


```
anova(m_speed_n_ef)
```


```
Analysis of Variance Table

Response: Speed_n_m
                 Df    Sum Sq    Mean Sq F value  Pr(>F)  
Task              1 0.0000167 0.00001668  0.1067 0.74484  
Stim              1 0.0000400 0.00003999  0.2559 0.61451  
EF_mag            1 0.0000028 0.00000282  0.0181 0.89345  
Task:Stim         1 0.0002374 0.00023742  1.5191 0.22176  
Task:EF_mag       1 0.0008680 0.00086799  5.5537 0.02117 *
Stim:EF_mag       1 0.0000115 0.00001150  0.0736 0.78699  
Task:Stim:EF_mag  1 0.0000008 0.00000084  0.0054 0.94184  
Residuals        72 0.0112529 0.00015629                  
---
Signif. codes:  0 ‘***’ 0.001 ‘**’ 0.01 ‘*’ 0.05 ‘.’ 0.1 ‘ ’ 1
```


```
data_subset <- subset(df_ef_muni, (Stim == "real_frontal" | Stim == "real_parietal") & Task == '2back')
data_subset <- droplevels(data_subset)
levels(data_subset$Stim)
```


```
[1] "real_frontal"  "real_parietal"
```


```
levels(data_subset$Task)
```


```
[1] "2back"
```


```
m_speed_n_ef_2back <- lm(formula = Speed_n_m ~ Stim*EF_mag, data=data_subset)
summary(m_speed_n_ef_2back)
```


```
Call:
lm(formula = Speed_n_m ~ Stim * EF_mag, data = data_subset)

Residuals:
      Min        1Q    Median        3Q       Max 
-0.028775 -0.009113 -0.002905  0.010357  0.028886 

Coefficients:
                          Estimate Std. Error t value Pr(>|t|)
(Intercept)               0.003609   0.008721   0.414    0.681
Stimreal_parietal        -0.002830   0.010361  -0.273    0.786
EF_mag                   -0.075108   0.126519  -0.594    0.556
Stimreal_parietal:EF_mag -0.031796   0.145731  -0.218    0.829

Residual standard error: 0.01396 on 36 degrees of freedom
Multiple R-squared:  0.09434,   Adjusted R-squared:  0.01887 
F-statistic:  1.25 on 3 and 36 DF,  p-value: 0.3061
```


```
anova(m_speed_n_ef_2back)
```


```
Analysis of Variance Table

Response: Speed_n_m
            Df    Sum Sq    Mean Sq F value Pr(>F)
Stim         1 0.0002361 0.00023615  1.2125 0.2781
EF_mag       1 0.0004849 0.00048491  2.4898 0.1233
Stim:EF_mag  1 0.0000093 0.00000927  0.0476 0.8285
Residuals   36 0.0070113 0.00019476
```


```
data_subset <- subset(df_ef_muni, (Stim == "real_frontal" | Stim == "real_parietal") & Task == '3back')
data_subset <- droplevels(data_subset)
levels(data_subset$Stim)
```


```
[1] "real_frontal"  "real_parietal"
```


```
levels(data_subset$Task)
```


```
[1] "3back"
```


```
m_speed_n_ef_3back <- lm(formula = Speed_n_m ~ Stim*EF_mag, data=data_subset)
summary(m_speed_n_ef_3back)
```


```
Call:
lm(formula = Speed_n_m ~ Stim * EF_mag, data = data_subset)

Residuals:
       Min         1Q     Median         3Q        Max 
-0.0158199 -0.0079429  0.0004544  0.0063760  0.0226718 

Coefficients:
                          Estimate Std. Error t value Pr(>|t|)  
(Intercept)              -0.012160   0.006783  -1.793   0.0814 .
Stimreal_parietal         0.003221   0.008059   0.400   0.6917  
EF_mag                    0.102158   0.098405   1.038   0.3061  
Stimreal_parietal:EF_mag -0.018279   0.113348  -0.161   0.8728  
---
Signif. codes:  0 ‘***’ 0.001 ‘**’ 0.01 ‘*’ 0.05 ‘.’ 0.1 ‘ ’ 1

Residual standard error: 0.01085 on 36 degrees of freedom
Multiple R-squared:  0.09209,   Adjusted R-squared:  0.01643 
F-statistic: 1.217 on 3 and 36 DF,  p-value: 0.3175
```


```
anova(m_speed_n_ef_3back)
```


```
Analysis of Variance Table

Response: Speed_n_m
            Df    Sum Sq    Mean Sq F value  Pr(>F)  
Stim         1 0.0000413 0.00004127  0.3502 0.55767  
EF_mag       1 0.0003859 0.00038590  3.2753 0.07869 .
Stim:EF_mag  1 0.0000031 0.00000306  0.0260 0.87279  
Residuals   36 0.0042415 0.00011782                  
---
Signif. codes:  0 ‘***’ 0.001 ‘**’ 0.01 ‘*’ 0.05 ‘.’ 0.1 ‘ ’ 1
```

### Bifocal conditions


```
data_subset <- subset(df_ef_muni, Stim == "real_in-phase" | Stim == "real_out-of-phase")
data_subset <- droplevels(data_subset)
levels(data_subset$Stim)
```


```
[1] "real_in-phase"     "real_out-of-phase"
```


We will now choose the model following the same criteria as we
applied to test all the previous aspects.


```
m0 <- lm(formula = Acc_m ~ Task*Stim*EF_front*EF_par, data=data_subset)
summary(m0)
```


```
Call:
lm(formula = Acc_m ~ Task * Stim * EF_front * EF_par, data = data_subset)

Residuals:
      Min        1Q    Median        3Q       Max 
-0.017558 -0.004856  0.000220  0.003644  0.018649 

Coefficients:
                                                 Estimate Std. Error t value Pr(>|t|)  
(Intercept)                                      0.006694   0.009091   0.736   0.4642  
Task3back                                        0.004043   0.012856   0.314   0.7542  
Stimreal_out-of-phase                           -0.023397   0.012856  -1.820   0.0734 .
EF_front                                        -0.033267   0.146399  -0.227   0.8210  
EF_par                                           0.001812   0.115380   0.016   0.9875  
Task3back:Stimreal_out-of-phase                  0.013891   0.018181   0.764   0.4477  
Task3back:EF_front                              -0.184230   0.207040  -0.890   0.3769  
Stimreal_out-of-phase:EF_front                   0.272229   0.207040   1.315   0.1932  
Task3back:EF_par                                 0.008749   0.163172   0.054   0.9574  
Stimreal_out-of-phase:EF_par                     0.138985   0.163172   0.852   0.3975  
EF_front:EF_par                                 -0.102873   1.818301  -0.057   0.9551  
Task3back:Stimreal_out-of-phase:EF_front        -0.131254   0.292799  -0.448   0.6555  
Task3back:Stimreal_out-of-phase:EF_par          -0.226392   0.230760  -0.981   0.3303  
Task3back:EF_front:EF_par                        0.515472   2.571466   0.200   0.8418  
Stimreal_out-of-phase:EF_front:EF_par           -1.281627   2.571466  -0.498   0.6199  
Task3back:Stimreal_out-of-phase:EF_front:EF_par  1.990231   3.636602   0.547   0.5861  
---
Signif. codes:  0 ‘***’ 0.001 ‘**’ 0.01 ‘*’ 0.05 ‘.’ 0.1 ‘ ’ 1

Residual standard error: 0.007392 on 64 degrees of freedom
Multiple R-squared:  0.3209,    Adjusted R-squared:  0.1618 
F-statistic: 2.016 on 15 and 64 DF,  p-value: 0.02751
```


```
anova(m0)
```


```
Analysis of Variance Table

Response: Acc_m
                          Df    Sum Sq    Mean Sq F value   Pr(>F)   
Task                       1 0.0005740 0.00057398 10.5040 0.001892 **
Stim                       1 0.0001479 0.00014794  2.7074 0.104785   
EF_front                   1 0.0000140 0.00001397  0.2557 0.614852   
EF_par                     1 0.0000573 0.00005734  1.0494 0.309505   
Task:Stim                  1 0.0000018 0.00000183  0.0334 0.855513   
Task:EF_front              1 0.0002559 0.00025591  4.6832 0.034198 * 
Stim:EF_front              1 0.0004301 0.00043014  7.8716 0.006644 **
Task:EF_par                1 0.0000073 0.00000731  0.1337 0.715798   
Stim:EF_par                1 0.0000027 0.00000275  0.0503 0.823298   
EF_front:EF_par            1 0.0000000 0.00000001  0.0002 0.989853   
Task:Stim:EF_front         1 0.0000005 0.00000047  0.0087 0.926099   
Task:Stim:EF_par           1 0.0001057 0.00010568  1.9339 0.169150   
Task:EF_front:EF_par       1 0.0000377 0.00003771  0.6902 0.409193   
Stim:EF_front:EF_par       1 0.0000014 0.00000136  0.0248 0.875291   
Task:Stim:EF_front:EF_par  1 0.0000164 0.00001637  0.2995 0.586091   
Residuals                 64 0.0034972 0.00005464                    
---
Signif. codes:  0 ‘***’ 0.001 ‘**’ 0.01 ‘*’ 0.05 ‘.’ 0.1 ‘ ’ 1
```


```
m1 <- lmer(formula = Acc_m ~ Task*Stim*EF_front*EF_par + (1 | ID), data=data_subset)
```


```
boundary (singular) fit: see help('isSingular')
```


```
summary(m1)
```


```
Linear mixed model fit by REML. t-tests use Satterthwaite's method ['lmerModLmerTest']
Formula: Acc_m ~ Task * Stim * EF_front * EF_par + (1 | ID)
   Data: data_subset

REML criterion at convergence: -509.4

Scaled residuals: 
     Min       1Q   Median       3Q      Max 
-2.37526 -0.65694  0.02977  0.49300  2.52288 

Random effects:
 Groups   Name        Variance  Std.Dev.
 ID       (Intercept) 0.000e+00 0.000000
 Residual             5.464e-05 0.007392
Number of obs: 80, groups:  ID, 20

Fixed effects:
                                                 Estimate Std. Error        df t value Pr(>|t|)  
(Intercept)                                      0.006694   0.009091 64.000000   0.736   0.4642  
Task3back                                        0.004043   0.012856 64.000000   0.314   0.7542  
Stimreal_out-of-phase                           -0.023397   0.012856 64.000000  -1.820   0.0734 .
EF_front                                        -0.033267   0.146399 64.000000  -0.227   0.8210  
EF_par                                           0.001812   0.115380 64.000000   0.016   0.9875  
Task3back:Stimreal_out-of-phase                  0.013891   0.018181 64.000000   0.764   0.4477  
Task3back:EF_front                              -0.184230   0.207040 64.000000  -0.890   0.3769  
Stimreal_out-of-phase:EF_front                   0.272229   0.207040 64.000000   1.315   0.1932  
Task3back:EF_par                                 0.008749   0.163172 64.000000   0.054   0.9574  
Stimreal_out-of-phase:EF_par                     0.138985   0.163172 64.000000   0.852   0.3975  
EF_front:EF_par                                 -0.102873   1.818301 64.000000  -0.057   0.9551  
Task3back:Stimreal_out-of-phase:EF_front        -0.131254   0.292799 64.000000  -0.448   0.6555  
Task3back:Stimreal_out-of-phase:EF_par          -0.226392   0.230760 64.000000  -0.981   0.3303  
Task3back:EF_front:EF_par                        0.515472   2.571466 64.000000   0.200   0.8418  
Stimreal_out-of-phase:EF_front:EF_par           -1.281627   2.571466 64.000000  -0.498   0.6199  
Task3back:Stimreal_out-of-phase:EF_front:EF_par  1.990231   3.636602 64.000000   0.547   0.5861  
---
Signif. codes:  0 ‘***’ 0.001 ‘**’ 0.01 ‘*’ 0.05 ‘.’ 0.1 ‘ ’ 1
```


```
Correlation matrix not shown by default, as p = 16 > 12.
Use print(x, correlation=TRUE)  or
    vcov(x)        if you need it
```


```
optimizer (nloptwrap) convergence code: 0 (OK)
boundary (singular) fit: see help('isSingular')
```


```
anova(m1)
```


```
Type III Analysis of Variance Table with Satterthwaite's method
                              Sum Sq    Mean Sq NumDF DenDF F value  Pr(>F)  
Task                      7.9840e-05 7.9840e-05     1    64  1.4611 0.23120  
Stim                      1.7897e-04 1.7897e-04     1    64  3.2752 0.07503 .
EF_front                  4.9720e-06 4.9720e-06     1    64  0.0910 0.76390  
EF_par                    5.9780e-06 5.9780e-06     1    64  0.1094 0.74191  
Task:Stim                 3.1898e-05 3.1898e-05     1    64  0.5837 0.44766  
Task:EF_front             1.5916e-04 1.5916e-04     1    64  2.9128 0.09273 .
Stim:EF_front             1.0883e-04 1.0883e-04     1    64  1.9915 0.16302  
Task:EF_par               4.4779e-05 4.4779e-05     1    64  0.8195 0.36873  
Stim:EF_par               2.7300e-06 2.7300e-06     1    64  0.0500 0.82384  
EF_front:EF_par           9.0000e-09 9.0000e-09     1    64  0.0002 0.98985  
Task:Stim:EF_front        1.0981e-05 1.0981e-05     1    64  0.2010 0.65547  
Task:Stim:EF_par          5.2595e-05 5.2595e-05     1    64  0.9625 0.33025  
Task:EF_front:EF_par      3.7714e-05 3.7714e-05     1    64  0.6902 0.40919  
Stim:EF_front:EF_par      1.3570e-06 1.3570e-06     1    64  0.0248 0.87529  
Task:Stim:EF_front:EF_par 1.6367e-05 1.6367e-05     1    64  0.2995 0.58609  
---
Signif. codes:  0 ‘***’ 0.001 ‘**’ 0.01 ‘*’ 0.05 ‘.’ 0.1 ‘ ’ 1
```


```
m_acc_ef <- lm(formula = Acc_m ~ Task*Stim*EF_front*EF_par, data=data_subset)
summary(m_acc_ef)
```


```
Call:
lm(formula = Acc_m ~ Task * Stim * EF_front * EF_par, data = data_subset)

Residuals:
      Min        1Q    Median        3Q       Max 
-0.017558 -0.004856  0.000220  0.003644  0.018649 

Coefficients:
                                                 Estimate Std. Error t value Pr(>|t|)  
(Intercept)                                      0.006694   0.009091   0.736   0.4642  
Task3back                                        0.004043   0.012856   0.314   0.7542  
Stimreal_out-of-phase                           -0.023397   0.012856  -1.820   0.0734 .
EF_front                                        -0.033267   0.146399  -0.227   0.8210  
EF_par                                           0.001812   0.115380   0.016   0.9875  
Task3back:Stimreal_out-of-phase                  0.013891   0.018181   0.764   0.4477  
Task3back:EF_front                              -0.184230   0.207040  -0.890   0.3769  
Stimreal_out-of-phase:EF_front                   0.272229   0.207040   1.315   0.1932  
Task3back:EF_par                                 0.008749   0.163172   0.054   0.9574  
Stimreal_out-of-phase:EF_par                     0.138985   0.163172   0.852   0.3975  
EF_front:EF_par                                 -0.102873   1.818301  -0.057   0.9551  
Task3back:Stimreal_out-of-phase:EF_front        -0.131254   0.292799  -0.448   0.6555  
Task3back:Stimreal_out-of-phase:EF_par          -0.226392   0.230760  -0.981   0.3303  
Task3back:EF_front:EF_par                        0.515472   2.571466   0.200   0.8418  
Stimreal_out-of-phase:EF_front:EF_par           -1.281627   2.571466  -0.498   0.6199  
Task3back:Stimreal_out-of-phase:EF_front:EF_par  1.990231   3.636602   0.547   0.5861  
---
Signif. codes:  0 ‘***’ 0.001 ‘**’ 0.01 ‘*’ 0.05 ‘.’ 0.1 ‘ ’ 1

Residual standard error: 0.007392 on 64 degrees of freedom
Multiple R-squared:  0.3209,    Adjusted R-squared:  0.1618 
F-statistic: 2.016 on 15 and 64 DF,  p-value: 0.02751
```


```
anova(m_acc_ef)
```


```
Analysis of Variance Table

Response: Acc_m
                          Df    Sum Sq    Mean Sq F value   Pr(>F)   
Task                       1 0.0005740 0.00057398 10.5040 0.001892 **
Stim                       1 0.0001479 0.00014794  2.7074 0.104785   
EF_front                   1 0.0000140 0.00001397  0.2557 0.614852   
EF_par                     1 0.0000573 0.00005734  1.0494 0.309505   
Task:Stim                  1 0.0000018 0.00000183  0.0334 0.855513   
Task:EF_front              1 0.0002559 0.00025591  4.6832 0.034198 * 
Stim:EF_front              1 0.0004301 0.00043014  7.8716 0.006644 **
Task:EF_par                1 0.0000073 0.00000731  0.1337 0.715798   
Stim:EF_par                1 0.0000027 0.00000275  0.0503 0.823298   
EF_front:EF_par            1 0.0000000 0.00000001  0.0002 0.989853   
Task:Stim:EF_front         1 0.0000005 0.00000047  0.0087 0.926099   
Task:Stim:EF_par           1 0.0001057 0.00010568  1.9339 0.169150   
Task:EF_front:EF_par       1 0.0000377 0.00003771  0.6902 0.409193   
Stim:EF_front:EF_par       1 0.0000014 0.00000136  0.0248 0.875291   
Task:Stim:EF_front:EF_par  1 0.0000164 0.00001637  0.2995 0.586091   
Residuals                 64 0.0034972 0.00005464                    
---
Signif. codes:  0 ‘***’ 0.001 ‘**’ 0.01 ‘*’ 0.05 ‘.’ 0.1 ‘ ’ 1
```


```
eta_squared(m_acc_ef)
```


```
# Effect Size for ANOVA (Type I)

Parameter                 | Eta2 (partial) |       95% CI
---------------------------------------------------------
Task                      |           0.14 | [0.03, 1.00]
Stim                      |           0.04 | [0.00, 1.00]
EF_front                  |       3.98e-03 | [0.00, 1.00]
EF_par                    |           0.02 | [0.00, 1.00]
Task:Stim                 |       5.22e-04 | [0.00, 1.00]
Task:EF_front             |           0.07 | [0.00, 1.00]
Stim:EF_front             |           0.11 | [0.02, 1.00]
Task:EF_par               |       2.09e-03 | [0.00, 1.00]
Stim:EF_par               |       7.85e-04 | [0.00, 1.00]
EF_front:EF_par           |       2.55e-06 | [0.00, 1.00]
Task:Stim:EF_front        |       1.35e-04 | [0.00, 1.00]
Task:Stim:EF_par          |           0.03 | [0.00, 1.00]
Task:EF_front:EF_par      |           0.01 | [0.00, 1.00]
Stim:EF_front:EF_par      |       3.88e-04 | [0.00, 1.00]
Task:Stim:EF_front:EF_par |       4.66e-03 | [0.00, 1.00]

- One-sided CIs: upper bound fixed at [1.00].
```


```
data_subset <- subset(df_ef_muni, (Stim == "real_in-phase" | Stim == "real_out-of-phase") & Task == '2back')
data_subset <- droplevels(data_subset)
levels(data_subset$Stim)
```


```
[1] "real_in-phase"     "real_out-of-phase"
```


```
levels(data_subset$Task)
```


```
[1] "2back"
```


```
m_acc_ef_2back <- lm(formula = Acc_m ~ Stim*EF_front*EF_par, data=data_subset)
summary(m_acc_ef_2back)
```


```
Call:
lm(formula = Acc_m ~ Stim * EF_front * EF_par, data = data_subset)

Residuals:
       Min         1Q     Median         3Q        Max 
-0.0175270 -0.0048994  0.0002644  0.0044714  0.0186495 

Coefficients:
                                       Estimate Std. Error t value Pr(>|t|)  
(Intercept)                            0.006694   0.009339   0.717    0.479  
Stimreal_out-of-phase                 -0.023397   0.013207  -1.772    0.086 .
EF_front                              -0.033267   0.150396  -0.221    0.826  
EF_par                                 0.001812   0.118530   0.015    0.988  
Stimreal_out-of-phase:EF_front         0.272229   0.212692   1.280    0.210  
Stimreal_out-of-phase:EF_par           0.138985   0.167626   0.829    0.413  
EF_front:EF_par                       -0.102873   1.867936  -0.055    0.956  
Stimreal_out-of-phase:EF_front:EF_par -1.281627   2.641660  -0.485    0.631  
---
Signif. codes:  0 ‘***’ 0.001 ‘**’ 0.01 ‘*’ 0.05 ‘.’ 0.1 ‘ ’ 1

Residual standard error: 0.007594 on 32 degrees of freedom
Multiple R-squared:  0.2195,    Adjusted R-squared:  0.04881 
F-statistic: 1.286 on 7 and 32 DF,  p-value: 0.2886
```


```
anova(m_acc_ef_2back)
```


```
Analysis of Variance Table

Response: Acc_m
                     Df     Sum Sq    Mean Sq F value  Pr(>F)  
Stim                  1 0.00005845 5.8447e-05  1.0135 0.32162  
EF_front              1 0.00007515 7.5146e-05  1.3031 0.26212  
EF_par                1 0.00005280 5.2796e-05  0.9155 0.34583  
Stim:EF_front         1 0.00022958 2.2958e-04  3.9811 0.05459 .
Stim:EF_par           1 0.00007125 7.1250e-05  1.2355 0.27462  
EF_front:EF_par       1 0.00001828 1.8282e-05  0.3170 0.57733  
Stim:EF_front:EF_par  1 0.00001357 1.3574e-05  0.2354 0.63087  
Residuals            32 0.00184537 5.7668e-05                  
---
Signif. codes:  0 ‘***’ 0.001 ‘**’ 0.01 ‘*’ 0.05 ‘.’ 0.1 ‘ ’ 1
```


```
eta_squared(m_acc_ef_2back)
```


```
# Effect Size for ANOVA (Type I)

Parameter            | Eta2 (partial) |       95% CI
----------------------------------------------------
Stim                 |           0.03 | [0.00, 1.00]
EF_front             |           0.04 | [0.00, 1.00]
EF_par               |           0.03 | [0.00, 1.00]
Stim:EF_front        |           0.11 | [0.00, 1.00]
Stim:EF_par          |           0.04 | [0.00, 1.00]
EF_front:EF_par      |       9.81e-03 | [0.00, 1.00]
Stim:EF_front:EF_par |       7.30e-03 | [0.00, 1.00]

- One-sided CIs: upper bound fixed at [1.00].
```


```
data_subset <- subset(df_ef_muni, (Stim == "real_in-phase" | Stim == "real_out-of-phase") & Task == '3back')
data_subset <- droplevels(data_subset)
levels(data_subset$Stim)
```


```
[1] "real_in-phase"     "real_out-of-phase"
```


```
levels(data_subset$Task)
```


```
[1] "3back"
```


```
m_acc_ef_3back <- lm(formula = Acc_m ~ Stim*EF_front*EF_par, data=data_subset)
summary(m_acc_ef_3back)
```


```
Call:
lm(formula = Acc_m ~ Stim * EF_front * EF_par, data = data_subset)

Residuals:
       Min         1Q     Median         3Q        Max 
-0.0175583 -0.0045203  0.0000099  0.0025308  0.0146618 

Coefficients:
                                       Estimate Std. Error t value Pr(>|t|)
(Intercept)                            0.010737   0.008835   1.215    0.233
Stimreal_out-of-phase                 -0.009506   0.012495  -0.761    0.452
EF_front                              -0.217496   0.142291  -1.529    0.136
EF_par                                 0.010561   0.112142   0.094    0.926
Stimreal_out-of-phase:EF_front         0.140975   0.201230   0.701    0.489
Stimreal_out-of-phase:EF_par          -0.087406   0.158593  -0.551    0.585
EF_front:EF_par                        0.412599   1.767273   0.233    0.817
Stimreal_out-of-phase:EF_front:EF_par  0.708604   2.499301   0.284    0.779

Residual standard error: 0.007185 on 32 degrees of freedom
Multiple R-squared:  0.2531,    Adjusted R-squared:  0.08969 
F-statistic: 1.549 on 7 and 32 DF,  p-value: 0.1867
```


```
anova(m_acc_ef_3back)
```


```
Analysis of Variance Table

Response: Acc_m
                     Df     Sum Sq    Mean Sq F value  Pr(>F)  
Stim                  1 0.00009132 9.1324e-05  1.7692 0.19289  
EF_front              1 0.00019473 1.9473e-04  3.7724 0.06095 .
EF_par                1 0.00001185 1.1855e-05  0.2297 0.63504  
Stim:EF_front         1 0.00020103 2.0103e-04  3.8944 0.05714 .
Stim:EF_par           1 0.00003717 3.7173e-05  0.7201 0.40241  
EF_front:EF_par       1 0.00001944 1.9441e-05  0.3766 0.54376  
Stim:EF_front:EF_par  1 0.00000415 4.1490e-06  0.0804 0.77860  
Residuals            32 0.00165184 5.1620e-05                  
---
Signif. codes:  0 ‘***’ 0.001 ‘**’ 0.01 ‘*’ 0.05 ‘.’ 0.1 ‘ ’ 1
```


```
eta_squared(m_acc_ef_3back)
```


```
# Effect Size for ANOVA (Type I)

Parameter            | Eta2 (partial) |       95% CI
----------------------------------------------------
Stim                 |           0.05 | [0.00, 1.00]
EF_front             |           0.11 | [0.00, 1.00]
EF_par               |       7.13e-03 | [0.00, 1.00]
Stim:EF_front        |           0.11 | [0.00, 1.00]
Stim:EF_par          |           0.02 | [0.00, 1.00]
EF_front:EF_par      |           0.01 | [0.00, 1.00]
Stim:EF_front:EF_par |       2.51e-03 | [0.00, 1.00]

- One-sided CIs: upper bound fixed at [1.00].
```

### The relationship between the intensity of the applied electric field on the target and the normalized accuracy


```
data_subset <- subset(df_ef_muni, Stim == "real_in-phase" | Stim == "real_out-of-phase")
data_subset <- droplevels(data_subset)
levels(data_subset$Stim)
```


```
[1] "real_in-phase"     "real_out-of-phase"
```


```
m0 <- lm(formula = Acc_n_m ~ Task*Stim*EF_front*EF_par, data=data_subset)
summary(m0)
```


```
Call:
lm(formula = Acc_n_m ~ Task * Stim * EF_front * EF_par, data = data_subset)

Residuals:
      Min        1Q    Median        3Q       Max 
-0.024744 -0.006586 -0.000798  0.003654  0.103315 

Coefficients:
                                                 Estimate Std. Error t value Pr(>|t|)
(Intercept)                                      0.012026   0.020121   0.598    0.552
Task3back                                        0.004734   0.028455   0.166    0.868
Stimreal_out-of-phase                           -0.033871   0.028455  -1.190    0.238
EF_front                                         0.036660   0.324037   0.113    0.910
EF_par                                           0.038221   0.255380   0.150    0.881
Task3back:Stimreal_out-of-phase                  0.020705   0.040242   0.515    0.609
Task3back:EF_front                              -0.356968   0.458258  -0.779    0.439
Stimreal_out-of-phase:EF_front                   0.276232   0.458258   0.603    0.549
Task3back:EF_par                                -0.076503   0.361161  -0.212    0.833
Stimreal_out-of-phase:EF_par                     0.182981   0.361161   0.507    0.614
EF_front:EF_par                                 -1.543151   4.024588  -0.383    0.703
Task3back:Stimreal_out-of-phase:EF_front        -0.078726   0.648075  -0.121    0.904
Task3back:Stimreal_out-of-phase:EF_par          -0.258512   0.510759  -0.506    0.615
Task3back:EF_front:EF_par                        2.814502   5.691626   0.494    0.623
Stimreal_out-of-phase:EF_front:EF_par           -0.732773   5.691626  -0.129    0.898
Task3back:Stimreal_out-of-phase:EF_front:EF_par  1.160498   8.049175   0.144    0.886

Residual standard error: 0.01636 on 64 degrees of freedom
Multiple R-squared:  0.1899,    Adjusted R-squared:  2.18e-05 
F-statistic:     1 on 15 and 64 DF,  p-value: 0.4663
```


```
anova(m0)
```


```
Analysis of Variance Table

Response: Acc_n_m
                          Df    Sum Sq    Mean Sq F value  Pr(>F)  
Task                       1 0.0016762 0.00167620  6.2614 0.01491 *
Stim                       1 0.0005990 0.00059903  2.2377 0.13960  
EF_front                   1 0.0000612 0.00006121  0.2286 0.63417  
EF_par                     1 0.0000293 0.00002934  0.1096 0.74167  
Task:Stim                  1 0.0000796 0.00007959  0.2973 0.58748  
Task:EF_front              1 0.0002902 0.00029018  1.0840 0.30173  
Stim:EF_front              1 0.0006759 0.00067589  2.5248 0.11700  
Task:EF_par                1 0.0000003 0.00000028  0.0010 0.97430  
Stim:EF_par                1 0.0000729 0.00007293  0.2724 0.60350  
EF_front:EF_par            1 0.0000030 0.00000298  0.0111 0.91636  
Task:Stim:EF_front         1 0.0000042 0.00000417  0.0156 0.90105  
Task:Stim:EF_par           1 0.0003278 0.00032777  1.2244 0.27264  
Task:EF_front:EF_par       1 0.0001905 0.00019047  0.7115 0.40209  
Stim:EF_front:EF_par       1 0.0000004 0.00000038  0.0014 0.96989  
Task:Stim:EF_front:EF_par  1 0.0000056 0.00000556  0.0208 0.88581  
Residuals                 64 0.0171330 0.00026770                  
---
Signif. codes:  0 ‘***’ 0.001 ‘**’ 0.01 ‘*’ 0.05 ‘.’ 0.1 ‘ ’ 1
```


```
m1 <- lmer(formula = Acc_n_m ~ Task*Stim*EF_front*EF_par + (1 | ID), data=data_subset)
```


```
boundary (singular) fit: see help('isSingular')
```


```
summary(m1)
```


```
Linear mixed model fit by REML. t-tests use Satterthwaite's method ['lmerModLmerTest']
Formula: Acc_n_m ~ Task * Stim * EF_front * EF_par + (1 | ID)
   Data: data_subset

REML criterion at convergence: -407.7

Scaled residuals: 
    Min      1Q  Median      3Q     Max 
-1.5123 -0.4025 -0.0488  0.2233  6.3144 

Random effects:
 Groups   Name        Variance  Std.Dev.
 ID       (Intercept) 0.0000000 0.00000 
 Residual             0.0002677 0.01636 
Number of obs: 80, groups:  ID, 20

Fixed effects:
                                                 Estimate Std. Error        df t value Pr(>|t|)
(Intercept)                                      0.012026   0.020121 64.000000   0.598    0.552
Task3back                                        0.004734   0.028455 64.000000   0.166    0.868
Stimreal_out-of-phase                           -0.033871   0.028455 64.000000  -1.190    0.238
EF_front                                         0.036660   0.324037 64.000000   0.113    0.910
EF_par                                           0.038221   0.255380 64.000000   0.150    0.881
Task3back:Stimreal_out-of-phase                  0.020705   0.040242 64.000000   0.515    0.609
Task3back:EF_front                              -0.356968   0.458258 64.000000  -0.779    0.439
Stimreal_out-of-phase:EF_front                   0.276232   0.458258 64.000000   0.603    0.549
Task3back:EF_par                                -0.076503   0.361161 64.000000  -0.212    0.833
Stimreal_out-of-phase:EF_par                     0.182981   0.361161 64.000000   0.507    0.614
EF_front:EF_par                                 -1.543151   4.024588 64.000000  -0.383    0.703
Task3back:Stimreal_out-of-phase:EF_front        -0.078726   0.648075 64.000000  -0.121    0.904
Task3back:Stimreal_out-of-phase:EF_par          -0.258512   0.510759 64.000000  -0.506    0.615
Task3back:EF_front:EF_par                        2.814502   5.691626 64.000000   0.494    0.623
Stimreal_out-of-phase:EF_front:EF_par           -0.732773   5.691626 64.000000  -0.129    0.898
Task3back:Stimreal_out-of-phase:EF_front:EF_par  1.160498   8.049175 64.000000   0.144    0.886
```


```
Correlation matrix not shown by default, as p = 16 > 12.
Use print(x, correlation=TRUE)  or
    vcov(x)        if you need it
```


```
optimizer (nloptwrap) convergence code: 0 (OK)
boundary (singular) fit: see help('isSingular')
```


```
anova(m1)
```


```
Type III Analysis of Variance Table with Satterthwaite's method
                              Sum Sq    Mean Sq NumDF DenDF F value Pr(>F)
Task                      0.00015051 0.00015051     1    64  0.5622 0.4561
Stim                      0.00036573 0.00036573     1    64  1.3662 0.2468
EF_front                  0.00000558 0.00000558     1    64  0.0208 0.8857
EF_par                    0.00001182 0.00001182     1    64  0.0442 0.8342
Task:Stim                 0.00007087 0.00007087     1    64  0.2647 0.6087
Task:EF_front             0.00040048 0.00040048     1    64  1.4960 0.2258
Stim:EF_front             0.00014305 0.00014305     1    64  0.5343 0.4675
Task:EF_par               0.00017378 0.00017378     1    64  0.6491 0.4234
Stim:EF_par               0.00001185 0.00001185     1    64  0.0443 0.8340
EF_front:EF_par           0.00000298 0.00000298     1    64  0.0111 0.9164
Task:Stim:EF_front        0.00000395 0.00000395     1    64  0.0148 0.9037
Task:Stim:EF_par          0.00006858 0.00006858     1    64  0.2562 0.6145
Task:EF_front:EF_par      0.00019047 0.00019047     1    64  0.7115 0.4021
Stim:EF_front:EF_par      0.00000038 0.00000038     1    64  0.0014 0.9699
Task:Stim:EF_front:EF_par 0.00000556 0.00000556     1    64  0.0208 0.8858
```


```
m_acc_n_ef <- lm(formula = Acc_n_m ~ Task*Stim*EF_front*EF_par, data=data_subset)
summary(m_acc_n_ef)
```


```
Call:
lm(formula = Acc_n_m ~ Task * Stim * EF_front * EF_par, data = data_subset)

Residuals:
      Min        1Q    Median        3Q       Max 
-0.024744 -0.006586 -0.000798  0.003654  0.103315 

Coefficients:
                                                 Estimate Std. Error t value Pr(>|t|)
(Intercept)                                      0.012026   0.020121   0.598    0.552
Task3back                                        0.004734   0.028455   0.166    0.868
Stimreal_out-of-phase                           -0.033871   0.028455  -1.190    0.238
EF_front                                         0.036660   0.324037   0.113    0.910
EF_par                                           0.038221   0.255380   0.150    0.881
Task3back:Stimreal_out-of-phase                  0.020705   0.040242   0.515    0.609
Task3back:EF_front                              -0.356968   0.458258  -0.779    0.439
Stimreal_out-of-phase:EF_front                   0.276232   0.458258   0.603    0.549
Task3back:EF_par                                -0.076503   0.361161  -0.212    0.833
Stimreal_out-of-phase:EF_par                     0.182981   0.361161   0.507    0.614
EF_front:EF_par                                 -1.543151   4.024588  -0.383    0.703
Task3back:Stimreal_out-of-phase:EF_front        -0.078726   0.648075  -0.121    0.904
Task3back:Stimreal_out-of-phase:EF_par          -0.258512   0.510759  -0.506    0.615
Task3back:EF_front:EF_par                        2.814502   5.691626   0.494    0.623
Stimreal_out-of-phase:EF_front:EF_par           -0.732773   5.691626  -0.129    0.898
Task3back:Stimreal_out-of-phase:EF_front:EF_par  1.160498   8.049175   0.144    0.886

Residual standard error: 0.01636 on 64 degrees of freedom
Multiple R-squared:  0.1899,    Adjusted R-squared:  2.18e-05 
F-statistic:     1 on 15 and 64 DF,  p-value: 0.4663
```


```
anova(m_acc_n_ef)
```


```
Analysis of Variance Table

Response: Acc_n_m
                          Df    Sum Sq    Mean Sq F value  Pr(>F)  
Task                       1 0.0016762 0.00167620  6.2614 0.01491 *
Stim                       1 0.0005990 0.00059903  2.2377 0.13960  
EF_front                   1 0.0000612 0.00006121  0.2286 0.63417  
EF_par                     1 0.0000293 0.00002934  0.1096 0.74167  
Task:Stim                  1 0.0000796 0.00007959  0.2973 0.58748  
Task:EF_front              1 0.0002902 0.00029018  1.0840 0.30173  
Stim:EF_front              1 0.0006759 0.00067589  2.5248 0.11700  
Task:EF_par                1 0.0000003 0.00000028  0.0010 0.97430  
Stim:EF_par                1 0.0000729 0.00007293  0.2724 0.60350  
EF_front:EF_par            1 0.0000030 0.00000298  0.0111 0.91636  
Task:Stim:EF_front         1 0.0000042 0.00000417  0.0156 0.90105  
Task:Stim:EF_par           1 0.0003278 0.00032777  1.2244 0.27264  
Task:EF_front:EF_par       1 0.0001905 0.00019047  0.7115 0.40209  
Stim:EF_front:EF_par       1 0.0000004 0.00000038  0.0014 0.96989  
Task:Stim:EF_front:EF_par  1 0.0000056 0.00000556  0.0208 0.88581  
Residuals                 64 0.0171330 0.00026770                  
---
Signif. codes:  0 ‘***’ 0.001 ‘**’ 0.01 ‘*’ 0.05 ‘.’ 0.1 ‘ ’ 1
```


```
data_subset <- subset(df_ef_muni, (Stim == "real_in-phase" | Stim == "real_out-of-phase") & Task == '2back')
data_subset <- droplevels(data_subset)
levels(data_subset$Stim)
```


```
[1] "real_in-phase"     "real_out-of-phase"
```


```
levels(data_subset$Task)
```


```
[1] "2back"
```


```
m_acc_n_ef_2back <- lm(formula = Acc_n_m ~ Stim*EF_front*EF_par, data=data_subset)
summary(m_acc_n_ef_2back)
```


```
Call:
lm(formula = Acc_n_m ~ Stim * EF_front * EF_par, data = data_subset)

Residuals:
      Min        1Q    Median        3Q       Max 
-0.022550 -0.007965 -0.002078  0.003778  0.103315 

Coefficients:
                                      Estimate Std. Error t value Pr(>|t|)
(Intercept)                            0.01203    0.02575   0.467    0.644
Stimreal_out-of-phase                 -0.03387    0.03642  -0.930    0.359
EF_front                               0.03666    0.41469   0.088    0.930
EF_par                                 0.03822    0.32682   0.117    0.908
Stimreal_out-of-phase:EF_front         0.27623    0.58646   0.471    0.641
Stimreal_out-of-phase:EF_par           0.18298    0.46220   0.396    0.695
EF_front:EF_par                       -1.54315    5.15050  -0.300    0.766
Stimreal_out-of-phase:EF_front:EF_par -0.73277    7.28391  -0.101    0.920

Residual standard error: 0.02094 on 32 degrees of freedom
Multiple R-squared:  0.09605,   Adjusted R-squared:  -0.1017 
F-statistic: 0.4858 on 7 and 32 DF,  p-value: 0.8377
```


```
anova(m_acc_n_ef_2back)
```


```
Analysis of Variance Table

Response: Acc_n_m
                     Df    Sum Sq    Mean Sq F value Pr(>F)
Stim                  1 0.0005577 0.00055765  1.2719 0.2678
EF_front              1 0.0000424 0.00004242  0.0968 0.7578
EF_par                1 0.0000177 0.00001768  0.0403 0.8421
Stim:EF_front         1 0.0003931 0.00039313  0.8967 0.3508
Stim:EF_par           1 0.0003550 0.00035497  0.8096 0.3750
EF_front:EF_par       1 0.0001205 0.00012053  0.2749 0.6037
Stim:EF_front:EF_par  1 0.0000044 0.00000444  0.0101 0.9205
Residuals            32 0.0140300 0.00043844
```


```
data_subset <- subset(df_ef_muni, (Stim == "real_in-phase" | Stim == "real_out-of-phase") & Task == '3back')
data_subset <- droplevels(data_subset)
levels(data_subset$Stim)
```


```
[1] "real_in-phase"     "real_out-of-phase"
```


```
levels(data_subset$Task)
```


```
[1] "3back"
```


```
m_acc_n_ef_3back <- lm(formula = Acc_n_m ~ Stim*EF_front*EF_par, data=data_subset)
summary(m_acc_n_ef_3back)
```


```
Call:
lm(formula = Acc_n_m ~ Stim * EF_front * EF_par, data = data_subset)

Residuals:
       Min         1Q     Median         3Q        Max 
-0.0247442 -0.0065200 -0.0006081  0.0031840  0.0225153 

Coefficients:
                                      Estimate Std. Error t value Pr(>|t|)
(Intercept)                            0.01676    0.01211   1.384    0.176
Stimreal_out-of-phase                 -0.01317    0.01713  -0.769    0.448
EF_front                              -0.32031    0.19502  -1.642    0.110
EF_par                                -0.03828    0.15370  -0.249    0.805
Stimreal_out-of-phase:EF_front         0.19751    0.27580   0.716    0.479
Stimreal_out-of-phase:EF_par          -0.07553    0.21736  -0.347    0.731
EF_front:EF_par                        1.27135    2.42218   0.525    0.603
Stimreal_out-of-phase:EF_front:EF_par  0.42772    3.42548   0.125    0.901

Residual standard error: 0.009847 on 32 degrees of freedom
Multiple R-squared:  0.2148,    Adjusted R-squared:  0.04307 
F-statistic: 1.251 on 7 and 32 DF,  p-value: 0.3053
```


```
anova(m_acc_n_ef_3back)
```


```
Analysis of Variance Table

Response: Acc_n_m
                     Df     Sum Sq    Mean Sq F value  Pr(>F)  
Stim                  1 0.00012096 1.2097e-04  1.2475 0.27235  
EF_front              1 0.00030897 3.0897e-04  3.1863 0.08374 .
EF_par                1 0.00001195 1.1945e-05  0.1232 0.72790  
Stim:EF_front         1 0.00028693 2.8693e-04  2.9591 0.09505 .
Stim:EF_par           1 0.00004574 4.5739e-05  0.4717 0.49716  
EF_front:EF_par       1 0.00007292 7.2915e-05  0.7520 0.39231  
Stim:EF_front:EF_par  1 0.00000151 1.5120e-06  0.0156 0.90141  
Residuals            32 0.00310294 9.6967e-05                  
---
Signif. codes:  0 ‘***’ 0.001 ‘**’ 0.01 ‘*’ 0.05 ‘.’ 0.1 ‘ ’ 1
```

### The relationship between the intensity of the applied electric field on the target and the speed


```
data_subset <- subset(df_ef_muni, Stim == "real_in-phase" | Stim == "real_out-of-phase")
data_subset <- droplevels(data_subset)
levels(data_subset$Stim)
```


```
[1] "real_in-phase"     "real_out-of-phase"
```


```
m0 <- lm(formula = Speed_m ~ Task*Stim*EF_front*EF_par, data=data_subset)
summary(m0)
```


```
Call:
lm(formula = Speed_m ~ Task * Stim * EF_front * EF_par, data = data_subset)

Residuals:
    Min      1Q  Median      3Q     Max 
-34.922  -6.812  -0.027   5.879  32.036 

Coefficients:
                                                 Estimate Std. Error t value Pr(>|t|)  
(Intercept)                                         1.706     14.798   0.115   0.9086  
Task3back                                           6.930     20.928   0.331   0.7416  
Stimreal_out-of-phase                             -29.166     20.928  -1.394   0.1682  
EF_front                                         -228.370    238.321  -0.958   0.3415  
EF_par                                            -73.355    187.825  -0.391   0.6974  
Task3back:Stimreal_out-of-phase                    20.070     29.597   0.678   0.5002  
Task3back:EF_front                                -95.988    337.037  -0.285   0.7767  
Stimreal_out-of-phase:EF_front                    639.442    337.037   1.897   0.0623 .
Task3back:EF_par                                 -173.439    265.625  -0.653   0.5161  
Stimreal_out-of-phase:EF_par                      396.762    265.625   1.494   0.1402  
EF_front:EF_par                                  3114.404   2959.982   1.052   0.2967  
Task3back:Stimreal_out-of-phase:EF_front         -357.876    476.642  -0.751   0.4555  
Task3back:Stimreal_out-of-phase:EF_par           -205.260    375.650  -0.546   0.5867  
Task3back:EF_front:EF_par                        2030.254   4186.047   0.485   0.6293  
Stimreal_out-of-phase:EF_front:EF_par           -9031.975   4186.047  -2.158   0.0347 *
Task3back:Stimreal_out-of-phase:EF_front:EF_par  4751.644   5919.964   0.803   0.4251  
---
Signif. codes:  0 ‘***’ 0.001 ‘**’ 0.01 ‘*’ 0.05 ‘.’ 0.1 ‘ ’ 1

Residual standard error: 12.03 on 64 degrees of freedom
Multiple R-squared:  0.1764,    Adjusted R-squared:  -0.01667 
F-statistic: 0.9136 on 15 and 64 DF,  p-value: 0.5535
```


```
anova(m0)
```


```
Analysis of Variance Table

Response: Speed_m
                          Df Sum Sq Mean Sq F value  Pr(>F)  
Task                       1    2.3    2.26  0.0156 0.90095  
Stim                       1   20.2   20.17  0.1393 0.71025  
EF_front                   1   17.1   17.10  0.1181 0.73221  
EF_par                     1  178.8  178.76  1.2345 0.27070  
Task:Stim                  1   85.0   84.98  0.5869 0.44645  
Task:EF_front              1   16.7   16.72  0.1155 0.73511  
Stim:EF_front              1   17.2   17.21  0.1188 0.73145  
Task:EF_par                1    5.8    5.82  0.0402 0.84174  
Stim:EF_par                1  395.6  395.62  2.7320 0.10325  
EF_front:EF_par            1   42.5   42.46  0.2933 0.59002  
Task:Stim:EF_front         1    0.0    0.00  0.0000 0.99540  
Task:Stim:EF_par           1   57.0   57.01  0.3937 0.53259  
Task:EF_front:EF_par       1  320.9  320.86  2.2158 0.14152  
Stim:EF_front:EF_par       1  732.2  732.25  5.0567 0.02798 *
Task:Stim:EF_front:EF_par  1   93.3   93.29  0.6442 0.42515  
Residuals                 64 9267.6  144.81                  
---
Signif. codes:  0 ‘***’ 0.001 ‘**’ 0.01 ‘*’ 0.05 ‘.’ 0.1 ‘ ’ 1
```


```
m1 <- lmer(formula = Speed_m ~ Task*Stim*EF_front*EF_par + (1 | ID), data=data_subset)
summary(m1)
```


```
Linear mixed model fit by REML. t-tests use Satterthwaite's method ['lmerModLmerTest']
Formula: Speed_m ~ Task * Stim * EF_front * EF_par + (1 | ID)
   Data: data_subset

REML criterion at convergence: 436.2

Scaled residuals: 
     Min       1Q   Median       3Q      Max 
-2.54585 -0.57369  0.05329  0.48137  2.56670 

Random effects:
 Groups   Name        Variance Std.Dev.
 ID       (Intercept)  15.28    3.909  
 Residual             129.53   11.381  
Number of obs: 80, groups:  ID, 20

Fixed effects:
                                                 Estimate Std. Error        df t value Pr(>|t|)  
(Intercept)                                         1.706     14.798    61.931   0.115   0.9086  
Task3back                                           6.930     19.793    48.000   0.350   0.7278  
Stimreal_out-of-phase                             -29.166     19.793    48.000  -1.474   0.1471  
EF_front                                         -228.370    238.321    61.931  -0.958   0.3417  
EF_par                                            -73.355    187.825    61.931  -0.391   0.6975  
Task3back:Stimreal_out-of-phase                    20.070     27.992    48.000   0.717   0.4769  
Task3back:EF_front                                -95.988    318.757    48.000  -0.301   0.7646  
Stimreal_out-of-phase:EF_front                    639.442    318.757    48.000   2.006   0.0505 .
Task3back:EF_par                                 -173.439    251.218    48.000  -0.690   0.4933  
Stimreal_out-of-phase:EF_par                      396.762    251.218    48.000   1.579   0.1208  
EF_front:EF_par                                  3114.404   2959.982    61.931   1.052   0.2968  
Task3back:Stimreal_out-of-phase:EF_front         -357.876    450.791    48.000  -0.794   0.4312  
Task3back:Stimreal_out-of-phase:EF_par           -205.260    355.276    48.000  -0.578   0.5661  
Task3back:EF_front:EF_par                        2030.254   3959.011    48.000   0.513   0.6104  
Stimreal_out-of-phase:EF_front:EF_par           -9031.975   3959.011    48.000  -2.281   0.0270 *
Task3back:Stimreal_out-of-phase:EF_front:EF_par  4751.644   5598.887    48.000   0.849   0.4003  
---
Signif. codes:  0 ‘***’ 0.001 ‘**’ 0.01 ‘*’ 0.05 ‘.’ 0.1 ‘ ’ 1
```


```
Correlation matrix not shown by default, as p = 16 > 12.
Use print(x, correlation=TRUE)  or
    vcov(x)        if you need it
```


```
anova(m1)
```


```
Type III Analysis of Variance Table with Satterthwaite's method
                          Sum Sq Mean Sq NumDF DenDF F value  Pr(>F)  
Task                      190.30  190.30     1    48  1.4692 0.23140  
Stim                      242.02  242.02     1    48  1.8685 0.17801  
EF_front                   14.73   14.73     1    16  0.1137 0.74031  
EF_par                      1.89    1.89     1    16  0.0146 0.90541  
Task:Stim                  66.59   66.59     1    48  0.5141 0.47685  
Task:EF_front             192.71  192.71     1    48  1.4878 0.22852  
Stim:EF_front             540.67  540.67     1    48  4.1742 0.04655 *
Task:EF_par               312.84  312.84     1    48  2.4153 0.12673  
Stim:EF_par               355.11  355.11     1    48  2.7416 0.10429  
EF_front:EF_par            28.85   28.85     1    16  0.2227 0.64334  
Task:Stim:EF_front         81.63   81.63     1    48  0.6303 0.43117  
Task:Stim:EF_par           43.23   43.23     1    48  0.3338 0.56613  
Task:EF_front:EF_par      320.86  320.86     1    48  2.4772 0.12208  
Stim:EF_front:EF_par      732.25  732.25     1    48  5.6533 0.02145 *
Task:Stim:EF_front:EF_par  93.29   93.29     1    48  0.7203 0.40027  
---
Signif. codes:  0 ‘***’ 0.001 ‘**’ 0.01 ‘*’ 0.05 ‘.’ 0.1 ‘ ’ 1
```


```
m_speed_ef <- lm(formula = Speed_m ~ Task*Stim*EF_front*EF_par, data=data_subset)
summary(m_speed_ef)
```


```
Call:
lm(formula = Speed_m ~ Task * Stim * EF_front * EF_par, data = data_subset)

Residuals:
    Min      1Q  Median      3Q     Max 
-34.922  -6.812  -0.027   5.879  32.036 

Coefficients:
                                                 Estimate Std. Error t value Pr(>|t|)  
(Intercept)                                         1.706     14.798   0.115   0.9086  
Task3back                                           6.930     20.928   0.331   0.7416  
Stimreal_out-of-phase                             -29.166     20.928  -1.394   0.1682  
EF_front                                         -228.370    238.321  -0.958   0.3415  
EF_par                                            -73.355    187.825  -0.391   0.6974  
Task3back:Stimreal_out-of-phase                    20.070     29.597   0.678   0.5002  
Task3back:EF_front                                -95.988    337.037  -0.285   0.7767  
Stimreal_out-of-phase:EF_front                    639.442    337.037   1.897   0.0623 .
Task3back:EF_par                                 -173.439    265.625  -0.653   0.5161  
Stimreal_out-of-phase:EF_par                      396.762    265.625   1.494   0.1402  
EF_front:EF_par                                  3114.404   2959.982   1.052   0.2967  
Task3back:Stimreal_out-of-phase:EF_front         -357.876    476.642  -0.751   0.4555  
Task3back:Stimreal_out-of-phase:EF_par           -205.260    375.650  -0.546   0.5867  
Task3back:EF_front:EF_par                        2030.254   4186.047   0.485   0.6293  
Stimreal_out-of-phase:EF_front:EF_par           -9031.975   4186.047  -2.158   0.0347 *
Task3back:Stimreal_out-of-phase:EF_front:EF_par  4751.644   5919.964   0.803   0.4251  
---
Signif. codes:  0 ‘***’ 0.001 ‘**’ 0.01 ‘*’ 0.05 ‘.’ 0.1 ‘ ’ 1

Residual standard error: 12.03 on 64 degrees of freedom
Multiple R-squared:  0.1764,    Adjusted R-squared:  -0.01667 
F-statistic: 0.9136 on 15 and 64 DF,  p-value: 0.5535
```


```
anova(m_speed_ef)
```


```
Analysis of Variance Table

Response: Speed_m
                          Df Sum Sq Mean Sq F value  Pr(>F)  
Task                       1    2.3    2.26  0.0156 0.90095  
Stim                       1   20.2   20.17  0.1393 0.71025  
EF_front                   1   17.1   17.10  0.1181 0.73221  
EF_par                     1  178.8  178.76  1.2345 0.27070  
Task:Stim                  1   85.0   84.98  0.5869 0.44645  
Task:EF_front              1   16.7   16.72  0.1155 0.73511  
Stim:EF_front              1   17.2   17.21  0.1188 0.73145  
Task:EF_par                1    5.8    5.82  0.0402 0.84174  
Stim:EF_par                1  395.6  395.62  2.7320 0.10325  
EF_front:EF_par            1   42.5   42.46  0.2933 0.59002  
Task:Stim:EF_front         1    0.0    0.00  0.0000 0.99540  
Task:Stim:EF_par           1   57.0   57.01  0.3937 0.53259  
Task:EF_front:EF_par       1  320.9  320.86  2.2158 0.14152  
Stim:EF_front:EF_par       1  732.2  732.25  5.0567 0.02798 *
Task:Stim:EF_front:EF_par  1   93.3   93.29  0.6442 0.42515  
Residuals                 64 9267.6  144.81                  
---
Signif. codes:  0 ‘***’ 0.001 ‘**’ 0.01 ‘*’ 0.05 ‘.’ 0.1 ‘ ’ 1
```


```
eta_squared(m_speed_ef)
```


```
# Effect Size for ANOVA (Type I)

Parameter                 | Eta2 (partial) |       95% CI
---------------------------------------------------------
Task                      |       2.44e-04 | [0.00, 1.00]
Stim                      |       2.17e-03 | [0.00, 1.00]
EF_front                  |       1.84e-03 | [0.00, 1.00]
EF_par                    |           0.02 | [0.00, 1.00]
Task:Stim                 |       9.09e-03 | [0.00, 1.00]
Task:EF_front             |       1.80e-03 | [0.00, 1.00]
Stim:EF_front             |       1.85e-03 | [0.00, 1.00]
Task:EF_par               |       6.28e-04 | [0.00, 1.00]
Stim:EF_par               |           0.04 | [0.00, 1.00]
EF_front:EF_par           |       4.56e-03 | [0.00, 1.00]
Task:Stim:EF_front        |       5.24e-07 | [0.00, 1.00]
Task:Stim:EF_par          |       6.11e-03 | [0.00, 1.00]
Task:EF_front:EF_par      |           0.03 | [0.00, 1.00]
Stim:EF_front:EF_par      |           0.07 | [0.00, 1.00]
Task:Stim:EF_front:EF_par |       9.97e-03 | [0.00, 1.00]

- One-sided CIs: upper bound fixed at [1.00].
```


#### Explore the triple interactions


```
n_rows = 100
choose_model <- m_speed_ef
stim_list <- levels(data_subset$Stim)
i_start = 0
i_stop = 0.3

columns = c("Stim","Task","EF_front", "EF_par") 
df_test = data.frame(matrix(nrow = n_rows, ncol = length(columns))) 
colnames(df_test) = columns

columns = c("Stim","Task","EF_front", "EF_par") 
df_temp = data.frame(matrix(nrow = n_rows, ncol = length(columns))) 
colnames(df_temp) = columns

for (s in stim_list){
  for (t in c("2back", "3back")){
    if (s == stim_list[1] & t == '2back'){
    df_test$Stim <- rep(s, n_rows)
    df_test$EF_front <- rep(seq(i_start, i_stop, length.out = 10), 10)
    df_test$EF_par <- rep(seq(i_start, i_stop, length.out = 10), each=10)
    #df_test$Task <- sample(c('2back', '3back'), 100, replace=TRUE)
    df_test$Task <- rep(t, 100)
    }
    else{
    df_temp$Stim <- rep(s, n_rows)
    df_temp$EF_front <- rep(seq(i_start, i_stop, length.out = 10), 10)
    df_temp$EF_par <- rep(seq(i_start, i_stop, length.out = 10), each=10)
    #df_temp$Task <- sample(c('2back', '3back'), 100, replace=TRUE)
    df_temp$Task <- rep(t, 100)
    df_test <- rbind(df_test, df_temp)
    }
  }
  
  
  #df_slice <- subset(df_test, Stim == s)
  #val_list <- append(val_list, seq(from=0, to=0.3, by=0.3/(nrow(df_slice)-1)))
}
```


```
# Make predictions
preds <- predict(choose_model, df_test)
df_test$Pred <- preds
```


```
# Viewing angles for the plots
angle_columns = c("Stim","Azim","Colatitude") 
df_angles = data.frame(matrix(nrow = 4, ncol = length(angle_columns))) 
colnames(df_angles) = angle_columns

df_angles$Stim <- stim_list
df_angles$Azim <- c(0, 0, 0, 0) # Theta, default 0
df_angles$Colatitude <- c(15, 15, 15, 15) # Phi, default 15
```


```
# Make plots
for (s in stim_list){
  for (t in c("2back", "3back")){
    disp_df <- subset(df_test, Stim == s & Task == t)
    persp(x=seq(i_start, i_stop, length.out = 10), y=seq(i_start, i_stop, length.out = 10), z=matrix(disp_df$Pred, nrow=10, ncol=10,byrow=TRUE), 
          theta = subset(df_angles, Stim == s)$Azim, phi = subset(df_angles, Stim == s)$Colatitude,
          xlab="EF_front",ylab="EF_par",zlab="Pred_Speed_m", main=paste(s,t), zlim=c(-200,300)) 
  }
}
```


```
Warning: surface extends beyond the box
```


```
# Export the data for plotting
save_file_name <- file.path(paste(data_directory, "MUNI_Efield_interactions.csv", sep='/'))
write.csv(df_test, save_file_name)
```

### Symmetry

To further explore the interaction between the induced electric
fields and the changes in behavior, we will look into the relative
magnitude among the electric fields induced at both sites (i.e., the
symmetry). For this exploration, we will only look at the bifocal
stimulation conditions.


```
data_subset <- subset(df_ef_muni, Stim == 'real_in-phase' | Stim == 'real_out-of-phase')
data_subset <- droplevels(data_subset)
levels(data_subset$Stim)
```


```
[1] "real_in-phase"     "real_out-of-phase"
```


```
m0 <- lm(formula = Speed_m ~ Task*Stim*EF_sym, data=data_subset)
summary(m0)
```


```
Call:
lm(formula = Speed_m ~ Task * Stim * EF_sym, data = data_subset)

Residuals:
    Min      1Q  Median      3Q     Max 
-34.822  -5.818  -0.750   5.048  37.700 

Coefficients:
                                       Estimate Std. Error t value Pr(>|t|)  
(Intercept)                              3.1588     5.0841   0.621   0.5364  
Task3back                               -1.2502     7.1900  -0.174   0.8624  
Stimreal_out-of-phase                  -12.7374     7.1900  -1.772   0.0807 .
EF_sym                                  -5.4550     3.1236  -1.746   0.0850 .
Task3back:Stimreal_out-of-phase          7.5164    10.1683   0.739   0.4622  
Task3back:EF_sym                        -0.3418     4.4175  -0.077   0.9385  
Stimreal_out-of-phase:EF_sym             8.4080     4.4175   1.903   0.0610 .
Task3back:Stimreal_out-of-phase:EF_sym  -2.4430     6.2472  -0.391   0.6969  
---
Signif. codes:  0 ‘***’ 0.001 ‘**’ 0.01 ‘*’ 0.05 ‘.’ 0.1 ‘ ’ 1

Residual standard error: 11.85 on 72 degrees of freedom
Multiple R-squared:  0.1017,    Adjusted R-squared:  0.01442 
F-statistic: 1.165 on 7 and 72 DF,  p-value: 0.3336
```


```
anova(m0)
```


```
Analysis of Variance Table

Response: Speed_m
                 Df  Sum Sq Mean Sq F value  Pr(>F)  
Task              1     2.3    2.26  0.0161 0.89937  
Stim              1    20.2   20.17  0.1437 0.70579  
EF_sym            1   237.8  237.79  1.6939 0.19723  
Task:Stim         1    85.0   84.98  0.6054 0.43908  
Task:EF_sym       1    35.2   35.16  0.2505 0.61826  
Stim:EF_sym       1   743.1  743.05  5.2932 0.02431 *
Task:Stim:EF_sym  1    21.5   21.47  0.1529 0.69692  
Residuals        72 10107.3  140.38                  
---
Signif. codes:  0 ‘***’ 0.001 ‘**’ 0.01 ‘*’ 0.05 ‘.’ 0.1 ‘ ’ 1
```


```
m1 <- lmer(formula = Speed_m ~ Task*Stim*EF_sym + (1 | ID), data=data_subset)
summary(m1)
```


```
Linear mixed model fit by REML. t-tests use Satterthwaite's method ['lmerModLmerTest']
Formula: Speed_m ~ Task * Stim * EF_sym + (1 | ID)
   Data: data_subset

REML criterion at convergence: 582.5

Scaled residuals: 
     Min       1Q   Median       3Q      Max 
-2.70206 -0.51452 -0.08627  0.38864  3.07333 

Random effects:
 Groups   Name        Variance Std.Dev.
 ID       (Intercept)   9.706   3.116  
 Residual             130.672  11.431  
Number of obs: 80, groups:  ID, 20

Fixed effects:
                                       Estimate Std. Error       df t value Pr(>|t|)  
(Intercept)                              3.1588     5.0841  70.9819   0.621   0.5364  
Task3back                               -1.2502     6.9370  54.0000  -0.180   0.8576  
Stimreal_out-of-phase                  -12.7374     6.9370  54.0000  -1.836   0.0718 .
EF_sym                                  -5.4550     3.1236  70.9819  -1.746   0.0851 .
Task3back:Stimreal_out-of-phase          7.5164     9.8104  54.0000   0.766   0.4469  
Task3back:EF_sym                        -0.3418     4.2620  54.0000  -0.080   0.9364  
Stimreal_out-of-phase:EF_sym             8.4080     4.2620  54.0000   1.973   0.0537 .
Task3back:Stimreal_out-of-phase:EF_sym  -2.4430     6.0274  54.0000  -0.405   0.6869  
---
Signif. codes:  0 ‘***’ 0.001 ‘**’ 0.01 ‘*’ 0.05 ‘.’ 0.1 ‘ ’ 1

Correlation of Fixed Effects:
            (Intr) Tsk3bc Stm_-- EF_sym Ts3:S_-- T3:EF_ S_--:E
Task3back   -0.682                                            
Stmrl_t-f-p -0.682  0.500                                     
EF_sym      -0.853  0.582  0.582                              
Tsk3bc:S_--  0.482 -0.707 -0.707 -0.412                       
Tsk3bck:EF_  0.582 -0.853 -0.427 -0.682  0.604                
Stmr_--:EF_  0.582 -0.427 -0.853 -0.682  0.604    0.500       
T3:S_--:EF_ -0.412  0.604  0.604  0.482 -0.853   -0.707 -0.707
```


```
anova(m1)
```


```
Type III Analysis of Variance Table with Satterthwaite's method
                 Sum Sq Mean Sq NumDF DenDF F value  Pr(>F)  
Task              34.16   34.16     1    54  0.2614 0.61124  
Stim             437.87  437.87     1    54  3.3509 0.07269 .
EF_sym           183.32  183.32     1    18  1.4029 0.25164  
Task:Stim         76.71   76.71     1    54  0.5870 0.44691  
Task:EF_sym       35.16   35.16     1    54  0.2691 0.60606  
Stim:EF_sym      743.05  743.05     1    54  5.6864 0.02064 *
Task:Stim:EF_sym  21.47   21.47     1    54  0.1643 0.68685  
---
Signif. codes:  0 ‘***’ 0.001 ‘**’ 0.01 ‘*’ 0.05 ‘.’ 0.1 ‘ ’ 1
```


```
AIC(m0, m1)
```


```
BIC(m0, m1)
```


```
m_speed_ef_sym <- lmer(formula = Speed_m ~ Task*Stim*EF_sym + (1 | ID), data=data_subset)
summary(m_speed_ef_sym)
```


```
Linear mixed model fit by REML. t-tests use Satterthwaite's method ['lmerModLmerTest']
Formula: Speed_m ~ Task * Stim * EF_sym + (1 | ID)
   Data: data_subset

REML criterion at convergence: 582.5

Scaled residuals: 
     Min       1Q   Median       3Q      Max 
-2.70206 -0.51452 -0.08627  0.38864  3.07333 

Random effects:
 Groups   Name        Variance Std.Dev.
 ID       (Intercept)   9.706   3.116  
 Residual             130.672  11.431  
Number of obs: 80, groups:  ID, 20

Fixed effects:
                                       Estimate Std. Error       df t value Pr(>|t|)  
(Intercept)                              3.1588     5.0841  70.9819   0.621   0.5364  
Task3back                               -1.2502     6.9370  54.0000  -0.180   0.8576  
Stimreal_out-of-phase                  -12.7374     6.9370  54.0000  -1.836   0.0718 .
EF_sym                                  -5.4550     3.1236  70.9819  -1.746   0.0851 .
Task3back:Stimreal_out-of-phase          7.5164     9.8104  54.0000   0.766   0.4469  
Task3back:EF_sym                        -0.3418     4.2620  54.0000  -0.080   0.9364  
Stimreal_out-of-phase:EF_sym             8.4080     4.2620  54.0000   1.973   0.0537 .
Task3back:Stimreal_out-of-phase:EF_sym  -2.4430     6.0274  54.0000  -0.405   0.6869  
---
Signif. codes:  0 ‘***’ 0.001 ‘**’ 0.01 ‘*’ 0.05 ‘.’ 0.1 ‘ ’ 1

Correlation of Fixed Effects:
            (Intr) Tsk3bc Stm_-- EF_sym Ts3:S_-- T3:EF_ S_--:E
Task3back   -0.682                                            
Stmrl_t-f-p -0.682  0.500                                     
EF_sym      -0.853  0.582  0.582                              
Tsk3bc:S_--  0.482 -0.707 -0.707 -0.412                       
Tsk3bck:EF_  0.582 -0.853 -0.427 -0.682  0.604                
Stmr_--:EF_  0.582 -0.427 -0.853 -0.682  0.604    0.500       
T3:S_--:EF_ -0.412  0.604  0.604  0.482 -0.853   -0.707 -0.707
```


```
anova(m_speed_ef_sym)
```


```
Type III Analysis of Variance Table with Satterthwaite's method
                 Sum Sq Mean Sq NumDF DenDF F value  Pr(>F)  
Task              34.16   34.16     1    54  0.2614 0.61124  
Stim             437.87  437.87     1    54  3.3509 0.07269 .
EF_sym           183.32  183.32     1    18  1.4029 0.25164  
Task:Stim         76.71   76.71     1    54  0.5870 0.44691  
Task:EF_sym       35.16   35.16     1    54  0.2691 0.60606  
Stim:EF_sym      743.05  743.05     1    54  5.6864 0.02064 *
Task:Stim:EF_sym  21.47   21.47     1    54  0.1643 0.68685  
---
Signif. codes:  0 ‘***’ 0.001 ‘**’ 0.01 ‘*’ 0.05 ‘.’ 0.1 ‘ ’ 1
```


```
eta_squared(m_speed_ef_sym)
```


```
# Effect Size for ANOVA (Type III)

Parameter        | Eta2 (partial) |       95% CI
------------------------------------------------
Task             |       4.82e-03 | [0.00, 1.00]
Stim             |           0.06 | [0.00, 1.00]
EF_sym           |           0.07 | [0.00, 1.00]
Task:Stim        |           0.01 | [0.00, 1.00]
Task:EF_sym      |       4.96e-03 | [0.00, 1.00]
Stim:EF_sym      |           0.10 | [0.01, 1.00]
Task:Stim:EF_sym |       3.03e-03 | [0.00, 1.00]

- One-sided CIs: upper bound fixed at [1.00].
```


#### Compare the slopes


```
slope_comp <- emtrends(m_speed_ef_sym, pairwise ~ Stim, var = "EF_sym")$contrasts
```


```
NOTE: Results may be misleading due to involvement in interactions
```


```
summary(slope_comp)
```


```
 contrast                              estimate   SE df t.ratio p.value
 (real_in-phase) - (real_out-of-phase)    -7.19 3.01 54  -2.385  0.0206

Results are averaged over the levels of: Task 
Degrees-of-freedom method: kenward-roger
```

### Combined E-field magnitude (all stimulation conditions)


```
data_subset <- subset(df_ef_muni, Stim != 'Placebo_loc')
data_subset <- droplevels(data_subset)
levels(data_subset$Stim)
```


```
[1] "real_frontal"      "real_parietal"     "real_in-phase"     "real_out-of-phase"
```


```
m0 <- lm(formula = Speed_m ~ Task*Stim*EF_mag, data=data_subset)
summary(m0)
```


```
Call:
lm(formula = Speed_m ~ Task * Stim * EF_mag, data = data_subset)

Residuals:
    Min      1Q  Median      3Q     Max 
-42.492  -8.293  -0.027   8.198  45.008 

Coefficients:
                                       Estimate Std. Error t value Pr(>|t|)
(Intercept)                               3.232      8.091   0.399    0.690
Task3back                               -17.464     11.442  -1.526    0.129
Stimreal_parietal                        -1.450      9.613  -0.151    0.880
Stimreal_in-phase                       -17.163     10.987  -1.562    0.120
Stimreal_out-of-phase                    -7.029     10.987  -0.640    0.523
EF_mag                                  -83.058    117.377  -0.708    0.480
Task3back:Stimreal_parietal               4.423     13.594   0.325    0.745
Task3back:Stimreal_in-phase              19.771     15.538   1.272    0.205
Task3back:Stimreal_out-of-phase          18.026     15.538   1.160    0.248
Task3back:EF_mag                        193.546    165.996   1.166    0.246
Stimreal_parietal:EF_mag                -76.883    135.201  -0.569    0.570
Stimreal_in-phase:EF_mag                182.724    137.566   1.328    0.186
Stimreal_out-of-phase:EF_mag             65.458    137.566   0.476    0.635
Task3back:Stimreal_parietal:EF_mag       61.428    191.203   0.321    0.748
Task3back:Stimreal_in-phase:EF_mag     -235.801    194.547  -1.212    0.227
Task3back:Stimreal_out-of-phase:EF_mag -174.316    194.547  -0.896    0.372

Residual standard error: 12.95 on 144 degrees of freedom
Multiple R-squared:  0.09601,   Adjusted R-squared:  0.001848 
F-statistic:  1.02 on 15 and 144 DF,  p-value: 0.4385
```


```
anova(m0)
```


```
Analysis of Variance Table

Response: Speed_m
                  Df  Sum Sq Mean Sq F value  Pr(>F)  
Task               1     2.5    2.52  0.0150 0.90258  
Stim               3   152.8   50.92  0.3037 0.82265  
EF_mag             1    27.6   27.56  0.1644 0.68575  
Task:Stim          3   446.2  148.74  0.8873 0.44936  
Task:EF_mag        1   537.8  537.76  3.2080 0.07538 .
Stim:EF_mag        3   461.4  153.80  0.9175 0.43416  
Task:Stim:EF_mag   3   935.6  311.87  1.8604 0.13895  
Residuals        144 24138.9  167.63                  
---
Signif. codes:  0 ‘***’ 0.001 ‘**’ 0.01 ‘*’ 0.05 ‘.’ 0.1 ‘ ’ 1
```


```
m1 <- lmer(formula = Speed_m ~ Task*Stim*EF_mag + (1 | ID), data=data_subset)
summary(m1)
```


```
Linear mixed model fit by REML. t-tests use Satterthwaite's method ['lmerModLmerTest']
Formula: Speed_m ~ Task * Stim * EF_mag + (1 | ID)
   Data: data_subset

REML criterion at convergence: 1130.7

Scaled residuals: 
    Min      1Q  Median      3Q     Max 
-2.5312 -0.5913 -0.0688  0.6256  3.8295 

Random effects:
 Groups   Name        Variance Std.Dev.
 ID       (Intercept)  29.71    5.45   
 Residual             137.41   11.72   
Number of obs: 160, groups:  ID, 20

Fixed effects:
                                        Estimate Std. Error        df t value Pr(>|t|)  
(Intercept)                               3.2553     7.7726  143.8162   0.419   0.6760  
Task3back                               -17.4640    10.3597  125.9067  -1.686   0.0943 .
Stimreal_parietal                        -0.9078     8.9924  135.4140  -0.101   0.9197  
Stimreal_in-phase                       -16.7223    10.1450  132.7636  -1.648   0.1016  
Stimreal_out-of-phase                    -6.5886    10.1450  132.7636  -0.649   0.5172  
EF_mag                                  -83.4189   112.0946  142.1330  -0.744   0.4580  
Task3back:Stimreal_parietal               4.4227    12.3082  125.9067   0.359   0.7199  
Task3back:Stimreal_in-phase              19.7714    14.0682  125.9067   1.405   0.1624  
Task3back:Stimreal_out-of-phase          18.0264    14.0682  125.9067   1.281   0.2024  
Task3back:EF_mag                        193.5458   150.2923  125.9067   1.288   0.2002  
Stimreal_parietal:EF_mag                -85.3257   127.3605  137.0139  -0.670   0.5040  
Stimreal_in-phase:EF_mag                178.2284   127.4992  133.0195   1.398   0.1645  
Stimreal_out-of-phase:EF_mag             60.9623   127.4992  133.0195   0.478   0.6333  
Task3back:Stimreal_parietal:EF_mag       61.4282   173.1144  125.9067   0.355   0.7233  
Task3back:Stimreal_in-phase:EF_mag     -235.8007   176.1423  125.9067  -1.339   0.1831  
Task3back:Stimreal_out-of-phase:EF_mag -174.3156   176.1423  125.9067  -0.990   0.3243  
---
Signif. codes:  0 ‘***’ 0.001 ‘**’ 0.01 ‘*’ 0.05 ‘.’ 0.1 ‘ ’ 1
```


```
Correlation matrix not shown by default, as p = 16 > 12.
Use print(x, correlation=TRUE)  or
    vcov(x)        if you need it
```


```
anova(m1)
```


```
Type III Analysis of Variance Table with Satterthwaite's method
                 Sum Sq Mean Sq NumDF   DenDF F value  Pr(>F)  
Task             315.47  315.47     1 125.907  2.2957 0.13224  
Stim             332.31  110.77     3 134.957  0.8061 0.49258  
EF_mag             7.02    7.02     1  64.079  0.0511 0.82188  
Task:Stim        466.02  155.34     3 125.907  1.1305 0.33942  
Task:EF_mag      531.08  531.08     1 125.907  3.8648 0.05151 .
Stim:EF_mag      480.40  160.13     3 132.403  1.1653 0.32552  
Task:Stim:EF_mag 935.61  311.87     3 125.907  2.2695 0.08367 .
---
Signif. codes:  0 ‘***’ 0.001 ‘**’ 0.01 ‘*’ 0.05 ‘.’ 0.1 ‘ ’ 1
```


```
AIC(m0, m1)
```


```
BIC(m0, m1)
```


```
m_speed_ef_mag <- lmer(formula = Speed_m ~ Task*Stim*EF_mag + (1 | ID), data=data_subset)
summary(m_speed_ef_mag)
```


```
Linear mixed model fit by REML. t-tests use Satterthwaite's method ['lmerModLmerTest']
Formula: Speed_m ~ Task * Stim * EF_mag + (1 | ID)
   Data: data_subset

REML criterion at convergence: 1130.7

Scaled residuals: 
    Min      1Q  Median      3Q     Max 
-2.5312 -0.5913 -0.0688  0.6256  3.8295 

Random effects:
 Groups   Name        Variance Std.Dev.
 ID       (Intercept)  29.71    5.45   
 Residual             137.41   11.72   
Number of obs: 160, groups:  ID, 20

Fixed effects:
                                        Estimate Std. Error        df t value Pr(>|t|)  
(Intercept)                               3.2553     7.7726  143.8162   0.419   0.6760  
Task3back                               -17.4640    10.3597  125.9067  -1.686   0.0943 .
Stimreal_parietal                        -0.9078     8.9924  135.4140  -0.101   0.9197  
Stimreal_in-phase                       -16.7223    10.1450  132.7636  -1.648   0.1016  
Stimreal_out-of-phase                    -6.5886    10.1450  132.7636  -0.649   0.5172  
EF_mag                                  -83.4189   112.0946  142.1330  -0.744   0.4580  
Task3back:Stimreal_parietal               4.4227    12.3082  125.9067   0.359   0.7199  
Task3back:Stimreal_in-phase              19.7714    14.0682  125.9067   1.405   0.1624  
Task3back:Stimreal_out-of-phase          18.0264    14.0682  125.9067   1.281   0.2024  
Task3back:EF_mag                        193.5458   150.2923  125.9067   1.288   0.2002  
Stimreal_parietal:EF_mag                -85.3257   127.3605  137.0139  -0.670   0.5040  
Stimreal_in-phase:EF_mag                178.2284   127.4992  133.0195   1.398   0.1645  
Stimreal_out-of-phase:EF_mag             60.9623   127.4992  133.0195   0.478   0.6333  
Task3back:Stimreal_parietal:EF_mag       61.4282   173.1144  125.9067   0.355   0.7233  
Task3back:Stimreal_in-phase:EF_mag     -235.8007   176.1423  125.9067  -1.339   0.1831  
Task3back:Stimreal_out-of-phase:EF_mag -174.3156   176.1423  125.9067  -0.990   0.3243  
---
Signif. codes:  0 ‘***’ 0.001 ‘**’ 0.01 ‘*’ 0.05 ‘.’ 0.1 ‘ ’ 1
```


```
Correlation matrix not shown by default, as p = 16 > 12.
Use print(x, correlation=TRUE)  or
    vcov(x)        if you need it
```


```
anova(m_speed_ef_mag)
```


```
Type III Analysis of Variance Table with Satterthwaite's method
                 Sum Sq Mean Sq NumDF   DenDF F value  Pr(>F)  
Task             315.47  315.47     1 125.907  2.2957 0.13224  
Stim             332.31  110.77     3 134.957  0.8061 0.49258  
EF_mag             7.02    7.02     1  64.079  0.0511 0.82188  
Task:Stim        466.02  155.34     3 125.907  1.1305 0.33942  
Task:EF_mag      531.08  531.08     1 125.907  3.8648 0.05151 .
Stim:EF_mag      480.40  160.13     3 132.403  1.1653 0.32552  
Task:Stim:EF_mag 935.61  311.87     3 125.907  2.2695 0.08367 .
---
Signif. codes:  0 ‘***’ 0.001 ‘**’ 0.01 ‘*’ 0.05 ‘.’ 0.1 ‘ ’ 1
```


```
eta_squared(m_speed_ef_mag)
```


```
# Effect Size for ANOVA (Type III)

Parameter        | Eta2 (partial) |       95% CI
------------------------------------------------
Task             |           0.02 | [0.00, 1.00]
Stim             |           0.02 | [0.00, 1.00]
EF_mag           |       7.97e-04 | [0.00, 1.00]
Task:Stim        |           0.03 | [0.00, 1.00]
Task:EF_mag      |           0.03 | [0.00, 1.00]
Stim:EF_mag      |           0.03 | [0.00, 1.00]
Task:Stim:EF_mag |           0.05 | [0.00, 1.00]

- One-sided CIs: upper bound fixed at [1.00].
```

### The relationship between the intensity of the applied electric field on the target and the normalized speed


```
data_subset <- subset(df_ef_muni, Stim != 'Placebo_loc')
data_subset <- droplevels(data_subset)
levels(data_subset$Stim)
```


```
[1] "real_frontal"      "real_parietal"     "real_in-phase"     "real_out-of-phase"
```


```
m0 <- lm(formula = Speed_n_m ~ Task*Stim*EF_front*EF_par, data=data_subset)
summary(m0)
```


```
Call:
lm(formula = Speed_n_m ~ Task * Stim * EF_front * EF_par, data = data_subset)

Residuals:
       Min         1Q     Median         3Q        Max 
-0.0287751 -0.0075936 -0.0003625  0.0066544  0.0288856 

Coefficients: (8 not defined because of singularities)
                                                 Estimate Std. Error t value Pr(>|t|)  
(Intercept)                                      0.003609   0.007429   0.486   0.6279  
Task3back                                       -0.015769   0.010506  -1.501   0.1357  
Stimreal_parietal                               -0.002830   0.008826  -0.321   0.7490  
Stimreal_in-phase                               -0.007318   0.016399  -0.446   0.6561  
Stimreal_out-of-phase                           -0.028816   0.016399  -1.757   0.0811 .
EF_front                                        -0.075108   0.107778  -0.697   0.4871  
EF_par                                           0.311003   0.185560   1.676   0.0960 .
Task3back:Stimreal_parietal                      0.006051   0.012483   0.485   0.6286  
Task3back:Stimreal_in-phase                      0.024172   0.023192   1.042   0.2992  
Task3back:Stimreal_out-of-phase                  0.043585   0.023192   1.879   0.0623 .
Task3back:EF_front                               0.177266   0.152421   1.163   0.2469  
Stimreal_parietal:EF_front                             NA         NA      NA       NA  
Stimreal_in-phase:EF_front                      -0.023880   0.258943  -0.092   0.9267  
Stimreal_out-of-phase:EF_front                   0.441233   0.258943   1.704   0.0907 .
Task3back:EF_par                                -0.399566   0.262422  -1.523   0.1302  
Stimreal_parietal:EF_par                        -0.417907   0.195521  -2.137   0.0344 *
Stimreal_in-phase:EF_par                        -0.327108   0.262422  -1.246   0.2147  
Stimreal_out-of-phase:EF_par                           NA         NA      NA       NA  
EF_front:EF_par                                 -5.505173   2.924287  -1.883   0.0619 .
Task3back:Stimreal_parietal:EF_front                   NA         NA      NA       NA  
Task3back:Stimreal_in-phase:EF_front            -0.300036   0.366201  -0.819   0.4140  
Task3back:Stimreal_out-of-phase:EF_front        -0.631247   0.366201  -1.724   0.0870 .
Task3back:Stimreal_parietal:EF_par               0.590350   0.276508   2.135   0.0346 *
Task3back:Stimreal_in-phase:EF_par               0.228211   0.371120   0.615   0.5396  
Task3back:Stimreal_out-of-phase:EF_par                 NA         NA      NA       NA  
Task3back:EF_front:EF_par                        6.849328   4.135566   1.656   0.1000 .
Stimreal_parietal:EF_front:EF_par                      NA         NA      NA       NA  
Stimreal_in-phase:EF_front:EF_par                7.348429   4.135566   1.777   0.0778 .
Stimreal_out-of-phase:EF_front:EF_par                  NA         NA      NA       NA  
Task3back:Stimreal_parietal:EF_front:EF_par            NA         NA      NA       NA  
Task3back:Stimreal_in-phase:EF_front:EF_par     -4.747932   5.848574  -0.812   0.4183  
Task3back:Stimreal_out-of-phase:EF_front:EF_par        NA         NA      NA       NA  
---
Signif. codes:  0 ‘***’ 0.001 ‘**’ 0.01 ‘*’ 0.05 ‘.’ 0.1 ‘ ’ 1

Residual standard error: 0.01189 on 136 degrees of freedom
Multiple R-squared:  0.1201,    Adjusted R-squared:  -0.02868 
F-statistic: 0.8072 on 23 and 136 DF,  p-value: 0.7177
```


```
anova(m0)
```


```
Analysis of Variance Table

Response: Speed_n_m
                           Df    Sum Sq    Mean Sq F value  Pr(>F)  
Task                        1 0.0000037 0.00000366  0.0259 0.87235  
Stim                        3 0.0000402 0.00001340  0.0948 0.96278  
EF_front                    1 0.0000322 0.00003224  0.2281 0.63368  
EF_par                      1 0.0000533 0.00005335  0.3774 0.54000  
Task:Stim                   3 0.0003205 0.00010684  0.7560 0.52073  
Task:EF_front               1 0.0001093 0.00010926  0.7731 0.38082  
Stim:EF_front               2 0.0000681 0.00003403  0.2408 0.78635  
Task:EF_par                 1 0.0001379 0.00013786  0.9754 0.32509  
Stim:EF_par                 2 0.0003427 0.00017134  1.2123 0.30072  
EF_front:EF_par             1 0.0000109 0.00001094  0.0774 0.78131  
Task:Stim:EF_front          2 0.0001063 0.00005316  0.3762 0.68720  
Task:Stim:EF_par            2 0.0005659 0.00028293  2.0019 0.13904  
Task:EF_front:EF_par        1 0.0003310 0.00033103  2.3422 0.12824  
Stim:EF_front:EF_par        1 0.0004090 0.00040898  2.8937 0.09121 .
Task:Stim:EF_front:EF_par   1 0.0000931 0.00009314  0.6590 0.41832  
Residuals                 136 0.0192216 0.00014134                  
---
Signif. codes:  0 ‘***’ 0.001 ‘**’ 0.01 ‘*’ 0.05 ‘.’ 0.1 ‘ ’ 1
```


```
m1 <- lmer(formula = Speed_n_m ~ Task*Stim*EF_front*EF_par + (1 | ID), data=data_subset)
```


```
fixed-effect model matrix is rank deficient so dropping 8 columns / coefficients
```


```
summary(m1)
```


```
Linear mixed model fit by REML. t-tests use Satterthwaite's method ['lmerModLmerTest']
Formula: Speed_n_m ~ Task * Stim * EF_front * EF_par + (1 | ID)
   Data: data_subset

REML criterion at convergence: -897.1

Scaled residuals: 
     Min       1Q   Median       3Q      Max 
-1.85463 -0.61019 -0.07638  0.62654  2.34516 

Random effects:
 Groups   Name        Variance  Std.Dev.
 ID       (Intercept) 2.796e-05 0.005288
 Residual             1.133e-04 0.010643
Number of obs: 160, groups:  ID, 20

Fixed effects:
                                              Estimate Std. Error         df t value Pr(>|t|)   
(Intercept)                                   0.003972   0.007215 134.476999   0.551  0.58282   
Task3back                                    -0.015769   0.009406 117.887953  -1.676  0.09631 . 
Stimreal_parietal                            -0.003004   0.008351 135.026501  -0.360  0.71964   
Stimreal_in-phase                            -0.013455   0.015220 129.193027  -0.884  0.37832   
Stimreal_out-of-phase                        -0.034953   0.015220 129.193027  -2.296  0.02326 * 
EF_front                                     -0.080759   0.104209 135.737238  -0.775  0.43971   
EF_par                                        0.398235   0.176497 134.070282   2.256  0.02567 * 
Task3back:Stimreal_parietal                   0.006051   0.011175 117.887953   0.541  0.58919   
Task3back:Stimreal_in-phase                   0.024172   0.020763 117.887953   1.164  0.24672   
Task3back:Stimreal_out-of-phase               0.043585   0.020763 117.887952   2.099  0.03794 * 
Task3back:EF_front                            0.177266   0.136459 117.887953   1.299  0.19646   
Stimreal_in-phase:EF_front                    0.083994   0.241063 129.697563   0.348  0.72808   
Stimreal_out-of-phase:EF_front                0.549107   0.241063 129.697563   2.278  0.02437 * 
Task3back:EF_par                             -0.399566   0.234940 117.887952  -1.701  0.09163 . 
Stimreal_parietal:EF_par                     -0.508096   0.183555 131.477137  -2.768  0.00645 **
Stimreal_in-phase:EF_par                     -0.327108   0.234940 117.887952  -1.392  0.16645   
EF_front:EF_par                              -7.007385   2.775870 133.373938  -2.524  0.01276 * 
Task3back:Stimreal_in-phase:EF_front         -0.300036   0.327851 117.887953  -0.915  0.36198   
Task3back:Stimreal_out-of-phase:EF_front     -0.631247   0.327851 117.887952  -1.925  0.05659 . 
Task3back:Stimreal_parietal:EF_par            0.590350   0.247551 117.887952   2.385  0.01869 * 
Task3back:Stimreal_in-phase:EF_par            0.228211   0.332256 117.887952   0.687  0.49352   
Task3back:EF_front:EF_par                     6.849328   3.702480 117.887951   1.850  0.06683 . 
Stimreal_in-phase:EF_front:EF_par             7.348429   3.702480 117.887952   1.985  0.04950 * 
Task3back:Stimreal_in-phase:EF_front:EF_par  -4.747932   5.236098 117.887951  -0.907  0.36638   
---
Signif. codes:  0 ‘***’ 0.001 ‘**’ 0.01 ‘*’ 0.05 ‘.’ 0.1 ‘ ’ 1
```


```
Correlation matrix not shown by default, as p = 24 > 12.
Use print(x, correlation=TRUE)  or
    vcov(x)        if you need it
```


```
fit warnings:
fixed-effect model matrix is rank deficient so dropping 8 columns / coefficients
```


```
anova(m1)
```


```
Missing cells for: Stimreal_parietal:EF_front, Stimreal_frontal:EF_par, Task2back:Stimreal_parietal:EF_front, Task3back:Stimreal_parietal:EF_front, Task2back:Stimreal_frontal:EF_par, Task3back:Stimreal_frontal:EF_par, Stimreal_frontal:EF_front:EF_par, Stimreal_parietal:EF_front:EF_par, Task2back:Stimreal_frontal:EF_front:EF_par, Task3back:Stimreal_frontal:EF_front:EF_par, Task2back:Stimreal_parietal:EF_front:EF_par, Task3back:Stimreal_parietal:EF_front:EF_par.  
Interpret type III hypotheses with care.
```


```
Type III Analysis of Variance Table with Satterthwaite's method
                              Sum Sq    Mean Sq NumDF   DenDF F value  Pr(>F)  
Task                      0.00001611 0.00001611     1 117.888  0.1422 0.70680  
Stim                      0.00018210 0.00006070     3 124.874  0.5358 0.65858  
EF_front                  0.00005144 0.00005144     1  97.651  0.4541 0.50199  
EF_par                    0.00007092 0.00007092     1 107.531  0.6261 0.43054  
Task:Stim                 0.00059875 0.00019958     3 117.888  1.7618 0.15826  
Task:EF_front             0.00009207 0.00009207     1 117.888  0.8128 0.36914  
Stim:EF_front             0.00026816 0.00013408     2 125.668  1.1836 0.30957  
Task:EF_par               0.00014054 0.00014054     1 117.888  1.2406 0.26762  
Stim:EF_par               0.00029806 0.00014903     2 127.644  1.3155 0.27194  
EF_front:EF_par           0.00005301 0.00005301     1 120.085  0.4679 0.49527  
Task:Stim:EF_front        0.00045947 0.00022973     2 117.888  2.0280 0.13617  
Task:Stim:EF_par          0.00081625 0.00040812     2 117.888  3.6027 0.03029 *
Task:EF_front:EF_par      0.00033103 0.00033103     1 117.888  2.9221 0.09000 .
Stim:EF_front:EF_par      0.00040898 0.00040898     1 117.888  3.6102 0.05987 .
Task:Stim:EF_front:EF_par 0.00009314 0.00009314     1 117.888  0.8222 0.36638  
---
Signif. codes:  0 ‘***’ 0.001 ‘**’ 0.01 ‘*’ 0.05 ‘.’ 0.1 ‘ ’ 1
```


```
m_speed_n_ef <- lm(formula = Speed_n_m ~ Task*Stim*EF_front*EF_par, data=data_subset)
summary(m_speed_n_ef)
```


```
Call:
lm(formula = Speed_n_m ~ Task * Stim * EF_front * EF_par, data = data_subset)

Residuals:
       Min         1Q     Median         3Q        Max 
-0.0287751 -0.0075936 -0.0003625  0.0066544  0.0288856 

Coefficients: (8 not defined because of singularities)
                                                 Estimate Std. Error t value Pr(>|t|)  
(Intercept)                                      0.003609   0.007429   0.486   0.6279  
Task3back                                       -0.015769   0.010506  -1.501   0.1357  
Stimreal_parietal                               -0.002830   0.008826  -0.321   0.7490  
Stimreal_in-phase                               -0.007318   0.016399  -0.446   0.6561  
Stimreal_out-of-phase                           -0.028816   0.016399  -1.757   0.0811 .
EF_front                                        -0.075108   0.107778  -0.697   0.4871  
EF_par                                           0.311003   0.185560   1.676   0.0960 .
Task3back:Stimreal_parietal                      0.006051   0.012483   0.485   0.6286  
Task3back:Stimreal_in-phase                      0.024172   0.023192   1.042   0.2992  
Task3back:Stimreal_out-of-phase                  0.043585   0.023192   1.879   0.0623 .
Task3back:EF_front                               0.177266   0.152421   1.163   0.2469  
Stimreal_parietal:EF_front                             NA         NA      NA       NA  
Stimreal_in-phase:EF_front                      -0.023880   0.258943  -0.092   0.9267  
Stimreal_out-of-phase:EF_front                   0.441233   0.258943   1.704   0.0907 .
Task3back:EF_par                                -0.399566   0.262422  -1.523   0.1302  
Stimreal_parietal:EF_par                        -0.417907   0.195521  -2.137   0.0344 *
Stimreal_in-phase:EF_par                        -0.327108   0.262422  -1.246   0.2147  
Stimreal_out-of-phase:EF_par                           NA         NA      NA       NA  
EF_front:EF_par                                 -5.505173   2.924287  -1.883   0.0619 .
Task3back:Stimreal_parietal:EF_front                   NA         NA      NA       NA  
Task3back:Stimreal_in-phase:EF_front            -0.300036   0.366201  -0.819   0.4140  
Task3back:Stimreal_out-of-phase:EF_front        -0.631247   0.366201  -1.724   0.0870 .
Task3back:Stimreal_parietal:EF_par               0.590350   0.276508   2.135   0.0346 *
Task3back:Stimreal_in-phase:EF_par               0.228211   0.371120   0.615   0.5396  
Task3back:Stimreal_out-of-phase:EF_par                 NA         NA      NA       NA  
Task3back:EF_front:EF_par                        6.849328   4.135566   1.656   0.1000 .
Stimreal_parietal:EF_front:EF_par                      NA         NA      NA       NA  
Stimreal_in-phase:EF_front:EF_par                7.348429   4.135566   1.777   0.0778 .
Stimreal_out-of-phase:EF_front:EF_par                  NA         NA      NA       NA  
Task3back:Stimreal_parietal:EF_front:EF_par            NA         NA      NA       NA  
Task3back:Stimreal_in-phase:EF_front:EF_par     -4.747932   5.848574  -0.812   0.4183  
Task3back:Stimreal_out-of-phase:EF_front:EF_par        NA         NA      NA       NA  
---
Signif. codes:  0 ‘***’ 0.001 ‘**’ 0.01 ‘*’ 0.05 ‘.’ 0.1 ‘ ’ 1

Residual standard error: 0.01189 on 136 degrees of freedom
Multiple R-squared:  0.1201,    Adjusted R-squared:  -0.02868 
F-statistic: 0.8072 on 23 and 136 DF,  p-value: 0.7177
```


```
anova(m_speed_n_ef)
```


```
Analysis of Variance Table

Response: Speed_n_m
                           Df    Sum Sq    Mean Sq F value  Pr(>F)  
Task                        1 0.0000037 0.00000366  0.0259 0.87235  
Stim                        3 0.0000402 0.00001340  0.0948 0.96278  
EF_front                    1 0.0000322 0.00003224  0.2281 0.63368  
EF_par                      1 0.0000533 0.00005335  0.3774 0.54000  
Task:Stim                   3 0.0003205 0.00010684  0.7560 0.52073  
Task:EF_front               1 0.0001093 0.00010926  0.7731 0.38082  
Stim:EF_front               2 0.0000681 0.00003403  0.2408 0.78635  
Task:EF_par                 1 0.0001379 0.00013786  0.9754 0.32509  
Stim:EF_par                 2 0.0003427 0.00017134  1.2123 0.30072  
EF_front:EF_par             1 0.0000109 0.00001094  0.0774 0.78131  
Task:Stim:EF_front          2 0.0001063 0.00005316  0.3762 0.68720  
Task:Stim:EF_par            2 0.0005659 0.00028293  2.0019 0.13904  
Task:EF_front:EF_par        1 0.0003310 0.00033103  2.3422 0.12824  
Stim:EF_front:EF_par        1 0.0004090 0.00040898  2.8937 0.09121 .
Task:Stim:EF_front:EF_par   1 0.0000931 0.00009314  0.6590 0.41832  
Residuals                 136 0.0192216 0.00014134                  
---
Signif. codes:  0 ‘***’ 0.001 ‘**’ 0.01 ‘*’ 0.05 ‘.’ 0.1 ‘ ’ 1
```

## Relationship between behavior and the phase-amplitude coupling index (PAC)

As part of our analysis, we extracted the PAC at both stimulation
sites for each subject, as was done by Reinhart and Nguyen (2019). In
this paper, the authors looked at the PAC at the “memory ROI” (left
centro-temporal cluster), and they found high-performing young adults to
have high PAC values in this region. In contrast, older adults had low
PAC values in that region, as well as low performance in the WM task.
However, when stimulation was applied, performance improved shortly
after starting the stimulation, and the PAC was also high in older
adults. So, the objective of this analysis is to see whether there are
meaningful differences in PAC correlating with performance.

The PAC features we are using are a comparison between the 2-back and
the 3-back tasks (subtraction), and they are the following: - Memory ROI
(Like in Reinhart and Nguyen) - Frontal stimulation ROI - Parietal
stimulation ROI

The comparison between the 2-back and the 3-back task was done
through a subtraction in the PAC values. The rationale is that this
difference isolates the neural activity related to retaining information
in working memory for longer, which is assumed to be more difficult in
the 3-back compared to the 2-back task and is supported by the
behavioral differences seen in both the speed and the accuracy when
comparing performance in both tasks. Therefore, the resulting delta in
PAC among both tasks would be related to an individual’s ability to
perform the (harder) 3-back task.


```
data_directory <- file.path(paste(curr_dir, '../../../Code/Notebooks/Results', sep='/'))

file_name <- file.path(paste(data_directory, "Behavioral_and_PAC_blocks_iCOG_Design_30_06_2023.txt", sep='/'))
df_pac <- read.delim(file_name, header = TRUE, na.strings = "NN")
head(df_pac, 5)
```


```
df_pac$ID <- as.factor(df_pac$ID)
df_pac$School <- as.factor(df_pac$School)
df_pac$Day <- as.factor(df_pac$Day)
df_pac$Task <- as.factor(df_pac$Task)
df_pac$Stim <- as.factor(df_pac$Stim)
df_pac$Acc_u <- as.numeric(df_pac$Acc_u)
df_pac$Acc_n_u <- as.numeric(df_pac$Acc_n_u)
df_pac$Speed_u <- as.numeric(df_pac$Speed_u)
df_pac$Speed_n_u <- as.numeric(df_pac$Speed_n_u)
df_pac$Acc_m <- as.numeric(df_pac$Acc_m)
df_pac$Acc_n_m <- as.numeric(df_pac$Acc_n_m)
df_pac$Speed_m <- as.numeric(df_pac$Speed_m)
df_pac$Speed_n_m <- as.numeric(df_pac$Speed_n_m)
df_pac$PAC_mem <- as.numeric(df_pac$PAC_mem)
df_pac$PAC_front <- as.numeric(df_pac$PAC_front)
df_pac$PAC_par <- as.numeric(df_pac$PAC_par)
```


```
df_pac_muni <- subset(df_pac, School == 'MUNI')
df_pac_muni <- droplevels(df_pac_muni)
df_pac_muni$Stim <- factor(df_pac_muni$Stim, levels(df_pac_muni$Stim)[c(1, 2, 5, 3, 4)])
levels(df_pac_muni$Stim)
```


```
[1] "Placebo_loc"       "real_frontal"      "real_parietal"     "real_in-phase"     "real_out-of-phase"
```


### Relationship between PAC and performance

Based on Reinhart’s and Nguyen’s findings, people with higher PAC
values at the “Memory ROI” would be expected to have a better
performance in the task. Therefore, we will first test the relationship
between the PAC and the performance under the placebo condition. As the
PAC values we are using consist on the difference of values between the
2back and 3back conditions, which is meant to reflect the coupling
related to higher working memory demands, we will use the behavioral
scores of the 3back task. #### Accuracy and memory ROI


```
data_subset <- subset(df_pac_muni, Stim == "Placebo_loc" & Task == "3back")
data_subset <- droplevels(data_subset)
levels(data_subset$Stim)
```


```
[1] "Placebo_loc"
```


```
levels(data_subset$Task)
```


```
[1] "3back"
```


#### Average accuracy


```
m_pac_mem_acc_u_placebo <- lm(formula = Acc_u ~ PAC_mem, data=data_subset)
summary(m_pac_mem_acc_u_placebo)
```


```
Call:
lm(formula = Acc_u ~ PAC_mem, data = data_subset)

Residuals:
     Min       1Q   Median       3Q      Max 
-0.17660 -0.08519  0.03668  0.05620  0.13956 

Coefficients:
            Estimate Std. Error t value Pr(>|t|)    
(Intercept)  0.76780    0.02166  35.449   <2e-16 ***
PAC_mem     10.33397   11.89578   0.869    0.397    
---
Signif. codes:  0 ‘***’ 0.001 ‘**’ 0.01 ‘*’ 0.05 ‘.’ 0.1 ‘ ’ 1

Residual standard error: 0.09396 on 17 degrees of freedom
  (1 observation deleted due to missingness)
Multiple R-squared:  0.0425,    Adjusted R-squared:  -0.01382 
F-statistic: 0.7547 on 1 and 17 DF,  p-value: 0.3971
```


```
anova(m_pac_mem_acc_u_placebo)
```


```
Analysis of Variance Table

Response: Acc_u
          Df   Sum Sq   Mean Sq F value Pr(>F)
PAC_mem    1 0.006662 0.0066623  0.7547 0.3971
Residuals 17 0.150080 0.0088282
```


```
eta_squared(m_pac_mem_acc_u_placebo)
```


```
For one-way between subjects designs, partial eta squared is equivalent to eta squared. Returning eta squared.
```


```
# Effect Size for ANOVA

Parameter | Eta2 |       95% CI
-------------------------------
PAC_mem   | 0.04 | [0.00, 1.00]

- One-sided CIs: upper bound fixed at [1.00].
```

#### Accuracy and PAC at 10/20 EEG electrode-based targets


```
m_pac_targets_acc_u_placebo <- lm(formula = Acc_u ~ PAC_front*PAC_par, data=data_subset)
summary(m_pac_targets_acc_u_placebo)
```


```
Call:
lm(formula = Acc_u ~ PAC_front * PAC_par, data = data_subset)

Residuals:
     Min       1Q   Median       3Q      Max 
-0.18003 -0.04571  0.01453  0.06110  0.11485 

Coefficients:
                   Estimate Std. Error t value Pr(>|t|)    
(Intercept)       7.926e-01  2.416e-02  32.807 2.22e-15 ***
PAC_front         2.229e+01  1.139e+01   1.957   0.0692 .  
PAC_par           6.389e+00  1.544e+01   0.414   0.6849    
PAC_front:PAC_par 2.407e+03  4.900e+03   0.491   0.6304    
---
Signif. codes:  0 ‘***’ 0.001 ‘**’ 0.01 ‘*’ 0.05 ‘.’ 0.1 ‘ ’ 1

Residual standard error: 0.09097 on 15 degrees of freedom
  (1 observation deleted due to missingness)
Multiple R-squared:  0.2081,    Adjusted R-squared:  0.04966 
F-statistic: 1.314 on 3 and 15 DF,  p-value: 0.3068
```


```
anova(m_pac_targets_acc_u_placebo)
```


```
Analysis of Variance Table

Response: Acc_u
                  Df   Sum Sq   Mean Sq F value  Pr(>F)  
PAC_front          1 0.030580 0.0305802  3.6953 0.07377 .
PAC_par            1 0.000034 0.0000343  0.0041 0.94952  
PAC_front:PAC_par  1 0.001996 0.0019964  0.2412 0.63042  
Residuals         15 0.124132 0.0082754                  
---
Signif. codes:  0 ‘***’ 0.001 ‘**’ 0.01 ‘*’ 0.05 ‘.’ 0.1 ‘ ’ 1
```


```
eta_squared(m_pac_targets_acc_u_placebo)
```


```
# Effect Size for ANOVA (Type I)

Parameter         | Eta2 (partial) |       95% CI
-------------------------------------------------
PAC_front         |           0.20 | [0.00, 1.00]
PAC_par           |       2.76e-04 | [0.00, 1.00]
PAC_front:PAC_par |           0.02 | [0.00, 1.00]

- One-sided CIs: upper bound fixed at [1.00].
```

#### Check for a relationship between PAC in the placebo condition and performance under stimulation


```
data_subset <- subset(df_pac_muni, Stim != "Placebo_loc" & Task == "3back")
data_subset <- droplevels(data_subset)
levels(data_subset$Stim)
```


```
[1] "real_frontal"      "real_parietal"     "real_in-phase"     "real_out-of-phase"
```


```
levels(data_subset$Task)
```


```
[1] "3back"
```


```
m_pac_mem_acc_u_stim <- lm(formula = Acc_u ~ Stim*PAC_mem, data=data_subset)
summary(m_pac_mem_acc_u_stim)
```


```
Call:
lm(formula = Acc_u ~ Stim * PAC_mem, data = data_subset)

Residuals:
      Min        1Q    Median        3Q       Max 
-0.153098 -0.069575  0.009971  0.064313  0.140007 

Coefficients:
                                Estimate Std. Error t value Pr(>|t|)    
(Intercept)                     0.773090   0.019290  40.077   <2e-16 ***
Stimreal_parietal              -0.006026   0.027281  -0.221    0.826    
Stimreal_in-phase               0.011946   0.027281   0.438    0.663    
Stimreal_out-of-phase          -0.005345   0.027281  -0.196    0.845    
PAC_mem                        14.711637  10.594655   1.389    0.169    
Stimreal_parietal:PAC_mem     -13.911985  14.983105  -0.929    0.356    
Stimreal_in-phase:PAC_mem     -10.249402  14.983105  -0.684    0.496    
Stimreal_out-of-phase:PAC_mem -15.460631  14.983105  -1.032    0.306    
---
Signif. codes:  0 ‘***’ 0.001 ‘**’ 0.01 ‘*’ 0.05 ‘.’ 0.1 ‘ ’ 1

Residual standard error: 0.08368 on 68 degrees of freedom
  (4 observations deleted due to missingness)
Multiple R-squared:  0.03873,   Adjusted R-squared:  -0.06022 
F-statistic: 0.3914 on 7 and 68 DF,  p-value: 0.9043
```


```
anova(m_pac_mem_acc_u_stim)
```


```
Analysis of Variance Table

Response: Acc_u
             Df  Sum Sq   Mean Sq F value Pr(>F)
Stim          3 0.00437 0.0014554  0.2078 0.8906
PAC_mem       1 0.00576 0.0057642  0.8231 0.3675
Stim:PAC_mem  3 0.00906 0.0030184  0.4310 0.7314
Residuals    68 0.47618 0.0070027
```


```
m_pac_targets_acc_u_stims <- lm(formula = Acc_u ~ Stim*PAC_front*PAC_par, data=data_subset)
summary(m_pac_targets_acc_u_stims)
```


```
Call:
lm(formula = Acc_u ~ Stim * PAC_front * PAC_par, data = data_subset)

Residuals:
      Min        1Q    Median        3Q       Max 
-0.155027 -0.045734  0.002691  0.061280  0.137782 

Coefficients:
                                          Estimate Std. Error t value Pr(>|t|)    
(Intercept)                              7.913e-01  2.269e-02  34.869   <2e-16 ***
Stimreal_parietal                       -2.436e-02  3.209e-02  -0.759    0.451    
Stimreal_in-phase                        7.341e-03  3.209e-02   0.229    0.820    
Stimreal_out-of-phase                   -1.838e-02  3.209e-02  -0.573    0.569    
PAC_front                                1.603e+01  1.070e+01   1.499    0.139    
PAC_par                                 -1.832e+00  1.450e+01  -0.126    0.900    
Stimreal_parietal:PAC_front             -1.278e+01  1.513e+01  -0.845    0.401    
Stimreal_in-phase:PAC_front             -2.215e+00  1.513e+01  -0.146    0.884    
Stimreal_out-of-phase:PAC_front         -9.281e+00  1.513e+01  -0.614    0.542    
Stimreal_parietal:PAC_par               -1.174e+01  2.051e+01  -0.573    0.569    
Stimreal_in-phase:PAC_par                1.566e+00  2.051e+01   0.076    0.939    
Stimreal_out-of-phase:PAC_par           -5.293e+00  2.051e+01  -0.258    0.797    
PAC_front:PAC_par                       -3.991e+02  4.603e+03  -0.087    0.931    
Stimreal_parietal:PAC_front:PAC_par     -5.364e+02  6.509e+03  -0.082    0.935    
Stimreal_in-phase:PAC_front:PAC_par      2.456e+03  6.509e+03   0.377    0.707    
Stimreal_out-of-phase:PAC_front:PAC_par -1.126e+03  6.509e+03  -0.173    0.863    
---
Signif. codes:  0 ‘***’ 0.001 ‘**’ 0.01 ‘*’ 0.05 ‘.’ 0.1 ‘ ’ 1

Residual standard error: 0.08545 on 60 degrees of freedom
  (4 observations deleted due to missingness)
Multiple R-squared:  0.1157,    Adjusted R-squared:  -0.1054 
F-statistic: 0.5234 on 15 and 60 DF,  p-value: 0.9176
```


```
anova(m_pac_targets_acc_u_stims)
```


```
Analysis of Variance Table

Response: Acc_u
                       Df  Sum Sq   Mean Sq F value  Pr(>F)  
Stim                    3 0.00437 0.0014554  0.1993 0.89643  
PAC_front               1 0.03008 0.0300806  4.1201 0.04682 *
PAC_par                 1 0.00886 0.0088612  1.2137 0.27500  
Stim:PAC_front          3 0.00657 0.0021896  0.2999 0.82533  
Stim:PAC_par            3 0.00482 0.0016072  0.2201 0.88205  
PAC_front:PAC_par       1 0.00006 0.0000556  0.0076 0.93078  
Stim:PAC_front:PAC_par  3 0.00256 0.0008537  0.1169 0.94984  
Residuals              60 0.43805 0.0073009                  
---
Signif. codes:  0 ‘***’ 0.001 ‘**’ 0.01 ‘*’ 0.05 ‘.’ 0.1 ‘ ’ 1
```


```
eta_squared(m_pac_targets_acc_u_stims)
```


```
# Effect Size for ANOVA (Type I)

Parameter              | Eta2 (partial) |       95% CI
------------------------------------------------------
Stim                   |       9.87e-03 | [0.00, 1.00]
PAC_front              |           0.06 | [0.00, 1.00]
PAC_par                |           0.02 | [0.00, 1.00]
Stim:PAC_front         |           0.01 | [0.00, 1.00]
Stim:PAC_par           |           0.01 | [0.00, 1.00]
PAC_front:PAC_par      |       1.27e-04 | [0.00, 1.00]
Stim:PAC_front:PAC_par |       5.81e-03 | [0.00, 1.00]

- One-sided CIs: upper bound fixed at [1.00].
```

#### Normalized accuracy


```
data_subset <- subset(df_pac_muni, Stim == "Placebo_loc" & Task == "3back")
data_subset <- droplevels(data_subset)
levels(data_subset$Stim)
```


```
[1] "Placebo_loc"
```


```
levels(data_subset$Task)
```


```
[1] "3back"
```


```
m_pac_mem_acc_n_u_placebo <- lm(formula = Acc_n_u ~ PAC_mem, data=data_subset)
summary(m_pac_mem_acc_n_u_placebo)
```


```
Call:
lm(formula = Acc_n_u ~ PAC_mem, data = data_subset)

Residuals:
     Min       1Q   Median       3Q      Max 
-0.24792 -0.10570 -0.05358  0.03248  0.43234 

Coefficients:
            Estimate Std. Error t value Pr(>|t|)    
(Intercept)  1.00491    0.04205  23.899  1.6e-14 ***
PAC_mem      4.33206   23.09423   0.188    0.853    
---
Signif. codes:  0 ‘***’ 0.001 ‘**’ 0.01 ‘*’ 0.05 ‘.’ 0.1 ‘ ’ 1

Residual standard error: 0.1824 on 17 degrees of freedom
  (1 observation deleted due to missingness)
Multiple R-squared:  0.002066,  Adjusted R-squared:  -0.05664 
F-statistic: 0.03519 on 1 and 17 DF,  p-value: 0.8534
```


```
anova(m_pac_mem_acc_n_u_placebo)
```


```
Analysis of Variance Table

Response: Acc_n_u
          Df  Sum Sq  Mean Sq F value Pr(>F)
PAC_mem    1 0.00117 0.001171  0.0352 0.8534
Residuals 17 0.56565 0.033273
```


```
m_pac_targets_acc_n_u_placebo <- lm(formula = Acc_n_u ~ PAC_front*PAC_par, data=data_subset)
summary(m_pac_targets_acc_n_u_placebo)
```


```
Call:
lm(formula = Acc_n_u ~ PAC_front * PAC_par, data = data_subset)

Residuals:
     Min       1Q   Median       3Q      Max 
-0.17807 -0.09381 -0.03567  0.02866  0.34735 

Coefficients:
                    Estimate Std. Error t value Pr(>|t|)    
(Intercept)        9.614e-01  4.205e-02  22.862 4.51e-13 ***
PAC_front         -3.358e+01  1.982e+01  -1.694    0.111    
PAC_par           -4.053e+01  2.687e+01  -1.508    0.152    
PAC_front:PAC_par -1.809e+03  8.529e+03  -0.212    0.835    
---
Signif. codes:  0 ‘***’ 0.001 ‘**’ 0.01 ‘*’ 0.05 ‘.’ 0.1 ‘ ’ 1

Residual standard error: 0.1583 on 15 degrees of freedom
  (1 observation deleted due to missingness)
Multiple R-squared:  0.3365,    Adjusted R-squared:  0.2038 
F-statistic: 2.536 on 3 and 15 DF,  p-value: 0.09591
```


```
anova(m_pac_targets_acc_n_u_placebo)
```


```
Analysis of Variance Table

Response: Acc_n_u
                  Df  Sum Sq  Mean Sq F value  Pr(>F)  
PAC_front          1 0.08300 0.082996  3.3104 0.08886 .
PAC_par            1 0.10662 0.106624  4.2529 0.05695 .
PAC_front:PAC_par  1 0.00113 0.001128  0.0450 0.83488  
Residuals         15 0.37607 0.025071                  
---
Signif. codes:  0 ‘***’ 0.001 ‘**’ 0.01 ‘*’ 0.05 ‘.’ 0.1 ‘ ’ 1
```

#### Stimulations


```
data_subset <- subset(df_pac_muni, Stim != "Placebo_loc" & Task == "3back")
data_subset <- droplevels(data_subset)
levels(data_subset$Stim)
```


```
[1] "real_frontal"      "real_parietal"     "real_in-phase"     "real_out-of-phase"
```


```
levels(data_subset$Task)
```


```
[1] "3back"
```


```
m_pac_mem_acc_n_u_stim <- lm(formula = Acc_n_u ~ Stim*PAC_mem, data=data_subset)
summary(m_pac_mem_acc_n_u_stim)
```


```
Call:
lm(formula = Acc_n_u ~ Stim * PAC_mem, data = data_subset)

Residuals:
     Min       1Q   Median       3Q      Max 
-0.32667 -0.09812 -0.01375  0.08739  0.35842 

Coefficients:
                               Estimate Std. Error t value Pr(>|t|)    
(Intercept)                     1.05566    0.03492  30.229   <2e-16 ***
Stimreal_parietal              -0.07985    0.04939  -1.617   0.1106    
Stimreal_in-phase              -0.08627    0.04939  -1.747   0.0852 .  
Stimreal_out-of-phase          -0.07129    0.04939  -1.443   0.1535    
PAC_mem                       -13.44757   19.18030  -0.701   0.4856    
Stimreal_parietal:PAC_mem      15.12586   27.12504   0.558   0.5789    
Stimreal_in-phase:PAC_mem      15.00580   27.12504   0.553   0.5819    
Stimreal_out-of-phase:PAC_mem  29.11333   27.12504   1.073   0.2869    
---
Signif. codes:  0 ‘***’ 0.001 ‘**’ 0.01 ‘*’ 0.05 ‘.’ 0.1 ‘ ’ 1

Residual standard error: 0.1515 on 68 degrees of freedom
  (4 observations deleted due to missingness)
Multiple R-squared:  0.06662,   Adjusted R-squared:  -0.02947 
F-statistic: 0.6933 on 7 and 68 DF,  p-value: 0.6774
```


```
anova(m_pac_mem_acc_n_u_stim)
```


```
Analysis of Variance Table

Response: Acc_n_u
             Df  Sum Sq   Mean Sq F value Pr(>F)
Stim          3 0.08447 0.0281553  1.2268 0.3067
PAC_mem       1 0.00046 0.0004641  0.0202 0.8873
Stim:PAC_mem  3 0.02646 0.0088185  0.3842 0.7647
Residuals    68 1.56066 0.0229509
```


```
m_pac_targets_acc_n_u_stim <- lm(formula = Acc_n_u ~ Stim*PAC_front*PAC_par, data=data_subset)
summary(m_pac_targets_acc_n_u_stim)
```


```
Call:
lm(formula = Acc_n_u ~ Stim * PAC_front * PAC_par, data = data_subset)

Residuals:
     Min       1Q   Median       3Q      Max 
-0.41755 -0.07458  0.01242  0.06327  0.26742 

Coefficients:
                                          Estimate Std. Error t value Pr(>|t|)    
(Intercept)                              1.060e+00  3.636e-02  29.153  < 2e-16 ***
Stimreal_parietal                       -4.125e-02  5.142e-02  -0.802  0.42559    
Stimreal_in-phase                       -5.676e-02  5.142e-02  -1.104  0.27404    
Stimreal_out-of-phase                   -9.010e-02  5.142e-02  -1.752  0.08484 .  
PAC_front                                1.841e+01  1.714e+01   1.074  0.28694    
PAC_par                                 -5.812e+01  2.323e+01  -2.502  0.01510 *  
Stimreal_parietal:PAC_front              2.086e+01  2.423e+01   0.861  0.39277    
Stimreal_in-phase:PAC_front              8.404e+00  2.423e+01   0.347  0.72997    
Stimreal_out-of-phase:PAC_front         -4.288e+01  2.423e+01  -1.770  0.08187 .  
Stimreal_parietal:PAC_par                8.269e+01  3.286e+01   2.517  0.01453 *  
Stimreal_in-phase:PAC_par                8.994e+01  3.286e+01   2.738  0.00813 ** 
Stimreal_out-of-phase:PAC_par            7.766e+01  3.286e+01   2.364  0.02136 *  
PAC_front:PAC_par                       -1.208e+04  7.375e+03  -1.637  0.10678    
Stimreal_parietal:PAC_front:PAC_par      2.039e+04  1.043e+04   1.956  0.05518 .  
Stimreal_in-phase:PAC_front:PAC_par      1.736e+04  1.043e+04   1.664  0.10124    
Stimreal_out-of-phase:PAC_front:PAC_par  8.369e+03  1.043e+04   0.802  0.42545    
---
Signif. codes:  0 ‘***’ 0.001 ‘**’ 0.01 ‘*’ 0.05 ‘.’ 0.1 ‘ ’ 1

Residual standard error: 0.1369 on 60 degrees of freedom
  (4 observations deleted due to missingness)
Multiple R-squared:  0.3275,    Adjusted R-squared:  0.1593 
F-statistic: 1.948 on 15 and 60 DF,  p-value: 0.03557
```


```
anova(m_pac_targets_acc_n_u_stim)
```


```
Analysis of Variance Table

Response: Acc_n_u
                       Df  Sum Sq  Mean Sq F value  Pr(>F)  
Stim                    3 0.08447 0.028155  1.5023 0.22312  
PAC_front               1 0.07456 0.074558  3.9782 0.05064 .
PAC_par                 1 0.01076 0.010757  0.5740 0.45165  
Stim:PAC_front          3 0.13282 0.044273  2.3623 0.08019 .
Stim:PAC_par            3 0.15649 0.052162  2.7832 0.04853 *
PAC_front:PAC_par       1 0.00041 0.000409  0.0218 0.88305  
Stim:PAC_front:PAC_par  3 0.08804 0.029348  1.5659 0.20699  
Residuals              60 1.12451 0.018742                  
---
Signif. codes:  0 ‘***’ 0.001 ‘**’ 0.01 ‘*’ 0.05 ‘.’ 0.1 ‘ ’ 1
```


```
eta_squared(m_pac_targets_acc_n_u_stim)
```


```
# Effect Size for ANOVA (Type I)

Parameter              | Eta2 (partial) |       95% CI
------------------------------------------------------
Stim                   |           0.07 | [0.00, 1.00]
PAC_front              |           0.06 | [0.00, 1.00]
PAC_par                |       9.48e-03 | [0.00, 1.00]
Stim:PAC_front         |           0.11 | [0.00, 1.00]
Stim:PAC_par           |           0.12 | [0.00, 1.00]
PAC_front:PAC_par      |       3.64e-04 | [0.00, 1.00]
Stim:PAC_front:PAC_par |           0.07 | [0.00, 1.00]

- One-sided CIs: upper bound fixed at [1.00].
```

#### Compare the slopes resulting from the interaction between the stimulation and the difference in PAC scores


```
slope_comp <- emtrends(m_pac_targets_acc_n_u_stim, pairwise ~ Stim, var = "PAC_par")$contrasts
```


```
NOTE: Results may be misleading due to involvement in interactions
```


```
summary(slope_comp)
```


```
 contrast                              estimate   SE df t.ratio p.value
 real_frontal - real_parietal            -62.39 25.9 60  -2.409  0.0863
 real_frontal - (real_in-phase)          -72.66 25.9 60  -2.805  0.0333
 real_frontal - (real_out-of-phase)      -69.32 25.9 60  -2.676  0.0460
 real_parietal - (real_in-phase)         -10.27 25.9 60  -0.396  0.9787
 real_parietal - (real_out-of-phase)      -6.93 25.9 60  -0.268  0.9932
 (real_in-phase) - (real_out-of-phase)     3.34 25.9 60   0.129  0.9992

P value adjustment: tukey method for comparing a family of 4 estimates
```

### PAC and speed

#### Placebo


```
data_subset <- subset(df_pac_muni, Stim == "Placebo_loc" & Task == "3back")
data_subset <- droplevels(data_subset)
levels(data_subset$Stim)
```


```
[1] "Placebo_loc"
```


```
levels(data_subset$Task)
```


```
[1] "3back"
```

#### Memory ROI


```
m_pac_mem_speed_u_placebo <- lm(formula = Speed_u ~ PAC_mem, data=data_subset)
summary(m_pac_mem_speed_u_placebo)
```


```
Call:
lm(formula = Speed_u ~ PAC_mem, data = data_subset)

Residuals:
   Min     1Q Median     3Q    Max 
-276.8 -137.3  -17.9  106.3  362.8 

Coefficients:
            Estimate Std. Error t value Pr(>|t|)    
(Intercept)   1076.2       38.4   28.03 1.14e-15 ***
PAC_mem      12227.8    21090.2    0.58     0.57    
---
Signif. codes:  0 ‘***’ 0.001 ‘**’ 0.01 ‘*’ 0.05 ‘.’ 0.1 ‘ ’ 1

Residual standard error: 166.6 on 17 degrees of freedom
  (1 observation deleted due to missingness)
Multiple R-squared:  0.01939,   Adjusted R-squared:  -0.03829 
F-statistic: 0.3362 on 1 and 17 DF,  p-value: 0.5697
```


```
anova(m_pac_mem_speed_u_placebo)
```


```
Analysis of Variance Table

Response: Speed_u
          Df Sum Sq Mean Sq F value Pr(>F)
PAC_mem    1   9328  9327.9  0.3362 0.5697
Residuals 17 471735 27749.1
```

#### Frontal and parietal targets


```
m_pac_targets_speed_u_placebo <- lm(formula = Speed_u ~ PAC_front*PAC_par, data=data_subset)
summary(m_pac_targets_speed_u_placebo)
```


```
Call:
lm(formula = Speed_u ~ PAC_front * PAC_par, data = data_subset)

Residuals:
    Min      1Q  Median      3Q     Max 
-246.65 -116.11   14.75   78.30  315.48 

Coefficients:
                   Estimate Std. Error t value Pr(>|t|)    
(Intercept)       1.115e+03  4.248e+01  26.238 5.99e-14 ***
PAC_front         3.225e+04  2.002e+04   1.611    0.128    
PAC_par           1.516e+04  2.715e+04   0.558    0.585    
PAC_front:PAC_par 2.420e+05  8.617e+06   0.028    0.978    
---
Signif. codes:  0 ‘***’ 0.001 ‘**’ 0.01 ‘*’ 0.05 ‘.’ 0.1 ‘ ’ 1

Residual standard error: 160 on 15 degrees of freedom
  (1 observation deleted due to missingness)
Multiple R-squared:  0.2022,    Adjusted R-squared:  0.04261 
F-statistic: 1.267 on 3 and 15 DF,  p-value: 0.3213
```


```
anova(m_pac_targets_speed_u_placebo)
```


```
Analysis of Variance Table

Response: Speed_u
                  Df Sum Sq Mean Sq F value  Pr(>F)  
PAC_front          1  79958   79958  3.1250 0.09743 .
PAC_par            1  17281   17281  0.6754 0.42405  
PAC_front:PAC_par  1     20      20  0.0008 0.97797  
Residuals         15 383803   25587                  
---
Signif. codes:  0 ‘***’ 0.001 ‘**’ 0.01 ‘*’ 0.05 ‘.’ 0.1 ‘ ’ 1
```


```
eta_squared(m_pac_targets_speed_u_placebo)
```


```
# Effect Size for ANOVA (Type I)

Parameter         | Eta2 (partial) |       95% CI
-------------------------------------------------
PAC_front         |           0.17 | [0.00, 1.00]
PAC_par           |           0.04 | [0.00, 1.00]
PAC_front:PAC_par |       5.26e-05 | [0.00, 1.00]

- One-sided CIs: upper bound fixed at [1.00].
```

#### Stimulation groups


```
data_subset <- subset(df_pac_muni, Stim != "Placebo_loc" & Task == "3back")
data_subset <- droplevels(data_subset)
levels(data_subset$Stim)
```


```
[1] "real_frontal"      "real_parietal"     "real_in-phase"     "real_out-of-phase"
```


```
levels(data_subset$Task)
```


```
[1] "3back"
```

#### Memory ROI


```
m_pac_mem_speed_u_stim <- lm(formula = Speed_u ~ Stim*PAC_mem, data=data_subset)
summary(m_pac_mem_speed_u_stim)
```


```
Call:
lm(formula = Speed_u ~ Stim * PAC_mem, data = data_subset)

Residuals:
    Min      1Q  Median      3Q     Max 
-353.06 -117.53   12.94   91.71  463.34 

Coefficients:
                               Estimate Std. Error t value Pr(>|t|)    
(Intercept)                    1043.957     42.889  24.341   <2e-16 ***
Stimreal_parietal                -5.361     60.654  -0.088    0.930    
Stimreal_in-phase                -7.904     60.654  -0.130    0.897    
Stimreal_out-of-phase            -9.797     60.654  -0.162    0.872    
PAC_mem                       12549.147  23555.605   0.533    0.596    
Stimreal_parietal:PAC_mem     30509.949  33312.656   0.916    0.363    
Stimreal_in-phase:PAC_mem     27538.902  33312.656   0.827    0.411    
Stimreal_out-of-phase:PAC_mem 12916.696  33312.656   0.388    0.699    
---
Signif. codes:  0 ‘***’ 0.001 ‘**’ 0.01 ‘*’ 0.05 ‘.’ 0.1 ‘ ’ 1

Residual standard error: 186.1 on 68 degrees of freedom
  (4 observations deleted due to missingness)
Multiple R-squared:  0.1019,    Adjusted R-squared:  0.009394 
F-statistic: 1.102 on 7 and 68 DF,  p-value: 0.3722
```


```
anova(m_pac_mem_speed_u_stim)
```


```
Analysis of Variance Table

Response: Speed_u
             Df  Sum Sq Mean Sq F value  Pr(>F)  
Stim          3     723     241  0.0070 0.99919  
PAC_mem       1  228962  228962  6.6143 0.01231 *
Stim:PAC_mem  3   37248   12416  0.3587 0.78301  
Residuals    68 2353890   34616                  
---
Signif. codes:  0 ‘***’ 0.001 ‘**’ 0.01 ‘*’ 0.05 ‘.’ 0.1 ‘ ’ 1
```

#### Frontal and parietal targets


```
m_pac_targets_speed_u_stim <- lm(formula = Speed_u ~ Stim*PAC_front*PAC_par, data=data_subset)
summary(m_pac_targets_speed_u_stim)
```


```
Call:
lm(formula = Speed_u ~ Stim * PAC_front * PAC_par, data = data_subset)

Residuals:
    Min      1Q  Median      3Q     Max 
-341.47 -105.70   22.89  105.69  463.60 

Coefficients:
                                          Estimate Std. Error t value Pr(>|t|)    
(Intercept)                              1.071e+03  4.987e+01  21.476   <2e-16 ***
Stimreal_parietal                        6.884e+00  7.053e+01   0.098    0.923    
Stimreal_in-phase                        1.607e+01  7.053e+01   0.228    0.821    
Stimreal_out-of-phase                   -3.647e+00  7.053e+01  -0.052    0.959    
PAC_front                                1.954e+04  2.350e+04   0.831    0.409    
PAC_par                                  2.295e+04  3.186e+04   0.720    0.474    
Stimreal_parietal:PAC_front              7.432e+03  3.324e+04   0.224    0.824    
Stimreal_in-phase:PAC_front              1.870e+04  3.324e+04   0.563    0.576    
Stimreal_out-of-phase:PAC_front          4.475e+03  3.324e+04   0.135    0.893    
Stimreal_parietal:PAC_par               -1.219e+04  4.506e+04  -0.270    0.788    
Stimreal_in-phase:PAC_par               -8.779e+03  4.506e+04  -0.195    0.846    
Stimreal_out-of-phase:PAC_par           -1.863e+04  4.506e+04  -0.413    0.681    
PAC_front:PAC_par                        2.942e+06  1.011e+07   0.291    0.772    
Stimreal_parietal:PAC_front:PAC_par     -6.957e+06  1.430e+07  -0.486    0.628    
Stimreal_in-phase:PAC_front:PAC_par     -7.126e+06  1.430e+07  -0.498    0.620    
Stimreal_out-of-phase:PAC_front:PAC_par -1.133e+07  1.430e+07  -0.792    0.431    
---
Signif. codes:  0 ‘***’ 0.001 ‘**’ 0.01 ‘*’ 0.05 ‘.’ 0.1 ‘ ’ 1

Residual standard error: 187.8 on 60 degrees of freedom
  (4 observations deleted due to missingness)
Multiple R-squared:  0.1929,    Adjusted R-squared:  -0.008894 
F-statistic: 0.9559 on 15 and 60 DF,  p-value: 0.5107
```


```
anova(m_pac_targets_speed_u_stim)
```


```
Analysis of Variance Table

Response: Speed_u
                       Df  Sum Sq Mean Sq F value   Pr(>F)   
Stim                    3     723     241  0.0068 0.999215   
PAC_front               1  291228  291228  8.2606 0.005597 **
PAC_par                 1  146034  146034  4.1422 0.046251 * 
Stim:PAC_front          3   24898    8299  0.2354 0.871342   
Stim:PAC_par            3    3813    1271  0.0361 0.990741   
PAC_front:PAC_par       1   16045   16045  0.4551 0.502515   
Stim:PAC_front:PAC_par  3   22778    7593  0.2154 0.885376   
Residuals              60 2115305   35255                    
---
Signif. codes:  0 ‘***’ 0.001 ‘**’ 0.01 ‘*’ 0.05 ‘.’ 0.1 ‘ ’ 1
```

#### Normalized speed


```
data_subset <- subset(df_pac_muni, Stim == "Placebo_loc" & Task == "3back")
data_subset <- droplevels(data_subset)
levels(data_subset$Stim)
```


```
[1] "Placebo_loc"
```


```
levels(data_subset$Task)
```


```
[1] "3back"
```


```
m_pac_mem_speed_n_u_placebo <- lm(formula = Speed_n_u ~ PAC_mem, data=data_subset)
summary(m_pac_mem_speed_n_u_placebo)
```


```
Call:
lm(formula = Speed_n_u ~ PAC_mem, data = data_subset)

Residuals:
     Min       1Q   Median       3Q      Max 
-0.22614 -0.12757 -0.03714  0.11539  0.37146 

Coefficients:
            Estimate Std. Error t value Pr(>|t|)    
(Intercept)  1.00651    0.04171  24.133 1.36e-14 ***
PAC_mem     23.15316   22.90614   1.011    0.326    
---
Signif. codes:  0 ‘***’ 0.001 ‘**’ 0.01 ‘*’ 0.05 ‘.’ 0.1 ‘ ’ 1

Residual standard error: 0.1809 on 17 degrees of freedom
  (1 observation deleted due to missingness)
Multiple R-squared:  0.05669,   Adjusted R-squared:  0.001203 
F-statistic: 1.022 on 1 and 17 DF,  p-value: 0.3263
```


```
anova(m_pac_mem_speed_n_u_placebo)
```


```
Analysis of Variance Table

Response: Speed_n_u
          Df  Sum Sq  Mean Sq F value Pr(>F)
PAC_mem    1 0.03344 0.033443  1.0217 0.3263
Residuals 17 0.55647 0.032734
```


```
m_pac_targets_speed_n_u_placebo <- lm(formula = Speed_n_u ~ PAC_front*PAC_par, data=data_subset)
summary(m_pac_targets_speed_n_u_placebo)
```


```
Call:
lm(formula = Speed_n_u ~ PAC_front * PAC_par, data = data_subset)

Residuals:
     Min       1Q   Median       3Q      Max 
-0.26665 -0.12956 -0.03092  0.08116  0.41323 

Coefficients:
                   Estimate Std. Error t value Pr(>|t|)    
(Intercept)          1.0245     0.0478  21.434 1.15e-12 ***
PAC_front            2.7355    22.5268   0.121    0.905    
PAC_par             49.8444    30.5412   1.632    0.123    
PAC_front:PAC_par 7367.1887  9694.6557   0.760    0.459    
---
Signif. codes:  0 ‘***’ 0.001 ‘**’ 0.01 ‘*’ 0.05 ‘.’ 0.1 ‘ ’ 1

Residual standard error: 0.18 on 15 degrees of freedom
  (1 observation deleted due to missingness)
Multiple R-squared:  0.1764,    Adjusted R-squared:  0.01169 
F-statistic: 1.071 on 3 and 15 DF,  p-value: 0.391
```


```
anova(m_pac_targets_speed_n_u_placebo)
```


```
Analysis of Variance Table

Response: Speed_n_u
                  Df  Sum Sq  Mean Sq F value Pr(>F)
PAC_front          1 0.00066 0.000657  0.0203 0.8886
PAC_par            1 0.08471 0.084705  2.6152 0.1267
PAC_front:PAC_par  1 0.01870 0.018705  0.5775 0.4591
Residuals         15 0.48585 0.032390
```

#### Stimulations


```
data_subset <- subset(df_pac_muni, Stim != "Placebo_loc" & Task == "3back")
data_subset <- droplevels(data_subset)
levels(data_subset$Stim)
```


```
[1] "real_frontal"      "real_parietal"     "real_in-phase"     "real_out-of-phase"
```


```
levels(data_subset$Task)
```


```
[1] "3back"
```


```
m_pac_mem_speed_n_u_stim <- lm(formula = Speed_n_u ~ Stim*PAC_mem, data=data_subset)
summary(m_pac_mem_speed_n_u_stim)
```


```
Call:
lm(formula = Speed_n_u ~ Stim * PAC_mem, data = data_subset)

Residuals:
     Min       1Q   Median       3Q      Max 
-0.32658 -0.10240 -0.01196  0.10915  0.39502 

Coefficients:
                               Estimate Std. Error t value Pr(>|t|)    
(Intercept)                     0.94132    0.03768  24.981   <2e-16 ***
Stimreal_parietal               0.02578    0.05329   0.484    0.630    
Stimreal_in-phase               0.02626    0.05329   0.493    0.624    
Stimreal_out-of-phase           0.08077    0.05329   1.516    0.134    
PAC_mem                        31.20463   20.69517   1.508    0.136    
Stimreal_parietal:PAC_mem     -28.48625   29.26740  -0.973    0.334    
Stimreal_in-phase:PAC_mem     -23.63526   29.26740  -0.808    0.422    
Stimreal_out-of-phase:PAC_mem -28.34310   29.26740  -0.968    0.336    
---
Signif. codes:  0 ‘***’ 0.001 ‘**’ 0.01 ‘*’ 0.05 ‘.’ 0.1 ‘ ’ 1

Residual standard error: 0.1635 on 68 degrees of freedom
  (4 observations deleted due to missingness)
Multiple R-squared:  0.06434,   Adjusted R-squared:  -0.03198 
F-statistic: 0.668 on 7 and 68 DF,  p-value: 0.6982
```


```
anova(m_pac_mem_speed_n_u_stim)
```


```
Analysis of Variance Table

Response: Speed_n_u
             Df  Sum Sq  Mean Sq F value Pr(>F)
Stim          3 0.05965 0.019884  0.7442 0.5295
PAC_mem       1 0.03068 0.030683  1.1483 0.2877
Stim:PAC_mem  3 0.03461 0.011537  0.4318 0.7309
Residuals    68 1.81692 0.026719
```


```
m_pac_targets_speed_n_u_stim <- lm(formula = Speed_n_u ~ Stim*PAC_front*PAC_par, data=data_subset)
summary(m_pac_targets_speed_n_u_stim)
```


```
Call:
lm(formula = Speed_n_u ~ Stim * PAC_front * PAC_par, data = data_subset)

Residuals:
     Min       1Q   Median       3Q      Max 
-0.32854 -0.10484  0.00775  0.08382  0.38449 

Coefficients:
                                          Estimate Std. Error t value Pr(>|t|)    
(Intercept)                              9.295e-01  4.306e-02  21.587   <2e-16 ***
Stimreal_parietal                        1.791e-02  6.089e-02   0.294   0.7697    
Stimreal_in-phase                        1.738e-02  6.089e-02   0.285   0.7763    
Stimreal_out-of-phase                    1.023e-01  6.089e-02   1.680   0.0983 .  
PAC_front                               -1.669e+01  2.029e+01  -0.822   0.4142    
PAC_par                                 -2.374e+01  2.751e+01  -0.863   0.3917    
Stimreal_parietal:PAC_front             -4.121e+00  2.870e+01  -0.144   0.8863    
Stimreal_in-phase:PAC_front             -1.096e+01  2.870e+01  -0.382   0.7039    
Stimreal_out-of-phase:PAC_front          2.264e+01  2.870e+01   0.789   0.4334    
Stimreal_parietal:PAC_par                2.463e+01  3.891e+01   0.633   0.5291    
Stimreal_in-phase:PAC_par                2.583e+01  3.891e+01   0.664   0.5093    
Stimreal_out-of-phase:PAC_par            2.536e+01  3.891e+01   0.652   0.5170    
PAC_front:PAC_par                       -1.444e+04  8.733e+03  -1.653   0.1035    
Stimreal_parietal:PAC_front:PAC_par      1.378e+04  1.235e+04   1.116   0.2689    
Stimreal_in-phase:PAC_front:PAC_par      2.733e+03  1.235e+04   0.221   0.8256    
Stimreal_out-of-phase:PAC_front:PAC_par  7.767e+03  1.235e+04   0.629   0.5318    
---
Signif. codes:  0 ‘***’ 0.001 ‘**’ 0.01 ‘*’ 0.05 ‘.’ 0.1 ‘ ’ 1

Residual standard error: 0.1621 on 60 degrees of freedom
  (4 observations deleted due to missingness)
Multiple R-squared:  0.1879,    Adjusted R-squared:  -0.01514 
F-statistic: 0.9254 on 15 and 60 DF,  p-value: 0.5417
```


```
anova(m_pac_targets_speed_n_u_stim)
```


```
Analysis of Variance Table

Response: Speed_n_u
                       Df  Sum Sq  Mean Sq F value  Pr(>F)  
Stim                    3 0.05965 0.019884  0.7565 0.52294  
PAC_front               1 0.01328 0.013280  0.5052 0.47996  
PAC_par                 1 0.07489 0.074885  2.8491 0.09662 .
Stim:PAC_front          3 0.04937 0.016456  0.6261 0.60094  
Stim:PAC_par            3 0.03311 0.011037  0.4199 0.73936  
PAC_front:PAC_par       1 0.09654 0.096536  3.6729 0.06007 .
Stim:PAC_front:PAC_par  3 0.03802 0.012673  0.4822 0.69591  
Residuals              60 1.57701 0.026284                  
---
Signif. codes:  0 ‘***’ 0.001 ‘**’ 0.01 ‘*’ 0.05 ‘.’ 0.1 ‘ ’ 1
```

LS0tDQp0aXRsZTogImlDT0cgTVMgc3RhdHMgZm9yIHRoZSBNVU5JIGRhdGEiDQpvdXRwdXQ6IGh0bWxfbm90ZWJvb2sNCi0tLQ0KDQpgYGB7cn0NCmxpYnJhcnkoTWF0cml4KQ0KbGlicmFyeShsbWU0KQ0KbGlicmFyeShjYXJEYXRhKQ0KbGlicmFyeShjYXIpDQpsaWJyYXJ5KGxtZXJUZXN0KQ0KbGlicmFyeShlbW1lYW5zKQ0KbGlicmFyeShlZmZlY3RzaXplKQ0KbGlicmFyeShnZ3Bsb3QyKQ0KbGlicmFyeShSbWlzYykNCmxpYnJhcnkoZmxleHBsb3QpDQpgYGANCmBgYHtyfQ0KY3Vycl9kaXIgPC0gZ2V0d2QoKQ0KZGF0YV9kaXJlY3RvcnkgPC0gZmlsZS5wYXRoKHBhc3RlKGN1cnJfZGlyLCAnLi4vLi4vLi4vQ29kZS9Ob3RlYm9va3MvUmVzdWx0cycsIHNlcD0nLycpKQ0KDQpmaWxlX25hbWUgPC0gZmlsZS5wYXRoKHBhc3RlKGRhdGFfZGlyZWN0b3J5LCAiaUNPR19hbGxfZGF0YS50eHQiLCBzZXA9Jy8nKSkNCmRmIDwtIHJlYWQuZGVsaW0oZmlsZV9uYW1lLCBoZWFkZXIgPSBUUlVFLCBuYS5zdHJpbmdzID0gIk5OIikNCmhlYWQoZGYsIDUpDQpgYGANCg0KYGBge3J9DQpkZiRJRCA8LSBhcy5mYWN0b3IoZGYkSUQpDQpkZiRTY2hvb2wgPC0gYXMuZmFjdG9yKGRmJFNjaG9vbCkNCmRmJERheSA8LSBhcy5mYWN0b3IoZGYkRGF5KQ0KZGYkVGFzayA8LSBhcy5mYWN0b3IoZGYkVGFzaykNCmRmJEJsb2NrIDwtIGFzLm51bWVyaWMoZGYkQmxvY2spDQpkZiRTdGltIDwtIGFzLmZhY3RvcihkZiRTdGltKQ0KZGYkQWNjIDwtIGFzLm51bWVyaWMoZGYkQWNjKQ0KZGYkU3BlZWRfY29yciA8LSBhcy5udW1lcmljKGRmJFNwZWVkX2NvcnIpDQpkZiREX3ByaW1lIDwtIGFzLm51bWVyaWMoZGYkRF9wcmltZSkNCmRmJEFjY19ub3JtIDwtIGFzLm51bWVyaWMoZGYkQWNjX25vcm0pDQpkZiRTcGVlZF9ub3JtIDwtIGFzLm51bWVyaWMoZGYkU3BlZWRfbm9ybSkNCmRmJERfcHJpbWVfbm9ybSA8LSBhcy5udW1lcmljKGRmJERfcHJpbWVfbm9ybSkNCg0KYGBgDQojIE1VTkkgRGF0YSBSZXN1bHRzDQpgYGB7cn0NCmRmX211bmkgPC0gc3Vic2V0KGRmLCBTY2hvb2wgPT0gJ01VTkknKQ0KZGZfbXVuaSA8LSBkcm9wbGV2ZWxzKGRmX211bmkpDQpkZl9tdW5pJFN0aW0gPC0gZmFjdG9yKGRmX211bmkkU3RpbSwgbGV2ZWxzKGRmX211bmkkU3RpbSlbYygxLCAyLCA1LCAzLCA0KV0pDQpsZXZlbHMoZGZfbXVuaSRTdGltKQ0KYGBgDQoNCiMjIEFjY3VyYWN5IFN0YXRzDQojIyMgU2ltcGxlIG1vZGVsLCBhdmVyYWdlcw0KSGVyZSwgd2UgcHJlc2VudCBkaWZmZXJlbnQgbW9kZWxzIHRvIGNoYXJhY3Rlcml6ZSBlYWNoIHJlbGV2YW50IHBhcmFtZXRlciByZWxhdGVkIHRvIHRoZSBwYXJ0aWNpcGFudHMnIHBlcmZvcm1hbmNlLiBJbiBlYWNoIGNhc2UsIHRoZSBtb2RlbHMgc3RhcnQgb2ZmIGZyb20gYSBzaW1wbGUNCmZvcm0gKGkuZS4sIGdyb3VwIGF2ZXJhZ2VzIHBlciBzZXNzaW9uKSwgYW5kIHRoZXkgZ3JvdyBpbiBjb21wbGV4aXR5IGFzIHdlIGFkZCBvdGhlciBmaXhlZCBmYWN0b3JzIChlLmcuLCB0cmFpbmluZyBibG9ja3MpIGFuZCByYW5kb20gZWZmZWN0cyB0byBhY2NvdW50IGZvciB0aGUgDQp2YXJpYWJpbGl0eSBhY3Jvc3Mgc3ViamVjdHMuIEVhY2ggbW9kZWwgaXMgY29tcGFyZWQgc3RhdGlzdGljYWxseSB0byBpdHMgKHNpbXBsZXIpIHByZWRlY2Vzc29yIHRvIGFzc2VzcyB3aGV0aGVyIHRoZSBldmVudHVhbCBpbXByb3ZlbWVudCBpbiBkYXRhIGV4cGxhaW5hYmlsaXR5LCANCmFzIHF1YW50aWZpZWQgYnkgdHdvIGNvbnZlbnRpb25hbCBjcml0ZXJpYSAoaS5lLiwgQWthaWtlJ3MgYW5kIEJheWVzJyBpbmZvcm1hdGlvbiBjcml0ZXJpYSksIGp1c3RpZmllcyB0aGUgdXNlIG9mIGEgbW9yZSBjb21wbGV4IG1vZGVsLiANCmBgYHtyfQ0KbTAgPC0gbG0oZm9ybXVsYSA9IEFjYyB+IFN0aW0qVGFzaywgZGF0YT1kZl9tdW5pKQ0Kc3VtbWFyeShtMCkNCmFub3ZhKG0wKQ0KYGBgDQojIyMgTm90ZToNCk5leHQsIHdlIHdpbGwgdGVzdCBhIG1vcmUgY29tcGxleCBtb2RlbCBpbmNsdWRpbmcgdGhlIHRlbXBvcmFsIGNvbXBvbmVudCBvZiB0aGUgdGFzayAoaS5lLiwgdGhlIHRyYWluaW5nIGJsb2NrcykuDQoNCiMjIyBMaW5lYXIgbW9kZWwsIG5vIHJhbmRvbSBlZmZlY3RzDQpgYGB7cn0NCm0xIDwtIGxtKGZvcm11bGEgPSBBY2MgfiBTdGltKlRhc2sqQmxvY2ssIGRhdGE9ZGZfbXVuaSkNCnN1bW1hcnkobTEpDQphbm92YShtMSkNCmBgYA0KIyMjIE5vdGU6DQpXaGVuIHVzaW5nIHRoaXMgbW9yZSBjb21wbGV4IG1vZGVsLCB0aGVyZSBpcyBvbmx5IGV2aWRlbmNlIGZvciB0aGUgdGFzayBkaWZmZXJlbmNlIHRoZSBwcmV2aW91cyBtb2RlbCBoYWQgc3VnZ2VzdGVkLiBXZSB3aWxsIG5vdyBjb21wYXJlIHRoZSB0d28gbW9kZWxzIHN0YXRpc3RpY2FsbHkuIA0KIyMjIyBDb21wYXJlIG1vZGVscw0KYGBge3J9DQphbm92YShtMCwgbTEpDQpgYGANCiMjIyBOb3RlOg0KVGhlIGFkZGl0aW9uIG9mIHRoZSBibG9ja3MgdG8gdGhlIG1vZGVsIGRvZXMgbm90IHNpZ25pZmljYW50bHkgaW1wcm92ZSB0aGUgYW1vdW50IG9mIHZhcmlhbmNlIGV4cGxhaW5lZCBieSB0aGUgbW9kZWwuIEhvd2V2ZXIsIGl0IG1heSBzdGlsbCBiZSB0aGF0IHRoZSBhZGRpdGlvbiBvZiByYW5kb20gZWZmZWN0cyB3aWxsIGltcHJvdmUgdGhlIG1vZGVsJ3MgYWJpbGl0eSB0byBleHBsYWluIHRoZSB2YXJpYW5jZSBpbiB0aGUgZGF0YS4gV2Ugd2lsbCBhc3Nlc3MgdGhlIG91dGNvbWUgb2YgdGhpcyBhZGRpdGlvbiBuZXh0Lg0KDQojIyMjIFJhbmRvbSBJbnRlcmNlcHQgcGVyIHN1YmplY3QNCmBgYHtyfQ0KbTIgPC0gbG1lcihmb3JtdWxhID0gQWNjIH4gU3RpbSpUYXNrICsgKDEgfCBJRCksIGRhdGE9ZGZfbXVuaSkNCnN1bW1hcnkobTIpDQphbm92YShtMikNCmBgYA0KIyMjIE5vdGU6DQpXaGVuIGFkZGluZyBhIHJhbmRvbSBpbnRlcmNlcHQgdG8gdGhlIG1vZGVsLCB0aGVyZSBpcyBhIHRyZW5kIHRvd2FyZHMgYSBzaWduaWZpY2FudCBkaWZmZXJlbmNlIGFtb25nIHRoZSBibG9ja3MuIFdlIHdpbGwgbm93IGFzc2VzcyB3aGV0aGVyIHRoZSBhbW91bnQgb2YgdmFyaWFuY2UgZXhwbGFpbmVkIGJ5IHRoaXMgbW9kZWwgaXMgc2lnbmlmaWNhbnRseSBsYXJnZXIgdGhhbiB0aGUgb25lIGV4cGxhaW5lZCBieSB0aGUgc2ltcGxlIG1vZGVsIChtMCksIGFzIG91ciB0ZXN0cyByZXZlYWxlZCBubyBpbXByb3ZlbWVudCBmcm9tIHRoZSBsaW5lYXIgbW9kZWwgKG0xKQ0KIyMjIyBDb21wYXJlIG1vZGVscw0KV2hlbiBjb21wYXJpbmcgYSBsaW5lYXIgbW9kZWwgKGxtKSB0byBhIGxpbmVhciBtaXhlZC1lZmZlY3QgbW9kZWwgKGxtZSksIHRoZSByZWd1bGFyIEFOT1ZBIGRvZXMgbm90IHdvcmssIHNvIEkgd2lsbCBvbmx5IGxvb2sgYXQgdGhlIGluZm9ybWF0aW9uIGNyaXRlcmlhDQpgYGB7cn0NCkFJQyhtMCwgbTIpDQpCSUMobTAsIG0yKQ0KYGBgDQojIyMgTm90ZToNClRoZSBtb2RlbCBpbmNsdWRpbmcgcmFuZG9tIGludGVyY2VwdHMgaXMgYmV0dGVyIHRoYW4gdGhlIHJlZ3VsYXIgbGluZWFyIG1vZGVsLiBXZSB3aWxsIHNlZSB3aGV0aGVyIGluY2x1ZGluZyBhIHJhbmRvbSBzbG9wZSBpbXByb3ZlcyB0aGUgbW9kZWwuDQoNCiMjIyBSYW5kb20gaW50ZXJjZXB0IGZvciBzdWJqZWN0cywgYW5kIGJsb2Nrcw0KYGBge3J9DQptMyA8LSBsbWVyKGZvcm11bGEgPSBBY2MgfiBTdGltKlRhc2sqQmxvY2sgKyAoMSB8IElEKSwgZGF0YT1kZl9tdW5pKQ0Kc3VtbWFyeShtMykNCmFub3ZhKG0zKQ0KYGBgDQpgYGB7cn0NCmFub3ZhKG0yLCBtMykNCmBgYA0KVGhlIGluY2x1c2lvbiBvZiB0aGUgYmxvY2tzIGRvZXMgbm90IHNpZ25pZmljYW50bHkgaW1wcm92ZSB0aGUgbW9kZWwNCg0KIyMjIyBSYW5kb20gaW50ZXJjZXB0IGFuZCByYW5kb20gc2xvcGUgcGVyIHN1YmplY3QNCmBgYHtyfQ0KbTQgPC0gbG1lcihmb3JtdWxhID0gQWNjIH4gU3RpbSpUYXNrKkJsb2NrICsgKDEgKyBCbG9ja3xJRCksIGRhdGE9ZGZfbXVuaSkNCnN1bW1hcnkobTQpDQphbm92YShtNCkNCmBgYA0KIyMjIyBDb21wYXJlIG1vZGVscywgZXZlbiB0aG91Z2ggdGhpcyBsYXN0IHdhcyBzaW5ndWxhcg0KYGBge3J9DQphbm92YShtMiwgbTQpDQpgYGANCiMjIyBOb3RlOg0KVGhlIGFkZGl0aW9uIG9mIGEgcmFuZG9tIHNsb3BlIGZvciBlYWNoIHN1YmplY3QgZG9lcyBub3Qgc2lnbmlmaWNhbnRseSBpbXByb3ZlIHRoZSBhbW91bnQgb2YgdmFyaWFuY2UgZXhwbGFpbmVkIGJ5IHRoZSBtb2RlbC4gDQoNCiMjIyBSYW5kb20gaW50ZXJjZXB0IHBlciBzdWJqZWN0IGFuZCBwZXIgZGF5DQpgYGB7cn0NCm01IDwtIGxtZXIoZm9ybXVsYSA9IEFjYyB+IFN0aW0qVGFzayArICgxICsgKDF8SUQpICsgKDF8RGF5KSksIGRhdGE9ZGZfbXVuaSkNCnN1bW1hcnkobTUpDQphbm92YShtNSkNCmBgYA0KYGBge3J9DQphbm92YShtMiwgbTUpDQpgYGANCg0KIyMjIE1vZGVsIGNob2ljZQ0KVGhlIG1vZGVsIGluY2x1ZGluZyBhIHJhbmRvbSBpbnRlcmNlcHQgZm9yIGRheSBhbmQgZm9yIHN1YmplY3QgaXMgc2lnbmlmaWNhbnRseSBiZXR0ZXIgdGhhbiB0aGUgb25lIGluY2x1ZGluZyBvbmx5IHRoZSByYW5kb20gaW50ZXJjZXB0IGZvciBzdWJqZWN0LiBBcyBhIGxhc3QgdGVzdCwgd2Ugd2lsbCBzZWUgaWYgYWRkaW5nIHRoZSBibG9ja3MgdG8gbTUgaW1wcm92ZXMgdGhlIG1vZGVsLg0KDQpgYGB7cn0NCm02IDwtIGxtZXIoZm9ybXVsYSA9IEFjYyB+IFN0aW0qVGFzaypCbG9jayArICgxICsgKDF8SUQpICsgKDF8RGF5KSksIGRhdGE9ZGZfbXVuaSkNCnN1bW1hcnkobTYpDQphbm92YShtNikNCmBgYA0KYGBge3J9DQphbm92YShtNSwgbTYpDQpgYGANCiMjIyBNb2RlbCBjaG9pY2UNCkFkZGluZyB0aGUgYmxvY2tzIHRvIHRoZSBtb2RlbCBpbmNsdWRpbmcgcmFuZG9tIGludGVyY2VwdHMgZm9yIHN1YmplY3RzIGFuZCBkYXlzIGRvZXMgbm90IGltcHJvdmUgdGhlIG1vZGVsLiBUaGVyZWZvcmUsIHdlIHdpbGwga2VlcCBtNSBhcyB0aGUgbW9kZWwgZm9yIGFjY3VyYWN5Lg0KDQojIyMgTW9kZWwgZm9yIGFjY3VyYWN5DQpgYGB7cn0NCm1fYWNjIDwtIGxtZXIoZm9ybXVsYSA9IEFjYyB+IFN0aW0qVGFzayArICgxICsgKDF8SUQpICsgKDF8RGF5KSksIGRhdGE9ZGZfbXVuaSkNCnN1bW1hcnkobV9hY2MpDQphbm92YShtX2FjYykNCmV0YV9zcXVhcmVkKG1fYWNjKQ0KYGBgDQpUaGVyZSBpcyBhIHNpZ25pZmljYW50IGRpZmZlcmVuY2UgYW1vbmcgdGhlIHRhc2tzLCBzbyB3ZSB3aWxsIHRlc3QgdGhlbSBzZXBhcmF0ZWx5Lg0KDQojIyMgQWNjdXJhY3kgaW4gdGhlIDItYmFjayB0YXNrDQoNCmBgYHtyfQ0KZGF0YV9zdWJzZXQgPC0gc3Vic2V0KGRmX211bmksIFRhc2sgPT0gJzJiYWNrJykNCmRhdGFfc3Vic2V0IDwtIGRyb3BsZXZlbHMoZGF0YV9zdWJzZXQpDQpsZXZlbHMoZGF0YV9zdWJzZXQkVGFzaykNCmBgYA0KYGBge3J9DQptX2FjY18yYmFjayA8LSBsbWVyKGZvcm11bGEgPSBBY2MgfiBTdGltICsgKDEgKyAoMXxJRCkgKyAoMXxEYXkpKSwgZGF0YT1kYXRhX3N1YnNldCkNCnN1bW1hcnkobV9hY2NfMmJhY2spDQphbm92YShtX2FjY18yYmFjaykNCmV0YV9zcXVhcmVkKG1fYWNjXzJiYWNrKQ0KYGBgDQojIyMgTm90ZToNClRoZXJlIGlzIG5vIGV2aWRlbmNlIGZvciBhIHNpZ25pZmljYW50IGVmZmVjdCBvZiBzdGltdWxhdGlvbi4NCg0KIyMjIEFjY3VyYWN5IGluIHRoZSAzLWJhY2sgdGFzaw0KYGBge3J9DQpkYXRhX3N1YnNldCA8LSBzdWJzZXQoZGZfbXVuaSwgVGFzayA9PSAnM2JhY2snKQ0KZGF0YV9zdWJzZXQgPC0gZHJvcGxldmVscyhkYXRhX3N1YnNldCkNCmxldmVscyhkYXRhX3N1YnNldCRUYXNrKQ0KYGBgDQpgYGB7cn0NCm1fYWNjXzNiYWNrIDwtIGxtZXIoZm9ybXVsYSA9IEFjYyB+IFN0aW0gKyAoMSArICgxfElEKSArICgxfERheSkpLCBkYXRhPWRhdGFfc3Vic2V0KQ0Kc3VtbWFyeShtX2FjY18zYmFjaykNCmFub3ZhKG1fYWNjXzNiYWNrKQ0KZXRhX3NxdWFyZWQobV9hY2NfM2JhY2spDQpgYGANCiMjIyBOb3RlOg0KVGhlcmUgaXMgbm8gZXZpZGVuY2UgZm9yIHNpZ25pZmljYW50IGRpZmZlcmVuY2VzIGluIHRoZSAzLWJhY2sgdGFzay4NCg0KIyMgTm9ybWFsaXplZCBhY2N1cmFjeQ0KSHVtYW4gYmVoYXZpb3IgdGVuZHMgdG8gYmUgaGlnaGx5IHZhcmlhYmxlIGFjcm9zcyBzdWJqZWN0cywgd2hpY2ggc29tZXRpbWVzIG1ha2VzIGEgZGlyZWN0IGNvbXBhcmlzb24gb2YgcGVyZm9ybWFuY2UgY2hhbGxlbmdpbmcuIEZvciB0aGlzIHJlYXNvbiwgd2Ugd2lsbCBub3cNCmFzc2VzcyB0aGUgY2hhbmdlIGluIGFjY3VyYWN5IGV4cGVyaWVuY2VkIGJ5IGVhY2ggcGFydGljaXBhbnQsIHdoaWNoIHdlIGludGVuZCB0byB1c2UgYXMgYSBjb3JyZWN0aW9uIGZvciBuYXRpdmUgZGlmZmVyZW5jZXMgcHJlc2VudCBhdCB0aGUgc3RhcnQgb2YgZWFjaCBzZXNzaW9uLg0KVGhpcyBjb3JyZWN0aW9uIHdhcyBkb25lIGJ5IGRpdmlkaW5nIHRoZSBhY2N1cmFjeSBzY29yZSBvZiBlYWNoIGJsb2NrIGJ5IHRoYXQgb2YgdGhlIGZpcnN0IHRyYWluaW5nIGJsb2NrIG9mIGVhY2ggc2Vzc2lvbi4gUGxlYXNlIG5vdGUgdGhpcyBjb3JyZWN0aW9uIHdhcyBkb25lIA0Kd2l0aGluIGVhY2ggc2Vzc2lvbiAoaS5lLiwgdXNpbmcgdGhlIGZpcnN0IHRyYWluaW5nIGJsb2NrIG9mIGVhY2ggc2Vzc2lvbiksIGFzIG9wcG9zZWQgdG8gdXNpbmcgdGhlIGZpcnN0IGJsb2NrIG9mIHRyYWluaW5nIGV2ZXIgcGVyZm9ybWVkIGJ5IGVhY2ggcGFydGljaXBhbnQuIA0KDQojIyMgR3JvdXAgYXZlcmFnZXMNCmBgYHtyfQ0KbTAgPC0gbG0oZm9ybXVsYSA9IEFjY19ub3JtIH4gU3RpbSpUYXNrLCBkYXRhPWRmX211bmkpDQpzdW1tYXJ5KG0wKQ0KYW5vdmEobTApDQpgYGANCkluY2x1ZGUgdGhlIGJsb2Nrcw0KYGBge3J9DQptMSA8LSBsbShmb3JtdWxhID0gQWNjX25vcm0gfiBTdGltKlRhc2sqQmxvY2ssIGRhdGE9ZGZfbXVuaSkNCnN1bW1hcnkobTEpDQphbm92YShtMSkNCmBgYA0KDQpgYGB7cn0NCmFub3ZhKG0wLCBtMSkNCmBgYA0KIyMjIE5vdGU6DQpUaGUgaW5jbHVzaW9uIG9mIHRoZSBibG9ja3MgaW4gdGhlIG1vZGVsIGRpZCBub3Qgc2lnbmlmaWNhbnRseSBpbXByb3ZlIHRoZSBhbW91bnQgb2YgdmFyaWFuY2UgZXhwbGFpbmVkIGJ5IHRoZSBtb2RlbC4gV2Ugd2lsbCBub3cgdGVzdCB3aGV0aGVyIHRoZSBpbmNsdXNpb24gb2YgcmFuZG9tIGVmZmVjdHMgYnJpbmdzIGFueSBpbXByb3ZlbWVudC4gDQojIyMgSW5jbHVkZSByYW5kb20gZWZmZWN0cw0KIyMjIyBSYW5kb20gaW50ZXJjZXB0IHBlciBzdWJqZWN0DQpgYGB7cn0NCm0yIDwtIGxtZXIoZm9ybXVsYSA9IEFjY19ub3JtIH4gU3RpbSpUYXNrICsgKDEgfCBJRCksIGRhdGE9ZGZfbXVuaSkNCnN1bW1hcnkobTIpDQphbm92YShtMikNCmBgYA0KIyMjIE5vdGU6IA0KVGhlIGluY2x1c2lvbiBvZiBhIHJhbmRvbSBpbnRlcmNlcHQgcmVzdWx0cyBpbiBhIG1vZGVsIHByb3ZpZGluZyBldmlkZW5jZSBmb3IgYSBzaWduaWZpY2FudCBlZmZlY3Qgb2Ygc3RpbXVsYXRpb24sIHdoaWNoIGRpZmZlcnMgYW1vbmcgdGhlIHRhc2tzLiBXZSB3aWxsIG5vdyBjb21wYXJlIHRoaXMgbW9kZWwgdG8gdGhlIGJlc3QgbW9kZWwgd2UgaGFkIGJlZm9yZSAobTApDQpgYGB7cn0NCkFJQyhtMCwgbTIpDQpCSUMobTAsIG0yKQ0KYGBgDQojIyMgTm90ZToNCkluIHRoaXMgY2FzZSwgdGhlIG1vZGVsIGluY2x1ZGluZyByYW5kb20gZWZmZWN0cyBpbXByb3ZlcyB0aGUgYW1vdW50IG9mIHZhcmlhbmNlIGV4cGxhaW5lZCBieSB0aGUgbW9kZWwuDQpgYGB7cn0NCm0zIDwtIGxtZXIoZm9ybXVsYSA9IEFjY19ub3JtIH4gU3RpbSpUYXNrKkJsb2NrICsgKDEgfCBJRCksIGRhdGE9ZGZfbXVuaSkNCnN1bW1hcnkobTMpDQphbm92YShtMykNCmBgYA0KYGBge3J9DQphbm92YShtMiwgbTMpDQpgYGANCkluY2x1ZGluZyB0aGUgYmxvY2tzIGRvZXMgbm90IHNpZ25pZmljYW50bHkgaW1wcm92ZSB0aGUgbW9kZWwuIFdlIHdpbGwgbm93IHRlc3Qgd2hldGhlciBhIHJhbmRvbSBpbnRlcmNlcHQgZm9yIHRoZSBkYXlzIGltcHJvdmVzIGl0Lg0KDQpgYGB7cn0NCm00IDwtIGxtZXIoZm9ybXVsYSA9IEFjY19ub3JtIH4gU3RpbSpUYXNrICsgKDEgKyAoMXxJRCkgKyAoMXxEYXkpKSwgZGF0YT1kZl9tdW5pKQ0Kc3VtbWFyeShtNCkNCmFub3ZhKG00KQ0KYGBgDQpgYGB7cn0NCmFub3ZhKG0yLCBtNCkNCmBgYA0KQWRkaW5nIGEgcmFuZG9tIGludGVyY2VwdCBmb3IgZGF5cyBpbXByb3ZlcyB0aGUgbW9kZWwuDQpgYGB7cn0NCm01IDwtIGxtZXIoZm9ybXVsYSA9IEFjY19ub3JtIH4gU3RpbSpUYXNrKkJsb2NrICsgKDEgKyAoMXxJRCkgKyAoMXxEYXkpKSwgZGF0YT1kZl9tdW5pKQ0Kc3VtbWFyeShtNSkNCmFub3ZhKG01KQ0KYGBgDQpgYGB7cn0NCmFub3ZhKG00LCBtNSkNCmBgYA0KQWRkaW5nIHRoZSBibG9ja3MgZG9lcyBub3QgaW1wcm92ZSBtNS4gV2Ugd2lsbCBsYXN0IHRlc3QgdGhlIGVmZmVjdCBvZiBhZGRpbmcgYSByYW5kb20gc2xvcGUuDQpgYGB7cn0NCm02IDwtIGxtZXIoZm9ybXVsYSA9IEFjY19ub3JtIH4gU3RpbSpUYXNrKkJsb2NrICsgKDEgKyBCbG9ja3xJRCksIGRhdGE9ZGZfbXVuaSkNCnN1bW1hcnkobTYpDQphbm92YShtNikNCmBgYA0KIyMjIE1vZGVsIGNob2ljZQ0KVGhlIGZpdCBvZiB0aGUgbW9kZWwgaW5jbHVkaW5nIGEgcmFuZG9tIHNsb3BlIGlzIHNpbmd1bGFyLCB3aGljaCB3YXMgdG8gYmUgZXhwZWN0ZWQgYXMgdGhlIGluY2x1c2lvbiBvZiB0aGUgYmxvY2tzIGluIHRoZSBkaWZmZXJlbnQgdGVzdGVkIG1vZGVscyBuZXZlciBpbXByb3ZlZCB0aGUgYW1vdW50IG9mIHZhcmlhbmNlIGV4cGxhaW5lZC4gVGhlcmVmb3JlLCB3ZSB3aWxsIGtlZXAgbTQsIHdoaWNoIGluY2x1ZGUgcmFuZG9tIGludGVyY2VwdHMgZm9yIGluZGl2aWR1YWxzIGFuZCBmb3IgdGhlIGRheXMuDQoNCmBgYHtyfQ0KbV9hY2Nfbm9ybSA8LSBsbWVyKGZvcm11bGEgPSBBY2Nfbm9ybSB+IFN0aW0qVGFzayArICgxICsgKDF8SUQpICsgKDF8RGF5KSksIGRhdGE9ZGZfbXVuaSkNCnN1bW1hcnkobV9hY2Nfbm9ybSkNCmFub3ZhKG1fYWNjX25vcm0pDQpldGFfc3F1YXJlZChtX2FjY19ub3JtKQ0KYGBgDQpgYGB7cn0NCm15X21vZGVsIDwtIG1fYWNjX25vcm0NCmxldmVsXzEgPC0gIlN0aW0iDQoNCm15X21vZGVsLmNvbXBhcmUgPC0gZW1tZWFucyhteV9tb2RlbCwgbGV2ZWxfMSwgYnk9IlRhc2siKQ0KbXlfbW9kZWwuY29tcGFyZS5wYWlycyA8LSBwYWlycyhteV9tb2RlbC5jb21wYXJlLCBhZGp1c3Q9J3R1a2V5JykNCnRlc3QobXlfbW9kZWwuY29tcGFyZS5wYWlycywgc2lkZT0ndHdvLXNpZGVkJykNCmNvbmZpbnQobXlfbW9kZWwuY29tcGFyZSwgY2FsYyA9IGMobiA9IH4ud2d0LikpDQplZmZfc2l6ZShteV9tb2RlbC5jb21wYXJlLCBzaWdtYSA9IHNpZ21hKG15X21vZGVsKSwgZWRmID0gMjMpDQpgYGANCg0KIyMjIE5vcm1hbGl6ZWQgYWNjdXJhY3kgaW4gdGhlIDItYmFjayB0YXNrDQpgYGB7cn0NCmRhdGFfc3Vic2V0IDwtIHN1YnNldChkZl9tdW5pLCBUYXNrID09ICcyYmFjaycpDQpkYXRhX3N1YnNldCA8LSBkcm9wbGV2ZWxzKGRhdGFfc3Vic2V0KQ0KbGV2ZWxzKGRhdGFfc3Vic2V0JFRhc2spDQpgYGANCmBgYHtyfQ0KbV9hY2Nfbm9ybV8yYmFjayA8LSBsbWVyKGZvcm11bGEgPSBBY2Nfbm9ybSB+IFN0aW0gKyAoMSArICgxfElEKSArICgxfERheSkpLCBkYXRhPWRhdGFfc3Vic2V0KQ0Kc3VtbWFyeShtX2FjY19ub3JtXzJiYWNrKQ0KYW5vdmEobV9hY2Nfbm9ybV8yYmFjaykNCmV0YV9zcXVhcmVkKG1fYWNjX25vcm1fMmJhY2spDQpgYGANCiMjIyBOb3RlOg0KVGhlcmUgaXMgYW4gZWZmZWN0IG9mIHN0aW11bGF0aW9uIGluIHRoZSAyLWJhY2sgdGFzay4gV2Ugd2lsbCBub3cgZG8gcG9zdC1ob2MgY29tcGFyaXNvbnMgdG8gc2VlIHdoZXJlIHRoZXNlIGRpZmZlcmVuY2VzIGNvbWUgZnJvbS4NCmBgYHtyfQ0KbXlfbW9kZWwgPC0gbV9hY2Nfbm9ybV8yYmFjaw0KbGV2ZWxfMSA8LSAiU3RpbSINCg0KbXlfbW9kZWwuY29tcGFyZSA8LSBlbW1lYW5zKG15X21vZGVsLCBsZXZlbF8xKQ0KbXlfbW9kZWwuY29tcGFyZS5wYWlycyA8LSBwYWlycyhteV9tb2RlbC5jb21wYXJlLCBhZGp1c3Q9J3R1a2V5JykNCnRlc3QobXlfbW9kZWwuY29tcGFyZS5wYWlycywgc2lkZT0ndHdvLXNpZGVkJykNCmNvbmZpbnQobXlfbW9kZWwuY29tcGFyZSwgY2FsYyA9IGMobiA9IH4ud2d0LikpDQplZmZfc2l6ZShteV9tb2RlbC5jb21wYXJlLCBzaWdtYSA9IHNpZ21hKG15X21vZGVsKSwgZWRmID0gMjMpDQpgYGANCiMjIyBOb3RlOg0KVGhlIHBvc3QtaG9jIHRlc3RzIHJldmVhbGVkIHRoYXQgdGhlIGltcHJvdmVtZW50IGluIHRoZSBhY2N1cmFjeSB3YXMgc2lnbmlmaWNhbnRseSBoaWdoZXIgd2hlbiBwYXJ0aWNpcGFudHMgcmVjZWl2ZWQgaW4tcGhhc2Ugc3RpbXVsYXRpb24uIEhvd2V2ZXIsIGV4cGxvcmF0aW9uIG9mIHRoZSBpbmRpdmlkdWFsIHNjb3JlcyBvYnRhaW5lZCBieSBwYXJ0aWNpcGFudHMgcmV2ZWFsZWQgdGhlIHByZXNlbmNlIG9mIHdoYXQgY291bGQgYmUgYW4gb3V0bGllciwgd2l0aCBhIHNpbmdsZSBwYXJ0aWNpcGFudCAoOTAwNCkgaGF2aW5nIGEgc2luZ2xlIHNjb3JlIGFib3ZlIDQgKGkuZS4sIDQwMCUgaW1wcm92ZW1lbnQgaW4gb25lIG9mIHRoZSAxMyBibG9ja3MpLiBUaGVyZWZvcmUsIHdlIHdpbGwgbm93IHNob3cgdGhlIHNhbWUgYW5hbHlzaXMgYW5kIHRoZSByZXN1bHRzIG9idGFpbmVkIHdoZW4gZXhjbHVkaW5nIHRoaXMgcGFydGljaXBhbnQuDQpgYGB7cn0NCmRhdGFfc3Vic2V0IDwtIHN1YnNldChkZl9tdW5pLCBUYXNrID09ICcyYmFjaycgJiBJRCAhPSAiOTAwNCIpDQpkYXRhX3N1YnNldCA8LSBkcm9wbGV2ZWxzKGRhdGFfc3Vic2V0KQ0KbGV2ZWxzKGRhdGFfc3Vic2V0JFRhc2spDQpsZXZlbHMoZGF0YV9zdWJzZXQkSUQpDQpgYGANCmBgYHtyfQ0KbV9hY2Nfbm9ybV8yYmFja19leGNsdWRlXzkwMDQgPC0gbG1lcihmb3JtdWxhID0gQWNjX25vcm0gfiBTdGltICsgKDEgKyAoMXxJRCkgKyAoMXxEYXkpKSwgZGF0YT1kYXRhX3N1YnNldCkNCnN1bW1hcnkobV9hY2Nfbm9ybV8yYmFja19leGNsdWRlXzkwMDQpDQphbm92YShtX2FjY19ub3JtXzJiYWNrX2V4Y2x1ZGVfOTAwNCkNCmV0YV9zcXVhcmVkKG1fYWNjX25vcm1fMmJhY2tfZXhjbHVkZV85MDA0KQ0KYGBgDQpgYGB7cn0NCm15X21vZGVsIDwtIG1fYWNjX25vcm1fMmJhY2tfZXhjbHVkZV85MDA0DQpsZXZlbF8xIDwtICJTdGltIg0KDQpteV9tb2RlbC5jb21wYXJlIDwtIGVtbWVhbnMobXlfbW9kZWwsIGxldmVsXzEpDQpteV9tb2RlbC5jb21wYXJlLnBhaXJzIDwtIHBhaXJzKG15X21vZGVsLmNvbXBhcmUsIGFkanVzdD0ndHVrZXknKQ0KdGVzdChteV9tb2RlbC5jb21wYXJlLnBhaXJzLCBzaWRlPSd0d28tc2lkZWQnKQ0KY29uZmludChteV9tb2RlbC5jb21wYXJlLCBjYWxjID0gYyhuID0gfi53Z3QuKSkNCmVmZl9zaXplKG15X21vZGVsLmNvbXBhcmUsIHNpZ21hID0gc2lnbWEobXlfbW9kZWwpLCBlZGYgPSAyMykNCmBgYA0KIyMjIE5vdGU6DQpUaGUgbW9kZWwgcmV2ZWFsZWQgdGhhdCwgd2hlbiBleGNsdWRpbmcgc3ViamVjdCA5MDA0IGZyb20gdGhlIGFuYWx5c2lzLCB0aGUgY2hhbmdlIGluIGFjY3VyYWN5IGluIHRoZSBwYXJ0aWNpcGFudHMgd2FzIG5vIGxvbmdlciBzaWduaWZpY2FudGx5IGRpZmZlcmVudCB3aGVuIHJlY2VpdmluZyBpbi1waGFzZSBzdGltdWxhdGlvbiBjb21wYXJlZCB0byB3aGVuIHRoZXkgcmVjZWl2ZWQgdGhlIHBsYWNlYm8uDQoNCiMjIyBOb3JtYWxpemVkIGFjY3VyYWN5IGluIHRoZSAzLWJhY2sgdGFzaw0KYGBge3J9DQpkYXRhX3N1YnNldCA8LSBzdWJzZXQoZGZfbXVuaSwgVGFzayA9PSAnM2JhY2snKQ0KZGF0YV9zdWJzZXQgPC0gZHJvcGxldmVscyhkYXRhX3N1YnNldCkNCmxldmVscyhkYXRhX3N1YnNldCRUYXNrKQ0KYGBgDQpgYGB7cn0NCm1fYWNjX25vcm1fM2JhY2sgPC0gbG1lcihmb3JtdWxhID0gQWNjX25vcm0gfiBTdGltICsgKDEgKyAoMXxJRCkgKyAoMXxEYXkpKSwgZGF0YT1kYXRhX3N1YnNldCkNCnN1bW1hcnkobV9hY2Nfbm9ybV8zYmFjaykNCmFub3ZhKG1fYWNjX25vcm1fM2JhY2spDQpldGFfc3F1YXJlZChtX2FjY19ub3JtXzNiYWNrKQ0KYGBgDQpgYGB7cn0NCm15X21vZGVsIDwtIG1fYWNjX25vcm1fM2JhY2sNCmxldmVsXzEgPC0gIlN0aW0iDQoNCm15X21vZGVsLmNvbXBhcmUgPC0gZW1tZWFucyhteV9tb2RlbCwgbGV2ZWxfMSkNCm15X21vZGVsLmNvbXBhcmUucGFpcnMgPC0gcGFpcnMobXlfbW9kZWwuY29tcGFyZSwgYWRqdXN0PSd0dWtleScpDQp0ZXN0KG15X21vZGVsLmNvbXBhcmUucGFpcnMsIHNpZGU9J3R3by1zaWRlZCcpDQpjb25maW50KG15X21vZGVsLmNvbXBhcmUsIGNhbGMgPSBjKG4gPSB+LndndC4pKQ0KZWZmX3NpemUobXlfbW9kZWwuY29tcGFyZSwgc2lnbWEgPSBzaWdtYShteV9tb2RlbCksIGVkZiA9IDIzKQ0KYGBgDQoNCiMjIFNwZWVkDQpOb3csIHdlIHByZXNlbnQgdGhlIHNhbWUgdHlwZSBvZiBzdGF0aXN0aWNhbCBhbmFseXNpcyBvbiB0aGUgc3BlZWQgb2YgdGhlIHBhcnRpY2lwYW50cy4gUGxlYXNlIG5vdGUgZm9yIHRoaXMgY29tcGFyaXNvbiwgd2UgY29uc2lkZXIgdGhlIHNwZWVkIG9mIGNvcnJlY3QNCnRyaWFscyBvbmx5Lg0KDQoNCmBgYHtyfQ0KbTAgPC0gbG0oZm9ybXVsYSA9IFNwZWVkX2NvcnIgfiBTdGltKlRhc2ssIGRhdGE9ZGZfbXVuaSkNCnN1bW1hcnkobTApDQphbm92YShtMCkNCmBgYA0KIyMjIE5vdGU6IFRoaXMgbW9kZWwgc2hvd3MgYSBzaWduaWZjYW50IGRpZmZlcmVuY2UgYW1vbmcgdGhlIHRhc2tzLiBXZSB3aWxsIG5vdyBpbmNsdWRlIHRoZSBibG9ja3MgaW4gdGhlIG1vZGVsIGFuZCBjb21wYXJlIGJvdGggbW9kZWxzIHN0YXRpc3RpY2FsbHkuDQojIyMgTGluZWFyIG1vZGVsLCBubyByYW5kb20gZWZmZWN0cw0KYGBge3J9DQptMSA8LSBsbShmb3JtdWxhID0gU3BlZWRfY29yciB+IFN0aW0qVGFzaypCbG9jaywgZGF0YT1kZl9tdW5pKQ0Kc3VtbWFyeShtMSkNCmFub3ZhKG0xKQ0KYGBgDQojIyMjIENvbXBhcmUgbW9kZWxzDQpgYGB7cn0NCmFub3ZhKG0wLCBtMSkNCmBgYA0KVGhlIGFtb3VudCBvZiB2YXJpYW5jZSBleHBsYWluZWQgYnkgdGhlIG1vZGVsIGlzIG5vdCBzdGF0aXN0aWNhbGx5IGxhcmdlciB0aGFuIGluIGluIHRoZSBzaW1wbGVyIG1vZGVsIChtMCksIHNvIHRoZSBpbmNsdXNpb24gb2YgdGhlIGJsb2NrcyBpcyBub3QganVzdGlmaWVkLiBXZSB3aWxsIG5vdyBhc3Nlc3MgbW9kZWxzIGluY2x1ZGluZyByYW5kb20gZWZmZWN0cy4NCg0KYGBge3J9DQptMiA8LSBsbWVyKGZvcm11bGEgPSBTcGVlZF9jb3JyIH4gU3RpbSpUYXNrICsgKDEgfCBJRCksIGRhdGE9ZGZfbXVuaSkNCnN1bW1hcnkobTIpDQphbm92YShtMikNCmBgYA0KDQpgYGB7cn0NCkFJQyhtMCwgbTIpDQpCSUMobTAsIG0yKQ0KYGBgDQoNClRoZSBtb2RlbCBpbmNsdWRpbmcgcmFuZG9tIGludGVyY2VwdHMgaXMgYmV0dGVyIHRoYW4gdGhlIHJlZ3VsYXIgbGluZWFyIG1vZGVsIGluIHRoaXMgY2FzZS4NCg0KYGBge3J9DQptMyA8LSBsbWVyKGZvcm11bGEgPSBTcGVlZF9jb3JyIH4gU3RpbSpUYXNrKkJsb2NrICsgKDEgfCBJRCksIGRhdGE9ZGZfbXVuaSkNCnN1bW1hcnkobTMpDQphbm92YShtMykNCmBgYA0KYGBge3J9DQphbm92YShtMiwgbTMpDQpgYGANCkluIHRoaXMgY2FzZSwgdGhlIG1vZGVsIGNvbXBhcmlzb24gc2VlbXMgdG8geWllbGQgY29udHJhZGljdG9yeSByZXN1bHRzLCBhcyBhY2NvcmRpbmcgdG8gdGhlIEFJQywgdGhlIHNlY29uZCBtb2RlbCBpcyBiZXR0ZXIsIHdoaWxlIGFjY29yZGluZyB0byB0aGUgQklDLCB0aGUgZmlyc3QgbW9kZWwgaXMgYmV0dGVyLiBUaGUgQklDIGlzIGNvbnNpZGVyZWQgdG8gYmUgYSBtb3JlIHJvYnVzdCBtZWFzdXJlLCBhcyBpdCB0YWtlcyBpbnRvIGFjY291bnQgdGhlIHNhbXBsZSBzaXplcyBhbmQgaXMgdGhvdWdodCB0byBiZSBhIGJldHRlciBjaG9pY2UgZm9yIG1vZGVsIHNlbGVjdGlvbiwgd2hpbGUgQUlDIGlzIGNvbnNpZGVyZWQgYmV0dGVyIGluIHNlbGVjdGluZyBhIG1vZGVsIHRvIG1ha2UgcHJlZGljdGlvbnMgKHNlZSBDaGFrcmFiYXJ0aSBhbmQgR2hvc2gsIDIwMTEpLiBBY2NvcmRpbmcgdG8gdGhlIEJJQywgdGhlIGFkZGl0aW9uIG9mIHRoZSBibG9ja3Mgd29yc2VucyB0aGUgbW9kZWwsIHNvIHdlIHdpbGwgbGVhdmUgdGhlbSBvdXQuDQoNCiMjIyMgUmFuZG9tIGludGVyY2VwdCBhbmQgcmFuZG9tIHNsb3BlIHBlciBzdWJqZWN0DQpgYGB7cn0NCm00IDwtIGxtZXIoZm9ybXVsYSA9IFNwZWVkX2NvcnIgfiBTdGltKlRhc2sqQmxvY2sgKyAoMSArIEJsb2NrfElEKSwgZGF0YT1kZl9tdW5pKQ0Kc3VtbWFyeShtNCkNCmFub3ZhKG00KQ0KYGBgDQojIyMjIENvbXBhcmUgbW9kZWxzLCBldmVuIHRob3VnaCB0aGlzIGxhc3Qgb25lIGRpZCBub3QgY29udmVyZ2UNCmBgYHtyfQ0KYW5vdmEobTIsIG00KQ0KYGBgDQpCYXNlZCBvbiB0aGUgQklDLCBhZGRpbmcgYSByYW5kb20gc2xvcGUgd29yc2VucyB0aGUgYW1vdW50IG9mIHZhcmlhbmNlIGV4cGxhaW5lZCBieSB0aGUgbW9kZWwgaW5jbHVkaW5nIG9ubHkgYSByYW5kb20gaW50ZXJjZXB0LiANCg0KYGBge3J9DQptNSA8LSBsbWVyKGZvcm11bGEgPSBTcGVlZF9jb3JyIH4gU3RpbSpUYXNrICsgKDEgKyAoMXxJRCkgKyAoMXxEYXkpKSwgZGF0YT1kZl9tdW5pKQ0Kc3VtbWFyeShtNSkNCmFub3ZhKG01KQ0KYGBgDQpgYGB7cn0NCmFub3ZhKG0yLCBtNSkNCmBgYA0KIyMjIE1vZGVsIGNob2ljZQ0KQmFzZWQgb24gdGhlIEJJQywgd2hpY2ggaXMgdGhvdWdodCB0byBiZSBhIG1vcmUgcm9idXN0IHBhcmFtZXRlciBmb3IgbW9kZWwgc2VsZWN0aW9uIHRoYW4gQUlDIChhcyBtZW50aW9uZWQgcHJldmlvdXNseSksIHRoZSBiZXN0IG1vZGVsIGlzIG01LCBpbmNsdWRpbmcgcmFuZG9tIGludGVyY2VwdHMgZm9yIGJvdGggc3ViamVjdHMgYW5kIGRheXMuIEhvd2V2ZXIsIG01IGRpZCBub3QgY29udmVyZ2UsIHNvIHdlIHdpbGwgdGVzdCB3aGV0aGVyIHRoZSBhZGRpdGlvbiBvZiB0aGUgYmxvY2tzIGluIHRoZSBtb2RlbCBpbXByb3ZlcyB0aGUgZXN0aW1hdGUuDQoNCmBgYHtyfQ0KbTYgPC0gbG1lcihmb3JtdWxhID0gU3BlZWRfY29yciB+IFN0aW0qVGFzaypCbG9jayArICgxICsgKDF8SUQpICsgKDF8RGF5KSksIGRhdGE9ZGZfbXVuaSkNCnN1bW1hcnkobTYpDQphbm92YShtNikNCmBgYA0KYGBge3J9DQphbm92YShtMiwgbTYpDQpgYGANCiMjIyBNb2RlbCBjaG9pY2UNClRoZSBtb2RlbCBpbmNsdWRpbmcgcmFuZG9tIGludGVyY2VwdHMgZm9yIGJvdGggc3ViamVjdHMgYW5kIGRheXMgc2lnbmlmaWNhbnRseSB3b3JzZW5zIHRoZSBhbW91bnQgb2YgdmFyaWFuY2UgZXhwbGFpbmVkIGJ5IHRoZSBtb2RlbCwgc28gd2Ugd2lsbCBrZWVwIG0yLCBpbmNsdWRpbmcgb25seSByYW5kb20gaW50ZXJjZXB0cyBmb3Igc3ViamVjdHMuDQoNCg0KYGBge3J9DQptX3NwZWVkIDwtIGxtZXIoZm9ybXVsYSA9IFNwZWVkX2NvcnIgfiBTdGltKlRhc2sgKyAoMSB8IElEKSwgZGF0YT1kZl9tdW5pKQ0Kc3VtbWFyeShtX3NwZWVkKQ0KYW5vdmEobV9zcGVlZCkNCmV0YV9zcXVhcmVkKG1fc3BlZWQpDQpgYGANCiMjIyBOb3RlOiANClRoZXJlIGlzIGEgc2lnbmlmaWNhbnQgZGlmZmVyZW5jZSBiZXR3ZWVuIHRoZSB0YXNrcy4gV2Ugd2lsbCBtYWtlIG1vZGVscyBmb3IgZWFjaCB0YXNrIHNlcGFyYXRlbHkuDQoNCiMjIyBTcGVlZCBpbiB0aGUgMi1iYWNrIHRhc2sNCmBgYHtyfQ0KZGF0YV9zdWJzZXQgPC0gc3Vic2V0KGRmX211bmksIFRhc2sgPT0gJzJiYWNrJykNCmRhdGFfc3Vic2V0IDwtIGRyb3BsZXZlbHMoZGF0YV9zdWJzZXQpDQpsZXZlbHMoZGF0YV9zdWJzZXQkVGFzaykNCmBgYA0KYGBge3J9DQptX3NwZWVkXzJiYWNrIDwtIGxtZXIoZm9ybXVsYSA9IFNwZWVkX2NvcnIgfiBTdGltICsgKDEgfCBJRCksIGRhdGE9ZGF0YV9zdWJzZXQpDQpzdW1tYXJ5KG1fc3BlZWRfMmJhY2spDQphbm92YShtX3NwZWVkXzJiYWNrKQ0KZXRhX3NxdWFyZWQobV9zcGVlZF8yYmFjaykNCmBgYA0KIyMjIE5vdGU6DQpUaGVyZSBpcyBubyBldmlkZW5jZSBmb3IgYSBzaWduaWZpY2FudCBlZmZlY3Qgb2Ygc3RpbXVsYXRpb24uDQoNCiMjIyBTcGVlZCBpbiB0aGUgMy1iYWNrIHRhc2sNCmBgYHtyfQ0KZGF0YV9zdWJzZXQgPC0gc3Vic2V0KGRmX211bmksIFRhc2sgPT0gJzNiYWNrJykNCmRhdGFfc3Vic2V0IDwtIGRyb3BsZXZlbHMoZGF0YV9zdWJzZXQpDQpsZXZlbHMoZGF0YV9zdWJzZXQkVGFzaykNCmBgYA0KYGBge3J9DQptX3NwZWVkXzNiYWNrIDwtIGxtZXIoZm9ybXVsYSA9IFNwZWVkX2NvcnIgfiBTdGltICsgKDEgfCBJRCksIGRhdGE9ZGF0YV9zdWJzZXQpDQpzdW1tYXJ5KG1fc3BlZWRfM2JhY2spDQphbm92YShtX3NwZWVkXzNiYWNrKQ0KZXRhX3NxdWFyZWQobV9zcGVlZF8zYmFjaykNCmBgYA0KIyMjIE5vdGU6DQpUaGVyZSBpcyBubyBldmlkZW5jZSBmb3IgYSBzaWduaWZpY2FudCBlZmZlY3Qgb2Ygc3RpbXVsYXRpb24gb24gdGhlIHNwZWVkIG9mIHRoZSAzLWJhY2sgdGFzay4NCg0KIyMgTm9ybWFsaXplZCBTcGVlZA0KDQpgYGB7cn0NCm0wIDwtIGxtKGZvcm11bGEgPSBTcGVlZF9ub3JtIH4gU3RpbSpUYXNrLCBkYXRhPWRmX211bmkpDQpzdW1tYXJ5KG0wKQ0KYW5vdmEobTApDQpgYGANCg0KYGBge3J9DQptMSA8LSBsbShmb3JtdWxhID0gU3BlZWRfbm9ybSB+IFN0aW0qVGFzaypCbG9jaywgZGF0YT1kZl9tdW5pKQ0Kc3VtbWFyeShtMSkNCmFub3ZhKG0xKQ0KYGBgDQpgYGB7cn0NCmFub3ZhKG0wLCBtMSkNCmBgYA0KVGhlIG1vZGVsIGluY2x1ZGluZyB0aGUgYmxvY2tzIGRvZXMgbm90IHNpZ25pZmljYW50bHkgaW1wcm92ZSB0aGUgcHJvcG9ydGlvbiBvZiB0aGUgZGF0YSBleHBsYWluZWQgYnkgdGhlIG1vZGVsLiBXZSB3aWxsIG5vdyBhc3Nlc3Mgd2hldGhlciB0aGUgYWRkaXRpb24gb2YgcmFuZG9tIGVmZmVjdHMgaW1wcm92ZXMgdGhlIGxpbmVhciBtb2RlbC4NCg0KYGBge3J9DQptMiA8LSBsbWVyKGZvcm11bGEgPSBTcGVlZF9ub3JtIH4gU3RpbSpUYXNrICsgKDEgfCBJRCksIGRhdGE9ZGZfbXVuaSkNCnN1bW1hcnkobTIpDQphbm92YShtMikNCmBgYA0KYGBge3J9DQpBSUMobTAsIG0yKQ0KQklDKG0wLCBtMikNCmBgYA0KVGhlIGFkZGl0aW9uIG9mIHRoZSByYW5kb20gaW50ZXJjZXB0IGltcHJvdmVzIHRoZSBtb2RlbC4gV2Ugd2lsbCBub3cgdGVzdCBpZiBhZGRpbmcgdGhlIGJsb2NrcyBkb2VzIGFzIHdlbGwuDQoNCmBgYHtyfQ0KbTMgPC0gbG1lcihmb3JtdWxhID0gU3BlZWRfbm9ybSB+IFN0aW0qVGFzaypCbG9jayArICgxIHwgSUQpLCBkYXRhPWRmX211bmkpDQpzdW1tYXJ5KG0zKQ0KYW5vdmEobTMpDQpgYGANCmBgYHtyfQ0KYW5vdmEobTIsIG0zKQ0KYGBgDQpBZGRpbmcgdGhlIGJsb2NrcyB0byBtMiBkb2VzIG5vdCBzaWduaWZpY2FudGx5IGltcHJvdmUgdGhlIG1vZGVsLiBOZXh0LCB3ZSB3aWxsIHRlc3Qgd2hldGhlciBhIHJhbmRvbSBzbG9wZSBpbXByb3ZlcyBpdC4NCg0KDQpgYGB7cn0NCm00IDwtIGxtZXIoZm9ybXVsYSA9IFNwZWVkX25vcm0gfiBTdGltKlRhc2sqQmxvY2sgKyAoMSArIEJsb2NrfElEKSwgZGF0YT1kZl9tdW5pKQ0Kc3VtbWFyeShtNCkNCmFub3ZhKG00KQ0KYGBgDQoNCmBgYHtyfQ0KYW5vdmEobTIsIG00KQ0KYGBgDQpBZGRpbmcgYSByYW5kb20gc2xvcGUgc2lnbmlmaWNhbnRseSB3b3JzZW5zIHRoZSBtb2RlbCBhY2NvcmRpbmcgdG8gdGhlIEJJQy4gTmV4dCwgd2Ugd2lsbCB0ZXN0IHRoZSBhZGRpdGlvbiBvZiBhIHJhbmRvbSBpbnRlcmNlcHQgZm9yIHRoZSBkYXlzLg0KDQpgYGB7cn0NCm01IDwtIGxtZXIoZm9ybXVsYSA9IFNwZWVkX25vcm0gfiBTdGltKlRhc2sgKyAoMSArICgxfElEKSArICgxfERheSkpLCBkYXRhPWRmX211bmkpDQpzdW1tYXJ5KG01KQ0KYW5vdmEobTUpDQpgYGANCmBgYHtyfQ0KYW5vdmEobTIsIG01KQ0KYGBgDQpUaGUgbW9kZWwgaW5jbHVkaW5nIHJhbmRvbSBpbnRlcmNlcHRzIGZvciBkYXlzIGFuZCBzdWJqZWN0cyBpbXByb3ZlcyB0aGUgb25lIGluY2x1ZGluZyBvbmx5IGEgcmFuZG9tIGludGVyY2VwdCBmb3Igc3ViamVjdHMuIA0KDQpgYGB7cn0NCm02IDwtIGxtZXIoZm9ybXVsYSA9IFNwZWVkX25vcm0gfiBTdGltKlRhc2sqQmxvY2sgKyAoMSArICgxfElEKSArICgxfERheSkpLCBkYXRhPWRmX211bmkpDQpzdW1tYXJ5KG02KQ0KYW5vdmEobTYpDQpgYGANCmBgYHtyfQ0KYW5vdmEobTUsIG02KQ0KYGBgDQpBZGRpbmcgdGhlIGJsb2NrcyB0byBtNSBkb2VzIG5vdCBzaWduaWZpY2FudGx5IGltcHJvdmUgaXQsIHNvIHdlIHdpbGwga2VlcCBtNS4NCg0KYGBge3J9DQptX3NwZWVkX25vcm0gPC0gbG1lcihmb3JtdWxhID0gU3BlZWRfbm9ybSB+IFN0aW0qVGFzayArICgxICsgKDF8SUQpICsgKDF8RGF5KSksIGRhdGE9ZGZfbXVuaSkNCnN1bW1hcnkobV9zcGVlZF9ub3JtKQ0KYW5vdmEobV9zcGVlZF9ub3JtKQ0KZXRhX3NxdWFyZWQobV9zcGVlZF9ub3JtKQ0KYGBgDQoNCiMjIyBOb3JtYWxpemVkIHNwZWVkIGluIHRoZSAyLWJhY2sgdGFzaw0KYGBge3J9DQpkYXRhX3N1YnNldCA8LSBzdWJzZXQoZGZfbXVuaSwgVGFzayA9PSAnMmJhY2snKQ0KZGF0YV9zdWJzZXQgPC0gZHJvcGxldmVscyhkYXRhX3N1YnNldCkNCmxldmVscyhkYXRhX3N1YnNldCRUYXNrKQ0KYGBgDQpgYGB7cn0NCm1fc3BlZWRfbm9ybV8yYmFjayA8LSBsbWVyKGZvcm11bGEgPSBTcGVlZF9ub3JtIH4gU3RpbSArICgxICsgKDF8SUQpICsgKDF8RGF5KSksIGRhdGE9ZGF0YV9zdWJzZXQpDQpzdW1tYXJ5KG1fc3BlZWRfbm9ybV8yYmFjaykNCmFub3ZhKG1fc3BlZWRfbm9ybV8yYmFjaykNCmV0YV9zcXVhcmVkKG1fc3BlZWRfbm9ybV8yYmFjaykNCmBgYA0KYGBge3J9DQpteV9tb2RlbCA8LSBtX3NwZWVkX25vcm1fMmJhY2sNCmxldmVsXzEgPC0gIlN0aW0iDQoNCm15X21vZGVsLmNvbXBhcmUgPC0gZW1tZWFucyhteV9tb2RlbCwgbGV2ZWxfMSkNCm15X21vZGVsLmNvbXBhcmUucGFpcnMgPC0gcGFpcnMobXlfbW9kZWwuY29tcGFyZSwgYWRqdXN0PSd0dWtleScpDQp0ZXN0KG15X21vZGVsLmNvbXBhcmUucGFpcnMsIHNpZGU9J3R3by1zaWRlZCcpDQpjb25maW50KG15X21vZGVsLmNvbXBhcmUsIGNhbGMgPSBjKG4gPSB+LndndC4pKQ0KZWZmX3NpemUobXlfbW9kZWwuY29tcGFyZSwgc2lnbWEgPSBzaWdtYShteV9tb2RlbCksIGVkZiA9IDIzKQ0KYGBgDQpFdmVuIHRob3VnaCB0aGVyZSBpcyBhIHNpZ25pZmljYW50IGVmZmVjdCBvZiBzdGltdWxhdGlvbiwgdGhlcmUgaXMgbm8gZXZpZGVuY2UgZm9yIGRpZmZlcmVuY2UgYmV0d2VlbiBhbnkgb2YgdGhlIHN0aW11bGF0aW9uIHZhcmlhbnRzIGFuZCB0aGUgcGxhY2Viby4NCg0KIyMjIE5vcm1hbGl6ZWQgYWNjdXJhY3kgaW4gdGhlIDMtYmFjayB0YXNrDQpgYGB7cn0NCmRhdGFfc3Vic2V0IDwtIHN1YnNldChkZl9tdW5pLCBUYXNrID09ICczYmFjaycpDQpkYXRhX3N1YnNldCA8LSBkcm9wbGV2ZWxzKGRhdGFfc3Vic2V0KQ0KbGV2ZWxzKGRhdGFfc3Vic2V0JFRhc2spDQpgYGANCmBgYHtyfQ0KbV9zcGVlZF9ub3JtXzNiYWNrIDwtIGxtZXIoZm9ybXVsYSA9IFNwZWVkX25vcm0gfiBTdGltICsgKDEgKyAoMXxJRCkgKyAoMXxEYXkpKSwgZGF0YT1kYXRhX3N1YnNldCkNCnN1bW1hcnkobV9zcGVlZF9ub3JtXzNiYWNrKQ0KYW5vdmEobV9zcGVlZF9ub3JtXzNiYWNrKQ0KZXRhX3NxdWFyZWQobV9zcGVlZF9ub3JtXzNiYWNrKQ0KYGBgDQpgYGB7cn0NCm15X21vZGVsIDwtIG1fc3BlZWRfbm9ybV8zYmFjaw0KbGV2ZWxfMSA8LSAiU3RpbSINCg0KbXlfbW9kZWwuY29tcGFyZSA8LSBlbW1lYW5zKG15X21vZGVsLCBsZXZlbF8xKQ0KbXlfbW9kZWwuY29tcGFyZS5wYWlycyA8LSBwYWlycyhteV9tb2RlbC5jb21wYXJlLCBhZGp1c3Q9J3R1a2V5JykNCnRlc3QobXlfbW9kZWwuY29tcGFyZS5wYWlycywgc2lkZT0ndHdvLXNpZGVkJykNCmNvbmZpbnQobXlfbW9kZWwuY29tcGFyZSwgY2FsYyA9IGMobiA9IH4ud2d0LikpDQplZmZfc2l6ZShteV9tb2RlbC5jb21wYXJlLCBzaWdtYSA9IHNpZ21hKG15X21vZGVsKSwgZWRmID0gMjMpDQpgYGANCiMjIFN0YXRpc3RpY2FsIGFuYWx5c2lzIHJlbGF0aW5nIHRoZSBlc3RpbWF0ZWQgaW5kdWNlZCBlbGVjdHJpYyBmaWVsZHMgYW5kIGJlaGF2aW9yLg0KYGBge3J9DQpmaWxlX25hbWUgPC0gZmlsZS5wYXRoKHBhc3RlKGRhdGFfZGlyZWN0b3J5LCAiQmVoYXZpb3JhbF9hbmRfZWZpZWxkX2Jsb2Nrc19pQ09HX0Rlc2lnbl8yNV8wNF8yMDIzLnR4dCIsIHNlcD0nLycpKQ0KZGZfZWZpZWxkcyA8LSByZWFkLmRlbGltKGZpbGVfbmFtZSwgaGVhZGVyID0gVFJVRSwgbmEuc3RyaW5ncyA9ICJOTiIpDQpoZWFkKGRmX2VmaWVsZHMsIDUpDQpgYGANCmBgYHtyfQ0KZGZfZWZpZWxkcyRJRCA8LSBhcy5mYWN0b3IoZGZfZWZpZWxkcyRJRCkNCmRmX2VmaWVsZHMkU2Nob29sIDwtIGFzLmZhY3RvcihkZl9lZmllbGRzJFNjaG9vbCkNCmRmX2VmaWVsZHMkRGF5IDwtIGFzLmZhY3RvcihkZl9lZmllbGRzJERheSkNCmRmX2VmaWVsZHMkVGFzayA8LSBhcy5mYWN0b3IoZGZfZWZpZWxkcyRUYXNrKQ0KZGZfZWZpZWxkcyRTdGltIDwtIGFzLmZhY3RvcihkZl9lZmllbGRzJFN0aW0pDQpkZl9lZmllbGRzJEFjY191IDwtIGFzLm51bWVyaWMoZGZfZWZpZWxkcyRBY2NfdSkNCmRmX2VmaWVsZHMkU3BlZWRfdSA8LSBhcy5udW1lcmljKGRmX2VmaWVsZHMkU3BlZWRfdSkNCmRmX2VmaWVsZHMkQWNjX20gPC0gYXMubnVtZXJpYyhkZl9lZmllbGRzJEFjY19tKQ0KZGZfZWZpZWxkcyRBY2Nfbl9tIDwtIGFzLm51bWVyaWMoZGZfZWZpZWxkcyRBY2Nfbl9tKQ0KZGZfZWZpZWxkcyRTcGVlZF9tIDwtIGFzLm51bWVyaWMoZGZfZWZpZWxkcyRTcGVlZF9tKQ0KZGZfZWZpZWxkcyRTcGVlZF9uX20gPC0gYXMubnVtZXJpYyhkZl9lZmllbGRzJFNwZWVkX25fbSkNCmRmX2VmaWVsZHMkRUZfZnJvbnQgPC0gYXMubnVtZXJpYyhkZl9lZmllbGRzJEVGX2Zyb250KQ0KZGZfZWZpZWxkcyRFRl9wYXIgPC0gYXMubnVtZXJpYyhkZl9lZmllbGRzJEVGX3BhcikNCmRmX2VmaWVsZHMkRUZfbWFnIDwtIGFzLm51bWVyaWMoZGZfZWZpZWxkcyRFRl9tYWcpDQpkZl9lZmllbGRzJEVycm9yX2Zyb250IDwtIGFzLm51bWVyaWMoZGZfZWZpZWxkcyRFcnJvcl9mcm9udCkNCmRmX2VmaWVsZHMkRXJyb3JfcGFyIDwtIGFzLm51bWVyaWMoZGZfZWZpZWxkcyRFcnJvcl9wYXIpDQpgYGANCmBgYHtyfQ0KZGZfZWZfbXVuaSA8LSBzdWJzZXQoZGZfZWZpZWxkcywgU2Nob29sID09ICdNVU5JJykNCmRmX2VmX211bmkgPC0gZHJvcGxldmVscyhkZl9lZl9tdW5pKQ0KI2xldmVscyhkZl9lZl9lcGZsJFNjaG9vbCkNCmhlYWQoZGZfZWZfbXVuaSwgNSkNCmBgYA0KYGBge3J9DQpkZl9lZl9tdW5pJFN0aW0gPC0gZmFjdG9yKGRmX2VmX211bmkkU3RpbSwgbGV2ZWxzKGRmX2VmX211bmkkU3RpbSlbYygxLCAyLCA1LCAzLCA0KV0pDQpsZXZlbHMoZGZfZWZfbXVuaSRTdGltKQ0KYGBgDQoNCiMjIyBOb3RlIGFib3V0IHRoZSBhbmFseXNpcw0KVGhlIGlkZWEgb2YgdGhpcyBhbmFseXNpcyBpcyB0byB2ZXJpZnkgd2hldGhlciB0aGVyZSB3YXMgYSBkaWZmZXJlbnRpYXRlZCBlZmZlY3Qgb2Ygc3RpbXVsYXRpb24gcmVzdWx0aW5nIGZyb20gdGhlIG1hZ25pdHVkZSBvZiB0aGUgZWxlY3RyaWMgZmllbGRzIGluZHVjZWQgYXQgYm90aCBzdGltdWxhdGlvbiBzaXRlcyBhdCBhbiBpbmRpdmlkdWFsIGxldmVsLiBUaGUgbWFpbiBhbmFseXNpcyBkZXNjcmliZWQgYWJvdmUgc2hvd2VkIHNpZ25pZmljYW50IGVmZmVjdHMgb2Ygc3RpbXVsYXRpb24gaW4gdGhlIG5vcm1hbGl6ZWQgZGF0YSwgd2hpY2ggZGVwaWN0cyByZWxhdGl2ZSBjaGFuZ2VzIGluIHBlcmZvcm1hbmNlIGZvciBlYWNoIGluZGl2aWR1YWwuIEZvciB0aGlzIHJlYXNvbiwgd2Ugd2lsbCBhc3Nlc3MgdGhlIGVmZmVjdCBvZiB0aGUgbWFnbml0dWRlIG9mIHRoZSBpbmR1Y2VkIGVsZWN0cmljIGZpZWxkcyBvbiB0aGUgY2hhbmdlIGluIHBlcmZvcm1hbmNlIChpLmUuLCBpbiB0ZXJtcyBvZiBzcGVlZCBhbmQgYWNjdXJhY3kpIHdpdGhpbiBlYWNoIHRyYWluaW5nIHNlc3Npb24uIFRoaXMgY2hhbmdlIGluIHBlcmZvcm1hbmNlIGlzIGRlc2NyaWJlZCBhcyB0aGUgc2xvcGUgb2YgdGhlIGxpbmUgbWluaW1pemluZyB0aGUgZXJyb3IgaW4gYSBsZWFzdC1zcXVhcmVkIHNlbnNlLCBmaXR0ZWQgdG8gZWFjaCBpbmRpdmlkdWFsLiANCg0KVGhpcyBkYXRhIGZyYW1lIGNvbnRhaW5zIHRoZSBtYWduaXR1ZGUgb2YgdGhlIGVzdGltYXRlZCBlbGVjdHJpY2FsIGZpZWxkIGZvciBlYWNoIGVsZWN0cm9kZSAoaS5lLiwgZnJvbnRhbCBhbmQgcGFyaWV0YWwpLiBUaGUgZWxlY3Ryb2RlIGxvY2F0aW9ucyB3ZXJlIGRldGVybWluZWQgYnkgaGF2aW5nIHBhcnRpY2lwYW50cyBwZXJmb3JtIHRoZSAyLWJhY2sgdGFzayBpbnNpZGUgdGhlIE1SSSBzY2FubmVyIGFuZCBmaW5kaW5nIHRoZSByZWdpb24gd2VyZSB0aGUgdGFzay1yZWxhdGVkIGJvbGQgc2lnbmFsIHBlYWtlZC4gVGhpcyByZWdpb25zIHdlcmUgdGhlbiBwcm9qZWN0ZWQgaW50byB0aGUgc2NhbHAgb2YgZWFjaCBwYXJ0aWNpcGFudCBhbmQgdGhlIHJlc3VsdGluZyBjb29yZGluYXRlcyB3ZXJlIHVzZWQgdG8gcGxhY2UgdGhlIGVsZWN0cm9kZXMuIEluIHRoZSBkYXRhIGZyYW1lLCB0aGUgY29sdW1ucyBsYWJlbGVkIGFzICJFRiIgY29udGFpbiB0aGUgbWFnbml0dWRlIG9mIHRoZSBlbGVjdHJpYyBmaWVsZCBlc3RpbWF0ZWQgZm9yIGVhY2ggaW50ZW5kZWQgdGFyZ2V0LCB3aGlsZSB0aGUgY29sdW1ucyBsYWJlbGVkIHdpdGggImVycm9yIiBpbmNsdWRlIHRoZSBkaXN0YW5jZSBiZXR3ZWVuIHRoZSBpbnRlbmRlZCB0YXJnZXQgYW5kIHRoZSBsb2NhdGlvbiB3ZXJlIHRoZSBtYXhpbXVtIGVsZWN0cmljIGZpZWxkIHdhcyBpbmR1Y2VkLiBUaGUgY29sdW1uIGxhYmVsZWQgIkVGX21hZyIgY29udGFpbnMgdGhlIG1hZ25pdHVkZSBvZiB0aGUgZWxlY3RyaWMgZmllbGQgaW5kdWNlZCBvbiBib3RoIHNpdGVzIChtYWcgPSBzcXJ0KGFeMiArIGJeMikpLiBUaGUgY29sdW1uICJFRl9zeW0iIGNvbnRhaW5zIHRoZSByYXRpbyBvZiB0aGUgZWxlY3RyaWMgZmllbGQgaW5kdWNlZCBvbiB0aGUgZnJvbnRhbCBzaXRlIHRvIHRoZSBlbGVjdHJpYyBmaWVsZCBpbmR1Y2VkIGluIHRoZSBwYXJpZXRhbCBzaXRlIChFRl9mcm9udC9FRl9wYXIpLCBhbmQgY29uc3RpdHV0ZXMgYSBtZWFzdXJlIG9mIGhvdyBzeW1tZXRyaWMgdGhlIGluZHVjZWQgZmllbGRzIHdlcmUuIEJ5IGRlc2lnbiBhbmQgdW5kZXIgaWRlYWwgY2lyY3Vtc3RhbmNlcywgdGhlIGluZHVjZWQgZmllbGRzIHdvdWxkIGJlIGlkZW50aWNhbCwgYnV0IGFuYXRvbWljYWwgZGlmZmVyZW5jZXMgYW1vbmcgdGhlIHNpdGVzIG9mdGVuIHJlc3VsdCBpbiBkaWZmZXJlbnQgZmllbGRzIGJlaW5nIGluZHVjZWQuDQoNCkluIHRoZSBNVU5JIGNvaG9ydHMsIHRoZSBpbnRlbnNpdHkgb2YgdGhlIGFwcGxpZWQgY3VycmVudCB3YXMgb2YgMiBtQSBwZWFrLXRvLXBlYWsuIFRoZSBmaXJzdCB0d28gc3RpbXVsYXRpb24gY29uZGl0aW9ucyBpbnZvbHZlZCBtb25vZm9jYWwgc3RpbXVsYXRpb24gKGkuZS4sIGVpdGhlciBmcm9udGFsIG9yIHBhcmlldGFsKSwgd2hpbGUgY29uZGl0aW9uIDMgYW5kIGNvbmRpdGlvbiA0IGludm9sdmVkIGJpZm9jYWwgc3RpbXVsYXRpb24sIGFwcGxpZWQgZWl0aGVyIGluIHBoYXNlIChjb25kaXRpb24gMykgb3Igb3V0IG9mIHBoYXNlIChjb25kaXRpb24gNCkuIFdlIHdpbGwgYXNzZXNzIHRoZSBpbmZsdWVuY2Ugb2YgdGhlIG1hZ25pdHVkZSBvZiB0aGUgaW5kdWNlZCBlbGVjdHJpYyBmaWVsZHMgb24gYmVoYXZpb3IgY29uc2lkZXJpbmcgdGhlIG1vbm9mb2NhbCBhbmQgdGhlIGJpZm9jYWwgc3RpbXVsYXRpb24gY29uZGl0aW9ucyBzZXBhcmF0ZWx5Lg0KDQoNCiMjIyBUaGUgcmVsYXRpb25zaGlwIGJldHdlZW4gdGhlIGludGVuc2l0eSBvZiB0aGUgYXBwbGllZCBlbGVjdHJpYyBmaWVsZCBvbiB0aGUgdGFyZ2V0IGFuZCB0aGUgYWNjdXJhY3kNCg0KIyMjIE1vbm9mb2NhbCBjb25kaXRpb25zDQpGb3IgdGhlIG1vbm9mb2NhbCBjb25kaXRpb25zLCB0aGUgdmFyaWFibGUgd2Ugd2lsbCBiZSB1c2luZyBmb3IgdGhlIGZpZWxkcyBpcyAiRUZfbWFnIi4gVGhpcyB2YXJpYWJsZSBpcyB0aGUgbWFnbml0dWRlIG9mIHRoZSB2ZWN0b3IgZGVzY3JpYmluZyB0aGUgZWxlY3RyaWMgZmllbGQgaW4gdGVybXMgb2YgYSBmcm9udGFsIGFuZCBhIHBhcmlldGFsIGNvbXBvbmVudCwgYnV0IGFzIHRoZXNlIHN0aW11bGF0aW9uIHZhcmlhbnRzIGFyZSBtb25vZm9jYWwsIHRoZSB2YWx1ZSBmb3IgRUZfbWFnIGlzIHRoZSBzYW1lIGFzIGZvciBFRl9mcm9udCBpbiB0aGUgZnJvbnRhbCBjb25kaXRpb24gYW5kIEVGX3BhciBpbiB0aGUgcGFyaWV0YWwgY29uZGl0aW9uLg0KYGBge3J9DQpkYXRhX3N1YnNldCA8LSBzdWJzZXQoZGZfZWZfbXVuaSwgU3RpbSA9PSAicmVhbF9mcm9udGFsIiB8IFN0aW0gPT0gInJlYWxfcGFyaWV0YWwiKQ0KZGF0YV9zdWJzZXQgPC0gZHJvcGxldmVscyhkYXRhX3N1YnNldCkNCmxldmVscyhkYXRhX3N1YnNldCRTdGltKQ0KYGBgDQoNCmBgYHtyfQ0KbTAgPC0gbG0oZm9ybXVsYSA9IEFjY19tIH4gVGFzaypTdGltKkVGX21hZywgZGF0YT1kYXRhX3N1YnNldCkNCnN1bW1hcnkobTApDQphbm92YShtMCkNCmBgYA0KYGBge3J9DQptMSA8LSBsbWVyKGZvcm11bGEgPSBBY2NfbSB+IFRhc2sqU3RpbSpFRl9tYWcgKyAoMSB8IElEKSwgZGF0YT1kYXRhX3N1YnNldCkNCnN1bW1hcnkobTEpDQphbm92YShtMSkNCmBgYA0KYGBge3J9DQpBSUMobTAsIG0xKQ0KQklDKG0wLCBtMSkNCmBgYA0KYGBge3J9DQptX2FjY19lZiA8LSBsbShmb3JtdWxhID0gQWNjX20gfiBUYXNrKlN0aW0qRUZfbWFnLCBkYXRhPWRhdGFfc3Vic2V0KQ0Kc3VtbWFyeShtX2FjY19lZikNCmFub3ZhKG1fYWNjX2VmKQ0KZXRhX3NxdWFyZWQobV9hY2NfZWYpDQpgYGANCmBgYHtyfQ0KbTAgPC0gbG0oZm9ybXVsYSA9IEFjY19uX20gfiBUYXNrKlN0aW0qRUZfbWFnLCBkYXRhPWRhdGFfc3Vic2V0KQ0Kc3VtbWFyeShtMCkNCmFub3ZhKG0wKQ0KYGBgDQpgYGB7cn0NCm0xIDwtIGxtZXIoZm9ybXVsYSA9IEFjY19uX20gfiBUYXNrKlN0aW0qRUZfbWFnICsgKDEgfCBJRCksIGRhdGE9ZGF0YV9zdWJzZXQpDQpzdW1tYXJ5KG0xKQ0KYW5vdmEobTEpDQpgYGANCmBgYHtyfQ0KQUlDKG0wLCBtMSkNCkJJQyhtMCwgbTEpDQpgYGANCmBgYHtyfQ0KbV9hY2Nfbl9lZiA8LSBsbShmb3JtdWxhID0gQWNjX25fbSB+IFRhc2sqU3RpbSpFRl9tYWcsIGRhdGE9ZGF0YV9zdWJzZXQpDQpzdW1tYXJ5KG1fYWNjX25fZWYpDQphbm92YShtX2FjY19uX2VmKQ0KZXRhX3NxdWFyZWQobV9hY2Nfbl9lZikNCmBgYA0KYGBge3J9DQptMCA8LSBsbShmb3JtdWxhID0gU3BlZWRfbSB+IFRhc2sqU3RpbSpFRl9tYWcsIGRhdGE9ZGF0YV9zdWJzZXQpDQpzdW1tYXJ5KG0wKQ0KYW5vdmEobTApDQpgYGANCmBgYHtyfQ0KbTEgPC0gbG1lcihmb3JtdWxhID0gU3BlZWRfbSB+IFRhc2sqU3RpbSpFRl9tYWcgKyAoMSB8IElEKSwgZGF0YT1kYXRhX3N1YnNldCkNCnN1bW1hcnkobTEpDQphbm92YShtMSkNCmBgYA0KYGBge3J9DQpBSUMobTAsIG0xKQ0KQklDKG0wLCBtMSkNCmBgYA0KYGBge3J9DQptX3NwZWVkX2VmIDwtIGxtZXIoZm9ybXVsYSA9IFNwZWVkX20gfiBUYXNrKlN0aW0qRUZfbWFnICsgKDEgfCBJRCksIGRhdGE9ZGF0YV9zdWJzZXQpDQpzdW1tYXJ5KG1fc3BlZWRfZWYpDQphbm92YShtX3NwZWVkX2VmKQ0KZXRhX3NxdWFyZWQobV9zcGVlZF9lZikNCmBgYA0KYGBge3J9DQpkYXRhX3N1YnNldCA8LSBzdWJzZXQoZGZfZWZfbXVuaSwgKFN0aW0gPT0gInJlYWxfZnJvbnRhbCIgfCBTdGltID09ICJyZWFsX3BhcmlldGFsIikgJiBUYXNrID09ICcyYmFjaycpDQpkYXRhX3N1YnNldCA8LSBkcm9wbGV2ZWxzKGRhdGFfc3Vic2V0KQ0KbGV2ZWxzKGRhdGFfc3Vic2V0JFN0aW0pDQpsZXZlbHMoZGF0YV9zdWJzZXQkVGFzaykNCmBgYA0KYGBge3J9DQptX3NwZWVkX2VmXzJiYWNrIDwtIGxtZXIoZm9ybXVsYSA9IFNwZWVkX20gfiBTdGltKkVGX21hZyArICgxIHwgSUQpLCBkYXRhPWRhdGFfc3Vic2V0KQ0Kc3VtbWFyeShtX3NwZWVkX2VmXzJiYWNrKQ0KYW5vdmEobV9zcGVlZF9lZl8yYmFjaykNCmV0YV9zcXVhcmVkKG1fc3BlZWRfZWZfMmJhY2spDQpgYGANCmBgYHtyfQ0KZGF0YV9zdWJzZXQgPC0gc3Vic2V0KGRmX2VmX211bmksIChTdGltID09ICJyZWFsX2Zyb250YWwiIHwgU3RpbSA9PSAicmVhbF9wYXJpZXRhbCIpICYgVGFzayA9PSAnM2JhY2snKQ0KZGF0YV9zdWJzZXQgPC0gZHJvcGxldmVscyhkYXRhX3N1YnNldCkNCmxldmVscyhkYXRhX3N1YnNldCRTdGltKQ0KbGV2ZWxzKGRhdGFfc3Vic2V0JFRhc2spDQpgYGANCmBgYHtyfQ0KbV9zcGVlZF9lZl8zYmFjayA8LSBsbWVyKGZvcm11bGEgPSBTcGVlZF9tIH4gU3RpbSpFRl9tYWcgKyAoMSB8IElEKSwgZGF0YT1kYXRhX3N1YnNldCkNCnN1bW1hcnkobV9zcGVlZF9lZl8zYmFjaykNCmFub3ZhKG1fc3BlZWRfZWZfM2JhY2spDQpgYGANCmBgYHtyfQ0KZGF0YV9zdWJzZXQgPC0gc3Vic2V0KGRmX2VmX211bmksIFN0aW0gPT0gInJlYWxfZnJvbnRhbCIgfCBTdGltID09ICJyZWFsX3BhcmlldGFsIikNCmRhdGFfc3Vic2V0IDwtIGRyb3BsZXZlbHMoZGF0YV9zdWJzZXQpDQpsZXZlbHMoZGF0YV9zdWJzZXQkU3RpbSkNCmBgYA0KYGBge3J9DQptMCA8LSBsbShmb3JtdWxhID0gU3BlZWRfbl9tIH4gVGFzaypTdGltKkVGX21hZywgZGF0YT1kYXRhX3N1YnNldCkNCnN1bW1hcnkobTApDQphbm92YShtMCkNCmBgYA0KYGBge3J9DQptMSA8LSBsbWVyKGZvcm11bGEgPSBTcGVlZF9uX20gfiBUYXNrKlN0aW0qRUZfbWFnICsgKDEgfCBJRCksIGRhdGE9ZGF0YV9zdWJzZXQpDQpzdW1tYXJ5KG0xKQ0KYW5vdmEobTEpDQpgYGANCmBgYHtyfQ0KQUlDKG0wLCBtMSkNCkJJQyhtMCwgbTEpDQpgYGANCmBgYHtyfQ0KbV9zcGVlZF9uX2VmIDwtIGxtKGZvcm11bGEgPSBTcGVlZF9uX20gfiBUYXNrKlN0aW0qRUZfbWFnLCBkYXRhPWRhdGFfc3Vic2V0KQ0Kc3VtbWFyeShtX3NwZWVkX25fZWYpDQphbm92YShtX3NwZWVkX25fZWYpDQpgYGANCmBgYHtyfQ0KZGF0YV9zdWJzZXQgPC0gc3Vic2V0KGRmX2VmX211bmksIChTdGltID09ICJyZWFsX2Zyb250YWwiIHwgU3RpbSA9PSAicmVhbF9wYXJpZXRhbCIpICYgVGFzayA9PSAnMmJhY2snKQ0KZGF0YV9zdWJzZXQgPC0gZHJvcGxldmVscyhkYXRhX3N1YnNldCkNCmxldmVscyhkYXRhX3N1YnNldCRTdGltKQ0KbGV2ZWxzKGRhdGFfc3Vic2V0JFRhc2spDQpgYGANCmBgYHtyfQ0KbV9zcGVlZF9uX2VmXzJiYWNrIDwtIGxtKGZvcm11bGEgPSBTcGVlZF9uX20gfiBTdGltKkVGX21hZywgZGF0YT1kYXRhX3N1YnNldCkNCnN1bW1hcnkobV9zcGVlZF9uX2VmXzJiYWNrKQ0KYW5vdmEobV9zcGVlZF9uX2VmXzJiYWNrKQ0KYGBgDQpgYGB7cn0NCmRhdGFfc3Vic2V0IDwtIHN1YnNldChkZl9lZl9tdW5pLCAoU3RpbSA9PSAicmVhbF9mcm9udGFsIiB8IFN0aW0gPT0gInJlYWxfcGFyaWV0YWwiKSAmIFRhc2sgPT0gJzNiYWNrJykNCmRhdGFfc3Vic2V0IDwtIGRyb3BsZXZlbHMoZGF0YV9zdWJzZXQpDQpsZXZlbHMoZGF0YV9zdWJzZXQkU3RpbSkNCmxldmVscyhkYXRhX3N1YnNldCRUYXNrKQ0KYGBgDQpgYGB7cn0NCm1fc3BlZWRfbl9lZl8zYmFjayA8LSBsbShmb3JtdWxhID0gU3BlZWRfbl9tIH4gU3RpbSpFRl9tYWcsIGRhdGE9ZGF0YV9zdWJzZXQpDQpzdW1tYXJ5KG1fc3BlZWRfbl9lZl8zYmFjaykNCmFub3ZhKG1fc3BlZWRfbl9lZl8zYmFjaykNCmBgYA0KIyMjIEJpZm9jYWwgY29uZGl0aW9ucw0KDQpgYGB7cn0NCmRhdGFfc3Vic2V0IDwtIHN1YnNldChkZl9lZl9tdW5pLCBTdGltID09ICJyZWFsX2luLXBoYXNlIiB8IFN0aW0gPT0gInJlYWxfb3V0LW9mLXBoYXNlIikNCmRhdGFfc3Vic2V0IDwtIGRyb3BsZXZlbHMoZGF0YV9zdWJzZXQpDQpsZXZlbHMoZGF0YV9zdWJzZXQkU3RpbSkNCmBgYA0KV2Ugd2lsbCBub3cgY2hvb3NlIHRoZSBtb2RlbCBmb2xsb3dpbmcgdGhlIHNhbWUgY3JpdGVyaWEgYXMgd2UgYXBwbGllZCB0byB0ZXN0IGFsbCB0aGUgcHJldmlvdXMgYXNwZWN0cy4NCg0KYGBge3J9DQptMCA8LSBsbShmb3JtdWxhID0gQWNjX20gfiBUYXNrKlN0aW0qRUZfZnJvbnQqRUZfcGFyLCBkYXRhPWRhdGFfc3Vic2V0KQ0Kc3VtbWFyeShtMCkNCmFub3ZhKG0wKQ0KYGBgDQpgYGB7cn0NCm0xIDwtIGxtZXIoZm9ybXVsYSA9IEFjY19tIH4gVGFzaypTdGltKkVGX2Zyb250KkVGX3BhciArICgxIHwgSUQpLCBkYXRhPWRhdGFfc3Vic2V0KQ0Kc3VtbWFyeShtMSkNCmFub3ZhKG0xKQ0KYGBgDQpgYGB7cn0NCm1fYWNjX2VmIDwtIGxtKGZvcm11bGEgPSBBY2NfbSB+IFRhc2sqU3RpbSpFRl9mcm9udCpFRl9wYXIsIGRhdGE9ZGF0YV9zdWJzZXQpDQpzdW1tYXJ5KG1fYWNjX2VmKQ0KYW5vdmEobV9hY2NfZWYpDQpldGFfc3F1YXJlZChtX2FjY19lZikNCmBgYA0KYGBge3J9DQpkYXRhX3N1YnNldCA8LSBzdWJzZXQoZGZfZWZfbXVuaSwgKFN0aW0gPT0gInJlYWxfaW4tcGhhc2UiIHwgU3RpbSA9PSAicmVhbF9vdXQtb2YtcGhhc2UiKSAmIFRhc2sgPT0gJzJiYWNrJykNCmRhdGFfc3Vic2V0IDwtIGRyb3BsZXZlbHMoZGF0YV9zdWJzZXQpDQpsZXZlbHMoZGF0YV9zdWJzZXQkU3RpbSkNCmxldmVscyhkYXRhX3N1YnNldCRUYXNrKQ0KYGBgDQpgYGB7cn0NCm1fYWNjX2VmXzJiYWNrIDwtIGxtKGZvcm11bGEgPSBBY2NfbSB+IFN0aW0qRUZfZnJvbnQqRUZfcGFyLCBkYXRhPWRhdGFfc3Vic2V0KQ0Kc3VtbWFyeShtX2FjY19lZl8yYmFjaykNCmFub3ZhKG1fYWNjX2VmXzJiYWNrKQ0KZXRhX3NxdWFyZWQobV9hY2NfZWZfMmJhY2spDQpgYGANCmBgYHtyfQ0KZGF0YV9zdWJzZXQgPC0gc3Vic2V0KGRmX2VmX211bmksIChTdGltID09ICJyZWFsX2luLXBoYXNlIiB8IFN0aW0gPT0gInJlYWxfb3V0LW9mLXBoYXNlIikgJiBUYXNrID09ICczYmFjaycpDQpkYXRhX3N1YnNldCA8LSBkcm9wbGV2ZWxzKGRhdGFfc3Vic2V0KQ0KbGV2ZWxzKGRhdGFfc3Vic2V0JFN0aW0pDQpsZXZlbHMoZGF0YV9zdWJzZXQkVGFzaykNCmBgYA0KYGBge3J9DQptX2FjY19lZl8zYmFjayA8LSBsbShmb3JtdWxhID0gQWNjX20gfiBTdGltKkVGX2Zyb250KkVGX3BhciwgZGF0YT1kYXRhX3N1YnNldCkNCnN1bW1hcnkobV9hY2NfZWZfM2JhY2spDQphbm92YShtX2FjY19lZl8zYmFjaykNCmV0YV9zcXVhcmVkKG1fYWNjX2VmXzNiYWNrKQ0KYGBgDQojIyMgVGhlIHJlbGF0aW9uc2hpcCBiZXR3ZWVuIHRoZSBpbnRlbnNpdHkgb2YgdGhlIGFwcGxpZWQgZWxlY3RyaWMgZmllbGQgb24gdGhlIHRhcmdldCBhbmQgdGhlIG5vcm1hbGl6ZWQgYWNjdXJhY3kNCg0KYGBge3J9DQpkYXRhX3N1YnNldCA8LSBzdWJzZXQoZGZfZWZfbXVuaSwgU3RpbSA9PSAicmVhbF9pbi1waGFzZSIgfCBTdGltID09ICJyZWFsX291dC1vZi1waGFzZSIpDQpkYXRhX3N1YnNldCA8LSBkcm9wbGV2ZWxzKGRhdGFfc3Vic2V0KQ0KbGV2ZWxzKGRhdGFfc3Vic2V0JFN0aW0pDQpgYGANCmBgYHtyfQ0KbTAgPC0gbG0oZm9ybXVsYSA9IEFjY19uX20gfiBUYXNrKlN0aW0qRUZfZnJvbnQqRUZfcGFyLCBkYXRhPWRhdGFfc3Vic2V0KQ0Kc3VtbWFyeShtMCkNCmFub3ZhKG0wKQ0KYGBgDQpgYGB7cn0NCm0xIDwtIGxtZXIoZm9ybXVsYSA9IEFjY19uX20gfiBUYXNrKlN0aW0qRUZfZnJvbnQqRUZfcGFyICsgKDEgfCBJRCksIGRhdGE9ZGF0YV9zdWJzZXQpDQpzdW1tYXJ5KG0xKQ0KYW5vdmEobTEpDQpgYGANCmBgYHtyfQ0KbV9hY2Nfbl9lZiA8LSBsbShmb3JtdWxhID0gQWNjX25fbSB+IFRhc2sqU3RpbSpFRl9mcm9udCpFRl9wYXIsIGRhdGE9ZGF0YV9zdWJzZXQpDQpzdW1tYXJ5KG1fYWNjX25fZWYpDQphbm92YShtX2FjY19uX2VmKQ0KYGBgDQpgYGB7cn0NCmRhdGFfc3Vic2V0IDwtIHN1YnNldChkZl9lZl9tdW5pLCAoU3RpbSA9PSAicmVhbF9pbi1waGFzZSIgfCBTdGltID09ICJyZWFsX291dC1vZi1waGFzZSIpICYgVGFzayA9PSAnMmJhY2snKQ0KZGF0YV9zdWJzZXQgPC0gZHJvcGxldmVscyhkYXRhX3N1YnNldCkNCmxldmVscyhkYXRhX3N1YnNldCRTdGltKQ0KbGV2ZWxzKGRhdGFfc3Vic2V0JFRhc2spDQpgYGANCmBgYHtyfQ0KbV9hY2Nfbl9lZl8yYmFjayA8LSBsbShmb3JtdWxhID0gQWNjX25fbSB+IFN0aW0qRUZfZnJvbnQqRUZfcGFyLCBkYXRhPWRhdGFfc3Vic2V0KQ0Kc3VtbWFyeShtX2FjY19uX2VmXzJiYWNrKQ0KYW5vdmEobV9hY2Nfbl9lZl8yYmFjaykNCmBgYA0KYGBge3J9DQpkYXRhX3N1YnNldCA8LSBzdWJzZXQoZGZfZWZfbXVuaSwgKFN0aW0gPT0gInJlYWxfaW4tcGhhc2UiIHwgU3RpbSA9PSAicmVhbF9vdXQtb2YtcGhhc2UiKSAmIFRhc2sgPT0gJzNiYWNrJykNCmRhdGFfc3Vic2V0IDwtIGRyb3BsZXZlbHMoZGF0YV9zdWJzZXQpDQpsZXZlbHMoZGF0YV9zdWJzZXQkU3RpbSkNCmxldmVscyhkYXRhX3N1YnNldCRUYXNrKQ0KYGBgDQpgYGB7cn0NCm1fYWNjX25fZWZfM2JhY2sgPC0gbG0oZm9ybXVsYSA9IEFjY19uX20gfiBTdGltKkVGX2Zyb250KkVGX3BhciwgZGF0YT1kYXRhX3N1YnNldCkNCnN1bW1hcnkobV9hY2Nfbl9lZl8zYmFjaykNCmFub3ZhKG1fYWNjX25fZWZfM2JhY2spDQpgYGANCiMjIyBUaGUgcmVsYXRpb25zaGlwIGJldHdlZW4gdGhlIGludGVuc2l0eSBvZiB0aGUgYXBwbGllZCBlbGVjdHJpYyBmaWVsZCBvbiB0aGUgdGFyZ2V0IGFuZCB0aGUgc3BlZWQNCg0KYGBge3J9DQpkYXRhX3N1YnNldCA8LSBzdWJzZXQoZGZfZWZfbXVuaSwgU3RpbSA9PSAicmVhbF9pbi1waGFzZSIgfCBTdGltID09ICJyZWFsX291dC1vZi1waGFzZSIpDQpkYXRhX3N1YnNldCA8LSBkcm9wbGV2ZWxzKGRhdGFfc3Vic2V0KQ0KbGV2ZWxzKGRhdGFfc3Vic2V0JFN0aW0pDQpgYGANCmBgYHtyfQ0KbTAgPC0gbG0oZm9ybXVsYSA9IFNwZWVkX20gfiBUYXNrKlN0aW0qRUZfZnJvbnQqRUZfcGFyLCBkYXRhPWRhdGFfc3Vic2V0KQ0Kc3VtbWFyeShtMCkNCmFub3ZhKG0wKQ0KYGBgDQpgYGB7cn0NCm0xIDwtIGxtZXIoZm9ybXVsYSA9IFNwZWVkX20gfiBUYXNrKlN0aW0qRUZfZnJvbnQqRUZfcGFyICsgKDEgfCBJRCksIGRhdGE9ZGF0YV9zdWJzZXQpDQpzdW1tYXJ5KG0xKQ0KYW5vdmEobTEpDQpgYGANCmBgYHtyfQ0KbV9zcGVlZF9lZiA8LSBsbShmb3JtdWxhID0gU3BlZWRfbSB+IFRhc2sqU3RpbSpFRl9mcm9udCpFRl9wYXIsIGRhdGE9ZGF0YV9zdWJzZXQpDQpzdW1tYXJ5KG1fc3BlZWRfZWYpDQphbm92YShtX3NwZWVkX2VmKQ0KZXRhX3NxdWFyZWQobV9zcGVlZF9lZikNCmBgYA0KIyMjIyBFeHBsb3JlIHRoZSB0cmlwbGUgaW50ZXJhY3Rpb25zDQpgYGB7cn0NCm5fcm93cyA9IDEwMA0KY2hvb3NlX21vZGVsIDwtIG1fc3BlZWRfZWYNCnN0aW1fbGlzdCA8LSBsZXZlbHMoZGF0YV9zdWJzZXQkU3RpbSkNCmlfc3RhcnQgPSAwDQppX3N0b3AgPSAwLjMNCg0KY29sdW1ucyA9IGMoIlN0aW0iLCJUYXNrIiwiRUZfZnJvbnQiLCAiRUZfcGFyIikgDQpkZl90ZXN0ID0gZGF0YS5mcmFtZShtYXRyaXgobnJvdyA9IG5fcm93cywgbmNvbCA9IGxlbmd0aChjb2x1bW5zKSkpIA0KY29sbmFtZXMoZGZfdGVzdCkgPSBjb2x1bW5zDQoNCmNvbHVtbnMgPSBjKCJTdGltIiwiVGFzayIsIkVGX2Zyb250IiwgIkVGX3BhciIpIA0KZGZfdGVtcCA9IGRhdGEuZnJhbWUobWF0cml4KG5yb3cgPSBuX3Jvd3MsIG5jb2wgPSBsZW5ndGgoY29sdW1ucykpKSANCmNvbG5hbWVzKGRmX3RlbXApID0gY29sdW1ucw0KDQpmb3IgKHMgaW4gc3RpbV9saXN0KXsNCiAgZm9yICh0IGluIGMoIjJiYWNrIiwgIjNiYWNrIikpew0KICAgIGlmIChzID09IHN0aW1fbGlzdFsxXSAmIHQgPT0gJzJiYWNrJyl7DQogICAgZGZfdGVzdCRTdGltIDwtIHJlcChzLCBuX3Jvd3MpDQogICAgZGZfdGVzdCRFRl9mcm9udCA8LSByZXAoc2VxKGlfc3RhcnQsIGlfc3RvcCwgbGVuZ3RoLm91dCA9IDEwKSwgMTApDQogICAgZGZfdGVzdCRFRl9wYXIgPC0gcmVwKHNlcShpX3N0YXJ0LCBpX3N0b3AsIGxlbmd0aC5vdXQgPSAxMCksIGVhY2g9MTApDQogICAgI2RmX3Rlc3QkVGFzayA8LSBzYW1wbGUoYygnMmJhY2snLCAnM2JhY2snKSwgMTAwLCByZXBsYWNlPVRSVUUpDQogICAgZGZfdGVzdCRUYXNrIDwtIHJlcCh0LCAxMDApDQogICAgfQ0KICAgIGVsc2V7DQogICAgZGZfdGVtcCRTdGltIDwtIHJlcChzLCBuX3Jvd3MpDQogICAgZGZfdGVtcCRFRl9mcm9udCA8LSByZXAoc2VxKGlfc3RhcnQsIGlfc3RvcCwgbGVuZ3RoLm91dCA9IDEwKSwgMTApDQogICAgZGZfdGVtcCRFRl9wYXIgPC0gcmVwKHNlcShpX3N0YXJ0LCBpX3N0b3AsIGxlbmd0aC5vdXQgPSAxMCksIGVhY2g9MTApDQogICAgI2RmX3RlbXAkVGFzayA8LSBzYW1wbGUoYygnMmJhY2snLCAnM2JhY2snKSwgMTAwLCByZXBsYWNlPVRSVUUpDQogICAgZGZfdGVtcCRUYXNrIDwtIHJlcCh0LCAxMDApDQogICAgZGZfdGVzdCA8LSByYmluZChkZl90ZXN0LCBkZl90ZW1wKQ0KICAgIH0NCiAgfQ0KICANCiAgDQogICNkZl9zbGljZSA8LSBzdWJzZXQoZGZfdGVzdCwgU3RpbSA9PSBzKQ0KICAjdmFsX2xpc3QgPC0gYXBwZW5kKHZhbF9saXN0LCBzZXEoZnJvbT0wLCB0bz0wLjMsIGJ5PTAuMy8obnJvdyhkZl9zbGljZSktMSkpKQ0KfQ0KYGBgDQoNCmBgYHtyfQ0KIyBNYWtlIHByZWRpY3Rpb25zDQpwcmVkcyA8LSBwcmVkaWN0KGNob29zZV9tb2RlbCwgZGZfdGVzdCkNCmRmX3Rlc3QkUHJlZCA8LSBwcmVkcw0KYGBgDQpgYGB7cn0NCiMgVmlld2luZyBhbmdsZXMgZm9yIHRoZSBwbG90cw0KYW5nbGVfY29sdW1ucyA9IGMoIlN0aW0iLCJBemltIiwiQ29sYXRpdHVkZSIpIA0KZGZfYW5nbGVzID0gZGF0YS5mcmFtZShtYXRyaXgobnJvdyA9IDQsIG5jb2wgPSBsZW5ndGgoYW5nbGVfY29sdW1ucykpKSANCmNvbG5hbWVzKGRmX2FuZ2xlcykgPSBhbmdsZV9jb2x1bW5zDQoNCmRmX2FuZ2xlcyRTdGltIDwtIHN0aW1fbGlzdA0KZGZfYW5nbGVzJEF6aW0gPC0gYygwLCAwLCAwLCAwKSAjIFRoZXRhLCBkZWZhdWx0IDANCmRmX2FuZ2xlcyRDb2xhdGl0dWRlIDwtIGMoMTUsIDE1LCAxNSwgMTUpICMgUGhpLCBkZWZhdWx0IDE1DQpgYGANCg0KYGBge3J9DQojIE1ha2UgcGxvdHMNCmZvciAocyBpbiBzdGltX2xpc3Qpew0KICBmb3IgKHQgaW4gYygiMmJhY2siLCAiM2JhY2siKSl7DQogICAgZGlzcF9kZiA8LSBzdWJzZXQoZGZfdGVzdCwgU3RpbSA9PSBzICYgVGFzayA9PSB0KQ0KICAgIHBlcnNwKHg9c2VxKGlfc3RhcnQsIGlfc3RvcCwgbGVuZ3RoLm91dCA9IDEwKSwgeT1zZXEoaV9zdGFydCwgaV9zdG9wLCBsZW5ndGgub3V0ID0gMTApLCB6PW1hdHJpeChkaXNwX2RmJFByZWQsIG5yb3c9MTAsIG5jb2w9MTAsYnlyb3c9VFJVRSksIA0KICAgICAgICAgIHRoZXRhID0gc3Vic2V0KGRmX2FuZ2xlcywgU3RpbSA9PSBzKSRBemltLCBwaGkgPSBzdWJzZXQoZGZfYW5nbGVzLCBTdGltID09IHMpJENvbGF0aXR1ZGUsDQogICAgICAgICAgeGxhYj0iRUZfZnJvbnQiLHlsYWI9IkVGX3BhciIsemxhYj0iUHJlZF9TcGVlZF9tIiwgbWFpbj1wYXN0ZShzLHQpLCB6bGltPWMoLTIwMCwzMDApKSANCiAgfQ0KfQ0KYGBgDQpgYGB7cn0NCiMgRXhwb3J0IHRoZSBkYXRhIGZvciBwbG90dGluZw0Kc2F2ZV9maWxlX25hbWUgPC0gZmlsZS5wYXRoKHBhc3RlKGRhdGFfZGlyZWN0b3J5LCAiTVVOSV9FZmllbGRfaW50ZXJhY3Rpb25zLmNzdiIsIHNlcD0nLycpKQ0Kd3JpdGUuY3N2KGRmX3Rlc3QsIHNhdmVfZmlsZV9uYW1lKQ0KYGBgDQoNCg0KIyMjIFN5bW1ldHJ5DQpUbyBmdXJ0aGVyIGV4cGxvcmUgdGhlIGludGVyYWN0aW9uIGJldHdlZW4gdGhlIGluZHVjZWQgZWxlY3RyaWMgZmllbGRzIGFuZCB0aGUgY2hhbmdlcyBpbiBiZWhhdmlvciwgd2Ugd2lsbCBsb29rIGludG8gdGhlIHJlbGF0aXZlIG1hZ25pdHVkZSBhbW9uZyB0aGUgZWxlY3RyaWMgZmllbGRzIGluZHVjZWQgYXQgYm90aCBzaXRlcyAoaS5lLiwgdGhlIHN5bW1ldHJ5KS4gRm9yIHRoaXMgZXhwbG9yYXRpb24sIHdlIHdpbGwgb25seSBsb29rIGF0IHRoZSBiaWZvY2FsIHN0aW11bGF0aW9uIGNvbmRpdGlvbnMuDQpgYGB7cn0NCmRhdGFfc3Vic2V0IDwtIHN1YnNldChkZl9lZl9tdW5pLCBTdGltID09ICdyZWFsX2luLXBoYXNlJyB8IFN0aW0gPT0gJ3JlYWxfb3V0LW9mLXBoYXNlJykNCmRhdGFfc3Vic2V0IDwtIGRyb3BsZXZlbHMoZGF0YV9zdWJzZXQpDQpsZXZlbHMoZGF0YV9zdWJzZXQkU3RpbSkNCmBgYA0KYGBge3J9DQptMCA8LSBsbShmb3JtdWxhID0gU3BlZWRfbSB+IFRhc2sqU3RpbSpFRl9zeW0sIGRhdGE9ZGF0YV9zdWJzZXQpDQpzdW1tYXJ5KG0wKQ0KYW5vdmEobTApDQpgYGANCmBgYHtyfQ0KbTEgPC0gbG1lcihmb3JtdWxhID0gU3BlZWRfbSB+IFRhc2sqU3RpbSpFRl9zeW0gKyAoMSB8IElEKSwgZGF0YT1kYXRhX3N1YnNldCkNCnN1bW1hcnkobTEpDQphbm92YShtMSkNCmBgYA0KYGBge3J9DQpBSUMobTAsIG0xKQ0KQklDKG0wLCBtMSkNCmBgYA0KYGBge3J9DQptX3NwZWVkX2VmX3N5bSA8LSBsbWVyKGZvcm11bGEgPSBTcGVlZF9tIH4gVGFzaypTdGltKkVGX3N5bSArICgxIHwgSUQpLCBkYXRhPWRhdGFfc3Vic2V0KQ0Kc3VtbWFyeShtX3NwZWVkX2VmX3N5bSkNCmFub3ZhKG1fc3BlZWRfZWZfc3ltKQ0KZXRhX3NxdWFyZWQobV9zcGVlZF9lZl9zeW0pDQpgYGANCiMjIyMgQ29tcGFyZSB0aGUgc2xvcGVzDQpgYGB7cn0NCnNsb3BlX2NvbXAgPC0gZW10cmVuZHMobV9zcGVlZF9lZl9zeW0sIHBhaXJ3aXNlIH4gU3RpbSwgdmFyID0gIkVGX3N5bSIpJGNvbnRyYXN0cw0Kc3VtbWFyeShzbG9wZV9jb21wKQ0KYGBgDQojIyMgQ29tYmluZWQgRS1maWVsZCBtYWduaXR1ZGUgKGFsbCBzdGltdWxhdGlvbiBjb25kaXRpb25zKQ0KYGBge3J9DQpkYXRhX3N1YnNldCA8LSBzdWJzZXQoZGZfZWZfbXVuaSwgU3RpbSAhPSAnUGxhY2Vib19sb2MnKQ0KZGF0YV9zdWJzZXQgPC0gZHJvcGxldmVscyhkYXRhX3N1YnNldCkNCmxldmVscyhkYXRhX3N1YnNldCRTdGltKQ0KYGBgDQpgYGB7cn0NCm0wIDwtIGxtKGZvcm11bGEgPSBTcGVlZF9tIH4gVGFzaypTdGltKkVGX21hZywgZGF0YT1kYXRhX3N1YnNldCkNCnN1bW1hcnkobTApDQphbm92YShtMCkNCmBgYA0KYGBge3J9DQptMSA8LSBsbWVyKGZvcm11bGEgPSBTcGVlZF9tIH4gVGFzaypTdGltKkVGX21hZyArICgxIHwgSUQpLCBkYXRhPWRhdGFfc3Vic2V0KQ0Kc3VtbWFyeShtMSkNCmFub3ZhKG0xKQ0KYGBgDQpgYGB7cn0NCkFJQyhtMCwgbTEpDQpCSUMobTAsIG0xKQ0KYGBgDQpgYGB7cn0NCm1fc3BlZWRfZWZfbWFnIDwtIGxtZXIoZm9ybXVsYSA9IFNwZWVkX20gfiBUYXNrKlN0aW0qRUZfbWFnICsgKDEgfCBJRCksIGRhdGE9ZGF0YV9zdWJzZXQpDQpzdW1tYXJ5KG1fc3BlZWRfZWZfbWFnKQ0KYW5vdmEobV9zcGVlZF9lZl9tYWcpDQpldGFfc3F1YXJlZChtX3NwZWVkX2VmX21hZykNCmBgYA0KIyMjIFRoZSByZWxhdGlvbnNoaXAgYmV0d2VlbiB0aGUgaW50ZW5zaXR5IG9mIHRoZSBhcHBsaWVkIGVsZWN0cmljIGZpZWxkIG9uIHRoZSB0YXJnZXQgYW5kIHRoZSBub3JtYWxpemVkIHNwZWVkDQpgYGB7cn0NCmRhdGFfc3Vic2V0IDwtIHN1YnNldChkZl9lZl9tdW5pLCBTdGltICE9ICdQbGFjZWJvX2xvYycpDQpkYXRhX3N1YnNldCA8LSBkcm9wbGV2ZWxzKGRhdGFfc3Vic2V0KQ0KbGV2ZWxzKGRhdGFfc3Vic2V0JFN0aW0pDQpgYGANCmBgYHtyfQ0KbTAgPC0gbG0oZm9ybXVsYSA9IFNwZWVkX25fbSB+IFRhc2sqU3RpbSpFRl9mcm9udCpFRl9wYXIsIGRhdGE9ZGF0YV9zdWJzZXQpDQpzdW1tYXJ5KG0wKQ0KYW5vdmEobTApDQpgYGANCmBgYHtyfQ0KbTEgPC0gbG1lcihmb3JtdWxhID0gU3BlZWRfbl9tIH4gVGFzaypTdGltKkVGX2Zyb250KkVGX3BhciArICgxIHwgSUQpLCBkYXRhPWRhdGFfc3Vic2V0KQ0Kc3VtbWFyeShtMSkNCmFub3ZhKG0xKQ0KYGBgDQpgYGB7cn0NCm1fc3BlZWRfbl9lZiA8LSBsbShmb3JtdWxhID0gU3BlZWRfbl9tIH4gVGFzaypTdGltKkVGX2Zyb250KkVGX3BhciwgZGF0YT1kYXRhX3N1YnNldCkNCnN1bW1hcnkobV9zcGVlZF9uX2VmKQ0KYW5vdmEobV9zcGVlZF9uX2VmKQ0KYGBgDQojIyBSZWxhdGlvbnNoaXAgYmV0d2VlbiBiZWhhdmlvciBhbmQgdGhlIHBoYXNlLWFtcGxpdHVkZSBjb3VwbGluZyBpbmRleCAoUEFDKQ0KDQpBcyBwYXJ0IG9mIG91ciBhbmFseXNpcywgd2UgZXh0cmFjdGVkIHRoZSBQQUMgYXQgYm90aCBzdGltdWxhdGlvbiBzaXRlcyBmb3IgZWFjaCBzdWJqZWN0LCBhcyB3YXMgZG9uZSBieSBSZWluaGFydCBhbmQgTmd1eWVuICgyMDE5KS4gSW4gdGhpcyBwYXBlciwgdGhlIGF1dGhvcnMgbG9va2VkIGF0IHRoZSBQQUMgYXQgdGhlICJtZW1vcnkgUk9JIiAobGVmdCBjZW50cm8tdGVtcG9yYWwgY2x1c3RlciksIGFuZCB0aGV5IGZvdW5kIGhpZ2gtcGVyZm9ybWluZyB5b3VuZyBhZHVsdHMgdG8gaGF2ZSBoaWdoIFBBQyB2YWx1ZXMgaW4gdGhpcyByZWdpb24uIEluIGNvbnRyYXN0LCBvbGRlciBhZHVsdHMgaGFkIGxvdyBQQUMgdmFsdWVzIGluIHRoYXQgcmVnaW9uLCBhcyB3ZWxsIGFzIGxvdyBwZXJmb3JtYW5jZSBpbiB0aGUgV00gdGFzay4gSG93ZXZlciwgd2hlbiBzdGltdWxhdGlvbiB3YXMgYXBwbGllZCwgcGVyZm9ybWFuY2UgaW1wcm92ZWQgc2hvcnRseSBhZnRlciBzdGFydGluZyB0aGUgc3RpbXVsYXRpb24sIGFuZCB0aGUgUEFDIHdhcyBhbHNvIGhpZ2ggaW4gb2xkZXIgYWR1bHRzLiBTbywgdGhlIG9iamVjdGl2ZSBvZiB0aGlzIGFuYWx5c2lzIGlzIHRvIHNlZSB3aGV0aGVyIHRoZXJlIGFyZSBtZWFuaW5nZnVsIGRpZmZlcmVuY2VzIGluIFBBQyBjb3JyZWxhdGluZyB3aXRoIHBlcmZvcm1hbmNlLiANCg0KVGhlIFBBQyBmZWF0dXJlcyB3ZSBhcmUgdXNpbmcgYXJlIGEgY29tcGFyaXNvbiBiZXR3ZWVuIHRoZSAyLWJhY2sgYW5kIHRoZSAzLWJhY2sgdGFza3MgKHN1YnRyYWN0aW9uKSwgYW5kIHRoZXkgYXJlIHRoZSBmb2xsb3dpbmc6DQotIE1lbW9yeSBST0kgKExpa2UgaW4gUmVpbmhhcnQgYW5kIE5ndXllbikNCi0gRnJvbnRhbCBzdGltdWxhdGlvbiBST0kNCi0gUGFyaWV0YWwgc3RpbXVsYXRpb24gUk9JDQoNClRoZSBjb21wYXJpc29uIGJldHdlZW4gdGhlIDItYmFjayBhbmQgdGhlIDMtYmFjayB0YXNrIHdhcyBkb25lIHRocm91Z2ggYSBzdWJ0cmFjdGlvbiBpbiB0aGUgUEFDIHZhbHVlcy4gVGhlIHJhdGlvbmFsZSBpcyB0aGF0IHRoaXMgZGlmZmVyZW5jZSBpc29sYXRlcyB0aGUgbmV1cmFsIGFjdGl2aXR5IHJlbGF0ZWQgdG8gcmV0YWluaW5nIGluZm9ybWF0aW9uIGluIHdvcmtpbmcgbWVtb3J5IGZvciBsb25nZXIsIHdoaWNoIGlzIGFzc3VtZWQgdG8gYmUgbW9yZSBkaWZmaWN1bHQgaW4gdGhlIDMtYmFjayBjb21wYXJlZCB0byB0aGUgMi1iYWNrIHRhc2sgYW5kIGlzIHN1cHBvcnRlZCBieSB0aGUgYmVoYXZpb3JhbCBkaWZmZXJlbmNlcyBzZWVuIGluIGJvdGggdGhlIHNwZWVkIGFuZCB0aGUgYWNjdXJhY3kgd2hlbiBjb21wYXJpbmcgcGVyZm9ybWFuY2UgaW4gYm90aCB0YXNrcy4gVGhlcmVmb3JlLCB0aGUgcmVzdWx0aW5nIGRlbHRhIGluIFBBQyBhbW9uZyBib3RoIHRhc2tzIHdvdWxkIGJlIHJlbGF0ZWQgdG8gYW4gaW5kaXZpZHVhbCdzIGFiaWxpdHkgdG8gcGVyZm9ybSB0aGUgKGhhcmRlcikgMy1iYWNrIHRhc2suDQoNCmBgYHtyfQ0KZGF0YV9kaXJlY3RvcnkgPC0gZmlsZS5wYXRoKHBhc3RlKGN1cnJfZGlyLCAnLi4vLi4vLi4vQ29kZS9Ob3RlYm9va3MvUmVzdWx0cycsIHNlcD0nLycpKQ0KDQpmaWxlX25hbWUgPC0gZmlsZS5wYXRoKHBhc3RlKGRhdGFfZGlyZWN0b3J5LCAiQmVoYXZpb3JhbF9hbmRfUEFDX2Jsb2Nrc19pQ09HX0Rlc2lnbl8zMF8wNl8yMDIzLnR4dCIsIHNlcD0nLycpKQ0KZGZfcGFjIDwtIHJlYWQuZGVsaW0oZmlsZV9uYW1lLCBoZWFkZXIgPSBUUlVFLCBuYS5zdHJpbmdzID0gIk5OIikNCmhlYWQoZGZfcGFjLCA1KQ0KYGBgDQpgYGB7cn0NCmRmX3BhYyRJRCA8LSBhcy5mYWN0b3IoZGZfcGFjJElEKQ0KZGZfcGFjJFNjaG9vbCA8LSBhcy5mYWN0b3IoZGZfcGFjJFNjaG9vbCkNCmRmX3BhYyREYXkgPC0gYXMuZmFjdG9yKGRmX3BhYyREYXkpDQpkZl9wYWMkVGFzayA8LSBhcy5mYWN0b3IoZGZfcGFjJFRhc2spDQpkZl9wYWMkU3RpbSA8LSBhcy5mYWN0b3IoZGZfcGFjJFN0aW0pDQpkZl9wYWMkQWNjX3UgPC0gYXMubnVtZXJpYyhkZl9wYWMkQWNjX3UpDQpkZl9wYWMkQWNjX25fdSA8LSBhcy5udW1lcmljKGRmX3BhYyRBY2Nfbl91KQ0KZGZfcGFjJFNwZWVkX3UgPC0gYXMubnVtZXJpYyhkZl9wYWMkU3BlZWRfdSkNCmRmX3BhYyRTcGVlZF9uX3UgPC0gYXMubnVtZXJpYyhkZl9wYWMkU3BlZWRfbl91KQ0KZGZfcGFjJEFjY19tIDwtIGFzLm51bWVyaWMoZGZfcGFjJEFjY19tKQ0KZGZfcGFjJEFjY19uX20gPC0gYXMubnVtZXJpYyhkZl9wYWMkQWNjX25fbSkNCmRmX3BhYyRTcGVlZF9tIDwtIGFzLm51bWVyaWMoZGZfcGFjJFNwZWVkX20pDQpkZl9wYWMkU3BlZWRfbl9tIDwtIGFzLm51bWVyaWMoZGZfcGFjJFNwZWVkX25fbSkNCmRmX3BhYyRQQUNfbWVtIDwtIGFzLm51bWVyaWMoZGZfcGFjJFBBQ19tZW0pDQpkZl9wYWMkUEFDX2Zyb250IDwtIGFzLm51bWVyaWMoZGZfcGFjJFBBQ19mcm9udCkNCmRmX3BhYyRQQUNfcGFyIDwtIGFzLm51bWVyaWMoZGZfcGFjJFBBQ19wYXIpDQpgYGANCg0KYGBge3J9DQpkZl9wYWNfbXVuaSA8LSBzdWJzZXQoZGZfcGFjLCBTY2hvb2wgPT0gJ01VTkknKQ0KZGZfcGFjX211bmkgPC0gZHJvcGxldmVscyhkZl9wYWNfbXVuaSkNCmRmX3BhY19tdW5pJFN0aW0gPC0gZmFjdG9yKGRmX3BhY19tdW5pJFN0aW0sIGxldmVscyhkZl9wYWNfbXVuaSRTdGltKVtjKDEsIDIsIDUsIDMsIDQpXSkNCmxldmVscyhkZl9wYWNfbXVuaSRTdGltKQ0KYGBgDQojIyMgUmVsYXRpb25zaGlwIGJldHdlZW4gUEFDIGFuZCBwZXJmb3JtYW5jZQ0KQmFzZWQgb24gUmVpbmhhcnQncyBhbmQgTmd1eWVuJ3MgZmluZGluZ3MsIHBlb3BsZSB3aXRoIGhpZ2hlciBQQUMgdmFsdWVzIGF0IHRoZSAiTWVtb3J5IFJPSSIgd291bGQgYmUgZXhwZWN0ZWQgdG8gaGF2ZSBhIGJldHRlciBwZXJmb3JtYW5jZSBpbiB0aGUgdGFzay4gVGhlcmVmb3JlLCB3ZSB3aWxsIGZpcnN0IHRlc3QgdGhlIHJlbGF0aW9uc2hpcCBiZXR3ZWVuIHRoZSBQQUMgYW5kIHRoZSBwZXJmb3JtYW5jZSB1bmRlciB0aGUgcGxhY2VibyBjb25kaXRpb24uIEFzIHRoZSBQQUMgdmFsdWVzIHdlIGFyZSB1c2luZyBjb25zaXN0IG9uIHRoZSBkaWZmZXJlbmNlIG9mIHZhbHVlcyBiZXR3ZWVuIHRoZSAyYmFjayBhbmQgM2JhY2sgY29uZGl0aW9ucywgd2hpY2ggaXMgbWVhbnQgdG8gcmVmbGVjdCB0aGUgY291cGxpbmcgcmVsYXRlZCB0byBoaWdoZXIgd29ya2luZyBtZW1vcnkgZGVtYW5kcywgd2Ugd2lsbCB1c2UgdGhlIGJlaGF2aW9yYWwgc2NvcmVzIG9mIHRoZSAzYmFjayB0YXNrLg0KIyMjIyBBY2N1cmFjeSBhbmQgbWVtb3J5IFJPSQ0KYGBge3J9DQpkYXRhX3N1YnNldCA8LSBzdWJzZXQoZGZfcGFjX211bmksIFN0aW0gPT0gIlBsYWNlYm9fbG9jIiAmIFRhc2sgPT0gIjNiYWNrIikNCmRhdGFfc3Vic2V0IDwtIGRyb3BsZXZlbHMoZGF0YV9zdWJzZXQpDQpsZXZlbHMoZGF0YV9zdWJzZXQkU3RpbSkNCmxldmVscyhkYXRhX3N1YnNldCRUYXNrKQ0KYGBgDQojIyMjIEF2ZXJhZ2UgYWNjdXJhY3kNCmBgYHtyfQ0KbV9wYWNfbWVtX2FjY191X3BsYWNlYm8gPC0gbG0oZm9ybXVsYSA9IEFjY191IH4gUEFDX21lbSwgZGF0YT1kYXRhX3N1YnNldCkNCnN1bW1hcnkobV9wYWNfbWVtX2FjY191X3BsYWNlYm8pDQphbm92YShtX3BhY19tZW1fYWNjX3VfcGxhY2VibykNCmV0YV9zcXVhcmVkKG1fcGFjX21lbV9hY2NfdV9wbGFjZWJvKQ0KYGBgDQojIyMjIEFjY3VyYWN5IGFuZCBQQUMgYXQgMTAvMjAgRUVHIGVsZWN0cm9kZS1iYXNlZCB0YXJnZXRzDQpgYGB7cn0NCm1fcGFjX3RhcmdldHNfYWNjX3VfcGxhY2VibyA8LSBsbShmb3JtdWxhID0gQWNjX3UgfiBQQUNfZnJvbnQqUEFDX3BhciwgZGF0YT1kYXRhX3N1YnNldCkNCnN1bW1hcnkobV9wYWNfdGFyZ2V0c19hY2NfdV9wbGFjZWJvKQ0KYW5vdmEobV9wYWNfdGFyZ2V0c19hY2NfdV9wbGFjZWJvKQ0KZXRhX3NxdWFyZWQobV9wYWNfdGFyZ2V0c19hY2NfdV9wbGFjZWJvKQ0KYGBgDQojIyMjIENoZWNrIGZvciBhIHJlbGF0aW9uc2hpcCBiZXR3ZWVuIFBBQyBpbiB0aGUgcGxhY2VibyBjb25kaXRpb24gYW5kIHBlcmZvcm1hbmNlIHVuZGVyIHN0aW11bGF0aW9uDQpgYGB7cn0NCmRhdGFfc3Vic2V0IDwtIHN1YnNldChkZl9wYWNfbXVuaSwgU3RpbSAhPSAiUGxhY2Vib19sb2MiICYgVGFzayA9PSAiM2JhY2siKQ0KZGF0YV9zdWJzZXQgPC0gZHJvcGxldmVscyhkYXRhX3N1YnNldCkNCmxldmVscyhkYXRhX3N1YnNldCRTdGltKQ0KbGV2ZWxzKGRhdGFfc3Vic2V0JFRhc2spDQpgYGANCmBgYHtyfQ0KbV9wYWNfbWVtX2FjY191X3N0aW0gPC0gbG0oZm9ybXVsYSA9IEFjY191IH4gU3RpbSpQQUNfbWVtLCBkYXRhPWRhdGFfc3Vic2V0KQ0Kc3VtbWFyeShtX3BhY19tZW1fYWNjX3Vfc3RpbSkNCmFub3ZhKG1fcGFjX21lbV9hY2NfdV9zdGltKQ0KYGBgDQpgYGB7cn0NCm1fcGFjX3RhcmdldHNfYWNjX3Vfc3RpbXMgPC0gbG0oZm9ybXVsYSA9IEFjY191IH4gU3RpbSpQQUNfZnJvbnQqUEFDX3BhciwgZGF0YT1kYXRhX3N1YnNldCkNCnN1bW1hcnkobV9wYWNfdGFyZ2V0c19hY2NfdV9zdGltcykNCmFub3ZhKG1fcGFjX3RhcmdldHNfYWNjX3Vfc3RpbXMpDQpldGFfc3F1YXJlZChtX3BhY190YXJnZXRzX2FjY191X3N0aW1zKQ0KYGBgDQojIyMjIE5vcm1hbGl6ZWQgYWNjdXJhY3kNCmBgYHtyfQ0KZGF0YV9zdWJzZXQgPC0gc3Vic2V0KGRmX3BhY19tdW5pLCBTdGltID09ICJQbGFjZWJvX2xvYyIgJiBUYXNrID09ICIzYmFjayIpDQpkYXRhX3N1YnNldCA8LSBkcm9wbGV2ZWxzKGRhdGFfc3Vic2V0KQ0KbGV2ZWxzKGRhdGFfc3Vic2V0JFN0aW0pDQpsZXZlbHMoZGF0YV9zdWJzZXQkVGFzaykNCmBgYA0KYGBge3J9DQptX3BhY19tZW1fYWNjX25fdV9wbGFjZWJvIDwtIGxtKGZvcm11bGEgPSBBY2Nfbl91IH4gUEFDX21lbSwgZGF0YT1kYXRhX3N1YnNldCkNCnN1bW1hcnkobV9wYWNfbWVtX2FjY19uX3VfcGxhY2VibykNCmFub3ZhKG1fcGFjX21lbV9hY2Nfbl91X3BsYWNlYm8pDQpgYGANCmBgYHtyfQ0KbV9wYWNfdGFyZ2V0c19hY2Nfbl91X3BsYWNlYm8gPC0gbG0oZm9ybXVsYSA9IEFjY19uX3UgfiBQQUNfZnJvbnQqUEFDX3BhciwgZGF0YT1kYXRhX3N1YnNldCkNCnN1bW1hcnkobV9wYWNfdGFyZ2V0c19hY2Nfbl91X3BsYWNlYm8pDQphbm92YShtX3BhY190YXJnZXRzX2FjY19uX3VfcGxhY2VibykNCmBgYA0KIyMjIyBTdGltdWxhdGlvbnMNCmBgYHtyfQ0KZGF0YV9zdWJzZXQgPC0gc3Vic2V0KGRmX3BhY19tdW5pLCBTdGltICE9ICJQbGFjZWJvX2xvYyIgJiBUYXNrID09ICIzYmFjayIpDQpkYXRhX3N1YnNldCA8LSBkcm9wbGV2ZWxzKGRhdGFfc3Vic2V0KQ0KbGV2ZWxzKGRhdGFfc3Vic2V0JFN0aW0pDQpsZXZlbHMoZGF0YV9zdWJzZXQkVGFzaykNCmBgYA0KYGBge3J9DQptX3BhY19tZW1fYWNjX25fdV9zdGltIDwtIGxtKGZvcm11bGEgPSBBY2Nfbl91IH4gU3RpbSpQQUNfbWVtLCBkYXRhPWRhdGFfc3Vic2V0KQ0Kc3VtbWFyeShtX3BhY19tZW1fYWNjX25fdV9zdGltKQ0KYW5vdmEobV9wYWNfbWVtX2FjY19uX3Vfc3RpbSkNCmBgYA0KYGBge3J9DQptX3BhY190YXJnZXRzX2FjY19uX3Vfc3RpbSA8LSBsbShmb3JtdWxhID0gQWNjX25fdSB+IFN0aW0qUEFDX2Zyb250KlBBQ19wYXIsIGRhdGE9ZGF0YV9zdWJzZXQpDQpzdW1tYXJ5KG1fcGFjX3RhcmdldHNfYWNjX25fdV9zdGltKQ0KYW5vdmEobV9wYWNfdGFyZ2V0c19hY2Nfbl91X3N0aW0pDQpldGFfc3F1YXJlZChtX3BhY190YXJnZXRzX2FjY19uX3Vfc3RpbSkNCmBgYA0KIyMjIyBDb21wYXJlIHRoZSBzbG9wZXMgcmVzdWx0aW5nIGZyb20gdGhlIGludGVyYWN0aW9uIGJldHdlZW4gdGhlIHN0aW11bGF0aW9uIGFuZCB0aGUgZGlmZmVyZW5jZSBpbiBQQUMgc2NvcmVzDQpgYGB7cn0NCnNsb3BlX2NvbXAgPC0gZW10cmVuZHMobV9wYWNfdGFyZ2V0c19hY2Nfbl91X3N0aW0sIHBhaXJ3aXNlIH4gU3RpbSwgdmFyID0gIlBBQ19wYXIiKSRjb250cmFzdHMNCnN1bW1hcnkoc2xvcGVfY29tcCkNCmBgYA0KDQoNCiMjIyBQQUMgYW5kIHNwZWVkDQojIyMjIFBsYWNlYm8NCmBgYHtyfQ0KZGF0YV9zdWJzZXQgPC0gc3Vic2V0KGRmX3BhY19tdW5pLCBTdGltID09ICJQbGFjZWJvX2xvYyIgJiBUYXNrID09ICIzYmFjayIpDQpkYXRhX3N1YnNldCA8LSBkcm9wbGV2ZWxzKGRhdGFfc3Vic2V0KQ0KbGV2ZWxzKGRhdGFfc3Vic2V0JFN0aW0pDQpsZXZlbHMoZGF0YV9zdWJzZXQkVGFzaykNCmBgYA0KIyMjIyBNZW1vcnkgUk9JDQpgYGB7cn0NCm1fcGFjX21lbV9zcGVlZF91X3BsYWNlYm8gPC0gbG0oZm9ybXVsYSA9IFNwZWVkX3UgfiBQQUNfbWVtLCBkYXRhPWRhdGFfc3Vic2V0KQ0Kc3VtbWFyeShtX3BhY19tZW1fc3BlZWRfdV9wbGFjZWJvKQ0KYW5vdmEobV9wYWNfbWVtX3NwZWVkX3VfcGxhY2VibykNCmBgYA0KIyMjIyBGcm9udGFsIGFuZCBwYXJpZXRhbCB0YXJnZXRzDQpgYGB7cn0NCm1fcGFjX3RhcmdldHNfc3BlZWRfdV9wbGFjZWJvIDwtIGxtKGZvcm11bGEgPSBTcGVlZF91IH4gUEFDX2Zyb250KlBBQ19wYXIsIGRhdGE9ZGF0YV9zdWJzZXQpDQpzdW1tYXJ5KG1fcGFjX3RhcmdldHNfc3BlZWRfdV9wbGFjZWJvKQ0KYW5vdmEobV9wYWNfdGFyZ2V0c19zcGVlZF91X3BsYWNlYm8pDQpldGFfc3F1YXJlZChtX3BhY190YXJnZXRzX3NwZWVkX3VfcGxhY2VibykNCmBgYA0KIyMjIyBTdGltdWxhdGlvbiBncm91cHMNCmBgYHtyfQ0KZGF0YV9zdWJzZXQgPC0gc3Vic2V0KGRmX3BhY19tdW5pLCBTdGltICE9ICJQbGFjZWJvX2xvYyIgJiBUYXNrID09ICIzYmFjayIpDQpkYXRhX3N1YnNldCA8LSBkcm9wbGV2ZWxzKGRhdGFfc3Vic2V0KQ0KbGV2ZWxzKGRhdGFfc3Vic2V0JFN0aW0pDQpsZXZlbHMoZGF0YV9zdWJzZXQkVGFzaykNCmBgYA0KIyMjIyBNZW1vcnkgUk9JDQpgYGB7cn0NCm1fcGFjX21lbV9zcGVlZF91X3N0aW0gPC0gbG0oZm9ybXVsYSA9IFNwZWVkX3UgfiBTdGltKlBBQ19tZW0sIGRhdGE9ZGF0YV9zdWJzZXQpDQpzdW1tYXJ5KG1fcGFjX21lbV9zcGVlZF91X3N0aW0pDQphbm92YShtX3BhY19tZW1fc3BlZWRfdV9zdGltKQ0KYGBgDQojIyMjIEZyb250YWwgYW5kIHBhcmlldGFsIHRhcmdldHMNCmBgYHtyfQ0KbV9wYWNfdGFyZ2V0c19zcGVlZF91X3N0aW0gPC0gbG0oZm9ybXVsYSA9IFNwZWVkX3UgfiBTdGltKlBBQ19mcm9udCpQQUNfcGFyLCBkYXRhPWRhdGFfc3Vic2V0KQ0Kc3VtbWFyeShtX3BhY190YXJnZXRzX3NwZWVkX3Vfc3RpbSkNCmFub3ZhKG1fcGFjX3RhcmdldHNfc3BlZWRfdV9zdGltKQ0KYGBgDQojIyMjIE5vcm1hbGl6ZWQgc3BlZWQNCmBgYHtyfQ0KZGF0YV9zdWJzZXQgPC0gc3Vic2V0KGRmX3BhY19tdW5pLCBTdGltID09ICJQbGFjZWJvX2xvYyIgJiBUYXNrID09ICIzYmFjayIpDQpkYXRhX3N1YnNldCA8LSBkcm9wbGV2ZWxzKGRhdGFfc3Vic2V0KQ0KbGV2ZWxzKGRhdGFfc3Vic2V0JFN0aW0pDQpsZXZlbHMoZGF0YV9zdWJzZXQkVGFzaykNCmBgYA0KYGBge3J9DQptX3BhY19tZW1fc3BlZWRfbl91X3BsYWNlYm8gPC0gbG0oZm9ybXVsYSA9IFNwZWVkX25fdSB+IFBBQ19tZW0sIGRhdGE9ZGF0YV9zdWJzZXQpDQpzdW1tYXJ5KG1fcGFjX21lbV9zcGVlZF9uX3VfcGxhY2VibykNCmFub3ZhKG1fcGFjX21lbV9zcGVlZF9uX3VfcGxhY2VibykNCmBgYA0KYGBge3J9DQptX3BhY190YXJnZXRzX3NwZWVkX25fdV9wbGFjZWJvIDwtIGxtKGZvcm11bGEgPSBTcGVlZF9uX3UgfiBQQUNfZnJvbnQqUEFDX3BhciwgZGF0YT1kYXRhX3N1YnNldCkNCnN1bW1hcnkobV9wYWNfdGFyZ2V0c19zcGVlZF9uX3VfcGxhY2VibykNCmFub3ZhKG1fcGFjX3RhcmdldHNfc3BlZWRfbl91X3BsYWNlYm8pDQpgYGANCiMjIyMgU3RpbXVsYXRpb25zDQpgYGB7cn0NCmRhdGFfc3Vic2V0IDwtIHN1YnNldChkZl9wYWNfbXVuaSwgU3RpbSAhPSAiUGxhY2Vib19sb2MiICYgVGFzayA9PSAiM2JhY2siKQ0KZGF0YV9zdWJzZXQgPC0gZHJvcGxldmVscyhkYXRhX3N1YnNldCkNCmxldmVscyhkYXRhX3N1YnNldCRTdGltKQ0KbGV2ZWxzKGRhdGFfc3Vic2V0JFRhc2spDQpgYGANCmBgYHtyfQ0KbV9wYWNfbWVtX3NwZWVkX25fdV9zdGltIDwtIGxtKGZvcm11bGEgPSBTcGVlZF9uX3UgfiBTdGltKlBBQ19tZW0sIGRhdGE9ZGF0YV9zdWJzZXQpDQpzdW1tYXJ5KG1fcGFjX21lbV9zcGVlZF9uX3Vfc3RpbSkNCmFub3ZhKG1fcGFjX21lbV9zcGVlZF9uX3Vfc3RpbSkNCmBgYA0KYGBge3J9DQptX3BhY190YXJnZXRzX3NwZWVkX25fdV9zdGltIDwtIGxtKGZvcm11bGEgPSBTcGVlZF9uX3UgfiBTdGltKlBBQ19mcm9udCpQQUNfcGFyLCBkYXRhPWRhdGFfc3Vic2V0KQ0Kc3VtbWFyeShtX3BhY190YXJnZXRzX3NwZWVkX25fdV9zdGltKQ0KYW5vdmEobV9wYWNfdGFyZ2V0c19zcGVlZF9uX3Vfc3RpbSkNCmBgYA0KDQoNCg0KDQoNCg0KDQoNCg0KDQoNCg0KDQoNCg0KDQoNCg0KDQoNCg0K
