# Supplementary material for: Physiology-inspired bifocal fronto-parietal tACS for working memory enhancement
Source: Heliyon. 2024 Sep 6;10(18):e37427. doi: 10.1016/j.heliyon.2024.e37427 (PMC11417162; doi:10.1016/j.heliyon.2024.e37427)
Supplement: Multimedia component 4 [file mmc4.zip › Supplementary materials_Reports on statistics_fMRI.html]

R\_Notebook\_for\_iCOG\_fMRI\_analysis


Code 

- Show All Code
- Hide All Code
- Download Rmd

# R\_Notebook\_for\_iCOG\_fMRI\_analysis


```
library(Matrix)
library(lme4)
library(carData)
library(car)
library(lmerTest)
library(emmeans)
library(effectsize)
library(ggplot2)
library(Rmisc)
library(nlme)
library(reshape)
```


```
setwd("~/iCOG/WP1/data/rs-fMRI")
iCOG_Clear<-read.csv2("iCOG_fMRI_FPCN_differences.csv")
names(iCOG_Clear)[names(iCOG_Clear)=="ď.żID_subject"]<-"ID_subject"
head(iCOG_Clear,5)
```


```
iCOG_Clear$Stim_protocol=as.factor(iCOG_Clear$Stim_protocol)
iCOG_Clear$Stim_protocol<-factor(iCOG_Clear$Stim_protocol, labels = c("Sham", "Frontal", "Parietal", "In-phase", "Out-of-phase"))
levels(iCOG_Clear$Stim_protocol)
```


```
[1] "Sham"         "Frontal"      "Parietal"     "In-phase"     "Out-of-phase"
```


**Connectivity within FPCN network statistics**

Simple model, averages

Here, we present different models to characterize each relevant
parameter related to the participants’ connectivity withing FPCN
network. In each case, the models start off from a simple form (i.e.,
group-averaged differencies after-before the stimulation per session),
and they grow in complexity as we add other random effects to account
for the variability across subjects. Each model is compared to its
(simpler) predecessor to assess whether the eventual improvement in data
explainability, as quantified by the conventional criteria (i.e.,
Akaike’s information criteria), justifies the use of a more complex
model.

Model 0 including all stimulation protocols to see which one would be
different from placebo stimulation


```
vars = names(iCOG_Clear[42:length(names(iCOG_Clear))])

models0_diff = lapply(setNames(vars,vars), function(var) {
  form = paste(var ," ~ Stim_protocol")
  lm(form, data=iCOG_Clear)
})
anova_models0_diff<-sapply(models0_diff,anova)
anova_models0_p_diff<-anova_models0_diff[5,]
p_LMM_table_models0_diff<-data.frame(t(sapply(anova_models0_p_diff,c)))
p_LMM_table_models0_diff
```


```
AIC_models0_diff<-sapply(models0_diff,AIC)
```


Model 1 - random effect of ID\_Subject, including all
stimulation protocols to see which one would be different from placebo
stimulation. P-values of the


```
models1_diff = lapply(setNames(vars,vars), function(var) {
  form = paste(var ," ~ Stim_protocol+(1|ID_subject)")
  lmer(form, data=iCOG_Clear)
})
```


```
boundary (singular) fit: see help('isSingular')
boundary (singular) fit: see help('isSingular')
boundary (singular) fit: see help('isSingular')
boundary (singular) fit: see help('isSingular')
boundary (singular) fit: see help('isSingular')
boundary (singular) fit: see help('isSingular')
boundary (singular) fit: see help('isSingular')
boundary (singular) fit: see help('isSingular')
boundary (singular) fit: see help('isSingular')
boundary (singular) fit: see help('isSingular')
```


```
anova_models1_diff<-sapply(models1_diff,anova)
anova_models1_p_diff<-anova_models1_diff[6,]
p_LMM_table_models1_diff<-data.frame(t(lapply(anova_models1_p_diff,c)))
p_LMM_table_models1_diff
```


```
AIC_models1_diff<-sapply(models1_diff,AIC)
```


When using this more complex model, a problem with singularity
evolves for all the connections. We will therefore end the process of
model selection in this step, choosing the model 0 as our final
model.

We will now do the post-hoc tests for the significant connectivity
seed pairs (raPFClaIPL,raPFCrdlPFC ), to find out where these
differences come from.


```
sig_vars= c("raPFClaIPL","raPFCrdlPFC")

sig_models0_diff = lapply(setNames(sig_vars,sig_vars), function(var) {
  form = paste(var ," ~ Stim_protocol")
  lm(form, data=iCOG_Clear)
})

emm_stim_protocol_sig_model0_diff <- sapply(sig_models0_diff, emmeans, ~Stim_protocol)
P_pairwaise_comparison__stim_protocol_signif_model0_diff<-sapply(emm_stim_protocol_sig_model0_diff, pairs ,adjust = "none")
P_pairwaise_comparison__stim_protocol_signif_model0_diff
```


```
$raPFClaIPL
 contrast                    estimate     SE df t.ratio p.value
 Sham - Frontal                0.0520 0.0648 70   0.803  0.4246
 Sham - Parietal               0.0915 0.0637 70   1.437  0.1553
 Sham - (In-phase)            -0.0926 0.0627 70  -1.476  0.1444
 Sham - (Out-of-phase)         0.0272 0.0627 70   0.433  0.6661
 Frontal - Parietal            0.0395 0.0637 70   0.620  0.5376
 Frontal - (In-phase)         -0.1446 0.0627 70  -2.306  0.0241
 Frontal - (Out-of-phase)     -0.0249 0.0627 70  -0.396  0.6931
 Parietal - (In-phase)        -0.1841 0.0616 70  -2.988  0.0039
 Parietal - (Out-of-phase)    -0.0643 0.0616 70  -1.044  0.3000
 (In-phase) - (Out-of-phase)   0.1198 0.0606 70   1.976  0.0520


$raPFCrdlPFC
 contrast                    estimate     SE df t.ratio p.value
 Sham - Frontal                0.1460 0.0626 70   2.332  0.0226
 Sham - Parietal               0.0543 0.0616 70   0.882  0.3810
 Sham - (In-phase)            -0.0420 0.0606 70  -0.693  0.4907
 Sham - (Out-of-phase)         0.0235 0.0606 70   0.388  0.6991
 Frontal - Parietal           -0.0917 0.0616 70  -1.490  0.1407
 Frontal - (In-phase)         -0.1880 0.0606 70  -3.101  0.0028
 Frontal - (Out-of-phase)     -0.1225 0.0606 70  -2.020  0.0472
 Parietal - (In-phase)        -0.0963 0.0595 70  -1.617  0.1104
 Parietal - (Out-of-phase)    -0.0307 0.0595 70  -0.516  0.6073
 (In-phase) - (Out-of-phase)   0.0655 0.0586 70   1.119  0.2670
```


The post-hoc analysis revealed that only the connectivity between the
raPFC - rdlPFC was significantly different in the frontal stimulation
condition as compared to sham stimulation.

We further looked on this particular connection in an individual
model including pre-post stimulation fMRI connectivity measures for the
frontal and sham stimulation. Therefore, in this model, there are
variables stim\_protocol, Time and their interaction Time\*Stim protocol.
We again build different models, including random slopes and random
intercepts.


```
iCOG_Clear_BA<-read.csv2("iCOG_fMRI_FPCN_before_after.csv")

names(iCOG_Clear_BA)[names(iCOG_Clear_BA)=="ď.żID_subject"]<-"ID_subject"
iCOG_Clear_BA$Stim_protocol=as.factor(iCOG_Clear_BA$Stim_protocol)
iCOG_Clear_BA$Stim_protocol<-factor(iCOG_Clear_BA$Stim_protocol, labels = c("Sham", "Frontal", "Parietal", "In-phase", "Out-of-phase"))
iCOG_Clear_BA$Time=as.factor(iCOG_Clear_BA$Time)
iCOG_Clear_BA_frontal = subset(iCOG_Clear_BA,iCOG_Clear_BA$Stim_protocol %in% c("Sham","Frontal"))


form0 = paste("B_raPFCrdlPFC" ," ~ Stim_protocol*Time")
  signif_model0_BA_frontal = lm(form0, data=iCOG_Clear_BA_frontal)
  
anova(signif_model0_BA_frontal)
```


```
Analysis of Variance Table

Response: B_raPFCrdlPFC
                   Df  Sum Sq  Mean Sq F value Pr(>F)
Stim_protocol       1 0.00061 0.000612  0.0147 0.9040
Time                1 0.01180 0.011795  0.2830 0.5970
Stim_protocol:Time  1 0.07459 0.074590  1.7893 0.1868
Residuals          52 2.16768 0.041686
```


```
AIC(signif_model0_BA_frontal)
```


```
[1] -13.17388
```


```
form1 = paste("B_raPFCrdlPFC" ," ~ Stim_protocol*Time + (1|ID_subject)")
signif_model1_BA_frontal = lmer(form1, data=iCOG_Clear_BA_frontal)
  
anova(signif_model1_BA_frontal)
```


```
Type III Analysis of Variance Table with Satterthwaite's method
                     Sum Sq  Mean Sq NumDF  DenDF F value  Pr(>F)  
Stim_protocol      0.000075 0.000075     1 39.744  0.0044 0.94750  
Time               0.011795 0.011795     1 37.001  0.6922 0.41076  
Stim_protocol:Time 0.074590 0.074590     1 37.001  4.3772 0.04334 *
---
Signif. codes:  0 ‘***’ 0.001 ‘**’ 0.01 ‘*’ 0.05 ‘.’ 0.1 ‘ ’ 1
```


```
AIC(signif_model1_BA_frontal)
```


```
[1] -15.03311
```


```
form2 = paste("B_raPFCrdlPFC" ," ~ Stim_protocol*Time + (ID_Session_6to1|ID_subject)")
signif_model2_BA_frontal = lmer(form2, data=iCOG_Clear_BA_frontal)
```


```
boundary (singular) fit: see help('isSingular')
```


```
anova(signif_model2_BA_frontal)
```


```
Type III Analysis of Variance Table with Satterthwaite's method
                     Sum Sq  Mean Sq NumDF  DenDF F value  Pr(>F)  
Stim_protocol      0.000264 0.000264     1 39.565  0.0161 0.89964  
Time               0.011795 0.011795     1 36.509  0.7194 0.40187  
Stim_protocol:Time 0.074590 0.074590     1 36.509  4.5492 0.03973 *
---
Signif. codes:  0 ‘***’ 0.001 ‘**’ 0.01 ‘*’ 0.05 ‘.’ 0.1 ‘ ’ 1
```


```
AIC(signif_model2_BA_frontal)
```


```
[1] -12.11563
```


The model 1, which includes fixed effects and a random intercept for
each individual seems to be the best model. We will further look into
pairwise comparisons for this model.


```
emm_interaction__signif_model1_BA_frontal <- emmeans(signif_model1_BA_frontal,~ Stim_protocol*Time )
P_pairwaise_comparison_raPFCrdlPFC_frontal<-pairs(emm_interaction__signif_model1_BA_frontal, adjust = 'none')
P_pairwaise_comparison_raPFCrdlPFC_frontal
```


```
 contrast                      estimate     SE   df t.ratio p.value
 Sham Time1 - Frontal Time1     -0.0754 0.0509 38.5  -1.481  0.1468
 Sham Time1 - Sham Time2        -0.0440 0.0493 37.0  -0.891  0.3786
 Sham Time1 - Frontal Time2      0.0266 0.0509 38.5   0.522  0.6048
 Frontal Time1 - Sham Time2      0.0315 0.0509 38.5   0.618  0.5404
 Frontal Time1 - Frontal Time2   0.1020 0.0493 37.0   2.068  0.0457
 Sham Time2 - Frontal Time2      0.0705 0.0509 38.5   1.385  0.1741

Degrees-of-freedom method: kenward-roger
```


LS0tDQp0aXRsZTogIlJfTm90ZWJvb2tfZm9yX2lDT0dfZk1SSV9hbmFseXNpcyINCm91dHB1dDogaHRtbF9ub3RlYm9vaw0KLS0tDQoNCmBgYHtyfQ0KbGlicmFyeShNYXRyaXgpDQpsaWJyYXJ5KGxtZTQpDQpsaWJyYXJ5KGNhckRhdGEpDQpsaWJyYXJ5KGNhcikNCmxpYnJhcnkobG1lclRlc3QpDQpsaWJyYXJ5KGVtbWVhbnMpDQpsaWJyYXJ5KGVmZmVjdHNpemUpDQpsaWJyYXJ5KGdncGxvdDIpDQpsaWJyYXJ5KFJtaXNjKQ0KbGlicmFyeShubG1lKQ0KbGlicmFyeShyZXNoYXBlKQ0KYGBgDQoNCmBgYHtyfQ0Kc2V0d2QoIn4vaUNPRy9XUDEvZGF0YS9ycy1mTVJJIikNCmlDT0dfQ2xlYXI8LXJlYWQuY3N2MigiaUNPR19mTVJJX0ZQQ05fZGlmZmVyZW5jZXMuY3N2IikNCm5hbWVzKGlDT0dfQ2xlYXIpW25hbWVzKGlDT0dfQ2xlYXIpPT0ixI8uxbxJRF9zdWJqZWN0Il08LSJJRF9zdWJqZWN0Ig0KaGVhZChpQ09HX0NsZWFyLDUpDQpgYGANCg0KYGBge3J9DQppQ09HX0NsZWFyJFN0aW1fcHJvdG9jb2w9YXMuZmFjdG9yKGlDT0dfQ2xlYXIkU3RpbV9wcm90b2NvbCkNCmlDT0dfQ2xlYXIkU3RpbV9wcm90b2NvbDwtZmFjdG9yKGlDT0dfQ2xlYXIkU3RpbV9wcm90b2NvbCwgbGFiZWxzID0gYygiU2hhbSIsICJGcm9udGFsIiwgIlBhcmlldGFsIiwgIkluLXBoYXNlIiwgIk91dC1vZi1waGFzZSIpKQ0KbGV2ZWxzKGlDT0dfQ2xlYXIkU3RpbV9wcm90b2NvbCkNCmBgYA0KDQoqKkNvbm5lY3Rpdml0eSB3aXRoaW4gRlBDTiBuZXR3b3JrIHN0YXRpc3RpY3MqKg0KDQpbU2ltcGxlIG1vZGVsLCBhdmVyYWdlc117LnVuZGVybGluZX0NCg0KSGVyZSwgd2UgcHJlc2VudCBkaWZmZXJlbnQgbW9kZWxzIHRvIGNoYXJhY3Rlcml6ZSBlYWNoIHJlbGV2YW50IHBhcmFtZXRlciByZWxhdGVkIHRvIHRoZSBwYXJ0aWNpcGFudHMnIGNvbm5lY3Rpdml0eSB3aXRoaW5nIEZQQ04gbmV0d29yay4gSW4gZWFjaCBjYXNlLCB0aGUgbW9kZWxzIHN0YXJ0IG9mZiBmcm9tIGEgc2ltcGxlIGZvcm0gKGkuZS4sIGdyb3VwLWF2ZXJhZ2VkIGRpZmZlcmVuY2llcyBhZnRlci1iZWZvcmUgdGhlIHN0aW11bGF0aW9uIHBlciBzZXNzaW9uKSwgYW5kIHRoZXkgZ3JvdyBpbiBjb21wbGV4aXR5IGFzIHdlIGFkZCBvdGhlciByYW5kb20gZWZmZWN0cyB0byBhY2NvdW50IGZvciB0aGUgdmFyaWFiaWxpdHkgYWNyb3NzIHN1YmplY3RzLiBFYWNoIG1vZGVsIGlzIGNvbXBhcmVkIHRvIGl0cyAoc2ltcGxlcikgcHJlZGVjZXNzb3IgdG8gYXNzZXNzIHdoZXRoZXIgdGhlIGV2ZW50dWFsIGltcHJvdmVtZW50IGluIGRhdGEgZXhwbGFpbmFiaWxpdHksIGFzIHF1YW50aWZpZWQgYnkgdGhlIGNvbnZlbnRpb25hbCBjcml0ZXJpYSAoaS5lLiwgQWthaWtlJ3MgaW5mb3JtYXRpb24gY3JpdGVyaWEpLCBqdXN0aWZpZXMgdGhlIHVzZSBvZiBhIG1vcmUgY29tcGxleCBtb2RlbC4NCg0KTW9kZWwgMCBpbmNsdWRpbmcgYWxsIHN0aW11bGF0aW9uIHByb3RvY29scyB0byBzZWUgd2hpY2ggb25lIHdvdWxkIGJlIGRpZmZlcmVudCBmcm9tIHBsYWNlYm8gc3RpbXVsYXRpb24NCg0KYGBge3J9DQp2YXJzID0gbmFtZXMoaUNPR19DbGVhcls0MjpsZW5ndGgobmFtZXMoaUNPR19DbGVhcikpXSkNCg0KbW9kZWxzMF9kaWZmID0gbGFwcGx5KHNldE5hbWVzKHZhcnMsdmFycyksIGZ1bmN0aW9uKHZhcikgew0KICBmb3JtID0gcGFzdGUodmFyICwiIH4gU3RpbV9wcm90b2NvbCIpDQogIGxtKGZvcm0sIGRhdGE9aUNPR19DbGVhcikNCn0pDQphbm92YV9tb2RlbHMwX2RpZmY8LXNhcHBseShtb2RlbHMwX2RpZmYsYW5vdmEpDQphbm92YV9tb2RlbHMwX3BfZGlmZjwtYW5vdmFfbW9kZWxzMF9kaWZmWzUsXQ0KcF9MTU1fdGFibGVfbW9kZWxzMF9kaWZmPC1kYXRhLmZyYW1lKHQoc2FwcGx5KGFub3ZhX21vZGVsczBfcF9kaWZmLGMpKSkNCnBfTE1NX3RhYmxlX21vZGVsczBfZGlmZg0KQUlDX21vZGVsczBfZGlmZjwtc2FwcGx5KG1vZGVsczBfZGlmZixBSUMpDQpgYGANCg0KW01vZGVsIDFdey51bmRlcmxpbmV9IC0gcmFuZG9tIGVmZmVjdCBvZiBJRF9TdWJqZWN0LCBpbmNsdWRpbmcgYWxsIHN0aW11bGF0aW9uIHByb3RvY29scyB0byBzZWUgd2hpY2ggb25lIHdvdWxkIGJlIGRpZmZlcmVudCBmcm9tIHBsYWNlYm8gc3RpbXVsYXRpb24uIFAtdmFsdWVzIG9mIHRoZQ0KDQpgYGB7cn0NCm1vZGVsczFfZGlmZiA9IGxhcHBseShzZXROYW1lcyh2YXJzLHZhcnMpLCBmdW5jdGlvbih2YXIpIHsNCiAgZm9ybSA9IHBhc3RlKHZhciAsIiB+IFN0aW1fcHJvdG9jb2wrKDF8SURfc3ViamVjdCkiKQ0KICBsbWVyKGZvcm0sIGRhdGE9aUNPR19DbGVhcikNCn0pDQoNCmFub3ZhX21vZGVsczFfZGlmZjwtc2FwcGx5KG1vZGVsczFfZGlmZixhbm92YSkNCmFub3ZhX21vZGVsczFfcF9kaWZmPC1hbm92YV9tb2RlbHMxX2RpZmZbNixdDQpwX0xNTV90YWJsZV9tb2RlbHMxX2RpZmY8LWRhdGEuZnJhbWUodChsYXBwbHkoYW5vdmFfbW9kZWxzMV9wX2RpZmYsYykpKQ0KcF9MTU1fdGFibGVfbW9kZWxzMV9kaWZmDQpBSUNfbW9kZWxzMV9kaWZmPC1zYXBwbHkobW9kZWxzMV9kaWZmLEFJQykNCmBgYA0KDQpXaGVuIHVzaW5nIHRoaXMgbW9yZSBjb21wbGV4IG1vZGVsLCBhIHByb2JsZW0gd2l0aCBzaW5ndWxhcml0eSBldm9sdmVzIGZvciBhbGwgdGhlIGNvbm5lY3Rpb25zLiBXZSB3aWxsIHRoZXJlZm9yZSBlbmQgdGhlIHByb2Nlc3Mgb2YgbW9kZWwgc2VsZWN0aW9uIGluIHRoaXMgc3RlcCwgY2hvb3NpbmcgdGhlIG1vZGVsIDAgYXMgb3VyIGZpbmFsIG1vZGVsLg0KDQpXZSB3aWxsIG5vdyBkbyB0aGUgcG9zdC1ob2MgdGVzdHMgZm9yIHRoZSBzaWduaWZpY2FudCBjb25uZWN0aXZpdHkgc2VlZCBwYWlycyAocmFQRkNsYUlQTCxyYVBGQ3JkbFBGQyApLCB0byBmaW5kIG91dCB3aGVyZSB0aGVzZSBkaWZmZXJlbmNlcyBjb21lIGZyb20uDQoNCmBgYHtyfQ0Kc2lnX3ZhcnM9IGMoInJhUEZDbGFJUEwiLCJyYVBGQ3JkbFBGQyIpDQoNCnNpZ19tb2RlbHMwX2RpZmYgPSBsYXBwbHkoc2V0TmFtZXMoc2lnX3ZhcnMsc2lnX3ZhcnMpLCBmdW5jdGlvbih2YXIpIHsNCiAgZm9ybSA9IHBhc3RlKHZhciAsIiB+IFN0aW1fcHJvdG9jb2wiKQ0KICBsbShmb3JtLCBkYXRhPWlDT0dfQ2xlYXIpDQp9KQ0KDQplbW1fc3RpbV9wcm90b2NvbF9zaWdfbW9kZWwwX2RpZmYgPC0gc2FwcGx5KHNpZ19tb2RlbHMwX2RpZmYsIGVtbWVhbnMsIH5TdGltX3Byb3RvY29sKQ0KUF9wYWlyd2Fpc2VfY29tcGFyaXNvbl9fc3RpbV9wcm90b2NvbF9zaWduaWZfbW9kZWwwX2RpZmY8LXNhcHBseShlbW1fc3RpbV9wcm90b2NvbF9zaWdfbW9kZWwwX2RpZmYsIHBhaXJzICxhZGp1c3QgPSAibm9uZSIpDQpQX3BhaXJ3YWlzZV9jb21wYXJpc29uX19zdGltX3Byb3RvY29sX3NpZ25pZl9tb2RlbDBfZGlmZg0KDQpgYGANCg0KVGhlIHBvc3QtaG9jIGFuYWx5c2lzIHJldmVhbGVkIHRoYXQgb25seSB0aGUgY29ubmVjdGl2aXR5IGJldHdlZW4gdGhlIHJhUEZDIC0gcmRsUEZDIHdhcyBzaWduaWZpY2FudGx5IGRpZmZlcmVudCBpbiB0aGUgZnJvbnRhbCBzdGltdWxhdGlvbiBjb25kaXRpb24gYXMgY29tcGFyZWQgdG8gc2hhbSBzdGltdWxhdGlvbi4NCg0KV2UgZnVydGhlciBsb29rZWQgb24gdGhpcyBwYXJ0aWN1bGFyIGNvbm5lY3Rpb24gaW4gYW4gaW5kaXZpZHVhbCBtb2RlbCBpbmNsdWRpbmcgcHJlLXBvc3Qgc3RpbXVsYXRpb24gZk1SSSBjb25uZWN0aXZpdHkgbWVhc3VyZXMgZm9yIHRoZSBmcm9udGFsIGFuZCBzaGFtIHN0aW11bGF0aW9uLiBUaGVyZWZvcmUsIGluIHRoaXMgbW9kZWwsIHRoZXJlIGFyZSB2YXJpYWJsZXMgc3RpbV9wcm90b2NvbCwgVGltZSBhbmQgdGhlaXIgaW50ZXJhY3Rpb24gVGltZVwqU3RpbSBwcm90b2NvbC4gV2UgYWdhaW4gYnVpbGQgZGlmZmVyZW50IG1vZGVscywgaW5jbHVkaW5nIHJhbmRvbSBzbG9wZXMgYW5kIHJhbmRvbSBpbnRlcmNlcHRzLg0KDQpgYGB7cn0NCmlDT0dfQ2xlYXJfQkE8LXJlYWQuY3N2MigiaUNPR19mTVJJX0ZQQ05fYmVmb3JlX2FmdGVyLmNzdiIpDQoNCm5hbWVzKGlDT0dfQ2xlYXJfQkEpW25hbWVzKGlDT0dfQ2xlYXJfQkEpPT0ixI8uxbxJRF9zdWJqZWN0Il08LSJJRF9zdWJqZWN0Ig0KaUNPR19DbGVhcl9CQSRTdGltX3Byb3RvY29sPWFzLmZhY3RvcihpQ09HX0NsZWFyX0JBJFN0aW1fcHJvdG9jb2wpDQppQ09HX0NsZWFyX0JBJFN0aW1fcHJvdG9jb2w8LWZhY3RvcihpQ09HX0NsZWFyX0JBJFN0aW1fcHJvdG9jb2wsIGxhYmVscyA9IGMoIlNoYW0iLCAiRnJvbnRhbCIsICJQYXJpZXRhbCIsICJJbi1waGFzZSIsICJPdXQtb2YtcGhhc2UiKSkNCmlDT0dfQ2xlYXJfQkEkVGltZT1hcy5mYWN0b3IoaUNPR19DbGVhcl9CQSRUaW1lKQ0KaUNPR19DbGVhcl9CQV9mcm9udGFsID0gc3Vic2V0KGlDT0dfQ2xlYXJfQkEsaUNPR19DbGVhcl9CQSRTdGltX3Byb3RvY29sICVpbiUgYygiU2hhbSIsIkZyb250YWwiKSkNCg0KDQpmb3JtMCA9IHBhc3RlKCJCX3JhUEZDcmRsUEZDIiAsIiB+IFN0aW1fcHJvdG9jb2wqVGltZSIpDQogIHNpZ25pZl9tb2RlbDBfQkFfZnJvbnRhbCA9IGxtKGZvcm0wLCBkYXRhPWlDT0dfQ2xlYXJfQkFfZnJvbnRhbCkNCiAgDQphbm92YShzaWduaWZfbW9kZWwwX0JBX2Zyb250YWwpDQpBSUMoc2lnbmlmX21vZGVsMF9CQV9mcm9udGFsKSANCg0KZm9ybTEgPSBwYXN0ZSgiQl9yYVBGQ3JkbFBGQyIgLCIgfiBTdGltX3Byb3RvY29sKlRpbWUgKyAoMXxJRF9zdWJqZWN0KSIpDQpzaWduaWZfbW9kZWwxX0JBX2Zyb250YWwgPSBsbWVyKGZvcm0xLCBkYXRhPWlDT0dfQ2xlYXJfQkFfZnJvbnRhbCkNCiAgDQphbm92YShzaWduaWZfbW9kZWwxX0JBX2Zyb250YWwpDQpBSUMoc2lnbmlmX21vZGVsMV9CQV9mcm9udGFsKSAgDQoNCmZvcm0yID0gcGFzdGUoIkJfcmFQRkNyZGxQRkMiICwiIH4gU3RpbV9wcm90b2NvbCpUaW1lICsgKElEX1Nlc3Npb25fNnRvMXxJRF9zdWJqZWN0KSIpDQpzaWduaWZfbW9kZWwyX0JBX2Zyb250YWwgPSBsbWVyKGZvcm0yLCBkYXRhPWlDT0dfQ2xlYXJfQkFfZnJvbnRhbCkNCiAgDQphbm92YShzaWduaWZfbW9kZWwyX0JBX2Zyb250YWwpDQpBSUMoc2lnbmlmX21vZGVsMl9CQV9mcm9udGFsKQ0KYGBgDQoNClRoZSBtb2RlbCAxLCB3aGljaCBpbmNsdWRlcyBmaXhlZCBlZmZlY3RzIGFuZCBhIHJhbmRvbSBpbnRlcmNlcHQgZm9yIGVhY2ggaW5kaXZpZHVhbCBzZWVtcyB0byBiZSB0aGUgYmVzdCBtb2RlbC4gV2Ugd2lsbCBmdXJ0aGVyIGxvb2sgaW50byBwYWlyd2lzZSBjb21wYXJpc29ucyBmb3IgdGhpcyBtb2RlbC4NCg0KYGBge3J9DQplbW1faW50ZXJhY3Rpb25fX3NpZ25pZl9tb2RlbDFfQkFfZnJvbnRhbCA8LSBlbW1lYW5zKHNpZ25pZl9tb2RlbDFfQkFfZnJvbnRhbCx+IFN0aW1fcHJvdG9jb2wqVGltZSApDQpQX3BhaXJ3YWlzZV9jb21wYXJpc29uX3JhUEZDcmRsUEZDX2Zyb250YWw8LXBhaXJzKGVtbV9pbnRlcmFjdGlvbl9fc2lnbmlmX21vZGVsMV9CQV9mcm9udGFsLCBhZGp1c3QgPSAnbm9uZScpDQpQX3BhaXJ3YWlzZV9jb21wYXJpc29uX3JhUEZDcmRsUEZDX2Zyb250YWwNCmBgYA0K
